# Supplementary material for: Grief reaction and psychosocial impacts of child death and stillbirth on bereaved North Indian parents: A qualitative study
Source: PLoS One. 2021 Jan 27;16(1):e0240270. doi: 10.1371/journal.pone.0240270 (PMC7840017; doi:10.1371/journal.pone.0240270)
Supplement: S2 File — (PDF) [file pone.0240270.s002.pdf]

List of In-depth Interviews- Parents of deceased children

S. No.	Participants	Age	Religion
1	Parents 1	Mother-20-25 years<br>Father-25-30 years	Hindu
2	Parents 2	Mother-20-25 years<br>Father-25-30 years	Christian
3	Parents 3	Mother-20-25 years<br>Father-20-25 years	Muslim
4	Parents 4	Mother-25-30 years<br>Father-25-30 years	Hindu
5	Parents 5	Mother-25-30 years<br>Father-25-30 years	Muslim
6	Parents 6	Mother-20-25 years<br>Father-25-30 years	Muslim
7	Parents 7	Mother-25-30 years<br>Father-30-35 years	Hindu
8	Parents 8	Mother-30-35 years	Hindu
9	Parents 9	Mother-30-35 years	Muslim
10	Parents 10	Mother-25-30 years<br>Father-35-40 years	Hindu
11	Parents 11	Mother-20-25 years<br>Father-25-30 years	Muslim
12	Parents 12	Mother-25-30 years<br>Father-30-35 years	Hindu
13	Parents 13	Mother-35-40 years<br>Father-40-45 years	Hindu

In-depth Interview- Parents of Deceased Child 1

1. **Demography**

**1.1**	**Primary respondent (relation with the child)**	Mother, Father
**1.2**	**Age of the primary respondent**	Mother-20-25 years<br>Father-25-30 years
**1.3**	**Other members present**	Nani, Maternal uncle (mama of child)

2.1 Where do you usually go or whom do you consult during the illness of your child?
Mother- There are many doctors here, people go to the doctor which they like. There are many more doctors here.
2.2 Where do you usually go or whom do you consult for vaccination of your children?
Mother- Everybody goes to the Anganwadi center for vaccination.
2.4 Please specify the reasons for the preference for the facility and/or healthcare provider.
Mother- There is lot of traffic here, we cannot take him at distance so, if the child is less sick then we take him to nearby doctor.
3.1 Can you please tell us know about the illness and events that led to death of <name> your child?
Mother- Our child got ill, as when he got vaccinated at Aangawadi center after that he got swelling, then we used to do ice fomentation (NANI- swelling was there), then we took him for check-up at Aangawadi centre, it was not getting treated even after 20 days. Then doctor there said to seek advice of other. Then we took him to doctor and then doctor gave the tube to be applied and advised to do ice fomentation. Then we used to apply the tube, then it was getting less and still little was left. Child had a fever that was shown to the private doctor, then doctor said to give medicine, he gave fever medicine bottle, and then also the fever was coming. After doing this, he left drinking milk one day, then he used to drink it with spoon, I used to give him my milk only. He used to weep, one day he started crying a lot and not drinking milk then we showed him to doctor in Sarita Vihar where everyone was saying to take him. Then, doctor gave medicines to eat, and he checked everything, they checked ears, nose and then said all is right, then I said that he does not drink milk and he had fever. I gave him milk with spoon one by one, then he fell asleep. Then after sleeping in afternoon I gave him milk, then he again slept and woke up in the evening then I fed him milk, I said to my mother that now he is drinking milk, then she said to feed him with the spoon. Then when I was feeding him, he drank some milk, during drinking milk he started shouting and crying loudly. Then I took him in the lap and tried to counsel him, then his hand and legs started getting tight.
After looking at him, all of us started shouting and then we took him to the doctor immediately, the old doctor, he checks everything and then we quickly took them him to that doctor. I was not able to understand anything at that time, we were the mother daughter alone at that time. The doctor said and told that it is a problem of breathing. He said, take him to K Hospital early, so we said okay. Then we came home and we could not find any car, then the brother who stay nearby took us. It was Monday, and there was a lot of traffic, and we got struck for 2-2.30 hours. Our child was shouting from home and also on the way, he did not stopped crying, as soon as we reached hospital his voice stopped but he was breathing. Then I took him inside and called doctor, then all doctor put him on machine, then doctor gave me the machine to press it. Then for 3-4 hours he was breathing. Then around 12-12.30 he was not alive. We took him at 7 o clock from our home and reached at 9 pm, due to traffic. We went to hospital with our neighbor.
3.2 Who all supported you during the illness and hospitalization of your child?
Mother- When I went to the hospital, my husband was with me, my father, my mother and neighbor went along with to the hospital.
4.1 Now we would request you to tell us something about the period of hospitalization for your child? (Probe: hospital, ward, duration of stay, course of illness)
Mother- The hospital took a sign and asked what happened to the child, he asked if the child ever turned blue, then we said no, did the child get a high fever, or such a problem had happened earlier So, I told that

it had not happened before, this all happened after vaccination, he got fever and after that all this happened. He had played for two months, after vaccination in the second month, he had all this condition.
<duration of stay>
From night 8 till 1 o clock we were in hospital. At 12.30am he died, after that they told us it will take half an hour to do paper formality, then they will leave us. One report was still not available, they said it will take 5 hours to give the report to us, but we said we cannot wait for 5 hours.
We didn't know that he has died, my husband told me that doctor has said that you should take your child to other hospital. Then we left hospital at 12.30am. We got one report, and it was mentioned in that there is blood infection. And we didn't take 2nd report as it said to be given after 5 hours.
Nani – He was dead so we came, these people did not tell us that it has died.
4.3Could you please describe your experience about communication by various hospital staffs during hospital stay? (Who all communicated, who was the primary communicator, nurses, doctors, other staffs, frequency of communication)
Mother- behavior of doctor was good. When doctor provided all things then, they were all looking at other children also, they were not looking at one child only. Cleanliness was fine, it was clean. No money was spent.
<nurse>
Mother- we need to tell nurse that our child is not moving. Nurse just gave the bottle of water injected, and said your child will be cured.
<primary communicator child health Well, no one would tell, doctor called my husband and explained everything, that there is such condition, there is no hope of survival, and after this they took signature. Then half an hour later, he died.
<did you understood what doctor explained> |
Father- When I did not understand what to do, I understand that time which I understood, took the signature and then went away.
5.1 Could you please tell us about the death and death declaration of your child? (Probe: who declared death, the body language and expression, preceding events before death)
<who declared death>
Mother- she was looking as head doctor, as nurse and other person were telling about the things they gave to the child.
<body language & expression> Mother- I had heard that time too, from them that let the mother of child go out then we will discharge, this all is said by doctor.
5.2 What was your and your family member's reaction to the death of your child?
We didn't know when death happened, my husband knew about that he was unable to speak he was not able to come in front of us. Even my father knew about that, he was also not able to speak. They didn't say anything because we would have started crying in hospital. Even doctor told not to tell mother here, the doctor said, please take mother out, then we will give you discharge. Then I came outside, then my husband came after discharge formality.
5.3 Do you blame someone/something for the death of your child? (Probe: who or what is it, what did you do in this context)
Mother- whom should we blame
Father- we can blame, the person who vaccinated the child, who gave the medicine (mother- we went to khadar village for medicine), today he got ill we got him medicine then he drank milk, then slept, then in evening the problem started, then when he woke up and then he started shouting.
Mother- This all has happened in one day. We gave medicine and after that he woke up in evening, and all this started happening.
Father- There was no problem before.
Mother- Whom we should blame either this doctor or that doctor? We have not got the report, so how can we know what was the reason and how did this happen.

5.3 How was your experience about the procedure after death till handover of the body to you? (Probe: processes, documentation and papers given, respect for religious norms, time taken, cost/payment, assistance from the staffs)
Mother- It took half an hour.
Father- We did not get any slip, we only got one report, we got one slip to take the child out of hospital.
Nani- At 12.30 -1 we took the auto, and we came home at 2 am in night.
Father- When in hospital how can stay long, I made the excuse to my wife and mother-in-law, came with stone on my heart, these people will be separated then who will handle them, we were already in tension. So, I didn't tell anyone that child is dead, as if I would told them they would have started crying and shouting there.
5.4 Who all from your family and community/neighborhood supported you during this period? (Probe: who informed them, who all came, what type of support given)
Mother- our whole family was there like, my mother, father and my husband.
5.5 Is/there any change in the interpersonal relationship between you and your spouse and other family members after the event
Nothing has changed like this my husband tells me that what is there in life, it can happen to anyone in life. It is not necessary that we keep sitting on the same thing and do not do something. He says that you should be happy, the thing which happened has happened, what to do now, new beginning can happen again everyone tells that. I will tell my sadness to my husband, he is supporting me.
6.1 What all rituals and procedures did you follow after death of your child? (Probe: body preparation, who prepared, burial/ cremation, other rituals and timings; who performed them according to the rituals)
Mother- All night child was at home, then around 6-7 in morning we take him for cremation. No bathing was performed, because it is said this all is not performed for the child. No worship is performed, only essence sticks are Enlighted, then we bury him, then nothing was done. We didn't cook for 4-5 days.
6.2 How did you, your spouse (wife/husband) and immediate family members cope with the difficult phase?
Mother- Both of us handle each other, we understand. My husband had started working on 15-20 days later. As it is not yet engaged in the company, he is still learning. So, I said him to resume his work as how we will run the house.
6.3 How did the family members support you and your wife during the difficult phase? (Probe: burial/cremation rituals, community norms/rituals, coping with the phase, financial)
Mother- My father was present with my other relatives and all other family member was there. My mother-in-law also came.
Father- My family stays in the village, they came later, 7-8 members came from the village.
6.4 What were the reactions/ counselling and suggestions given by the relatives/neighbours/community after the episode?
Mother- The neighbors were saying that they should took the child to a big hospital or takes something else. Someone said that they should have taken the child to Apollo, someone said to take Kalawati. So, I said which hospital is bigger than this hospital?
Father- In hospital all check-up is done. My wife delivery and check-up everything happened there only. So, which hospital is bigger than that?
<knowing the cause> Mother- Neighbor says that child was suffering from pneumonia, but I said if this was the reason then this would have appeared in the report. And we would take-care of everything, we avoid putting him on floor, avoid taking out he was 2 months old, we would keep him inside as there was air outside. He was weak when he was born his weight was 2 kg, and after one and half months his weight was 3.30 kg. We thought when he will be of 3 months then we will take him outside.
6.5 How your and your family life changed after the death of the child?
No such change has happened, we live as we were living before, they only explain to me, yet remember the child and cries and explains to me. We give support to each other and tell each other we should live like this, what else we can do, if anything happens we will tell each other only. My husband is such that

he remain happy in sadness and in happiness. He doesn't share his sadness with anyone. He says if he will share his sadness everyone at home will remain unhappy, so if he hides his sadness everyone will remain happy. But man will always be in tension, he cannot forget everything so soon.

7.1 What was the cause of death of your child and what is your perception about the value of knowing the cause? (Probe: knowledge about the cause of death, willingness to know the detailed cause, potential factors that led to the illness/death, potential impact on the other family members or next pregnancy)

Mother- My delivery was normal. Only after vaccination, we were in trouble, because after that he developed fever, we took him to the doctor and doctor gave the medicine of fever.

<willingness to know>

Mother- the cause of the death must be known, that how it happened. We didn't even go to collect the report because there is delay in giving report. Whole day is spent, and there is a lot of traffic and there report is not given on time.

<reason told by doctor>

Mother- The doctor told the infection in the blood and explained the problem of breathing.

7.2 In your view, what more could have been done to know the exact cause of death and the reasons causing the disease? (Probe: investigation, autopsy, any other)

Mother- If we find that report then we would know something from the report.

7.3 Are you aware of any method for identifying the exact cause of death? (Probe: autopsy, investigations, etc.)

Mother- We have heard, post mortem is performed for some accident or some incident, we have not heard that it is performed for some disease.

Father- for the disease, even I have not heard.

7.4 What are your views about the autopsy/ post-mortem for identifying the cause of death? (Probe: necessity, religious aspect, time needed, disfigurement, etc.)

Mother- we don't want tearing thing. We do not know, there is something different, in the last rites of post mortem body. We do not know anything like this. We have never seen anything like that.

7.5 What are your views about collection of the tissue (like done for biopsy to diagnose diseases) and body fluid samples for identifying the cause of death? (Probe: necessity, religious aspect, time needed, disfigurement, etc.)

Mother- if they do not tell us it is fine, we cannot see in front of our eyes. And that time if someone has told we and we would know the reason then we would have agreed.

Maternal uncle- There was nobody to tell that time about MITS. If the doctor told us, then we would have agreed, but we do not have any information about it.

7.6 How parents and family members can be approached for the tissue and fluid sampling? (Probe: who should approach, whom, when and how should approach; what should be told and in what detail; should some picture of the method be used to explain)

Mother- That time parents are not in such condition. My father or some relatives they would explain us. In hospital if head doctor tells us about this it is ok.

7.7 In your view, what factors are important for acceptance of the tissue sampling by the parents and family members? (Probe: desire to know the cause, trust, confidence on the health care provider)

Mother- We also used to think that the person who has gone is gone and the time has passed, now what we will do after taking the report, but in our heart, it is the wish that if we had brought that report, then we would have known what was cause of death and we would have become aware of the future.

Maternal uncle- if some person falls ill, this happens step-wise, we know person was not well, but if everything happens in one day, then we don't understand anything.

Mother- Therefore, we wanted to get the report, as there is still we are in our mind that we should have get the report, and then we will become aware in the future.

<trust about tissue sampling>

Maternal uncle- In everyone's mind postmortem is about tearing out the body and body parts are taken out. More than half the people are scared. All these things have ended a man's life and now tearing will take place.

8.1 In your view, what can be done to improve identification of cause of death and efforts to reduce the deaths in children?

Mother- Only after seeing the report will we know that how it happened.

Maternal uncle- Could this be the reason that we have shifted our sister house when the child was born?

Mother- As we were living in rented house and we shifted their 1-2 days before, so could that be the reason of our child getting ill. Then, the people used to say that there is something in that house. But, we don’t in all these things, but people will speak what they think.

Maternal uncle- But, this could not be due to the house.

7.1 In routine day to day operation who takes decision about the major activities about the family?

my father takes decision about major activites as my in laws stays in village. Everyone has a decision for treatment and everyone speaks, who should take it to the doctor and whom to show. Everyone speaks including my husband, father, brother and mother.

7.2 During the hospital stay who discussed with doctors/nurses to make the decision about treatment and other procedures

At that time nobody was at home, only me and my mother was there, my neighbor who is like brother accompanied me to hospital. i took the decision during that time for reports, admission and for making slip. After sometime my father, husband came to hospital. My father was doing al work in hospital, my husband was sitting when doctor came for inquiry.

7.3 After death of your child, who decided about the cremation and other post-death activities and rituals

My father was taking all decisions during cremation, he didnt discuss anything with anyone because he was a small child. my mother, neighbor and some relatives was also present, took the child and buried.

In-depth Interview- Parents of deceased children-2

**1.1**	**Primary respondent (relation with the child)**	Mother, Father
**1.2**	**Age of the primary respondent**	Mother-20-25 years<br>Father-25-30 years
**1.6**	**Other members present**	None

2.1 Where do you usually go or whom do you consult during the illness of your child?
People around here refer to the Astha clinic for their child. It is a private clinic and the is the CH Hospital, where they go, and if their condition becomes more serious than, they go to S hospital.
2.2 Where do you usually go or whom do you consult for vaccination of your children?
For the vaccination, we go to the nearby dispensary, or Asha sister or Aganwadi worker come to us for same.
2.4 Please specify the reasons for the preference for the facility and/or healthcare provider.
There is no need to stand in the line, gets their early. Go directly and meet to the doctor. It is private and there is less time consuming. My wife does not know Hindi properly, then I do not have too much time, I have duty also, I work in the restaurant.
3.1 Can you please tell us know about the illness and events that led to death of <name> your child?
<Pneumonia or hole in the heart >
My child ... she had become pneumonia, so we went to the hospital, she was born at home. Then when we went to the hospital, we know that she had a hole in her heart, he did not check it, just said seeing that it could happen. We know all this in Nepal only. Then the doctor said that I have to do echo test, then I said that I cannot live in village so many days because I have the duty, that's why I brought them here. She had pneumonia first then there was a big hospital three or four hours ahead of my village.
I was not here; my wife took my baby. She was there for 8-10 days in hospital. They suspect a hole in baby's heart. Then I told to my wife that let we go back to home otherwise we have to pay hospital charge for next 10 days even baby's condition was not improving. Then we came to home and left untreated till six months. I called my mother and child to Delhi, when I came back and consult at R Hospital. A doctor at Malviya Nagar advised us to go to R hospital because S hospital and A hospital were usually overcrowded. Otherwise I prefer E hospital and we have ESI card also. If we went ESI, it could be free of cost and time saving.
Here, no more expenses incurred but they were demanding 30-35k for heart operation. Then I thought, we have ESI card, we should go there. Still, arranging 30-35k was not a big deal, at cost of baby health. We had arranged blood from 4-5 persons for the surgery and money. Each time when we went for surgery, baby was suffering from cold and fever and doctor postponed the surgery. It was about 5-6 times when doctor cancel the operation and refuse to admit.
3.2 Who all supported you during the illness and hospitalization of your child?
In the whole procedure, my wife and mine are there and sister-in-law came for a short time as she could not be with us for a long time.
4.1 Now we would request you to tell us something about the period of hospitalization for your child? (Probe: hospital, ward, duration of stay, course of illness)
<How did you reach Safdarjung>
At that day, He had fever it was about 4-5 days. Once it got cure by consultation to CH Hospital. After that my wife took him to AS Hospital in my absence. I was then at duty. Nothing improvement had been seen at there. Once a time, when I came from duty around 12-12:30am, then baby's face started swell, his face was not looking normal and had irregular breathing. His face became tilted. Then we were scared and called Ambulance for referral. First I thought to go RML but later on when the situation became worse, we decided to go S Hospital as it was in nearby.
We went to emergency ward. Doctor told ''he had an episode of seizure'' and look our previous report. They were unable to start treatment as earliest. We had a long list of reports, they were copying all. Then they took injection to my baby as she were not sleeping and keep crying all time. Then, they started treatment and shift the baby to ward no 18 at morning. After shifting in the ward,

there was random treatment had happened. We reached at 2 am (night) and she was dead at next day 11:30 am.

4.2 Could you please describe you overall experience about the hospital care? (Probe: investigations, treatment given, satisfaction level, quality of care, cost of care)

<Satisfaction> They know more about this. Either they were giving oxygen or water or vapor. After some time, we had urged to shift baby in emergency but they did not and send me to take medicines. After 9-10 o'clock, Security guards were not allowing us to go in ward, they allow only in case of providing medicines.

My wife was there. Doctors came to ward only after call. Once, Doctor took water from her bone for investigation and told me to take medicine. I went to purchase medicine. It was long que there so became late to return.

A bone test was conducted another was blood test actually blood test was repeated. One report came earlier and baby was dead before second report came.

4.3 Could you please describe your experience about communication by various hospital staffs during hospital stay? (Who all communicated, who was the primary communicator, nurses, doctors, other staffs, frequency of communication)

<Primary communicator> I missed the name of all but in hospital, there was a senor doctor, a young age doctor, a learner and another one. A person was there who always been scolded by seniors.

<Nurse Behavior > <supporting staff> Nurse's behavior was average. There were many people like us in hospital and she had to cater all. But, limited facilities were there, Doctors came there (at ward) later on. Its common among all govt. institution, staffs are not talking politely and finished all task instantly without mind the quality.

5.1 Could you please tell us about the death and death declaration of your child? (Probe: who declared death, the body language and expression, preceding events before death?)

<Who told you> <about declaration of death>

Baby dead at 12:45 AM, he was dead before I reached with medicine. They did not talk about, even after death they (Doctor) continued to inspect baby. I told them that there was no use to check him, baby was dead. They were silent even after. Again I told him to make a discharge card. They did not tell me about death, probably told to my wife. Death was occurred when I took medicine outside from ward. My wife called (Phone) me then but I missed phone as congested network. I call back but could not connect with. When I came back child was dead.

<Wife didn't know Hindi; father was asking in his language about the question asked to her>

<Husband asked to wife about the behavior of doctor during death declaration>

It was average they just told she is no more.

5.2 Do you blame someone/something for the death of your child? (Probe: who or what is it, what did you do in this context)

We can't blame anyone even we are responsible for that. We had tried what we can rest of all also put their effort. Whatever happened was done. We can't blame after that. We were continuing their treatment since their birth. She could have cured at earliest if possible. We went to private doctor also they did not treat after that we went to govt. hospital. We can't say, they were irresponsible because doctor came every time when we called. That's it.

5.3 How was your experience about the procedure after death till handover of the body to you? (Probe: processes, documentation and papers given, respect for religious norms, time taken, cost/payment, assistance from the staffs)

After death, we told to discharge. They took a bit time in packing after death and did not come even after 2-3 call. It took 30-45 min after death to release. After handover the body, we had a shawl to wrap and went directly to crematorium from Ambulance. Did not come to home.

6.1 What all rituals and procedures did you follow after death of your child? (Probe: body preparation, who prepared burial / cremation, other rituals and timings; who performed them according to the rituals)

We are Christen so not followed that (funeral). After death, we did pray and gathering

6.2 How did you, your spouse (wife/husband) and immediate family members cope with the difficult phase?

Some sympathies after came here, some over a phone call. But nothing was possible after death. It's good to leave all things to god, would be better at that time.

6.3 How did the family members support you and your wife during the difficult phase? (Probe: burial/cremation rituals, community norms/rituals, coping with the phase, financial)
Many people gather after that, brother, etc. we took from there at 12:30. Jija ji, Didi was with us. Actually she tied Rakhi. No expense had incurred, as we went from Ambulance.
<Emotional support post death> (religious leader)
My sister and brother were with us and people and the priest were not regular at Church. After getting a call my sisters would visit me. One of my sisters who would live in Saket also came. Father was busy at that day, so his wife was there.
<Body preparation>
Baby did not bathe, they taken directly from the hospital.
6.4 What were the reactions/ counselling and suggestions given by the relatives/neighbours/community after the episode?
<Knowing the cause of death>
Parents told the lady that you were not destined to have the child that is why she left this abode. I had not spent any single penny on the heath care of the child. The only money being spent was on the transportation to various government institutions. Everyone seeing us, first we were at ground floor later on we were not feel well there so shifted to upper floor.
No one told me about this but what we can do after knowing the reason.
7.1 What was the cause of death of your child and what is your perception about the value of knowing the cause? (Probe: knowledge about the cause of death, willingness to know the detailed cause, potential factors that led to the illness/death, potential impact on the other family members or next pregnancy)
As per my understanding, knowing reason is important. It can improvise our next case. She is pregnant and expecting baby by 25th. This learning may be useful there. We can work more confidently in next time. Even it's important for others also. It's for knowledge.
7.2 In your view, what more could have been done to know the exact cause of death and the reasons causing the disease? (Probe: investigation, autopsy, any other)
<Post mortem>
Doctor can do these things to know the death reason and we can do as per our home. Postmortem was needed but we did not.
7.4 What are your views about the autopsy/ post-mortem for identifying the cause of death? (Probe: necessity, religious aspect, time needed, disfigurement, etc.)
<Religious aspect >
We can know all the reason behind the death. I mean if any other reason was there beyond fever. May be other reason was there like wrong meal consumption, wrong medicine or over dose.
All rituals are same. In our case we put it into box. It's nothing affected to postmortem. MITS is not interfering our rituals.
7.5 What are your views about collection of the tissue (like done for biopsy to diagnose diseases) and body fluid samples for identifying the cause of death? (Probe: necessity, religious aspect, time needed, disfigurement, etc.)
<Time needed>
Yes! It could be possible, no harm in this. We could stay 30 min more for this. Even if they told about this we can stay a bit more.
7.6 How parents and family members can be approached for the tissue and fluid sampling? (Probe: who should approach, whom, when and how should approach; what should be told and in what detail; should some picture of the method be used to explain)
We could be convinced from doctor or our brother. After knowing the reason from post mortem, one may feel regret about our effort in order to overcome.
7.7 In your view, what factors are important for acceptance of the tissue sampling by the parents and family members? (Probe: desire to know the cause, trust, confidence on the health care provider)
We can say him like what happened to be done (sympathize), now try MITS to know the exact reason. You must get the answer by this technique. It should be done at the time when they pack (Wrap) and release because after packing, it may be difficulty in MITS. It should be better if they tell after death. It should be in knowledge of their parent. It obviously not happened before death.

8.1 In your view, what can be done to improve children's efforts to reduce deaths and to identify the causes of death?

Doctor can do these thinks to know the death reason, we can’t do anything. He went there with faith. We are not expecting this at there. If you give time, treat good etc. then its ok.

In-depth Interview- Parents of Deceased Child 3

**1.1**	**Primary respondent (relation with the child)**	Mother and Father
**1.2**	**Age of the primary respondent**	Mother-20-25 years<br>Father-20-25 years
**1.3**	**Other members present**	Maternal grand mother

Maternal grandmother- my child was fine, he suffered from cough and fever, then we went to doctor, when we reached senior doctor came and took investigation of child, he said your child is normal. he said the pipe can be removed in 1-2 hour, your child will be fine, then second doctor came, didnt know what she does the nerve burst, doctor said nerve burst has happened. My husband (child maternal grandfather) got expired 6 months ago. i dont have any support, i have 3 daughters and 1 son he takes drugs, why should i lie, i am living in rented house. my husband also died in S hospital.
Mother- I was in bad state, after my son was born i felt like i was born again. my child was born in G hospital, they have all my records of my name, my address they can tell you how i have delivered my child. earlier doctor refused and said you are serious and you may not survive. i had normal delivery with small operation.

2.1 Can you please let us know about the illness and events that led to death of your child?

Child was having cough two days back, we gave him medicine of cough, he got bit relieved, on same day in afternoon his health detoriated and he was also having fever, then we took him to local doctor, he gave medicine and said dont worry your child will become fine.
In the morning his health was getting worse, we was better at night after we gave him medicine, he was even playing, he slept near me on floor, and he was fine. At 6 in morning he was not drinking milk and then at 8 we took him to local doctor. He nebulized the child, he was better after getting nebulized, and then doctor told to give medicine at 9-10. To avoid any reaction I gave medicine at 10:30, as he had one dose in morning. in afternoon his face started becoming white and he was crying, we took him to hospital. We took him to S hospital in emergency ward, he was crying at that time. tell me onething even you can note that point, that if child is not breathing then how he can cry. i doubt on this point, when child can not breathe, i also have breathing problem, when we can not breathe then we cannot even speak. that child was crying so much, he was even crying in my lap. In hospital they said to admit the child, we disagree initially, we said give medicine, we can go home. our heart was not allowing to admit the child here. when i saw my child crying, then i said to nebulize my child, after nebulization he was fine, after that even he slept.
when child was on nebulization one doctor came and started giving oxygen using a pipe other doctor even refused to do this as child was normal and said there is no use for this pipe, the child was crying, doctor inserted the emergency pipe (bag and tube) and removed the nebulizer, and in this process we lost our child. our child died and doctor didnt gave us report from the hospital and we didnt even signed till now.

Hospitalization period

3 Now we would request you to tell us something about the period of hospitalization for your child?

Mother- we stayed for one night at hospital, the child died at 10 in the morning. we went at 4 in evening and next day at 10 in the morning child died, and then we was handed to us. in morning at 8 when doctor saw the child he said your child is normal, he said till 2 we can remove emergency pipe (bag and tube) if child is breathing normally. then i dont know what other doctor at 10 did, he was giving glucose using bottle infront of us, we thought he was giving medicine. tell me onething my child was not given fever medicine then only his fever reached to brain. they said fever is there, thats why brain nerve bursted. hospital think we dont know anything, tell me if they had given medicine on time then fever would not reach in brain. they didnt give medicine on time then only fever reached brain, we took the child to hospital for treatment. he was not having fever at home. fever appeared on the way.
Maternal grndmother- child was breathing fast.

Mother- earlier we didnt know why he was breathing fast. in hospital neighboring bed ladies told about this. we didnt recognise earlier about fast breathing, when we came to know we rushed to hospital.

4 Could you please describe your overall experience about the hospital care?

Mother- The doctor did not perform any test in the hospital, just inserted two needles and glucose. No blood was taken for the test.

Maternal grandmother- We are telling, one doctor told other doctor not to insert the emergency pipe , yet the second doctor put the pipe in child. No money was not spent in hospital. We are not educated that's why we were driven away and was told one person stay inside and other person go outside.

Mother- We do not know which doctor came at 8 o'clock, he was a big doctor or a small doctor, the big one was of black color, when our baby died and the one who had checked and saw the report, this doctor shouted at other doctor that when child and his report was normal how he can die. The big doctor came on round at 10, he was shouting at other doctors and were having some meeting.

5 Could you please describe your experience about communication by the various hospital staffs during hospital stay?

Mother- the doctor inserted the canula, no she was not nurse. When we said why are you putting this, then she scold us and said you take home then, why our child was nebulized, my baby cried, he was getting relived by nebulization, he was sleeping, they took oxygen from baby, when you want to go home, then what is need of oxygen, then we admitted baby and said no problem you can do your procedure. They forcefully inserted pipe in baby mouth when baby was sleeping and playing. I think my baby died due to forceful insertion of pipe.

<Doctor told the reason for inserting pipe>they said baby has problem in breathing. We know that our baby did not have breathing problems, our baby was playing well, was looking with eyes open, was moving his feet and was also crying, he cried on during nebulization. Now you tell me that when the child is crying, how he can have breathing problem.

Death and post death period

6 Could you please tell us about the death and death declaration of your child?

Mother- they told us nothing. When the baby died, he started moving his hands to remove his pipe, he was hungry, we asked doctor baby is hungry remove his pipe so that I can feed him, then doctor said you cannot feed the child, we are giving glucose, they give 2-2 bottles of water, tell me in fever no water is to be given.

Maternal grandmother- we didnt know what to do when baby was handed over in lap, we should cry or what to do, we buried him, who would support a poor person.

<Who told you about death>when I saw my baby and checked him, I saw his hands and feet are cold and his stomach is warm, and when I touched his chest he was not breathing. Then I rushed to doctor and called the doctor and said my child is not breathing. Then doctor came and said your baby is no more. I don't know anything, they said your baby is not breathing. The doctor who came told about the death of my baby.

7 What was your and your family member reaction to the death of your child?

Mother- I was not aware what was going on. His father showed some courage but I was not aware what was happening. Even his grandfather was not aware what was happening. My mother was not present at that time.

8 How was your experience about the procedure after death till handover of the body to you?

Mother- he was handed over to us within 10 minutes, and returned immediately. Like he died at 10 o'clock and at 10.30 we had left the hospital. His father was made to sign on one paper and said we will give paper to you after one month. Child was wrapped in blue cloth when he was handed to us.

Maternal grandmother- baby was handed to us immediately, when my husband died in evening he was handed to us next morning. Child was handed to us within 10 minutes.

9 Who all from your family and community/neighbourhood were present at the hospital and how they supported you during this period?

Mother- my husband, my father-in-law was with me at hospital.

Maternal grandmother- my sister-in-law visited hospital when child was normal. Then I called and ask how the child is, my sister-in-law said that there is no need to worry now, the child is safe, the child is now normal there is no fear. She said me to visit hospital in morning at 4, I was waiting for my sister-in-law when child died.

Maternal grandmother- Baby was well, my daughter and sister-in-law took baby to hospital. Child father and grandfather came later. My sister-in-law was saying that there is no need to panic, child is being nebulized in evening. My sister-in-law returned at 11 in the night from hospital.

10 What all rituals and procedures did you follow after death of your child?

Mother crying

We brought the child home from the hospital. Child was bathed at home after that he was taken for burial. Mulana bathed the child, like there is pandit in Hindus, he is big person in our community, he performed all the rituals from bathing to burial.

<Who accompanied till burial place> A lot of people had gone, all the neighbors, men had gone, the ladies had not gone, the ladies did not go in us. I watched the baby at home only. Nothing was done afterwards as he was small child, one day morning was observed.

11 How did you, your spouse (wife/husband) and immediate family members cope with the difficult phase?

Mother- One day in dreams I saw that the child is playing in my lap, my mother brought baby to me and I was playing with child very well. This dream came after 3-4 days of child death. The child was healthy as he used to drink my milk, no one could say he was three months old.

Maternal grandmother- i am vegetable vendor, one day I saw in my dreams that baby is playing with me in my lap, I am cuddling with him. The child is happy and playing with me.

12 Is/was there any change in the interpersonal relationships between you and your spouse and other family members after the event?

Mother- my husband and me supported eachother.

Mother crying

Maternal grandmother-After child death her in-laws fight with my daughter, her husband and her father-in-law were ready to leave my daughter, her husband was ready to give her divorce, this all happened after child death, they blamed my daughter that why you took child to S hospital.

Mother- they were saying why you took child to S Hospital, they said you shuld have called us, i called him (child grandfather) he said to take child to private hospital, this would have not happen. my husband was at his home, i took the child to hospital with my relative. my child became healthy and even he was playing after nebulization.

<What is the situation now> mother-now the things the alright. They were not saying anything their neighbors were putting some things in their mind, then person mind will divert to those things. At that time he was misguided by his neighbors, he was right before. Now all things are right.

13 How did the family members support you and your wife during the difficult phase?

Right now my mother and family members understand me and support me.

14 What were the reactions/ counselling and suggestions given by the relatives/neighbours/community after the episode?

Mother- I read namaz (holy book).

Maternal grandmother- Whose child is gone, the attention remains in it. Like I am maternal grandmother, when we still see the picture of the child, we start crying, the child's attention is not getting off anyone.

15 Do you think and/or discuss with your spouse/ family member(s) about the death and related events and blame someone/something or feel guilty for the same?

i blame the doctor who checked my baby and inserted the pipe, his fault is there. and 2nd i blame who gave glucose to child, i dont know whether she was learning or something as in this hospital some come to learn things, i think she was nurse. one doctor came and checked the baby, and 2nd one came and said the baby is no more.

16 When did father and mother (if working) returned to work after the death of the child?

Mother- I stay at home and do not work.

Maternal grandmother- I earn, I care for child. I sell vegetables. My daughter's husband started going to work two months after the child's death, first he was staying at home and mourned for baby death. We too have been in mourning because of baby. I still cry thinking of the baby. When the child dies, who was brought so hard, when he was born he was in hospital for 12 days, my daughter had 6 bottles of blood then, she got her treatment in G hospital. The doctor there was also shock to see 6 kg child.

Other talk

Mother- my child was born in 9 months, there was no problem in child, all investigations were done at G hospital. I became anaemic at the time of having a child and had swelling on my body. The doctor checked the baby with all the machines he was fine. After delivering baby 6 bottle blood transfusion was done.
Maternal grandmother- one doctor was very nice in nursery, god bless her always, one day i started crying she said dont worry if you cant arrange blood from anywhere then i would help you to get from the hospital, 2-3 bottle blood was arranged from outside. when that doctor used to come then she would play and talk with my child.
Mother- i was patient to doctor in G hospital, she was very nice doctor. She used to see that if I was not feeling good then she used to say that what happened, if you have anything tell me, I used to say that nothing just I am not able to go home that's why I am upset. I got discharged after 12 days.

17 How your and your family's life has changed after the death of the child?

Mother- Ever since the child is gone, I have become more attached to my baby girl. Earlier both of my sisters used to take care of my baby girl and I used to handle baby as he was a small boy. My one sister is in sixth class and other is in eighth class. Both of them after returning from school used to take care of her. Love was not less for the first child, but as he was small, so we started loving baby more. My daughter was handled by her grandmother and sister, when I stayed for 12 days in the hospital, then I would miss her, now my baby girl is going to be two years old.

Knowing cause of death in detail- Autopsy and MITS

18 What was the cause of death of your child and what is your perception about the value of knowing the cause?

Mother- I want to know the cause of death, I need to know what caused our child to die. We want to know why our baby died.
<Next pregnancy> yes It is also necessary for the future that no such thing happen in the next child. If my child was alive then we would take no chance and do not have to think about the other child.
Maternal grandmother- If a child is poor or anything else, it should not happen. This should not happen to anyone as it has happened with us. Right now not thinking that there should be a second child, during this pregnancy then there is a nurse in GTB, she was very happy, bidding my baby girl that now you have two children, it is enough and she was very happy and prayed for her, the nurse brought bread from her house, she was not a nurse, she was a doctor (Mother), the same doctor said that my girl is short of blood, and the next child be a threat to your health.

19 In your view, what more could have been done to know the exact cause of death and the reasons causing the disease?

Mother- You will find out by having a test, but now nothing can happen, what was to be happened has happen. Many children die in S Hospital, many children were finished in front of us.

20 Are you aware of any method for identifying the exact cause of death?

Maternal grandmother- You people will know the reason for death, by which reason our baby have died, we are illiterate. If I will not earn anything, how will I eat it, and how will I pay rent. Only you guys can tell how death happened. It is here that we cannot do anything but we can appreciate you, when you tell then we can come where you say.

21 What are your views about the autopsy/ post-mortem for identifying the cause of death?

Maternal grandmother- The child cannot have a post mortem, anyway he has been buried into the soil. I have no idea why the post mortem happens.
Mother-I know that post mortem happens when someone is killed by hanging, if someone consumes poison then post mortem take place. Post mortem of child cannot take place. It is not allowed in our religion also. The person who has an accident has a post mortem in the hospital itself. If his heart is alive, so doctors take out all organs by doing post mortem.

22 What are your views about collection of the tissue (like done for biopsy to diagnose diseases) and body fluid samples for identifying the cause of death?

Mother- This technique is good, there is no tearing in it, people of our religion will agree for this, there is nothing like it.

23 How parents and family members can be approached for the tissue and fluid sampling?

Maternal grandmother- Nobody told about this technique in the hospital, I wouldn't have agree to perform this, as for now baby has gone, it cannot come back.

Mother- Like when the doctor told us about the death, then he should have told about this technique, all this should be told by nurse and the doctor.
<Whom should they tell> if I was not in that state, then this technique could be explained to child father or to my father-in-law. At that time child father was alright, doctor would have told to him about the procedure which can tell us about the cause of death of our child.
<How should they tell> doctor would have told this verbally.
24 In your view, what factors are important for acceptance of the tissue sampling by the parents and family members?
Mother- no one told us the about the cause of death at that time, they only told the child brain nerve has burst, there is bleeding it first started from mouth and then from the nose. we should know the reason at that time only, when our child was normal, how did this happen.
Decision making dynamics
25 In routine day to day operation who takes decision about the major activities (purchases, treatment, spending money) about the family?
Mother- For routine activity decision is taken by my husband, for any treatment decision is taken by my father-in-law. My husband does what his father says. All the decision in our house is in the hands of our father-in-law.
26 During the hospital stay who discussed with the doctors/ nurses to make the decision(s) about treatment and other procedures.
Mother—Nothing was told to us in the hospital, so what decision we take, we were just sent from one ward to the other ward from the emergency, and they did not tell anything in the whole night. We were just pumping bag and tube all night. They were not saying anything when we were asking.
Maternal grandmother- When my sister-in-law came in the night, she was saying that the child is fine, no need to fear.
27 After death of your child, who decided about the cremation and other post-death activities and rituals?
Mother- All decision were taken by my father-in-law, he only gave money for shroud, during burial. When the child dies, it costs less money and the elder cost more.
28 Now, we are completing the interaction/interview. Would you like to add anything?
mother- We tell you that in S Hospital, there is too much carelessness, G Hospital is still fine even child dies there, it is also a government hospital. Doctors do not let go deliberately, S Hospital is too dirty, no toilet is right and no water comes, nothing happens there. No facility of bathroom and toilet is there which is very troublesome. Where we were sent there was no water in the old building, we were kept in ward 18. In toilet there is no supply of water, even huggees are lying in toilet. The guards do not let anyone in. The guards disturb everyone. For us two person were allowed due to bag and tube, even if we had to ask something from doctor, the guard would show up, not allow us to enter. There should be place for the attendant in the hospital. All government hospitals are like this only, they intentionally mess up. One thing I must say that the doctor in hospital does not pay attention properly, so all this happens.

In-depth Interview- Parents of Deceased Child 4

**1.1**	**Primary respondent (relation with the child)**	Mother
**1.2**	**Age of the primary respondent**	Mother-25-30 years<br>Father-25-30 years
**1.3**	**Other members present**	None

Context- Her child was admitted in ICU at S Hospital, the total stay of her baby was 25 days before that baby was getting treated at K hospital.

2. Events that led to death of the child

2. Can you please let us know about the illness and events that led to death of your child?

When she was born, she was very weak, had jaundice. The delivery was done 10 days after the scheduled time. Then after that he had jaundice. She was kept in her S Hospital for a week, then she became healthy. The doctor said that the child is very weak.

Then it was good for two to three months, it was thick. But he started getting fever, I used to show him to the ordinary doctor, one day a very high fever came (102) and he was not getting well. The doctor said, put a bandage on the head. I put the bandage on. His chest became jammed, with it running cooler overnight. I was alone and the other child had to leave school. It was raining outside, sometimes I used to get wet in the rain, we had to feed milk. Due to crying, we had to be fed immediately. The child was taken to K Hospital due to excessive cold. Was admitted there for 15 days. His money was off. There was a pipe for urine. When he pulled out the pipe, blood was coming out. I got angry after seeing this. The child was screaming and the doctors were not paying attention. In this way my baby girl will die. My son was watching all this. His mind was getting worse. After the debate, he said in K Hospital that you take the child. I said stop now, it has urinated, it will be fine when I give it milk. But the doctor there does not have a treatment method. If we had money, we would not come here, go to a private hospital. As soon as he got out the oxygen pipe, he started having trouble breathing.

After that, the child was taken to S Hospital. Admitted to ICU there. Admitted there for 25 days. There was swelling in his body day by day. The girl died 25 days later.

Was admit there, then day after day, day after day, it was swollen, it was swollen and then it was over at around 12 in the night, did not tell what is the disease, it was said that his chest is not this lung. It is done, and brings this medicine, bring that medicine, do this, do that, after 25 days of admission, it is over.

3. Hospitalization period

3. Now we would request you to tell us something about the period of hospitalization for your child?

<Duration of stay>

15 days in K Hospital and here (S Hospital) admit in 25 days

<About HCP of S Hospital >

All was right, I did not go and forcefully took me away from time to time to make milk, the nurses give milk to them

<course of illness>

When I first went, put the girl in the emergency ward. He said that the bed is not empty in the ICU, then the doctor of emergency ward phoned the doctor with ICU and asked if it would be empty in the morning, these are small children. There was some pipe and bag in his mouth. Pump it up, he had to press only that night in the emergency, then my hands started hurting all night, one night I did not sleep there (kalawati) for 15 days, then my father arrived at 5 o'clock in the morning, then he pressed for a while. Then it got accepted (ICU) at around 8-9 pm, so we were thrown out, there was no loss in his body, so how to deliver medicine to him, he was bald, dipped in his head In my mind, I used to give glucose or medicine to him, then after two days, after applying such swollen medicines, I asked the doctor how Madam it has swollen, so he said that you are looking swollen, I said I have a baby girl, I do not know, she was such a bone, if she was swollen, she would have done something, she did not say anything, she has come here to get treatment, not to answer the question, she said that and

then after that The doctor who did not come in the round, in the morning there was a lady doctor in him, he had also said this that he is telling me that we are trying to save him, if the son will be right then it is a good thing or not We are trying our best, so those people who used to speak and who used to keep it during the day, the doctors used to decide how much milk to give from that medicine, and the reports that were made during the day were filed. I used to check the paper which I kept, then I had to feed him after two hours every day, then later his stool stopped, after 20 days he was completely fine. He was a doctor again one day, Who speaks the language, he was not an Indian, he was black, long, good, very normal, talking like ordinary people do, so he called me that now it is difficult to save, I said what you will tell, I am his mother. I can feel every beat of him, I touch him, then it seems to be over, his eye did not close like these 20 days later, when I opened his eye like this, it looks like water starts coming out of his eye. Father, where he works in the Kothi, he has a domestic doctor, he had taken it to him earlier, he meant that some specialist of the child was such a doctor, he once in private, he once meant money from the Kothi. To give my child a treatment, he wrote that S Hospital said, "Take it there, there are good nurses, there will be oxygen. You will not be able to treat it here, so we took it there when they started removing water from their eyes." I understood that its entire body is filled with water, now nothing can happen, then they explained to me, I thought that what was to happen is in the hands of the above, now what do they know about it? Doctor) I do not even understand Hindi as much as the people who used to talk in English, who used to explain to me, I used to understand that, they used to tell me those people. Once you said about my baby, you give your milk or you feed it on the top, I said that if there was no milk, then I mostly gave it the top milk, which one is it, then I said whatever This 21-rupee packet does not come with a pouch, it used to drink, I used to drink it, it was suppressed, the girl became very good to me, then after that you said, bring milk from the dairy.
I did not say, I will not get it from dairy, I have spent so much money, I will bring a packet of 4-5 hundred rupees, I will give it by heating water, I do not know how the milk will be, so if I can buy it then why I did not say Will I be able to buy, can I not buy medicine here, I do not buy, I buy it every day, then they gave it in writing, then it started feeding, then it was good, at that time I was thinking, my baby is well , Two days later I do not know what happened Madam, she did not realize it, after removing it from here, she again injected it, then she did not feel conscious, meaning there was a needle here, there was a needle With, soldering that one, then it was applied in this thigh, if it was not applied in this thigh, the blood was not going to stop, even after it was finished, its blood was not stopped, which is applied by the solder. The needle was made of plastic, then it was finished at 12 o'clock at night. Then 4-5 days he (doctor) did not call me, he used to call only for medicines, then stopped his milk, he stopped urinating, latrine too. The same people used to see a doctor for 24 hours, then other doctors used to go inside the ICU, that is, they call time after time, like those people call after half an hour after an hour, which means that those people are negligent in the day. Did not, now at night we cannot say, now there were two doctors who used to treat for 24 hours, that means they used to stay in front even if I went out, sometimes I used to go for feeding, then the nurses did not come forward to everyone. My child used to come here after feeding me. My child had 14 number beds. So it was the last of all, then it used to take time to make milk and these people used to go to talk on the phone too, two or three doctors were very good, they used to say, come here to nurse and sit in two minutes. . There were two madams, both were Priyanka. A South Indian was a doctor, he was good, he was also very good, and he was very good, and he was like a rage, if we have children, he is in a mess, then we will make it clear, he spoke to you or something like that in some English, we do not understand anything, do not do that. Now if there is dirt, then we will clear it, otherwise the infection will spread, if we change the sheet one day in the morning time, the nurse is saying that it is clean to the children. A packet does not come to clean the potty of the children who are from the throat, I used to clean their body every day because they used to remove blood, neither did the blood sit anywhere in the body, so it was said that if you keep it clean Would be so nice Most used to call me because I was a small child, saying that you should come and try yourself in an hour or two, I was allowed to go inside, the youngest was the same, so the guards did not stop me. I used to look at him, meaning someone is a nurse, he used to say a little harsh, so one day a doctor came, he was also on the round, I was inside him, which meant that the doctors who came round had come. I said that I was doing it very clearly, in such a long time, those people were 4-5 doctors used to come together, these people were also together, they used to talk in English, so I am telling my son your baby girl That

pneumonia has worsened, we got it done several tests, that means x-rays were getting worse day by day, then showed me that it was a board like a TV, it was switched on and it turned white and then the TV became white again. After that, I put this X-ray again and showed it to me that I did not understand anything or else said, look, it has started to be white, when it was first done, it was so much, after that it happened at least 8-10 X- Ray got it done, did not explain it on the same day, when it was over, the girl was alive, explained that day, and the same night the girl was finished, the same doctor came that day was very good, explained to me, he is saying son Look we tried Do not say that it is the doctor's fault. Now we are not so educated, what did those people talk about in English, we used to just stare, then those people used to tell medicine, then I do not know what they used to say in English, they used to tell me that you should not stop going, medicine. We used to give expensive medicines from there and then we used to buy them from outside, outside the main road there are all those medicines which were used to buy medicines from there. <about cost> At that time, I did not take that much attention, as they kept writing medicines, writing the goods, buying them, it was more important to save that time, one was upset that here it had become typhoid (live child). , I was worried about taking two children, I had no one here, I had left the child near the neighbour, after seeing these people, my father-in-law came, so for 15 days, he was ill, I could come here, there Was not able to live either, I said it will also go, it will also go, so let the above one, that person has become lovely. After that, it hurt me more, it sat on his mind, he was passing urine from the pipe, neither did he act like crazy, he had seen it, he loved it more than his sister, most of the night. In the same mobile till two and a half hours, both the two sides used to listen to song in the mobile. If I would put sleep there, I was lying on the pillow of the clitoris, both of them (live child) still know that its sister S Hospital is with the doctor, I did not say that the doctor is a doctor, so I am happy that my sister is there If we say that she has gone to the one above, then we say no or she is in the hospital. He is 6 years old, so later the doctor did not speak it and did not sit on his mind, he did not take the child to the hospital, he was crying a lot to see his sister, the doctor said yes Show it for 10 minutes at the same time that the scandal took place, playing before that, both of them were playing a lot in mobile.

4. Could you please describe your overall experience about the hospital care?

<About treatment>

I liked the way they talked. You cannot know the method of their treatment. Often, check slips were given to me. People who did not understand, used to explain. Where you could not go like OPD then send it with someone there.

5. Could you please describe your experience about communication by the various hospital staffs during hospital stay?

<About HCP>

Doctors used to explain in Hindi, they understood it. But he told us directly. That the child's pneumonia has deteriorated, otherwise we will do our best and it is God's will. We already knew that is pneumonia. The heartbeat started growing at his home. Foam started coming from his mouth, he was very much. Previously she was taken to the private doctor. Bola is pneumonia. Now double pneumonia is done. Take it to the hospital soon, then I took it there. At K Hospital But the treatment at K Hospital is not as good as that of S Hospital.

<About communication of HCP>

I used to cry day and night. Nobody used to bother me that much. Spoke directly. I used to live alone. One day the doctor told me that no one is with you, so I said that my husband goes to work during the day and here at night. After all, money is also necessary. There are so many expensive medicines here. If no one does the job, the treatment will be from where? My little child used to do small things in the hospital during the day. Like bringing test report from another building, bringing some similar purchases during the day. Right now my boy is very small. When there was some urgent work, the husband used to take leave from the job.

<Primary communicator>

Nobody used to tell me clearly. Every day I used to ask them how my child is today, whether to feed him or not. He used to say less when asked about everything. He was a doctor and he used to talk a little bit and everything was fine.

<Skills of communicator>

There were 3-4 doctors who liked the best. A doctor was a bit mean, it was not bad in the heart, none of the meaning was like they do not put pipes and say what they say inside the mouth, do it in English,

when the foam increases. He used to do it in his mouth, as if he was late for two minutes, the nurse used to come and shout, the doctors were saying "Do this work first", they used to speak in Hindi, otherwise I don't know if there was any patient. Some who used to get exhausted, a lot of men came and abused them. I have seen how many corpses I have seen in 25 days, I do not know there, I used to take out one or two every day, I mean I have not been there every single day, I have not seen a single day coming out of ICU, in three days in 25 days Since then I also felt in my heart that what these people used to explain to us, they used to do something else, they were unable to understand. Even a patient should come right out of it from the ICU, no one came, there was a girl, what she had come to speak, after four days from UP she was in my number 13 and my daughter in number 14, So after 3-4 days, he was transferred to OPD, after a week, he was brought back to the same bed again. He was negligent in OPD. I mean, the doctor brought 4-5 operations to his ICU, then put the pipe in it, the doctor came from outside, to do the operation, one has to clean it with water, which used to wash the kidneys, then a doctor always chairs But he used to sit near the patient, meaning the child's doctor was negligent, he was a little lacking, whenever he used to go he meant duty, he used to sit in a chair and saw how much water was filled under the measurement, how much was left. Used to change immediately.

Death and post death period

6. Could you please tell us about the death and death declaration of your child?

No one came to tell me that my heart was very nervous at 12 o'clock in the night, I was sleeping, I went and asked the nurse to feed the child, he said that the baby has to be stopped. His toilets and potty were already 2-3 closed. One day the condition of the child had deteriorated, there was a lot of water in his eye. It seemed that now my child will not be left. I started crying. I thought if there is luck, then the child will be cured or else God will snatch it. People explained to me, said, "Go out and come and eat something, or if your health is bad, you will see the child." I sat outside for an hour, then went inside, at night when I was feeling very nervous, became sweaty, sweaty, I used to walk so fast AC in ICU, we used to sleep two or three blankets. , I was completely wet in sweat, then stood up, then I come to see once again, how is it that when I went to the girl, I could not see the machine directly, one day if I had explained it to me in Kalavati It will be above 90, neither its heartbeat will be right, if it starts coming down from 90 to 70, 80,60, then it was my attention to call us immediately, then I did not see my daughter, I see above, I am machine locked. Then I paid attention to the bed, then I see that her (dead child) dam is kept blind, so if I went inside, I was not going inside any more, then I asked the doctor, doctor sir, like I told the doctor, till then he started speaking, his father is there, send the father, you go out, I picked up his father. I did not say right I have come to know, what is the matter, tell me, why are you so nervous, I have not said - do not go, the doctors are calling, then after seeing it they finished it. He (husband) was a doctor thinly, who used to do duty during the day, that was also good. Doctor means three men were doctors and two lady doctors were cheated, five meant that they used to see all the day, so as its father out. When he came, he said to me, "You sit here, I am praying from outside, I started to fire after them, now at that time no one was able to stop me."

7. What was your and your family member reaction to the death of your child?

I was crying a lot, my father was also crying, then he called his relatives, all four of them are on fire, this is the same, when they came to the hospital, I was looking at them and was crying a lot, so its Father said, take it home, I myself forbade him, everyone was speaking, take it home, its grandma, grandpa is at home, everyone will see the child, my son will one day see if he is sick yet, then he will see in such condition No, what will happen then it is gone. The second thing is, I live in a rented house, I know why the tenant has brought here, where he was so ill, he had brought the infection, spread the dirt, then my elder brother, Tau, uncle's first elder. Then there were uncles, she was saying that she is very right, and it was a bit strange that she was so weird, so I said don't take it, then she said that she presses the little child, so she asked the doctor So the doctor said, we do not know whether the people who are driving outside ask them with the ambulance, they will know them, then they asked if they asked outside, and told a place, Kalindi Kunj had told something like this. Take the same thing, bury the child there, first we used to stop here, then we stopped at Rohini and then I called the father-in-law here at night and told the neighbor. Then we live here very well, in whose time these days, who is there in 15-20 days, I put the child in their hands and got these people treated with their money (), their parents are not there Later, he came and gave me the breakfast of the morning, the

child used to send the lunch, the woman who sent it to school, I used to take care of it, I was carefree, I could even call my jewelry from my hand and call it Therefore, we do not have such a good house, we pay more rent, at 12 o'clock at one o'clock if there is any problem, then we give it to each other, that is, there is 8-10 women working here and there. Reached me, at 12 o'clock my daughter was finished, at one o'clock these people reached me at night, then we went to the same place with me, they all took me too, so I wanted to see, show my children a little, So the doctor had refused to do it earlier, its (dead baby) was not grossly spoiled, do not open it, the doctor had tied it, its (dead baby) used to remove a lot of dirt from the nose, a lot of dirt from the mouth, do not touch your hands, infection will spread, you Have more children.

<Cause of death explained>

Anything told, it ended, at 12 o'clock, it was over, and it was told to us that the pneumonia has worsened it, the lungs have worsened, but what was the reason for it, nothing was told Madam.

8. How was your experience about the procedure after death till handover of the body to you?

<About documentation>

After the death, the father of the girl gave the discharge paper, he gave it to me, what use was it when the girl died. Then on the same day the day my daughter died, we would eat and drink food during the day, in the evening we were sitting outside in the hall which was made like hall. Whenever he used to call, he used to go inside, there was a girl from ICU, went to college, his mother worked in the brick bhatti, her husband lived in the village. She used to talk to us. My daughter has become like this. He has undergone complete surgery from here to here, he is not wearing anything. It is kept just like this, I go at night, I am scared in myself, to see my child. God says my child is right, if it is not right, if it ends, then if something happens, I will give a lot of bad to the doctor here, I cast a lot and shift my survivors, I hope so, on the same day At 4 o'clock in the evening, her child died, her mother gave such a badge, she was crying even more to the doctor and to the guards. She was going to faint again and again. Said that these people killed my baby girl. For this kind of disease, the whole child tore my child, from here to here, saying, I do not know if the infection has failed, I do not understand anything, then another death occurred on the same day, he also spoke very bad. We have not even spoken as much as it means to do anything. As soon as we asked questions, lied only, but only understood us, we understood, thought the doctor would be speaking right, the doctor is in the form of God, when everyone speaks outside, when someone goes inside the ICU, Goes to die nowadays no right hock returned. Meaning that other people who live outside, they say that we have been living for 6 months, some say that we have been living for 3 months, some say that we have been living for 2 months. I did not talk to anyone as long as we were there for 25 days, when I was crying a lot, neither many people gathered at night, then everyone started speaking. Everyone used to sleep on the bed outside.

9. Who all from your family and community/neighbourhood were present at the hospital and how they supported you during this period?

<Who all supported you>

His father called his mother, he called my father-in-law, then the father-in-law told his mother-in-law, at that time my son was very ill, then the father-in-law had not gone. If you go, then you will get the bath, then I said that the bath will be in your house or not, if you go to take a bath inside the bathroom, then everyone will say why are you bathing a child with infection, then I did not bathe, went direct from the hospital.

<Who all supported you>

All the people were there, their father was crying a lot, then Tau, uncle were those people.

Post death procedure and coping mechanism

10. What all rituals and procedures did you follow after death of your child?

<Number of people was going to burial place>

I, its father, its grandmother, uncle, two aunts, 10-15 people who come to work at night, can do it at night, we can do it at night also, and there is no prohibition.

<Timing of burial rituals>

These people came to the hospital at one o'clock, then we sat there for a while, saying, stop now, you are going from there asking this car, you are asking for more money, then it took an hour to find the

car, it was two o'clock then there Everyone sat in a car and then we came, meaning those who went from here, some people went in the car with the corpse.
<About neighbour> those people were in their own car, so it was three o'clock on the go. Then while talking there while we were trying to be in the mosque and buried it was also dark around 4-5 o'clock.
<Who performed burial rituals> its father only did everything.

11. How did you, your spouse (wife/husband) and immediate family members cope with the difficult phase?

I was faint at the time when these people went to bury him. For a month and a half, I was not getting food and drink properly, I was a heart patient myself. I felt at that time that I would die. I could not see anything, everything seemed like a dream. I was dreaming that I am playing with my child, like in the house, I opened my eyes, I fell asleep crying, a woman says, I empty her from here and go away, that means her on the shoulder She was sleeping with her head still in her arms
I opened my eyes once again and I started crying my daughter then, I see these people coming from the forest, I was scared to see them, they had taken two rooms, one was in her circle, one was that if there was a room, he emptied it. I was crying a lot there. I came home for a month and a half I was right, after that it seemed as if she (dead child) came and drank milk.
<When after death>
After a month and a half, she was born, since then my period had not come, on the day she was 13, my health was very bad on that day. Had 103 fever. Four days ago, I had so much fever, 103 did not come out, I had the test said that typhoid is there, typhoid medicine used to say that I used to have more fever. 102 Fever used to be like I used to sleep or did not feel like my baby. I am giving milk, the bed gets wet, I do not know where the milk was coming from at that time, when it was alive it was not milk, I used to get the bed wet, so my mother-in-law said, in the custom is to put milk in the bowl and mother would do it, I used to do it when the cat came and drank milk, then my son felt that the sister had come and drank milk, so this (live baby) night When my health got worse, then one day he was sleeping, he started crying while sleeping, his hair started scratching, the head started banging on the wall, he said, "Why did my baby leave, why did you put money in the piggy bank?" When the Raksha Bandhan comes, my sister will tie a rakhi in my hand to bring the doctor an hour of speaking, because I used to call her (neighbour) girl every year and get her tied, it was very nice to get her sister tied, Now some child said that your sister has died, someone has gone inside the soil and someone has told her in my mind, I did not ask my son, when he was ill, he used to say that the sister will come to the hospital. Now later the doctor said not to keep this in his mind, to tell him slowly, otherwise he will remain in his mind, but now we speak, he does not believe it, he still knows his sister in the hospital is.
<What rituals performed after the burial>
They do not worship. But washing clothes, temples, pooja lessons did all this and then after a month and a half I became very ill. People used to come to see, then they said that the daughter also went, now the mother will also go, so in our Calcutta there is a lot of sorcery, then people used to say, do you know if it will be possessed by a ghost, the mother is after the child is over Children do not find the father, they seek the mother only, what address does the soul of her (dead baby) bother her. Send it to the village, so when I got blood test done again, the typhoid came out, the doctor said that its typhoid has worsened, pneumonia has happened, at that time I did not even have much money, I spent my money in his (dead baby) affair. It is done, debts were also incurred at that time, so then (her) owner gave my ticket. The owner is good, sent the ticket to the village. I went there and got medicine, medicine is also of no use to me. I mean, Baba called me, he means I have perished and said that he has settled in his blood, who is dead inside the hospital, then he has to come out, then asked me you see something, I said I get strange sounds. Hai, I see a woman, wearing a black sari everyday and doing havan from the front and wearing a big dot. Big hair used to look at him, close his eyes, be full of his eyes, he would look gross, so Baba said, yes, its evil thing has entered its body. They dusted me in the morning, they say dust in the evening, till then the people live, they are still alive, when it is finished, it becomes the devil means we believe it, so like Madame dust me twice in the morning, in the evening, like in the evening My body did not stop sweating for three days and I fell ill on the 5th, my ticket was on the next 5th, went home, reached the 6th, my treatment started on the 7th, it is the month of October, I have just come on the 5th, then took them to the doctor, then he said that if he is sweating, his body is getting cold, then do not give medicine for fever, he becomes a person and

becomes ill, cold is one and fever If you give medicine then it will get cold, do not give antibiotic yellow and give medicine for fever, if fever comes, then I give puja recitation, we get it done in us, nor can we go to the temple until it is worshiped, worship Cannot do, I cannot feel festival. I made him a great pundit, he knows a lot, then he said that if you will take a child within this year, you will be left or neither will that child bother you again and again, the body will ruin it, So you have to do a lot, then we did all that, and the child looks at me, ma'am, still looks like I am giving him milk, so he said that he tries to come here, so he has to stop now 2018 If this is not good then I saw her (dead child) horoscope, when she was born, we told her that it is like this, so my horoscope is mixed saying that she is trying to come again, so she has to stop this year after January. Meaning 2019 will take you in it.

12. Is/was there any change in the interpersonal relationships between you and your spouse and other family members after the event?

There is no such change, they just take care of me, I cry to them, as they (husband) at night, when I open my eyes, I see that they are not asleep because I have just given birth to both children, but care for them (both baby), I didn't know anything. When I got married at a very young age, it was the cleaning of the latrine, the feeding by her husband, then she taught me, then later on, when I started taking care of them, so they have a little more.

<Relation and attachment with other children>

Yes, more attention is paid to the other child, but it still needs a sister, then I will talk to the doctor if they will say what they said otherwise, if they want to take another after 2 years to defend it. You cannot take it now it has not been even a year.

13. How did the family members support you and your wife during the difficult phase?

My mother and father have done the most to me, be it with money, whether through treatment. Had my mother been here a few more days, my health would have deteriorated (mother's health). Everyone said that admit it in S Hospital. My children died. I saw that there is a useless treatment, if any good happens, it will become ill. Some do not pay attention. They give injections anywhere. The blood keeps coming out. I am very scared to see all that. Now I will die but I will not go to that hospital.

14. What were the reactions/ counselling and suggestions given by relatives/neighbours/ community after the episode?

15. Do you think and/or discuss with your spouse/ family member(s) about the death and related events and blame someone/something or feel guilty for the same?

I think the same thing happened in Kalavati because 36 times a day he used to extract blood from the needle in his body. Those people did not mean much. Like when to feed the baby etc. There is a separate machine for the person who is Aamir, different for the poor. The poor one goes bad. A child has pneumonia. When the AC is running, it gets colder, then cold milk will go in and it will cool, now I did not have my baby with me, I was not getting salary, I will not lie given the same milk, my father works , Which was taken to me for 15 days, so I made me believe that my father used to go to get 1000 rupees of his daily, he used to go near it (live child), 250 rupees auto, then from here I used to go to 250 rupees auto in the morning, then from there they used to come to Malviyanagar and Kalavati for 250 rupees autos, then they used to go to reach me at 9 o'clock at night, meaning poor had to go to four places four times a day. Twice I have duty once here.

16. When did father and mother (if working) returned to work after the death of the child?

The owner of the company himself said that I had kept another driver, as long as your child is treated, then you take care of him. The Japanese company said it was not going to cut your money, it is good means a foreigner, but it is good, then I finished the whole month, gave it to my father for 20-25 days. Used to call for hours, then they used to ask the doctor that there is no work, because my work was outside. These people used to get me to go outside to get a report and go away, some work was done, the outside work was done by the man and the inside work was done by the woman. His father went to duty after finishing two or three, which means it has been four days, and after that he had gone for five days.

17. How you and your family's life have changed after the death of the child?

18. What was the cause of death of your child and what is your perception about the value of knowing the cause?
I do not understand if the girl died due to doctor's negligence or because of my negligence. Sometimes it seems that the doctor did some reverse medicine. Then, it seems that if the medicine were upside down, many people would have a problem, but yes! There is a lot of carelessness in the treatment that is done in OPD. One girl was very healthy. She had gone in front of me, said mother, I will go home and have food. She went there for three to three days, it made the situation worse, the doctor also makes mistakes, but where do these people (doctors) believe. Now I cannot enter the treatment, how was the treatment done. Now that she has left the world, what reason should I say? You have to ask your father only, he will take the decision.
19. In your view, what more could have been done to know the exact cause of death and the reasons causing the disease?
No I don’t know anything
20. Are you aware of any method for identifying the exact cause of death?
They all say, they check, what is not there, then they find out whether it was caused by the doctor's medicine, if someone dies like this, they kill, then it is known in how much time Yes, a woman told me there, she used to work there, there is a post-mortem, those big men, they are disgusted to clean up the potty people, then people came If one day she was eating food, then she used to shift to go daily, tell someone else if needed, then share the money in each other, so I said, come and eat the food. What have I brought, I have brought it from the hotel, I have to eat, I want to eat it, I have brought Bachura Aajao Chhola, so my husband was saying my husband is over, I will not burn him, I will get him posted and someone else will say that in the hospital. She came, was saying, the doctor killed my man a lot. He ate food at night, the one in the front, the one in the front, the one in the side, see it is over at 4 in the morning. I do not know if I have given any medicine or Gave needle, bleed from nose you done, speaking like this, then the aunt was telling the post-mortem that this thing happens if the medicine is over, neither will it be known that the doctor gave the medicine wrong.
21. What are your views about the autopsy/ post-mortem for identifying the cause of death?
I would have thought of having my baby girl. Now if the girl is there or not, then what is the benefit of the post mortem, if there is a death in the hospital, then it must be done once, it will be known that it is your fault or the doctor's negligence. Must be done once.
<Religious aspect>
No our religion is not forbidden.
<Disfigurement>
we have heard that people speak, take out kidneys, do not know what they do, they tear it out, they tear it all, doctors all sell it, now what to tell All is true that some people say lies, some people speak, doctors take all these things and those who are short, they want to stay alive.
22. What are your views about collection of the tissue (like done for biopsy to diagnose diseases) and body fluid samples for identifying the cause of death?
This is right
23. How parents and family members can be approached for the tissue and fluid sampling?
<Who should approach>
Like you - people have come to ask that if someone like this can go to them and ask something, the doctor should tell himself the size that you do this thing. Some- doctors are good, one had TB disease there, whenever she used to come and not inside, there was no patient, who used to take care of them, then she used to say that you should wear gloves which are not in the mouth. If you wear what you wear on your feet, then your germ will go into someone else, someone else's germ will come in you, the doctors used to explain it, the doctors did not explain who were feeding, the men were weaning and There were also nurses, these people used to say all these things, the doctor never spoke all these things, he had explained to them, it has been kept here to wear all these things and then go to the patient.
If the doctor himself told me that you should do post mortem or my man, then we will say that yes, I should do it once, as if I am a mother, I would control myself at that time, someone used to say, While speaking there, who will take my man to someone else, I will get him tested. What is the fault of the doctor? If I had this in my mind, I would have said the same at that time. I knew that my child was suffering, he (the doctor) did not leave anything that I doubt on him. They (doctors) already knew that

this would be over, now these people used to pay a lot of attention to the children and there is negligence among the big people, nobody saw three children among the children were children 13,14,15 Only children lived in them, there was very little negligence in them.
Earlier, I did not even know the language here myself, now I understand and speak.

24. In your view, what factors are important for acceptance of the tissue sampling by the parents and family members?

Decision making dynamics

25. In routine day to day operation who takes decision about the major activities (purchases, treatment, spending money) about the family?

My father does everything there, my father lives in the village. If I want to do anything, meaning I want to do any work, then I definitely consult them. <About treatment> This would decide its father

26. During the hospital stay who discussed with the doctors/ nurses to make the decision(s) about treatment and other procedures.

When there was an admission in the hospital, the report that the doctors used to give, which means, mostly I, they were afraid that its father is very timid, my child stayed inside for 25 days, he did not go, and he was very scared. Because there were very terrible patients. One day I was scared myself. I myself have been ill for two months during her affair. Now I did not know, that girl is finished, I went and laid her hand on her bed. I was absolutely scared.

27. After death of your child, who decided about the cremation and other post-death activities and rituals?

My uncle was doing this the child is buried in us. When they went there, they said that they had to buy land for it, then they took 2100 rupees and then buried them there inside the forest. I do not know where my father was buried, then the father was saying that his hands were open, so he meant the soil which he makes by digging the pit, who cares there, he did not ask for anything, those people know Have not said, put money in his hand and then put mud on it, but his father closed his fist with 101 rupees in his hand, then put mud on it, then when he came from there, his father said It had something, it was something of the last birth, they came to do it, put money in his hand, this was told by his father later. Those people do all this day and night, neither the soul is not afraid of the soul, that is, they know a lot by doing something in themselves, because these people only do this work day and night, burning, then they know everything.

In-depth Interview- Parents of Deceased Child 5

**1.1**	**Primary respondent (relation with the child)**	Mother
**1.2**	**Age of the primary respondent**	Mother-25-30 years<br>Father-25-30 years
**1.3**	**Other members present during the interaction**	None

Context- father works in marketing line field work and his age is 30yrs.
Not just this incident, these stresses are there since marriage. I think because of stress and tension the child has got so many problems.

Events that led to death of the child

2. Can you please let us know about the illness and events that led to death of your child?

There was a normal delivery but the child had a lot of problems, he also had cleft lip and cleft palate, we came to know after a month that he also has problems in the heart, when we were taken to S Hospital for the first time the doctor said directly that the operation will be done. We were not sure that there would be a problem in the heart of the child we thought that as we first came to know about cleft lip with ultrasound, the heart problem should also be detected by ultra sound. We brought S Hospital for the same reason that it was born. Still we were not able to believe that its heart really has a problem, but when eco, etc., found out in S Hospital, it had a lot of problems in its heart, the pipe was leakage and the baby was born After that, the vein that stops the natural, that too was not closed. The doctor sent him to the operation in the SSB building in S Hospital. But these people said that there is no operation of small children here. Then we were sent objectives. When the doctors referred to the aims, I asked when the operation should be done then the doctor said that in one to one and a half months. But in the aims, it had been a month of circling. And these people also only spoke to aim, did not give anything in writing. If these people had given anything in writing, or if the operation had happened quickly, then my child would have been cured.

Hospitalization period

3. Now we would request you to tell us something about the period of hospitalization for your child?

When the child was diagnosed with Pneumonia, he was first shown in freezing private, when he could not get rest, the doctor said that you should either go to our place or take us to S Hospital where the child is born. So we also thought that he took the same place where it was born, because it also had a cleft lip problem and the doctors in S Hospital are also good there. We went in the night and went to S Hospital in emergency. Stayed in the hospital for eight days, this was the first time. Then get the eco test done, which had a very long date, 15 days.
We also came to know from this test that the operation will have to be done. We were running here and there for the operation that the child used to get infection of Pneumonia in between. Everybody used to say that operation, operation is the only option. Those people had told that its operation is not done in our place, and those who have heart problems get pneumonia infection very quickly.
We came to the hospital five times in four and a half months, sometimes one, sometimes five days, sometimes ten days lived in the hospital.
<Duration of stay>
Admitted the number 20 in the ward, then we had been there for ten days then discharged and we came home. Two days later, the child had a problem again.
We then assigned the child where she was the first admit, in the ward number 18, the doctors told that there is Pneumonia. We had told the doctor that just two days ago, two discharges have taken the child, then the doctor told that this is only a treatment operation and it will not be here in another hospital, do the operation as soon as possible. This doctor had said, but we did not have enough money to get the child operated out. When we went for the last time, we were in the hospital for five days and on the fifth day my child was finished.

4. Could you please describe your overall experience about the hospital care?

<About treatment>
<About investigation> we used to go ourselves.

<About cost>
Nothing much had happened because he would have had the same infection of Pneumonia, then it would not have cost much but when I lived in the hospital, I used to see that people who used to write tests to people, they used to write from outside also, but our child did not have any tests done from outside.
<Who had supported you in the hospital from your family>
My mother and my husband were with me, whatever decision I made, I would take it myself. I had consulted my husband about the children who apply for breath because I knew that even the winning children had a separate tube for breath, no one was left all the children were dead. My child also felt that the doctor had done a lot of carelessness and did not put it properly the light did not burn properly. In one line, I can say that I was not paying attention properly.
<About HCP>
Behavior we used to talk in a very useless and dirty way, I also told an employee that we should take care of our patient that cleanliness here. He gave me the opposite answer, said that you should spread the dirt and cleanliness.
5. Could you please describe your experience about communication by the various hospital staffs during hospital stay?
<About doctor>
The doctors were all lost in themselves. Not good there. Some doctors were good, but more doctors used to speak very rudely. The nurse used to speak in the same way incorrectly. According to me, a senior doctor should always be there. They used to come to the round. Because if we had reached on Saturday, the medicine would not have started till Monday, because I used to go to the middle of the week and senior doctors do not come at that time. If any senior work was done by senior doctors, everyone had to wait till Monday.
<about nurse> The nurse was fine, I had a lot of attachment with 2-3 nurses, I had gone many times, so much nurse was fine, it was enough anyway, the rest of the number 20 is the same in the ward. So, we speak in a very eloquent manner, we are afraid to ask something again, whether we ask or not. The nurse was fine. Some nurses had become familiar. The behavior of some nurses was also bad. The doctor used to speak abusively. I used to be afraid to ask them.
<Who all communicated>
Senior doctors used to tell. They used to tell rightly whether there is improvement in the child or not. They used to take this decision only if they said that they do not have to give anything to eat and drink, then people did not give it, they used to come the next day and tell how to treat it, what to do next. The condition is fine for the child then he used to say that the doctor said yes, something can be fed or otherwise.
<Frequency of communication>
They used to come to see only one, senior doctor used to tell only once in a day, understand.
<Skill of communicator>

The method was quite good for the senior doctor, otherwise the same people used to go, they can do this to ask, can they milk or not. Yes, he used to answer well.
Death and post death period
6. Could you please tell us about the death and death declaration of your child?
At that time only I and my mother were in the hospital. It had happened in front of me, I was there, it was not known, when he was applying oxygen or not, while applying oxygen, he died. When the doctor was putting in the death, he was putting oxygen, first he said that he should suction every two hours when we know that our child is serious, then we will come to see the doctor or we will go there again and again Had to ask whether to suction it, not to suction it, the doctor used to look at it and said, "It is not working right now, it has to be done suction, then its oxygen pipe was completely tight since morning, then I said ma'am Tight is going on, then again saying that it has to be suctioned, then they sent a band who was learning things there and while my daughter was serious, at that time, the doctor sent such a band now to her Do you know how to do it so quickly, do not have to do it because oxygen is not going that little at that time, if done suction, they were doing it by removing oxygen, I was there at that time. Just like they were doing the massage of the heart and the people there, then after that, they had applied injection to increase BP, to increase the rate of heart, then it remained

close to them for a long time, if not, then we said that we had injection Have you tried it now, watch 10-15 minutes, then I was giving oxygen from the same oxygen pipe, then after a while, the doctor told me that the baby is not there. Earlier, it was said that it has become very dirty, meaning Pneumonia, and it was 3-4 days when he saw it, there was no difference in his breath, he started breathing fast, breathing fast, understanding the medicine It seemed like it was not making any impact, I do not know what treatment was being done, I did not understand anything.

<Who declared death>

At that time, he was junior doctor, in coming to the senior doctor, he might come around 9:30 in the morning, his (baby) death has taken place in the morning; At that time there were junior doctors, they did not come and told me that they were the same, mother was also there, when the doctors were removed, then they said that you try many times, the heartbeat comes, but at that time there is nothing like it Was not, yet I pumped for 10 minutes, nothing happened, nothing was seen, nothing happened in the child, then the doctor came and told me, he did not give any sympathy, he just spoke And went again.

7. What was your and your family member reaction to the death of your child?

I was just crying, I did not understand anything, at that time my mother was taking care of me, we were two people, when the husband came to know, his BP was also low, there (S Hospital) brother came. , husband did not come to the hospital. His health had deteriorated neither when he came to know. Brother, I had called him immediately (brother) when I was changing the oxygen, I had called him only then brother had sent me.

8. How was your experience about the procedure after death till handover of the body to you?

<About documentation>

Brother is doing

<Time taken>

The brother had come there when he had given it, and the sign was made by the brother. Did not take time there. Those people stayed here with us because from here, these in-laws, mother-in-low, brother-in-law did not go from here, they were saying that we are living there, then we will go there, so these people time to come got it.

9. Who all from your family and community/neighbourhood were present at the hospital and how they supported you during this period?

Post death procedure and coping mechanism

10. What all rituals and procedures did you follow after death of your child?

From there we came till one o'clock. Then I came to my in-law's house. The shape is then buried. Just like what happens in us, the bath was washed again after washing it.

<Who prepared body>

They seem in our relationship, uncle, they did, Yes she was from the in-laws' house. I do not know which cemetery they went to only gents goes there neither will my husband know which cemetery he went to. We do not grieve among children here. No, nothing happens in our children. Nothing normal happens after feeding because a small child is deemed to be a crime in us.

<About time>

brought home, then took him to the cemetery within half an hour, his soil was added around 4:30 pm and went till five and a half in the evening, and we came home from the hospital about one o'clock, yes around one and half o'clock Came near

11. How did you, your spouse (wife/husband) and immediate family members cope with the difficult phase?

Well, remember, I still do my housework, I do a little sewing, I do sewing at home, not outside, I do sewing already, from the beginning.

<Religious activity>

Yes, just praying for Namaz and praying for him, the rest is housework and nothing else.

12. Is/was there any change in the interpersonal relationships between you and your spouse and other family members after the event?

No there is no change in our way of life. Nobody handled me at that time, I took care of myself and did not have any support, after two to three days I went to my house and then after four to five days I came here.

13. How did the family members support you and your wife during the difficult phase?
No one comes around to support the neighbourhood they do not mean that our in-laws do not mean anyone. I was not even here, but the neighbourhood does not know what happened, what happened, what they would suddenly say they had just come to see the in-laws keep it from the three people the same people had just come because no one knew anything, because I was living in McKay. Even the in-laws do not speak the same, the above was their wish, she had her wish, she took it, she also had a lot of trouble, she would have recovered if the operation was done, we went out of the way, went to the curry, went to the hospital Had gone to max, Gangaram was asking for four and a half lakh rupees for the operation, now there was not much money, if it was done by operation time then it would have been fine.
14. What were the reactions/ counselling and suggestions given by relatives/neighbours/ community after the episode?
Right now there is no talk to anyone there is no talk about it with anyone
15. Do you think and/or discuss with your spouse/ family member(s) about the death and related events and blame someone/something or feel guilty for the same?
I feel the fault of A Hospital people and S Hospital people also feel the care of both and there is not even care in S Hospital. If you are referring, if you know the condition of the child this is the condition, you are also saying that the operation should be done before one to one and a half months, then you have to do that, in this thing you will have complete ways, if you are sending If not, send it in such a way that in one to one and a half months, they have done all their tests, whereas eco etc. all the tests are here, that girl is finished fighting while fighting with her life. She took S Hospital Referred Paper. I doubt that while on the treatment of (S Hospital), I did not understand even some treatment.
<Blame someone>
I cannot tell you anything right now and I cannot say anything further, right now I do not consider anyone guilty of this incident, it is nothing like this. Yes, she was stressed at the time, which she has to give like the one above, but cannot blame anyone, tension was there, yes tension was at all times even when I was pregnant. Yes my delivery was normal.
16. When did father and mother (if working) return to work after the death of the child?
Right now my husband has joined work from 12th, he is going because he had left the job at that time did not have a job, has joined from 12th to another place.
17. How your and your family's life has changed after the death of the child?
Everything is the same, there is no change.
Knowing cause of death in detail- Autopsy and MITS
18. What was the cause of death of your child and what is your perception about the value of knowing the cause?
The doctor said that Pneumonia had become very dirty and he had the same heart problem.
19. In your view, what more could have been done to know the exact cause of death and the reasons causing the disease?
I don't know
20. Are you aware of any method for identifying the exact cause of death?
<About PM>
Yes, I know a little, I don't know much. That means it shows that because of which, what was the problem, why it was dying, what was the reason, I do not know much more than this, I do not know the process.
21. What are your views about the autopsy/ post-mortem for identifying the cause of death?
<Religious aspect>
It is forbidden in our religion
22. What are your views about collection of the tissue (like done for biopsy to diagnose diseases) and body fluid samples for identifying the cause of death?
Yes it should be
<Religious aspect>
No, so it is not forbidden. Those who bite the part are forbidden for that, yes it can be done according to our religion.
23. How parents and family members can be approached for the tissue and fluid sampling?

<Who should approach>
Yes, if the doctor had said, then there would have been a lot of doctors who were right, they understood well and talked well.
<Whom>
Speaking to my husband, or meaning I used to ask him (husband) to say like this then if he had agreed then something could have happened.
<Explain about MITS>
If you are not explaining to me well, nor can you explain in a similar way, then it can be understood, yes it is possible.
24. In your view, what factors are important for acceptance of the tissue sampling by the parents and family members?
There is no shortage in future planning and treatment, it is important to know
Decision making dynamics
25. In routine day to day operation who takes decision about the major activities (purchases, treatment, spending money) about the family?
Mother-in-law takes, yes father-in-law.
26. During the hospital stay who discussed with the doctors/ nurses to make the decision(s) about treatment and other procedures.
I used to take it. I was at Mother House at that time which is Sangam Vihar. Yes, We were separated from there, actually we have been here for some time now, do not know further whether to live together or we were separated, yes our family was separated, we do not know anything of the present. Only after we have come here and later we will be seen that we have to live together, what is not known, the husband used to walk at that time, although I was the only one who had taken my daughter, they do not know that much. I only know about the treatment.
27. After death of your child, who decided about the cremation and other post-death activities and rituals?
They were taking our father-in-law.
Summarization
28. Now, we are completing the interaction/interview. Would you like to add anything?
There is nothing to say, I just want to take good care of the children there, any parents, because I had not seen anyone who had that oxygen, nor have I gone there (S Hospital) five times, hardly any half-child. It was okay, the rest all expire. There is a deficiency in treatment and there is no care, let us see once right, if any problem is happening, then we are telling people that such a problem is real, in that also those people are irritable, speaking. Do not be afraid to see this again and again, when the child is serious, then the parents will not panic then who will panic.

In-depth Interviews- Parents of deceased children 6

**1.1**	**Primary respondent (relation with the child)**	Mother
**1.2**	**Age of the primary respondent**	Mother-20-25 years
**1.3**	**Other members present**	Grandmother, uncle of child

During interview child mother, grandmother and uncle were present. Father was not at home. The child stayed for 5 days at S Hospital.

2.1 Can you please let us know about the illness and events that led to death of your child?

Uncle: This was born a hole in the heart.

Grand Mother: Birth, there was a hole in the heart.

Mother: It was just shy, and there was a fever one day.

Uncle: I have been telling from the beginning, I was born in the village, then after two or three days, I was crying a lot, it was blue, it was red, then if I kept giving medicine to the little doctor, then be right immediately. Go, when E.S.I. When he reached the doctor, he made an account of the whole, that there is something wrong in his heart, there is a hole in the heart, according to which it is bluish-yellow, he said, "Go and show it in Delhi, etc. Then we brought him to Delhi, and we did the first day in the medical (A Hospital) recruitment. First admitted in the medical, there was no hearing in the medical there, after doing full formality, he gave an estimate of 80 thousand rupees, nothing happened there, then we knew that he gave us the BPL that you have, BPL was not in a hurry we forgot everything in haste or we have We made it simple, then to look back on the other side, we showed in S Hospital, in the new one, from there we showed it to the BPL, nothing even happened there, the same was raped, treated Kept hiring day, etc. There is no thought about the operation.

Grand Mother: Stayed 15 days, meaning slightly corrected, just discharged.

Uncle: What is the name of the doctor, well if his operation is done within 15-20 days, or within a month and a half, then there were chances of survival. Was a boy.

Uncle: The problem was such that the doctors used to say that you must have listened to the hole of the heart, but here you keep that disease and this one is equal to two, these drugs speak something which makes good filter, they were in bad shape, and bad What she does in blood has come in good, the heart was also dry, the drugs were blocked, etc., she was very strong, you will see the reports or not.

Grand Mother: Both the files are stored son all. < S Hospital had taken or referred to himself from medical> did not refer. He used to say (in A Hospital) there is no empty bed, go take it from here.

Hospitalization period

3 Now we would request you to tell us something about the period of hospitalization for your child?

<duration of stay>

Grand Mother: Then it was 4-5 days.

Mother: 5 days

<course of illness>

This was described as pneumonia.

Grand Mother: Drain a lot, so big and so thick needles in your arms and legs, everywhere you mean it was given a full hole, as many children have not done.

Uncle: In Emergency in S Hospital, put the old one in New, and then transferred from there directly to S Hospital itself, number 18 in the ICU ward, 6 days, the sixth day may have ended, while inside dead in Hospital.

4 Could you please describe your overall experience about the hospital care?

<investigations / treatment given>

Uncle: Yes, there were tests, etc.

<cost of care>

Grand Mother: Yes, outdoor medicine means very expensive two to two hundred rupees injections yes they brought it from outside.

Uncle: Yes, if someone used to ask for medicine from outside, then he would write that go get it from outside.
Grand Mother: In the end, they did not get five to five hundred rupees injections, no matter what happened, only in the end when the child was at the end.
5 Could you please describe your experience about communication by the various hospital staffs during hospital stay?
<who all communicated>
Mother: The doctor used to come, the sister like you used to come, meaning the big doctor was just like you used to come.
<frequency>
He used to come on the round once, like the little nurses would not see him.
Uncle: No, the doctors did not say anything.
Grand Mother: I do not tell anything and nothing at all, if I say something, I used to shout that nothing is there, now there is no condition to be saved, so I said you keep doing it, my luck is left or not. Where you had given a date now, you were asking for money, deposit money and then you will give a date, 80 thousand rupees.
<80 thousand in S Hospital?> Uncle: No-no in medical
Grand Mother: In Medical
Grand Mother: In S Hospital, I do not say that nothing will happen, that means there will be no operation, that is, it can be a medicine for cold it cannot be operated, that it will be operated in medical, Apollo was taken there and how many lakhs. Used to ask for money.
Uncle: It was said in Apollo that if you apply BPL, it will not cost six and a half lakh rupees, and if you do normally, then you will get 16-17 lakh rupees.
Grand Mother: Yes, he had taken him to the AP hospital too, he had taken one there.
Uncle: That was also sent by Kejriwal, he wrote that there means a ladies that sits on the name of Barkha he did not see the BPL. There is no idea that it will take years that too you will avoid the submission line.
Grand Mother: It takes two years, no idea, what life will know, and one is here like Palwal, there brother took two time Satya Sai.
Uncle: SS Hospital
Mother: SS Hospital, near Ballabhgarh, it is government.
Uncle: Everything free, everything free of cost for the people living there.
Mother: Heard the name of All India and returned there.
Grand Mother: Hearing the name of All India and returned it, it will not be here.
<How was communication in S Hospital when you stayed for 5 days>
Mother: The correct practice is that sometimes they used to never come, to see that the medicine that is going on is going on, put it on, and put it on Is sitting.
Grand Mother: Yes, you put it on, and you are sitting, and if you ask, keep quiet - shut up otherwise you carry your child, go, take more, take your child at home, we are doctors that you are a doctor, if you are becoming more doctors then take it home.
Uncle: Doctor you are that we are, the practice is such that the government is the one who gets care in private, it is not there or it is 10% -5%. Suppose it makes a difference out of 100%, no matter what. If you go, the message is giving; the doctor is me or you.
Grand Mother: Two times my boy has a fight son, understand this, as if I asked for blood once, in the middle of January, I asked for blood in the month of January. People sat on a drink, they said, "My child, you are sitting on a drink by yourself, and you are calling me an alcoholic, so all of them gathered together and killed my boy one or two hands and then I got an equal boy. He said that do not raise your hand, he is the one who has killed the child and you are killing him, meaning then took blood at three o'clock. My boy's there, then some blood must have been given to him.
Uncle: Not plated but kept empty
Mother: Had kept it, I had offered it a little bit so that the blood came out to check.

Grand Mother: And the blood was taken so much that we kept watching, my boy is still having weakness, understand that he has put a machine and filled it very big polythene, he filled it up completely.
Uncle: No blood taken
<Nurse Behavior>
Mother: Nurses were also like this, we used to say Madam this medicine is over, you said, if you will put it then you will not say, I will not come and you have to go there.
Uncle: It means to say that if there are 20 patients, then there are 2-3 caregivers, 2 nurses and 1 doctor, now how will you look at 20 patients?
Death and post death period
6 Could you please tell us about the death and death declaration of your child?
<who declared death>
Mother: The doctor who sits there is the one who was there; I do not know when the child was dead.
Uncle: The child is over; the child was dead around 5.30-6 in the evening.
Mother: We did not know, the mother told.
Uncle: They did not tell them, they had told their mother.
Mother: At the time when the child did not die, it was just me, then after 5 minutes my mother came and my grandmother came to my husband after that.
Grand Mother: She had gone to take medicine, did not know where she came but did not find any medicine, someone writes such medicine.
Uncle: What did he write Yusuf Sarai all seventeen sites, etc., I got there, but I did not meet, then I went to Rajiv Chowk, Connaught Place, there were chances of meeting there but there was a crowd I said that the child is weak. Anything can happen quickly; he went out and got a call.
7 What was your and your family member reaction to the death of your child?
Uncle: Direct is not told, they were told (child mother) when they have brought the dead child from there, brought it here, brought it to home.
<you didn't tell about death of the child to mother>
We said that we are taking others to the hospital, they were broken there already, they were shattered, they were alone, and they were not alone. Then I have transferred from here, there is a hospital near my house, Majidia has to take it there, I said a lie and got all the people in the auto and brought all the people.
Aunty: The maternal grandmother, who was the grandmother of the boy, was not.
Uncle: I took a call from someone from the neighbourhood. The child's grandmother was told by the doctor that there is no child in the hospital. First granny had to know, the doctor said that there is nothing in the child anymore.
(Uncle showing us the picture of the child)
Uncle: This child was someone tells us that there is a problem.
Grand Mother: Photo will be of Lalla (child), all buried in it, nothing is kept son, kept so many sack-filled clothes, all the paper-wagons who kept everything as they said In the cemetery, where the soil is buried, everyone said that after putting the cloth on the bury, they said that there are two pieces of paper, filled with sacks, there were many files, some are stored there, nothing is kept at home. , There is no sign of it, that the child (father) has become very mad.
Uncle: No sign kept.
Grand Mother: Now his (child father) knot is coming out here, he is saying what will happen to our brother, when the child is gone; we will understand the son and give.
8 How was your experience about the procedure after death till handover of the body to you?
Uncle: Yes, I gave it a slip, yes it was done.
<Time taken>
He must have written the time etc. according to me. They took a lot of time to make the paper, how many are being made of the paper. I told you that if you do 1 man on 20 members, it takes time, oh yes; you are going to be made, at least after death. After half an hour we have been able to leave, after half an hour we can leave at 6-7 pm.
Grand Mother: Could leave at 6-7 pm, and then brought it home.

Uncle: He kept all the papers in formality, gave a slip so much as to sign me.
9 Who all from your family and community/neighbourhood were present at the hospital and how they supported you during this period?
Grand Mother: Yes, yes, there were four people, the child's mother, father, uncle and grandmother.
Aunty: Four people.
Uncle: The rest and those who were his patients were giving us comfort, they were explaining to us, they were also supporting me in lying that no-no is right, your babu (child) doctor has transferred, has referred the other. Do not take too much tension.
10 What all rituals and procedures did you follow after death of your child?
Grand Mother: Yes, the graveyard does not open at night, and then it was found here at 10–11 in the morning. Yes-yes, bathed means from my law, it is right here among the Muslims, all should work according to the law. Like a bath, then a bath.
Uncle: The child body remained kept throughout the night, such children in relatives do not wait for the soil, should not be taken as soon as possible, the time was lost, we do not have it, there is a time table, Then we started before 2 o'clock in the morning, before the namaz, the elder brother just came from the village and did not see any wait for anyone. Woke up like this throughout the night, bathed in the morning, washed it, then brought the shroud, here is the shop, did everything that the formality belongs to us, fixed it in the shroud and took it back, we said it in the morning. At 6 o'clock to dig, the tomb was ready by 11 o'clock in the morning; all the prisoners were again buried. There were many of his entire family, many from the neighbourhood; at least 30-40 were closed.
Grand Mother: Enough was enough, enough was enough?
Uncle: Grandpa and I bathed the child.
Grand Mother: Dad was crying, he was very mad, he was begging and there, he was falling on the feet of the doctors too when the condition worsened, but where do the doctors listen to anyone.
<rituals after death>
Grand Mother: Yes, it was imparted, like a child would be given milk there for 40 days, like someone who is fascinated, poor, that means a packet of biscuits that a person or child eats, that is given in a little milk. If I used to eat biscuits or papes, then they come in the morning.
Uncle: Which means the poor are more.
Grand Mother: Yes, yes, we give it for 40 days, then if it means a little bit of scope, then you divide the child by tearing.
11 How did you, your spouse (wife/husband) and immediate family members cope with the difficult phase?
<So how the parents have emerged from that thing>
Grand Mother: Understand, they are comforting, they are grandparents at home, then they think that they will give more sons, as long as they were in luck, they stayed with you and the luck If I was not gone, the boy is so mad that my Babu is sitting there, calling Babu, this is Babu, he will go out of the house so loudly, that seeing him, the boys cry out We will keep on thinking, Lalla will give more.
Uncle: Give comfort.
12 Is/was there any change in the interpersonal relationships between you and your spouse and other family members after the event?
Mother: No, no, nothing.
Uncle: No, no tension is there.
13 How did the family members support you and your wife during the difficult phase?

14 What were the reactions/ counselling and suggestions given by the relatives/neighbours/community after the episode?
Uncle: Yes, yes, outsiders also understand.
Grand Mother: Yes, yes, even outside people understand, they say that the son does not go crazy like this, look great, then what do they do, our girl's 22-year-old accident is over, How is she living, now this is comforting here, everyone does not talk at all in front of her child, that she will miss it.

15 Do you think and/or discuss with your spouse/ family member(s) about the death and related events and blame someone/something or feel guilty for the same?

Uncle: No, it is not so, yes it is that S Hospital can hold those responsible, so much that they can do anything when they were fully aware that they wanted to talk about everything.

Grand Mother: No one is responsible.

Grand Mother: That means if they could have wanted, then they could have said that you can give us half 10-20 thousand rupees and bring your children, we can do the operation, they could, but they did not. Did not take it in there in the manner, used to say, bring - bring money, it will be operated, bring - bring, you will not be depositing money, go from here

Uncle: You guys will have to deposit money, when admitted to medical for the first time, then I saw a week, seven or eight days, then they saw that they could not deposit the money, then they used to pretend that they referenced it. Here for (S Hospital)

Grand Mother: Then the meaning started to get more, then I used to run away from here, kept it for a while - after two hours, then from the Medical (A Hospital) in S Hospital that the beds are not empty here, yes they used to keep the same hours and two hours.

Uncle: Now, if anything happens, he used to run away from medical, medical to S Hospital he kept 1st time in medical then used to refer to S Hospital.

Mother: Three children, four children each on a bed.

Grand Mother: Looked a little and used to say that now take S Hospital, yes, it was said that there is nothing to be done here, it is the cold pneumonia, its medicine, the operation will be in medical, nothing can happen, three on each bed -Three, four-four children, yes, four-in-one, five-in-five children in S Hospital, all kinds of good and bad living in it, even if there is no such disease, then the disease will spread.

Uncle: Cleanliness is good in medical, but there is no cleanliness in S Hospital, neither in toilet, nor in toilet

Grand Mother: Yes, so much dirty in the toilet, there is too much in S Hospital

Uncle: In the ICU, cockroach used to be lying around the bed, the boards keep going inside.

16 When did father and mother (if working) return to work after the death of the child?

Grand Mother: At work going nowhere, they have not been able to work for 2-3 years, do sewing, the work is not going on at all, meaning cutting is not done on that day, public knows, if you know cutting Meaning there is no full teller, no cutting work is done Noida etc. has run, the company is not here at all

Mother: Not going now.

Uncle: not a Full Taylor.

17 How you and your family's life have changed after the death of the child?

Uncle: Nothing special, as before you were still living, you are still living, yes there is a bit of grief that at least earlier there was a difference in it, earlier it was to play songs, TV. Gone, that is nothing, no feeling comes now, see the sorrow.

Grand Mother: We are very sad, my condition becomes like this.

Uncle: No, not at all, their husband did not have any bad habit after this accident. This is as bad as it was after, whatever was there, it is improving gradually, I tell myself that I have suffered a big shock I could not collect enough money to get my child treated.

Knowing cause of death in detail- Autopsy and MITS

18 What was the cause of death of your child and what is your perception about the value of knowing the cause?

Mother: Nothing directly said that the child is no longer there, its shock has gone, they gave some reason why the child is finished.

Uncle: Didn't say anything.

Mother: Then after this I was sent out, my mother explained that there is no strike, then started saying, try and see if there is a strike, then they tried for 5 minutes, did not in front of me, tell the mother She had been, then told in front of him, then did not tell anything, just phoned him (child chacha) and he came.

<Perception about the value of knowing the cause>

Uncle: What is the cause of death? This is just the child was ill and in exactly the same condition, when the doctor admitted to medical (A Hospital) for the first time, then this operation is as soon as possible before two and half months - If it is first, then it is fine, otherwise there is no chance after that, even if you do the operation or not.
Grand Mother: Now whenever we had an operation guarantee, there was nothing
19 In your view, what more could have been done to know the exact cause of death and the reasons causing the disease?
20 Are you aware of any method for identifying the exact cause of death?
Uncle: Yes, just like there is a death, accident, etc., hanged and dies, then post-mortem takes place, it becomes known, a government case.
21 What are your views about the autopsy/ post-mortem for identifying the cause of death?
Uncle: They comfort their hearts that there was a disease, heart attack, etc., that heart attack came to that person, it happened.
Grand Mother: Yes it is done - it is done, a person takes peace in his heart.
<ritual aspect>
Uncle: It is not justified, we do not want to get any death done here, it is another matter that there is a long case, if the police case is there, then forcibly.
Grand Mother: There is no such thing as dead body.
22 What are your views about collection of the tissue (like done for biopsy to diagnose diseases) and body fluid samples for identifying the cause of death?
Grand Mother: Yes, we would have got it done, it is a little bad, it is a good thing, it is good, it is not forbidden for this test, that when our child is gone, it will be so much more that it will be known to the heart. It will be comforting for what reason my child has gone
Uncle: Yes, it would have confirmed it, yes it would have cleared us what caused the death
<religious aspect>
Grand Mother: There is no prohibition of religion in this.
Uncle: There is no prohibition of religion in it.
23 How parents and family members can be approached for the tissue and fluid sampling?
Uncle: It won't happen without parental permission
<whom should they approach>
Which man would be in the responsible of a doctor like I
<who should approach>
Main doctor should tell him
<mode of explaining MITS>
They say the same (main doctor) in their mouths that we will take out every part with some injection and after testing it will tell what has caused your child's death
<when approach>
After giving yourself some comfort, you can come to the side and say that if you explain such a thing about sudden death, you will not understand it soon.
24 In your view, what factors are important for acceptance of the tissue sampling by the parents and family members?
Uncle: Now we have come to know that this is new technology that we will be the reason whether the doctor has died or the child has died due to illness.
Decision making dynamics
25 In routine day to day operation who takes decision about the major activities (purchases, treatment, spending money) about the family?
Mother: They used to go together (brother in law).
<treatment>
Uncle: I take decision
Grandmother: S Hospital used to take it anywhere

26 During the hospital stay who discussed with the doctors/ nurses to make the decision(s) about treatment and other procedures

Grand Mother: So they (child Uncle) were doing all this work here only.

Uncle: I have been the main.

27 After death of your child, who decided about the cremation and other post-death activities and rituals?

Uncle: Those who were educated there are maulvi, etc., who tell all of the child that to do this, that is what the father allows, allows and the first three fists are the only father, then the grandfather Then add any.

Grand Mother: Those who are educated tell them as they are

Uncle: No-no never consulted anyone.

28 Now, we are completing the interaction/interview. Would you like to add anything?

Uncle: Well, I have my own walk in S Hospital; I have just had a baby girl on August 15, the last girl, so copper-T is putting copper-T on everyone's side of their choice

Grand Mother: Yes, put it on everyone, now it is hurting, it is causing trouble

Uncle: Ever since he got copper-T, the pain, swelling has increased here, there is a normal check-up or not, all is right, just leave the medicine and food, not taking any combinations, we want That the two copper-T got out, why the sagging aunt in her family had got copper-T, she was in a very bad condition, remained 25%, after that the copper-T came out, she got it right and the same happened to her aunt's girl. Copper-T was in place, the same happened 25-30% of their chances, then the Copper-T came out, they were correct, We have done this for two cases in his family, we have also gone to remove the copper-T and postponed it and then they are not coming out and then should not be applied without permission, he has said that after getting the child, the copper-T will look like yours, so he said I will not do it with my will, my husband is outside and my mother-in-law has to ask him that yes we will ask him later, You sign, forcibly sign, he signed in fear that he will threaten many children there, etc. I will do so, that is, the nurses will kill one of the ladies just like my wife. Don't know why the wrong behaviour means yes to copper-T.

Grand Mother: Yes, they start speaking upside down, they die, and they turn a lot.

Grand Mother: What would you like to ask, now that they have a future ahead?

Uncle: Just give me the opinion that how the copper-T will come out, my wife is suffering a lot.

In-depth Interview- Parents of Deceased Child 7

1.1	**Primary respondent (relation with the child)**	Father, Mother
1.2	**Age of the primary respondent**	Mother-25-30 years<br>Father-30-35 years
1.3	**Other members present during the interaction**	None

2. Events that led to death of the child

2. Can you please let us know about the illness and events that led to death of your child?

Father - Mam actually happened, the girl was at home, and all three of us who were born have also been at home, all the children have been at home, this happened once we had all the children of the three children. The thing had become of MM dispensary, from OPD, he took it there and showed it to the elder boy, it is time, just after bringing home, delivery was done, when he took the middle girl, he started saying that S hospital has to go here is It is a month's time when she was also brought home, the younger boy did not take any time for the boy, in fact it happened that she comes to our house as a nurse, so do not take her away when the two made up their mind So do not take it, you did not carry it, tried to carry it this time as well. Asked not to speak to me, left well, what happened after 10-15 days, a little bit came, this was the fourth child After coming and coming I said to take the MM dispensary, when I took the MM dispensary, then the wife was taken. She said that she is telling the hole in her heart I said how can it be done, such a niche was set, and there was a hole in my heart, I could have said, these MM dispensary doctors had told, I said more experience will I have said, I can transfer and then transfer S hospital has happened, so I said go get eco wager, then we will see, if eco etc will be done in front, then I saw that there is a hole, I asked the doctor, I told him that he was telling about 3-4mm eyelet infection, everything was telling. After treatment, the treatment continued and 2-3 times were admitted. Blood also happened, everything happened, just look at what we say is ours, so let's say we have an unknown expression, something that happens may be a little bit from the present to the parents, some may be such an education, some may be careless and the doctor people also have to do a little duty for 24 hours the same night, some people feel tired at some point in it, who went three times, the girl was completely come. For the fourth time, if the old doctors were not found, then the girl is a bit careless, the old doctors were not found in S hospital, there was such a good madam by the name of Deepali, I talked to all the people, but I am not good I thought, because she never used to come, I used to think that laughing at a crying person is behind the big success and anytime the big men who feed the baby, they would have fed very well even if they did not have the desire. She used to play, she used to speak and recognize everyone it was just that, just a little mistake has been made from them and for that I will say that they should pay attention to what they have done from their side.

Hospitalization period

3. Now we would request you to tell us something about the period of hospitalization for your child?

Father - last 6 or 7 days admit and or who has gone, has been taken from here in the evening on 20th date and expires around 3 and 2 pm, expired on the 20th of the night, yes it is a The day before it happened, it was 4-5 days, it was admitted, the treatment was done and then it was going to be discharged, this was the fourth time when she had gone to admit, she was removed from her in the emergency and went to the ward and negligence in the ward. Happened It is not that there is no negligence, at last he felt as if he had too much cold and that there was a hole in the heart, then the infection was spread by him. The eyeball catches pneumonia very quickly and becomes double. Sometimes, in this round, we have seen and went to see it and it was right after taking it. Husband, quote, everything happened, after that I came and had three children at home. Wife was telling that often on the night when her first two three girls turned up once, for the second time, the tightness of such hand went on again She went. The wife was in the hospital with the rest and she was alone, there was a pump on the ground, still there is a shovelling, she is walking, see what happens, what does not happen. The doctor is nothing, actually the one who was about to leave is gone Now the dead body is left, the doctor said, yes this is the feeling, I see two minutes and

you press the pump, I tried for two minutes from my side, every parent tries from their side and often this If it happened, then after 2 minutes, I removed the pump after five minutes and say, take it away. In the last case, Madam, I did not ask anything and at that time the wife would be crying, I could not ask it and I am feeling sir that the wife was saying something, something has definitely happened.

4. Could you please describe your overall experience about the hospital care?

Mother **-** Doctor, it was right 15 days before it started, it became normal at home, the child was not able to feed, so this little boy fed him while feeding him, feed him comfortably, I told him not to feed my son. , So he said that he is a very good person himself, so I said let's get medicine, I can't leave Babu like this, I went to MM dispensary, after going there, Doctor Sahab said what happened to this little baby, I said yes he has a niche I started to check, I said that it is not bad That boy is bad, I said, so he said that the boy is not bad, this girl is ill, why did I say that there is a hole in his heart, I said how such doctor, we said that we are referring to S hospital and want to eco Is, do not be negligent, we were taken in emergency, went there and asked in emergency, if not such great doctors said that they sit in OPD, they do not sit in emergency. You show me in it, then after that I made a slip in it, then I got everything done in eco, after getting the eco done, Dr. Saheb said that you will come to Friday, that day the heart doctors will come there at 2 o'clock. It was a good girl for us to know that the doctor knew that now, when the doctor said that the child will have to be admitted, the health of the girl is very bad. One day, I was feeling ill while doing home, I was also feeling ill, I used to drink less milk and wept blue. It was after that, she was admitted in the morning, got discharged in the evening, the doctor said in the morning the child is right in the evening I had to leave at four o'clock, I was admitted for the first time, I did not know what the meaning of recruitment is. Evening is a holiday, leave at four in the evening. Then said nothing happened to bring again, for the second time, in the emergency, he said that the child is very ill, the cold had increased and the light puffed up, that day increased as if he had to need a medicine and his medication was not there. Was getting a heart CF was not available, wrote tablet, said four parts, one part to be fed and the fourth part, then I started to drink by myself, the other one used to drink medicine like urine. And his weight also started increasing, slowly the weight increased, the doctor said, son, now there is no need to panic, heart holes are being filled and there is no such problem. After that it was x-ray for the second time. The old people and eco were the old ones who had done it before, then the field doctors had come, they were older, they said, the child is right, he went for the third time he had such a mild cough, so let's not take care of this heart Doctor sir, Lenny will have to pay the child may die So I was scared, but I went again, it was ten days in the third time, the doctors just said you take it, there is no cure for it, I did not know at that time I did not know whether there is a cure or not, Dr. Saheb did not tell, I had not seen this thing, so I did not know that he had said it repeatedly. He is senior, saying that the child will die again and again. Say the same thing then we said, Sir, can you tell me something? People say that doctors are in the form of God, they are not saying that we have come here so how can we refer, then what should I say, then I said okay, I am looking, blood and blood started to rise, but there is a lack of people There used to be those who used to sit at night, three out of 10 doctors were right, but whatever there were 7 or 5 who were like big doctors come in the field, they are telling us something. So let's go and sit there and scold him so badly, after scolding, we used to say that we are for the emergency child, it is not for your child who was going to die more than the child, he used to sleep in his own room. Used to get stuck, when the doctors of the field are going to speak anything, there is no medicine, then tell me, we do not have it, so if we do not bring medicine, then we could have explained it that we were lacking. Did not bring medicine and Dr. Saheb used to speak when he used to come to the field, if you do not give this medicine, if you do not get the needle, then you will not bring any matter from outside, then you will see it and will talk again later. He used to suppress the same thing he would not let us speak further, when we used to say this to Doctor Saheb, Doctor Sahib used to say why you have not written problems. Mother should know, Dr. Saheb used to say that when senior doctors come to the field, more comes Dadagiri, he will die one by one, who used to speak like that, senior doctors used to talk well "son like that No, "He used to talk very well about medicine, he was a senior who used to sit at night. He was very good. He kept me for the third time for four days." Here we were two, there is a front street, there were also a child, we were two, my daughter was weight, 4 kg, 24 grams said son weight is right, the steam used to go and the oxygen did not

reverse by applying steam. They used to get some rest again, they never got sick this girl was such that many people used to say that the child is right, the operation will be done in the sixth, but some people were right in it. Some were wrong, they used to shut our mouths in it and the children of the house are small, so we had to think about everything, carelessness is more in it, the girl finished the fourth time, yes, the fourth time I went, the girl was lightened at 11 o'clock. I had a cough, so I was afraid, took it and after going there, I had milk, after feeding, it was just that, the doctor was sitting feeding ladies girl Bid once you can see my report started saying that the treatment is going on from this, I showed all the reports of all the recruitment papers. , Then bid this girl will have to admit, she should not steam or put a bottle, just put so much needle and then reach there, she said, why are you bringing this sick so often, I said, I am in my hand, started speaking He will remain sick like this, he will die like this, he said the same thing again and again, I did not feel bad after that the doctors who sit there for steam from there do not steam in the emergency Earlier, we used to give a 15 to a 20 in a 5 minutes on a 10 minutes, but these doctors who were sitting that day, the ladies who were there, did not say anything, I was very stubborn, I met an old doctor. I started crying with them, then why are you crying, I said doctor, its health is a little worse, then I am saying why I said that green is making green latrine And the eyes started to do like this, when I was about to die, I did not die when I was feeling very bad, it was good enough, the doctor gave the medicine to steam, the girl turned around, started playing after turning, she had some old patients, she came. They are saying, 'Agni', I said yes, everyone spoke with fire, after that, the girl played her husband, then after that she started having strange eyes at 11 o'clock in the night, then I went away. If you say something about sleeping, when you go there, no one would meet, if you go again, we used to listen to data, when the health of the child was very bad, sit and sleep. What is the matter, doctor sir, he is speaking again and again, why you are getting scared that oxygen is coming out, is there a dare that the child will die, I said doctor sir, you can see once. I used to see one thing again and again, it did not work well, the life had already passed, the steam was right to turn back then wrote the medicine. I went very hard to get steam medicine and steam medicine As long as the girl was right for the part, I did not let it steam, I did not get steam, the child's heart would be stressed, she did not die much of try, she would not die if she let a steam Heart-born children do not increase their weight when they die, when there is a problem or there is an allergy inside it, even get everything done for a date, do not understand that we are unaware you are a doctor, you can see that report. Do not ignore that thing, let us know what is the disease in it, it can run medicine, otherwise it is not possible to answer the child Then in the morning after that, saying before the junior's arrival, remove the body very quickly, it used to be that no one with my child will die if the child ends up with him at 4 o'clock at 12 o'clock as the field is known That the doctor is going to come, after 8 o'clock, before clearing the body, he used to leave the hurry, take the body away. , When we have luck, let's have three, we had fourth, we did not want it when time has gone, it is not revealed that when the one above was given to us, we did not take it, it would not have been a sin if we had killed it, after taking such a disease. It started happening just as the doctor said, but for the fourth time he was careless.

<about investigation>

As the doctor took the blood of something, he used to give it to us. He used to tell us to stay safe. When we used to ask, he would tell me to be told, one hour and two hours he will be spoiled. Were, when we used to speak 2-3 times, the great doctors used to come on the field, when we used to say that it was your fault, we used to get bogged down, then we used to be silent. Tell us if there is a shortage in the morning before we were told, people used to do field work, which used to write what happened in the file, they would not let us speak.

<about cost>

expenses, what will I lie to you doctor sir, I am a poor man, his (husband) is also suffering from kidney, medicine is going on, I have three children, there were some of my 15 thousand yes medicines, some were written outside. Were not found in S hospital injection medicine was not available even in it, which was not available from outside, so there was a girl in it, she was a girl, she was right when I used to cry, do not cry, she used to help me, so sister should ask for anything. I used to ask for helplessness, but I did not feel bad about anything, it was just that the girl should be right.

<about behavior of HCP>

It was strange, it was not good to scold, the nurse medicine that people who are learning like this were a bit right, like anybody is a little proud, ask lovingly, when you can scold you can explain but only one doctor was right in that Deepali doctor. The child is in the ward 20 in the H block.

5. Could you please describe your experience about communication by the various hospital staffs during hospital stay?

<about communication>

No, they used to read on their own, they used to understand on their own, they did not tell that the medicine is running, they were also telling some such medicine that Deepali is running, if that medicine is used then it will stop.

Death and post death period

6. Could you please tell us about the death and death declaration of your child?

Doctor sir, like I said, after calling in ICU said that your mind is bad, mind has gone, if you do not have courage inside me, will you see the child? Why is not it, when I was fumbling, its leg was cold, so I thought fever would be going up, I used to measure with a thermometer in my head He never had a fever, then the doctor said, fever came, do the bandage, I started bandaging, I started jerking the bandage thrice, I could not try to turn three times, after that, she was tearing eyes and her eyes As you sit, neither was you agile, but you used to say, Mama play His life is coming out, these people did not come, I did not know, I was sitting with my oxygen, after sitting, I started crying a little more, the girl is not moving, which is not like the baby is not automatic. She is afraid, I went to Deepali, I was not a doctor, but I forgot her name in it, she was also right, I said, doctor, you see, see if this is a child, I have not said it means my child. Is in front what not? Inside this, I could not understand the gestures between them for 5 minutes. After that, bring the pump that is pumped and tear the mouth and put it in the drain, remove me then after that one is not saying now. Do not put the hose out, said it, put the steam, I made the boy sit with the steam, now the doctor goes out of his life. So the child shows life in their eyes, when it is their time, then they remove it, so do I, after bringing the needle at 3 o'clock in the night, I said someone with you, I did not bid, so I went The person who brought the medicine to steam her running, said after 2 hours, apply it at 5 o'clock, I said, "Okay, then call your family, I said that I am very sick, then you are crying, then you will What to call, I called her husband, she said this phone, what is the matter, I said, doctor, keep calling After that, after doing a full hand shake green latrine, Dr. Saheb came saying that he felt steamy. I got dialled 2-3 times, did not hear anything, and did not hear anything. Get a little hand started shaking, after that, doctor said, now it is good, then I started doing like this again, I kept running and crying, saying what happened then the child will not die How much should I say, do not bring here, do not bring, like it was finished when you put the pump on, it was over 10 minutes after 4 o'clock, then they do not speak a little bit, half of the children come out of their mouths as they come Started as soon as the drain was poured out, he often did the same thing, he will do the same thing for 3 days and 4 days, a child will go very well. Is, I bid you guys.

<cause of death explained>

Did not tell why Mara was killed, just said that there was a hole in the heart, it was said that there was a hole in the heart, I had gone out.

Father- It did not tell anything that there was a hole in the heart, so the child was killed, all these things were talked about; when the child is finished, then after that, take away the dead body. , No Meaning

7. What was your and your family member reaction to the death of your child?

Mother **-** crying there when it was found out that the baby is not there

<did someone try to console you>

The son started thinking that he was destined, the guard was right, he was also poor, the doctor was also there, in that he gave me Deepali (doctor), she did not come, so I was afraid sometimes she loved him (m) . Very good nature, no one is angry with anyone, no matter how much, someone is personal, no matter what anyone means to me, we do not mean to him, we do not mean to work. Whenever she meets, she works with one mind with a passion (father). But this time, the doctor who had done the report, he was getting scared about the eco for the second time in the same way, the doctor said, if someone is with you, then I did not bid, then say good you come once again. Then for the second time, when they used to do it

for the third time, they used to come round as a round, then kept it and had a child, then call him below and call him too, then do not tell anything after that, all these tests have been done in S hospital new building. In, the report that went to the Emergency, if they had seen the doctors, they could have saved it all. They ignored the girl, did not see the report, and they were asking for the third time that the recruitment papers were there, they suppressed there, no old doctor had met me or met only one and Deepali Doctor came in the morning.

8. How was your experience about the procedure after death till handover of the body to you?

<about documentation>

Paper was signed on the father (father). I have (mother) so big to tear it.

<about cost>

no money nothing (father).

<time taken>

It took 10-15 minutes to do the paper work, and nothing was done (Father).

9. Who all from your family and community/neighbourhood were present at the hospital and how they supported you during this period?

Whoever did the help with our neighbours and the doctors in the hospital and some of the people who were with me went to see a little listening to the bathroom to talk.

<what type support given>

Neighbours also made money with the help of children and even after washing their cry, it remains to be helped in everything.

Post death procedure and coping mechanism

10. What all rituals and procedures did you follow after death of your child?

Father- 4 o'clock in the morning, the girl was finished, so I waited on there and called someone from the house, and had to bring the wife also, I had to bring the child too, then went auto from here, I have my own auto here, I have them Had called and they had gone there, it was at least 6 o'clock in the morning to take the whole thing from there, in the morning, we left from 6 o'clock in the morning.

Yes, a lot of people came from relation, father etc. My mother had not just come from my heart, my mother was finished with cancer, my father is mad and my younger brother village once asked about poverty, then he went out and all my in-laws came from his mother-in-law, his father-in-law had come, mother-in-law had come. He was in a relationship (mother).

The mother-child has been recording when she was 6 days old; it was over when body came to have a photo. We have 3 children, we will be patient, after only one day, after how many days the mother is there, the mother is so much, the mother becomes mad for the child, and the disease remains something else.

Father- After coming home, all the relatives came and just took the dead body, then the body was washed and everything was done, then they were all dead and then they took the dead body to the cemetery in the hill. I want to say that the head that happened to us should not be with anyone, I think that the doctor who is coming to us is the future. It is his duty to see someone and tell someone that his religion is from one side. The doctor is the form of God is actually right, Because if we do not do it all over the place, then who will consider them, they say that no one has seen God, yet they believe that the educated people believe in them, they believe in them, otherwise I want to say that when we believe in them If they do, then they will not trust us until they follow up.

<who prepared body>

We were served neighbourhood they did the work, they did all the work, there is a little 10-minute road nearby, the forest is all dead body goes there; yes we did bury the baby's soil. We had gone to at least 60 people, all the people had gone, friends, how many people could not know because the relation was very big after working in the field, how many people started saying, I did not know, I said that was to be He is done.

11. How did you, your spouse (wife/husband) and immediate family members cope with the difficult phase?

Father - Look to get out of it, ma'am actually comes out the same thing, as much as what you think will feed in the mind, if you have to do something new to get it out of the mind, just work a little here and there, To show that I have not read till 12V in the field, yet I have to do all the work, I had to explain to them that a lot of things cannot be done, what is possible is possible which is impossible. is impossible
I have to manage myself, I do field work, the field worker will sit in the house with the people, so actually it will be as if I am reading this by taking the paper, then the focus will be on that I am reading the paper. I am a worker in the field, goes to the field and talks with the people, a little mind is removed.
Mother - Now everyone started saying that your child was small, the children of the heart do not live, now I did not know that much, who said that I listen to everyone, I cry in the house, neighbourhood explained, do not cry. On top of that, her (her) kidney is bad, she also thinks that she has gone whoever is 4 days, 5 days, 5 months, whichever day she is, now I think that she should be treated, so I think where will the money come from on rent? I live with 3 children, in that the first report showed kidney failure Now if I go, I say the stones are bad and the medicine is given a little comfort, I keep on worrying a lot, I think I will not be able to walk anywhere, I will go with 3 children and no one is tight to the in-laws and no one is in-laws' tight Will feed 3 children.

12. Is/was there any change in the interpersonal relationships between you and your spouse and other family members after the event?

No change is like before it is still there, not like it was before, it is still there, we talk in the same way, and there is already a little mind. What I am today was not there before whatever happens to get education is very good. I never really understood what all this happens, because I am a working person living in a village, he does not know what he is working in agriculture, he knows that it takes crops, crops are harvested in the house. He does not know what things are planted in the crop, how chemical is added to it when the crop is ready.

13. How did the family members support you and your wife during the difficult phase?

14. What were the reactions/ counselling and suggestions given by relatives/neighbours/ community after the episode?

Neighbours just say what has gone to sleep, if this is what it is, get out of your mind, do some good things, we have 3 children, think of their upbringing. The suggestion was to find out the cause of death, brother, there was a hole in the heart, because of that, it happened to us, even the actuarial doctor suggested something and neither did it tell us that yes, the disease was caused and that it went to the doctor. Did not happen And there is no problem even with the senior, we said, let us have a hole in my heart, brother, now we have to have patience because the status of living and dying is not the same, one day everyone is coming and everyone has to go. The way and the same path will be the end of everything.

15. Do you think and/or discuss with your spouse/ family member(s) about the death and related events and blame someone/something or feel guilty for the same?

No, I don't believe anyone guilty, but actually this is what I will say, on whom I tell you now, the doctor believes the people to be God, they should not believe in superstition, that is all I would like to say, I was patient Took how many people have to have patience, they have a big responsibility, they should understand their responsibility in a good way. There was a doubt in the treatment that you did not give us something that if you do this then your child can do it, and if you are asking for suggestions like this, are we also asked in the meeting, then we can say everything that we cannot In fact, anyone is getting benefit of this thing, in fact, it is the reason that they should benefit and why they should benefit Even then, he needs benefit. Our child does not want his child to die and I want that when the doctors are giving us an example of something, we are talking about something, we are talking about something. We do the same thing. And we have to listen to that.

16. When did father and mother (if working) return to work after the death of the child?

I was at least a week, that time the work was completely closed, now I have to be busy because even before you told me I do 2 working one part time one full time, full time is my clothing business part time my networking marketing work.

17. How you and your family's life have changed after the death of the child?
In life, everything goes on above, I think that for the rest of my children, as long as I am alive, my life will do something for my child, because I want to change my future and give them a good credit, I So today it does not explain that I give very good advice, I understand that I give as much knowledge and as you guys, I get very good knowledge and very good talks and this is what I want to put in my child. I am No matter how much anger someone says to someone, even if you call them dirty, you call it good and fold your hands, brother, you will grow up and we will remain small.
After that incident you have to take more care of the children (father), care has to be taken when there is no money, then you have to think that you have to pay rent. If you do not understand the poor then you sit down silently (mother).
<about substance abuse> No, not all of these, nothing from before, no drinking water from starting nothing, nor even today, I add my hands to all these things, I also tell the people who come, nothing is gained from their bodies. Drugs are nothing but waste. (Father)
<Plan for future pregnancy> No, I don't think of all this, I have a lot, I did not want to have children, for this I had tried lakhs, when I gave the above, then when I found it very late, I came to know after 5 months (mother).
Knowing cause of death in detail- Autopsy and MITS
18. What was the cause of death of your child and what is your perception about the value of knowing the cause?
No don't know about it (f)
19. in your view, what more could have been done to know the exact cause of death and the reasons causing the disease?
If our post is not done, how would we know (m)
20. Are you aware of any method for identifying the exact cause of death?
<about post mortem>
That is why, what was lacking, what caused the child to become a disease. It is our suggestion that when we could not get any advice in life, then after death, what should we do to the soil which is in our destiny and sat down thinking.
Father - Actually this happens when there is no faith inside your soul inside your mind, then it is useless to accuse someone if there is no faith in it, then if we ourselves are thieves then why make the other a thief, if we are honest another thief If brother is there for a minute, then the next may be the thief, we are thieves ourselves, so what does it mean to be the thief of another?
It may be the fault of both people, it can happen to us as well, it may not have happened to us, we did not think of any such thing. I keep saying again and again that the doctor will have to respect a little, attention has to be paid.
21. What are your views about the autopsy/ post-mortem for identifying the cause of death?
Father - <necessity>
It is absolutely necessary to know the cause of death, but some people are also unaware of their lives, what happens is often some people are unaware of their lives because the parents do not pay attention, secondly the parents also pay attention, but the children's count has spread so much In today's time, 12 years 15 years and 8 years 10 years old child starts drinking water, I do not think that if India is such a situation, then the future will be right for us because nowadays there is so much pollution, the biggest is poison. That is And how does pollution happen to our people, it happens to our people, some people pay attention to good things, do not do good things, some say it is crazy, you should do as much dirt as possible and put it on the road Is, they burn it, some people ride the bike as if they think that there is a sprinkling of the medicine of the mosquito, nor does the smoke come out of the bike, then we are the only reason for the disease of Surrey pollution They put silence on the government The government is not doing this, the government is not doing this, I say that there are four sons in a house, one father is unable to explain four sons, then how will the government run such a big world. It is important to know the reason that if you are satisfied with your soul from within, to know the reason everyone will have to be united and work to stop all these things.

Little is known about the big people, it happens that if something has happened within it or if they have committed suicide, then what is the reason, what is the motive, in all these cases, they do post mortem and if I am now 3-4 days ago I am talking about this shooting range. While coming towards Tughlakabad, there is a red well there. There was a person talking about water, he was a boy, he was a boy of 20-22 years, both arms were severed. Eyes were taken out, is there so much atrocity inside India, such a huge situation, post mortem of this thing It is known that brother, why did he have any enmity, there is a motive too, it is good to fight the case and also to work the case, now we press on the doctor in the most useless things Not a good thing but yes actually I will say again and again that the thing that we have faced should not be done by other people.
<time needed>
As time goes on, a dead body is scattered in the entire way, by accident, it is quick to win it, there is nothing in it, there is some dead body which takes two hours and a half hours for 18-19 years. Yes, now it has become very easy and gone in 2 hours, previously used for 2-2 days, for post mortem.
<about disfigurement>
Yes, the body gets spoiled which is a useful thing inside, like the kidneys, kidneys and everything is removed, actually there are two people in my relation who are safe and they say that there are many things which are good It is taken out and wrapped in that amount of time. It takes a lot of time to recreate what is once made, I think it is very good post mortem, people think it is bad that after dying, this body is burnt or buried in the soil. If there is something inside me that is removed and put in the other, if it fits well, then the life of the person can be saved, the person of his house is very good, then I am very good I think Some people do not know the reason for what happens, often they focus on something we do not do and what we focus on, they consider that thing wrong.
<religious aspect>
No, it is not that what is wrong with us is wrong, what is right is right.
22. What are your views about collection of the tissue (like done for biopsy to diagnose diseases) and body fluid samples for identifying the cause of death?
Father **-** Day by day the world is technology, I can understand that people talking to you started coming to my mind also, I can understand that before the body is cut, it was cut and then it is checked. Now this thing is a new technology, I like it so much that the body can be safe and technical check is done and it also becomes a good report. Why did it happen for what purpose, India should progress day by day, this is our morning wish, to progress very well in the coming future, and the country which is outside country makes progress, our India does not progress.
23. How parents and family members can be approached for the tissue and fluid sampling?
<whom>
If I say all these things, then there are some people in the house who say, check, post mortem, this will happen, I think this is the situation. I am or my wife is fine, either uncle Tau is someone else. The other thing is, taking the biggest decision makes a according to parents.
<who should approach>
If senior doctors say that it is necessary to get all these things done then I am the one who has come up with this new technique, if the doctor says to do a little test, then I would say yes if there is any problem then you can (f). This can be known, if the disease is detected then another child will not go (m).
<how to explain>
At that time, I am really normal man this time is enough, actually explaining to a normal man will have a very good effect, but it is very difficult to explain a high patient quality and the mind sometimes does not work for him too. Sometimes when such an incident happens. So I think, if you talk about it for ten minutes comfortably, it can be understood if it is normal, but if it is high patient then it is very difficult, then it will have to show the picture, madam means sympathy to her. Give him a little sympathy; if something falls for something, he has to support something to lift it. Actually after giving support, he should be told that this thing will have to be done, like I believe my wife is right now, if something happens to the children, then I actually told someone to me, then I said, tell me everything is fine, a little It is technical, which is being tested and it will be known that what happened to your child, which disease

was caused, it will be taken with a little injection in the body and nothing will happen, then you can also refuse it. . One can be requested twice.

24. In your view, what factors are important for acceptance of the tissue sampling by the parents and family members?

To know the reason, you only tell it, because of this, you know the reason.

Decision making dynamics

25. In routine day to day operation who takes decision about the major activities (purchases, treatment, spending money) about the family?

If we have to take a decision, both of us (husband, wife) sit and talk, the decision has to be made and the wife says that you have to go home, you have to go to the field, you have to say Which means I can say it but I can't, because that is the reason that happens often, some people go to the field, some people do it from home, so it is good to take both people, of course we meet Takes.

26. During the hospital stay who discussed with the doctors/ nurses to make the decision(s) about treatment and other procedures

I was the only one there, so I used to take decisions, the children used to stay here on the room, the husband used to go to work, see my street child here (Mother). Neighbour is here, see it, it is necessary to work as well (father). His health was deteriorating, his (dead child) was called ambulance so that even when the poor man does not have money, no one is going to know, then that person dies like that, there is no time. , Illness is something and illness tells something else.

27. after death of your child, who decided about the cremation and other post-death activities and rituals?

<who prepared body >

Our neighbours were servants, they did the work, they did all the work, there is a little 10-minute road nearby, the forest is all dead body goes there yes we did bury the baby's mud. We had gone to at least 60 people, all the people had gone, friends, how many people could not know because the relation was very big after working in the field, how many people started saying, I did not know, I said that was to be He is done.

Not in children, I don't know what happens, sometimes I have never seen the perfect mother-in-law, she told me, then nothing happens in the children, just don't go anywhere for a quarter of a month, nothing to do. Yes, she said that her mother-in-law's rituals will not work for mother home, she had told, her mother-in-law had explained a bit, all of my mother-in-law's relations had come uncle, her sister's, tau.

Summarization

28. Now, we are completing the interaction/interview. Would you like to add anything?

In-depth Interviews- Parents of deceased children-8

**1.1**	**Primary respondent (relation with the child)**	Mother
**1.2**	**Age of the primary respondent**	Mother-30-35 years
**1.3**	**Other members present**	None

Context-I lost my husband (he died). I used to sweep before. Right now I do nothing since my child is finished, my husband used to work hard, it has been three years since his death. After that I sweep, I have a son, I live together, but I have work and eat.

Events that led to death of the child

2. Can you please let us know about the illness and events that led to death of your child?

When the child first got fever, the little moths here had brought medicine for him to the doctor, then I suddenly saw one day I was sitting like this, I saw his eyes, then the eyes were becoming very yellow, then I took her directly to K Hospital, which is K Hospital, I admit there for fifteen days then from there, they were not able to do there, exactly, what was there that had also given glucose and blood in the jaundice, Yes in K Hospital and only injections were doing the same three medicines and nothing else, there I saw that there was a small ICU first, then sent it inside Bharti (dr), then it was sent to the big ICU up, big There was nothing in the ICU as well, there were also only glucose, the doctor there said it is jaundice, its brain has gone up Anytime you can give blood from your nose through your mouth, I said that you can give it, then you can do something in it, then we said that we cannot do anything in it, so I said that you cannot do anything, then give us leave, then speak Large we will not give leave, you can take it on your own free will, we will not give leave in writing, I will not give leave, I said, okay, you do not make a holiday, I am taking my child from here Then I brought it out on Tuesday and I took it from there again. In AIIMS it says that the bed is not empty, we will not do it again. We have no responsibility, if you are approved then you can admit, we have no responsibility, so I said, "Let's not take your responsibility, then there is no further hospital, where will we go to pay, then I admitted there on Tuesday." After Tuesday, not on Wednesday, on Thursday, the child has finished at half past one in the night, went on Tuesday, Wednesday has stopped, Thursday has started, it has finished at half past one on Thursday, stayed in the hospital for one and a half days, has also provided glucose on there. , had asked for blood at eleven and twelve o'clock at night, so the blood he could not climb, he had asked for blood, but he could not climb, he had not even filled a bottle in S Hospital, he had already spoken to us No responsibility Signed to me, anything can happen anytime, the child has no responsibility for us, did not tell anything about the chance of survival, just said that it is not our responsibility to save, now the more you lie There is more danger, then he had done recruitment in ICU, also in S Hospital in ICU.

Hospitalization period

3. Now we would request you to tell us something about the period of hospitalization for your child?

<duration of stay>

I admitted there on Tuesday, not on Tuesday, not on Wednesday, on Thursday, the child finished at half past one in the night, went on Tuesday, Wednesday has stopped, Thursday has started, it is over at half past one on Thursday, stayed in the hospital for one and a half days.

<course of illness>

In S Hospital, he had said in the beginning that we have no responsibility, had signed with me, anything can happen anytime, the child has no responsibility for us, did not tell anything about the chance of survival, just said There is no chance of survival, there is no more chance of late lying, then he had admitted in ICU, in S Hospital also in ICU.

4. Could you please describe your overall experience about the hospital care?

<about treatment>

When he had admitted, the treatment was fine, after that he sent him to the ward, in the ward, he did not even notice that the child is bleeding from the nose, we are also speaking, please hurry doctor. No what to do.
<about investigation> As soon as the test was done, the tests that were done in ICU were done in it, there is no test or check up later, 21 numbers were shifted in wards, 21 in 20.
<about cost> No money should not be spent in medicine.
<about satisfaction> She could tell when my baby was fine, everything is fine there, my child's life was so much, there was no fault of the hospitalist, in the ward when I would ask to see the child. If they were, they used to say, "What can we do, his mind is jaundiced. The vein of the brain has burst. "There were just glucose on it. Just glucose came at eleven twelve o'clock in the night. Blood came for blood, after coming blood said that it is still cold. It had to be heated when it was twelve o'clock , So much blood has been put on it, at half past one, two bottles had come, so they were able to apply only one bottle, even if they had not climbed, they had not been able to climb.

5. Could you please describe your experience about communication by the various hospital staffs during hospital stay?

<about HCP>
The behaviour was fine, everything else was fine.
<complete information about treatment procedure>
No, in the evening on Wednesday, you said that you should sign on the paper that the responsibility of the child is not ours, the child had already signed before the end, it was signed twice, once at the time of admit in the ICU, when the second time was admitted in the ward. Was signed on that day at four in the evening on Wednesday, the child has no responsibility, and on Thursday it was over at half past one in the morning. At three o'clock in the night, I was to take home.

Death and post death period

6. Could you please tell us about the death and death declaration of your child?

The doctor said that I was his brother-in-law, at that time in my hospital, the doctor said send him down to his mother, I saw that the child's latrine and bathroom had gone out, I knew that it had died. The doctor came and told me that my brother-in-law does not have your child that you can take it, the child has gone, then I said at half past one, we got a car at three o'clock and brought home at three o'clock.
Doctor told my brother-in-law that the child is not there and the brother-in-law did not tell me, the brother-in-law sent me down that we have the child sent me down, I came to know that the car has been sent down from the top. Everyone has done it, I had to make all the preparations to come home, told me at the time that I have to go home now, then I came to know that I do not have a child. No, no sympathy was given in the hospital.
<cause of death>
The reason for this was that they were telling that the vein of the brain was ruptured, if the vein of the brain is ruptured, then there is no chance of the child breaking even of the older person, then it is difficult to save, the vein of the brain ruptured When the blood started coming out of the mouth and nose, where are the chances of survival, when I was about to come home, then my brother-in-law told me that the car should be, after that he told me that we are now walking home.

7. What was your and your family member reaction to the death of your child?

<reaction of mother>
My situation was very bad when I heard that my son is gone
<did anyone try to console you>
My mother-in-law was sitting outside, she handled the same, and later both my brothers-in-law were below my mother-in-law, everyone else was out of the hospital the child had two people, his uncle and tau.

8. How was your experience about the procedure after death till handover of the body to you?

<about documentation>
Yes, he had given a little sleep that by this you would become a death certificate, he had to be made sleepy, he was a little bit, no, he did not have my sign on his uncle's.

<time taken>
It was over at half past two o'clock at two o'clock at two o'clock, we left from there at three o'clock;
<cost / payment>
body, I do not know if yes, but the ambulance had received fifteen hundred rupees.
9. Who all from your family and community/neighbourhood were present at the hospital and how they supported you during this period?
There were no neighbours here, there were only five people there, but after coming here, there were all the neighbours, my brother came from my heart, sister-in-law and mother came. No help.
Post death procedure and coping mechanism
10. What all rituals and procedures did you follow after death of your child?
I came home at three o'clock in the morning, then we buried him at 12 o'clock, we took him, at 12 o'clock in the day, no child does not.
<about body preparation>
Yes it was bathed at 12 o'clock, then immediately took it away.
<who prepared the body>
His Baba had bathed by his uncle. Tau had all bathed no nothing else is there in the child, only bathed. New clothes were brought new clothes were worn and taken at 12 o'clock. Here it is nearby or buried in Mohan. Do not allow the older child to be buried, burn the child older than fifteen years. My child was five years old, had celebrated his birthday on eleven Tariq, on eleven October, the other eleven could not even come.
11. How did you, your spouse (wife/husband) and immediate family members cope with the difficult phase?
Can't you still manage to forget, I also had my health as a jaundice, this son also had jaundice, he was gone in the jaundice, we had only three sons and his mother, so I am still tomorrow Came for 200 rupees medicine, no work, I work a little at home Right now, I cannot go anywhere for the 1.25 month until my son's. Means stay in the house, 1.25 month is going to come in the house for months, as anyone who knows, let's start coming to see for the 1.25 month.
12. Is/was there any change in the interpersonal relationships between you and your spouse and other family members after the event?
Single mother husband is not alive.
13. How did the family members support you and your wife during the difficult phase?
A lot of people supported the mother-in-law, two in-laws are also related to the discussion, the most attachment is to the mother-in-law.

14. What were the reactions/ counselling and suggestions given by relatives/neighbours/ community after the episode?
Yes, they should have been taken there, it is better than that, they should not have been taken to that hospital it does not carry in it. Many people had said that K Hospital is very good about the child, so I thought let's take it there. If they go, they give glucose in the jaundice whereas glucose does not go up in the jaundice.
15. Do you think and/or discuss with your spouse/ family member(s) about the death and related events and blame someone/something or feel guilty for the same?
No, I do not believe anyone guilty, I only believe my destiny, his life was so long, whom should I blame, doctors also do a lot on their behalf so that no patient is spoiled by our hands.
16. When did father and mother (if working) return to work after the death of the child?
Nothing to do in 1.25 month tomorrow will complete 3 week.
17. How you and your family's life has changed after the death of the child?
No, nothing to change
Knowing cause of death in detail- Autopsy and MITS
18. What was the cause of death of your child and what is your perception about the value of knowing the cause?

No I don't know I never had heard

19. In your view, what more could have been done to know the exact cause of death and the reasons causing the disease?

<about PM>

This is like taking out the stuff as soon as the eye is done, this heart is there, it takes out, it is in it like someone went to an accident and got hanged, takes them out.

20. Are you aware of any method for identifying the exact cause of death?

21. What are your views about the autopsy/ post-mortem for identifying the cause of death?

<necessity>

It is very important to know the reason, to come forward, if someone feels fever, then be careful before taking medicines quickly.

<religious aspect>

is wrong, by the way, people say it is wrong, even if we know why, we just say that we should not do post mortem, yes in our religion this is not to do post mortem. There is nothing written that people have said so.

22. What are your views about collection of the tissue (like done for biopsy to diagnose diseases) and body fluid samples for identifying the cause of death?

He is right by tearing, and then he is okay with tearing

<religious aspect>

No, it is not forbidden. People say things should not be torn. They say that a dead person should not give too much trouble, everything comes out, what goes on living in it, the person has left his life what can he do now.

23. How parents and family members can be approached for the tissue and fluid sampling?

If you want to say that we have to do this work then they cannot refuse, yes those people are unhappy at that time, but you will say at that time that they have to do this work, they will not refuse to say that, even on time they cannot speak.

<whom should approach>

Now I was sent down as if I was not there, I was a brother-in-law, now you could ask them that if we have to do this, they do not say a little or do not do it.

<method of explanation>

They will understand from the point of view, then they will tell from this that they will know what causes the child to get the test done, those who understand will understand it even if they do not understand Do it.

24. In your view, what factors are important for acceptance of the tissue sampling by the parents and family members?

Reason to know

Decision making dynamics

25. In routine day to day operation who takes decision about the major activities (purchases, treatment, spending money) about the family?

Decision taken by mother-in-law and father-in-law

26. During the hospital stay who discussed with the doctors/ nurses to make the decision(s) about treatment and other procedures.

In the hospital, the child had tau and there was uncle and I was there he was doing.

27. After death of your child, who decided about the cremation and other post-death activities and rituals?

My mother-in-law was the rest and it was as if the relatives were there, they called, bring clothes, then clothe them, then they took the mother-in-law, she was telling them.

Summarization

28. Now, we are completing the interaction/interview. Would you like to add anything?

My second son is older than that. He was younger with whom the incident happened. He was with him (son of sister-in-law). It was one day older than this. It has a birth date of 12, it is the birth of eleven dates, By the way, I was very fond of it because my elder one is also a little naughty he was so quiet that he did not know that there is a child in the house, he was a child, he was very dear to the whole street. The street was beloved of the street the street people are also serious of listening A lot of people had reached to see that he came to the hospital, he was in such a serious condition, he used to have a bottle in his hand, then he used to go to the latrine on his own, then the toilet did not go to bed on his own, never did the bottle, hand it I was engaged in K Hospital for fifteen days, used to remain in hand, yet went to the toilet Many children do latrine bathroom on the bed but they did not. His liver had stopped working, the doctor said, even if the jaundice will be cured, it will not be right, the liver was damaged due to the jaundice liver is such a thing that there is no other reason.

In-depth Interviews- Parents of deceased children-9

**1.1**	**Primary respondent (relation with the child)**	Mother
**1.2**	**Age of the primary respondent**	Mother-32 years
**1.3**	**Other member present**	Mother's brother

Father of the child was not present during the interview as he went to dispensary for taking treatment for his cough and fever. Elder son came in the middle of the interview and answered to some questions. And other three children went to school.

2.1 Can you please let us know about the illness and events that led to death of your child?

Mother- Our child has been ill, good enough to study in school, is five years old, has written names in school, had written in Jai Bharati. When he went to study, he suddenly went to play when he came home at 6-7 pm in the evening time, and then there is a slight fever, so there is a child doctor in Badarpur taken to the doctor in the morning. They have been given medicine, it has been relaxed. Then there was rest in the night and next morning fever start, then for the second time, his (father), our father, our elder child took his brother, looked at him, then gave him medicine . Then there was rest to taking medicine. Then on the third day, there was nothing to eat or drink nothing to eat, he tell him for water, I was getting more water, then that day he drink more water, he drink water all night. Be it, after drinking water vomiting started Then he went to the doctor on the third day from where he had come to bring medicines, so the doctor said that admit in S Hospital, he ran away from the same has been taken to S Hospital, then admitted at three o'clock in the day, admitted in emergency Have to take, then he give glucose water, then the elder son is asking for water to drink, then the doctor again agreed that do not give water, offered the bottle, hence he had considered water Then broke again after the water was about to end when half the bottle was finished, then they would vomit the blood, in front of the doctor show them to the doctor, some such disease is not there, we said that there is no such disease. In the same hospital, get all the x-rays all checked, because of which blood is there, some disease has not come out. last overnight and in the morning died 10.30 am.

Hospitalization period

3 Now we would request you to tell us something about the period of hospitalization for your child?

What did the doctor tell you about what was happening, when you recruit, tell me something> Mother - I kept asking them, what is the disease we said that it is not some disease. Then the slip was shown from where they were taken, to the doctor who had taken it, they saw it, and then they said that no one is right, to ask that TB disease is such a disease, not such a disease, nothing, (badarpur doctor) said, take it quickly, bring it to the hospital

<Who went to the hospital> elder child we Just like when he arrived there at three o'clock in the emergency, the doctor took him. He was the first private doctor

4 Could you please describe your overall experience about the hospital care?

<investigations, treatment given>

There were tests done (S Hospital), gave medicines in the same time, the medicine was given in pulse water in the needle, in the same. No medicines were brought from outside.

<cost of care>

Mother- Now I do not know this boy is known, I don't know.

5 Could you please describe your experience about communication by the various hospital staffs during hospital stay?

Mother- Now I do not know the name.

<who communicated>

There was a madam like you, just like a sister was a madam, she used to come and see.

<frequency of communication>

Yes, they used to come on time. <Those who understood you, they used to understand about the condition of the child> as they were taken in the ICU, they have shifted to the ICU at 3 o'clock at night, meaning where the child ward they have been in S Hospital. Where on the other side, where it is all, we went to the hospital first, but we did not go to the hospital first.
So the compounder of x-ray done stayed with him in the night, after taking him (x-ray), then he was a madam in the ICU and then he give a medicine, which means he says comfort. Then show up in the morning, the same are admitted in the ICU, so they should take care of all, how is the child not in the same bed. Did not tell us anything about what came in the report of x-ray.
Death and post death period
6 Could you please tell us about the death and death declaration of your child?
Mother- Madam, like you, they did not speak to us, they said to their child. We became nervous like this, did not get in front, the child kept on the bed, the boy is lying like we are sitting, we know as if our hands have become cold, so we started asking his father to go to the doctor. Ask what do you do, feet and feet are getting cold, some of them did not say Yes, I saw that she was sitting, it was getting cold with her hands and feet, call the doctor and see, he said (doctor) will break it later, the doctor will say, a madam Then the teacher who came like you looked after the voice, asked the voice, then as soon as the break came, after 10 o'clock, as his father was out of the room, so he said where will he find his father, call him, Call him, see the doctor and see that he is no longer your boy. Yes, I was there at that time.
<Give reason>
No, they did not tell us, gently told their (child) father in the ear, something was told, we have not heard so much in his father's ear.
7 What was your and your family member reaction to the death of your child?
Mother- My condition was very bad, wept on the bed, she was all over the sister, the gates were the ladies, they said, now what in the world is no more than crying, it became silent, so nervous as if we were the same Will die, and cried a lot.
<Your elder son was with you>
The elder son has not been together, when he has called, he has left the house, at that time there was only our husband, and no one was just both of us.
<Fathers reaction>
His condition also worsened.
8 How was your experience about the procedure after death till handover of the body to you?
Mother- Yes, it was one o'clock or maybe three o'clock by the time I came by car after we leave from there.
<How it took so much time>
kept the same, you have to read something written, so I will go away with the words, you have not said, do not have to take it like this, I had given a small slip, I do not know where we have kept it now. He said to show this slip outside, without going without showing, to show this slip outside.
<time taken for documentation and paper work>
I was late all the relatives of the house have brought everyone along with son. Took time. Time was spent in the hospital itself, was discharged late. We left and ran into the phone at home, we stayed there and our husband was there. Bewildered in getting up and sitting. How can we call the boy then, everyone has come and brought it back.
9 Who all from your family and community/neighbourhood were present at the hospital and how they supported you during this period?
Mother-elder son reached there, our nephew also arrived, and brother in law boy also came and stayed there and our husband.
10 What all rituals and procedures did you follow after death of your child?
Mother - Then brought it home.
<rituals>
We do it like we do, after Asir, 6-7 pm, go to body then buried. All relatives had come, all the relatives of had come, and more relatives came.

The bathing has been done by all the ladies. Many men had gone for burial, they had gone from outside to home.
<timings>
buried at 7 o'clock, followed by Asir followed by Azan buried after giving, Namaz went after recitation.
<rituals after death>
The third day is done like this, then forty-fourth, 40 months after one month, 1.25 month approximately, we eat food.

11 How did you, your spouse (wife/husband) and immediate family members cope with the difficult phase?

Mother - What to do now, now slowly have to be forgotten. Now recite Namaz to divert attention from it.

12 Is/was there any change in the interpersonal relationships between you and your spouse and other family members after the event?

Mother- No, not like this, there is nothing to fight. Our boys support us, all children give courage. He who passed away was the youngest.

13 How did the family members support you and your wife during the difficult phase?

Mother - nothing, speak silently, give courage, now you will not be able to cry because of crying, you will have to forget it slowly.

14 What were the reactions/ counselling and suggestions given by the relatives/neighbours/community after the episode?

15 Do you think and/or discuss with your spouse/ family member(s) about the death and related events and blame someone/something or feel guilty for the same?

Mother- We do not blame anyone. Now who is to blame, here everyone is a foreigner, everyone in his house is the same, who is to blame.
<No deficiency in treatment>
As the health suddenly worsened, at 6 o'clock in the morning, I went to the doctor, both husband and son.

16 When did father and mother (if working) return to work after the death of the child?

Mother - Our husbands do not work right now. <This happened after this incident or already> No, work was going down before this incident, and after which even does not work. Now age is over. <Started household work> Now you do it slowly, lethargy remains by breaking it. It took a quarter of a month, 40 days to mean like this.

17 How you and your family's life have changed after the death of the child?

Mother- Did not change, there are four such children, one elder brother, three more, now what to do, they are taking care of them.

Knowing cause of death in detail- Autopsy and MITS

18 What was the cause of death of your child and what is your perception about the value of knowing the cause?

Mother- Now someone said something, the house is all the same, someone is to be blamed, no one can be blamed, the playing child is gone, there was no illness at all, no injury was there. .
<willingness to know detailed cause of death
You should not be accused of being accused, but when you know that something has been done, it will not be known that you cannot treat anyone without asking, without seeing them, you are home. Or someone's outsiders see Bigger won't accuse you.
What do you think is the reason why you were playing suddenly, came from school, did not play in the evening, nor did all the children go to play in the evening like 6 o'clock in the evening, how did they come to the game, they broke the hunger was light, then took it in the morning His father, the elder boy, showed that he was ill and then gave medicine.

19 In your view, what more could have been done to know the exact cause of death and the reasons causing the disease?

Mother- How to know now, something would have happened now. Now they think for the child, they sit down while working, mind does not work.
20 Are you aware of any method for identifying the exact cause of death?
Mother- No, we have no idea about it. Now there is no what to do
21 What are your views about the autopsy/ post-mortem for identifying the cause of death?
Mother - for the post Mortem there, we said that we have agreed there, the child does not have disease, so we do not do post mortem
<who told you to do post mortem>
In the same hospital, there was a big madam there and said, "Take away the post mortem, then take it to do, then we said that we are a small child, we will not get it done, we have agreed." When there is no disease, the disease is nothing else, then let us take it, give us a slip do not know where the slip is kept, do not know where it is kept.
<why did you refuse to conduct post mortem of your child >
When there is no disease. Now I am nervous, have brought a small child. Nothing was detected when there was no illness
<any religious barrier>
We do not have a vein, nor do we have the same reason. Don't do it like a little kid now
<do denied because of some religious barrier or any other reason>
Now we don't care what the reason is, we don't know now
22 What are your views about collection of the tissue (like done for biopsy to diagnose diseases) and body fluid samples for identifying the cause of death?
Mother - they was tied to the bag by the doctor, the doctor said, how much has the water made, he offered water. So we asked the doctor who wants to put the bag of cloth, why the cloth, said that as much as we have offered water, now we have to see how much has been poured out and how much, the bag is all over How many bottles of water have been given and how much time can they have to finish and how to put the bags.
<Explained about MITS procedure>
Did not say anything. Just like the child had finished the holiday leave at 10 o'clock, we were calling each one, it was time, the doctor said not to write the first slip, so his father wrote the slip, now he is not getting the slip, Have been kept somewhere but forgot, do not keep it anywhere, you have not received the slip, the slip
<Have you agreed about MITS>
His father and boy are not ours
Elder son - Initially four blood samples were taken, and after that when he was admitted, three more bottles came out, the total 7 samples came out, and the test was also normal. When that four bottles were added, they were normal. <What do you think could be the cause of death> may cause overdose of injections, before that there was no Sirius Before I admitted myself, I was in line in emergency, five people were ahead of me, and I would have been before them if the conditions were more serious, but I had to wait and go from my turn. First of all was injected in her, then checked with hungry hand, then saw it is hungry then injected her, after 15-20 seconds of injecting she started bleeding. There was nothing before that. From here you will see that there is such a long route, reaching there, the bond with serious condition can be even more serious, but nothing like this happens. And the slip of medicine we were giving him was also kept, we also showed him the slip before being injected, then he saw it and injected it.
Elder son came during the interview.
Elder son- <Were you, when your brother died> Yes, I was there, morning time was at 9.10 minutes. <What the doctor told you> was not such a particular disease, I took him to sit on the bike himself, if there was a serious condition, he would not even sit on the bike, because here (from their home at prahladpur) S Hospital falls far away from And do not know how to travel on a bike. There was a normal hunger then first injected after the injection the blood started to vomit which was out of control, then increased blood, glucose, then he was in serious condition and then he Was put in another ward from the

Emergency. The hands that had to give us oxygen, had to shift from emergency when the emergency is shifted when the condition comes to normal, it was out of control and then it was put out in that ward, room number. 18 are probably, there was a shift when the condition was not even normal and we had to give oxygen, pump ourselves. Because that was not the machine it was at that time.
< he did not tell>
Elder son- No, he did not tell anything. He started his work as soon as death took place. The pipe, which was set up, left all the oxygen, they immediately wrapped it and wrapped it and said, "Death is a fire, you can carry it, you said it clearly."
<Who was told that death has happened>
My father, as I was entering, my sister got a call and was talking to him, then the father came right away saying that the doctor is saying that he is no more I said go and look in the room, when I went to see that they were wrapping it.
<The Post had spoken for Mortem>
No he had said nothing. Mother - Did not say like that, he was speaking to someone. Elder son- Just said that death is over, you can take it. She gave the sheet to wrap, did not say anything to post mortem.
23 How parents and family members can be approached for the tissue and fluid sampling?
<who, when should approach>
Elder son- Someone would have understood there was no one there. As soon as the Death is spoken, well, let's see what the problem was due to but no one said anything as soon as the Death, within 5 minutes, the pipe came out of oxygen quickly, immediately to the bed. Everyone took to cleaning. Nothing was given to any sensation. Said you broke it quickly and there are other patients too. Immediately started to wrap
<religious aspect for MITS> Elder son- It has no idea, but if you want to know the reason, then it can allow.
24 In your view, what factors are important for acceptance of the tissue sampling by the parents and family members?
Elder son- At that time what did (tissue sampling) had to see what the region is what was the region of death. It was necessary to know the reason as it was not a serious condition to be killed normal fever occurs to everyone he had fever in one or two ways, and in three ways was assigned.
I say about direct treatment because before that even the normal doctor showed it, there was nothing like this, vomiting of blood. The private doctor had said that the hunger was overstated, it was not happening in the control, that is to say, in this way, we are giving the treatment, if the hunger gets up again it was then we told you so Said that take you to S Hospital, there will be proper treatment I then reached S Hospital at the same time at 12.30 pm at 1.30 pm at the doctor's and had him admitted at 2.28 minutes, I still remember the time.

Decision making dynamics
25 In routine day to day operation who takes decision about the major activities (purchases, treatment, spending money) about the family?
Mother - We all take care of ourselves, men give gifts from outside then I see the whole thing.
<Decision about treatment>
Asked what to do for treatment, he sees the elder child. Right now my husband does nothing. Used to stitch before, now does not How the expenses of the house goes Have just rented a room below, my son does not work right now, elder son is 21 years old.
26 During the hospital stay who discussed with the doctors/ nurses to make the decision(s) about treatment and other procedures.
Elder son- The doctor said, take S Hospital and I have seen and gone, so I said that it will be the best and on the spot I did not waste time and I came home and I was getting something 12.30 Picked up the bike and took it to sit. My father had taken the decision at that time he had said to take it. They were unable to carry the bike, then I went with the bike, they do not know how to ride a bike

27 After death of your child, who decided about the cremation and other post-death activities and rituals?

Elder son - At that time my father was and my elder father had a son, who did everything as the arrangement is and my elder mother was also telling me how the rules are followed. As soon as people came to know, they took leave then at 9 o'clock, when I was dead, I called my brother, brother, it is such a thing, first of all my elder brother came to my elder father's boy, then the younger one came again. And he was the brother who came home then we brought home then whatever happened to (big mother) bath.

<rituals after death>

As the death took place on August 4, this mourning lasted for 25 ways, in whose midst there was no such thing just as the death happens to eat food we could not get it done because Muharram in the middle In the middle, it is not done between the moon of Muharram, we have got it done by waiting till November 5. Eating food blossoms in it, 40 days when the child is completed, food is served again in the death of the child or in the death of anyone

28 Now, we are completing the interaction/interview. Would you like to add anything?

Elder son- It was like everyone was there so quickly, but did not tell the reason, meaning as death happens, it is said that take it away. You have to mention the reason, once you check all the reports will be normal, even in the four samples that went, all the reports were normal.

In-depth Interviews- Parents of deceased children- 10

**1.1**	**Primary respondent (relation with the child)**	Father, Mother
**1.2**	**Age of the primary respondent**	Mother-25-30 years<br>Father-35-40 years
**1.3**	**Other members present**	None

Context- father- Brother and sister are all in Delhi

2. Can you please let us know about the illness and events that led to death of your child?

The girl was treated privately, the papers were thrown private hospital, the girl was born in Madan Mohan on 6 February then after three months the girl became ill and suffered a cold, all her treatment. First got it done in private but it was not done properly then we took it to Madan mohan. I was admitted in the emergency, the girl was seven days then a hole in her heart came out. He transferred to S Hospital then the doctor did not go there from there. F Hospital On the side of Okhla we took him there, he had surgery for his heart, yes he had surgery in Fortis his treatment was done his wound was healed, I had to go to some work post due to which I had to go home, I have gone home so (wife) is telling me, I was not here but I came later but my wife's phone was gone that the girl will not survive is serious brought home from Fortis when she was cured. Then in her fourth month, four to five months was very fine, she had reverse diarrhoea, and nothing happened, then took her to the metro station. The money is fixed, it is good to see his child, the child is not of a big age, so he took it as if there was a number, he replied that look son, I am not taking any fee from you, but only taking medicines Let's see what we had to see because you are poor, we don't want to rob the poor So do it like this, take him to MM Hospital, otherwise take him to medical or S Hospital, your girl has become very serious, yet no one has told that the girl is serious, the girl is playing a lot, she had a good time. If there was vomiting, then Madan Mohan went to S Hospital dispensary and then admitted, then put him in the heating machine, the girl's hand had failed completely, then put a pipe in her mouth and put a pipe in the way of urine water bottle Yes, this is the matter of Madan Mohan then he directly transferred to S Hospital. He lived there for one day and one night, at 3 o'clock on the second night, the girl was dead, yes in S Hospital, one day remained one night and the other day. In the second night, the girl was finished, meaning it was a night for two days, in S Hospital, it was over at four o'clock in the morning, we had also asked for a bottle of blood it had been two to three hours in Madan Mohan, at 7 o'clock we had gone to S Hospital at 10:30. We had reached, had asked for ambulance to refer.

Hospitalization period

3. Now we would request you to tell us something about the period of hospitalization for your child?

<duration of stay>

Took there one day and one night, on the second night, the girl was dead at 3 o'clock, yes in S Hospital, one day there has been one night, on the second day, the second night, the girl has finished, meaning two days had been a night in S Hospital dead it at four in the morning.

4. Could you please describe your overall experience about the hospital care?

<about investigation>

Yes, blood test was done, x ray was done, urine test was done, the whole test was done, heart surgery was done, it was done in Fortis and there was also (S Hospital), yes there we too Every week I used to go to S Hospital and doctors used to call us every week, we used to go out at 6 in the morning.

<cost of care>

3500 rupees was deposited, then 1500 rupees of blood was deposited, then it was spent so much, 1500 because this needle will come from outside for the child, so at that time I had no husband, no brother and brother. Just my mother in law and I was So I told the doctor, look, we have no one, our mother-in-law is blind where you sit and leave her here, and if you do not get lost somewhere, then I gave the money, then do not take tension, we will come, then we came In front of us, who had taken 3500 rupees of blood, I had

given me a card of blood, the blood had come, the whole bottle had climbed so much that the whole had survived, could not climb, till then the girl died.
<quality of care>
See, we got it according to the mix. Ok, now look at your doctor and see for yourself that you have done so much, if your child is not there then what is wrong for us, I have shown the system right there, got what we said to someone (M). Our child has not been found, what will we complain to anyone now? Nowadays no doctor wants this (F). According to us, it was fine according to the mixed from the time the girl was admitted, the doctor started checking herself, then in the end he said, look, trust the person above, now you will be lucky if the girl will be saved. People do not do such a thing, do not do wrong. We said, now it is up to you, I thought it was right on one basis.
5. Could you please describe your experience about communication by the various hospital staffs during hospital stay?
<communication of HCP>
The conversation was fine, there were only two or three doctors right, but the rest of the doctor was all dumb, the boys (doctors) also came. Ask from the heart no matter what hurts, are you getting work money Let me talk to her, let me talk to her, I came to her speaking to her on the same day that my girl was also discharged, on the same day I came and said, See what pain is there from a mother's heart. You are not even married, I think, I came in this way, you are the doctor who is doing it or we have left it all, I have spoken like this, you think we are not educated, we have a thumb impression, I had spoken to three doctors, I told them Sir Madam, the child's breath is getting stuck once you see "those people started speaking" You have produced a unique baby " I got into an argument with two or three doctors (M). The condition in which the child's breath was getting stuck at that time could have happened (F). He was a doctor, but he was very poor, but he had given a lot of help to God so good wife should be happy that he did not lack anything in his life, he did not refer, he was a doctor of S Hospital but 100 was right in 80 percent (M). Many doctors used to talk well, got government jobs, got good money (F).
<information about child condition or treatment by doctors>
No, no one used to tell, just say that you have faith in the above, your child's condition has deteriorated a lot already, so I said that you have not taken care of yourself, so please refer me and we know to produce children Even if we put 60, 70, 80 thousand, then we will not mind, I even said this, do not say it cannot happen I had also asked, if you do not feel Sir, then you will refer me somewhere and we will see you there if there is a good doctor of your life so much that if your brain is not being used in a child, I have opened up Talked to the doctor, you tell me base Lesley let us out of the hospital, we are so tense, So this is what the doctor said, "No son, you do not tension your child is 80 percent, it will be fine, just do not remove oxygen from the mouth". When I went to admit, I had only asked what had happened, I was in reverse and had diarrhea and nothing had happened, what happened was the report came, it did not tell me anything, what is the disease of the girl, ask more. "You walk out, you answer so many questions the poor man can only ask and what can he do, nor can you debate or fight with you."We have also thought that if you produce children too, if you die thinking, then do not bury me in the house, but go to S Hospital," I will never go to the government.
<about nurses>
The nurse has it all for her own meaning, we have come so much that we feel that how the nurse talks that if it is time to see, then she is looking after two hours, many are talking Drinking coffee
<during round of doctors>
What did you say about the child? Well, it is not already well, and did anyone ever pick up the hair and see the eye? Just the ladies in the room were all getting sad, "Stop the doctor there I used to feel like I was so angry in the room "I was so angry, I had no place to put nurse dipping in the body, where they have not dipped on the two, they used two sticks together This side is being planted, the other is putting that side It was with the girl (M). You are also a doctor, you have to tell one by one that you have to put everything together, from one side to another, the nurse is lying there, not seeing what is happening to the baby, the girl's voice is not coming out, Her body had done such a thing, that girl kept looking at her, she used to keep on tearing the bus, I have come to sing the time we used to call her fondly when I used to say son

would hold my finger. My girl died, that means she did not let her drink water, in such a way that her thumb went in the mouth (Father weeping), she drove me out, dying my daughter (F). That is why I had to fight with her in the name of S Hospital as well. Do not call any doctor here (about us), the doctor also said that when the girl child was finished that your blood money was deposited. You take the money, I said my girl died, even the money was dead, we are poor Kothi is not passing in the bungalow. Came to the doctor's mouth, take this, hold your hospice, I don't want my baby gone, I don't want money, so much fight took place that day from the doctor

Death and post death period

6. Could you please tell us about the death and death declaration of your child?

<cause of death explained>

Did not tell anything, only the doctor did not turn white, he had put in the hospitals and had referred, said, ask for the car, so I said do not tear the car, do nothing, do your auto, take the girl from the auto and fire. I was sitting with the child, neither did I look at the child, nor did I see him, nor did I fall unconscious, I was seeing that nothing happened, I was talking to him a while ago. Then I blinked once I understood the girl was finished on the table I fell down immediately, what happened in front of you, how did you fall, then I bid sister, my girl was finished, then I said to the doctor, once the doctor went to see my baby girl, when the doctor came, now you are saying that you are at home Take away, we are currently off. They did not give any reason why the girl finished, those people used to come only and used to give needles, then the needle did not work then they went away.

<any sympathy from HCP>

No, nothing, that's why we hated S Hospital, three children were finished that day, and my last girl was finished in the same room. Our baby girl was in the seventh month of the seventh month.

7. What was your and your family member reaction to the death of your child?

<reaction of mother>

The doctor also said when the girl was finished that your money was deposited, take the money, I said my girl died, even the money was dead, we are poor, Kothi is not passing in the bungalow that my girl is dead My money also died, I did not want any money, I torn the paper and came to the doctor's mouth, grab it, hold your innards I do not want my baby gone, I do not want money, so much fight took place that day from the doctor. When I was with the child, neither did I look at the child, nor did I see her nor I fell unconscious, I was noticing that nothing happened, I was talking to her a while ago, then I was seeing the same I blinked the eye, I understood that the girl was over the table, I fell down immediately. What happened in front of me, how did you fall, then I bid sister my girl was finished.

8. How was your experience about the procedure after death till handover of the body to you?

<about documentation>

Yes, it was signed on paper, my sign no, my (husband) sign was taken

<time taken>

The girl was finished in half an hour, called us here, took our sign and said, take it and then do your auto, after taking the child home by auto, the girl was finished by 4 o'clock in the morning. There were fires, twenty minutes of 4 or even 1 minute were happening, I did not notice, no, they did not give anything, did not even give ambulance, we came auto reserve

<about cost>

No just sign Curry took the baby girl and came away. Whatever money has been taken before them, let alone see what has been taken and what has not been taken (f). 1500 rupees of injections and 3500 rupees of blood are close to you. Send them. I said no sir. No one is with me (M). Why did you spend so much money, why don't I lie to you, why do I lie? I am not going to lie It is a different thing to eat, but do not blame lies, we can hear what you have explained (F).

9. Who all from your family and community/neighbourhood were present at the hospital and how they supported you during this period?

When the girl was finished, all the people had come to the hospital at that time, our brother-in-law, Husband had all come, at that time and we had served both the people had reached the car with the motorcycle, all got out of the gate, Yes, we called and told that the child is no more (m). I did not get

angry, did not tell anyone anything that was the wife who spoke (F). I got angry with the fact that I am speaking then to the doctor that my baby girl is not surviving, please feel free to refuse and give it to someone else (M). At that time he said that you have a unique child, there is no answer from me (F).
My family was saying this, leave the son and trust the above person, what has to happen has happened, now what will be more than this.
<What type of support given>
All the people had come, all were there, yes everyone helped that even my baby girl does not have as much weight, her father is sitting in debt till today, I am filling up the money of the girl's debt It was not, my child would have survived, that wealth would have been in front of my eyes, 80-90 thousand, we had put it in the hole in his heart, this whole area is public, we husband wife had raised money on the 20 rupees for 10 days instead of money. It was cured, she used to play very well there was a reverse diarrhea (F).
Fortis was taken from Madan Mohan to the doctor, yes surgery was done there in free, medicines used to come from outside, they were expensive, one bottle of fifteen hundred rupees was so small, some two and a half thousand, some three thousand and three thousand Ka, used to go for medicine and neither bothered the mind, it was so expensive girl's.
Yes, I was able to serve what I had to offer, then later we are giving it to him, yes many people have given support, they say this, now we are forced to speak wrong, we will not get the money in the month of tax. Every month, we will give you tax slowly, then we will do it slowly, we have given the rest of the money and its 20 thousand rupees are saved, then we will give it slowly by tax in 2-4 months in this round (M).
Post death procedure and coping mechanism
10. What all rituals and procedures did you follow after death of your child?
I was taken from here it is near to Jamuna ji. I do not burn a small child Kalindi Kunj went from here to the car, talked there and dug a pit across the land and then buried it
Yes, he had asked for money there for Rs. 3100. Then I said I am a small child. we are very poor then we give 1600 and 1000 for vehicles (father).
<who prepared body>
My elder sister came from my house, there was little sister, there was Devrani, there was Devar, and the girl of our mother-in-law was fiery Was bathed.
11. How did you, your spouse (wife/husband) and immediate family members cope with the difficult phase?
We do not worship, etc. in people, it happens later (M). I tell them that they do not know (wife) as there are little girls nor they feed them, the rest is not there in us, they were fed after five days, as many girls as eleven twelve, as many as twelve little ones came Is (f). What to do now, we will not come back from crying Now four bottles of water have gone up in his affair, what can we do? The medicine is also running, this (live child) still cries, I told him today that the doctors will come and take Pucci today I said yes Babu is crying then (M). So we said that it is right to tell that the book is ok (F). She was the youngest, she is the eldest, then she is and she was the youngest
12. Is/was there any change in the interpersonal relationships between you and your spouse and other family members after the event?
After her departure, we did not believe it, but there was so much girl, every other third day, her box of 302 rupees used to come with milk, since the girl finished, she did not know what was there in that girl. , From the day the girl has gone, we do not know what disease has taken place, do everything, do something, something is happening, the girl is not getting out of her mind, it looks like here I am sitting, someone has taken out the bus to rotate, I feel like I will come from somewhere in a while. After that incident, we have formed a relationship, it is breaking up, I do not know what has happened. After that relationship which has gone, we feel strange in our house as if the girl has taken some time to visit me.
13. How did the family members support you and your wife during the difficult phase?

14. What were the reactions/ counselling and suggestions given by relatives/neighbours/ community after the episode?
This is what the sister-in-law neighbor is not able to do in today's time, so what is the service, you also know, listen, sister, if we have four paisa, neither does the world support and when a bread in the house If there is no flour, then the person does not come out and ask for bread flour for the child, nowadays it is time, sister, this God who has given me poverty, asks God not to give anyone.
15. Do you think and/or discuss with your spouse/ family member(s) about the death and related events and blame someone/something or feel guilty for the same?
No, I do not believe anyone who spoke to me, the people above said what I had written in my luck with me, now it is expected that those who are left are our two (about live child) which we have It is up to these children well, that only their names will be known, what to do, what to say more than this, it is so sensible not to run into anything. Now we poor man stays for 24 hours day and night, we come in the evening at 7-8 in the evening, then the children live in food, then there is tension in the morning that they have to do something in the morning tomorrow, look at the children in the morning Let's go, I leave at 6 in the morning, he comes at 7-8 in the evening.
16. When did father and mother (if working) return to work after the death of the child?
We could not go to work for a month, it used to cry here, it did not leave me, it still keeps sticking like this, we have to come again and again from work, we go so far, weeping so fast (wife) calls that this (live child) is not getting silent, now you had called, then I had come back from half a distance and was going to work, so you called me so I thought that you are so far away If you will stand then I will go (F) after half an hour.
17. How you and your family's life has changed after the death of the child?

Knowing cause of death in detail- Autopsy and MITS
18. What was the cause of death of your child and what is your perception about the value of knowing the cause?
sister, we said the same thing that we have not read enough to know all this, now that you have asked people, then you will tell that you will inform the number is near, tell us, we will not be able to tell more than this (F). According to your accounts, everyone is saying that there was a hole in the heart, put all this money and made all the things, when there is no child, then what will happen to them, what will they do because of their life You tell me, the girl remains alive, so let us also talk to you about it, brother, for this reason we need that what is there in the girl that the girl is not doing well, now that she has been dead, what has happened to her (M).
19. In your view, what more could have been done to know the exact cause of death and the reasons causing the disease?

20. Are you aware of any method for identifying the exact cause of death?
<about post mortem>
So much was known that the girl has had a heart surgery, so much was known that the girl has a hole in her heart, she cannot take deep breaths, and as the child has toothache if she has toothache They cannot reverse, what is the reason for this, the doctor did not tell me, we have said, look, Sir, if we feel bad, speak on your face or let me out of the hospital. What is the reason that there is weakness in the liver that there is a hole in the heart, we just knew that there is a hole in the heart. Just after his treatment was completed, he was given medication for three months to two and a half months.
No, I do not know about it. If we do post mortem according to people, do not do it in the right way or do not do it, according to us, it is very wrong to do post-mortem, after doing a post-mortem a person lost his life even on his death. Do not like it, then tear it apart, we only say that any government would have been formed, but not to do so was lost, so the post mortem is very useless.
21. What are your views about the autopsy/ post-mortem for identifying the cause of death?
<about post mortem>

Whatever is done in the post mortem, it is told that we also know this much, and we do not know much about it. Post mortem, see if a man is hanged and dies, he also has an accident, he also has a bullet, when the patient goes to the doctor, then the doctor says that this medicine was given to the patient. When the post mortem is done then there are many ways too. The whole body will be irritated, what it is, I have not heard much about it. According to me, all this is a useless thing, a person has gone poor by dying, if his body is made dead, then it is out of my mind, yes, he bites more. What is left in the post-mortem, Madam, who takes away the work of the body, we also know, what is left of it, we take money and suppress it. (F)
<religious aspect>
It has not happened in our family, look, we would have known so much, we have been talking about it, our father was over in childhood, I was younger then only the elder brother was finished, then all the younger sisters are younger. Brother, how have you grown up, then married everyone, have done everything

22. What are your views about collection of the tissue (like done for biopsy to diagnose diseases) and body fluid samples for identifying the cause of death?

<about MITS>
It will be known about this that if all his tests have been done then we will also know the disease, at least seventeen diseases of a human being live, who says we do not have TB, if we have all this disease We will not be able to live a little, we will not be able to walk and this is what the doctors say. (M) This is fine, Madam, it is okay with the injection, the account of the tearing is not good in my mind. (F)

23. How parents and family members can be approached for the tissue and fluid sampling?

<whom should approach>
It is as if what is to be believed that I am the father of the child, the mother of the child, can give permission that will be fine, then our understanding is that it is mixed with what you have said from our mind. The best thing is, because the chord which is the rag, it is the most useless, that poor child is already gone, after his death, whether he should be a child or a child. That sister is not right with our mind. At that time, if I had asked me, I would have agreed, I or our wife would have accepted that thing, if you had said that I would tear a child's trunk, I would not agree to it. So let’s not happen. (F)
<methods to be explained>
In the same way you have just talked, in the same way that you put in our mind at that time, we would have told you that it is okay sister you can check the injection with our child. (F)
<who should approach>
As if you are a big doctor, you understand that the way you are talking is explained in the same way, because the man is already hit by his gum then his mind gets mad. I do not come, it is very well explained with a good mind, "Brother, look, your child is gone we cannot give a child."
If you say this thing or you do another type of test, then you have got this injection in your brain that we will give you the opinion that my son is sick, do not tear your teeth if you can do the whole test by injection so use it "(F).

24. In your view, what factors are important for acceptance of the tissue sampling by the parents and family members?

I liked your only question that through which this girl was finished (to know the cause of death), which disease had got inside it, which, after so much treatment, my child did not get rest or benefit (F).

Decision making dynamics

25. In routine day to day operation who takes decision about the major activities (purchases, treatment, spending money) about the family?

I do all these things my elder brother was finished my father has expired. Most of the time, my madam takes care about the treatment. The bone also has a problem, its expenses are different, no job can do the work of weight, if an accident had fallen, fracture has occurred, then the same junk is lying out and it is spent on it.

26. During the hospital stay who discussed with the doctors/ nurses to make the decision(s) about treatment and other procedures

At that time there was no brother-in-law in the hospital, only I and my mother-in-law were there, I was the only one there, I used to do it.

27. After death of your child, who decided about the cremation and other post-death activities and rituals?

No one told me, I had it done by the hand of a younger brother there was a pandit, who provides the child, there in Kalindi Kunj (F).

Summarization

28. Now, we are completing the interaction/interview. Would you like to add anything?

What I asked, what you asked of us, we told, who knew anything (F). Like, if we want to ask you this, if God does not eat crispy if we produce other children, if the above God does not give the same dish to his God, if suddenly we have an affair with S Hospital, then in the company of a mother but such Must not be moving What happens is not a very difficult thing, which has not happened in the company of people (M). This poor man who has no hearing in the government hospital, if he does not go to take it or even the fever checker, he says in his purchase, if we can do this thing ourselves, then why should we go to the feet of people Not only will we do it, but not the thing which is known, the person who has knowledge, he will go to the same, why we can do the thing which we do not know, if we know about any work So you will speak to us Raju Bhaiya, we do not know about this, you know that you can do this work, which will be good, you will get it done. If we know how to do this, then we will definitely do this work to show fake, we do not listen at all in the hospital. (F) What do we have to do if we do not live even then you would also say that stop in the room and play what will be seen (M). I have been 3-4 times in Madan Mohan, will give a bullet for the waist, no check up, no nothing. It was also done by private X ray, expenses in private X ray again went to S Hospital, there would be an operation, at that time there were no children, I did not even have one. Whoever is in the private we will get medicine, they will be lying in the operation, they will not be able to sit, they will not be able to do any work will they eat? Is there a wrestler here? The old man was treated there on the first strip, at which time he did So in the first strip I got rest Had to walk a bit and started getting up, there was 4 bandage, after that there was no money, then did not get treatment, later daughter, we have to see our children again. (F).

In-depth Interviews- Parents of deceased children-11

**1.1**	**Primary respondent (relation with the child)**	Father
**1.2**	**Age of the primary respondent**	Father-25 years<br>Mother-20 years
**1.3**	**Other members present**	None

2.1 Can you please let us know about the illness and events that led to death of your child?
Mother- He was in ESI for a month, after that, after a month, he was not well, then he was discharged, then we brought baby home. Then after two days, he started having fever, again we took him to hospital, there he checked him, he has cough, then they kept for two days, then on the third day epileptic attack started coming, it had never come before.
Father- we stayed only for one night at S Hospital.
Hospitalization period
3 Now we would request you to tell us something about the period of hospitalization for your child?
Father- The child had pneumonia, cough had gathered in the chest.
<what doctor said to you in S Hospital>
Doctor, when we went to night, he got an X-ray done, got an ultrasound done and then he referred to the ward, then they were giving medicines , then in the morning his (child) temperature increased by the time I went to fetch the cannula, his breath got faster and then went away.
Grandmother- Child was 4 months old.
4 Could you please describe your overall experience about the hospital care?
Father- All the tests were done on the spot for the child. They got X-ray done, ultrasound done. His report was received then, after looking at it, they told us, all report was told to us. No money was spent in S Hospital.
5 Could you please describe your experience about communication by the various hospital staffs during hospital stay?
Father- how we can know about behaviour in one days and night time. They did not say anything while admitting.
<The condition of the child was told>
As soon as he went, he told that the condition of the child is bad, its lungs are drying up due to which its temperature is getting high, fever is coming, whatever is coming, they said that they would try and see. Before doctors visit the baby died. He died at 8 in the morning. We arrived at 11–12 at night.
<who was communicator>
There was a doctor in the Emergency, she was lady doctor. She was not a senior doctor, she was a junior doctor.
<nurse beaviour>
Father-There was no nurse there.
Mother- The nurse was there. We were in ward 18. The nurse was not coming in but was just sitting. In morning I felt child was having fever then she (nurse) said that if you come to get cannula then I will inject it and I will not put it like that. <You were told to get cannula from outside> no from where doctor used to get in ward.
Death and post death period
6 Could you please tell us about the death and death declaration of your child?
Father- I was there only. I came to know that it was on my lap, it had stopped breathing in my lap, I understood that is no longer there.
Mother- As he was playing earlier, I put the cloth on him to go and get insert the cannula. < Then you call the doctor > father- The doctor was going from there, it was time for duty, she was going, so I showed baby to her, then she pumped the baby chest then saw that his breathing was not coming, even after half an hour his breathing did not come. <any sympathy/empathy>Father- At that time, Madam told us that we tried but we could not save him, the child was ill since night because we were late enough to get him at this hospital. Your child was admitted in ESI for a month, you used have paid attention to this thing. If no improvement was seen you should have changed.

7 What was your and your family member reaction to the death of your child?
Father- I managed to control but baby mother condition had deteriorated. <did someone try to console you and your wife> Father- By then my aunt had arrived there, she handled my wife.
<Mother started crying during the interview>
8 How was your experience about the procedure after death till handover of the body to you?
Father- The paper work was all done in an hour. At that time, it did not cost anything. At that time, their behaviour was fine because the doctors had also come, 3-4 madam had come, so there was no difficulty at that time, they had made the papers on time. From my family mu aunt came.
9 Who all from your family and community/neighbourhood were present at the hospital and how they supported you during this period?
Father- I and my wife were just in the hospital at that time.
10 What all rituals and procedures did you follow after death of your child?
Father- First the child was brought to home and then from home, there is a cemetery in front of it, after going there, he has buried it. Yes, the child was bathed first after that he was buried.
<Rituals followed at home>
Father-child was bathed then wears the shroud and then burials at the cemetery were performed. Grandmother- even the child is small or grown funeral prayer is performed for everyone, Father – funeral prayer was performed.
<What time Azan was given>
Dadi- after the burial 40 steps backward moved then azan is given after burial at burial place. Father- ritual is followed for 40 days, it was for 40 days. <What happens at that time at home> Father- First, fourth day, then 10th day, 20th day and then 40th day is considered it is all done according to Quran.
11 How did you, your spouse (wife/husband) and immediate family members cope with the difficult phase?
Father- Now you will have to recover and neither can you sit in that thought. He was our first child.
<Mother started crying again when asked about coping>
12 Is/was there any change in the interpersonal relationships between you and your spouse and other family members after the event?
Father- There was no difference in our relationship after he left. I take care of her from the beginning, everyone also takes care of it. Grandma – we involve her in household work.
13 How did the family members support you and your wife during the difficult phase?

14 What were the reactions/ counselling and suggestions given by the relatives/neighbours/community after the episode?
Father: The family members say that what is going to be known, no one can stop it, everyone tried it completely, everyone spend day and night to save him.
<reaction of family members>
Father- they also says the same thing. Everyone says this, who can stop what going to happen, all suggest to wait and calm. No one said why you take baby to that hospital. I and my wife were both there (S Hospital) we had to decide where to take it and where not to take. I was with my wife and mother, only three of us were there in ESI hospital.
15 Do you think and/or discuss with your spouse/ family member(s) about the death and related events and blame someone/something or feel guilty for the same?
Father- we don't blame anyone. It is just that whatever carelessness has happened, it has been done from our side we have kept baby for so many days at ESI hospital.
<What was the treatment in ESI>
The treatment was running normal it was going on for the last one and a half months. They were saying child will become alright, there will be improvement, improvement was also happening, the child was getting right even he was getting normal at home. Then we had fever we again took him to ESI.
16 When did father and mother (if working) returned to work after the death of the child?
Father- I went to office ten days after all this happened.
<when did mother started doing household work>
Her sister came to help her she mostly do the household work, now my wife is also helping her.
17 How your and your family's life has changed after the death of the child?

Father- everyone still sitting in sorrow, what is the change.

Knowing cause of death in detail- Autopsy and MITS

18 What was the cause of death of your child and what is your perception about the value of knowing the cause?

Father- Yes, the reason was told by the doctor that the lungs have dried up due to its cough.

<Not wanting to know anything from the doctor>

Actually we already knew what the reason was, everything was told in ESI had already at time of discharge. They told us his windpipe is narrow he is premature as he was born in 7 months.

19 In your view, what more could have been done to know the exact cause of death and the reasons causing the disease?

Father-all things were already declared that what was the reason. The report was present there is nothing that they have kept secret. He told whole thing beforehand. Reports were all given.

20 Are you aware of any method for identifying the exact cause of death?

21 What are your views about the autopsy/ post-mortem for identifying the cause of death?

<views about post-mortem>

Father-post mortem is done when death is not identified it is done to know the reason. And it is also performed in claim cases, from which reason death happened, or whether doctor is not fooling, post mortem can happen for many reasons.

<religious aspect> Father- No there is no religious aspect it can be done. But we don't need this as we know everything.

22 What are your views about collection of the tissue (like done for biopsy to diagnose diseases) and body fluid samples for identifying the cause of death?

Father- No, we do not believe, because it is not needed, because we all knew it, there was no such secret. Father- No, we do not believe, because it is not needed, because we all knew it, there was no such secret.

23 How parents and family members can be approached for the tissue and fluid sampling?

<whom to approach>

Father- If something like this happens to someone else, then it should be to talk to the child's father about the technology, because the ladies are broke and continue to weep in sorrow to take such a decision at that time, because it is male who can decide for that.

<Who should approach>

Whoever is a senior doctor there, or who is available there, or whatever the person concerned is there.

<Mode of communication>

Can be understood only by talking, at that time no one will want to see a picture or video.

24 In your view, what factors are important for acceptance of the tissue sampling by the parents and family members?

Father- The reason may be that if the parents do not believe that they have done the treatment properly or not, baby may have some other disease and medicine is going on in that case. Correct treatment is given or not.

Decision making dynamics

25 In routine day to day operation who takes decision about the major activities (purchases, treatment, spending money) about the family?

Father- It is decided by my mother that what to do.

Mother- treatment decision is taken by my husband (child father).

26 During the hospital stay who discussed with the doctors/ nurses to make the decision(s) about treatment and other procedures.

Father- It was mine and my wife decision to take baby to S Hospital. My sister had some reference of one doctor at S Hospital we took their reference because they were not admitting directly because ESI next hospital is K Hospital there primary hospital was K Hospital so S Hospital was taking it. There is one doctor in charge who helped us in getting admitted at S Hospital.

27 After death of your child, who decided about the cremation and other post-death activities and rituals?

Father- I, my brother and my uncle performed the last rites. The decision of that time is taken by senior of the house. In our family my uncle friend was telling everything.

28 Now, we are completing the interaction/interview. Would you like to add anything?

Father- Yes, we knew that the reason is not very accurate many times, some people do not know what is happening or what is not happening, which medicine is going on, even when asked. Some people could not ask due to doctor behaviour. But nothing like this happened with me, all of things were told in S Hospital.

In-depth Interview- Parents of Deceased Child 12

**1.1**	**Primary respondent (relation with the child)**	Mother
**1.2**	**Age of the primary respondent**	Mother-25-30 years<br>Father-30-35 years
**1.3**	**Other members present during the interaction**	Sister in law

Context- child's mother and child's chachi was present during the interview, the mother told that the child genetic sample has been taken for knowing the illness/cause of death in private hospital.

Events that led to death of the child

2. Can you please let us know about the illness and events that led to death of your child?

Mother-first child had fever sine March onwards till May then we showed him to local doctor, he was good doctor.

Sister-in-law- we first went to doctor L, DG, then to AKM and at last to A, all are private clinic.

Mother- he was admitted in L hospital for a week, at that time he was having swelling in stomach, doctor said there is no swelling, child has this type of stomach. Fever was getting normal and then again he was having fever. Doctor was also not able to understand what the problem was, then my father-in-law told doctor to give us advice, then doctor said he will have to continue medicine for long time. No investigation took place for stomach or for his disease, other test and ultrasound was done.

<What they said after admitting the child>

They said child is having pneumonia, fever, they gave him medicineand injection he got better after that they said now they will start treatment of stomach for that they conducted ultrasound for two times. They informed there is water in body, liver is enlarged. Then my father-in-law asked him for specialist, then he referred to G hospital. We went to Gangaram there they admitted my child. There they performed CT scan, endoscopy and other test including genetic test. They said there is no cure for the disease.

Aunty- there was some genetic problem.

Mother- I feed him serlax on 2nd October, on 19th he died, he was given syrup, it went to his throat, his face turned white, after that he started screaming, even had difficulty in breathing, after that doctor started treatment, then he was given injection, then his heart stop beating.

Aunty- he was given medicine in series when he was admitted.

Mother- after having medicine, he immediately had difficulty in breathing, before that my child was fine, he had swelling in stomach, then in gangaram he had swelling in hand-feet, when he was in ICU he was having swelling in head, his hair was also falling, doctor said there is no cure, we had spend a lot of money for his treatment and doctor was not telling what will be the prognosis of that. Then we took him to S Hospital. They didn't refer us to safadrjung hospital.

Hospitalization period

3. Now we would request you to tell us something about the period of hospitalization for your child?

<What happened at S Hospital>

Mother- On 10th of October we went in the morning at S Hospital, they did all the test etc.

Aunty- test were done there in the afternoon and then he died.

<duration of stay>

Mother- 8 days, when on 1st day they saw the child, they said the chance is less but we will try our best, they conducted several test, there was no ventilator, when his heart beat stopped for the 2nd time, then they brought the ventilator to the ward, they didn't start his medicines even he was not given food or milk. He was getting food at G hospital. They didn't start any medicine which he was getting at gangaram hospital they were only giving glucose, and at gangaram his heart beat stopped for atleast 4-5 times.

4. Could you please describe your overall experience about the hospital care?

<course of illness>

They didn't tell us whether it was improving or deteriorating, there were about 5-6 doctors including junior doctor, they used to talk among themselves in English, which we were not able to understand. So what did I not say about medicine, etc. I used to ask them again and again that what medicine is to

be given, when to feed they did not say anything, they used to listen after a long time, they did not pay attention.
<quality of care>
I did not like the behavior of staff there it seemed that where I have come, they used us to go here and there, behaved so wrongly that I did not like it someone is already in pain, so I did not like the behavior and treatment there.
<about investigation>
They didn't tell anything about test report, we used to bring the reports. They were keeping the reports with themselves, they used to note the report in diary, and they didn't tell anything to us.
5. Could you please describe your experience about communication by the various hospital staffs during hospital stay?
<about communication>
We used to take samples and we used to come after taking reports, they didn't tell anything to us. Everyday after 24 hours we used to get to know that he was not well, or his heart is stopping or not, then doctor used to say that his condition is not good, in betwwen nothing was told.
<Senior doctor used to say that>
The doctor used to come and say his condition was not good, they were having ambu, they were not having ventilators. The child suction was need to be performed, I used to clean, I asked twice to doctor that how to perform that, she told me once, and when I asked nurse about medicine then nurse said to give four medicines/tablets then my father-in-law said four tablets of yellow color it was brain medicine, they even started Gangaram medicine, we were not able to find one capsule, my father-in-law got angry and said what it meant to give four pills, they didn't know about the rule of giving medicine , doctor know how to give but they were not present. They know but they were not there at that time, the medicine I gave him came out of the mouth and then the same day doctor said that its condition is very bad.
<behaviour of nurse>
it was fine, they used to give them injection, what they were giving was not told to us, child had fever in between medicines were given every 4 hours. Facility didn't have thermometer, then I bought my own.
<cost>
Aunty- money was spent on the medicines. A lot of money was spent at Gangaram hospital.
Mother – a lot was spent in G hospital.
Father: we spend nearly 5 lakhs in Gangaram.
Mother: And he was already admitted.
<Satisfaction about treatment>
Father: S Hospital is a government hospital, there so many patients come many children come that's why children die because a lot of patient comes there.
<satisfaction with S Hospital >
Mother: No we are not satisfied. Father-no not satisfied.
Mother: After that, they did not even go to the child, did not even check how is it, what was it the nurse did not come to see.
Mother: Senior doctors used to tell something, junior doctors did not tell us anything.

Mother: I used to ask doctor about child condition but did not say that much, they used to say less hope is there child condition is very bad, and the expectation is very low, we are doing what we can do, She only spoke but she had not started the medicine.
Mother: Medication means that it was not started since the admission. He was getting medicine for his hert and breathing, no medicine was given realted to brain, and he was not feed.
Father: The child had gone there, he did not get a single feed for 10 days.
Mother: And his brain was fine in Gangaram, but when he went to S Hospital he got swelling and his condition had become very bad there.
<who were present from family>
My husband, my father-in-law, brother-in-law and wife was present with me.
Aunty: we were always present.
Death and post death period

6. Could you please tell us about the death and death declaration of your child?
<Who declared>
Mother-on 18 at 7:30 in evening his heart beat stopped, he was given medicine, I didn't know the rule of giving medicine I gave 1 tablet, so when I gave medicineit came out of his mouth, his breathing also stopped, my father-in-law was also there, doctor removed pipe from his nose, then I came out of there, after 10 minutes now he was fine, then my father-in-law told me to go, he stayed at night, it was last day in hospital.
<What was time of death>
Mother: I did not even know at 5:30 in the morning.
Mother- I slept at night and wake up at 5 in morning, I was there in hall.
Father: I was not there at night that day.
Mother: He was in the office
Father: The child also needed ICU, but ICU was not available.
Father: I brought the child home.
Mother: There was a lot of blood coming out of his mouth.
Mother- doctor were again and again changing his nose pipe due to which a lot of blood was coming out, I don't know whether it was changed in Gangaram or not, I don't know why they were changing frequently, whether it was needed or they were changing to teach others.
Mother- the pipe was also there for faecal matter, after that pipe was changed he was not doing toilet since then, earlier he used to excrete 500-700 grams.
Mother- they were not giving proper treatment and not taking proper care of the child.
7. What was your and your family member reaction to the death of your child?
Father- what reaction, you must know that will the reaction of the family.
8. How was your experience about the procedure after death till handover of the body to you?
<Time taken>
Father- they left us at 7.
<about documentation>
Paper work was going on they made me sign the paper.
<assiatance from staff>
They made us sign the paper, my father signed. I went there at that time but no support was given.
Mother- till last day, I didn't met the doctor and nurse, I directly went outside, at night at 7:45 I came out, then didn't went to child, the guard didn't allowed me to go inside.
Father- we were in ward no.18.
9. Who all from your family and community/neighbourhood were present at the hospital and how they supported you during this period?

Post death procedure and coping mechanism
10. What all rituals and procedures did you follow after death of your child?
Father: What happened we took the child for burial.
<bathing of body>
Father: No bathing took place
Mother: bathing happen in adults not in case of children.

Father: we went to 40 feet lane for burial.
<who came during burial>
All family member and neighbour came.
<who supported>
Father: There were family members and neighbours who were supporting us.
Father- we bury the child.
Mother: Since my child was born, progress was very good, laughing, speaking, playing means that there was no problem, even he understood.
Mother: child was delivered normally. He was 15 months old, then he had stomach swelling and fever due to these two things his condition worsened.
11. How did you, your spouse (wife/husband) and immediate family members cope with the difficult phase?

Father- no prayer happens in us.
12. Is/was there any change in the interpersonal relationships between you and your spouse and other family members after the event?
Father- there is no change and tension between us.
13. How did the family members support you and your wife during the difficult phase?
Father- relatives support financially, as a lot of money was spent in G hospital, at S Hospital no money was spent.
Father- all supported and consoled us.
Father- I went to work after 10 days.
14. What were the reactions/ counselling and suggestions given by relatives/neighbours/ community after the episode?
Father- some gave suggestion that you should have taken child to CP Jain some said to take at K hospital.
15. Do you think and/or discuss with your spouse/ family member(s) about the death and related events and blame someone/something or feel guilty for the same?
Father- i dont blame it is of no use, because at government hospitals doctors are less and patient are more, no nurse are there, there are more guards when compared to nurse, there is no system to work, in one ward four ward will be there, bu one nurse for all.
Mother- there is no cleanliness.
Father- treatment didnt stop it was continuing since 2 at night, but doctor didnt tell us clearly, 5-6 doctors saw his stomach all said it was normal , when it started swelling then some decision was taken.
Mother- first they said he is anemic.
16. When did father and mother (if working) return to work after the death of the child?

17. How your and your family's life has changed after the death of the child?

Knowing cause of death in detail- Autopsy and MITS
18. What was the cause of death of your child and what is your perception about the value of knowing the cause?
Father: I have given this to get the test done, to know the reason. (do you know the reason for death)
Father: Yes-yes (you are saying genetic test)
Father: I have given it to test (report has not come yet)
Father- child DNA test was to be done.
Mother- the sample is given at G hospital, after that his condition was bad, so we decided to do test later.
Father- yes we want to know the result of the test.
Mother- we wanted to know what and how it happened
Father: person had probably just gone to Bangalore, we got a call for the test, just two days ago, 24 thousand is asking for that test.
Father- yes I would like to go for that test.

Father- in those 24 thousand two tests will be conducted.
Mother- the doctor told there is 30-40% chance that this condition can occur in next pregnancy also.
19. In your view, what more could have been done to know the exact cause of death and the reasons causing the disease?

20. Are you aware of any method for identifying the exact cause of death?

21. What are your views about the autopsy/ post-mortem for identifying the cause of death?
Father: post-mortem did not happen they had spoken for the test then.
Mother: Post mortem only happens in accident, etc.
<religious barrier>
Father: No, there is nothing like that, there is no religious barrier.

Father: Those people had not given any such suggestion, the doctors asked whether it was necessary or not.
Father- I know about post-mortem
Mother- we didn’t face any situation like this earlier, that’s why didn’t know about it.
Father- there is cutting of the body, this much knowledge i have.
Father- this (MITS) was not done.
Father- i would have done, if someone had told about this.
Mother- this would be helpful for future.
Father- we would have known the reason.
Father- we would have known cause of death, it will be helpful for future pregnancy.
Mother- we would made clear, that we would keep in future.
22. What are your views about collection of the tissue (like done for biopsy to diagnose diseases) and body fluid samples for identifying the cause of death?

23. How parents and family members can be approached for the tissue and fluid sampling?
<who should approach>
father-if doctor would have told us we would have agreed about that.
<how to explain>
Father- this doctor could tell better.
Mother: As the condition of our child was very bad, we didn’t have power to think what to do next, should we take to other hospital, whether other hospital would take the child on seeing its condition. It was very difficult to think what to do.
24. In your view, what factors are important for acceptance of the tissue sampling by the parents and family members?

Decision making dynamics
25. In routine day to day operation who takes decision about the major activities (purchases, treatment, spending money) about the family?
Mother- my father-in-law takes decision regarding purchase and treatment.
Aunty- father-in-law takes the decision.
26. During the hospital stay who discussed with the doctors/ nurses to make the decision(s) about treatment and other procedures.
Father-in-law was present, but I used to get medicines.
27. After death of your child, who decided about the cremation and other post-death activities and rituals?
Father- my father was present at that time, he was taking all decisions.

Summarization
28. Now, we are completing the interaction/interview. Would you like to add anything?
Father- I just want to say that S hospital is not for serious patints, for serious child private hospital is fine, at all places there is issue of money, that’s why people go to S Hospital, if the child is saved at S Hospital he would always need someone support. They were not telling the truth about his treatment. Also in gangaram they were not telling the true picture 1% also, if it was less then they used to tell child condition was very bad. They were telling 10 diseases with one disease. When they were discharging, they were telling that everything of the baby has been corrected the kidney is working normal the child is passing urine, normalizing everything and telling that everything was healing.

Mother: very helpless people go there, when they give up, the same people go there and wise people do not go, and most of the time I have seen all the patients who have lost the same people. Everyone at this hospital who came said that I have come from this hospital, I have come from that hospital, I felt so much there, so what to do, I had to come here at last and the reason for our arrival was that the doctor at gangaram said that there is no cure for your child condition if we could have got treatment there we would have stayed there.

Mother: My child in S Hospital was 24 hours without ventilator, in beginning he was on ventilator but when they saw that his pulse is all right, the breath is right then the doctor there decided to remove from ventilator. But after removing it, when it was removed, it remained for 24 hours, then left it the same way, child was shifted to the ward the next day, then his heart stopped beating again, then if there was a need for ventilator then somehow My father-in-law has got it done by speaking and we needed ICU but no ICU was available. Due to unavailability of ICU he could get right medicine. In ward only 1 medicine was given through syringe so that his heart could beat.

In-depth Interview- Parents of Deceased Child 13

**1.1**	**Primary respondent (relation with the child)**	Father, Mother
**1.2**	**Age of the primary respondent**	Mother-35-40 years<br>Father-40-45 years
**1.3**	**Other members present**	None

During interview father and mother of the child were present. The child was first admitted to DD Hospital, there he stayed for one month then he was referred to S hospital, were he stayed for only one day.

2.1 Can you please let us know about the illness and events that led to death of your child?

Father: We have the nearest DD Hospital, our child was 22 days, he got loose motion, went to loose motion that he has lost motion, he is doing lettering again and again, but the lettering was not water- Is doing water, then said what was fed that the milk was fed upstairs, said that at this time we cannot give anything, we will not give medicine, just drink mother's milk, we said that we are not drinking mother's milk. Do whatever you say, just drink mother's milk, immediately sent us no medicine, no treatment.

<Did they refer to S Hospital>

No, it was 22 days. Then DD Hospital went to say that its diarrhea is not going to stop, feed him the same mother's milk and we have nothing, come, then we have taken him here. That the child is breathing fast, said that you have to do it quickly, you would like to do it in a nursing home or in DD Hospital, we said that it will not be done in DD Hospital. Will not, he wrote on the slip, took 500 rupees Said, go to S Hospital, take it and see it immediately. <Did not admit in DD Hospital > No, first kept a month, when I moved here from the nursing home to DD Hospital, then they kept admitting for a month, the baby did not gain weight, and the weight of the baby kept going down, just Just understand that there is a lack of water, glucose has to be offered. I said, offer it, glucose is given, then it is said that it has got pneumonia inside the hospital, said that now the pneumonia has worsened. I said that you are a doctor, you will treat us, we will do whatever is happening inside the hospital, neither do you, then we are watching all those tests, these tests, they did those tests when the baby was born So 2 kg was 800 grams, if discharged after a month, 2 kg was 400 grams, the weight lost, there was no significant success, he said leave, show it in OPD, bring it home On the 26th of the night, when I came, the child started breathing very fast. I said, what is the matter, I took advice from my friends and said, DD Hospital, I will give you rest, I said, I will take it in the morning, DD Hospital, after taking a shower in the morning, I said that such a condition of the child is happening, now they started saying that it was just 10 days ago. , How is it deteriorating now, he said that there is no blood in the body, we said that you have discharged, you should know what to do with the child, say take the child to S Hospital, the child will not be treated here.

Hospitalization period

3 Now we would request you to tell us something about the period of hospitalization for your child?

Father: Yes, on 27th we were taken from here, S Hospital directly, said, don't even ambulance, don't waste time for ambulance. Yes, (said in DD Hospital) the child was breathing very hard, we were not even drinking milk, doing nothing, taken to S Hospital, to say directly, the child is not breathing, no blood in the body of the child Turned yellow I said, "Let's talk with us, how will we take the ambulance? No, S Hospital is taken, then how are the children sent by the DD Hospital having so much problem, how did they send it without oxygen?"

Father: Yes, I went straight to the emergency, said why sent it, it was to be sent in an ambulance with oxygen, I said what can we do now as we said we took the auto and came, they admitted it and that pipe in mouth and nose Put, put pipe in place of bathroom, put syringe in hand, will have to say blood, then blood is given, blood got blood in a long time, some blood was given Run the pump separately, we will give breath, the pump was kept that two, keep pressing it, we kept giving it, then it said that the condition is very bad, we cannot say anything and there is no space in the ICU, I said no space in it If so, what will

you do? We will do something, refer you, say we cannot do anything we can send it to the ward. Yes (ward no. 18). They sent him to the ward and his health worsened. And the memes there are so many doctors, that doctor was speaking very diligently, she is thin, she smokes, one will stay alive, if you want to be treated, then do not make me do it. How to do a Jana, the child is also crying, they left me out of fear, as much as it happened, they sent me to Lao Medical to get the test done, to do the blood test in AIIMS, go there, I have been running all night, this night of 27 I was so elated Yes (27 at night). It was midday on the 27th, on the go, then on 28th morning I got run away, take this test, take that test, in the last I did not give my madam test, telling me that this test has not come, I said that we have given, we are not giving the report, they are saying, report two, we will cure today, no matter how we will treat, they said, last injection was given, and now there is no chance, child Sit near There is no chance now that the Madam child has died in front of us, meaning that we have wandered in DD Hospital from the beginning, you will cure the child who has lost motion, I will fix the government hospital which is open for what you can cure. It is not enough to drink mother's milk it will be okay, baby, drink mother's milk. I said, what should I do if mother's milk is not there?
<Medicine to mother for milk production> not given (no medicine given).
Mother-vitamin syrup was given
Father- Just, given a vitamin-D to the child later to give it yellow, but there is no improvement in the baby, the child was born very healthy. <Normal delivery was> No operation. It was on full time The child is up, but the child corrected in the operation. Now what can I say carelessly, <You stayed for one day in S Hospital > Yes, 27 was gone, the child was finished on 28th. Morning at 1:10, no-no in the afternoon.
4 Could you please describe your overall experience about the hospital care?
Father: Now used to send me again and again to get these tests done, now go to the new building now go to AIIMS. <Did you get AIIMS report> I had got the report. Those who were not telling anything, those who came in the report were telling the doctor (S Hospital).
<cost of care>
What medicine was given? They kept the pipe pulses, but nothing else, they used to put the injections.
5 Could you please describe your experience about communication by the various hospital staffs during hospital stay?
Father: Nobody tells you, (Doctors used to tell whether someone is improving or not), the condition is bad. Yes, he used to say that.
<behaviour of doctor>
The ward who was 18, the doctor was so filthy, he was thin, he was very dirty, I did not see any such ladies, witchcraft, did not talk in a manner whose child is about to die. You have no way of talking
<nurse behaviour>
Was the same for everyone No one was there, one or two nurses who were she was fine, but no one else, there is a very bitter doctor, there are number one. Did not say anything, did not say anything.
Death and post death period
6 Could you please tell us about the death and death declaration of your child?
<death declaration>
Father: I called the doctor and brought the child was not breathing, then all the doctors were coming, I feel that its breath has stopped, yes it is coming, it is just now, I said where it is, now She is crying and I am crying here, I feel that when I am giving a pump, the baby's stomach is bloating.
Mother crying during interview
Father: <because of the pump> yes sir. Means put the pipe on it.
Mother: There was bleeding from the mouth. I brought that blood is coming, they come and speak, we cannot leave the pump, you call, then we came to say that the blood is coming from the mouth of the child, so what did they get out of the mouth? , A lot of blood was coming.
<when did they told that child is no more> Father: 1:10 pm 12:30 pm He finds out that the child has swollen.
Father showed the video of the child.

Father: There was a lot of trouble with this. <who declared> was a junior doctor, he did not give time of 1:10, he means heart failure due to lack of blood, I said when we were undergoing treatment at DD Hospital, he did not treat it properly, as much blood was not formed. You are leaving so much.
Mother: Saying that the heart has failed due to anaemia.
Father: Yes, the blood drained in S Hospital too (the blood spilled in S Hospital too), the syringe that was put there turned blue at that place, meaning that they are joking like this. Yes, they used to say that who had given it away.
Mother: Blood was extracted 4-5 times; after every half an hour the blood came out, the whole hand turned blue.
Father: From where he had to offer blood, as if it was a joke for him, how much blood was flowing, how he was moving in such a fun way, I am telling you about the emergency, there.
7 What was your and your family member reaction to the death of your child?
Father: There was no one doing, how they were handling each other at that time.
Mother: No one was there, no doctor nor nurse, just kept crying and carrying my baby, my brother came.
Father: What would we have done while falling there? Later at the last time, when he was leaving, his brother came then from there we did auto, brought the auto here.
8 How was your experience about the procedure after death till handover of the body to you?
Father: Nothing, baby is over, take it.
<any reason of death>
There was no blood in the body, the heart failed so that's all. <No time or any problem in signing> No, let's say, it's done soon, not that stop, go quickly.
9 Who all from your family and community/neighbourhood were present at the hospital and how they supported you during this period?
Father: We were both just, yes. Now used to send me again and again to get these tests done, now go to the new building now go to AIIMS.
10 What all rituals and procedures did you follow after death of your child?
Father: He had brought home (where did you take it), everyone had come and kept it.
Mother: Saying that take away nothing can happen now.
<rituals>
Father: Nothing but sitting and wearing clothes, took him to the crematorium. The child was not bathed.
Mother: My father and everyone came and then took them. The children who came to are buried, they do not burn.
Father showing slip which he got from S Hospital
Father: Only I got it from the hospital, yes to get the child out and when the death certificate later. It is near Mayapuri (burial place), Delhi Cantt is located nearby.
<who all came>
We were father-in-law, our father-in-law, our brother-in-law, everyone were.
<rituals after death>
Father: Nothing happens. Mourning is still going on in our house, now when we see the photo in the phone, weeping starts.
11 How did you, your spouse (wife/husband) and immediate family members cope with the difficult phase?
Father: Daughter, now she will play at home, stay with the children, then take some rest in the day, then go out in the evening, and I come at 10 o'clock at night, never mind, and called If you know me, then I will come, I will see.
12 Is/was there any change in the interpersonal relationships between you and your spouse and other family members after the event?
Father: No, nothing just handles each other and pays attention to the child.
13 How did the family members support you and your wife during the difficult phase?
Father: What did you think, whose person he took, he says, it was not yours, comfort you, one to the other.

14 What were the reactions/ counselling and suggestions given by the relatives/neighbours/community after the episode?

15 Do you think and/or discuss with your spouse/ family member(s) about the death and related events and blame someone/something or feel guilty for the same?

Father: Here (child mother) used to tell me that he had taken DD Hospital - DD Hospital, could not show in private, said that DD Hospital Government Hospital is where the baby was born, everything happened, but the doctors here were negligent with him. , When we are running again and again.

Mother: They took out all the blood, when the blood was completely working, Jake gave it to me, and I was saying, Sir, the doctor himself said that there is a lack of blood, even if you want to offer blood, you can offer They were blood, I was saying there is a shortage of blood, then do something, saying that it will be right before you take yellow milk, it will be correct, if you wanted to offer blood, you could have given blood.

Father: Yes, when the delivery took place, they took a bottle of blood, they did not even offer it,

Mother: Did not even offer me, saying that there is no need, and that child should also be discharged, not offered, if he could have offered it, it is said as soon as it comes that everything is right.

<do you blame someone>

Father: I obey DD Hospital if I had stopped his diarrhoea at the first time, it might not have happened today. No, the treatment is not good there it is not good at all.

Mother: Everything is done due to DD Hospital negligence, whatever has happened, nothing happens if the child is not finished

Father: He was very careless that even when I am asking you, tell me the situation of your child, yes, yes, it is fine.

Father: Yes, you kept one month, the weight of the baby is not increasing, then you will see, I will see, if I had to see me, why would I bring it to the DD Hospital hospital, why did you feed me upstairs, I Said we fed the milk upstairs, when the mother is not doing, then you are telling me, what to do if the baby is crying, let it die hungry, we are also asking you what to do if mother's milk is not happening Give

Mother: When I came from there, I was drinking my own.

Father: It was very short, the baby's stomach was not even filling, it was a gap of seven years, the mother's milk did not increase in the middle, we said that if milk is not happening then what should we do in that mother's milk Just drink, oh tell me how to drink, yes, give some medicine, otherwise you give me milk outside, give some powder, so that the baby's stomach is full, not mother's milk.

Mother: He had said that give some medicine, etc., that milk starts descending, no such medicine is said, just correct your food and medicine, nothing happens. No, even after the operation, I had not been offered blood, they were right.

Father: Took blood, when you will give blood to the treatment, when you take the blood and do not offer it to the child, nor offer it to the mother of the child and they are saying that the blood has died.

Mother: But these people, after doing tests all over the world, sucked all the blood of the child out of the blood, his body is white, the photos are all there, the lack of blood is from here, do not test the surplus funds Nothing happens.

Father: Now when you are saying that you are not happening, some kind of hospital, Lady Harding would refer

Mother: Even the bones were taken from the back to reach the water but then they are saying that it is weak, so could not come out.

Father: Now there is no place in the ICU, when there is no place in the ICU, then you see that the condition of the child is worse, the child will be kept in the ICU and sent to the ward, three beds on one bed, yes, S Hospital In the |

Father: The situation is same here in DD Hospital, three times on one bed.

Mother: Not three, five, five.

16 When did father and mother (if working) return to work after the death of the child?

Father: I started ten days later. I was three months, two and a half months with the rest of the child, when the child was finished, I used to run away with DD Hospital on the next day, kept the admissions here for a month, then the child was on holidays, my work was very little at that time. Ever since the child was born, even when it was over, my duty was not complete, ten days in a month, fifteen days went just like that and my private job is there, now the owner has told the problem what to do if the child is admitted.
Mother: Even four-five times before pregnancy.
Father: Even before pregnancy, diabetes has happened four or five times, we have to administer more sugar, if not, we will do the cleaning.
Mother: Initially, not before but not after, then started insulin.
Father: Yes, coming home and injecting.
Mother: Used to come home.

17 How your and your family's life has changed after the death of the child?

Father: What is the change, that is what happened to the rest of the boy then I am happy that the girl is a boy, it was a boy after seven years, after seven years.

Knowing cause of death in detail- Autopsy and MITS

18 What was the cause of death of your child and what is your perception about the value of knowing the cause?

<any other reason apart from heart-fail>
Father: Yes, everyone said that the heart has failed.
<willingness to know the detailed cause>
Now what do I want to mean, I tried a lot, everyone tried to understand a lot, I myself was in depression so much depression, that I myself had to work after working , Bp I was high I have been admitted to DD Hospital, it is a matter of fact that only last week it is said that the doctor said that he is taking tension, yes he is taking tension, BP medicine said that you are taking too much tension, my hands and feet work Stopped, said this is happening due to tension, said you stop thinking more, now son is finished how are you saying that you forget, no one can understand ours

19 In your view, what more could have been done to know the exact cause of death and the reasons causing the disease?

Father: Now to find out the cause of death. If there is an accident, then even if some say what is there about the child.

20 Are you aware of any method for identifying the exact cause of death?

21 What are your views about the autopsy/ post-mortem for identifying the cause of death?

Father: Yes, it is true that if there has been a death in the hospital, the child who has taken a lot and if anything ends, then the post-mortem should be done, no matter what is said, now what will be known when you press the matter inside. Inside, the doctors suppressed this matter.
<religious aspect> No, there is nothing like this in our religion

22 What are your views about collection of the tissue (like done for biopsy to diagnose diseases) and body fluid samples for identifying the cause of death?

Father: No, nobody told me anything about the investigation.
Mother: Samples were taken from the bathroom, but there was no test HOA.
Father: No, nothing was taken after death.
Mother: Samples were taken earlier but nothing later, yes for treatment, all this did not happen.
Father: Yes, yes, I would have agreed if someone told me to do it.
Mother: I went there that day, the dispensary was saying Madame, bring some paper, etc., we should find out who did what, what happened, suddenly the child was absolutely right, you got some paper I said, Madam did not get anything.
Father: Here is the dispensary behind.
Mother: She was saying that it seems that suddenly it happened that this happened to you, it was over, then the child was right, healthy, you brought it that day, I said Madam, we do not know that blood Heart failure due to lack of

23 How parents and family members can be approached for the tissue and fluid sampling?

<who should approach> Father: Someone would have given us advice about this, we had not told it.
Mother: Did not tell at that time, if you told, no one came.
Father: It is like, we had controlled a lot on myself, now look, we were still crying, we had kept a lot of care to each other, what I can do now, speaks to us also that you can know the reason, we take blood and blood from here, then we would agree, yes, because it is over.
<method of explaining MITS>
understand only by saying, yes.
24 In your view, what factors are important for acceptance of the tissue sampling by the parents and family members?
Father: No, the treatment is not good there it is not good at all.
Mother: Everything is happening due to DD Hospital negligence, whatever has happened, if not the finished child, nothing happens.
Decision making dynamics
25 In routine day to day operation who takes decision about the major activities (purchases, treatment, spending money) about the family?
Father: Who is the decision to cook the house, is a wife.
<treatment decision>
remains mine
26 During the hospital stay who discussed with the doctors/ nurses to make the decision(s) about treatment and other procedures.
Father: The decision of both of us was whatever the hospital was.
27 After death of your child, who decided about the cremation and other post-death activities and rituals?
Father: The one who was a Pandit there. Give that milk like this to two children give milk pulses, salt pulses.
<from your family>
was my mother.
28 Now, we are completing the interaction/interview. Would you like to add anything?
Father: What will be the solution?
Mother: We also want to know what has happened, but now when there is no evidence, it cannot happen now
Father: They have time to talk on the phone, they have a head, see the children coming and coming, in S Hospital, it was the same situation, you are busy making fun of each other, no one is watching the children. Because your job is sure, you will get salary, if someone dies, it was like a joke for them.
Mother: And when the vein could not be found, there used to be a place somewhere, saying that the vein is not being found, to apply the cannula, even the whole body was lying blue, here in S Hospital saying that A cannula has to be applied on the forehead, saying that the vein is not being found, the whole is lying blue, said to get treatment, I said that it is done in DD Hospital sir, saying that it has a breath block, it was applied in the foot. Negligence was the biggest thing they did they just strayed and took them to S Hospital.
Father: I said write and give it to the referrer, saying that you should tell me that the child is being like this. Did not write it. I was discharged ten days ago, from here we were at home, I went to Emergency, I said, look at the child's condition, that day was Saturday, 28th was Saturday, I was off The day I was at home, I will not take it, I will come to check once, I went straight to the emergency, then he said, do not waste time, take it to S Hospital.
Mother: Just spent the whole night talking, crying, not drinking milk, mouth being removed again and again, disturbed the whole night, then went on like this on the second day, then DD Hospital sent people to S Hospital.

List of In-depth Interviews of parents of deceased neonates

S. No.	ID	Age	Religion
1	Parents 1	Mother- 20-25 years<br>Father-25-30 years	Muslim
2	Parents 2	Mother- 25-30 years<br>Father-3-35 years	Hindu
3	Parents 3	Mother- 25-30 years<br>Father-30-35 years	Hindu
4	Parents 4	Mother-30-35 years<br>Father-30-35 years	Hindu
5	Parents 5	Mother-25-30 years<br>Father-25-30 years	Hindu
6	Parents 6	Mother-20-25 years<br>Father-25-30 years	Hindu
7	Parents 7	Mother-35-40 years<br>Father-40-45 years	Hindu
8	Parents 8	Mother- 20-25 years<br>Father- 25-30 years	Hindu
9	Parents 9	Mother-20-25 years<br>Father-20-25 years	Muslim
10	Parents 10	Mother-18-20 years<br>Father-20-25 years	Muslim
11	Parents 11	Mother- 18-20 years<br>Father- 25-30 years	Hindu
12	Parents 12	Mother-30-35 years<br>Father-30-35 years	Hindu

In-depth Interview parents of deceased neonate-1

**1.1**	**Primary respondent (relation with the child)**	Mother, Father
**1.2**	**Age of the primary respondent**	Mother- 20-25 years<br>Father-25-30 years
**1.6**	**Other members present during the interaction**	Nani, Maternal uncle (mama of child)

2.1 Where do you usually go or who do you consult during your child's illness?
Father- We goes to the doctor.
Nani- She is our daughter, she stays in Muradnagar, and she gave birth to her child there. And for treatment she was referred to Meerut.
Father- We goes to doctor in 12 extensions it is 1km away, he is good MBBS doctor.
2.2 Where do you usually go or who do you consult to vaccinate your child?
Father- There is Kendra, we go there.
2.3 People in your area / community are generally asked for their children's illness?
Father- The people here goes to government centre where doctor sits it is 5 minutes away from this place
2.4 Mention the reasons for priority for the facility and / or healthcare provider.
Father- When child suffers from cold and cough, any other problem we go to that doctor only. For vaccination also of mother and child we go there only. The behavior of doctor and nurse is good. There is treatment for elder as well as for younger one, medicine is also available.
3.1 Can you please tell us about the illness and events that caused <name> your child to die?
Mother- The doctor only told that meconium has entered and nothing else. I was admitted in private hospital in Meerut, I was alright and I was normal, only they told meconium has entered, they kept my child for 2-3 days under machine, when the condition got worse my brother told me to get admitted at S Hospital.
Father- Then, after 6 days this happened (Death of child).
Mama- In Meerut only, they told us that the child will not survive, we took the risk and brought the child to S Hospital. We go to S Hospital only, there we get good facility and they don't take money. Then, I brought him to S hospital, he was alive for 5 days in hospital and then died on 6th day.
First of all, in M hospital they took a lot of money from us, it is a private hospital.
Father- in M hospital, they didn't show the child to us, so how could we know whether the child was ok or he is breathing or not.
Mama to father of child- You must be having the report of the child they are asking you where the fault did happened.
Mami- The expected date of delivery went 3-4 days above the date mentioned in the ultrasound. The movement of child was not felt in the womb, less movement was there, then she (Child mother) started having problem, then she was admitted in Meerut, there she got treated and her doctor refer there only.
Nani- Then after keeping her whole night, they told delivery will happen through big operation, because of meconium.
Mother- Rate of big operation was high, but it was necessary, so they delivered by baby through normal delivery performing little operation.
3.2 Who supported you during illness and your child's hospitalization?
Mother- When the child was admitted my whole family was there, my brother, his wife, my mother, my father, all were there, my in-law's family was also there, all people were together.
4.1 Now you are requested to tell about what should happen when your child is admitted in the hospital? (Investigation: hospital, ward, length of stay, course of illness)

Father- Everything was fine in S hospital, when ever there was difficulty in child breathing, we would call someone they used to come, they tried their best, but they already told in emergency that condition of your child is critical. Doctor told us about the serious condition of the child.
Nani- They kept telling us that child is serious.
Father- Doctor kept telling us that child is serious, and chance of survival is less, they told clearly that there is only 1% chance.
Mama- Chance is less, it was told in S Hospital as well in Meerut.
Father- Cleanliness was there, but there was problem from guard side.
Nani- They cared for our baby, when we used to call them, they didn't delay for 1 minute, they used to come.
Mama- We got a lot of facility in S hospital. For 5 days he was on oxygen, we used to pump by hand, for this his father, my mother, my father was present for 24 hours, because oxygen was coming through pumping by hand, machine was not there, so we were giving by hand. The baby also got better, for 3 days he was well, he also opened his eyes, even breathed, we also have video of the child.
Nani- We used to pump by rotation, three people used to stay in hospital. One of the reports even came correct, and other was not ok, they kept telling the condition of the child as serious.
Mama- The child was suffering from a lot of problems, firstly meconium entered, one of the tubes was there on child, one in mind, this was only told by doctor, and second problem reported by doctor was that blood is deposited in the chest, and they told openly that chance of survival is only 1%.
Nani- They were saying, he is surviving for these days otherwise he may survive for 2 hours only.
Mama- The investigations done were good, for the blood examination we used to go with syringe in new building, whether it was day or night all investigations were done. The investigation was done moderately as it was necessary. I also take my child to S hospital, they do a lot of investigation, they did all checkups, and performed all investigations.
We are satisfied with treatment, we also tried from our side, but we could not save him.
Nani- Yes, doctor tried to save the child, and we also tried but when luck is bad so what can we do.
Mama- They did not change the tube on the last time of the child, which was in child mouth it was filled with garbage, they couldn't change that.
Father- Together, four tubes were changed, there his condition started deteriorating, garbage was there in the tube.
Nani- For draining water out from his mind one tube was there, the clear water was drained from his nose.
Father- The tube which was put for breathing, in that garbage was there and some blood was coming out, the child condition got worst when four tubes were changed.
They were coming and checking the condition. They changed the tube 4 times, once the heart had stopped working altogether, there was little carelessness, they used to make mistakes 2% out of 100%.
Nani- No, the doctor used to give attention to the child. The doctor looked after our child.
Mama- Doctor should stay at this time, because at this any time he may be needed.
Father- No, expenditure was there in S hospital, we spend money in U.P. hospital.
Mama- No money was spent for investigations, only they told us to bring 4 injections from outside, otherwise no money was spent.

4.3 Can you describe your experience about communication by various hospital staff during your hospital stay?

Mama- There behavior was fine, doctor used to see the child, check the child and used to tell about the condition of the child that it is not good. The senior doctor used to come twice, one at 9 o'clock in the morning, then between 3-4 o'clock, after that other used to come (who sits in room meant for doctor and nurse), medicines was given three times, injection was also given, the water which was given to the child, in that also they used to give medicines 2-4 times, blood transfusion was also done.
Father- Firstly, doctor used to talk to each other, then they used to tell us, we used to understand what they told us, the other who used to accompany senior doctor also used to tell us.
<MOTHER CRYING>
<nurse behavior>

Father- The nurse was good in the hospital, she used to come before the medicine got finished and give medicines 3-4 times, and when we needed any help, then we used to call her.
<supporting staff>
Father- There was fine, but why we need to talk to them, they were okay.
<guard>
Mama- What we should say about guard, they have habit in every hospital that they allow only one person, for us two people were allowed. All gents were there, two used to stay inside and other stayed outside. Whenever, we used to see that cleaners are coming then we left the place ourselves.
5.1 Can you please tell us about your child's death and death declaration? (Investigation: Who had announced the death, body language and expression prior to the events before death)
Father- When I was pumping, then there was no movement in stomach, heartbeat was there, I put my hand over his heart to see heartbeat. My younger brother was with me I told him to call doctor, then she came and told us that he is no more. <who told you> Junior doctor who sits in the cabin told, he checked again for our satisfaction. Then senior doctor came, who comes in the morning. Doctor told us he is no more then, they were talking among themselves, then I called my brother to come quickly to hospital.
Mama- As we felt, the condition of the child is bad, then doctor came, and checked and then told your child is dead, then after 5 minutes senior doctor came.
Father- Senior doctor were not there, they were called from the cabin, then they came and told that heart has stopped working.
Doctor stayed there for 1-2 minutes, and then left then my brother came and he saw the file. Before this incidence, the tube was changed. The nurse did not say anything about the child.
5.2 Do you blame someone / something for the death of your child?
Father- No, we do not blame anyone. To say, U.P. doctor told us that operation will take place, I had gone to deposit money for blood as they told blood will be needed, and when I came back I got to know that there was a normal delivery.
Mother- U.P. doctor forcibly performed the normal delivery, because of which child had the problem. There were six doctor, they all were trainee doctor, no senior doctor was there, they forcibly performed the delivery, because of which child health got worse, and he didn’t cry. Neither they told me I have delivered girl or a boy, did not say anything.
Mama- We got discharged her from U.P. hospital after 4-5 days and thought that now we will show her to S hospital.
Mother- When the U.P. doctor told us that big operation will take place, they were also preparing themselves for that, they even took blood from 2-4 persons, they didn’t even transfuse the blood to me, the time which my husband took to deposit the money, by that time they did the normal delivery. There who had undergone big operation, there child was with them and who have undergone small operation their child was not with them.
Mama- Many complaints were told of U.P. hospital.
5.3 How was your experience about the process after death until the operation of the body?
Mama- After death, I reached hospital in half an hour were they gave the slip and told that you can take the child. Then, at night we took the child to U.P. by van with our own money, as hospital do not provide ambulance, it is only for Delhi and we buried the child in morning 10-11.
Father- They provided us the slip which is needed for death certificate, it was provided us my taking signature, the nurse took the signature.
5.4 Who supported your family and everyone in the community / neighborhood during this period?
Mama- Only gents were there in the hospital at time of death, the child father cried, everyone knew it was god will. Ladies were not there. My sister health was not good, only child father was there with the child, his grandfather and one brother were present, then we took the child in night and came.
<staff sympathy>
Mama- Everyone is busy in the hospital they resumed their work after telling about child death, as many more children were having some problem as our child, even 5-6 children like our child died before the

death of our child. Our child was lucky, that he survived for some days. He even opened his eyes, started getting better, before that he did not cry, did not even breathe, then he even started breathing.
Mama- Doctor already told that blood is deposited in the chest, this was told 2-3 times, we are doing treatment but he can die anytime, this thing was told in both hospital (S Hospital and U.P Hospital).
6.1 What procedures did you follow after your child's death?
Mami- Azaan, was given to child in his ear.
Mama- After birth of the child, Azaan is given whether the child is girl or boy, as in your religion Gayatari Mantr is recited. In hospital we did not performed any ritual, just brought and buried him. Next day Fatima is performed, for that Quran Sharif is read.
Father- At 8.30 PM the child died.
<time taken after death>
Mama- We two brother took the bike and went to hospital. It took an hour to reach hospital.
Nani- These two brothers, brought him.
<cost>
Mama- The ambulance which we hired, only money was spent there, otherwise no money was spent. This is good in S Hospital. Rs 2500, was taken by ambulance for carrying him to U.P.
6.2 How did you, your wife and family members face a difficult phase?
Father- Nothing was done, I resumed to work after 3 days.
Mama- You should ask this to mother, father/men resumes to work, mother is upset more as she kept child for 9 months in the womb. Still, she has not overcome out the grief.
<mother crying>
General talk
Mama- The government should put more restriction on private hospital as they try to rob poor man, they write something else, tell something else. Private hospitals are more problematic. We also faced problem due to private hospital, the centre government should make pressure against them
6.3 How did family members support you and your wife during difficult times? (Investigation: burial / cremation rituals, community norms / rituals, phase, coping with financials)
Mama- Everyone supported us. At 10 o'clock in the morning next day he was buried, we left the hospital at 10 o'clock in the night, and at 1 o'clock we reached the house (U.P.). The guests came in the night. All the relatives arrived. The baby is bathed and buried; anyone can bathe the child, whether the person is adult or a child, he is bathed, then wrapped in a white shroud, a fragrance is put on it and then kept. All this was done in the morning. Everyone went to cemetery from our side as well as from their side, Namaz, the prayer is read. Afterwards, the prayer is read for the person, so that god apologize him, whether the person is big or small prayer is performed, as the child has not done any sin, he is pure from sin.
7.1 What was the reason for your child's death and what is your perception about the value of knowing the cause? (Investigation: knowledge about the cause of death, a desire to know the cause of death, potential factors that cause illness / death, possible effects on other family members or the next pregnancy)
Father- The reason was already known, they told reason in starting only, and told only 1% chance is there for survival, and they were trying to save. It is important to know the reason, we did not ask the doctor for the reason, due to which he died.
7.3 Are you aware of any method to identify the exact cause of death?
Father- We does not know about post mortem, this is done in accident. We can know the reason after talking to doctor.
Mama- Yes, we know about post mortem, organ donation is done, it is done after accident, from government side, it is that they take kidney and seal the body later. In today time, no attention is given to post mortem. There is no post mortem for natural death. If the person suffered from fever whether he was younger or older, the ritual is done, if the person died due to accident or murder, then postmortem is done. The report is then checked to know, who killed, how did he kill, whose finger print are there. The children who died in hospital, there post mortem is not necessary. When someone complaints to the police, when

there is doubt whether the person died himself or someone killed by murder, then it is sent for post mortem, or the police take itself.

7.4 What are your thoughts about autopsy / post-mortem to identify the cause of death?

Mama- In our religion post mortem is not done, we don't like that our near and dear ones should undergo tearing process. And if it is necessary, then centre government says post mortem is done for accident or murder, to check how did it happen. If you perform post mortem then you will know the reason of death. But we do not like that the person who is known to us should undergo post mortem a everything is removed in it.

Nani- Everything is taken out in post mortem, whether it is good bone it is taken out, and we also don't know who is the person/what is his caste who will touch the dead body, we do not want that wrong person hands should be put on our known person.

Mama- In accident or murder, post mortem is necessary, if the police got to know then even if we try to stop then also they will do. Police can take the dead out of cemetery if it is needed, they check report how the murder was done.

Father- It is not mentioned anywhere in our religion that post mortem cannot be done.

7.5 What are your thoughts about the collection of tissue and body fluid samples to identify the cause of death?

Mama- This technique is right and good, it is good then tearing, the body which have undergone post mortem nobody likes to see it. I have watched post mortem 3-4days back on YouTube, I did not like to watch it, they did post mortem after consuming alcohol. I have also read about post mortem in newspaper two-three months back. If this technique (tissue sampling) is done then it is good, it will be performed by machine. After doing this all rituals performed will remain same.

7.6 How can parents and family members be contacted for tissue sampling?

Mama- About this technique, if the doctor would tell to parents that child has died and they should now allow them to do this technique, then parents will get to know how the child died.

Mama- How much time is need to perform this technique? If it will consume less time then nobody will deny, if it will take time, after child death then family prepare to take the body. At government hospital these procedures take time. Our child was small, otherwise in government hospital a lot of time is taken. If doctor would tell about this new technique, that blood will be taken to tell us about the reason of the death, then nobody will deny.

<family approach>

Mama- We will try to tell anyone who will encounter this problem in front of us.

Nani- At that time to tell parents will not be the right thing as they are already in grief, so they can tell us about this.

Mama- Whoever is wise, you can tell them, if doctor will tell about this technique then no one will deny. We want to know how the child died, the reports would come, we will see the report, in today's time the report comes in the form of film, in post mortem these only things are revealed.

7.7 In your view, what factors are important for the acceptance of tissue samples by parents and family members?

Mama- Parents should be told comfortably about this thing (tissue sampling). Now the death has already happened, doctor checked, in todays time nobody wants to see the death of the child, as it has happened to us, doctor is engaged in their work as there are many children, there were 15 children in the ward where our child was kept. We liked the technique (tissue sampling), but the doctors can only tell whether blood will be taken or what will be done with syringe.

<any change in hospital>

Mama- More and more advertisements should be done, in hospital many things are written on the wall in Hindi and English. For this technique someone should be present to tell. Other than that, you and other officials can use their mind.

8.1 In your view, what can be done to improve children's efforts to reduce deaths and to identify the causes of death?

Mama- The doctor who is treating child for 4-5 days and if he has done some mistake then they will not try to do this procedure, as by doing this they will fear that their mistake will be known to the family and also, they would be suspended. The child has died because of their mistake as they didn't change his tube. Now report will tell about the cause, so they will be suspended, so they will not try to tell about procedure. Now this technique (MITS) is in your hands that parents should know about this procedure.
Nani- They were telling about the condition of our child, we our not telling anyone mistake.
Nani- Tell me what to do next, since then my daughter's health is bad. Her health is not improving, she is still in the shock, that why I have brought her here in my house. Now what has happened, it was only Allah's will.

4.2 What was your and your family member's reaction to the death of your child?
my wife didnt came to hospital, she was at home. i was there at hospital, doctor checked the baby infront of me. doctor said there is no movement of the baby, at that time i felt sad and felt sorry at that time.

5.3 Is/there any change in the interpersonal relationship between you and your spouse and other family members after the event
There is no effect in the relationship, just a little bit, now the wife is worried because of our child. I explain her not to worry. The baby was our 1st child, so we remember the child, sometimes she is also in shock it will take some time.

5.7 when did father/mother returned to work after the death of the child?
i returned to job after 15 days, my wife started household work after 40 days. Now she is little disturbed, but now she is cooking for the family.

5.8 How you and your family life changed after the death of the child?
Two or four things have changed since then, everyone says that there will be no other problem, do not take tension and the time for eating and drinking has changed. Previously we used to eat in the morning, now there is no fixed time. I do not feel like eating and drinking after that incident. So far, I do not feel hungry very much, now I have thought of the child further, but my wife is not in condition, she had swollen, now I am showing her to the doctor.

7.1 In routine day to day operation who takes decision about the major activities about the family?
for routine work decision are taken by my father. for other workdecision is taken by my paternal uncle.

7.2 During the hospital stay who discussed with doctors/nurses to make the decision about treatment and other procedures
when child was in hospital all decisions were taken by my father-in-law.

7.3 After death of your child, who decided about the cremation and other post-death activities and rituals
At that time my father and my brother-in-law was there, they were telling all things.

In-depth Interview parents of deceased neonate-2

**1.1**	**Primary respondent (relation with the child)**	Mother, Father
**1.2**	**Age of the primary respondent**	Mother- 25-30 years<br>Father-3-35 years
**1.3**	**Other members present**	None

Introduction
We are living with three members; mine, my wife and my daughter.
2.1 Where do you usually go or who do you consult during your child's illness?
Usually we go to MN hospital, but in serious case prefer to go S Hospital. My daughter was born at S Hospital.
2.2 Where do you usually go or who do you consult to vaccinate your child?
For immunization, we go to a dispensary at W-12, SAINIK FARM. We had plan last delivery at a private hospital as people says Govt. hospitals are not providing good services. I was scared from govt Hospital.
2.3 People in your area / community are generally asked for their children's illness?
Poor people usually prefer Dispensary for small disease. That is a govt. dispensary and cheap. But in case of serious disease, people go to govt. hospital, because we cannot afford costly treatment.
3.1 Can you please tell us about the illness and events that caused <name> your child to die?
Initially, baby was suffering cold and cough. After that, my sister said, he (Baby) might be infected and went for advice to a known Doctor at MN hospital. At there, Doctor gave a nasal drop and recalled after two days. Again we went after two days, doctor said, baby has breathing problem, Baby has not kidney, Heart and told about a lot of disease. After giving a nasal drop, doctor recall after two days. On next appointment, doctor declare the case as a serious and drop us with her car after insert a pipe (for breathing) into the baby nose. Then we went to S Hospital, at there, doctor refuse to admit. They said that we don’t have machine to manage this case, at same time they said, this baby is about to die. The hands and legs of baby was shivering at that time. At the time when my baby was at MN hospital, he was good and that doctor help us a lot as she refers in their own vehicle. A lady doctor (Jr Doctor) from MN hospital, had come with us to S Hospital for admission. She put (lay down) my baby with earlier admitted child (3-4). In hospital ward, I called for emergency help but no one came to see my child. At same day, in midnight around 12- 1 o’clock, my baby was dead. They discharge us at morning 6 o’clock. No one at hospital care us, everyone was busy on their phone.
3.2 Who supported you during illness and your child's hospitalization?
We went hospital with wife only, later on Mother-in-Law and sister-in-Law came at hospital for help.
4.1 Now you are requested to tell about what should happen when your child is admitted in the hospital? (Investigation: hospital, ward, length of stay, course of illness)
Mother: There was a big trolley on which, around 4-6 children were kept. All together moves to next (top) floor for treatment. All children were fitted with an automatic breathing machine but mine (children) has a manual breathing machine, I had to push that manually for maintain baby breathing. We kept pushing the machine overnight for same. It was continued from 8pm to 6am. At morning someone (Doctor) told us, this cylinder (breathing equipment) had last at earlier means there was no gas (Oxygen) in that. No one informed us about this. We wasted our time to push an empty cylinder overnight which has no gas (Oxygen). Everyone was engaged in their phone.
4.2 Can you describe your overall experience of hospital care? (Investigation: investigation, treatment delivered, satisfaction level, quality of care, cost of care)
There was not any investigation had been performed at S Hospital. Prior to that, on MN hospital all investigations had been done. It was X-Ray, Ultrasound and all blood test. At S Hospital, they only gave injections (1-2) over whole admission period at name of treatment.
Prior to admission, we bought Pipe and 2-3 medicines that were wasted later on. They (hospital Staff) demand money for a machine (Breathing tool), but we did not have money at that time. We spent all our savings even at time of delivery. We request them to use the machine and give some time for payment but they demand money before. We were unable to instantly arrange the money at night time (10pm-11pm).

4.3 Can you describe your experience about communication by various hospital staff during your hospital stay? (Investigation: everyone communicated, which was the primary communicator, nurse, doctor, other staff, frequency of communication)

It was good but Doctor was very rude. Nurse was doing their work properly but doctor did not come to ward just even for a walk.

No one detail/brief about what had happen. Nurse only inject 1-2 injections and glucose. It was frothing mouth and many difficulties had happened at that time. No one was looking for manage frothing mouth. Lastly I use to clean my baby mouth at regular interval. It was supposed to be cleaned by machine but in our case we were doing same.

There was one person as caretaker of all at our ward (Hospital). I hardly remember whether she was a nurse or doctor. He visits the ward only on call. His advice only to push the breathing equipment and concentrate on that. The person who had come with us from MN Hospital to admit, told that, make a OPD card of S hospital and you will get machine (Breathing machine). The hospital staff refuse to provide that machine even in front of all. He demands 12000 to get that machine.

Usually, A junior doctor updates the status of my child. Senior doctor told us, your baby required machine, get it soon. We said our problem that we cannot afford that. He said all available machine engaged in earlier admitted patient, its already scarcity of this machine. We give it to serious case only. We continue request that our case was also critical please provide us a machine.

We reached hospital around 7-8 pm and that person from MN hospital made an OPD card of my baby. She told us that you get machine after the OPD card but that not happened.

5.1 Can you please tell us about your child's death and death declaration? (Investigation: Who had announced the death, body language and expression prior to the events before death)

Mother: Can you please tell us about your child's death and death declaration? (Investigation: Who had announced the death, body language and expression prior to the events before death)

We told that our baby is no more, he died at mid night but no one listens me.

After our repeated request, a senior doctor came to see our baby. No one told us about the situation. Even after knowing that baby was dead at midnight, I could not understand why they were not released us from hospital. Lastly at morning, they discharge and declare dead.

5.2 Do you blame someone / something for the death of your child? (Probe: Who or what, what did you do in this context)

Mother: Yes, I would like to blame doctor who told that your child will dead even before starting the treatment. They advised machine for baby but not issue same. We tried to get the services from hospital but not got. We fought from a lady staff for service. She rudely behaves with us. Earlier my child was absolutely right but don't know what happened after.

5.3 How was your experience about the process after death until the operation of the body? (Investigation: procedures, documents and papers given, respect for religious norms, time taken, cost / payment, assistance from staff)

They gave me a slip we left all things like cloth etc. at there and came home with baby. It took 2 and half hours. They told to collect death certificate after 1- 1.5 month.

Junior Doctors care a bit more as compare to seniors. The senior doctor came at once and instruct to push the manual (breathing) machine in 1, 2, 3 sequence. After that they did not came. A lady with that senior doctor repeats the instruction.

5.4 Who supported your family and everyone in the community / neighbourhood during this period? (Investigation: who informed them, who all came, what kind of support was given)

We went hospital with wife only, later on Mother-in-Law and sister-in-Law came at hospital for help.

6.1 What procedures did you follow after your child's death? (Investigation: Preparation of body, which prepared, burial / cremation, other rituals and time)

(After death declare) We came to home by an auto. We totally lost about what was happening with us. We admit our child just 2 days back in a good condition. At MN hospital, they told it was just cough and will get well soon. And suddenly, they told us about lot of disease (kidney, liver). As he told baby has not kidney and liver but it was not informed at the time of birth. Whenever the hospital where birth had happened was a good hospital, it's VB Hospital near M hospital.

6.2 How did you, your wife and family members face a difficult phase?

We lost our job because of this. Now, we are unemployed and not got another job because of tension. We are not able to do work

6.3 How did family members support you and your wife during difficult times? (Investigation: burial / cremation rituals, community norms / rituals, phase, coping with financials)
I was there with my mother and since I am new to this area, we took the dead body to mother's area. A gays residing at top floor is an auto driver, was also with us. A crematorium is there. We went there. In case of children, we burial the body rather than funeral. Yes, first bath the body and wrapping the dead body, etc. are the process. We were done Havan (offering prayers to god in front of fire) after one week of death, same was done at birth time also. A prayer on the name of child was also happened, in which cloth, flowers, rosary has to be offer to god. Wife did not go at cremation place. Around 10-12 people were present there, who belongs to my and my wife's family. Some people was my friend
6.4 What were the responses / counseling and suggestions given by the relatives / neighbors / community after the episode? (Investigation: what to do after death, observing rituals, finding out the cause of death, someone else)
Mother told that why you went there, these all were happening because of doctors' ignorance. Mother knows everything, she informed us immediate after death but we had not realized at that time. We had hope for their life.
7.1 What was the reason for your child's death and what is your perception about the value of knowing the cause? (Investigation: knowledge about the cause of death, a desire to know the cause of death, potential factors that cause illness / death, possible effects on other family members or the next pregnancy)
Many people told us to know the cause of death but we don't have time. In an incidence, where my sister lives, a doctor cut the breathing nerve of a child and go off from there silently, parent though that baby was unconscious but actually he was dead. It all had happened because of S Hospital. We should not go there for treatment. We should admit baby in private hospital even on the cost of our land of village (Asset). We went because my first baby born there. We are regretting from our decision to move S hospital. We had gone there just for treating a cold and cough and next day we lost him. That was only son of my family. Now we have a daughter only.
We knew the death when the doctor told to stop pushing the machine. They should be responsible for medicine and treatment. Doctor of MN Hospital told them my baby will get well soon, in S hospital, they will keep the baby in ICU, provide ventilator machine because manually it's not feasible and he was suffering from pneumonia. Here at S Hospital, he refused to admit baby in ICU, as Machine was not available. They clearly told that you can go somewhere else, but we do not have other option. We did not have money to treat them in private hospital. It was impossible to arrange money within overnight. We request him to provide ventilator for baby we will paid you next day morning for same. But no one cares. Once a while, they put pipe into baby nose for ventilator but remove immediately. Doctor came to ward only once during whole admission time. A nurse kept my baby under a small bed (SNCU) but senior doctor scold that nurse and ask security guard to keep my baby outside from bed. Doctor taunt us also that they don't know care of her own baby but always keep reproducing baby and make ill them.
7.2 In your view, what else could be done to find out the exact cause of death and the cause of the disease? (Probe: probe, corpse, any other)
Mother: They do not talk about cause of death. They do everything in solo mode and always try to avert us. In case of any query, they become loud and rude. Once I told to doctor, see my baby what is happening with his breathing, he (Doctor) answer, I will give an injection and will cure. After that, doctor did not come to see. Doctors of MN Hospital (Private) were better than here. They did full check-up and respond well. He said we don't have ventilator so you need to go S hospital. Earlier private hospital was must better than S Hospital.
7.3 Are you aware of any method to identify the exact cause of death? (Investigations: bodies, investigations, etc.)
Mother: Reason was that doctor not treats well our baby and insert pipe in mouth. That's why our baby is no more.
Don't know much about this. Yes, I have heard about postmortem that was conducted in my wife's dad case. That was happened in S Hospital in an incidence where someone gave a wrong medicine to him. As I remember from last incidence, postmortem identify the reason of death. A neighbor whose relative is in police department, ask me about postmortem of baby. He told me that you should do it

for stop this type of incidence amongst other. But my mom refuse, she told baby is no more so there is no point to do postmortem.

7.5 What are your thoughts about the collection of tissue and body fluid samples to identify the cause of death? (Investigation: need, religious aspect, need of time, disfigurement, etc.)

How is it possible, it should be done on same time? One fellow, who is driver in American embassy (Delhi) told me about postmortem and also ask that we should knew the reason of death. A wrong treatment had happened with their wife also at their delivery time in S Hospital. Then his owner (for which he drives) shift her wife to a private hospital that costs approx. 25 Lakhs. He was totally upset even his child was dead at that time.

There are no any changes as per our rituals. At the time of Dad's postmortem, they (hospital staff) extract everything and told us the reason of death. Some gave poison to him. At that case there was not any mistake from doctor side. Cremation ritual are same in all case. In case of young patient, they (hospital staff) extract some body parts and use it for others.

7.6 How can parents and family members be contacted for tissue sampling? (Investigation: who, when and how to approach and for whom)

I don't know about this but it's good. Next time it could be followed. In our case, we are totally lost/absent mind even we left all goods like mobile at hospital at death time. We can tell others about MITS. Not to Mother, Father but others like brother can help to proceed this. It's better than postmortem because less cuts and surgery will require in this. People will sure accept this method.

7.7 In your view, what factors are important for the acceptance of tissue samples by parents and family members? (Investigation: desire to know reason, trust, confidence on health care provider)

Some people afraid from this, but knowing reason of death is must. In our case we were very tense but people were emphasizing to do postmortem. Even they came at front for help during postmortem. Recently I went to my native place it's been just 2 days to come here. I am based out from Uttarakhand. We usually thought that why we will do this after death. If the doctor care well, talk well, treat well and still could not save the life then we could convince for their advice (MITS). If they would talk well no one refuse their advice. Even in our case if they ask for MITS we would go for same. Our baby was born at 1st of last month and dead at 31st

8.1 In your view, what can be done to improve children's efforts to reduce deaths and to identify the causes of death?

We don't know in details, but that death was because of ignorance, if they care well, admit my child in ICU in timely manner, provide ventilator machine, they could save my baby. Earlier, in MN hospital they diagnose this as Pneumonia so keeping that in mind at S Hospital, they should admit it in ICU but they didn't.

Updated (mother)

4.2 What was your and your family member's reaction to the death of your child?

at that time we were worried, that our child has gone, doctor showed carelessness towards child, we were angried at doctor, we didnt express our anger we were worried, we were not able to tell anything.

5.3 Is/there any change in the interpersonal relationship between you and your spouse and other family members after the event

my husband remains upset, at that time he even lost his job. earlier everyone was happy now we fight more, he says due to my carelessness we lost our child, you didnt take the child to doctor, you did this, now we fight alot. now also we remain upset, we have three year old daughter we are not even able to pay attention to her. We don't have anyone in our family who can sit with us.

5.8 How your and your family life changed after the death of the child?

We all cry every day and are worried that we think why we went to that hospital, we should not have taken our child their baby would have been fine if we had taken it elsewhere. My husband is just very upset, he does not even eat food, he has become very weak nowadays he was admitted in the hospital too he became very ill. To reduce the tension he goes to work and then he remain fine. Sometimes he don't feel good at work but what to do, you have to do it. He has anger for doctor, that they have killed my child. He doesn't drink or smoke.

7.1 In routine day to day operation who takes decision about the major activities about the family?

All decision are taken by me and my husband. my mother-in-law is not there. if some decision in which we dont understand then we ask my mother.

7.2 During the hospital stay who discussed with doctors/nurses to make the decision about treatment and other procedures

We did not think anything in the hospital, we were listening to what the doctors were saying, we did what the doctors said, the child was very serious, so we did not understand anything, what should we do and what not to do.

7.3 After death of your child, who decided about the cremation and other post-death activities and rituals

About cremation, my mother told us how to do and what to do.

In-depth Interview parents of deceased neonate-3

**1.1**	**Primary respondent (relation with the child)**	Father
**1.2**	**Age of the primary respondent**	Mother- 25-30 years<br>Father-30-35 years
**1.3**	**Other members present**	Father's brother

2.1 Where do you usually go or who do you consult during your child's illness?
A doctor is residing nearby area; we went usually there and take medicine from a dispensary that is a bit away. The doctor next door has their own private clinic.
2.2 Where do you usually go or who do you consult to vaccinate your child?
We go to dispensary for immunisation.
2.3 People in your area / community are generally asked for their children's illness?
A doctor is residing nearby area; we went usually there and take medicine from a dispensary that is a bit away. The doctor next door has their own private clinic.
2.4 Mention the reasons for priority for the facility and / or healthcare provider.
We usually go to dispensary for Immunisation. Govt. hospitals have more negligence; they are not responding in a good manner and hospitals are also not good they are like fake.
3.1 Can you please tell us about the illness and events that caused <name> your child to die?
Baby had jaundice. First we took to HF hospital (Private), At there a Doctor (M) had done nothing and just told that condition was very critical. As they had not machine for jaundice treatment, advice to refer at other hospital. Again we went to C hospital, they also refer so lastly we went to place where baby born that was S Hospital.
3.2 Who supported you during illness and your child's hospitalization?
My brother-in-law.
4.1 Now you are requested to tell about what should happen when your child is admitted in the hospital? (Investigation: hospital, ward, length of stay, course of illness)
Father was there till four days. Mother was for more than three days. At that time, Baby was eleven days old. We did not know where doctor was doing right treatment or wrong but they told about good treatment. They usually not tell the health condition of baby, told only when we asked.
<Ward> Otherwise, it was good. After new govt; many improvisation has witnessed, administration going to strict. Earlier, condition was worst. Since it was a preterm baby, they did not put him under machine. I had not seen any treatment procedure with my baby since admission. Initially, they told that we will keep it in machine but not did so. In the name of treatment, they only give an injection and told to come at 10 o'clock. After that, when we went at 10 o'clock, they asked '' is baby breast feeding''. I replied ''yes''. I replied all what they ask but they did not even check. It was about four days stay at hospital. They told ''what is blood group'' but our reports were lost, what we had. Did not know, where it was missed, either at dispensary, earlier hospital or somewhere else. They (hospital staff) are very irresponsible. We had handover all lab report to hospital but they missed. We got upset for report. In the wake of that, they were not discharging baby.
4.2 Can you describe your overall experience of hospital care? (Investigation: investigation, treatment delivered, satisfaction level, quality of care, cost of care)
We had performed blood test. They suck blood a lot. They did it themselves
At time of birth, they demanded Rs. 60. We did not had change so gave Rs 100, even they did not return rest amount.
4.3 Can you describe your experience about communication by various hospital staff during your hospital stay? (Investigation: everyone communicated, which was the primary communicator, nurse, doctor, other staff, frequency of communication)
Doctor had already started treatment; they were doing right or wrong don't know! Its only they know!
There was ignorance at their level. They came at ward only in emergency, otherwise not. When the baby salivating more in their worse condition, only after that nurse came and advice corrective measure like wipe baby's mouth. Lot of ignorance there.

When doctors came at round, they started to scold us. I was worried about baby condition, why was baby discharging saliva at every time, why his breathing rate was high. Staff had dull response on all of this concern. I used to speak them that baby was serious look at him.
Security guard not permit more than one attendant in ward, as they told only person with patient will go inside. Doctor every time pissed off on us and told why you people repeatedly ups and down the room. Some time when they need us, call us loudly from inside. We were totally confused where we sat. The patient beside us was also scold by doctors even more than us. Govt. doctors are more irresponsible, even they are earning more than their salary so they have very much attitude. When baby born, they took money but it was good as every things going to be good. Each time doctor showed their irresponsibility. We were urging every time to see our patient but they did not. On a moment, baby was facing seizure but doctor not done anything more than infusing glucose and blood test. Unfortunately, blood report was missed.

5.1 Can you please tell us about your child's death and death declaration? (Investigation: Who had announced the death, body language and expression prior to the events before death?)

My dad was there. For pushing a pump (manual ventilator), at any time any of us had to be there (at hospital). We had sense when baby stopped breathing. All of us knew about death, when doctor said that call her father. They forcefully push the ventilator pump. Nothing was clear, whether he breathe or not.
Doctor told to brother “call baby’s father’’. When I reached to hospital. I saw all doctors involved in our case and remove all support devices (Pipe). They didn’t say that baby was dead. Didn’t say to me about anything. At time of death, baby was not suffering from jaundice. Earlier doctor said ‘’ baby not having jaundice’’
They did not tell the reason of death. Did not say anything. At time of death just told ‘’call her father’’. Around 11 o’clock, when I reached at hospital, baby breath and pumping was continued for one and half hour. After that I called doctor, they came and give an injection that was not effective and again after 5 min they told call her father. Baby was dead a little before that.
All the doctors coming to me came to force me, started pumping, started pressing and did nothing before (father). The doctor did not say anything. He (the husband) called the doctor there is a lot of breath in between (brother). Once, the doctor does not tell why the breath is moving fast, what is happening, they just told that the skin was full (mother). Did not keep asking (father)
In case of ask to the nurse, she keep screaming, and stop or see the other (brother). Come, she starts shouting like this, that is fine but negligence is there a little over there (brother). Neither is negligence but it is fine (brother).

5.3 How was your experience about the process after death until the operation of the body? (Investigation: procedures, documents and papers given, respect for religious norms, time taken, cost / payment, assistance from staff)

That was done instantly. All procedure was completed by them and frees us within 15 min. we came by our own car. That was so called so big hospital and when we need machine for baby that was not there. They baby got machine, he could be cured. It was told at the time of birth that it was a week baby.

5.4 Who supported your family and everyone in the community / neighborhood during this period? (Investigation: who informed them, who all came, what kind of support was given)

Relative supported us at that time. There was some ignorance at the hospital. We, around 8 people were present at hospital at any time.

6.1 What procedures did you follow after your child's death? (Investigation: Preparation of body, which prepared burial / cremation, other rituals and time)

We went to crematorium at around 8-9 o’clock mornings and buried. Lot of people were present except ladies. Brother-in-law prepares the dead body although we knew all the procedure.

6.2 How did you, your wife and family members face a difficult phase?

At that day, we went crematorium and called priest for worship and repeat worship after 4th days of death. That was pray for piece of soul. Its cured by time. Gradually we cop up and started our routine work. All should focus ahead. At that time all relatives were present.

7.1 What was the reason for your child's death and what is your perception about the value of knowing the cause? (Investigation: knowledge about the cause of death, a desire to know the

cause of death, potential factors that cause illness / death, possible effects on other family members or the next pregnancy)

They did not detail about condition. Like said ‘’see saline is infused, this is status of body’’ that’s it. Did not talk politely. Not talk with sympathy. My condition became worst by time as by standing in long que of hospital. We went there at morning but got number in evening.

7.2 In your view, what else could be done to find out the exact cause of death and the cause of the disease? (Probe: probe, corpse, any other)

We had not done post mortem. It depends on person. Through this, one can get exact reason of death. There are some benefit and loss of that as well. Loss is that ‘’ it is conducted through more cuts and surgery so people do not want to cut the dead body after death.

7.4 What are your thoughts about autopsy / post-mortem to identify the cause of death? (Investigation: need, religious aspect, need of time, disfigurement, etc.)

We needed to know the reason but doctor did not tell us (father). Nothing can be done after death (mother).

It is important. At least one should know the exact cause of death. They are not even talk in polite way. Do not talk.

7.5 What are your thoughts about the collection of tissue and body fluid samples to identify the cause of death? (Investigation: need, religious aspect, need of time, disfigurement, etc.)

This is a good technique. Doctor should pitch this otherwise they will say ‘’are you doctor or me’’

7.6 How can parents and family members be contacted for tissue sampling? (Investigation: who, when and how to approach and for whom)

Mother or Sister would be right person for this. We would must welcome the technique what you told (MITS).

One accept this, if was told by doctor. All staff at there was talked very rudely. Many of us were victim of that. But we all were helpless. In case of any request to staff, they call the security guard. There was a case beside our bed, was left the hospital because of ignorance. The hospital staff told them ‘’this type of service you will expect in low cost’’. If we have money, we must go to a good hospital. We have not, that’s why we came here. We can’t afford must we cannot shell out house and shop, otherwise how we will manage other family member. We had no other option. One should compromise quality in low budget. Doctors were overloaded. Space in SNCU was also less. In one SNU bed, there were three children. Later on, when my baby needed more space, they remove another from there.

7.7 In your view, what factors are important for the acceptance of tissue samples by parents and family members? (Investigation: desire to know reason, trust, confidence on health care provider)

At that time, it’s impossible to understand about post-mortem or MITS. It’s hardly to notice, what’s going on. As in our case, how time disappeared, we did not notice. Once Doctor told about death, we came directly home. They said themselves to take baby to home otherwise they made a big issue of this (Discharge). They called baby’s father after death in hospital and suddenly all doctors came to see, they did not come all together before death even at once.

Told; sorry we cannot do anything. They had dubious role, in one side they told “No hope in this, call her father’’ and another side when they saw us started to pump baby like they were tried to do something for save life. They always keep patient roam around old and new building of hospital. Even, for the lab test, we need to give sample to lab. The person at lab again asks to go somewhere else to keep the sample.

8.1 In your view, what can be done to improve children's efforts to reduce deaths and to identify the causes of death?

No one wants to do post-mortem but technique (MITS) what you told is good. Doctor should have told about this, about do’s and don’ts’. Usually, they do not tell anything. If they start to talk, everyone will listen. We will also follow if they teach.

In-depth Interview parents of deceased neonate-4

**1.1**	**Primary respondent (relation with the child)**	Mother, Father
**1.2**	**Age of the primary respondent**	Mother-30-35 years<br>Father-30-35 years
**1.3**	**Other members present during the interaction**	Nani, Brother

Context- The mother delivered her baby in 7th month the delivery took place in S hospital. The baby after delivery was admitted in nursery then he was discharged after few days. The mother of the child is presently staying with her parents.

Events that led to death of the child

2. Can you please let us know about the illness and events that led to death of your child?

He had cold in the beginning, the child was sneezing, and his voice was not coming, meaning he had a cold. Then we took him to the doctor, in emergency. The child was admitted in nursery at S hospital after the delivery, then after 6 days he was discharged. When after 6 days we came home, on 15th day I bathed the child, due to which he got cold. Then we took him to the hospital and they said that the child is drinking milk he is fine, they didn't admit the child on Tuesday, then on Wednesday we again took him as his condition got worsened. On Wednesday we took him to OPD, from there they referred us to ward 18. Then we went there, they did not see anything, just inserted the tube in his mouth and nose, 3-4 days the baby was fine, then on 5th day when the third tube was replaced it got hurt in the baby's lips and mouth, then to the chest and to lungs. He stopped breathing and was dead.

Maternal grandmother- When we stayed in the hospital for five days, all the children were put on Bag and tube, their parents used to hold that, they were not put on oxygen as staff will have to visit again and again.

Mother- when we go to doctor then they used to say that why are you roaming after us, we are seeing patients, i said baby has problem, then she used to scold me.

Maternal grandmother- We saw how many children were dying there. Not a single child returned safe from ward number 18, the child who has gone there means that 5-6 children were admitted and died in a day, there blood was withdrawn.

Hospitalization period

3. Now we would request you to tell us something about the period of hospitalization for your child?

We stayed in the hospital for five days, on the fifth day the child died. We was born on 23rd and died on 23rd after one month.

<about hospitalisation>

I had gone to the OPD on Tuesday, they said that the child is fine, then we returned home, then on Wednesday we again took him and got him admitted. From Wednesday we stayed for 5 days in hospital, at 4 his heart stop beating. The child was fine, the child had no disease. He was coughing and having cold, so we took him, he was also urinating frequently. He was not able to cough properly as the mucus was accumulated in chest.

I and my husband and my mother used to go to hospital, me and my husband used to stay in hospital for pumping bag and tube.

4. Could you please describe your overall experience about the hospital care?

When I call the doctor, she did nothing, she just changed the tube. When senior doctor used to come they tell us that child is fine, you have to take care of the child, when they left then nurse showed carelessness. Other doctor used to talk among themselves when we go to them and they scold us.

<frequency>

Senior doctor came at 11 in the morning after breakfast they tell us about child condition, they came once in a day. One senior doctor came with two junior doctors. They used to tell that give medicine, take care of it properly, the child started recovering. Then doctor showed bit careless.

The baby was born in 7 months and they used to drain his blood 3-4 times daily from morning till evening. In five days they had withdrawn so much blood, from hands and feet, the child started becoming blue. He was 12 grams less than 2 kg. They used to take 2 tests, the third test was normal

everything has come normal, if they haven't put the third tube in the last, nothing would have happen to the child.
Maternal grandmother- the tube hurt the child on lips because of which he was not able to cry and he was bleeding.
Mother- we didnt buy any medicine from outside,when we returned home from nusery earlier from hospital we got calcium medicine, then child was fine.
Maternal grandmother- When child was lying in bed he used to move, he doesnt take any covering of cloth over him, he was active and fine and normal.
Mother- after i conceived, after one and half month after conception in february, something stopped blood started coming, i visited private doctor, she gave me some injection, i thought i had a miscarriage but the child survived, this was that child only.
Maternal grandmother- Everyone is saying that 7 months baby is weak but he was fine.
Mother- <cost> in S hospital we didnt spent money as test was conducted in new building. two test were done outside, cost of one test was Rs 60 and cost of other test was Rs 100.
Maternal grandmother- one ultrasound was condcted in private during pregnancy.

5. Could you please describe your experience about communication by the various hospital staffs during hospital stay?

Mother- Nurse was fine, she used to listen to us at once. doctor used to scold us and everyone. she said dont roam behind me.

Death and post death period

6. Could you please tell us about the death and death declaration of your child?

Mother- Senior doctor said child is serious after seeing the child. during admission also they said child is serious, they said child is gone, i said when the child is breathing how he can die, i brought the child here in better position, they fooled us and inserted the tube. he was suffering from cough they would have given oxygen and child would have become better. they inserted the tube on very 1st day.
<who declared>the senior doctor told child is serious, and there is no chance of survival. in morning i came back home, my husband was there in hospital, child cried and tube came out, my husband told doctor that tube has came out, then doctor inserted the tube through nose and mouth, it started bleeding, then after 4 his heart stop beating and he died.
Maternal grandmother- doctor came and saw the child, my child was lying with other two children and tube was there. the needle which was inserted in his hand to give glucose came out and there was blood all over even over his blanket.
Mother- When I went to the doctor and said that blood is coming out, then doctor said to bring cotton. I said to them to come and see my child once.
<cause of death>
They said child is serious and have double pneumonia, I said earlier he was not having pneumonia, in last doctor said child is having pneumonia.
<behaviour>
senior doctor was good, he said to take care of the child, when senior doctor used to leave then other doctor showed careless behaviour, they used to laugh among themselves, when we go to ask for something then they scold us. Only doctor who used to come and see the child was good. Also nurse was fine.
maternal grandmother- one child father slept during pumping the bag and tube, the staff came started scolding him that your baby is dying in life and you are not looking after him. the person get tried by constantly pumping bag and tube, there should be proper oxygen pump for children.
Mother- senior doctor was fine, other doctor used to scold, 1st they said they want blood donor, then i called my brother for blood donation, they said to him to sit and wait, my brother came at 12 at night, after that he said him to sit and wait because they didnt have time now, when we will be free we will call you. we dont know what they did of blood taken. for taking blood they said to sign the paper, and started scolding my brother then my brother also got angered that you have called me at night for blood donation and you are not taking blood now.

7. What was your and your family member reaction to the death of your child?

Mother- this all happened on sunday evening at 4. when all this happened i was with my husband, mother, sister-in-law, my brother went back after donating blood. my brother came when baby was

dead. doctor told my husband that your child is serious, they called me but sent me out because i was crying as baby died. after that they handed child to us and we returned back home.

8. How was your experience about the procedure after death till handover of the body to you?

It took 20 minutes after the baby was over, we had left soon. We just signed and they gave child to us. Family member left at 4 after taking the child. We did not bring the child home, my husband and brother took him straight to burial place from the hospital.

9. Who all from your family and community/neighbourhood were present at the hospital and how they supported you during this period?

Maternal grandmother- i and my dughter was there. My girl was crying and screaming and she was supporting her and I was crying myself. No neighbour and relatives came now, they came when child was born.

Post death procedure and coping mechanism

10. What all rituals and procedures did you follow after death of your child?

Mother- Money was paid at burial place. Child was directly taken from hospital, we didnt brought the child home. My husband, brother and his some friends went for burial, i was at home, i didnt go.

Brother- From the hospital we went near Green Park for burial of child, they took Rs 1500 for burial.

<who performed the rituals>

The child's father did. The pandit digged the ground, put on clothes, put some salt, performed some prayer, and then child was buried.

11. How did you, your spouse (wife/husband) and immediate family members cope with the difficult phase?

Mother- My husband supported me during that time, he said there is no use of crying now.

Maternal grandmother- She used to wake up at night and cry, she didnt properly sleep at night. child father was also very sad at that time, he supported my daughter.

12. Is/was there any change in the interpersonal relationships between you and your spouse and other family members after the event?

At that time my husband supported me, yes, he used to say, there is no use of crying. My sister-in-law also supported. When I used to cry my husband also sometimes cried along with me. He supported me, even my mother supported me. Now I keep my girl child with me especially during night to forget the memories of child.

13. How did the family members support you and your wife during the difficult phase?

My aunt used to come and explain, I used to cry, she said don't cry, it was not yours that’s why this happened it took one and half months to come out of that.

14. What were the reactions/ counselling and suggestions given by relatives/neighbours/ community after the episode?

Maternal grandmother- those who used to come used too counsel us.

15. Do you think and/or discuss with your spouse/ family member(s) about the death and related events and blame someone/something or feel guilty for the same?

I blame the doctor.

<view of family member>

they think I bated the child so all this have happened, that’s why they blame me for this.

16. When did father and mother (if working) returned to work after the death of the child?

<about father>

my husband returned to home after the dead of child as his sister-in-law also got expired. Then he returned to work.

17. How your and your family’s life has changed after the death of the child?

Mother- When i used to stay at home i always had tension, now i have joined job 10 days back, earlier also i used to work but left my job during pregnancy. it is better to work as you can earn some money even divert your mind from memories of child.

Maternal grandmother- i work in printing press of card.

Mother- my daughter has epileptic attack, she faints, we are giving her medicine from 3 years. my husband didnt smoke or drinks.

Knowing cause of death in detail- Autopsy and MITS

18. What was the cause of death of your child and what is your perception about the value of knowing the cause?
Mother- child died and we buried the child. knowing cause of death is important, doctor said he had pneumonia, we cant say anything. i dont think he was having pneunomia.
Maternal grandmother- What is the reason to know now, what has happened has occurred.
19. In your view, what more could have been done to know the exact cause of death and the reasons causing the disease?
Maternal grndmother- I do not know whether, what the doctor told me, what is right, what is wrong I do not know.
20. Are you aware of any method for identifying the exact cause of death?
Maternal grandmother- <about PM> yes i have heard of postmortem but never seen it, it happens in case of accident, suicide. i dont know whether it can take place in case of child death and we can know the cause of death. i dont have much knowledge as i am illiterate. i only visit S hospital for medicine and checkup.
21. What are your views about the autopsy/ post-mortem for identifying the cause of death?
Maternal grandmother- I do not know.
22. What are your views about collection of the tissue (like done for biopsy to diagnose diseases) and body fluid samples for identifying the cause of death?
Maternal grandmother- this is good technique if it happens, we can know the cause of death by this, in my view it is right. It is important for us to that no child should suffer. On Sundays I get worried and upset.
<religious aspect>
It is important to know the cause of death, it depends upon the person to do this or not.
23. How parents and family members can be approached for the tissue and fluid sampling?
Mother- they took out the blood many times, filled the blood in 3 bottles. nurse withdraw the blood many times, once she withdraw after 15 minutes agin she used to come and withdraw the blood.
Maternal grandmother- i can go to hospital if they call me for some thing by which we can know te cause of death, no child came out safe from that place. if they withdraw blood so many times then child is small it can affect his health, and he can become serious and turn blue.
Mother-<when> Should have spoken when the child was fine. <who told> senior doctor should explain about this. <method of explanation>they should have shown us the picture.
24. In your view, what factors are important for acceptance of the tissue sampling by the parents and family members?
What has caused the child to die.
Decision making dynamics
25. In routine day to day operation who takes decision about the major activities (purchases, treatment, spending money) about the family?
Mother- i take all decision myself, about treatment, husband used to go to work he dont know much about S hospital, even when i was pregnant all test, checkup and treatment was done here.
Maternal grandmother- she used to take care and took all decision by her known.
26. During the hospital stay who discussed with the doctors/ nurses to make the decision(s) about treatment and other procedures.
Mother- I and my husband used to bring medicines, he do not know that much. From my mother home S hospital is near as compared to my husband place. We decide on our own, we go to emergency in case we need.
Maternal grandmother- father stays far, if child was not well we used to call him so he comes direct to S hospital.
27. After death of your child, who decided about the cremation and other post-death activities and rituals?
Mother- I don't know about the rest, I had my family member and brother. In child we don't do any prayer it happens in elders, I didn't consult anyone.
Maternal grandmother- she doesnt have mother-in-law, her father-in-law and brother-in-law stays separate. i take care for her. Child medicine is brought from outside, and not from the hospital.
Summarization

28. Now, we are completing the interaction/interview. Would you like to add anything?
Mother- I have to say that this should not happen with any child from now on. The boy was in good position, boy only had fever, doctor came and put the tube on Thursday, that boy was finished. A good boy came from home, ate food, drank tea, ate bread next to him, the boy was fourteen years old. I and my husband was doing bag and tube turn by turn. The child was in the nursery, he was perfect, we brought back safe and there he was also provided with oxygen and was kept well. Ward 18 was very careless, the delivery of the child was also done in S hospital, fifteen days was fine at home, the child completed one month and then died.
Maternal grandmother- What should we say, the day we saw such a strong kid, he used to put his number first, seeing such a big boy, my mind gets upset. By doing bag and tube there is pain in hand, even regularly doing this person can sleep in between, doctor look at their own comfort, the best option is giving oxygen by machine.

In-depth Interview parents of deceased neonate-5

**1.1**	**Primary respondent (relation with the child)**	Mother, Father
**1.2**	**Age of the primary respondent**	Mother-25-30 years<br>Father-25-30 years
**1.3**	**Other members present during the interaction**	None

Mother of the child was staying at her parent's house; his husband was also present. During interview her mother and father were also present. Their two children have died at S hospital, first one died in 2016. Then after that they had 1 daughter and third child also died at S hospital.
Mother -Death in S Hospital is very high, its case is more. By the way, this time I have made a mistake, he told me that S people, because I was getting the same check-up from the beginning, I went from here, so whenever you talk about delivery, if you are pregnant. Would be fine with me okay. Father- That means whenever you have to have a child is sure to consult us.
Mother- Means you will consult me whether you are worthy of having a child or not, so I cannot speak of the lack of S Hospital, I had talked about this. Father- This time, we have no complaints in the hospital, but last time I had a lot of complaints and did a lot of wrong. Mother- Last time, when she was pregnant for two months, she had started the check-up, she had done all that, according to her decision, whatever she ate for food. As he had said to live, he even told me how to sleep, that the baby will not be turned upside down, but he told me in the last eight months that the baby does not have a spinal joint open. Is, there is water in the head, all this was told earlier in the boy, and this deficiency was first told in the boy, all this was told in the eighth month, not in the beginning. In three and a half months, in S Hospital, doctors had done ultrasound from inside, but they did not tell me what is in the report.
Father- Last time, as the doctor said, we have gone the same way, but this time we went in the last.
Mother- We had gone to the village, we are residents of Gorakhpur, when we came to know that the baby is upside down in the village, we came here for treatment, but when we got treatment, we also told about it in S Hospital. I have a large head of baby's spine, and the spine is open And do not know how long it will be alive after having a child, whether it will remain alive or not, but the child will come out alive, those people had guaranteed this thing, all of them had spoken for this child.
Mother- This time I was not mistaken when I started feeling pain when it was raining outside, and by the time the car arrived, my child was already home. Then we took our morning time there
Grandpa - It was raining so much that it rained outside all night we kept thinking that if taken outside it would get wind.
Mother- When I took her in the morning, it was said that this deficiency was asked, where is the child, so I said that the child was showing it, but the child is at home. Then they said that when they are at home, why have you brought them here, they have not treated me while keeping them at home, why should I lie, by referring them, they referred me directly to medical (A Hospital) Then when I went to medical (A Hospital), when I got time to see them, then treat them again, and gave the medicine, injection, and whatever was needed to the child. Then the child was kept till evening, then after that he said that now I do not have a seat and also do not have oxygen, I cannot keep it, that means I do not have a place in medical (A Hospital) , And said that you should move to GBP Hospital or S Hospital, first of all S Hospital was put on top. When I was going to S Hospital from the beginning, it would not be right for me to go elsewhere or somewhere, the decision was taken by me, my husband and my father, then we took the child to S Hospital itself. Then when we took the child there, those people transported me from morning till evening, where they said they went, then they sent me where the hospital is open now in the new building, after the new emergency there is SSB Surgery takes place Then when I went there, when I met the elder doctor, his name was…, he is the elder doctor of the hospital, he treated the child again, okay. And then when they see it, then they say that we cannot do any surgery for a five-day-old baby, we can do so much that we can put it on medicines, after 2-3 months, if we drink milk then it will come, then

we will Can do surgery on it and the rest of this child has no cure So then he asked me to get MRI, then see from the child's mind that even there is a shortage, then I got him MRI and gave it to him, I reached the report after five days, then he saw that the child's beating It is very small, so it cannot stand after the operation. It was said that even if we take it to Operation Theater, our hard work will also be sold and the child will not survive. My child was HOA on full time, but only after the doctor who gave time. He was not lacking in the rest of the body, everything was healthy and healthy.
Father- The doctor who had given the water and the head was all wrong.
Mother- There was no water in his head and the head was not too big, the head was small but the spine was open.
Granny-baby was like it was supposed to be, normal was the same, it was long, left, the weight was also like it was up to four kilos, it was a healthy child, it was not that weak, when expire, you left your arms and legs long, But he had to leave. There were a lot of breaks in the leg, what was lacking in the leg, when here (in the spine), there was no work, then the fingers were not moving, it was not moving. Tetanus was injected when the doctor, who is the doctor around me, told me that the baby did not cry after injecting, and then a week later, the finger started moving, both legs started moving. Then I gave the baby a kiss while massaging, then he smoothed the leg like this, then I told my girl that your baby's foot has become right
Grandpa - We are residents of Gorakhpur, there you will also know that there is a medical, but what is there is there are big drawbacks, we give medicines but there is a shortage, one is also in Lucknow - PGI, and many others Is a hospital. The operation of my brother's girl was done there, it cost him one and a half lakh rupees, in which the government had given 75 thousand, now it is fine. But what did the careless people do in S Hospital last time and this time too. There is a doctor NKD, S Hospital doctor has been there before, now he has retired
Granny- Doctor, we have to know why.
Mother- The former was in 2016, and the essence was over. And this time the baby should be 12-13 days old.
Father- This time we do not say that he has a mistake, but the first one is the fault of total, the doctor of S Hospital
Granny- My brother was married to a boy, had gone to marriage, I did not know the condition of my baby girl, I had given her the mode to go to the in-laws, we had given her a go, let's keep it for a year and a half. Have taken it, they did not think it necessary to talk to me, then when my girl came to the wedding, she told her mother that it was such a thing, when my girl came to see Me, I was leaving for Delhi. Then the quote is such a thing, then I got her ultrasound done, which is our nearest hospital, meaning there was an ultrasound done, ultrasound was done 2-3 times, water turned yellow and so on. The doctors said that the baby is upside down means that it is so bad, and then we said that this is not a big deal, the child is straight, it is reverse. , (Father- Even then, someone did not tell the deficiency, someone has something wrong in it), and did not tell the shortcomings, only this was told. If I leave it, then it is multiplied, it is not multiplied but I brought it.
Grandpa - I took it to the doctor, I was sitting in front of the computer at the time of ultrasound, the doctor saw with a stethoscope, the child's position was forward, the child's position was not getting properly, the baby was a little bit, the child was Whatever you will get, the doctor will talk, you will get what you can see, nor when the child was broken, when the child was broke, he was not able to see, only then he has no joint.
Mother- It looks like this in my boys only, my girl is very good, and she is after the first boy. When I had my first boy in the stomach, I had shown the same from the beginning, and my delivery was also the same and in the last eight months I told that whose spine has not been joined, and his head is large, He was saying that the child will be from the operation, careless care
Father - We have done a lot of carelessness while making delivery, now we are suffering the same trouble. Mother- It is a matter of time that very careless curry happened at two o'clock in the night (granny- water is not in the head of the child and after injecting so big, the water will come out if the water is coming out) When he was in the stomach, he was doing so many injections and checking if there

would be water in the spinal cord or head, there was nothing in it. Father- The boy was completely aware. He was born when he was born.
Granny - As soon as the injections were done, it was safe, as soon as the father came with it, we buried here and wherever he was injected, he had a totally different mark on the child. There is nothing visible in that period, no man can do anything except cry, the pain is painful then it is big or small, the former was just half an hour
Father- was alive for 15 minutes or half an hour, they were also taking it to get surgery, the doctors were taking it for treatment, it was there at that time, then it got expire.
Granny - So many injections were done, when I saw the child, there were signs of injection at every place, Nil was read wherever the injection was done, when there was no water, then it will come out.
<Whose decision was to take 2nd child to S Hospital > Father- The second time we did not go to S Hospital, we went to SV Hospital which was near here, took us there, our girl was born, it was clean, someone looked like someone There was no problem.
Mother- When the first child was finished, I did not go to S Hospital again, I would not lie down, and then when my girl came in my stomach, I did not get treatment in S Hospital, I have full treatment.
Father- There is a D Hospital, there is a hospital with children, all treatment is done in D Hospital, but delivery is done at three o'clock in the night, (Grandpa- is an old doctor, the government is retired). I did not think to go to the SV Hospital, then took it and then the delivery was normal, all this on the time of the other child
Granny- Doctor, as soon as I took her (2nd child), her father was in the village, I, my son and my eldest son, as soon as I took her as my granddaughter, immediately any doctor started crying. I did not come, everyone was asleep, when the baby cried, and all the eyes were filled with tears, and then said Madam, now you go out.
Mother- My girl was born normal in Shivalik, is a private hospital, and even this time (3rd child), when we came from the village, we had gone to S Hospital, thinking that she will go there, then she will be there, she will look like that. Father- Did not go there, the talent in the D Hospital, who was a doctor, was doing the treatment, then he said in the last that it will cost so much money to get it done in S Hospital itself. Then we went once or twice S Hospital, did not go again, and then started seeing these. Then they were taken when the boy was done, this is the story, whatever it is.
Granny- He said that you had to get the ultrasound done, and then went for ultrasound. Father- Then we went to get the Ultrasound done. Granny- Then went to see the report. Father- He did not enroll in S Hospital, got MRI done and got blood test done which is the new building where the blood test is done in the glass building, then he showed it after checking, then after that he said that after three months we have surgery will do By the time you take it to the house and do the dressing and bandage, if it had been admitted, the child could have survived or stayed in the hospital. Mother- I was told in S Hospital that we have our own children, who do not have oxygen for their delivery, we are putting our children on the pump and we cannot tell anything, if we want to recruit. So you keep running the pump. When I took the elder to the hospital, the second one opened In SSB, then he told me who to operate when the child is 60 days old, he wrote a vial like less, water was to be doused with glucose and he had to be wet on the child's wound and I did that I used to do this for three times by bringing here (mother place), as it used to be massaged, soaked warm water and then wiped it on her body first. Then open the bandage which was on his wound and keep the cotton cloth in Dettol and keep it so much, then he used to soak the cloth in water and keep it on his wound, and then put the tape, cotton on top. She used to put cotton on it. I used to do the same as told to me. To a nearby doctor who is a retired Doctor of Medical (A Hospital), I said that my boy is hungry, when he looked, and I showed the prescription of S Hospital, then it is said, you gave the child the infection medicine. Not now, infection has increased, wounds will open The wound will spread again and again, when they saw the leaflet, they had written only a bottle in it and had not written any medicine for infection, that means they should not have any kind of infection to the child (S Hospital doctor). Did not write then the doctor I showed them have written me an infection medicine. Then I gave the child the medicine in the morning time, like this was the time I took them to him, from there I was

fine. Then at three o'clock he had started crying, the child, then he felt so fast, then I took him (Father was -105 degrees). Then I saw that suddenly his stomach also got swollen, and it got badly.
Then me and my father, whoever sits here with private children doctors, it was Sunday time, all the doctors were closed, they were on leave, I could not find any doctors, I am telling the whole area near this. (Grandpa - everyone knows from here to Sector-30) No child doctor was open till Old Faridabad. I could not find any doctor with children. Then I brought her to S Hospital in the last. It took me half an hour to go At three o'clock my Babu was hungry, I reached S Hospital at 8-8.30 in the night, because when my child said to take me home, keep me at home, then he just kept I had given a medicine of glucose water, and then when my child was hungry, I took S Hospital again, which she took at 8 o'clock, there was a long line, there is a long line everywhere
(Father- When I spoke to the security guard, and then got ripped off) Then when I was taken in, the doctor saw how his blood got starved for the first test before treating my child, when 7-8 turn out when the blood came out, my child stopped beating, when the blood stopped, all the doctors were there, all the doctors were senior, they tried their best and started breathing and then started taking medicines. Done (Father- he started reading the tour, etc.) Mother- Then he started visiting on the tour quite a few times. He came back quite often the doctor was with the child until his visit was normal. When the child broke normal then he referred me from emergency, then shifted to the room (Father - here is the number 18 ward in the H-block) so when I sent it there was a line of children. Every five minutes two children were dying, then in the second minute four children were dying. Meaning no child had gone away from there, all was over, all the children, meaning by the time I was admitted till the morning, there were above fifty children. Father- He was also negligent there, the doctor was working well till he was in the emergency, after going there he started taking more carelessness.
<about treatment> Mother- The doctors of the emergency who wrote that medicine were running the same medicine, to change the medicine, because when the senior doctors came, they brought my child along with them. Beats were checked every two minutes. They said to raise Father-Blood, they did not offer it either. Then the doctors who came there started scold them all. The file did not even say our address was made disappear. Mother- Shivam, there was a shortage of the child, because of this, the people had suppressed the file of the child, that there is a deficiency in the child, the child was not to be saved anyway and those people had no other treatment, so What can we do with when the big doctor came, he asked for my child's file, then rebuked him and started treatment again. Then treated him as long as he had breath

2.1 Can you please let us know about the illness and events that led to death of your child?

Hospitalization period

3 Now we would request you to tell us something about the period of hospitalization for your child?

4 Could you please describe your overall experience about the hospital care?

5 Could you please describe your experience about communication by the various hospital staffs during hospital stay?

Father- According to me, who was doing us, it was not a mistake of the doctor. There was no mistake of the doctor in the treatment. Yes, but the lady who was taken for the first time was a lady doctor, she spoke upside down. Mother- They told me that when the child was normal, she was born at home, why did you bring it here, for whom have you brought it? The baby's tree is not moving, the leg is not moving, and why did you bring it here at home. Father- Meaning that on the other hand, they used to go upside down and were automatically thinking that we had not spoken anything yet, and understood themselves.

Death and post death period

6 Could you please tell us about the death and death declaration of your child?

Mother- The child was finished between 3.30-4 am. Father- As both of us were sitting together, as both of us came out, my elder brother-in-law was giving oxygen to the child, meaning we had been in places for two-three days anyway, and then broke out in the street. As soon as I sat down after 10-15 minutes, I saw that the child had expired, meaning he had come to speak. Mother- My brother was with me all the time, was not away from the child, as I was driving, on the go, my brother said that you are getting sleepy, I will take you rest, so my brother is pressing the pump Was. When the doctor came to treat him, his heart

stopped beating, then when the doctor saw it, blood came out of the mouth and nose and then it was over Otherwise I will not say that he had a shortcoming because he had told earlier, first Sister was when my first boy was over, then he said whenever I want to do a child after 3 years, I have to take a decision. And after that we will run medicine for some time, will test why you and your husband are lacking in such a child, what is lacking in the boy Still this time told me that whenever the child does, do it with opinion from us and do not do it before

Father- After the first boy was born, after that we went back to S Hospital. Mother- My case was spoiled by those people because I am telling you directly, whatever the deficiency. Father- We went to S Hospital, for that which we have run for 2-3 months, it is not, I did not run, I used to give the contract for paint, the boys thought that they used to go with them (wife). Went for three consecutive months but no hearing was held. Then said that the Chief Medical Officer who sits, went to him, then the same (wife) had gone, I was standing outside, so he also went careless. Mother said that your child's head was large, and it looks like this. Father- However, who lives in LN, there is an A Hospital, there was a professor and worked here. So I thought of going to them to talk to them to know what to do to get treatment, then I thought to work, it is a matter of home. He must have gone, he would have done something. Then showed in the emergency, it said that it would be surgery but nothing happened in the last

<cost> Father- Yes, the test was done from outside, MRI was done from outside, and the test was also done from outside. All the papers were there, now the child expired and threw everything.

<who declared the death> Mother- Do not know the name of the doctor, when she was taken to emergency, the girl was 25 years old, she was told that she had come

7 What was your and your family member reaction to the death of your child?

8 How was your experience about the procedure after death till handover of the body to you?

Father- The child was given immediately, that means whatever must have been put for 10 minutes or half an hour, keep the file, and there is no slip on the sister's gate without making a slip.

9 Who all from your family and community/neighbourhood were present at the hospital and how they supported you during this period?

Mother - It was May in the hospital, my husband and my elder brother were enough. We were just three. When he said it is no longer Shivam, it is now over. Then said now it is over, you can take it.

Father- Earlier Bauji was also there, but the time when he was expired was not the

10 What all rituals and procedures did you follow after death of your child?

Mother: I had brought him home directly from the hospital then he was taken away as the rituals are, as it is for the elders in us, in the same way as in the younger ones. Then my father took it all away. <who performed the rites> My father did all the action, the child's maternal grandfather did it. Father - 12-13 people went to burial. (Granny- All the people of our locality had gone, all of them had known)

11 How did you, your spouse (wife/husband) and immediate family members cope with the difficult phase?

Father - A quarter of a month is not worship. Mother- As soon as two months are over, my baby is finished. The 13th was over, now it has been two months, what is recovering as it was. One is my own little daughter, she is engrossed in it what else can she do. They pay more attention to it.

12 Is/was there any change in the interpersonal relationships between you and your spouse and other family members after the event?

Mother- Nothing, no problem, all is well.

13 How did the family members support you and your wife during the difficult phase?

Mother- My father and mother had handled me I have supported my husband more than that.

14 What were the reactions/ counselling and suggestions given by the relatives/neighbours/community after the episode?

15 Do you think and/or discuss with your spouse/ family member(s) about the death and related events and blame someone/something or feel guilty for the same?

The doctor and said that we tried our best but the child was not saved. Anyway, there was so much deficiency in the child. Father- See, because of that deficiency, we also did not say anything, now it looks like that, I did not say anything, I could not blame them either. Mother- It was our fault too much. Father- But when the first boy was the HOA, all the mistakes were made by the S Hospital. Even then, whatever tests were written, all the tests were done, an ultrasound cake was inside and the rest were done from outside.

Once the color ultrasound, all the tests, blood tests are inverted - all were done from outside. Mother- Whatever test they wrote, which could have been done from inside, they had got it done from inside, and what could not have been from inside, I had done it from outside, the medicine which they had given in my first child, I completed The medicine was eaten, whatever medicine they wrote from inside That means, whoever writes the pill from inside, I used to bring all the medicines, I have also consumed that medicine, earlier I did not leave any deficiency in the child. Even then my child had a spinal cord open and there was water in the head. That means there was no water in the baby's head, but there was no water in the baby's head, but yes was right, but there was a spinal injury.

(in between talk Granny telling her operation experience at S hospital) So in this child, I did not go there, when I went there in the eighth month, when the child was upside down, I did not know that time. Was that my child is lacking in this. He wrote for taking an ultrasound then got an ultrasound done and then it was found that the colored spine of the child has been opened again.

Then they saw me then gave them the medicine for infection, that the wounds of the baby which were water, should not be harmful for the mother.

Mother- This time I was late, I will not lie.

16 When did father and mother (if working) returned to work after the death of the child?

Father- My duty is not any government duty, you can go anytime. I started going to work after 15-20 days, but I felt less noticed. Nana - this is your job, if it comes to work, then it keeps on working day and night, the site does not come up, you have to sit, I do glass work, and they do paint work.

<The daughter enters the room> Mother- This is my girl. Granny - for this (2nd child) I did not take it anywhere but took it home. Nana - It is God who worsens, one is a doctor, another is a doctor, if there are deficiencies, he is not moving, everyone has to die, but when it is written, everyone has to die.

Granny- who is telling this (nana) Dr. N K Dogra is now old, he said that if there was a shortage in your girl, he did not do anything under pressure, he said that if the girl was lacking in you, then this (2nd child) child is also lacking in you. Your son-in-law is lacking, so the boy will fall, so I said openly to my son-in-law, do not consider it right for me, break the big people, get your check for understanding.

Mother - We will get that test done. Nana –Dr NK Dogra keeps talking to Saab, he worked at his place, he had said a test, got it done in medical, we said to get it done, but we will not get the report in private and will show all normal, will get it done in medical You will get a report.

17 How your and your family's life has changed after the death of the child?

Mother- Not like we were the same as before

Knowing cause of death in detail- Autopsy and MITS

18 What was the cause of death of your child and what is your perception about the value of knowing the cause?

Father- We knew that the reason is that the lump is there (Mother- They said that the infection has failed), the infection has occurred and has become hungry.

Mother- I want to know why this is happening. For this thing, think why there is a shortage, that whatever boy lives, why there is the same deficiency in one place. The remaining deficiency can also come in the hands and feet, it can come in any part of the whole body, but why the problem comes in the spinal cord, the information about this thing is necessary for us, nothing else is there for us.

19 In your view, what more could have been done to know the exact cause of death and the reasons causing the disease?

Father- Nobody said to get anything done. The earlier ones were probably asking for the test. Nana - No, I did not say that, just saying it upside down, the child is here then I said yes it is. Just then said that take it home.

Mother- delivery like baby when born was (1st child), they are normal baby as per the operation, they reverse the baby, whatever is inside the cucumber, it does not go inside, it is in the normal delivery It happens as if the child cried, he woke up and showed that you have to treat the child that your child is the same and this is the risk that you can see, the wound was also shown and the child had to be screwed. The child broke even cried then was taken for treatment.

20 Are you aware of any method for identifying the exact cause of death?

21 What are your views about the autopsy/ post-mortem for identifying the cause of death?
Nana- It is known that the post is in the accident of the mortem, but if not the HOA, what to think about it. Mother- We don't know anything about this (post-mortem)

22 What are your views about collection of the tissue (like done for biopsy to diagnose diseases) and body fluid samples for identifying the cause of death?
Mother- They did not take all of this. For this we would have agreed. Father- For this, what would we do for a couple of hours, for a day or two, if someone tells me to we do not see anything from him. We were never running out of money or treatment

Mother- If they had stopped me, we would like to find out why your child is falling, and why the child is dying. Means hungry, or infection, or because of which. He did not tell all these things. When Babu was over, we immediately drove away

23 How parents and family members can be approached for the tissue and fluid sampling?
Mother- At that time, parents are not in that position and they do not want their child to have a post mortem and there is a problem that in this way their child, the child is normal, will tear the child out, so any mother's heart is hurt. Will be there If we do tissue sampling in this way, then you will have to take the gift for treatment, then you can give it means that it is theirs, but I did not say it to my child, so I did not give it. <Who should approach> I cannot tell now, at that time someone from the hospital would tell

24 In your view, what factors are important for acceptance of the tissue sampling by the parents and family members?
Mother **-** The information you are taking from us, will be useful for giving feedback to someone later. Father- See, here I am talking about a boy who had become a boy, the doctors are talking like this, the people were quarreling with him, and the doctors had spoken as if his family had come to say remove this garbage, this garbage Remove, tell me like this. There were 7-8 people of that family the battle was fought again with the doctor and the fight with the guard. There were two or three children expire there then he broke and later the child got expires and then he too fought. It does not mean that they say that the child has expired or that there is no such thing that there is no quarrel and let go out of peace, they speak in reverse, and this thing is in front of me.

Decision making dynamics

25 In routine day to day operation who takes decision about the major activities (purchases, treatment, spending money) about the family?

26 During the hospital stay who discussed with the doctors/ nurses to make the decision(s) about treatment and other procedures.

27 after death of your child, who decided about the cremation and other post-death activities and rituals?
Father- My father-in-law took it, all the people who lived in the house also belonged to him, and the main decision was of the father.

28 Now, we are completing the interaction/interview. Would you like to add anything?
Granny - We were sitting outside, two or three women were on the other side, its father was also sitting there, my girl is also sitting there, she was sneezing, I asked what is the matter son no problem. Mom, nothing, and sent it in the morning to come to get it checked, it came to get the check done, to the doctor, there is a doctor nearby, but at my deposited Gurgaon work, I said if there is any problem, tell me, And I

was at home, my father was also there, my eldest boy was also at home Till then I asked him what time is there, there is no problem, so I did not bid, then I said you go in, I lay on the bed and then later told my father to speak to my maternal aunt, she came and said, Didi you sit here and Sushma are upset, as soon as I went in, hardly took 2-3 minutes and Babu got into it. Now half the torso and half the torso are out, tell me in that period, where can we take it now, nor can we take it private, nor can we take it to S Hospital, it is too far from here it takes time to go to S Hospital Now you tell me what it was raining outside. At 5.07 minutes, there was a born Babu got out of the bathroom like Babu got it on the ground. Now his father was giving me the street outside, you are not leaving, you are doing this, if anything happens to the girl, I will do it to you, as I was told to say, I am not one of them. Listened what I should tell them in which position they are in, like in the morning, I sent it to S Hospital, and the doctor said that it is like that

Mother- I am thinking that S Hospital will go and get feedback from him, whoever writes the test, he does not even know who will write the test or not. Father- He is speaking, we have no information on A Hospital to get the test done by A Hospital, just went for a turn, there is no knowledge, I have seen everything from S Hospital, so I go somewhere. Mother- The operation of my mother is the same, my younger sister also has the same operation of the appendix, and my delivery is also the same, now my younger brother-in-law is also treating his feet. And I have gone the same in this child, only then we have learned more and we always go there. Father- If he had said that he had to go to medical, then we would have gone; we have no information about medical. To get this test done in S Hospital, we speak upside down, see above, and tell me, I live outside, Gates stay outside, do not let them go inside. Mother- It is so, any problem has come in the child, as if there are any such things which are not understood by us, and then they speak upright in it. That you do not understand what we are talking about, this is the matter. They have a lot of cases too, now you have to see a man, see a man properly, they have to see millions. It is very crowded. Father- And if you ask anything, you speak, you become a doctor yourself, you speak like this.

In-depth Interview parents of deceased neonate-6

**1.1**	**Primary respondent (relation with the child)**	Mother, Father
**1.2**	**Age of the primary respondent**	Mother-20-25 years<br>Father-25-30 years
**1.3**	**Other members present**	None

2.1 Can you please let us know about the illness and events that led to death of your child?
Mother- It was Sunday, that day, the child was well all day, he had milk and slept, at 6-7 o'clock in the evening, baby had high fever. Father- He called me, then I came from the job, then I saw baby was crying a lot, and her body was very hot, after that we went with S Hospital. Mother- in morning we took her to hospital, after her admission there she got medicine and glucose after half an hour of medicine she opened her eyes, she was bit alright after getting medication. Then after that, she was shifted inside the ward. Then she was getting medicines, the doctor did not check her for the whole day that what problem she was having and he checked other children. She was getting medicines after half day passed doctor checked and said she is getting short of breathe that why we will have to put a pipe. We thought that if she is inserting pipe she will do well. When they inserted pipe, pipe got filled with blood, blood was coming from mouth. They went after inserting pipe, I went said a lot of blood is coming you can come and see that. Father - the blood was cleaned from the vacuum then more blood was coming. Mother- Then we asked why the blood is coming, so they said it is coming from the lungs, why it is coming was not told to us. Father- Did not tells the reason why the blood is coming, whether it was an infection or not they have read the report. Mother- I do not know how they inserted pipe we don't what happened to the lungs whether it hit the lungs.
Father – Blood should not come.
Mother –they inserted the tube after which blood came, they didn't told us about that. They inserted two tube one to clean her stomach, her stomach got enlarged, it was fine at home, after she had glucose some swelling appeared on stomach, second tube was inserted in her mouth. One child died in front of us. My baby was 14 day old. Other child died he was 10-12 year old, after that there was some fight in ward, they slapped the doctor. Then after that a child was killed in front of us, it was small. My baby girl was 14 days old. There was situation everybody was saying take your child from this hospital, here staff is not able to do anything. They give the medicine only and say if any problem, come to tell us. If we go to them, they didn't know that child is serious they should come and see the child. I said to my husband that go and call the doctor. They said child is serious they made us sign one paper after inserting the tube.

Hospitalization period

3 Now we would request you to tell us something about the period of hospitalization for your child?
<who inserted the pipe> Mother- there was one senior doctor and afterwards junior doctor came, senior doctor was telling junior doctor to insert tube and give injection. there was fight betwwen other patient and doctor, he slapped the doctor, he said you are keeping child for 2-3 days and there is no improvement, children keep dying there, the environment in ward was not good. we consulted one doctor that if we take our child to private hospital will he get proper oxygen through machine as here we were giving through ambu bag. they said its your wish, you can take to private hospital. then we went from S hospital to private hospital. there doctor saw the child and said baby condition is serious, baby had tube in his mouth. we took discharge from S hospital.
<how many day child stayed at S Hospital >father- we went the child for 2-3 days, we took discharge from S hospital and took the child to rainbow child hospital in malviya nagar, it was very costly hospital, then what could be done we again took the child to S hospital. mother- you have come with child with tube inside his mouth, child condition is serious, they said why you brought the child from S hospital. father- the child was not serious earlier.
<when did you come back to S Hospital> Mother- we took her back to S Hospital on the same day, after one and a half hours. Father- Within an hour or two, we took the ambulance to S Hospital. We had taken oxygen cylinder from private we had taken along with it. Earlier, at S Hospital they gave

LAMA as they said you taking your child by your known. Then when we went again they was not admitting the child in S hospital, they said go anywhere else. Mother- The child was very serious.
<how did S Hospital admit again> Father- After requesting a lot, I had requested for 45 minutes. The child was on a wheelchair, it was on ventilation.

4 Could you please describe your overall experience about the hospital care?

<what treatment they, after admitting the child again> Father- After admitting, they kept baby for 10 to 15 minutes inside Emergency, which is the new in S Hospital, then after that they sent back to ward 20-21. Mother- There was no machine they just kept baby. Father- earlier they were providing oxygen, on returning back they did not give oxygen, saying that this machine is not working, asked us to give oxygen by pressing Ambo bag, they told us to keep pressing, do well all night, till you do, the life of the child will remain, if you do not, we cannot do anything. Mother- we told many times to doctor to see our child.

5 Could you please describe your experience about communication by the various hospital staffs during hospital stay?

Father- we kept telling doctor to come and see our child she is having breathing problem, that my wife was having some problem, when we are vacumming the child we are not allowed to go anywhere.
Mother- they just used to say that they are coming, they are coming now. Their file work was not ending. Father- I did not know what file doctor had taken, they were noting something on it, don't know what she was doing it was more important to them they did not come to look at the child. Mother- at last they come and telling us that why didn't you call us, mean they don't know that the child is serious there, the child should be checked again and again, and they don't let more than two to go inside, I had an operation for delivery.
Father- my wife was there inside, it was her 1st delivery through operation, bouncer was not allowing me, my mother she is diabetic patient to go inside, not allowing any other patient, I thought my wife would have tried pressing bag now I should go inside to press ambu bag. I went down to give food to my mother till then I was pressing ambu bag, till then she was alive, after half an hour phone came that this has happened, I rushed back upstairs. Mother- when my baby stopped breathing then doctor came, earlier she didn't came even on calling. Father- after those 4-5 doctors gathered one by one. Mother- Later when after this happened they gathered, but no one came when my mother-in-law went many times to call doctor that child is having problem. They were just saying that they are coming, but no one came.
<after you took your child again, then when did she died>mother- we went back early. Father- she died at 2-2:30 at night, we got back to hospital at 12:30-1. Mother- it was dark when we reached back hospital. Father- at 2 in afternoon we took discharge from the hospital procedure took 1 hour, then we returned back to hospital at 4:30 in evening after one and half hour. Mother- it was 6-7in evening, we don't remember time.
<investigations>mother- test were conducted. Father- we didn't to investigation at S hospital, it was performed in MCD dispensary in DC, mother- all test were done before delivery. We were taking all treatment from there. Even at last they also performed something then referred to S hospital for delivery, at night we took ambulance and went to S hospital, even they didn't perform normal delivery, then I delivered the baby at S hospital through operation.
Discharge from S hospital after delivery- Father- It took one week. Mother-she was born on 3rd and I got discharge on 8th. Then after coming to home baby had fever, rash also appeared on her back, it was due to heat. We showed her in DCW, they gave ointments and medicine, then the rash was drying up, and they said that she got infection from S Hospital. They didn't tell how this infection happened.
<behavior of doctor or nurse> Father- behavior was not good in starting on admission, in last also itt was not good.
<what you did not find good> Mother- first they didn't allow anyone to come inside, I got operated during delivery of my child, I was not able to hold my child as I got stitches during delivery. My mother-in-law was inside, they were allowing just one member at a time, the child was very serious, yet they were not allowing . When the child was very serious in the last, then they called us both turn by turn. Secondly when we used to call the doctor, then didn't used to come, they used to come and and went away after seeing it. When the child is taking medicine through glucose, then she was breathing, it had been half a day, when they didn't check, then in half a day you are saying that the

child is not breathing. Well, when they put the tube the blood came from his mouth, they didn't explain why blood came from baby mouth no reason was told to us. It was not understood by anyone. father- as we reached hospital after one and half hour, baby treatment started, she got glucose, medicines, she was given breathing through ambu bag, after that after one and half hour they told us to sign on letter it was mentioned that your child is very serious and nothing can happen. Mother- baby only had fever. Father- they didn't tell us what the problem was or was there any infection. Directly after two hours of treatment, they said the girl will not be able to survive, they made me do signature on two papers, such a big letter was written over A 4 sheets and they got two papers signed by me, what I would do then. (This was when the first admit was done) mother- this all happened when we admitted for 1st time, we went after they inserted tube. Father- They had already broken the trust, then after that one child died, one more dead occurred of 11-year-old, one more baby died, they had fight with doctor. Then there was some fight between them. Then I started feeling like a fear, I thought it was better to get discharged from hospital.

<nurse behavior> Mother-nurse was all there. Father- the nurse was doing everything the doctor used to tell it to take and prepare the sample one was giving a bottle of glucose. Mother- everyone was taking blood sample. They didn't even know how to take properly as blood was falling from the tube. Father- He does not know how to take it, even the blood was coming out of hand. Then the blood was coming out of his hand then I took cotton and cleaned it with Dettol, after that blood stopped.

Death and post death period

6 Could you please tell us about the death and death declaration of your child?

Mother- i was there only, i called the doctor. father- i was with my sister and her husband, we were went down to bring food for my wife and mother, after that my wife called me from my mother phone to inform me.

<who declared about death> Mother- my baby was getting short of breath so I send my mother-in-law to call the doctor, then she went and called doctor. Then one was inserting pipe, and other one seeing that the child is running short of breath, yet she was still putting the pipe. After that baby was breathing less, there was no life left inside the child. Then they pressed her stomach still no breath came. Then after that, the doctor came at that time there was only one doctor at night time. No senior doctor came at that time. Father- when we went again the senior doctor was present, after that I don't know. My sister and mother used to get there treatment from S hospital. My sister told me this nurse was earlier a compounder at this hospital now she have become doctor. Mother- all were of small age. Father- out of those two were senior doctor there behavior was good, among ladies staff no behavior was good. One sir who was south indian came at time of death. Mother- when baby got expired then he came. Father- no he came earlier.

<what they told you> Mother – I went out when my husband came inside. What were they telling? (Mother asking father) Father- they didn't tell anything they gave Ambu and filled medicine with glucose. Mother- we bought ambu from chemist. Father- when I was discharging her they were not allowing us to take ambu, they said this ambu is government property you can not take it. First I rushed for getting ambulance and now for ambu, then i tried to get ambu from 5-6 chemist shop, then someone gave me contact number of person from where I could get ambu, then that person took 1100 rupees, otherwise it cost between 800-900 rupees.

<cost>father- only money ws spent on ambulance, no money was spent on treatment at S Hospital.

7 What was your and your family member reaction to the death of your child?

father- at that time my mother was present. mother- they left after telling that child is no more, they didnt give us anything in written, they directly said to take the child.

8 How was your experience about the procedure after death till handover of the body to you?

father- i said them to write on paper that we want to take the child, they didnt wrote anyhing. mother- when baby got expired it was there responsibility to write something, they just said you can take the child. Father- When the child expired after that they made me to do signature on paper, they said if you want death certificate you can come after one month and show this slip, I said you can keep this slip with you, I don't want as it is of no use. I signed at last.

<time taken to hand over body>mother- they said at that time only, my father and uncle was told imediately. father-death happened at 12:30, after one and half hour we left from the hospital.

9 Who all from your family and community/neighbourhood were present at the hospital and how they supported you during this period?

father- at hospital, my sister, her husband, my mother. after sometime my father and uncle came. uncle lives near to us.

10 What all rituals and procedures did you follow after death of your child?

Mother- we didn’t child to home, we took directly from hospital. We left from there at 3-4 pm, it was already morning. <Where were you till four o'clock> Father- We were told to go to near Kalkaji temple and bury the child we were sitting for quite some time till 4 o'clock , Outside the Kalkaji crematorium, which is in front of the Kalkaji temple. When we were sitting there outside crematorium one truck came, and he informed us that here burial doesn’t take place, you can go to kalandi kunj. You can bury on the banks of Yamuna river. Then we went there. Father- we buried the child, all procedure was done till 6 in morning and till 7 we reached home. I went with my father, uncle and two friends. Father- we bathed the child feet from taking water from Yamuna river, the burial person was telling us to perform ritual.

Mother- naming ceremony happen on 12th day, we happened her vanshika, we brought teddy for her. After so many years, a little baby came in the house, she was the first child.

<rituals>mother-we didnt do anything in case of child. we only did her naming ceremony on 12th day after she was born.

11 How did you, your spouse (wife/husband) and immediate family members cope with the difficult phase?

father- we are supporting each-other, try to remain happy but still remembers our child. mother- i started doing little bit household work after my stitches were cut.

12 Is/was there any change in the interpersonal relationships between you and your spouse and other family members after the event?

father-there is no change in relationship, we support each other and console eachothe, what else we could do.

13 How did the family members support you and your wife during the difficult phase?

mother- all family memember supported and consoled.

14 What were the reactions/ counselling and suggestions given by the relatives/neighbours/community after the episode?

Father- neighbors says they didnt believe how suddenly all this happened. mother- our baby was fine, she was 14-15 days old, but all used to say that she must be 3 months old. (mother-father showing pictures of the child in their phone) mother- this is picture of child on day she had fever, baby was vaccinated on that day, my sister-in-law came to see her as she didn’t came on naming ceremony of the child. No one could tell she was 14-15 days old child. Father- her birth weight was 2kg and 700 grams, she was delivered by operation.

15 Do you think and/or discuss with your spouse/ family member(s) about the death and related events and blame someone/something or feel guilty for the same?

Mother- We ask this to the doctor that what happened when tube was inserted, why blood came does the tube inserted hurt the lungs, what happened. We tell everyone these things, everyone says this was the fault of the doctor, because of the doctors this all happened and blood came. Father- On the day when the baby was born, she gave smile at the same time. She was born in the morning at 2.30 am, they were not letting go even for next day, visiting hour time was from 4-6, then I went, she was smiling. Mother- When she was born she had jaundice she was kept in light for 2-3 days at S Hospital after 3-4 days her jaundice was cured. Father- After the operation, the child was also discharged with mother. They didn’t discharge until the mother and child are well. When the child was well, they discharged with mother. Father- baby had fungal pimples after she was born, in stating it was only 1-2 rash after that it become pimple in 2-3 days. I asked what they were doing for that, then then the doctor did not give any response. They said, let the jaundice be cured, then when the jaundice is cured, then your wife will be discharged, now the new patient who comes will share the bed with your wife. My wife had operation for her delivery, how can they keep two patients on same bed, they said now 4 days have passed the stitches must have dried now they can keep other patient with her. What if accidently she got hit by her, then problem could occur for both. Mother- It was happening. Father- Fungus infection was present, they didn’t even see it, they just cured jaundice, and then the doctor discharged after curing jaundice. Mother – I had big operation, after that, two patients in one bed, and our child is also with us, how much is the size of the bed, it was small bed so a lot of problem was there. Father- Before our child, a boy had jaundice inside the ward, after that, our baby got jaundice

from him, after that the infection started spreading and it started happening in other children as well, so it started coming next to the next side. In the second ward, it was a machine inside which 2-3 child could be kept, but 5 children were kept in that lighted machine. It was so congested, what should I tell you. I thought it was bad experience at S Hospital. It was going to be very bad with us, I saw baby face again and again.

16 When did father and mother (if working) returned to work after the death of the child?

Father- I went after a week at work, I am in sales job at Adidas Company.

17 How your and your family's life has changed after the death of the child?

Father- few change like some negligence happened from our side as not keeping cleanliness, holding child without using sanitizer, all this was not told by anybody. i used to on job, at hospital they told infection has happened, whosoever used to come hold the child like that only, maybe this was the reason. we should remain careful in future, the report is with doctor, that after seeing that report after 2 hours senior doctor made us sign letter. <any substance abuse>father- no remained same as i was earlier. <next pregnancy>father- doctor told us to plan next pregnancy minimum after 3 years.

Knowing cause of death in detail- Autopsy and MITS

18 What was the cause of death of your child and what is your perception about the value of knowing the cause?

Father- We did not know the reason for the death of the child, we were just told that there was an infection, what was the infection was not told. <value of knowing the reason> Father- What I understand more than my child is, was not more than my child.

19 In your view, what more could have been done to know the exact cause of death and the reasons causing the disease?

father- you can go to S Hospital or send someone there you could see what happens there.

20 Are you aware of any method for identifying the exact cause of death?

Father- Yes, I had some information. Baby was not vaccinated which is given at time of birth, I have doubt I think BCG. Mother- baby was given that vaccine. Father- What could it be other than that, or a test that they had in the last blood test, after that we had taken the child after discharge, the report was still there, we have not taken that report Maybe something would have written in it.

21 What are your views about the autopsy/ post-mortem for identifying the cause of death?

Father- At that time it came in my mind that there would have been a post mortem or something like that, a lot of things and things are going on in my mind at that time, but could not be done, as we are middle class family problem also occur simultaneously. <necessity for post-mortem> Father- I would have gone for post-mortem to know what was the problem to my child or who was responsible for death of child. <religious barrier for post mortem> Mother-post mortem does not happen in the child done. Father-in elder there is no such problem.

22 What are your views about collection of the tissue (like done for biopsy to diagnose diseases) and body fluid samples for identifying the cause of death?

Father- i would have agreed for this technique, it is a good technique.

23 How parents and family members can be approached for the tissue and fluid sampling?

<who should approach> Father- something could happen if we would have tried. . <whom they should approach> Father- I could have done all these things my father also works in the hospital, is the Eye Hospital and the RC Hospital branch in MN, A Hospital, it is also for child. So I could find out some things from there too, if I tried to get it done. But did not do it, child was not there. Father- all this would have happen in S hospital? At that time there was no one, I console my wife, mother, because they lived with the child the most, the mother did not eat for at least a week. I was so upset, all these things were also necessary together. <how should they explain about technique> Father- by whatever way we would understand.

24 In your view, what factors are important for acceptance of the tissue sampling by the parents and family members?

Decision making dynamics

25 In routine day to day operation who takes decision about the major activities (purchases, treatment, spending money) about the family?

Father- My wife and mother, both of them take the decision, something has to be brought, so both of them do it. and discuss things together. <decision about treatment> Father- for small problem we go to muskan clinic near ISKCON temple. We get better by his medicine in day or two.

26 During the hospital stay who discussed with the doctors/ nurses to make the decision(s) about treatment and other procedures.

Father- at hospital my wife was taking decision, she said proper care of baby is not happening, take baby to private hospital, then we went, then we thought taking baby to lady harding hospital in CP. then my father had some reference at ajay hospital then we went there, there they said it is case of S hospital no one will take, then someone suggested rambo hospital but said it will cost rupees 4-5 lakhs, due to reference they said you can get treatment in 2.80 lakh, you can deposit rupees 80 thousand now and there is 10% guarantee your child can survive.

27 After death of your child, who decided about the cremation and other post-death activities and rituals?

Father – my uncle performed all the rituals, he dug the hole in mud to great extent after that I dug that, then I put baby inside it. After that we put some incense sticks. My uncle had some knowledge regarding this as it has happened to him. My father also went with us. We don't have any knowledge as it never happened with us, it was 1st time.

28 Now, we are completing the interaction/interview. Would you like to add anything?

Father- this was from our side, we have shared with you.

In-depth Interview parents of deceased neonate-7

**1.1**	**Primary respondent (relation with the child)**	Mother, mother-in-law
**1.2**	**Age of the primary respondent**	Mother-35-40 years<br>Father-40-45 years
**1.3**	**Other members present**	Mother-in-law

Context- Mother was not able to speak because of the health issue, so the mother-in-law was speaking on her behalf, Mother was adding to some questions. Age of her husband is 40years, mother got pregnant after 15 years. She has continuous abortion.

Events that led to death of the child

2. Can you please let us know about the illness and events that led to death of your child?

Mother-in-law-Baby was delivered at F hospital, my daughter in law was admitted there, she had BP, so she was admitted earlier, she delivered the baby at 6 months, she was not well, then doctor said there is delay of 15 days, so they said to operate, they said if we will deliver by normal delivery then there can be danger to child, then I said there should be no danger you can operate, so they delivered by operation. Doctor said child is fine but is weak. Since conception a lot of money has been spent. Then we thought to go to S Hospital, as doctor in fortis was not discharging her, at S Hospital my brother-in-law son knew some doctor at S Hospital, he talked to doctor and he said to bring baby to Hospital.

Mother- 6 months could not be completed even one day was left. They were saying child is weak, we will keep child in ICU for 3 months and we have to admit baby in nursery for 3 months, we were not able to bear the expenses of three months we had already spent 7 lakh rupees, and also expenses for my operation, spent 13 days in hospital. I used to get 3 injections for baby survival.

Hospitalization period

3. Now we would request you to tell us something about the period of hospitalization for your child?

Mother-in-law- we went at 6,on tuesday my son had off, we went at 6 in evening baby was there in hospital for whole night, i was there in evening, she was removing oxygen pipe again and again, even moving her hands and feet, my son stayed at night, baby was becoming weak, was turning black, then in morning i came to hospital at 9, and she expired at 12 in night.

4. Could you please describe your overall experience about the hospital care?

<about treatment> they were giving fine treatment.

<about investigation> it was fine.

<about cost> no money was spent in S hospital.

5. Could you please describe your experience about communication by the various hospital staffs during hospital stay?

<about behaviour HCP> It was good.

<communication of HCP> They were saying that we had already said that the child will not survive, they told her survival is difficult, we are keeping her and looking after her, if she survives that's good.

<frequency of communication> they used to tell us on time to time, doctor and nurse came to check baby, lady doctor was very good.

<completeness of information> they used to tell us whole thing, that her parts are not fully developed.

Death and post death period

6. Could you please tell us about the death and death declaration of your child?

During death my son and son-in-law was there at S hospital, i came back home at 9 in night, my son called doctor when he saw baby was not breathing, then doctor came and said baby is no more.

<skills of communicator>

nothing from doctor side, we were thinking if baby was kept in nursery, then she would have survived, she was kept in ward no.21, if i remember.

7. What was your and your family member reaction to the death of your child?

When baby goes away, mother will weep what the mother will do, at that time everyone felt equally bad, we also did not feel good, one member was small, even though he is older, and we felt bad, we were also crying.

8. How was your experience about the procedure after death till handover of the body to you?

Yes only some paper work was done and they discharged them, no time was wasted.

9. Who all from your family and community/neighbourhood were present at the hospital and how they supported you during this period?

Post death procedure and coping mechanism

10. What all rituals and procedures did you follow after death of your child?

We brought home, nothing happened at home, then we cremate with wood, not buried, we cremated.

<body preparation>

no bathing took place, as she was very small. 4-5 people went in car to cremate her in chirag delhi.

Mother- she was not alive what is to be done.

<about timing>

They came from hospital at night, they did not go at night we took the girl in the morning we do not burn at night. My son, son-in-law his friends went for cremation.

Mother- They came back in half an hour, we do not cremate at night.

11. How did you, your spouse (wife/husband) and immediate family members cope with the difficult phase?

We try to remain happy.

<change in physical state>

She is still not doing household work, as her operation was done, then she fell ill and got a shock, she has BP problem. Don’t know what happened to her last week, she suddenly had some shock after that her voice become weak, she had BP problem since 1st pregnancy, there was no problem in 1st child she was delivered normal.

12. Is/was there any change in the interpersonal relationships between you and your spouse and other family members after the event?

No there is no problem between by son and his wife. he cares for her, today also they had to go to doctor but came late at night from marriage so they will go to doctor next day, she is getting her treatment from M hospital.

13. How did the family members support you and your wife during the difficult phase?

Relatives give support, yes they helped even with money, we got support from daughter-in-law side and relatives from baby father side.

14. What were the reactions/ counselling and suggestions given by relatives/neighbours/ community after the episode?

Yes, the relatives came for support everyone came and what could they have done, no stone unturned on our behalf, now whatever will be the will of God, it was not ours, she was 15 days old baby and she died after that.

15. Do you think and/or discuss with your spouse/ family member(s) about the death and related events and blame someone/something or feel guilty for the same?

We do not blame anyone because the girl was like this, she was weak what could we do? She was getting weary and weak for 13 days, doctor said the weight is increasing, one gram of it has increased, he was speaking like this, but there was no increase in weight, she had a weight of 450 grams. And the mother had also become ill at the time of pregnancy, she used to get 3 injections, it used to take two daily and it is used for growth of the baby, but no growth happened.

16. When did father and mother (if working) returned to work after the death of the child?

Baby father returned to work after 2-3 days, he is property dealer.

17.   How your and your family’s life has changed after the death of the child?

No there is no change in mother life.

Knowing cause of death in detail- Autopsy and MITS

18. What was the cause of death of your child and what is your perception about the value of knowing the cause?

Mother-in-law- We know she would not survive she was very weak, even doctor used to say this, her brain was not developed properly.

Mother- What to know

19. In your view, what more could have been done to know the exact cause of death and the reasons causing the disease?
If there is something, then nothing has happened to our girl, no we do not know anything like this.
20. Are you aware of any method for identifying the exact cause of death?
<about Postmortem>
we get to know about cause of death, we do postmortem to know how someone died.
21. What are your views about the autopsy/ post-mortem for identifying the cause of death?
No, we do not know this, it has not happened to anyone.
22. What are your views about collection of the tissue (like done for biopsy to diagnose diseases) and body fluid samples for identifying the cause of death?
It is good technique, it does not happen on our baby, we don't know anything about this, it will be time saving and also there will no tearing of body.
23. How parents and family members can be approached for the tissue and fluid sampling?
<whom> it should be explained to child mother and father.
<who should approach> Doctor should tell about this.
<how explain MITS> explain as procedure will take place.
24. In your view, what factors are important for acceptance of the tissue sampling by the parents and family members?
Decision making dynamics
25. In routine day to day operation who takes decision about the major activities (purchases, treatment, spending money) about the family?
Mother-in-law- Decision are taken by me and son, we are three person at home, we dont involve relatives in decision making.
26. During the hospital stay who discussed with the doctors/ nurses to make the decision(s) about treatment and other procedures.
Mother-in-law- I took the decision at that time, a lot of money was spent at that time. We knew baby was very weak, then we called at S Hospital they said bring the child there. We only think if we could have got nursery in S Hospital she could have survived.
27. After death of your child, who decided about the cremation and other post-death activities and rituals?
I took that decision about cremation, we don't bury child, because if we bury child dog can pull the child, so cremation is practiced. We didn't consult anyone it was my decision.
Summarization
28. Now, we are completing the interaction/interview. Would you like to add anything?
Mother-in-law- no we don't blame anyone, when it was not ours so what can we do. Our daughter-in-law has fallen I someone eyes, we went for that someone told about it. The first one was only 7 months old there will be a child to play.
The girl was born, 6-month-old was finished in the stomach, then a 7-month-old, at that time there was not much information, at that time I went to G hospital, when 2nd one got expired then all the tests were done and there, I had a problem in kidney so they are confused because of this that BP increases and then took a chance after many years, this is the third time. It is done, whatever God will give. Either boy or girl it should be fine. BP medicine is going on right now.

In-depth Interview parents of deceased neonate-9

**1.1**	**Primary respondent (relation with the child)**	Father, Mother
**1.2**	**Age of the primary respondent**	Mother- 20-25 years<br>Father- 25-30 years
**1.3**	**Other members present**	Sister-in-law

The mother of the child was staying at her mother place, at the time of interview father, mother and sister-in-law of the mother was present. The delivery of the child took place at home.
Sister-in-law: He is son-in-law in our relationship his mother has gone to the village. We had arrived from our village, we already knew about the situation, the child was upstairs, there was a doctor in the room, there was a babysitter with him, the child did not cry even after normal delivery, his hands were closed, and the doctor said, the condition getting worse, move to S hospital, we immediately took it by three-wheeler. On arrival at hospital, the nurse started shouting like you did not know what could be happen, like baby may not be able to speak, may be mentally retard.
Father: This was our first baby we all are living in a rented flat

2.1 Can you please let us know about the illness and events that led to death of your child?

Sister-in-law: The baby delivered in home, a doctor with nurse came to the home for assisting. They tried by pushing and pressing the stomach. Enough time they had spent in this process. They came around 8-9 pm and baby delivered at midnight 12 o’clock. At that time, child was normal, but did not cry. The doctor tried to cry the baby by tapping her legs and at back side. Her hands were tightly closed. After some time, they asked to move S hospital. We had hire a 3 wheeler for same and reached at S hospital. Here, doctor initially refused to admit but we had requested as the child was critical. Then, they admit and brief about the condition. The baby was in critical condition, having episodes of seizures.

Hospitalization period

3 Now we would request you to tell us something about the period of hospitalization for your child?

Father: Admitted in three ways at night, after that the doctor told that she was seen as such, she had to admit at night, left at 12 o'clock at night and reached 12.30, then after inquiry 2.30- Admitted at 3 o'clock. After that, the child expires at 7-8 pm on 5th. For the first two days, they were just giving glucose, they did not put any new formula, but they just added glucose and they used to press the continue which pressurizes the oxygen, which was breathing and air was going on inside the pump. Sister-in-law, but her fist did not open, her fist so tight means how many planks are there in it, we were not able to open it openly, so tight it was closed, I did not know what had happened to her.

4 Could you please describe your overall experience about the hospital care?

Father: We stayed with him for the whole night, the doctors were not even listening, once he gave it at 10 o'clock and his seat was changed in about four hours, he gave it at 10 o'clock, then someone would come around 3 o'clock, He filled the entry and came to give medicines at 2 o'clock and gave the time of 2 o'clock and came at 4 o'clock, there is a direct 3-hour gap. Entered that we came to give medicine thrice while giving the medicine once in the beginning, after that we slept for 2 hours and talked about it, there were many children there and if the condition is disturbed then it will not see the condition, it will die anyway They say this in advance, the doctor, they speak just like that, they do not even give it, they will die, where did you create it, they did it, they said it as soon as they left, this behavior is not getting right , Will not be said anywhere, it is said right.
Bhabhi- We said Doctor is not opening this mouth. Father- we have said that you do not refer, do not even refer, just take it with your surety of your responsibility, we will not write the transfer. If you refer, then we can get admissions anywhere or not. Yes, I said to them, then said, take away from your will that I am taking on my responsibility, if I write this, then it is written proof. Let's do. Everyone demanded that till now they were getting treatment proof. Referee gets admitted, if he takes Serious at his own will, the child will not put his hand. We became very strong and could not be moved.

5 Could you please describe your experience about communication by the various hospital staffs during hospital stay?

Father- They did not listen to us, and will give medicines twice and will offer four times that we are giving medicines so many times. Sister-in-law: Now as the child is vomiting, the race is going on, the sister is vomiting, look at the child, but you did not listen. Father- One of them was a learner who was giving medicine, there is a doctor with him, so according to his own practice, the medicine was to be used. He was applying glucose in whatever injections were put into it. A bottle of glucose was kept in it and there was a mosquito in it, we saw it was going to be put in the glucose. Then we said that it is a fishmonger, otherwise it will kill more children then it threw that glucose and the other one gave glucose. When it is open like this, the mosquito will not go in it was not known that even the mosquitos were inside.

Father: Big doctors used to come in the day, they have limited time to visit their junior doctor only talks comfortably in presence of seniors otherwise they don't entertain us. Even we cannot tell the problem. Condition of child

Father: They were talking that only 1% survival rate in this case.

who communicated the information: One lady doctor with stethoscope usually visit and one lady must be sister(nurse) who gave the medicines, having 20-22 years of age. She must be newly recruited.

<nurse behavior> Sister in Law: Nurse first insert cannula in hand of baby and told to hold that cannula. It was slipped many times and she told to take another cannula of 150 from market.

Father: Nurse told that you have tempered the cannula and need to purchase another one from market. Otherwise you need to hold this cannula manually whole time.

<investigations>Father: we had conducted all the prescribed test. At same day, we have done blood test, ultrasound and many more. Yes, another test, MRI was also done. An X Ray was done from another building of hospital. We have collected the report of same from another building.

<cost>There were no as such expenses had happened inside hospital. My wife and me, only were present there in hospital.

Death and post death period

6 Could you please tell us about the death and death declaration of your child?

Father: we were in the hospital and hold Ambu bag. We called a doctor to see the condition. She moves her legs, respond as some one touch her body. Initially, she moves her tongue outside. Sister in Law also feed the milk. Earlier the baby condition was in control but at 4th day in the night, her legs movement had stopped and condition becoming worse gradually. Nearly. 6-7 am, she stopped breathing and dead. then we called a doctor to check the condition. Doctor check her by stethoscope and declare dead. Earlier, at 4 am, irregular breathing was reported by doctor and at 6-7 am she expired.

<sympathy>From two days before death, urination and defecation were stopped. So the hospital staff told, all these were accumulated inside the body and that lead to stop the blood flow.

<doctor told any cause/reason> Father: according to doctor, at the time of born, contaminated water was entered in the stomach. We been delayed in hospitalization also. We had arrived at hospital near 12 PM but admit at 2 – 2:30 PM. So the contaminated water might be act as a poison and spread into entire body that could be reason of death. Another side, hospital was not good. They don't have equipment, even they were managing ICU without oxygen. The attendant like me, had to manage all things like holding cannula in hand every time and it was very risky

7 What was your and your family member reaction to the death of your child?

Sister in Law: Then what! everyone is drowned in sorrow, someone's tears come out. Sister-in-law (child mother), the condition was worsened, the child who was given the filling, this was like that, due to heavy crowd and not having space, she(mother) stand and roam around hospital since three days. It will be broken on the ground, so now it hurts its waist. Father- As soon as the baby is born, it remained standing for 3-5 days, after that she was suffering from severe backache. one should be rested in this condition. Sister-in-law, she remained standing day and night last time she slept on the ground near hospital for a while.

Father- We were in the H block, on the third floor.

Sister-in-law: Our baby was kept in a room surrounded by glass.

Father- Before our child, 5-6 children were dead, due to the same cause i.e.: contaminated water

8 How was your experience about the procedure after death till handover of the body to you?

<time>Father: then, the hospital staff said to sign on a paper and take stamp on same mentioning date of expiry and handover the dead body.

Then every step completed quickly, they did all formalities in short time and give a duplicate copy of handover of dead body. We return from there after getting pass slip. Downstairs, our Mom and Dad were waiting. Then we left the hospital at 7 pm. with all family, we had stepped to our flat in a rented Auto.

9 Who all from your family and community/neighbourhood were present at the hospital and how they supported you during this period?

Father: we both (my wife and me) were there. Wife: Father in law and mother in law were also there.

10 What all rituals and procedures did you follow after death of your child?

Father: we came to my maternal house. We have done burial procedure near a crematorium near maternal house. At there, the ritual starts after taking a bath of each member and dead baby. The dead body was rapped with flower and buried. Before that, eye liner and oil applied on the dead body so everything happen in write manner.

11 How did you, your spouse (wife/husband) and immediate family members cope with the difficult phase?

Father: Slowly-slowly, we been familiar with this situation and try to understand the situation. As we are practical and educated. Everyone in world has some pain and no one is satisfied. Everyone has different kind of problem. In our case, when she is more, we all have to adjust in this situation.

Mother: I use to see her photo and weeping till now. I have not emerged completely form that tragedy.

12 Is/was there any change in the interpersonal relationships between you and your spouse and other family members after the event?

Just had a regret that if you had taken it to private, then there would have been good arrangement there. Just like they just treated our baby with limited or partial equipment in S hospital, after that they had no other way or formula to drain the contaminated water from stomach.

Mother- See the photo (in Mobile), the pipe that is visible that was inserted in mouth.

Father- In the name of treatment, they just inserted a Ambu bag and tell us to hold it. What could be expected from that incomplete treatment. We just keep pushing the Ambu bag and her stomach become bulge.

13 How did the family members support you and your wife during the difficult phase?

Father: At the time of death, my mother in law, father in law, mother and father were there at my home for moral support.

14 What were the reactions/ counselling and suggestions given by the relatives/neighbours/community after the episode?

Father- See, what he is educated, he has become aged/experienced but he has no idea. We thought that nothing would be ahead of his treatment. How will the child get this benefit or not? If we were looking at their treatment, then we already knew that it is not right to be here and that they are not writing the referrer, we cannot go to a big hospitalist in private. Then we also thought as long as there is breath, show it here. People in the neighbourhood said that you lived in the Kannur district, why not consulted there, why you took it there, people all around, has their own opinion. Sister-in-law, when a person goes away, many people give their brains, that if they do not move here, if they move here, it would have been right, the child would have been right if they had stayed at home, at first no one said anything. The hospital was bad.

Father- It was big but it is not for small children, means that it is suitable for treatment of major accident and other youngh and old age related issue.

Sister-in-law: they did not care so much there. We had not experience to select a write hospital

15 Do you think and/or discuss with your spouse/ family member(s) about the death and related events and blame someone/something or feel guilty for the same?

Father- We came to know on the third day that the child will not be cured with the treatment here, it will be over, this is what we said to him that she can be cured if we visited private hospital, it can be improved there, and they could treat with respect. Here, in the name of treatment, they just infused glucose. And the infused pipe also been detached after some interval. Who can we expect that some medicine infused with this disconnected cannula.

Father- What do we blame, consider ourselves, that now it is a matter of regret that we would have stayed at home or admitted to Kannur.

Sister-in-law: It could be better from here, if we gave desi treatment from our village. They did it from leaf of Jamun.

Father- The child was not weak, everyone in the ward was thin this child was healthy. I think it was not treated properly. It is with us that now we will never be taken there and advised others to stop visiting S Hospital. We will say do not take it there. There is no proper treatment. According to me, the doctor is not taught well, just comes with sister and new doctor to visit the ward, there is no good treatment there. Mother - Had asked about blood too, there was no lack of blood. Father- If there is a lack of blood then we can make the blood come out. Put aside, let the child go, just as much as it will be. There was no feeling that blood was coming out from where the canula was planted. Sister-in-law treatment was not being done right there, it is just this. We said that I was agreeing to leave the child. He was walking hand and foot and was fed milk, honey is eating everything.

Father- Gradually the dirty water kept increasing into the baby's stomach. The lungs got stuck, the movement of hands and feet stopped, when the dirty water is gone, it will continue to grow, it is not exhausted, it did not have any facility to get out. Some children died there, some of whom had 4-5 people, they had arranged oxygen from outside, the child was put oxygen and shown somewhere private.

16 When did father and mother (if working) returned to work after the death of the child?

Father: Baby was expired at 5th of month and father and mother went to their home after 5-6 days. They move there for the job and also rented a flat there. Bhabhi return yesterday after 10 days. She also residing at Noida flat

17 How your and your family's life has changed after the death of the child?

Father: There is sadness in everywhere and in every activity. Whenever anyone passed sadness over there.

<any substance abuse> No! The life gradually moves on the path as it was earlier. We are trying to resume the previous life style.

Knowing cause of death in detail- Autopsy and MITS

18 What was the cause of death of your child and what is your perception about the value of knowing the cause?

Father: They asked the cause of death in the beginning as well as contaminated water enters in the stomach and due to that the infection spread across the entire body and that why she passes away at 5th of this month.

<perception about the value of knowing cause>

Mother: you tell the reason of death; it would be helpful for us in next case. We knew that the treatment was not up to mark. She needed special treatment so the contaminated water/liquid could be drained from stomach. They should give some injection and should use cannula to drain the infectious. I hope in private hospital; all this could be happening.

19 In your view, what more could have been done to know the exact cause of death and the reasons causing the disease?

Father: As per our understanding, treatment was not proper. They just hanging Glucose in the name of treatment and make 3-4 times entry of same. If they visited frequently, the result could be different. She may be live.

20 Are you aware of any method for identifying the exact cause of death?

Sister in Law: Post-mortem was not done in our case. The problem was identified through x ray. Black spot over chest area was seen in x ray. They did not cure that black spot, and the case become complicated.

21 What are your views about the autopsy/ post-mortem for identifying the cause of death?

Father: As every incidence had happen in front of my eyes, I think post-mortem was not required. It would be required if the death was happened by poison or any suspicious reason or had a history of any rival with the doctor.

<religious aspect>

Father: No! we told the details from baby birth to death including all incidence like when we went hospital, when we get admitted, relieved etc.

Mother: Post mortem is not required in baby case cut/incision is not done in baby case.

22 What are your views about collection of the tissue (like done for biopsy to diagnose diseases) and body fluid samples for identifying the cause of death?

Father: If there were any provision like this, we may opt this. Even after death, one could able to know the reason of death, it is always a good option and everyone opt this option.

23 How parents and family members can be approached for the tissue and fluid sampling?
We were all together, so if anyone tells through the phone about this. Dead body was already there at the time of death so one can come and collect the sample.
<who should approach>
Any hospital staff may be detail about this, usually doctor or staff nurse explain this type of information. Obviously, it would not reliable if the information comes through patient side or from their relative side.
<how should they explain about tissue sampling>
It would be better to explain the process through mobile, a short video related to this via mobile.
<what time they should approach>
Immediately after death, it would better to do this process. May be on being able to get related information from the doctor like which baby/child expired and their other details. The relative and patient who are educated and wise must interest to know the detail reason of death.
Form this one can explain and share the cause of death with relative and all will know the area of ignorance that lead to death of baby. I think, everyone who is educated, will convinced/agreed for this test. Anyway, while treatment more than 50 times injections has been given to patient, another one injection after death wills not a matter of concern. Everyone will agree for this and will help in further plan.
24 In your view, what factors are important for acceptance of the tissue sampling by the parents and family members?
Father: This test would detail the exact cause of death so what type of ignorance has happened can be detailed out. This would also detail that what exactly could not be controlled in this case.
Decision making dynamics
25 In routine day to day operation who takes decision about the major activities (purchases, treatment, spending money) about the family?
Father: We are living here, so all the related decisions have to be taken by us. Like ration, accommodation etc.
In treatment case also, based on last experience and knowledge about which doctor would be better in this case etc. In minor complication, we usually visit local private hospital. Like there is private hospital "A Hospital'', my wife visits there in minor medical consultation
26 During the hospital stay who discussed with the doctors/ nurses to make the decision(s) about treatment and other procedures.
Had got the admission done, treatment was done and it was already guessed that the child would not be cured, and if we tried to refer, we could show the improvement elsewhere, maybe by spending 20-50 thousand, Maybe the child would have been correct, it would have been good, but they did not write the referral, and our relatives who had come also told that other specialized hospital would not take any such admission with referral from here, If they referred, our baby get special treatment there. There is a treatment of all type happening here, everyone has to see, there (in child hospital) only have to see the baby, there is a baby hospital and one is only a children's hospital, that is Where there will be a hospital with children only.
Despite all treating all one has to see only special cases of mother and child. That system would be better. Here in S Hospital, the doctors have to see every type of complication.
<any contact over phone>
Father: we have seen the baby and realized that this is a serious case and would not be cured. We had called my cousin brother. He came at hospital and tell that until we get a referral card from here no one is going to entertain us.
27 After death of your child, who decided about the cremation and other post-death activities and rituals?
Mother: Everyone from our family, were there at hospital.
Sister in Law: The other family member who were at home, prepare lunch for us and bring at hospital.
28 Now, we are completing the interaction/interview. Would you like to add anything?
Father: What to say for this! There is no any scheme from Indian Govt.
Mother: Don't know why baby has this much issue. Someone has cleft lip, someone has blocked anal passage. I have seen many children in S Hospital had severed medical issues and were admitted at there. In a small space, they had kept 3-4 children. There was no space for treatment.

In-depth Interview parents of deceased neonate-9

**1.1**	**Primary respondent (relation with the child)**	Mother, father
**1.2**	**Age of the primary respondent**	Mother-20-25 years<br>Father-20-25 years
**1.3**	**Other members present**	None

Events that led to death of the child

2. Can you please let us know about the illness and events that led to death of your child?

Had taken to S hospital for delivery, delivery took place in S hospital itself, discharged after 24 hours of delivery from hospital. Come back to home after discharge from hospital, keep child here for 8 days, 8 days later, the child latrines and does it again. After 5 days child stopped doing latrine, so will took the children to S hospital where delivery happened. Child was drinking milk and all (M)) child was drinking milk, child had diarrhoea (F). Taken there, they said go to S hospital you are not able to pay here (M). Previously we had taken to SJ hospital. Said there, bring 10 thousand rupees, will have to keep child for 20 days in hospital, it was 9 o'clock at night (F). So from there reach to S hospital, went to hospital at 9'o clock at night where doctor called me, I told the entire thing and told that the child din not defecate (M). Doctor also said that child have gastric problem, he did some treatment by putting pipe and send us to room number 18.

Hospitalization period

3. Now we would request you to tell us something about the period of hospitalization for your child?

<duration of stay> I, my wife and my uncle stayed for 8 days in Hospital.

<course of illness> No, not this all, will get well, Nothing, will tell them all think will be ok.

4. Could you please describe your overall experience about the hospital care?

<about treatment> told nothing, the blood kept coming out, they used to say it's okay and used to check, gives medicines, yes drip continued for 8 days, for 8 days, I was there for care, my wife and my brother also (M) we 4 people are there for care, I stayed at night (F).

<about investigation> Yes Check-up happened, nothing was in report, they told nothing, when I asked they told that all things would be fine.(F)

<about cost> stayed in S hospital for 8 days, expenses are about to 1000-15000, No-No, medicine was provided from inside from the hospital only, also took medicine from collage centre.

5. Could you please describe your experience about communication by the various hospital staffs during hospital stay?

<behaviour of HCP>

All are doing good, all nurses are new they are under training because when child specialist doctor come scold them a lot, doctor told them that what medicine are you giving, what is this, they all are new, Senior doctor told before two days of death of child that this child won't survive, doctor told before two days that poison has spread in the body, child won't survive, yes said that breath is not running, drive the pump, this thing told by the senior doctor (F) told that poison spread over the body it will need a needle (M). when child come earlier then he was okay, told that child is breathing, will have to did operation, I asked that I need to call all mother and father, if not survive then what will do, need to did operation, So had to do operation at same place (F). Then Operation took place (M), yes during that 8 days only, after operation doctor told that child become normal now but after that he told that now child will not survive (F).

<about behaviour of HCP> Behaviour of staff was good, all of them were good in term of behaviour, they take care timely, why should I lie.

<communication about treatment> Did not tell, did not tell anyone, they told that child is small, can become ill at any time. They told about treatment, idea was all right, stayed there for 8 days, suppose child was there but no one care, which medicine is given. Senior doctor told, one child specialist doctor comes at 12 o'clock at night he do, he is old but other doctors are new. (F)

Death and post death period

6. Could you please tell us about the death and death declaration of your child?

<cause of death explained> No didn't tell, I was at hospital on that time, child was dead at 6 O' clock at morning, after death doctor inject needle, still no body said that child was dead.
<who declared> No one told, he had Nimboi in his mouth, I started weeping and said that child was dead then free it but after that no one was freeing the child, when I called them then he asked for signature on various documents and left the body, no didn't open, yes it was revealed that child is no more (M). by seeing face it was knowing, take it 1 or 1 and half hour, if died at 6 o' clock then they give us at 7 o' clock, doctor said at 6 O' clock on that time, colour was changed, child was not breathing, body became cold, then go to doctor to give information (F). Doctor came and changes the piece that was in mouth.
7. What was your and your family member reaction to the death of your child?
<mother reaction> I started weeping.
<father reaction> No Reaction, returned to home from hospital after the child died. But my wife started weeping.
<did someone try to console you> No one when child was died no doctor was there.
8. How was your experience about the procedure after death till handover of the body to you?
<about documentation> Mother was signed
<time taken> One and half hour.
9. Who all from your family and community/neighbourhood were present at the hospital and how they supported you during this period?
Me, my wife and my Uncle was there in hospital, they supported a lot.
Post death procedure and coping mechanism
10. What all rituals and procedures did you follow after death of your child?
Reached elder brothers' home at 11 O' clock, 20 to 25 relatives come there and did the last rituals bury the body approx. 5:00 PM
<body preparation> Child body was buried by maternal uncle; 40-50 people were there during this.
11. How did you, your spouse (wife/husband) and immediate family members cope with the difficult phase?
We need to control our self, returned and start living and doing business.
12. Is/was there any change in the interpersonal relationships between you and your spouse and other family members after the event?
There is no change, but it will take time to cope.
13. How did the family members support you and your wife during the difficult phase?
They supported us, after hospital live at brothers' home for 4 days.
14. What were the reactions/ counselling and suggestions given by relatives/neighbours/ community after the episode?
Relative and neighbours said that why you go for treatment in S hospital, they don't treat well.
15. Do you think and/or discuss with your spouse/ family member(s) about the death and related events and blame someone/something or feel guilty for the same?
I blame myself I should not have taken the child to S hospital.
16. When did father and mother (if working) returned to work after the death of the child?
Started work after 4 days.
17. How your and your family's life has changed after the death of the child?
<substance abuse> No change.
Knowing cause of death in detail- Autopsy and MITS
18. What was the cause of death of your child and what is your perception about the value of knowing the cause?
Yes, it is important to know the cause of death it will help us to care for next child.
19. In your view, what more could have been done to know the exact cause of death and the reasons causing the disease?
I don't know because no doctor was present at the time of death.
20. Are you aware of any method for identifying the exact cause of death?
21. What are your views about the autopsy/ post-mortem for identifying the cause of death?
<about PM> Yes, I know, I have seen it, the head is open, chest is torn and the doctor checks what is he cause of death.
<religious aspect> This should not happen post-mortem is not allowed in religion

<disfigurement> yes

22. What are your views about collection of the tissue (like done for biopsy to diagnose diseases) and body fluid samples for identifying the cause of death?

<about MITS> Yes, this is right.

<religious aspect> Why does it make sense, no one gives guarantee because take it out from body. This is right.

23. How parents and family members can be approached for the tissue and fluid sampling?

<who should approach> Doctors

<whom> Father and mother

<method of explained> by oral explanation

24. In your view, what factors are important for acceptance of the tissue sampling by the parents and family members?

It's important to know the cause of death.

Decision making dynamics

25. In routine day to day operation who takes decision about the major activities (purchases, treatment, spending money) about the family?

Majority of time decision is taken by me only because in-laws are in village, sometime ask suggestion from brother on phone. (F)

26. During the hospital stay who discussed with the doctors/ nurses to make the decision(s) about treatment and other procedures.

I was in hospital, So I make decisions. (F)

27. After death of your child, who decided about the cremation and other post-death activities and rituals?

I was deciding about the cremation and post-death activities and rituals. (F)

Summarization

28. Now, we are completing the interaction/interview. Would you like to add anything?

What happens by diagnosis, just got information only, look, ma'am there was nothing when I brought the child to hospital he was playing but got very upset when drip was applied.

In-depth Interview parents of deceased neonate-10

**1.1**	**Primary respondent (relation with the child)**	Mother
**1.2**	**Age of the primary respondent**	Mother-18-20 years
**1.3**	**Other members present**	Nani (Grand mother of child)

The child was admitted in NICU. Mother of the child was staying at her parent's house after the delivery.

2.1 Can you please let us know about the illness and events that led to death of your child?

As the child birth took place in S hospital, so he was directly admitted to NICU.

Hospitalization period

3 Now we would request you to tell us something about the period of hospitalization for your child?

Nani: When baby was in the stomach, then she came here, started saying that no more time left to deliver, it will be here. I went to the hospital. The child did not breathe as soon as the delivery took place there. Mother- was born, was born well, Just, did not cry, neither cradle her. After getting admitted, I should have patted in reverse. Should have cried, delivery has happened at this hospital (S hospital). Where he took it, just went inside and admitted it, tested blood, and weighed two kilos and six hundred grams. - Water goes in the mouth of the child, the breath also becomes slightly up and down. Mother- If something gets stuck, it does not cry in the mouth, then cries after a while, they go straight and get admitted. Wrapped up. And he is saying that he got the phone on the phone and not a single missed call came on the phone.

I called my husband and said that the phone is not picking up. The doctors were lying. A missed call did not come in the phone that I had, the phone rang at 5 am.

It was up to 8 o'clock in the morning, and then admitted there. He said that your child has not cried. Was under developed, Was not small, was moderate, occurred throughout the ninth month.

Nany – when they kept in the ICU, where the children live, now they give milk, so tell them that you can feed the baby now. I tell you. The first time we went, we did not know that we would say go and feed the baby, (Mother - go on your own), then, she started saying that you feed the baby yourself, you will see yourself coming (Mother - there was no one to tell there) they could have told about all this, we don't know enough. We went for the first time, then said on the second-third day, after two-three days he said that your child has passed away.

<How long has your child been in the hospital>

Mother- Only one day. Delivered on the 21st, and then discharged on the 22nd, we had Eid on that day, Muharram was also on the same day.

<The doctor gave some reason>

Mother- No reason given, just sent. Granny- Did not tell anything, Madam, started saying take it away. Did not give any reason Just gave a paper and said take it away. And said to take the treatment paper, we said what the child will do when the child is no more.

4 Could you please describe your overall experience about the hospital care?

Mother- Blood test was done but did not tell anything about it.

I had given blood to bring a report. I went there, so gave the report to them, I do not know what I saw, what I did not see, we are not so educated, we are illiterate.

<Spent in S Hospital >

Mother - It has happened, it has happened a lot. From here my husband took four thousand rupees. I donate the blood. It cost to feed and feed him. Excess of expenditure was incurred, then after taking the child, his clothes came with everything which was kept in the hospital. I had to throw all the clothes that were kept at home. There was such a situation there in hospital, only the stomach was being pressed, looking up from the top of the body, by checking up, when a child is in the stomach of a human being, it is not pressed so tight. He was pushing hard, shaking, which was wrong. The delivery was normal. There is a problem when there is a first child.

5 Could you please describe your experience about communication by the various hospital staffs during hospital stay?
<when child was admitted what they used to communicate>
Mother- They did not say anything; they spoke just fine. Nani - First they said that she is breathing now, then it will be alright, then later on they started saying, there is no improvement. After that they started infusing glucose. Somebody said that he is having epilepsy. Don't know what was happening seizures were coming, then giving medicines, Sometimes injection,
Mother - they are lying or the truth, I do not know. The hospital was in a bad condition.
Mani - I could not understand anything. First started saying that he has started breathing, baby is in normal condition, then after a couple of hours he started speaking something else, then started saying that now the child has died, take it. They Signed on a paper and then gave the dead child.
<When the child is no more, who was he with>
Nani- There was no one there, the child was in ICU. We were not allowed to go to the doctor.
<Who Calls from ICU>
Nanny- Doctor, inside ICU, the doctor made the call that the child is no longer there. In Mother-ICU, number five was kept in the ward.
Death and post death period
6 Could you please tell us about the death and death declaration of your child?
<sympathy>
Yes, he started saying that now there is no child, he just said this and nothing else. Then he said that if your child was alive, he would be lazy, he would remain fascinated, he would continue to read such a lame, if he is such a living, what life would be like.
Mother - giving excuse like this, The doctor was speaking all this, the doctor who was recruiting all the children, he was speaking the same thing.
Nani – they were non trust worthy
Mother: It was my first baby.
Nani - When the child is breathing, you will live, now you speak like this. I said that now there is no use, the child is here, it is not even alive, so the children are then asleep, we were helpless,
Doctor said do all formality and relive the dead body.
<doctor behavior>
I felt bad at the doctor's behavior, very bad. If the doctor was good, my child would be playing in his lap today. Nanny-Lady's behavior was very bad. Mother- She was speaking as if she had never become a mother herself. As the doctor was behaving there it was very bad.
Grandmother- used to behave very badly.
Mother- She was also screaming during my delivery. My first delivery was At first, it was hurting so much. The incision was being made, the scissors were cutting the skin. I was saying that I have my first child, then said that you should do everything yourself, hold your feet yourself.
Nany - Some people do this.
Mother- I was holding her leg myself, at that time she has to bear pain and hold their feet and manage all. How difficult is everything at that time. And that was my first experience, I didn't even know about them all.
Nany - It is better to have delivery at home. The midwife gives help, but, at hospital, there is very bad situation.
Mother- Stitches are being put all over the on the body, it seems like, I became not pregnant again after that incidence. Who can one behave like her.
<nurse behavior> Nani: The nurse drives him out, does not allow him to enter.
Mother- The nurse used to treat people like dogs, tell me! If someone will admit a daughter, then relatives will come. So what should humans do Never let anyone live more than one. Someone will die alone inside. Their reputation was so bad.
Granny - I was not allowed to go inside; she was driven out. The guards used to drive everyone away. The nurse used to chase them away.
Mother- Now I have thought that there is no need to go to the hospital again, even if the person dies in the house. Who will go in such a situation? Nani- Madam, I used to hear from other people that there at hospital one get proper treatment, but after going there I saw very bad.
7 What was your and your family member reaction to the death of your child?

<Who narrated to you>Mother- My husband did not tell that the child died in the hospital. He said that he has injected. Still sleeping Will get up later, bring home like this. Then it was later revealed that the child is not there. Then I lost my life.

Nani- What would you say there? Bring home from there. Weakness had become so anyway, telling about it was a big setback, so I said to take it home. Then it will tell that the child has passed away.

Mother- Did not tell me that the child is not there. I was very weak.

The grandmother was weak and she was feeling dizzy. I brought it with me.

<What was the reaction when the doctor told no child>

Nani - The child was given in the hands of my husband.

<father response>Nani - Everyone was crying. The woman's cry was heard by everyone, not the man's. He was happy at first, then sad. Mother- When he heard that the child was born, he was happy, told his friends, told his housemates, then when he heard about this sad news, become client, not prefer to say anything. He would not cry in his mind, that even my first child was not left. Where the hospital considers good, it turned out to be so bad.

8 How was your experience about the procedure after death till handover of the body to you?

Mother- It took a long time to handover the child dead body. Nani- they were doing lot of paper work and getting signed, it was 7 o'clock in the morning.

Mother- The doctor called at 5 oclock in the morning.

Nani- In the morning, they sit down to drink tea. At the same time called.

Mother- around 8-8:30, they complete the process and handover the body

The child died at five o'clock. Dead body returned at 8 o'clock. When he left at 5 o'clock, he said that he is breathing a little.

Nany- Because of not being doctors, they were lying in giving bodies. I called my husband. When the child was dead, they were not handover the body. What will you guys do with it? He said that he will wrap you in cloth and give it to you.

9 Who all from your family and community/neighbourhood were present at the hospital and how they supported you during this period?

Nani: My husband and Mine with grandmother will be there.

10 What all rituals and procedures did you follow after death of your child?

Mother had brought her home (nani place) from the hospital.

Grandmother - had brought here only. After bath, was buried as he is buried in his Muslim.

<Azan read at the time >

Nani: No! he was there in the hospital. Who will offer blessings there Like I do at home, I give love to a child.

<How many days do you give azan>

Mother- Just like, after three days, the hair wall is cut, after the sixth class, then they offer azaan. Because when the pak gets cleaned, baby, when the pak is not cleared there, what will a human being give there? Had taken the child to Nahla at home.

11 How did you, your spouse (wife/husband) and immediate family members cope with the difficult phase?

<rituals after death>

Mother- Yes, I did not do anything for a month, my condition was bad anyway, under the tension of the child, in tension anyway I went mad with Roh-Roh, one month remained like this for two months. Then go to your senses, what to do now, when Allah kept it, now tell me what to do, now by crying, singing, leaving food, now it will not come to me. So I thought, now slowly, slowly, now slowly, I have held my senses, when the ninth month was when it was done, then it broke from the tenth eleventh month.

<coping mechanism> Mother- How to emerge, how you are coming out, my mind is that how I am like, because I will not be my first child, how will I get out of there, sometimes sitting in sadness, sometimes thinking, that Maybe I too would have children, now what should Allah tell me.

12 Is/was there any change in the interpersonal relationships between you and your spouse and other family members after the event?

Nani- Its man is right, he explains it. Mother- My husband is very right, he says that there is no problem, it is not there, it is not right, you are there or you will be enough. I was cheering me on, where did I go?

13 How did the family members support you and your wife during the difficult phase?
Mother- Mom, my father, my brother-in-law, father-in-law, since this was normal delivery, every one counsel me, never mind, don't worry, everything will be fine.
14 What were the reactions/ counselling and suggestions given by the relatives/neighbours/community after the episode?
Mother- What will everyone say, how is it that what has passed, what has happened, what to do now, tell everyone what had happened or the speaker thought, what the event was supposed to happen to me. It is done, now no one can bear me for a long time. <any reaction / suggestion> Nani- no one gives advice.
15 Do you think and/or discuss with your spouse/ family member(s) about the death and related events and blame someone/something or feel guilty for the same?
<do you blame someone for this event>
I consider the doctor guilty. Granny- Does anyone believe a guilty person, will blame their fate. Mother-man considers her luck to be the blame, and will not consider anyone guilty, what can anyone say now. If our luck is bad, what to do, what we have done wrong (S Hospital).
<whom do you blame from hospital>
Nani- He will know exactly who has a child in the hospital, now who will speak there, now there is a lot of child being born, now this is happening to everyone, someone says that by taking money. Mother- The doctor should tell the right thing, sometimes he is saying that he has got it right, now he is breathing, sometimes he said that epileptic tears are coming, what to do now. Granny- It is a big deal for a big man, a person who writes and knows, what the illiterate knows, just as they sit where they sit, sit down and tell them later, this is what happens. Well educated people understand the matter, why do they do such poor things?
Mother and Nani both crying during speaking
There (S Hospital) there is less of freedom, they are getting a job from the government, they are getting notes, then it is a job of self-interest, poor poor is dying, let him die, what does that mean to him? The Mother- Do not do this, you should treat everyone as equal, whether Muslim or Hindu, a woman is a woman.
16 When did father and mother (if working) returned to work after the death of the child?
Granny works in office, Private. Mother- You get something to pick something up, pass it, still working in the same company, to order someone or to speak to someone, they are calling,
<returned to work>
Nani- Ever since he was with his parents, he has been doing this work. Mother- The child has been working ever since he was finished. Did not sit at home on any day. She is discharged on Sunday, but she took leave that day. Because we reached there at night on Wednesday. Granny- son, now it is a matter of where the man will sit at home, will work even at home, now he will not sit with sadness broken. Mother- I started doing household chores after a month or two.
17 How your and your family's life has changed after the death of the child?
No Such change has seen
Knowing cause of death in detail- Autopsy and MITS
18 What was the cause of death of your child and what is your perception about the value of knowing the cause?
Madam, who will take such a tension, now who will go there and ask. <value of knowing the cause> Mother- I knew everything when there are so many presses in my stomach, there are so many fingers in the place of the body, I knew at the time that my child was no longer there. And how are you going to get out from there? You are not letting it out from there, when it was admitted, it was admitted, now the shadow is dead, the shade is normal, it is from the operation, the child has to go from here or not Left. What should I leave, no one is going to leave, my mother is not allowing me to come, how to get out of there, and to whom I speak, how do I tell my mother, I am not letting you come there, kept me inside. Is recruitment.
19 In your view, what more could have been done to know the exact cause of death and the reasons causing the disease?
Mother: How would I know this. No !
20 Are you aware of any method for identifying the exact cause of death?
Mother :don't know

21 What are your views about the autopsy/ post-mortem for identifying the cause of death?
<What is your opinion about post mortem>
Granny - Now buried, how will the post mortem. The post mortem is to be done by him, that has not happened.
<In which case post mortem is done>
Mother- In which case post mortem is done, we do not know.
Granny - We do not know all this.
<What happens in post mortem>
Mother- What happens in the post mortem, they do not even know.
22 What are your views about collection of the tissue (like done for biopsy to diagnose diseases) and body fluid samples for identifying the cause of death?
Mother- Now this is the point that there is no one to tell there, went for the first time, how would we know, there is so much crowd, no one is telling anything, there everyone is busy with himself.
<What would the doctors do if they told you>
Mother- Yes, I would have investigated him, so that we could know the cause of death
<religious aspect>
Granny- don't know, no we don't get it done, leave it to God,
The one who is doing it is God, the one who gives it and the one who takes it,
Now get the post mortem done, do a blood test, who has died from which disease, there are lot of disease, how long will the person keep checking, now where will anyone show it, who will see which doctor, which doctor will tell. Mother: Some doctors drive away.
Nani - Now what is the disease, what is not there, who will be so upset, what happens in the destiny, what to do now, the information is there where children are born in the hospital, there is no one willing to tell and no information from whom. Will take.
Mother- Now I do not understand that something must have gone wrong in the mouth of the child, then he would not have cried, at first, it gets stuck on the stomach, the breath is moving, this is how I feel They said that they have passed, in the first place they are saying that they are not crying, then they went and admitted that their beating was going on, then after two-three days they said that now it has passed. is. Until the child gives Roh too, now he does not know what he did with him, he knows something. <mother of the child asking her mother> Mother- you don't know, they will know who they have done with her. Nani - What to do, what not to do now
23 How parents and family members can be approached for the tissue and fluid sampling?
Mother- No such person is found there (S Hospital) who will explain this, there is no one there, and no person looks good there.
Nani- Madam, ask the people outside, who are lying outside, take information from them, they will tell everyone.
Mother - who lives outside the hospital. How the days are passing, their condition, how they are, they tell everything. How doctors are not listening, how to do it. Nani - do not let go in, how are one can standing with tea, and ignore the patient and relative. When ever ask something from them, they ask security guard to get out us.
ing cut, Madame is driving out, she is the watchman, security guards tell everyone how they do. They are poor, they are their children, they tell them everything. The one who passes over it tells.
24 In your view, what factors are important for acceptance of the tissue sampling by the parents and family members?
Mother: we opt this to know the death reason. But, nothing is possible this time.
Decision making dynamics
25 In routine day to day operation who takes decision about the major activities (purchases, treatment, spending money) about the family?
Mother: My husband and mother in law take a call for all things
26 During the hospital stay who discussed with the doctors/ nurses to make the decision(s) about treatment and other procedures.
Mother: My mother said that you can manage the delivery done at home, you would stay at home, the midwife would also help. I thought that my mother-in-law would blame on me if anything wrong had happened, so I thought that the hospital would be well supported, there is a lot of caretaker,

It also helps. I did not know that would be so bad with me. Yes, it was my decision to go to the hospital for delivery. Now I came to know that all this will happen, I do not have to go ahead from there.

27 After death of your child, who decided about the cremation and other post-death activities and rituals?

Ritual bath, in the last was done by my father and my husband, all the family of my brother was there (burial ground) to go to burial.

28 Now, we are completing the interaction/interview. Would you like to add anything?

Nani - Only when we have any information, we will tell you. What is not known, how will they tell? They only say that the child has died. They directly say that if this treatment is to be done, get it done or go back home. Do not let anyone go inside. Do anything. The nurse should help, but they do not do it at all. Whether the first child or the second one treats everyone badly. They should improve their speaking.

In-depth Interview parents of deceased neonate-11

**1.1**	**Primary respondent (relation with the child)**	Mother
**1.2**	**Age of the primary respondent**	Mother- 18-20 years
**1.3**	**Other members present during the interaction**	Mother-in-law

During the interview mother of the child and grandmother was present. The mother delivered twin babies at S hospital. After the delivery both mother and child were admitted in KMC (Kangaroo mother care) ward. One child is alive and healthy.

2.1 Can you please let us know about the illness and events that led to death of your child?

Mother-in-law: Both were twins. Mother- The problem was nothing. He was fine until morning. Mother-in-law, we came from the hospital in good condition. It was a healthy upto 10 days at home and I used to sleep with him (mother). Now she (mother) was feeding. I came to cook breakfast with the child. After keeping household garbage at outside, he had some difficulty breathing in belching, and then he (2nd child) started crying and then started feeding him. Now when he saw him sleeping, his (1st child) breath had stopped breathing. My son came to speak then immediately we took him to M Hospital because we had enough work time and did not take S Hospital. It was a child's thing we thought that what would happen would be seen. Then got the child admitted here. They were saying that there was difficulty in breathing. We will recruit it here. We will keep it admitted here (M hospital). It will take 25 days and 60 thousand rupees a day. So we said then you will guarantee for the health of the child, if you take 60 thousand rupees a day, then it is M Hospital, so I said who was preparing the paper for its discharge, it was good ladies. He told me that the child's condition is very bad. And if you take it then it is good otherwise this (M hospital) people will make you money and will do nothing. It was good that the one above prayed that he told the truth, then that thing sat in my mind, then the doctor said that it will have to be kept in nursery for 25 days the cost is 60 thousand a day. So I said Doctor Saab then you will guarantee me, then the child's my child is fine, to say that the guarantee is nothing, then the ladies told me first, who was going to write, yes, then I said when not taking the guarantee So, what should I administer, then I will go to S Hospital by taking it (1st child). Then called the S Hospital, the staff asked us to bring the child here. He had given us the number the KMC ward had left us by car, so he had said that no one can see you call us. We were in a hurry at that time, went there early, so he made a maximum hospital bill of 12 thousand within an hour and a half. Put the child on the machine for one-and-a-half hour, there was no movement in the child, pump the child, so the child used to move with it. Then after that, he called the S hospital and said, "Bring you here," the doctor at the nursery said, "You bring the child to the child." Then we told them (M hospital) that we will leave our child with S Hospital and then start saying, "First you ask the bed that the bed is not empty then we will give it to the child." We called again, so he (S hospital) said, we tell you in 10 minutes, then he called after 10 minutes there, then he (S hospital) said yes bring the bed empty. Then we told them (M hospital), give us our baby, we will take it, then started speaking to bring an ambulance. Then there is M Hospital here, there was a person to know, then he called him and reached with an ambulance. Then do not say that the child will go in our ambulance, the child will go to our machine, our doctor will accompany. Then he returned the ambulance, then he called his ambulance by phone, then he took him to the ambulance, and the doctor went along, the doctor kept doing the same pump.

Then when we went there, in S Hospital in an emergency ward, then we had a doctor. Then those who had come to leave the KMC had given the number to call the same number, called the same brother and said to them then they said to the doctor that they have taken the child. so doctor said, you do not come to remove the child from the machine, I come in the vehicle (ambulance), then I saw the child in the car, then I saw nothing in the child, no breath, then those doctors Some talk with each other, we talked among ourselves in English, we could not understand. Then those who have taken in that they go inside and admit, see, the child can be right, so I hope you know what it is said that it will be alright, then there he played 4 like that, kept it on him M Hospital's doctor came back to admit the child on the machine (M hospital doctor).

Hospitalization period

3 Now we would request you to tell us something about the period of hospitalization for your child?

Then put pipes in his nose, it was too late, four o'clock in the morning, 8.30 to 4. At 10 o'clock we had taken him to S Hospital, and reached there at 10.30. There was no movement in the child in such a long time, as he had brought the child it was the same since morning. Then we told the doctor to tell us the truth, do not deceive us. We too have children; I also have three children. Nothing is happening in the child since morning. Then he said that we tell you after 10-15 minutes. Say at 5-5.15 takes away the child. S Hospital went for 10.30 and discharged 5.15. It was said that take the child. But the doctors with the nursery had spoken in the morning with a view they said that there is no hope in the child. S Hospital's nurse said on seeing the vehicle saw the child in the ambulance and said nothing in the child. Then do not know what happened, the doctor talked to each other and then assigned him. Both children were born in S Hospital, meaning I had taken it for check-up, the day of check-up was in S Hospital, by the way, there is MM hospital. It was running in it, all the ladies write the same name when it is time for delivery. Then he (MM hospital) told us that you have two children, you can be seen in a big hospital sometime later, you came to the hospital a month ago, he wrote the name (S Hospital). Is then after a month we called, then we took it, then when it was taken with it, I saw it and said, admit it. There was only one day left for 8 months; 30 months would have been completed in 8 months. Then bid admit, it can be a child anytime. We admitted that to him then he said, wait for the blood, then the boy also gave a bottle of blood, then the child was born at 2.30 in the night, one was HOA at 2.35, the other was HOA at 2.40, both children were poor and weak. Then the doctor called me when he was born and said did you know that I had two children, I said yes, I knew that the hospital people of Malivya Nagar had said, this is the paper. So said, look, the children are weak, so they have to be kept in the nursery, so I said it is okay, the doctor will speak for the right, then they have admitted in the nursery. Then there was a child in the nursery, then after the nursery, gave the child in the KMC ward, first they used to go to the nursery to feed, then the child in the KMC ward would tie it to the chest in the KMC ward, then they were discharged, then came at home. The child was also fine for 9-10 days. At home, there was no visible.

4 Could you please describe your overall experience about the hospital care?

Mother-in-law: There was no fault of the hospita. He did not take any carelessness. He did very well, in every way the child was right, but his life was so much. So what will I blame someone, the child came right from there.

5 Could you please describe your experience about communication by the various hospital staffs during hospital stay?

<when you stayed in hospital from morning till evening, what doctor were telling you>

Mother-in-law (M hospital) said, the child is having trouble breathing, this hospital person said that he is having trouble breathing while weaning, like he fed (1st child) then He did not belch, thought that the child was asleep, the other one started crying, I came down to make breakfast. This (mother) stays up, so in so long she thought it was a sleep let's first give it yellow it also started crying. When it turned yellow when he saw him (1st child), he was dead, he died at home, when the child stopped breathing at the same time.

Death and post death period

6 Could you please tell us about the death and death declaration of your child?

<When doctor came to you at 5 o clock what did they told you>

Mother-in-law: Went out of the house from 8 in the morning, stayed in M Hospital for two-and-a-half hours, we reached here (S Hospital) at 10–10.30, they accepted, by five in the evening. No child will be cured, so you answer us, but we said, but then he said that we tell you in 10-15 minutes then he said.

<The child was put on oxygen>

That machine which is light, the machine was just on it. And he did not do anything, no medicine, no injection, nothing.

7 What was your and your family member reaction to the death of your child?

<reaction of father in hospital as mother was not present in the hospital>

Mother-in-law: Was very bad, in the hospital itself

(Mother crying during interview.)

8 How was your experience about the procedure after death till handover of the body to you?

Mother-in-law: It took a while, gave the discharge paper everything in hand. It did not take much time for all this work. After that he had brought home. They said that they pack the body in polythene we said no, they will be taken in clothes. Then we brought home

Mother-in-law was also crying during this question.

9 Who all from your family and community/neighbourhood were present at the hospital and how they supported you during this period?

Mother-in-law: I was in the hospital; his grandfather was also there. His father was also there, he had not taken it (mother), because he had a small child, he said (grandfather), do not take his mother and go, the younger child is afraid of infection. We were 5-6 people. He also had a mother.

10 What all rituals and procedures did you follow after death of your child?

Mother-in-law: They brought home from the hospital, many people came to the house, our family, and everyone came from the village. Then went to the crematorium.

<rituals>

Came home with the baby and bathed him

Mother- Mummy had agreed, the doctor said that until the baby is 2.30-3 kg, she does not have to take a bath. When he came from S Hospital, he was not even taken he took the cremation ghat directly. In it my husband and father-in-law went there.

<Something was worshiped in the house after that>

Mother: There was no worship, nothing happens in the case of children.

<procedure>

Mother-in-law: Just like this for 4-5 days, people came from her mother's house, my girl had all come, had a child, now she has another child so it is important to see everything.

Mother-in-law, I had a bhandara here. There is also kirtan in my house every month I thought that just normal delivery should be done whether it is boy or girl.

Navaratri was also going on, so I thought that Bhandara will do it, many people will get Prasad. Then when we came to the house, I went to Oh Pandit, that our children are fine, both, there is no Prasad Wagaira, even after the arrival of the hospital, I was 18 days later, I thought I will do the bhandara, when the name Karan Will do it, will set the time for the fifth Navratte in October, but then the child was finished on the 24th, then after that we went to the Pandit, so we said, now tell me what to do, so to speak. , Navratte is coming, Mother's gift was her own He said that his life was the same, the second child is right with the blessing of the one above, so please store it. So on 14 October, we have a further temple, there was a bhandara in the temple, distributed 700-800 man's offerings, then even in his name, 21 days had passed.

11 How did you, your spouse (wife/husband) and immediate family members cope with the difficult phase?

Mother: Now I will take good care of this child.

12 Is/was there any change in the interpersonal relationships between you and your spouse and other family members after the event?

Mother: My husband used to take care of me from the beginning, now he has started taking care of me and my child a little more.

13 How did the family members support you and your wife during the difficult phase?

Mother: Everyone was there, Ma, aunt, grandmother other and from here my aunt-in-law was mine, and all the others.

14 What were the reactions/ counselling and suggestions given by the relatives/neighbours/community after the episode?

15 Do you think and/or discuss with your spouse/ family member(s) about the death and related events and blame someone/something or feel guilty for the same?

Mother-in-law: No, we don't blame anyone, not anyone.

All I say is that God gave two toys for just this day.

16 When did father and mother (if working) returned to work after the death of the child?

Mother in Law: He started leaving after three to four days.

17 How your and your family's life has changed after the death of the child?

Mother in Law: No, nothing happened. Everything is the same.

Knowing cause of death in detail- Autopsy and MITS

18 What was the cause of death of your child and what is your perception about the value of knowing the cause?
Mother-in-law, we have to know the reason, yes, we wish. How did the breath suddenly disappear?
19 In your view, what more could have been done to know the exact cause of death and the reasons causing the disease?
Mother-in-law: You tell me you are a doctor; we don't know about it.
20 Are you aware of any method for identifying the exact cause of death?
21 What are your views about the autopsy/ post-mortem for identifying the cause of death?
Mother-in-law, understand from Post Mortem that if someone is a big man, ate some poison, then he should have it, this child was innocent. Don't want to be in the child, Bikul was such a child, what would be his post mortem, doctor, I did not even allow him to pack in polythene, I took my child like this. Now what would you have packed him, even if you have seen here, otherwise he would have packed it and given it like this. We brought our house by auto. The cremation ground is here and went there. Because the time was too much, it opens till 7 o'clock, then the rain also came. Then his grandfather went there (shamshan ghat) but then he told him that Panditji is an innocent child, now how will we keep in the house at night, it will be a small child, so we came here by 6.30 pm Got auto by 5.30, then came here, it took an hour here to 6.30, then he (panditji) said, bring it. Dug the hole for so long, then there were many people from here.
22 What are your views about collection of the tissue (like done for biopsy to diagnose diseases) and body fluid samples for identifying the cause of death?
<religious barrier>
Mother-in-law: would have agreed to this technique, which they did not say anything about.
<religious barrier>
Mother-in-law: There is no problem on behalf of religion.
23 How parents and family members can be approached for the tissue and fluid sampling?
Mother-in-law: Telling me or telling my grandfather, if there were any, I would definitely consult among us. In this matter, we would be pleased to say yes.
<in hospital who should approach>
Mother-in-law, as the doctor tells you it will happen, as if you do not know how someone has stopped breathing, you will know that if we do this. So we would say that it is okay, even for the future, we would have knowledge (knowledge) because of which death has happened, it was supposed to happen.
<how to explain about this technique>
Mother-in-law, even if they do not speak to us like this, do not see that you want to know how we will stop the child's breath, we will do this, he would have agreed to what he said. Had to break it even further
24 In your view, what factors are important for acceptance of the tissue sampling by the parents and family members?
Mother-in-law: I can't say anything about this. He came right from the hospital. The child had no problem. He got good care in the KMC ward too, all was good and there was no showing.
Decision making dynamics
25 In routine day to day operation who takes decision about the major activities (purchases, treatment, spending money) about the family?
Mother: The expenses are decided by my mother-in-law.
<decision about treatment> Who to take to the hospital, who has to be seen, all of them also do my father-in-law.
26 During the hospital stay who discussed with the doctors/ nurses to make the decision(s) about treatment and other procedures.
Mother-in-law: The one who becomes the ladies inside the hospital, where they said that to get the child's paper made for admission, we said, "Okay, let's get the admission papers made, I was also with her grandfather. There was a ladies' south -Indian was mean, you don't know what came in his mind that there is nothing in the child, it was understood that money will be made, we live here, our so is home, how many people stay in M Hospital, Even after selling everything without interruption, nothing happens to the patient. Yes, it was my and my husband's decision, he (S hospital) spoke to us, the nursery ward staff told the KMC ward that anytime the child is seen, you should bring it directly

here (S hospital). But when I saw it at home, it was nothing but breath means the child was nothing, at that time. At that time, I saw M Hospital in front of me, how could my child survive.

27 After death of your child, who decided about the cremation and other post-death activities and rituals?

Mother-in-law: The child was not bathed. We were all householders. There is a cremation ground here in the nearby village, the same thing happens to all elders, children and children. They burn the elders, bury the children. The priest is there, takes 500 rupees means then takes the child from the house in a new cloth with a cloth and buries it there.

28 Now, we are completing the interaction/interview. Would you like to add anything?

Mother: If the cause was known, then it would have been known what caused the death. Mother-in-law, did not mention this tissue sample to us, did not mean that the child is like this, it is okay that it was done, but you say that you should do it like this, if at that time we would have done the same. As soon as the injection is known by the class, it becomes known. He did not mention this to us, just said take away the child, nothing else.

In-depth Interview parents of deceased neonate-12

**1.1**	**Primary respondent (relation with the child)**	Mother
**1.2**	**Age of the primary respondent**	Mother's age 30
**1.3**	**Other members present**	None

Context-

Events that led to death of the child

2. Can you please let us know about the illness and events that led to death of your child?

This was my first child. He came here to get treatment. Previously used to live in Calcutta. Due to this my husband lost his job. The child was shown in S Hospital. It was not seen by the eye. The man did not recognize and did not even walk. He was just five years old. He who has just died, died two days after birth. Stayed in the hospital for two days after its birth, then brought it home. Then the health of the child started deteriorating and went to the hospital with him again. He took her early at 4 in the morning. One day, he was hospitalized and died the next day in the nursery. At around 4 in the morning my child died.

Hospitalization period

3. Now we would request you to tell us something about the period of hospitalization for your child?

Yes, I and his father were in the hospital. One child was delivered at home and one was at S Hospital. Everyone said that this child should be treated well. Before that my child who had died was also in S Hospital. This time also the heart check up and all other tests were done earlier. My child was not treated properly. This time it was sent after a little investigation. We have come home. After feeding here, foam started coming from the mouth. The heartbeat was accelerating.

4. Could you please describe your overall experience about the hospital care?

<about treatment>

Went to the hospital. I had tried my best. Had an x-ray done, a blood test was done. A day and a night after getting admitted, a senior doctor came. Seeing that, quickly get it done by x ray. After checking the empty blood, the doctor was waiting for the doctor to see if the doctor would come. Later put it in the lead and injected it into the vein everywhere. We did not tell anything in time, if we were quick, we would have taken the child to another hospital.

<about investigation>

The investigation was correct but nothing was known from it. There was no blood in the body.

<about cost>

Did not take money in S Hospital, did not take it there.

5. Could you please describe your experience about communication by the various hospital staffs during hospital stay?

The child had to pipe to breathe. The doctor had come and seen well in the first child. This time neither saw the investigation report, nor x ray, just discharged. The behavior of the doctor and nurse was not good. The treatment was also not done properly. People used to say that your child's condition is not looking good but the doctors did not tell anything.

Death and post death period

6. Could you please tell us about the death and death declaration of your child?

<who declared>

I did not know that who is a doctor and a who nurse. Somebody used to come and do some investigation and would not even tell. A senior doctor came and told the rest of the people that they should do this, do not do this. The child was not breathing. Still do not know what you were looking for. Did not tell us anything. My husband used to tell me that I do not understand what is happening. Those people were lying. Was saying that keep giving medicine.

<cause of death explained> Did not tell anything. Someone said that he has a heart disease. Nothing was found.

<sympathy/empathy> not given

7. What was your and your family member reaction to the death of your child?

No, the child is dead, take it. The father made his discharge paper and said, take it and get it signed. All the papers and medicines were with the doctor. His hands and feet were all swollen. Showed the doctor in Govindpuri. At that time my condition was very bad.

8. How was your experience about the procedure after death till handover of the body to you?

<Time taken> Signed in immediately. It was 12 o'clock here, 12 o'clock in the night.

<cost or payment> No

9. Who all from your family and community/neighbourhood were present at the hospital and how they supported you during this period?

Yes, he had called the neighbour, his name is Kishan. He had gone to his village, called him. Husband had called, called, helped, everyone brought the child. Over here everyone had left the child. Papa came with 3 people. Everyone helped.

Post death procedure and coping mechanism

10. What all rituals and procedures did you follow after death of your child?

The child was dead at 7:30, so we had gone at 12. First came home, then took it away. It was raining heavily. The child was kept outside. Was buried on the banks of river Yamuna, near Kalindi Kunj. About 10 people came. My relatives, who live in Ludhiana, had called them and called them. There was no worship on that day. Everyone had taken the same there.

11. How did you, your spouse (wife/husband) and immediate family members cope with the difficult phase?

12. Is/was there any change in the interpersonal relationships between you and your spouse and other family members after the event?

Nothing change !

13. How did the family members support you and your wife during the difficult phase?

No ! we had called one person and went with him

14. What were the reactions/ counselling and suggestions given by relatives/neighbours/ community after the episode?

Nadad and other relatives came and they were giving some suggestions and they understood those people stayed for 2-3 days. Their boy brought them. They say that what was to happen has happened, do not worry about it. Do not worry about that

15. Do you think and/or discuss with your spouse/ family member(s) about the death and related events and blame someone/something or feel guilty for the same?

The guilty do not understand anyone. As if it had done x-ray and blood tests, it would have been known as to why the child had died because of this. Only said this is heart disease.

16. When did father and mother (if working) returned to work after the death of the child?

Now ! By the way, he started working after 3-4 days, started work after 3 days.

17. How your and your family's life has changed after the death of the child?

Yes! Now more attention is paid to these children. We take him to the doctor only if there is some problem. Think that if he had taken the child in the night, it would have been right, he was late at home. Husbands do not have a bad habit such as drinking alcohol or cigarettes.

Knowing cause of death in detail- Autopsy and MITS

18. What was the cause of death of your child and what is your perception about the value of knowing the cause?

Should know we were told by the doctor that he had a beating disease, so he has died. If someone asks us how the child died, they cannot even tell.

19. In your view, what more could have been done to know the exact cause of death and the reasons causing the disease?

There is no information about it told me that he has a heart disease. Then it is over.

20. Are you aware of any method for identifying the exact cause of death?

<about post mortem> No ! don't know about that

21. What are your views about the autopsy/ post-mortem for identifying the cause of death?

22. What are your views about collection of the tissue (like done for biopsy to diagnose diseases) and body fluid samples for identifying the cause of death?

<about MITS> I am telling so many people. It would have been good to know the reason. Right now I have incomplete information about how my child died.

23. How parents and family members can be approached for the tissue and fluid sampling?

It is right to tell the doctor, but they do not even speak what has happened? How has it happened?
24. In your view, what factors are important for acceptance of the tissue sampling by the parents and family members?
Decision making dynamics
25. In routine day to day operation who takes decision about the major activities (purchases, treatment, spending money) about the family?
26. During the hospital stay who discussed with the doctors/ nurses to make the decision(s) about treatment and other procedures.
Its father! Used to do all the work
27. After death of your child, who decided about the cremation and other post-death activities and rituals?
Do not bathe him. We all kept weeping and went away like this. By the way let's bathe. Old people in the house tell all this, bathe it and wear it. Kafan wrapped him and took him, his uncle had brought him. His uncle had taken him under wraps. Not bathed is considered not washed. Mane was left in a weeping state the bathing is in the wash it was my pleasure that the same 2-4 men took it away.
Summarization
28. Now, we are completing the interaction/interview. Would you like to add anything?

List of participants: In-depth Interviews of parents who had Stillbirth

S. No.	ID	Age	Religion
1	Participant 1	Father- 25-30 years<br>Mother-25-30 Years	Hindu
2	Participant 2	Mother-20-25 years<br>Father-25-30 years	Muslim
3	Participant 3	Mother- 25-30Years<br>Father- 30-35 years	Hindu
4	Participant 4	Mother-30-35 years<br>Father-30-35 years	Hindu
5	Participant 5	Mother-25-30 years<br>Father-30-35 years	Hindu
6	Participant 6	Mother- 25-30 years<br>Father- 30-35 years	Muslim
7	Participant 7	Mother-30-35 years<br>Father-30-35 years	Hindu
8	Participant 8	Father- 35-40 years<br>Mother- 30-35 years	Punjabi Hindu
9	Participant 9	Mother-20-25 years<br>Father- 25-30 years	Hindu
10	Participant 10	Mother-20-25 years<br>Father-25-30 years	Muslim
11	Participant 11	Father- 30-35 years<br>Mother- 20-25 years	Muslim
12	Participant 12	Father- 25-30 years<br>Mother- 20-25 years	Muslim
13	Participant 13	Father-35-40 years<br>Mother-30-35 years	Muslim
14	Participant 14	Father-25-30 years<br>Mother-25-30 years	Christian
15	Participant 15	Mother- 20-25 years<br>Father- 25-30 years	Hindu
16	Participant 16	Mother- 35-40 years<br>Father- 40-45 years	Muslim
17	Participant 17	Mother- 30-35 years<br>Father- 35-40 years	Muslim
18	Participant 18	Mother- 20-25 years<br>Father- 25-30 years	Hindu
19	Participant 19	Mother- 20-25 years<br>Father- 25-30 years	Hindu
20	Participant 20	Mother- 18-20 years<br>Father- 20-25 years	Hindu
21	Participant 21	Mother- 25-30 years<br>Father- 30-35 years	Hindu
22	Participant 22	Mother- 25-30 years<br>Father- 30-35 years	Hindu

In-depth Interviews of parents who had Stillbirth-1

**1.1**	**Primary respondent (relation with the child)**	Father, mother
**1.2**	**Age of the primary respondent**	Father- 25-30 years<br>Mother-25-30 Years
**1.3**	**Other members present**	Grandmother

1. **Care seeking practices**

1.1 **Where did you usually go or whom do you consult for routine antenatal check-up duringthe last pregnancy?**

1.2 **Where did you usually go or whom did you consult for any problem during the last pregnancy?**

Father – In hospital, In molar band, we have a S clinic, treatment was going on there. It was already known (about pregnancy). Everything was done there only. Everything happened there only. It was not 7 months, 7 months were not completed that we in between, there is a polyclinic in badarpur. One also has to go there to get your name registered it is from MCD we get birth certificate from MCD only.

1.3 **Where do usually the pregnant women from your locality/community go for check-up and problems during pregnancy?**

Father- Yes, it is necessary to go there (Badarpur) or S Hospital. There is a Government dispensary there is a dispensary in Badarpur, It is a polyclinic address is in Badarpur market itself means, outside Main Road.

1.4 **Please specify the reasons for the preference for the facility and/or healthcare provider.**

(Father) - It is not about community, we have to go there, go two places have to get the name registered in government because we have to get the birth certificate, either in dispensary or S Hospital.

2. **Events that led to death of the stillbirth**

Please let us know the course of pregnancy and events around the delivery/ termination. (Please document as the event were told by the respondent)

Father- We went in the seventh month (S Hospital). Nothing like that had happened, we had gone, meaning someone had said, her aunt said it, that here in Badarpur itself, there is a N doctor, Lady doctor, to show to her, just for regular check up. For getting ultrasound done. To get the medicine, to take medicine means that is it all well or not. He looked there then he got the ultrasound done, then there he saw that the child was spoiled (badarpur clinic) he was sent to S Hospital again.

Mother- It was a matter of 8 months only.

Father- He said that my first child also got spoiled like that it happened two years ago, child was delivered through operation. It got spoiled after 2 hours following operation. Treatment was going on in S Hospital.

2.1 **Can you please let us know about the problems, illness and events immediately before the delivery?**

Mother- Illness this time I had no problem, last time it happened, at the time of first delivery. There was fever, it was dengue. This time there was no problem.

2.2 **Where did you go or whom did you consult for any problem during the last pregnancy?**

3. **Hospitalization period**

3.1 **Now we would request you to tell us something about the period of hospitalization for the pregnancy, delivery or termination? (Probe: hospital, ward, duration of stay, course of illness)**

3.2 **Could you please describe you overall experience about the hospital care?(Probe: investigations, treatment given, satisfaction level, quality of care, cost of care)**

Father- No, from outside we used to get the ultrasound done. I had to bear cost of that only. (Father) (Acceptance of ultrasound in S Hospital) Yes, would have agreed.

Mother - What happened in the first delivery was from S Hospital itself. When they had said to get it done from outside. He had got it done from outside.

Father- He is the one who says that it will take a month to get a number for ultrasound, get it done from outside.

3.3 Could you please describe your experience about communication by the various hospital staffs during hospital stay? (Probe: who all communicated, who was the primary communicator, nurses, doctors, other staffs, frequency of communication)

Mother- Well it was all fine. But a little of more crowd get we screwed and carelessness happens there. Last time it happened, that they had said for my operation. They had already informed me that your baby is spoiled. So you have to go for operation, Pan had started at night. It was also said at that time that the it will be operated the she said that it will be normal, then they waited till morning, it happened at 8 o'clock in the morning, at that time it was Saturday or Sunday, they were a little careless.

Father- It got spoiled due to doctor's negligence.

(Grandmother) - It was Wednesday, Wednesday. I went there thrice to call.

(Father) - She was a doctor, she would come, she would come, and the rest is a nurse. The nurse cannot do anything without the doctor's consent.

(Grandmother) - No, Big doctor is not there. There is so much negligence there.

Mother- Well, the doctor was okay, but some are careless. There are so many crowds. When they knew that, the tests were done, that operation has to be done then they have got the child out. Possibily he could have have survived. Afterwards they told that dirty water was swallowed.

Mother- Yes, It was good that only one was there. I do not even know the name she was a very good doctor, she was saying that we do not want to operate on you, we want it to be normal. They behaved very well this time. He tried his best, and so I was discharged in the middle, then he was admitted. Doctor used to come only once in the middle. The nurse came this time, once I was having so much pain, said that take it, now is the correct time, to bring me back down, this time was fine, last time they were very careless.

5 Stillbirth

We would like to know about the events around delivery and after that in the hospital.

5.1 Could you please tell us about the declaration of the event and outcome (Stillbirth)?(Probe: who declared the outcome, the body language and expression, preceding events before that)

Mother- I felt like the child is moving around a little, I was feeling, there is a nursing home, there is a Bhavani nursing home, near the State Bank, he told that your child is spoilt, I mean I did not feel even a little bit that I could have this problem, because there was no problem this time, this time there was no problem in eating and drinking. Still I don’t know what happened they themselves had referred. They had seen the ultrasound report twice.

No, they did not say anything to me, seeing the report from here, he had said, that your child is no more alive.

Father- This was told in Badarpur.

5.2 What was the cause of stillbirth mentioned to you and what is your perception about the value of knowing the cause? (Probe: knowledge about the cause of stillbirth, willingness to know the detailed cause, potential factors that led to the illness/death, potential impact on the other family members or next pregnancy)

Father- no (asked whether the cause of stillbirth mentioned to you).

(Mother) He had said the same thing earlier I had kept the discharge paper with me, the operation one. Due to which reason your first child had also got finished, that is to know, why this did happened again (S Hospital doctor)

Even before, they were trying to know what was the problem that the first child also got spoiled, they had asked for the discharge paper, last time I had fever, dengue had become, in the last time pregnancy.

(asked respondent if you asked the cause of death)

Mother - I did not even know this thing of the rest it is being said that there is shortage of water in water bag. Why does it happen, I used to drink plenty of water it is not that I did not had water. I drank 10-10 Liters of water, drank eight liters of water a day, this time there was

water in the child’s water bag, it is not like that, it was not there, there was water in the water bag.

Because when I was admitted, I was not given any treatment, the infection had already spread, nobody gave anything they said that till the time there is water in water bag there is no fear of infection. They said me to eat more food daily take juice daily. You should not get weak. Rest there will be no problem. The doctor also said that I take this gaurantee. Nothing will happen to you.

(Mother) They were sent in the 8th month, they were then discharged.

(After 10 days), then there was so much pain, after 2-3 days they had said if you have any problem, fever, headache, any problem or if water bag bursts etc., you have to come immediately Then when pain started, (after 10 days) I went to S Hospital.

5.3 Do you blame someone/something for the stillbirth?(Probe: who or what is it, what did you do in this context)

(Mother laughing) No. The complaint would have been lodged when I had not got anything This time I truly ate and drank, still I do not know, I am shocked I don’t know what happened, and what not happened. No (asked if blame anyone). Because it was not in my destiny otherwise I would have got it.

5.4 How was your experience about the procedure after death till handover of the body to you? (Probe: processes, documentation and papers given, respect for religious norms, time taken, cost/payment, assistance from the staffs)

(Husband) They did not bring home, there was no one at home all ladies used to stay in the hospital only.

(asked who received the body) Father- gave it to his mother only, to his grandmother. Because at that time, gave it to nurse. Because it was delivered at night only it was not given at night because where we would have taken it. It was given at 5 in the morning we were sleeping in surrey below. And there were ladies inside.

Father- (document) there was some paper. Like envelope. They had given in that only. It was a big envelope. (Showing stillbirth report).

(Father) - No, there was no problem there. All was told there. (Assistance of staff), they did not ask for anything (cost), (staff), they were helping, yes we did have full information.

(Mother) - (asked about HCP behavior during body handover) No, it was fine with me this time they had said that the child has been spoiled. If you feel anything, feel any problem or nervousness or if there is discomfort or whatever problem you have, then tell it immediately, if nobody listens then shout immediately. We are near only. Any problem after delivery, because your life is in danger.

5.5 Who all from your family and community/neighbourhood supported you during this period? (Probe: who informed them, who all came, what type support given)

(Mother), the rest were seated below. I was called to give the child,

(Father) No, we were about 7-10 people.

All of us could not go. I was there with my brother-in-law was there. We they also had boys, our sisters were both. She was a mother, it was also her mother. Which means whatever he came to know, he came in the same way its aunt is a girl, she works at the hospital. She was also on fire, had called me at night when we, in delivery time. Everyone was on fire.

(Mother) Do not let anyone go in the labor room, my mother was in ward. (Biological mother) my both mother were there.

6 Post-event procedures

6.1 What all rituals and procedures did you follow after stillbirth? (Probe: body preparation, who prepared, burial/ cremation, other rituals and timings)

(Father) - They are buried they get buried only when the body is found. We were given it at 5 in the morning. So we were engaged in bringing 1-2 hours, had done it by 7-8 pm. At that time, I, Papa, our Jijaji, Tau's boys and 1-2 servants were also there, all the women were here. His mother was in the hospital, my mother, no, there is no need (presence of females during burial).

No pandit is blessed we mean no need of a pundit in this. When the need of a Pandit reads, I am telling you the donation of the body, it is for him when someone's name becomes Karan or

not, then it happens, that Pandit, the name of charity. Now whatever has not happened, it has not been born. The molars are buried in close.

I did not tell you until the name are due or nothing happens (document).

(Father) gives (burial permission without any document). Nothing was found from there (document from burial ground).

6.2 How did you, your Husband/wife and immediate family members cope with the difficult phase?

(Father) will have to be raised, Madam, who has fallen asleep, if he was not in luck, he was not found, more than luck is not available.

6.3 How did the family members support you and your husband/wife during the difficult phase? (Probe: burial/cremation rituals, community norms/rituals, coping with the phase, financial)

(Long silence then after a while)

(Father) - He was the same in the family member, our sister had come to Jiaji, aunt had also come, and they meant brother's in-laws.

(Asked after returning from hospital, who supported among relatives) (Father) - No one came after him, after that his brother came to Vaghera, came to the hospital, he used to come home from there.

6.4 What were the reactions/ counselling and suggestions given by the relatives/neighbours/community after the episode? (Probe: what to do after death, following the rituals, knowing the cause of death, any other)

(Father) - In that service the address was read at the same time, when we were taken, I called up at home, that I am going to the hospital, S Hospital, I had called the mother to come to Badarpur, ambulance from there. We went again.

(Father) - At that time, toh was not there at home, no one was a father, yes after that you come.

(Mother) - No one speaks in front everyone gives comfort, no matter what the person says at the back, the rest was fine.

7 Knowing the cause of stillbirth in detail- autopsy and MITS

Many pregnancies in our community end with stillbirth due to various causes and in several instances the exact cause is not identified. We need to know the causes of stillbirth in detail. We want your views on the options of knowing the causes of stillbirth in detail.

7.1 In your view, what more could have been done to know the exact cause of stillbirth and the reasons causing it? (Probe: investigation, autopsy, any other)

(Asked why you were called by S Hospital after 1.5 months of stillbirth)

(Mother) - To forget the same thing I went to it, went to S Hospital, had called me, after 1.5 months I had asked.

(Father) - Delivery was done on 15 August, and we were called after 1.5 months.

(Asked why you were called by S Hospital after 1.5 months of stillbirth)

(Father) - Family planning was called, no department was the same, but family planning are different.

(Mother) - He had just said that the child does not conceive the body is weak, gave 3 months of medicine, up to 4 months, meaning after 6 months of pregnancy. It is closed for 2-3 days now, it is kept, and I forget it. Calcium, etc

(Mother) He did not only tell me what was the problem, what was the problem with the child, for which reason it was bad for you, he also did not tell me what happened inside and what did not happen. Beta saying this thing, you have someone, tell me the reason, because all the reports, all the tests, everything comes normal, mine, what had happened and what had not happened. I do not have any problem I do not know what happened.

7.2 Are you aware of any method for identifying the exact cause stillbirth? (Probe: autopsy, investigations, etc.)

In several instances, doctors advise for autopsy/ post-mortem to find out the causes of stillbirth so that appropriate action can be taken to prevent recurrence of such event.

7.3 What are your views about the autopsy/ post-mortem for identifying the cause of stillbirth? (Probe: necessity, religious aspect, time needed, disfigurement, etc.)

In some instances, autopsy/ post-mortem are not possible due to various reasons. To find out the exact cause of stillbirth and underlying disease, some tissue biopsy (collection of very small sample using needles) and fluids (blood, urine, etc.) from the body, as done for investigation. But these samples are to be collected as soon as possible.

7.4 What are your views about collection of the tissue and body fluid samples for identifying the cause of stillbirth? (Probe: necessity, religious aspect, time needed, disfigurement, etc.)

(Asked respondent view on MITS for 2nd stillbirth)
(Mother) No, they knew what happened and what didn't.
(Asked respondent view on MITS for 2nd stillbirth)
(Mother) - Yes, it would have been agreed.
(Father) - You know what the problem was.
(Father) - will get it done, will read what is the problem of the meaning of this thing, at that time our condition was such that the child should become normal.
(Father) Look at the blood, well, you have to read the blood, but Tisus cannot say well, you will have to ask the family.
(Asked respondent if collection of tissues/body fluids is against religious beliefs)
(Father) No, it will not happen, it is difficult anyway.

7.5 How parents and family members can be approached for the tissue sampling?(Probe: who, when and how should approach and whom)

Only you will know this (father). Whatever is right, it will be considered by everyone, father.
(Mother) - (Wife Indicating Towards Husband).

7.6 In your view, what factors are important for acceptance of the tissue sampling by the parents and family members?(Probe: desire to know the cause, trust, confidence on the health care provider)

8 Summarization

8.1 In your view, what can be done to improve identification of cause of stillbirth and efforts to prevent?

(Father) - She comes in the culture itself, according to us, she is a different thing, she is a coming, means that there are some people who are not called ghosts, something she is in our family, something, because of that This thing has happened, we had shown it from outside, yes it means whatever comes in it.

In-depth Interviews of parents who had Stillbirth- 2

**1.1**	**Primary respondent (relation with the child)**	Mother
**1.2**	**Age of the primary respondent**	20-25 years
**1.3**	**Other members present**	Maternal Grandmother

1. **Care seeking practices**

1.1 Where did you usually go or whom do you consult for routine antenatal check-up duringthe last pregnancy?

We used to go nowhere used to go. Got pregnant in last February 13 in 2014. (Did you consult anyone) No (while shaking her head, sideways) we have stones in Gallbladder. I have pain because of that. (Any problem during pregnancy) – No (so you did not consult anyone) No (since when in Delhi) we came here in the sixth month from Bihar. No investigations were done there. (Any check up, by anyone) I got vaccinated there but ultrasound was not done. (Routine check up there) Possibily it could be done No one went there we had asked lot of people that who is going. Please tell us the hospital so they said that we don't need to go to hospital. We knew that the ultrasound has to be done in second or third month. We also said that let's go but he said that we will go later Will go in the fifth month (to whom you said) I told my He mother-in-law it happens in the fifth month It happens in the month, we will get it done in the fifth month then they left me here and went away they said that they cannot keep me with them

1.2 Where did you usually go or whom did you consult for any problem during the last pregnancy?

They did not take us we were in Bihar, so they did not take us we came here 2 months ago we went here for the ultrasound to the S hospital, so they said that come after getting ultrasound done. When I brought to the ultrasound, deficiency was reported in child. Whe I consulted the doctor advised me to abort the child. We got our first ultrasound done in 7th Month. (Since when in Delhi) We came here in sixth month. From there (Bihar) before that we did not have any investigations done there. (Any check up, by anyone) I had got my vaccination done there but there was no one for ultrasound. That one only that has to be done in a month.

The one that has to be done in third and fourth month the child's (mother) two vaccines were done there. I don't know which vaccine was given The child was vaccinated with two vaccines there. (Did they come at home or you had to go somewhere) they came home and got it done. (Any report) (Which vaccination) Vaccination of newborns when the child was in abdomen. (Any doctor, there) Yes very far away no one took us there along us could not even go alone (How far) have to go by train do people there (in Bihar) go for any consultation People won't go very soon When it seems necessary then only If there is no problem then they will not go. We said it right Aaganwadis with no work would come they also gave calcium tablets I went during the time of my menstrual cycle. (Did anyone refer you to S Hospital) No

1.3 Where do usually the pregnant women from your locality/community go for check-up and problems during pregnancy?

We go to hospital far away in Gopalgunj they did not take us we were in village so they did not take us.

1.4 Please specify the reasons for the preference for the facility and/or healthcare provider.

(according to you which HCP is better any preference) We had said him to go to a doctor but when no one took us so we didn't go (anganwadi) those people never used to check they used to call us and give medicines and leave doctor's are always good but not aaganwadis

2. Events that led to death of the stillbirth

2.1 Can you please let us know about the problems, illness and events immediately before the delivery?

(Mother) We called me called here here they were not getting treatment done check up was not being done we called them she was there for 4-5 months we called her here in sixth month.

No treatement was being given we said her to come so we said her to come again she came with her father-in-law So, we let her stay with us Father-in-law said that we will come next week now a month

has passed and I kept wondering that her father-in-law did not came to Gaya So I said no your father-in-law was saying that I will get treatment. So I went to get ultrasound done I found in that ultrasound that body of whole child has become bad It was very bad I asked any chance whole child's body was poor in that ultrasound That was useless We said some hope had it been 19 – 20 so we would have kept it is not worth keeping it was visible in the ultrasound itself that one near lal kuan So we thought that what the use of keeping it is.

2.2 **Where did you go or whom did you consult for any problem during the last pregnancy?**

There was no problem that doctor had said that child will be over as soon as it is born there is no point then what is the use of keeping it. So it is better you abort it.

3. Hospitalization period

3.1 Now we would request you to tell us something about the period of hospitalization for the pregnancy, delivery or termination? (Probe: hospital, ward, duration of stay, course of illness)

Now I do not know where she had taken it one lady had taken it Jasola that lady had taken who used to administer polio drops they had told us to get an ultrasound done from S Hospital they had told us to get an ultrasound done from S Hospital when I got the ultrasound done then they told that child does not have head (How many times you visited the hospital) We went only once (After the ultrasound) No

3.2 Could you please describe you overall experience about the hospital care?(Probe: investigations, treatment given, satisfaction level, quality of care, cost of care)

No their behavior was good (Mother) They behaved right Our daughter-in-law also has two children she used to only take care of it she used to take her and bring her Getting vaccinated.

When we went, they said that you first Did not get the ultrasound done I said no then they said that your child has a problem When asked so she said that the back of your child’s head is not developed It is not worth to keep it like that you can consult your doctor for the time being they went to S hospital they said that you should abort the kid It is not good to keep the child your health can go down you can also die there is some danger from the pregnancy.

3.3. Could you please describe your experience about communication by the various hospital staffs during hospital stay? (Probe: who all communicated, who was the primary communicator, nurses, doctors, other staffs, frequency of communication)

No, the doctors are all very good used to talk well We went there We got admitted on Saturday or Sunday Then the doctor came on Monday She advised to give him the medicine of pain, if the pain arises then it is okay otherwise a Give it a break for the week when we were about to leave, the pain rose then we got relieved after delivery. Discharged only after

(nurse behavior) No, she was very good She used to talk nicely No her behavior was very nice In giving medicine with them too he was like a friend with everyone

(Behaviour of other people in hospital) Where ultrasound was done they also told me no where ultrasound He had also told (anything about cause was told to you) no, they said that you Should have got it checked once that your child is bad showing the doctor to go and get advice from him he said that drop it.

5 Stillbirth

5.1 Could you please tell us about the declaration of the event and outcome (Stillbirth)?(Probe: who declared the outcome, the body language and expression, preceding events before that)

(about stillbirth) where the ultrasound was done, only then they told me no, they did not tell everything before they said that, you get it checked, the doctor you are consulting, once you get advice from a senior doctor, they asked us to abort so I aborted. Then I drop it.

5.2 What was the cause of stillbirth mentioned to you and what is your perception about the value of knowing the cause? (Probe: knowledge about the cause of stillbirth, willingness to know the detailed cause, potential factors that led to the illness/death, potential impact on the other family members or next pregnancy)

No (willing to know) what can happen God’s wish from whom should we try to know It has happened room that should we ask (mother speaking) Done what will doctor do (daughter) The doctor said to us directly they give medicine to pregnant lady I think that was not give to you that is actually right that was not given to you that is actually right we did not eat any medicine may be they would be giving medicine of calcium or of something we did not have it.

(Mother) What can happen due to that If we stay hungry then there will be no head we have had 6-6 kids we did not eat any medicine It has become a trend now Polio and all we did gave a needle to the child fever did not come now what should we do now we did not know what will happen to them despite of eating the medicine this has happened.

No, what has happened, it happened even after fully drinking water. We have not seen a doctor. Here only this happened here only it happened here only we have not seen face of doctor.

5.3 Do you blame someone/something for the stillbirth?(Probe: who or what is it, what did you do in this context)

Nothing because we knew that that child will not survive what would we think about it. We already had said that child will die after being born when he was born he was alive Nurse had come his heart was pumping after that he just did one kick he hit the nurse and that was over he was dead in his hands was finished in his hand (did you see) hmmm he was not even 6 months old not even 6 months old.

5.4 How was your experience about the procedure after death till handover of the body to you? (Probe: processes, documentation and papers given, respect for religious norms, time taken, cost/payment, assistance from the staffs)

When he was born he was alive Nurse had come She instantly checked and found that his heart was throbbing after that he hit the nurse There was one kick and he was dead (did you see) hmmm was 6 months old not 6 months old. After that he was taken away after that they did not show it again to me they did not give it to us rather they sent it through brothers we did not see it again. He had brought the child and then pressed it into the soil.

(How they handed over) No, in clothes then they took the clothes from here. They bought the towel of Rs100. My elder daughter brought it for us we wrapped it took it away (where) Where it is being dug in soil In Cementry.

(What do you do) Do nothing, just bury it. Nothing likes that not so (do you read anything) have Read kalma valma, then we buried it. We put mud after reading Kalma.

(In how much time) In half an hour (time for burial), here we showed it to these people, Mummy and all. After that we read namaz, read kalma and then went away with the body. Two kept the namaz, read the kalma and then have gone with it.

(did you sign any paper) no.

(They give you any paper) No he said the child is useless, he is dead bury him as soon as possible. This kid has become bad. It was a girl.

(When they gave you body of child) gave it in the morning, around 9.30 am. Around 10 p.m, the child was born at about 9 o'clock at night

(cost) No, it must have happened there where they had buried, where you bury that person takes they don't give space without taking money

(how much) around my boy would know. Women do not go I don't know woman don't go there

(Process of handover) What to do in it we cannot keep at home the children took what they did.

5.5 Who all from your family and community/neighbourhood supported you during this period? (Probe: who informed them, who all came, what type support given)

No one was with me my mother was there they are the ones, who supported, nobody else,

6 Post-event procedures

6.1 What all rituals and procedures did you follow after stillbirth? (Probe: body preparation, who prepared, burial/ cremation, other rituals and timings)

(rituals) Yes, yes, we have to do the Quran- khani now.

We did it about the child he should live with peace for the peace of child It will be 40 days, we have to do it after 40 days only. There is some trouble going on I have not done it for a while now, so that the child gets a good place. I need to feed the baby Food and all (dua) It is not known what is read and what is not we do not know now only that person reads the janaza We do not allow ladies to go near janaza no interference is allowed We just bathed, washed. Wrap in we have bathed it wrap it in clean cloth and take it after that

(presence of female members) these people were there, we were in Hospital.

6.2 How did you, your Husband/wife and immediate family members cope with the difficult phase?

Yes, it hurts, who doesn't get hurt. What all we had thought we thought that a child will be born If there is no new born then what to do.
Look at the baby of an animal, he also feels sorry, and this was a human child.
If it is now not there then what can you do

6.3 How did the family members support you and your husband/wife during the difficult phase? (Probe: burial/cremation rituals, community norms/rituals, coping with the phase, financial)

(did anyone come from your house): No, no one has come no one has called yet Niether they called we have not sent her yet now she will stay here only
(Family support) No one was there with me my mother was there, she is the one who supported, nobody else, neighbours were saying that it did not go well Everyone is happy what to do now. To share the sorrow I have my sister I share everything with her and other one is she
<any one from neighbourhood> no

5.1 What were the reactions/ counselling and suggestions given by the relatives/neighbours/community after the episode? (Probe: what to do after death, following the rituals, knowing the cause of death, any other)

Know the reason for delivery

7 Knowing the cause of stillbirth in detail- autopsy and MITS

7.1 In your view, what more could have been done to know the exact cause of stillbirth and the reasons causing it? (Probe: investigation, autopsy, any other)

What to do, what to check, now it will not be investigated. Where is the child Have you not seen the child It is not worth living

7.2 Are you aware of any method for identifying the exact cause stillbirth? (Probe: autopsy, investigations, etc.)

What to do, what to check, now how it will be investigated. Where is the child Have you seen the child whether it will live
(daughter speaks) we have different things in villages.

7.3 What are your views about the autopsy/ post-mortem for identifying the cause of stillbirth? (Probe: necessity, religious aspect, time needed, disfigurement, etc.)

What will we do when we have already given it to them when they have not done then what will we do what will we do in that hospital people got scared seeing it that what kind of child is this it looked like gost only he had big eyes, like a monkey It was nothing whole body was not able to form.
Its face was not even good he had big big eyes, it seemed like lemon .
(which month of pregnancy) his eyes were hick 6th month it was 7th month the face was so weird where should we take the child they did not do so what to do

7.4 What are your views about collection of the tissue and body fluid samples for identifying the cause of stillbirth? (Probe: necessity, religious aspect, time needed, disfigurement, etc.)

No, they did not speak himself, doctor people no (if asked for) we will never agree. No we will not allow you to do that till now this had not happened whether a boy or a girl

7.5 How parents and family members can be approached for the tissue sampling?(Probe: who, when and how should approach and whom)

No one will give

7.6 In your view, what factors are important for acceptance of the tissue sampling by the parents and family members?(Probe: desire to know the cause, trust, confidence on the health care provider)

No, no one will get it done. We do not destroy people, The body goes all clean the body is scented and is sent it does not go like this He was not a child The whole body is scented and sent We have photo in phone please show it to them then what will they do they will not make cuts these
(mother speaks) tissue biopsy No we people don't get it done See how much is worthless (while showing baby photo), the whole body is made, it is a devil, This is not a child. The delivery was over 5 minutes immediately.

8 Summarization

8.1 In your view, what can be done to improve identification of cause of stillbirth and efforts to prevent?

Government do not hear us quickly, the girl came on her own where she went, her name did not come she came on her own in scooter she got down here after the kid is born nobody will listen to you I asked for leave, so he refused to give holiday I thought it is a matter of one night and then we will leave then again the same thing Admitted to I thought it would be a matter of one night to go away tomorrow again the same not taking my name only people who came after me are being relieved I got angry I asked them to pick up the stuff and head towards home what should I do.
I was not having clothes to wear my clothes were getting dirty (mother speaks) There was one more lady she had said me the same thing that let us see till when will our name be called for One week my name was not called now just imagine the girl without bath for 2 months.
Is the government going to listen if my child dies Poor people go there What to do We don't have so much of money we went to Apollo doctor they had asked for 1.5 lakh for the girl's procedure then I realized and was taken a back.
Are we being heard inside there is only name of hospital ask your daughter (daughter speaks). I did not stay in so much pain other ladies were having more pain, they were shouting (mother speaks) They used to hit me (daughter speaks) They used to shout not hit me they used to take us into PV room for check up they used to check, when they feel that it has not happened so they will leave.
So the woman shouts in so much pain Nurse also gets irritated on why they are shouting when we re saying that it has not happened mouth is not pen why they send us back.
(Mother speaks) Everything is from outside Needle syrup. Everything is written and given from where we will arrange 1.5 lakh and the we are not having a child further had it been good then we would have invested on whom should we spend there is no use
(Mother speaks) Everything is from outside, needle medicine. They write it down and we have to get it. From where we will arrange 1.5 lakh rupees if something happens, even one rupee is not available From government what kind of government is this it seems to be government for name sake? And top over it we have spent 50,000 rupees in coming and going.

In-depth Interviews of parents who had Stillbirth-3

**1.1**	**Primary respondent (relation with the child)**	Mother, Father
**1.2**	**Age of the primary respondent**	Mother- 25-30Years<br>Father- 30-35 years
**1.3**	**Other members present**	Grandfather

Care seeking practices

2.1 Where did you usually go or whom do you consult for routine antenatal check-up during the last pregnancy?

Father- MM Malviya Hospital, since start of every month.
(Visit for check up).
Mother- She used to get blood tests done, and sugar test was done only once.
Ultrasound is done only once.
Father- Where has it happened, not even once
Mother- Once written, but we had an ultrasound done outside, 6 months old
(Done ultrasound)

2.2 Where did you usually go or whom did you consult for any problem during the last pregnancy?

Father- Yes (Madan Mohan Malviya Nagar Hospital)

2.3 Where do usually the pregnant women from your locality/community go for check-up and problems during pregnancy?

(Asked if pregnant women from your locality usually go to MM hospital)
We go to M Hospital, it is here only, and I also went to the same. But you can show here once here, once only My child got spoiled because of people, they are careless, doctor and all, they keep sitting with their mobiles, and patient has to sit in vain.

2.4 Please specify the reasons for the preference for the facility and/or healthcare provider.

Father- No, someone said that MM Hospital is fine, the hospital is right, and that there doctor is right, that is why he went, but all government hospital, government hospital are of no use.

3. Events that led to death of the stillbirth Please let us know the course of pregnancy and events around the delivery/ termination.

(Please document as the event were told by the respondent)
Mother- Madam, I will speak one thing Madam, When I was sitting there I had very severe pain in abdomen, but madam there was talking about karva chauth.
Father-Patient is getting faint but they don't worry, we are about to file a case, we will file a case for sure, we are sure this has got bad from injection. Those are, but we will do the case. 100% it is spoiled by injection even to the doctor I showed it to even doctor (MM hospital).
Mother- Turning my page again and again, repeatedly, turning again and again, I was having severe pain in abdomen and that madam was talking about her karvchauth leave. She was saying this thing. I was talking in myself (MM hospital).
Father- He is likewise careless, even in S Hospital. During the time of delivery when her child got spoilt they referred to madan mohan from there. I went to MM Hospital on 8th October there. On the 8th of October, I was referred from there, I went to MM Hospital On October 8, when the baby stopped moving in abdomen then I went there then I went to see him and did ultrasound. After ultrasound they told that child is moving perfectly the child has just overturned, that much he has told. He didn't tell that it is mentioned in report.
Mother- Doctor did not tell us. It is mentioned in report.
Father- When I saw the report later, it was found that the child has turned around.
Mother- We has not studied English so much so that we see that my child turns upside down. See us in it. Didn't tell us anything later.
(asked respondent when they reached S Hospital).

Father- The same day at 8 o'clock in the morning had gone to madan mohan in morning. They referred last at 8 o clock. (S Hospital), saying this cannot be treated here. Mother can also be at risk. She is not well. Mother can also be at risk.You can take her there. They have referred us from there, so after going there 12 hours. Till then it did not even touch anything, for 12 hours, after going in room number 12 I got to know that there is a head of S Hospital so he told that if the child has been spoilt in the abdomen then there is going to be no infection. I take this guarantee. There is going to be no infection for 7 days you can stay with peace. No problem you will get treated well. On his saying I stopped there. Then also 24 hours passed. It has no treatment, she is having pain No one is there to listen When her water bag had burst then doctor came otherwise no one was concerned. And one child was born infront of her in bathroom he was also referred from Madan mohan malviya. So baby, so careless. Main thing is that people are careless there they don't allow gents to enter inside, had males entered there doctor won't be able to survive. People are so careless that I was irritated seeing all that.
Grandfather- When paient went there on 7th on seeing the condition why did they sent her home. After giving her Injection. They could have let the child out through operation.
Father- 9 months were already over, the child could be let out.
Father in Law- they had given time of 29th for delivery. She was given injection and tablet and sent back home.
Mother- Rather I was repeatedly saying to Madam, that I am going to deliver, going to deliver.
Father-Wrong date was given. Date of 21 was given. Rather the child was born on 10 -11.
Father- I showed this to the doctor, lot of doctor. He told it has happened because of buscopan, it is medicine for muscle. It is medicine for muscle. Buscopan which, this is a medicine for muscle, to inflate and its dosage is hard, you search and see this buscopan dosage. The injection will show that, I have seen at night, and have shown it to 2-3 people 100% the child is spoiled by this, and the child died instantly there was blood from the nose, when he was born then there was blood from the nose through the mouth. And blood was fresh.

3.1 Can you please let us know about the problems, illness and events immediately before the delivery?

Grandfather- nothing happened to him, he used to eat well, drank, nothing happened.

3.2 Where did you go or whom did you consult for any problem during the last pregnancy?

4. Hospitalization period

4.1 Now we would request you to tell us something about the period of hospitalisation for the pregnancy, delivery or termination? (Probe: hospital, ward, duration of stay, course of illness)

4.2 Could you please describe you overall experience about the hospital care? (Probe: investigations, treatment given, satisfaction level, quality of care, cost of care)

Father- So tablet of brufen gave us tablet, pain, for pain. When the pain did not subside after taking tablet then they gave this injection of buscopan. And she told that pain can become low due to this you can go. If the pain did not get lowered down then you can stay at night and come back in morning. Time, he gave it to them, and what did he say, you go, it helps pan You will go, if the pan is not working then stay home overnight and in the morning I want to try you In the morning with an ultrasound again, it speaks the child broke down.
(asked respondent when they reached S Hospital).
Father- The same day at 8 o'clock in the morning, I had gone to Madan Mohan in the morning, he was referred at 8 o'clock (S Hospital), that it will not be treated here, the mother will also be at risk, the health is not good, the mother is also at risk you take her there. She was referred from there. After going there she was not touched for 12 hours, I inquired with them in room number 12. We went to enquire there, there is head of S Hospital. He told that if the baby has been spoiled in abdomen then there is not going to be infection for 7 days. It's guaranteed there is no infection for 7 days, you stay relaxed, its treatment will be done properly, at the behest of that, I stopped there, then Even after 24 hours, no treatment, she is having lot of pain, doctor is not listening, when the water bag burst, then he was taken into labour room.

In Then the doctor came, otherwise nobody had any sense, and a child was born in bathroom in front of her. He was also referred from Madan mohan. What if her child falls in bathroom tub they do not allow the gents to enter, gents are not allowed inside. Had gents been entered inside then no doctor would have been spared.
Grandfather - When on Dt. 8th she went to hospital then why was she sent back after being given an injection they could have taken the child out following operation. Father- Oh, the 9th month was already complete its child could have come out.
Grandfather- 29th was the date given for delivery. Why was she sent back after giving tablet and after giving injection
Mother- When I was repeatedly speaking to Madam, mine is going to happen, is going to happen.
Father- They gave us the wrong date. They had given 21st as delivery date. But the child was born on 10 or 11th.
Father- I showed this to the doctor, I consulted so many doctors. 100% it happened due to buscopan. This has made the muscle tight. This is meant to make the muscle tight. You can search it. It has very hard dosage. You can search and see there is buscopan injection. I have seen this has made the muscle hard. I have searched at night. I have shown it to 2-3 people. And the child got expired recently. When he was born there was blood from nose, it was borne, then blood from nose to mouth and the blood was also fresh.
(Asked respondant about Cost)
Nothing was spent on father-expenses. Nothing was spent in Mam.
4.3 Could you please describe your experience about communication by the various hospital staffs during hospital stay? (Probe: who all communicated, who was the primary communicator, nurses, doctors, other staffs, frequency of communication)
Mother- He did not allow meeting, he would not have met anyone there. Lived alone Even if the mother lived, she would not have allowed the mother to enter. My She was not allowed to live together, as soon as the doctors came to see, she came to see everyone, she Everybody is sitting 3–3 on one seat, patients sitting like this, there are sputtering with pain, mother When people, now when doctors enter, they exclude everyone. Don't let them sit together Were, 3 men are sitting on one seat, then the mother will be said, the whole room is filled in the same has happened, she said she will sit. Then she used to sit outside. With me at that time nobody was there. The time I was hurting more.
Father- Even when it was told that the delivery was done, it was happy was asking for water, but no one gave water. Is there a nurse or a doctor Or anyone, nobody gave water.
Mother- My mother went there at the gate, my mother-in-law who has gone there at the gate How is my daughter-in-law to ask, to show once, she is speaking I hear been there, then I shouted mummy, give me a glass of water, but I No one listened to the voice, voice, everything means busy in our own work there Delivery is being done from here to here, from here to there, all are doing their work. No one is listening.

5 Stillbirths
5.1 Could you please tell us about thedeclaration of the event and outcome (Stillbirth)? (Probe: who declared the outcome, the body language and expression, preceding events before that?)
Father- Hey, tell them, tell the patient to the doctor, the doctor only. After seeing the report of ultrasound, it was told that if any patient goes, the patient Such cases should be told that the patient should tell those who go with the patient Should this not be the case, if someone has a heart problem, he will die, So told in the last, first told it.
Mother- told us, so tell us they do not allow respect they said to wait out, we told them Jake, tell us that.
Mother- Talked in this way, your baby is dead your baby is in your stomach bad words have been spoken like this. Likewise, all the time, all who see the doctor Were, the way of talking, everyone talks in anger. Talked in this way suddenly she speaks, the child in your stomach has died.
Father- The most careless person is in Gaini ward, here and there too.
Grandfather- Whoever comes for free treatment, will speak to the patient.

Mother - If you are in pain, she sends us out again and again and sits there. Yes, after a while, we will call you ourselves, but see 10 minutes - 15 minutes. Gone, did not say anything yet, then would have sent us out angrily if I went inside is.
Father- mother not came (nurse after repeated calling by respondant).
Mother- Doctor is sitting there, is not looking at here, and is there Go outside and wait or else: she will throw the emerald tear; throw the report torn used to speak like this

5.2 What was the cause of stillbirth mentioned to you and what is your perception about the value of knowing the cause? (Probe: knowledge about the cause of stillbirth, willingness to know the detailed cause, potential factors that led to the illness/death, potential impact on the other family members or next pregnancy)

Mother- very much, never mined that, nothing has been revealed yet why did my child die? I am in shock as to how this happened. For what, one or two days Suddenly, I had no such problem, nothing happened at home, one It was a pain in the day, my child got so bad in a day, nothing like this happened.
Mother- I am not daring at all, now I will think like this also Madam. Mine is both were afraid of going to hospital (potential impact on next pregnancy).

5.3 Do you blame someone/something for the stillbirth? (Probe: who or what is it, what did you do in this context)

Mother- Yes, the doctor definitely blames Madam, this is his fault, when my, the way you are thinking, the way I have been treated. Who madam Madam goes to tell them that I have more pain in my stomach, so how are you Talking to, he is scared after listening to that. And not even going to the hospital now, maybe he won't even go to the hospital. Even such days do not come, very scared I got it from the hospital.
Mother- I was in this pain, growing in this pain, anything drug treatment, anything
Not done. After that, go to the same hospital (normal delivery).
Father- There was no injection, it happened on its own.

5.4 How was your experience about the procedure after death till handover of the body to you? (Probe: processes, documentation and papers given, respect for religious norms, time taken, cost/payment, assistance from the staffs)

Mother - I was shown, and I was shown at that time. What time I am So she herself had become unconscious, and then we kept the child with our hands, Move your hands like this, if the child suddenly does a miracle, the child wakes up, Tried so much, they were not leaving their hands and Madam people What should they see: the people, those people: even a doctor himself came, that child again I went there and then, I don't know anything after that.
Mother- (how handed over the body) - the child is just the child. See a blood from the nose, the blood was going a little bit there, nothing after that, the child was complete such a good child was not visible. Didn't seem to be sleeping Looked like a child. At no time did it seem that the day had happened, nothing like that, he was a perfectly made child.
Mother- No, I did not give them that way, they are: they leave here and keep them, right? See as much
Father- He was a medicine cartoon Baby was shown in the cartoon (showing) there was a cartoon of this type. We went to pick up the grandfather-child, put it in cartoons, after that we went Clothes were taken. I took a white cloth. After that, wrap it in cloth.
(Asked respondant for documentation)
Grandfather - Yes, signed Death Certificate.
Father- had given it at the same time Discharge paper to it is (showing), that paper It is written in the Death Certificate. I still have Already
(Asked respondant for time taken to handover early/late)
Father- No, it was delivered at about 12 o'clock at night.
Mother - Delivery was at 4 o'clock
Father was delivered at 4 o'clock in the evening and I received at around 10 in the morning, 10 Let in.
Mother - The child is done at 4 o'clock at night, not giving that time and gave so much in the morning they.

(Asked for cost after body handover)
Father- No, only one was charged Rs. 175 for medicine, just for tablet.

5.5 Who all from your family and community/neighbourhood supported you during this period? (Probe: who informed them, who all came, what type support given)

Father- all were family members, I was, I was a mother, I was a father, I had a brother, I was a child. The same No one outside (relatives).

Father- He already knew or not in the report, but he had a mother In Uppper, the only Ladies Allowed was Gents Not Allowed. Ladies with him, Mom used to live with him. So when I was a child, my mother came and said that she was a boy, is over. But the bus is not allowing me to meet, just showed the mother once, Uppar Since, take it, after that, not even let the mother meet her, its condition too How is it, no, Mummy, it too, was able to meet her at 2 pm. 4 o'clock Delivery was on the night. Mom got it at 2 pm next day.

Mother-support was received from my family, my mother lived at that time, Like being like time, there was another woman there, watching, everybody like this Sehli used to see my body at the same time, when the water burst completely, mother and both of us were taken to bed, after that they did not even have enough everyone was sitting outside.

6 Post-event procedures

6.1 What all rituals and procedures did you follow after stillbirth? (Probe: body preparation, who prepared burial / cremation, other rituals and timings)

Grandfather - Yes.
Father is buried, carried across the Yamuna. I had a father and a serving.
Grandfather - brought direct from there.
Father- went direct from the same, did not bring home. Dead body what is the use.
Grandfather - There was also a pandit where he was taken on board.
Father- He cremated, of Hindu society.
Father- Yamuna Par, Kalindi Kunj.
Grandfather - I also have a receipt. There, he deducted a receipt of 1500 rupees, he, the rule of the people, which will be, the receipt of 1500 rupees was cut and 400 - 500 rupees more Took, these flowers, the Ganges water, and the sandal wood, all this, 4-500, 2000 rupees total had asked. Yes, gave 1500 receipt.
Father- I don't know (taboo for women to go to cremation ground) Sometimes she goes.
Grandfather - The woman does not go, we burn the grown up, so the woman does not goes. Yes, gents go.

6.2 How did you, your Husband/wife and immediate family members cope with the difficult phase?

Father- What can I tell now
Grandfather - this is the first time in our family.
Father-puzzle has happened only once.
Grandfather- Like my girl also has two she also has 2 children both have operations after taking the child out, I have 2 daughters-in-law, this is normal.

6.3 How did the family members support you and your husband/wife during the difficult phase? (Probe: burial/cremation rituals, community norms/rituals, coping with the phase, financial)

Grandfather - No, it is like that, isn't it, my mother is in my singing, not my father Is mine, I asked them what to do, it has happened, it has to be somewhere in the Ganges, The Ganges is in the river, where it is, go and bury it, find it here, one Man said go here, here is very good for Hindus, of rebirth So, we auto reserved the same, just gave the baby, left immediately, I have 20-25 It took min to poach there. I have no one to support here, here I am alone I am a father, these people are all children, none of the relatives are mine.
Father- yes, (father supported)

6.4 What were the reactions/ counselling and suggestions given by the relatives/ neighbours/community after the episode? (Probe: what to do after death, following the rituals, knowing the cause of death, any other)

Grandfather - no, no, no suggestions yet, stay here for rent People, who would suggest, everyone has their own meaning, here someone there is no point Knew, but the serpent will know, like my 4-5 days the shop

was closed here. My customer who is a foodie, this is what happened this is the regular shop has been closed for 5 days. We have told those people, tell the owner as to whom I live in.

7 Knowing the cause of stillbirth in detail- autopsy and MITS

Many pregnancies in our community end with stillbirth due to various causes and in several instances the exact cause is not identified. We need to know the causes of stillbirth in detail. We want your views on the options of knowing the causes of stillbirth in detail.

7.1 In your view, what more could have been done to know the exact cause of stillbirth and the reasons causing it? (Probe: investigation, autopsy, any other)

Grandfather - this is not it, we can not take up this matter now, we He is a poor man, and cannot even fight a case with the government. Flame Quality the man said, now he does not have time to work, he gains Even we will not. We cannot fight the government's case. We will as our cows used to live, all the cows used to do something together and move there. But this will not do here. We do this here, right now, in the hospital in the crowd of 500 gets used. But this does not happen.

7.2 Are you aware of any method for identifying the exact cause stillbirth?(Probe: autopsy, investigations, etc.)

(Silence) no response by the respondants

In several instances, doctors advise for autopsy/ post-mortem to find out the causes of stillbirth so that appropriate action can be taken to prevent recurrence of such event.

7.3 What are your views about the autopsy/ post-mortem for identifying the cause of stillbirth? (Probe: necessity, religious aspect, time needed, disfigurement, etc.)

Father- No the post mortem does not get the younger child done. Not forbidden (religious aspect), but what is the benefit of having a post mortem of a small child. I know the reason 100 percent, this is also a doctor, ask, you have told me after seeing this. Here As many as the total student doctors of all mosques come, as many as Showed that 100% is spoiled by the child, by injection, bascopan, Reason knows, if Reason was not known, he would have done something, one day baby With the baby on the right day, the baby will go bad? In some instances, autopsy/ post-mortem are not possible due to various reasons. To find out the exact cause of stillbirth and underlying disease, some tissue biopsy (collection of very small sample using needles) and fluids (blood, urine, etc.) from the body, as done for investigation. But these samples are to be collected as soon as possible.

7.4 What are your views about collection of the tissue and body fluid samples for identifying the cause of stillbirth? (Probe: necessity, religious aspect, time needed, disfigurement, etc.)

Grandfather- the doctor will not tell you here. Not to tell, puzzles show when times she used to go and see it month after month, in Malviya Nagar, it we used to say that we will not go there. It speaks such a bad word, the government is there, we people thought that come on, someone said that in Malaviya it is very good treatment. Free there is treatment and it is also good, come on, brother is fine, hospital is very big thought, the work is useless.

Father- I would have given at that time would have known the reason. At that time, we would have given it. Yes, it would have happened at that time (agree for MITS).

7.5 How parents and family members can be approached for the tissue sampling? (Probe: who, when and how should approach and whom)

Father- Family members would have got them done, Papa.

Mother- Dad.

7.6 In your view, what factors are important for acceptance of the tissue sampling by the parents and family members? (Probe: desire to know the cause, trust, confidence on the health care provider)

8 Summarization

8.1 In your view, what can be done to improve identification of cause of stillbirth and efforts to prevent?

Mother- what would we suggest right now this happened to me in the hospital and Do not be with anyone this is what happens when people go to the bathroom, dying of pain Somebody, all that should be improved, the doctor should increase the care more because after the time, the children come, after that,

she comes to see. It keeps flickering like this, no one sees it on time, it is the doctor who has little should pay attention and.

In-depth Interviews of parents who had Stillbirth-04

**1.1**	**Primary respondent (relation with the child)**	Father, Mother
**1.2**	**Age of the primary respondent**	Mother-30-35 years<br>Father-30-35 years
**1.3**	**Other members present**	None

1. **Events that led to the stillbirth**
   - **1.1 Can you please let us know about the problems, illness and events immediately before delivery?**

     In the beginning, she used to work in S Hospital itself… So initially she was checked for 2–4 months there. She was on leave for 6 months. She was in security services you want to see there are gaurds at S Hospital.
   - **1.2 Where did you go or whom did you consult for any problem during the last pregnancy?**

     I was on leave for 6 months. They said, get your treatment from here if you apply with your documents, then you will get your salary.
   - **1.3 Where do usually the pregnant women from your locality/community go for check-up and problems during pregnancy?**

     Now as here is an E dispensary here people whose cards have been made the man says that now my ESI deducting money so why don't we just show up there is E hospital, Madam It is a very big hospital It has a good facility It is so Now they show in lot of private facility Now it is a matter of scope We see the budget and work accordingly nothing you will get benefits from One rupee to 50 lakhs, we get a cut montly we get a cut worth Rupees 2000.
   - **1.4 Please specify the reasons for the preference for the facility and/or healthcare provider**

     This is how my ESI card was made. Was on leave for 6 months, say, get treatment from you If you apply documents on it, then you will get your salary ESI people will give that's why getting my treatment done from here.
2. **Events that led to death of the stillbirth**
   - **2.1 Can you please let us know about the problems, illness and events immediately before the delivery?**

So now when my health went down in 9th month, she was admitted in hospital for pain in abdomen.We got nervous. Not much information is available. It was for first time information is not so much, so we went to the hospital it was the first chance. We don't have so much information. There is not much information so they admitted us <in S Hospital> not here in ESI Recruitment was done there. After 3 to 4 days of recruitment, all the checks were done fresh. They told everything is all right the child is a little weak.

But you have time there are 20-25 days. It will come. It will be all right, do the medicine They discharged us He took leave and came back home Then they shifted back here for 2–4 days she wa fine, there were less movements in abdomen She told me next day so we said, "Let us show it, it will be all right" Her health got worse at Sunday night We were on duty We had night duty She spent the night somehow in pain.

Then when we came in the morning on Monday, then again She had got some relief She was not feeling well in evening there was a little time too she was not able to understand that whether child is dead inside or there is some problem inside. So it was around same time, only 2–3 days were left so they were taken there When they reached there it was 10-10:30 they said that It is not possible to do ultrasound here. So they showed in emergency. Then they sent us to the labor room.

<in S Hospital?> In ESI So they wrote that you go in labor room here mature ladies go upstairs only so when they got it checked, they must have checked her with machine they told that there is no heart beat in child It is possible that child is no more alive So you get the ultrasound done after that we will start the treatment.

Then we went to Govindpuri, there is a hospital in Govindpuri, so he said brother we will be able to in the morning, it is not possible to do it now then we went to Sarita Vihar if it is done then it is right they refused there also Then there his health had become more worse There was no one with him So

they took her away so there is one chance only come to S Hospital There is 24 hours of service so we took her to S Hospital.
He also showed there. There they wrote for an ultrasound there there was so much crowd We could not get any place She was screaming in pain there was a guard We told them that we are from staff only Get my ultrasound done We showed I card to them then they let us go inside ultrasound was done then he was admitted inside no hearing there are too many patient what a doctor can do no one was hearing and she was irritated.
It is taken inside the room at 4 o'clock then at 11 o'clock in the morning her child was born So they started saying that the child has expired When you see the report of ultrasound.
He was taken in on Monday night I am not getting heart beat of the baby Was not able to get heart beat of child So it is possible that child is dead So from there only parents were She started crying We said don't worry, first let us get the ultrasound done. If went to two places, when it did no happen there, then we went to S Hospital
<delivery time and process> So at 11:15 in morning We
Had shown it to the doctor wrote ultrasound after showing it to doctor after ultrasound he had written an ultrasound. It must have been 11-12, that the ultrasound reports arrived they saw and said that the child has expired.
<illness> Madam, how can we say cold cough and fever all this goes on it is common it did not have any disease Lot of check ups were done we had got it done Nothing was detected Here also nothing came out so how can we say that she was suffering from something.

2.2 Where did you go or whom did you consult for any problem during the last pregnancy?

I have my own ESI card, so my treatment will be done there Now as there is an ISI dispensary now like here we have ESI dispensary So why we cannot show there ESI is cutting my money E hospital is there madam There is a very big hospital There are lot of facilities there.
It was like there was an ultrasound written in there for the fifth month so we had got done that ultrasound but we could not understand that thing that what is this ultrasound in that it was 1400 - 1800 rupees. Of in S Hospital everyone used to say to get it done from outside when ultrasound was written so it was doen from outside so baby was normal in it so, everything about the child was depicted there in ultrasound.
Whatever was there it was coloured one that one was written so they said that we have shown all documents in ESI We have got the ultrasound done in 5th month we have shown it to madam that look here are the apers She said that it is the plain one we wanted the one where one can come to know that there is no problem in the kid you will be responsible for it and get it signed If everything is right then you get it done what can I do if not done.

3. Hospitalization period

3.1 Now we would request you to tell us something about the period of hospitalization for the pregnancy, delivery or termination? (Probe: hospital, ward, duration of stay, course of illness)

<in ESI> In the labor room two three days 3 days (Shaving Discovery Paper) This is the discharge paper See what the date is <28th august was your admission> It was for the 9th month we knew this We knew it Yes This will be of August (Showing S hospital's discharge paper) This is of S Hospital <ward> 9

3.2 Could you please describe you overall experience about the hospital care?(Probe: investigations, treatment given, satisfaction level, quality of care, cost of care)

But the child was alive due to the negligence of the people the child expired a little problem was that the child had fainted again the child was jumping meaning It was going on like a ball How will it end It ends, mother gets upset Yes in negligence.
(cost in S Hospital) Nothing was the cost madam only it was spent on medicines. No injection were given <was ultrasound free> It was given free only.
what to say there is negligent due to a lack of attention in crowd there are so many Patients that on a bed we have 3-3 patients So what do you expect, what will be the condition of patient They do not allow anyone to go inside They never used to allow anyone to go it does not matter if one dies.
They are telling us how to go from inside when the name is called, then you have to go and she screaming inside that call my family at least they should see once. No one asks for water There are

patients who are agonizing in pain over water, they do not even ask for water <Any problem inside the hospital> She was speaking There was no hearing inside.
I was crying inside, I had to forcefully enter inside and fall Then she saw me that I have severe problem <how frequently she was checked> She never used to see just say that have patience Do not get disturbed Doctors were speaking we were not inside Now madam we were not inside what can we say. The doctor was saying that the nurse and all there are employees, they were looking after her people there were looking after her.

3.3 Could you please describe your experience about communication by the various hospital staffs during hospital stay?

5 Stillbirth

The doctor said after seeing the report. No the doctor said that the child has expired.

5.1 Could you please tell us about the declaration of the event and outcome (Stillbirth)?

The doctor said after seeing the report. No the doctor told that the child has expired, then we had gone to Pochocha at eleven in the morning at 10.30 am had shown him to the doctor after seeing the doctor, he had an ultrasound. Had written it must have been 11-12, after the ultrasound came So the ultrasound came, saw and said that the child has expired.

5.2 What was the cause of stillbirth mentioned to you and what is your perception about the value of knowing the cause?

So did not say anything <did you ask> Madam my mind was bad, what did I have to ask that expired I had to stop brainstorming there is so much security that I have to meet the doctor Did not give Now when its child became child, then its health had deteriorated inside there It was a problem because of this we did it somewhere.
Now, fever, happiness also started coming from it, so we said that if you prescribe medicine, there was no one to give you a tikki Go bring it, players so we said Sir, get it out of here, and we will take it. That you have to take it, then take the sign cards and take it. Would have studied there and kept two more days.
We used to wash our hands from that too such a position was done Half of the time I came home was correct. Healthy Man also becomes patient there.
<willingness to know the detailed cause> I mean madam I did not get time to talk to the doctor. Did not let us go there. We were worried about how the child had become shown here and shown here. In that month, since then, Normal was telling all the reports I have come to leave the paper to make it. So the doctor said, tell me how all of your things were correct in that month. It told everyone that everything is normal, so how did your child finish If you are in this position after today, do not come to this hospital. She shouted profusely. Now he has 6 months leave and increased it so we said that you stay home for 2-3 months.

5.3 Do you blame someone/something for the stillbirth?

Whom to blame We blame our fate Whom to blame <Any complaint> What to complain, Madam When I was not in luck, what can we do We have been exactly equal for 6 months Everything is done in 4-5 days.

5.4 How was your experience about the procedure after death till handover of the body to you?

Did not say that I was taken in the night on Monday I am not getting any of this baby so it may be that the child is over husband wife broke up She started crying. We said don't worry, first let the ultrasound happen. If we did not go to two places, then we went to S Hospital <delivery time and process> so at eleven in the morning we went to Pohoc at 10.30 am had shown the doctor He had written an ultrasound after seeing it.
It must have been 11-12 hours after the ultrasound came so the ultrasound came, saw and said that the child had expired <Handover of body> We had brought the clothes down When the child was born, they said that you will see the child, a woman is light-hearted, it ends up at the same, yet it was destroyed Born immediately gave it to her hand that it was born dead is.
He should not have said that he did not do anything, took the child and put it in the trolley Then we went and told us that you will see the child, we said, show it then see this child is your The position of the child was very bad I do not know how he got out He had one eye.
The damage was done I do not know what happened to him I do not know what had happened It seemed like someone has suppressed him. <operation or normal> No normal If the operation was

done, the child would probably be alive. We were speaking we did not get to know at all. Because the child was boiling just like a balloon we said that if it was over.

Came here and there tt was told that there is no cover that was told <cost> No no nothing, did not read a penny from us.

<Support from HCP> No one said they will take the baby now or take it in a while, we said that uncle is there, we will be told to bury him alone. If you are ready, we will take it if uncle ignites we have taken the child and then get the wine made.

<paper> Nothing is a paper, we have deposited in it that no 6 is open and one had signed the file which was made in that file.

5.5 Who all from your family and community/neighbourhood supported you during this period?

We will be called alone to bury. If it is there, we will take it if uncle ignites, then we take the child again.

Now she was my sister, she is in Ghaziabad she came, came the same day the day the child was born she was and she had a friend.

6. **Post-event procedures**

6.1 What all rituals and procedures did you follow after stillbirth

It is as if the child is buried and is not shed. Now, like a big man, we make him follow now if the child was small, then he bury him.

(Family present) My uncle was <where does he live> I am beside him.

(Female-religious taboo) No We do not have women in the people we cannot tell the people, we do not go to the people, the mother does not go when we took the child it was one o'clock Immediately we did auto go to Kale Khan, buried on the same. (document) Yes, I had given that we would ask for it there. I had given a paper from S Hospital that if I ask for it, give it. (cost) No expenditure was incurred, medicine was spent, and no expenditure was incurred.

Our mind was not working said the clothes the clothes were taken that was there, the nurse had cleared it we had given the clothes she had worn the same the nurse He had worn it wrapped it and gave it, “Take it.”

6.2 How did you, your Husband/wife and immediate family members cope with the difficult phase?

Now she was my sister, she is in Ghaziabad she came, came the same day the day the baby was born she was and she had a friend she also came to see, She was saying, discharge our patient, we will not show up here It is completely night He said to the doctor very much how are you talking at which time we They will say that you are not seen 11 o'clock patient admitted in your hospital, there is no hearing yet.

You said that the child has died how the child died What is the proof that the child is dead, and the mother is alright then why not the mother Hey, when the child is dead inside your abdomen, that time there is a problem and After 10 minutes the child is done so we finished the matter there. Both of them were there, they went to the hospital to see the younger sister was the same she continued to care. Clothes were taken and buried like that… Did not clean something, our mind was not working said Clothes were taken that is where the nurse cleaned them.

6.4 What were the reactions/ counselling and suggestions given by the relatives/neighbours/community after the episode?

He went to see that brother is so ill of daughter -in-law My uncle and aunt had gone ‘We kept on explaining that what has happened has already happened what to do it was in my fate and all this was there in delhi.

Here who is my own, we don’t have too much of kinship, we shifted here on 11$^{th.}$ Initially we were there we enquired about it we saw when he was being taken away we sw that When the child was born we saw it all were talking to us nicely all were explaining us.

7. **Knowing the cause of stillbirth in detail- autopsy and MITS**

7.1 In your view, what more could have been done to know the exact cause of stillbirth and the reasons causing it?

Now Madam, what can we do? Now what can we do and if at that time we could have met the doctor asking that what happened that we have made a mistake if there is a sprain in her leg so we could have felt relieved that she has got a shock from somewhere. She do not have to go anywhere from house

for 3 months she did not go anywhere sister-in law was called all the work was done by her. I had gone to call sister-in law 1-one and a half month earlier.

7.2 Are you aware of any method for identifying the exact cause stillbirth?

Said, it would make the child's condition worse, Madam who would have done the post mortem of such a small child on the day our child was born we had to keep the soil separate there were many children Most were not born alive we forgot our grief after seeing people what wrong they had done that this happened with them.

7.3 What are your views about the autopsy/ post-mortem for identifying the cause of stillbirth?

No, it is not something that is not considered good I have my own thinking. If someone wants to get information, then the postmortem is the one who makes the person what to do body get it done Panchanma and take it home there is a case, then the incident is different for the accident so it becomes a police case let's go for post mortem.

7.4 What are your views about collection of the tissue and body fluid samples for identifying the cause of stillbirth?

Now, I did not have so much knowledge madam that was the point. Had there been any person, more educated they could have told us something about it so we could have known that how it happened now we did not had so much knowledge had it been such a small thing so we could have got it done.I would have known that what the problem was. Now so much information was not available. <permission> for such a small thing, we would have got it done at least we would have come to know that what was the problem in this child.

7.5 How parents and family members can be approached for the tissue sampling?

Now who should we tell now this advice can only be given by a doctor now if we could have asked a doctor that what was the cause of death, why did the child died so they could have told exactly what happened they would have told us that you to do like this if you want to know then we could have done like that. Advice to wife can only be given by doctor

7.6 In your view, what factors are important for acceptance of the tissue sampling by the parents and family members?

Now I did not know so many madams, it was like had there been any educated person had he told us about it then we would have thought.

8. Summarization

8.1 In your view, what can be done to improve identification of cause of stillbirth and efforts to prevent?

Now this is happening because of the huge crowd there I understand that what can a doctor do it is so crowded people are so upset if 20 patients get discharged from there so at least 50 patients come there government hospital what will they do There is so much dirt there.

In-depth Interviews of parents who had Stillbirth-5

**1.1**	**Primary respondent (relation with the child)**	Father, Mother
**1.2**	**Age of the primary respondent**	Mother-25-30 years<br>Father-30-35 years
**1.3**	**Other members present**	Grandmother, Aunt

1. **Care seeking practices**

1.1 Where did you usually go or whom do you consult for routine antenatal check-up duringthe last pregnancy?

He had taken treatment in MM He started treating these people in M nagar only when When called for check up when I was able to take it I could not go and sent it to someone I am in M Nagar in MM Hospital continue to treat them last week one week in ninth month The first one was the next day of Muharram that day was When the checkup was brought by When all the reports It was normal, etc on the sixth day I told me that I am having such pain I will take M nagar Again in the city took it from here around 4 o'clock after moving, speak there, do not know, check Did not done are saying that the beating of the child has ended have you shown there since Beginning Exactly He is showing the same from the beginning I showed both my children there and both of them. Done was the routine check up done there only Yes don't know about months They have consumed medicines according to the They have eaten the claims according to the months in M Nagar.

1.2 Where did you usually go or whom did you consult for any problem during the last pregnancy?

He had taken treatment in M nagar He started treating these people in M Nagar only when he Called he sent for check when I was taken I could not go with anyone. I am in M Nagar in MM Hospital continue to treat them last week one week in ninth month The first one was taken the next day of Muharram that day was taken when the checkup was brought by when all the reports, etc.It was normal on the sixth day, he told me that such pain is coming I will take you to M Nagar again.

1.3 Where do usually the pregnant women from your locality/community go for check-up and problems during pregnancy?

(Father) - Mostly, MM Hospital will go or what is the name in S Hospital or Private your wish is done Batra is done who has more money No man will do according to the situation So now you agree that you have money to eat and you are asking where the private gift will be from.

1.4 Please specify the reasons for the preference for the facility and/or healthcare provider.

(Grandmother) Doctors are the only form of God Everything has knowledge they have knowledgeIs found in writing studies Neighbor cases are as low as you can handle. He no the case will be disturbed by you guys, we will show it from anywhere Sell houses, Does anyone can do anything for their problem you can do anything you can do anything Are If the government is not available then it is private right that we are poor why go because we are government There doctors are good but big doctors Small Doctor Teachers and the child who is in end time When you start coming out, brother will give you a hand from 12 o'clock, admit you but someone hands Not injected We do not like that since that day, the hospital you do not mind because we see there Did not like the facilities Government has seen all the facilities but the doctors are negligent The wrong thing that should not be done by them which I would be senior, they don't come they teach They do everything if you ask me you will listen to their opposite answer you will not be asked for the opposite answer Not a little is kept here for the help of others two or three people are taken in one bed Operation The ones What is the answer in MM Hospital that no, you have to sit outside The official one is Rithali in Noida it is such a good hospital that there is no answer to it my sister Is inside Noida Just yesterday, a girl has been born She is from Rithaali Sheela Dixit has got it built.

(Neighbor) No Indira Gandhi now anyone has made it from my side we there Go

(Paternal aunt) There is also the ability to speak to God the doctor has the ability to speak Son where do you want to go someone speaks in S Hospital (grandmother) is very good every man Moves towards good

2. Events that led to death of the stillbirth

2.1 Can you please let us know about the problems, illness and events immediately before the delivery?

Speaking to me don't know, haven't checked up speaking to me The baby's heart is gone what happened in the last month as soon as I reached I am I have entered 10 minutes back telling me that the baby's heart is over M nagar he transferred from the city said to take S Hospital when I reached there at 11 o'clock Closer don't know what checks were taken for the sub it's been up all night since morning delivery at 4 o'clock At 11 o'clock we got the news that your baby is born dead

2.2 Where did you go or whom did you consult for any problem during the last pregnancy?

(Father) was taken to M Nagar for start up In M nagar, these people were treated there.
They When he called, he sent for the check when he took it I could not go with anyone.
In MM Hospital there was treatment for them.

3. Hospitalization period

3.1 Now we would request you to tell us something about the period of hospitalization for the pregnancy, delivery or termination?

I reached S Hospital there at 11 o'clock I thought I didn't know that the check was taken away by the everybody from morning to night gone Delivery at 4:30 in the night. He was delivered at 4 o'clock At 11 o'clock I received the news that the child You have been born dead then when I asked him to tell me that baby, when you believe I am finished if I have come to you at 11 o'clock, so you will have to take it at 2:30 pm He had to clear. It takes two and a half hours check-up wait-up after doing all this delivery 2-hour it is done in the hour so the doctor says to me upside down that you have not come to peace with the doctor.Here when the doctor comes, it will happen and cannot go any further. It was Sunday so in Emergency You should always be a doctor a doctor cannot be found somewhere due to emergency Is Was not a doctor all night Got set up in the morning my condition Our mother stopped, my Aunt stopped Then after that came the mother-in-law of the younger brother, and aunt came to see my child Saw empty we did not show anyone they saw empty

3.2 Could you please describe you overall experience about the hospital care?

(Father) behaviour who like we would not let me go who speaks to me. Ok I have to get my check up done but his check up continued the rest of the report kept coming up What happened to this story in the last as soon as I have reached I have entered 10 minutes back It is said that the baby's heartbeat is over at the time of delivery which hospital & Yes MM Hospital Have got it done yes same doctor or different used to be different Last went to S Hospital when MM Hospital gave the transfer This is the fate of the people otherwise they had no faith in the stomach for 24 hours Normal Happened this is also the will of God No one has put a hand and when it is over It is over 3 days ago saying what is the answer for three days three days ladies will leave when baby Will it be over, will the ladies run for three days we went at 7:30 pm we said what is say of mother. Is Say nothing it is a little painful so we said that you did something Say what to do in it.The child will be on its own this is the answer to this how wills it happen on its own we said look Let me do something Now the baby is not in my hand it is left at 8:30 AM after that Tell Why do we go because we are governmental there doctors are good but big doctors are small doctors who teach and When the child starts coming out in end time, then you will put your hands from 12 o'clock, you will be admitted Did but no one put a hand nor did it inject I mean don't even let go One case happened in front of our eyes in M nagar in front of my eyes for three days. The ladies were suffering. Mother and daughter both came If it was not happening then I would not leave it in the labor room. That mother and child are both finished finished the child has died Ladies also died.

3.3 Could you please describe your experience about communication by the various hospital staffs during hospital stay?

Their is fine see when we do not speak well, nor do they even speak well Will say there is something missing inside you as well like if you will speak in a hurry then you will Their nature will speak in that way we will not speak well Look at us, do not speak wrong doctor Because we talked to them in this way when they were asked too, they were very well Answered there is nothing for mother even now you will see now you will have a child by night 7:30 I will go buzzing because we spoke well to them .That means I spoke better to you as much as we At the same time there was we did not say anything wrong (mother) ok bass that labor room I was not looking at the doctors, just a little the nurse's gift She cries she is a little bit angry for the rest of the time she talks right where are you looking?

5 Stillbirth

5.1 Could you please tell us about the declaration of the event and outcome (Stillbirth)?

Our sister-in-law was her sister (Grandmother) who does cleanliness and neither did she come call inside Was He said that the child is done Called in, showed up Until the call came, we reached the top we Went down for some work called us up, so his elder daughter came down so we went up Who started saying who I said his mother-in-law both of us, his mother, daughter-in-law's mother we both are one They had also come together Talked to the doctor first I asked how the child would say that the child was in the stomach three days before Was over This gift was shown in direct No, nothing told these people directly to the child Showed these people only did not show them to me they did not talk much empty child Shown where it is finished you can do only one thing that you can show that the child will be spoiled this child's whole The body is cheeked the nurse showed it was the doctor who showed the baby boy once Can show the child shows how it has happened the whole body of the child has gone cheek Says this much talk It was from Docker itself the doctor said that the stomach had been left over for three days it was said by the doctor Was before showing the child when we went in, it was sign on him I have a sign on it said how is the child Have said the child will be completely finished I said how the saying baby team would be over four days ago Three days Already, the child was finished, it has been three days, the child has finished the body of the child has gone completely showed the mother Let's give it then we called Uncle I won't be able to see the baby that is the time the child showed us Was to their younger daughter's mother we asked how tender it is so they said that it is okay we said that Do not tell them, they will have to tell the button they will have to tell it first.

5.2 What was the cause of stillbirth mentioned to you and what is your perception about the value of knowing the cause?

Who would not want to know at all you will come and ask them instead why my child is like this Happened while the sari treatment was going well everything was checked up everything was going on and so Why did this happen in time there will be some lack some will be Reason it means some problem It will happen there is no communication or there will be no loose pint, how will it happen when There will be a lousy point only then it is so hard. So this thing should be told not to the doctor yes brother Happened Mind you, next time we will Honda and meditate and take care of that thing so that it doesn't fall short Come and then we are taking you to me too so you are looking at the night that is it When the child is finished This will be done, deliver it in two to three hours.

5.3 Do you blame someone/something for the stillbirth?

Doctor will be responsible why won't you be you were admitted at 4 o'clock in the morning and 4 o'clock in the morning What will you say while you are delivering what will your husbands say, they will fight there Take it over I say it is over Please clear it in that Do it after 2 hours You seem to be injecting something else to be a pan so you should clear it quickly in patient coherence Why is admitted go and check the patient immediately give medicine and treat There is no such problem God

knows, in our luck, they have no fault blame them oh no use Is By blaming, see, one should not be guilty, Tehran May be not in our case right now Do not meet by making things not higher than the world, four people have four things

5.4 How was your experience about the procedure after death till handover of the body to you?

Just showed the child then took him in that we will give the child in the morning He gave it in the morning the child said at 5 o'clock you will not take more than this we will not stop because at night where you will take people, you will not be taken anywhere. We said, "Okay" Was then in the morning when his brothers and sons have gone, they have given him support they have given him the whole seal didn’t show face even after I have given it by means then we have also sent mother from 10:10: 30am Removed it bring it to the biscuit give it to him he brought it he was a nurse, he fed Feeding (Documents) Yes, it was early in the morning I have a sign in the night the latter is mine and when it is early morning, it is my mother's father (father).(Cost) No expense, this is medicine, the pill has all come 2–3 thousand medicines, etc. have come two and a half thousand rupees I have come to take medicine etc. like they have told me to eat something they have brought it alllike hospital In 300-600 rupees was spent in the hospital which she had written out of the outside she kept the paper Will also he had brought it I was there If he had gone to bury the child, he had also taken money for burial They (Mother-in-law) do not let a little bit of forced money there

5.5 Who all from your family and community/neighbourhood supported you during this period?

Nobody is like this No one has a bass, the whole family has become one Yes, I was my aunt, my aunt's boy There were warts these people all went with us there were two vehicles the vehicle was all full No one from here go was given brother from here take it in front of me later you keep coming again relatives etc

6. Post-event procedures

6.1 What all rituals and procedures did you follow after stillbirth?

So they brought it in the same way, then they wrapped it and put it in the carton then in the morning When their brothers and sons have gone, they have given them together they have given those complete seal even after that The face has not shown Yes, I was my aunt, my aunt's son was my warts these people all went with us there were two vehicles all the vehicles Was filled up no one left from here was fed from here in front of me Live relatives etc will be for her just peace liberation punished well Is after doing it is done on the 5th day or the tenth day whatever Panditji did for him. Mantra for him, etc no one has spoken better than ever whatever is born Now all that Pundit ji may know to work.

6.2 How did you, your Husband/wife and immediate family members cope with the difficult phase?

I will be there for him just peace liberatio have cremated well later on 5th day Or on the tenth day, it is Havan whatever the Panditji has done for him Mantra for him, etc. You have also said better than ever whatever is born now that everyone knows only Panditji to work Maybe we have a program ahead what to do we can't do anything in it when I have No, what can I do What can I do with him I cannot sleep all night mind It is true in the affair of the child it has been 14 years since marriage In 18 years, if there was only one It is relaxed now there is not even one so do not sleep all night 2 delivery

6.3 How did the family members support you and your husband/wife during the difficult phase?

Somebody gave some suggestions Come here and our father said that take them and show them in the hospital what is the problem when they saw it and it was night in the morning, I said that remove it, I will take it in family in Noida when you did not have patience, why would you have brought it if you had more money Do not even let go

6.4 What were the reactions/ counselling and suggestions given by the relatives/neighbours / community after the episode?

7. **Knowing the cause of stillbirth in detail- autopsy and MITS**

7.1 In your view, what more could have been done to know the exact cause of stillbirth and the reasons causing it?

No we have no idea

7.2 Are you aware of any method for identifying the exact cause stillbirth?

No we have no idea

7.3 What are your views about the autopsy/ post-mortem for identifying the cause of stillbirth?

Of course should be given it will be known from which region the child has finished will know What the lack was of was there a weakness in the child what was there what was not there Same man's pat immovable is what he is killed by eating this child too It was found out what was it, what was not, what happened because of the what was lacking inside what was the problem It was like now they have said that the heartbeat will be reduced they should also do it suppose we do not have anything you will know either if we have to do this.

7.4 What are your views about collection of the tissue and body fluid samples for identifying the cause of stillbirth?

Madam, I do not even have the knowledge of this thing Yes, if I will come and speak for good work Nobody will mind and they will tell us that if you want to get done then tell me if we will ask you to come and say yes, does it again you should be ascendant any parent should be the only person Would like to know how our child went.

7.5 How parents and family members can be approached for the tissue sampling?

You can speak to anyone in our house you can speak to anyone in the house this is all we advise they do it by themselves they need to type Pick anyone in my house.

7.6 In your view, what factors are important for acceptance of the tissue sampling by the parents and family members?

What you were telling what will happen in the post mortem, you will tear the incision of the child we will also take the wheels you tell Are you okay sir now they will give away food, what are the benefits of sealing school and what is the use of it They also take it out I have seen it many times my boys go to sparrows take out the kidneys etc. There is no thing it is already taken out And cotton is given weight in it No I will refuse to accept post-mortem at all know everything from blood in blood There are germs and they themselves tell them that the body is a problem.

8. **Summarization**

8.1 In your view, what can be done to improve identification of cause of stillbirth and efforts to prevent?

In-depth Interviews of parents who had Stillbirth-06

**1.1**	**Primary respondent (relation with the child)**	Mother , father
**1.2**	**Age of the primary respondent**	Mother- 25-30 years<br>Father- 30-35 years
**1.3**	**Other members present**	Paternal grandmother, Maternal grandmother, aunt

2. Care seeking practices
2.1 Where did you usually go or whom do you consult for routine antenatal check-up duringthe last pregnancy?
(Mother) - Find out in the second month. Madam people come to the Anganwadi to inject Have shown them He injected ‘Sui Vui’ and administered medicine. Eat the same medicine
(Asked respondent not shown in hospital or clinic?)
(Mother) - She had gone to SV dispensary, and was official.
(When did the respondent call in?)
(Mother) - used to call every month. I went there too, then got injected from here
Sec Used to send checks there (in SV dispensary)
(asked respondent ultrasound done)
(Mother) Yes, I did ultrasound.
2.2 Where did you usually go or whom did you consult for any problem during the last pregnancy?
2.3 Where do usually the pregnant women from your locality/community go for check-up and problems during pregnancy?
(Mother) - These people are seen in the same dispensary K villages.
2.4 Please specify the reasons for the preference for the facility and/or healthcare provider.
(Mother) - Here is the dispensary hospital, (Asked respondent is free treatment therefore) (Mother) - Yes, since living here, it is seen that as many pregnant people, children If you are sick, then you take them to the dispensary, stick the card to the wick For, not free of charge (treatment), which is given by iron tablets, one month They give to eat, they say the same.
3. Events that led to death of the stillbirth
Please let us know the course of pregnancy and events around the delivery/ termination.
(Please document as the event were told by the respondent)
(Mother) - Yes, it had been done since 6 months there was no one in the house nor was everyone at work so my husband was also at work, I did not say that my mother in law goes in the morning. At 12 o'clock, if there were none of them, then the health worsened, such little pain the water broke as it happened. After seeing the people, they called my beautiful people, then these people came and they called their brother and then took him to the hospital, In S Hospital. These people said that take the S Hospital water had broken in the house. Auto, S Hospital went straight after booking. Went to S Hospital if I took the check again If the sub wake is done, then the machine does not look in the stomach, to check the ground, then felt So, the child is upside down, it is not known whether it is moving or not If it has to be operated, the child is out of hand. Then operation will know if the child is alive or is finished. Then operation after it was found out that the child was suffocated inside.
3.1 Can you please let us know about the problems, illness and events immediately before the delivery?
(Mother) - No, there was no such problem. Woke up suddenly Speak if fever is uplifted in hospital. Those people too, doctor people I did not have any disease.
(Maternal grandmother) - No problem at all, when I came, everybody said went to S Hospital. Now if I went too, the doctor did not allow me to enter.
Something went wrong at the time of leaving. Don't know what is going on, first
The child was born there too. This child happened at home. The first child was born in the Holy Family.
(Maternal grandmother) This was the number three pregnancy.

(Mother) 2 children were finished.
(Mother) - Take it, one was finished in private.
(Maternal grandmother) The first one is finished and the last one is finished.
(Maternal grandmother) Everything was right, there was no problem, it was very right. I do not know. When I came, I took it to the hospital, its first night He was in pain, but there was no one here, he did not even have a mother, we also Was not alone, did not speak to anyone alone.
(Mother) - Mild pain stops waking, waking up, and water on the way had broken.
(Maternal grandmother) - It was 7-8 p.m.
(Mother) - My mother in law goes to work And at 12 o'clock at night, you come to work, Father in law also goes.

3.2 Where did you go or whom did you consult for any problem during the last pregnancy?

4. Hospitalization period

4.1 Now we would request you to tell us something about the period of hospitalisation for the pregnancy, delivery or termination? (Probe: hospital, ward, duration of stay, course of illness)

(Mother) - No operation. 3 no. Ward was no, number 3, stayed 4 days.
(Maternal grandmother) - When this baby was born, the nurse called and brought it home, suddenly,
S Hospital will not take even 2 minutes of it, this 5 year old (child), suddenly happened at home. There was a nurse from us the child does raw news, they told him to come, the nurse gave him a comfortable baby, there was no problem, Happened in normal.
(Paternal grandmother) - happened in normal, happened at home,
(Mother) - It was said that if S Hospital were to be in the normal, then the child got out of hand.
Had it happened, the hand was out, so the operation was done
(Maternal grandmother) - Mother-in-law tells daily, she tells it only in the morning.
(Paternal grandmother) - From the time when I go on duty, I come every day and ask,
The pain is not many, there will not be many problems, we go to work everyday and then go to work, at 4 o'clock I leave at 11-12.
(Maternal grandmother) - Even on that day her mother said that her mother is hurting a little bit, she
At the waist, at the bottom, if the child is in the stomach, then it hurts little, his mother-in-law Did not care, mother in law had gone to work, we were not even here, it was not even, baby was taken, she was in shock, the child came straight away the ultrasound also came straight Child, absolutely straight, in our mind, someone went by auto vote, pushed hard someone down below.
(Paternal grandmother) The path is not right here, it is bad till the main road, from there The path is straight, there is no problem, everything is a problem from here to here, pit low pit low There will be a slight shock in the body.
(Maternal grandmother) - And the child also weighed 3 kg.
(Mother) nurse came inside she said that you will have normal delivery. Then these Talked to the people that the sign is two normal deliveries, they were taken inside, after going to check up again, the child said that the child is upside down, so the ground is not known. Has been, again speaking that baby donor, when he took it to the delivery room, then said baby donor

4.2 Could you please describe you overall experience about the hospital care? (Probe: investigations, treatment given, satisfaction level, quality of care, cost of care)

(Maternal grandmother) - Yes, the cost is very much, brother. Not by operation
Happened (Mother asking how many days stayed, so relative), 3 no. Ward was No. 3, Stayed 7 days
(Mother) - The medicine given from there that give the pill to eat in the afternoon He has given And then, while giving leave, wrote medicines out of the way.
(Maternal grandmother) - Nothing given, no medicine water.
(Mother) - We write from outside, no one writes from the hospital. That Day medicine needle, then out of the house, gave medicine
(Paternal grandmother) - If it was 7 days, then medicine was also everything for 6 days gave. And discharged, gave medicines at that time and out, then we Bought out

4.3 Could you please describe your experience about communication by the various hospital staffs during hospital stay? (Probe: who all communicated, who was the primary communicator, nurses, doctors, other staffs, frequency of communication)
(Father) - Doctor Madam had called me, asked, which will be the operation or did you want to get normal or get delivery done? I said do as you like, sign in normal and then go home I walked, after that I did not go inside again.
(Paternal grandmother) - The time when I came to sign in normal It was asked that, I have said that my mother has signed in normal, I have heard, Doctor Said to be in the normal Sitting, he had also tried that it would be good if it was normal, then seeing that Will not be normal, then say to them send it soon, it is not down, baby Here it is stuck up, then said hurry to take them quickly and their operation Will be like this Then they were taken, then they were not allowed to go again, then later So the baby happened Then later gave it to his Behen, then sister gave it to other Gave it He brought it outside, then I sat and showed it there, so I neither Seeing that he fainted. I did not faint, so I told them, A little show me once, your sister said that you are not Will not see, what will you do, then I go from him, I will come to see then Who will see them?
(Mother) - Doctor people were right the doctor who was acquitted came to talk with love She was giving medicines, she used to give medicines, then nurses used to come and see, so one The nurse used to shout a little. Meaning as I am a patient, see you will need someone to do it. As the mother has stopped here, so, along with coming I used to shout, come out, why you keep crowd, sit down did not give, did not let the nurses shout a little. No my night I had a stomach ache, told my behen he had given me medicine at night.
(Mother) - Nurse, who is a ladies' person, not a doctor, a nurse, she told, we are behaving If you were doing it right, then you were saying to me that the child was not hearing Is, upside down, then said, Dharkan is going on, it is not known, operation Direct spoken operation will be done.
(Mother) would have made up her mind to drink water.
(Paternal grandmother) - A nurse had come to the doctor, so he was speaking Kajal Who is with the patient, nor who is with the patient with Kajal, neither do I, say wake up, these after seeing it has already been fulfilled, then it is saying that the pad below them is It got dirty remove it soon, but I don't have anything, now I don't know where the shop is. Speaking to me, then I patted it It was then that I went to change it, then said that don't do good clothes apply. You go to the shop, Madam where is the shop, go straight from here Jake Asking will show the guard. Hey, I asked the guards to go then they showed me here what the shop says. Where is dispensary, where is it called Go straight madam, see it, do not look outside. So I went. Go and get 3 packets the pad came from the locker. Come again and open it, I put it double double twice Took them a bad one and put a good one. Then returned the cloth was, then changed the cloth back, put it to sleep.
(Maternal grandmother) - was not very kind
(Father) - It was a little too right, a little wrong too and it took rest and took the nose off of it, took it off the ear, there, gave it off, the rest gave to the nurse.
(Mother) given in the hand of the nurse I told you that your husband is perfect outside Will give it to my husband, then went to ask for my sister. We don't know Gone. We gave it to people then my unhappy people did not tell us of their nose.
(Maternal grandmother) - Let's get lost, lost.
(Paternal grandmother) - I told her this, if so many burrows You have kept you in the stomach, when that person will go stealing the world, what do you do with sleeping Will, I explained it to him. If this child survives, you’re as much as the operation was done, if you could see the baby once in your mouth, all you’re the dog will go, let's have a baby. Even the child is not covered in the operation what it would be like in Jin, I explained it to you, what does mother say.
5. Stillbirth
We would like to know about the events around delivery and after that in the hospital.
5.1 Could you please tell us about the declaration of the event and outcome (Stillbirth)?(Probe: who declared the outcome, the body language and expression, preceding events before that)
(Mother) - Those people are speaking when they made checks and not when they were speaking to me.

Asked if the child's heart is not moving, was the child upside down, is it upside down, alive or May also be perfect. Heart is running, you will know after the operation. So I took the thumb sign from my hand from me, in the paper, after it happened so everyone is saying it is over. Yes (after performing the operation told that the child is not) my sister had said the acquitted one.

(asked respondent who told to your sister) The doctor (told the sister). After recruiting these people again my sister People called, in the hospital, acquitted, let a person enter.

5.2 What was the cause of stillbirth mentioned to you?

(Mother)- No (nodding head).

(Father) - When he said he wanted to know the cause of stillbirth that cost has been given, then we did not say anything, then we came, at night taking the child, no reason not told, no such thing.

(Mother) - No, I was unconscious I had an operation in 4.20 minutes, conscious I am at 10 o'clock at night.

(Paternal grandmother) - The time that she was conscious, we were the first Whose baby was brought from the operation, gave the child, even then we will handle then later If I regain consciousness, I will give it to you, then in half an hour from work to the same to their sister Seated, sitting there, sitting, watching, don't you take them in the slurry If she is looking, then by looking at her hand, I recognized that Kajal is offering right now. Then income Tha said look, your patient, then I got together, the doctor saw it she also got into the elevator then took another one from there, then go to her Put to bed.

5.3 What is your perception about the value of knowing the cause? (Probe: knowledge about the cause of stillbirth, willingness to know the detailed cause, potential factors that led to the illness/death, potential impact on the other familymembers or next pregnancy)

(Father) But to whom I would ask, if the same guy does not let go to talk then How will I ask, when everyone was saying how it was over, now the dharna was going on Was, gave the baby immediately, madam did not take even half an hour, by operation Gave the child, told everything, did not know how the child died.

5.4 Do you blame someone/something for the stillbirth?(Probe: who or what is it, what did you do in this context)

To whom should I say (father) guilty, now I became like the one who was destined.

(Mother) Don't know who I will blame now, they are saying it is over. To me then I said that the child is upside down, I am not able to know, I did not say that it was over It is in the stomach itself, I said that I am not aware of the opposite.

5.5 How was your experience about the procedure after death till handover of the body to you? (Probe: processes, documentation and papers given, respect for religious norms, time taken, cost/payment, assistance from the staffs)

(Father) 10 minutes 5 minutes something like this, first the child is finished then The operation was done, let's find out again that blood is needed, then it was also closed here To give blood from 10-12 went, then suddenly it is heard that its The operation was done, then the child said he was dead, then brought the child after 10 minutes Let's give He had given his sister (child), wrapped in cloth and tied it Itself Meaning he gave everything. Only one dam was dropped in bar.

(asked respondent about cost / documents)

(Father) No such expenses were not incurred, not given anything (documents), yes the discharge paper was given.

(Mother) - Yes, it was given (document) I had given medicine in it.

5.6 Who all from your family and community/neighbourhood supported you during this period? (Probe: who informed them, who all came, what type support given)

(Paternal grandmother and father) That day, we all sat outside.

(Mother) Yes, I had called again, no, after enlisting, everyone called. (Paternal grandmother and father) We were sitting down.

(Mother) used to let someone in, only one. Not many people know let’s give

6 Post-event procedures

6.1 What all rituals and procedures did you follow after stillbirth? (Probe: body preparation, who prepared burial / cremation, other rituals and timings)
(asked respondent usually burried or put in river)
(Father) - After that, we had brought here, we had brought autos here, had brought my father (child), had taken 2–4 photos, then we buried him.
(asked respondent usually burried or put in river)
(Father) - No, let's bury, no, all this was not done (asked respondent about Bathing, wearing new clothes).
(Maternal grandmother) - This child is not taking a bath, given 5 licks of water.
(Aunt) - Water was given.
(Paternal grandmother) - gives 5 teaspoons of water, 5 teaspoons of water by hand In the body, they have not seen the world, no one has cried, they have not done anything.
(asked respondent if read kuran sharif)
(Paternal grandmother)- Here he came from his stomach. Not found in this affair. We 5 strips Water was given, little digging pit. In him we die like we humans Goes, burys, similarly kept the child in such a manner.
(Asked respondent if read kuran sharif)
(Paternal grandmother) - Kaal will read, tomorrow is jumma or not, tomorrow will read.
6.2 How did you, your Husband/wife and immediate family members cope with the difficult phase?
(Father) - What can be said in this?
6.3 How did the family members support you and your husband/wife during the difficult phase? (Probe: burial/cremation rituals, community norms/rituals, coping with the phase, financial)
6.4 What were the reactions/ counselling and suggestions given by the relatives/neighbours/ Community after the episode? (Probe: what to do after death, following the rituals, knowing the cause of death, any other)
(asked if neighbours accompanied to burial ground and counseling/suggestions given)
(Father) family also, friends also got (support). Everyone came, at that time so all of them were deserted. After seeing the child, I sent them all outside came in the last around 1.5.
(Father) Yes, (neighbor), yes they got full support.
7 Knowing the cause of stillbirth in detail- autopsy and MITS
Many pregnancies in our community end with stillbirth due to various causes and in several instances the exact cause is not identified. We need to know the causes of stillbirth in detail. We want your views on the options of knowing the causes of stillbirth in detail.
7.1 In your view, what more could have been done to know the exact cause of stillbirth and the reasons causing it? (Probe: investigation, autopsy, any other)
7.2 Are you aware of any method for identifying the exact cause stillbirth? (Probe: autopsy, investigations, etc.)
In several instances, doctors advise for autopsy/ post-mortem to find out the causes of stillbirth so that appropriate action can be taken to prevent recurrence of such event.
7.3 What are your views about the autopsy/ post-mortem for identifying the cause of stillbirth? (Probe: necessity, religious aspect, time needed, disfigurement, etc.)
(Father) No, not doing post mortem, there is no one who has done that It was meant to be a madam, which went away, now it was cut out of the pit Do not like it. In our Dharam we do not even get beaten up, even if you are a child. Are elder that's done.
(Maternal grandmother) - Baby, the world is not visible. To attack what is the use. If we have luck, it will happen again.
In some instances, autopsy/ post-mortem is not possible due to various reasons. To find out the exact cause of stillbirth and underlying disease, some tissue biopsy (collection of very small sample using needles) and fluids (blood, urine, etc.) from the body, as done for investigation. But these samples are to be collected as soon as possible.
7.4 What are your views about collection of the tissue and body fluid samples for identifying the cause of stillbirth? (Probe: necessity, religious aspect, time needed, disfigurement, etc.)

Not (father) what is done, it is Madam, who is gone is gone. Buried now no more.
(Mother) - How long has it been?
(clarified that if MITS was advised to you at that time of stillbirth)
(Father) - Yes, that time would have been agreed (during stillbirth). Then we get it done.

7.5 How parents and family members can be approached for the tissue sampling?(Probe: who, when and how should approach and whom)

(Father) - If my father's mother understood him to speak, then we too would have agreed. We should have known not what reason the child was killed.

7.6 In your view, what factors are important for acceptance of the tissue sampling by the parents and family members?(Probe: desire to know the cause, trust, confidence on the health care provider)

(Father) - At that time 79% I would have agreed, yes I would have spoken, 2% would not have been. (Further asked respondent the reason for refusal) I mean my family If he had refused, I would have forced myself, I needed to know as a child.

8 Summarization

8.1 In your view, what can be done to improve identification of cause of stillbirth and efforts to prevent?

(Father) - I want to say that as soon as my child is finished, as if not outside Called inside, we were two four, its mother, whoever it was, invited two lives take it in, talk like this is such a thing that your child is finished. So give that time to you or give it to you later and the ladies are ill, and come they will run with the child, take the killed child, then the person who is alive will also die.

I mean to tell you after half an hour, such an operation has been done, your child It is over, it was such a problem, it was a problem, because of this your The child died. It doesn't matter at all, the child should leave it, Gave us the baby, we have come again, the people of the house, their sister from above, we So he was not up, his sister was up.

(Mother) - Did not even let me neither meet anyone nor let me see the child they had already given, they showed, I did not even show.

In-depth Interviews of parents who had Stillbirth–07

**1.1**	**Primary respondent (relation with the child)**	Mother
**1.2**	**Age of the primary respondent**	Mother 30-35 years
**1.3**	**Other members present**	Grand mother

1. Events that led to the stillbirth

1.1. Can you please let us know about the problems, illness and events immediately before delivery?

At first it was absolutely correct when I went to S Hospital, it was still a good thing and after that my blood had gone On the second day, his heartbeat did not decrease then he spoke The child died in the stomach (When this happened) only when my blood was shed not a day ago (Which month) in the month of November tThere was no problem I had trouble for the same eight days, there was no blood in my body.

(Mother) I do not know as soon as the blood starts climbing, the child was moving well, blood first what happened now what exactly was our destinyWell we have gone from here, the child was right, it was moving now there is a lack of blood so I understand something else? If someone has got a high or low foot like someone has read a foot in a dirty place or someone had a hand that's how blood will be reduced in their body in just eight days.

There is no such wind, it sucks the blood but there is no difference in the child, the child is not moving the child has turned around a lot He has gone to M Hospital, has gone to S Hospital when after all that has happened to their child Now they also know what has happened (Mother). As they had five bags of blood, they had blood as if they were climbing two days And there was a two-day climb in the night and there was a climb later as if there was a climb that day delivery was done

1.2. Where did you go or whom did you consult for any problem during the last pregnancy?

(Any check up done before) First it was done everything was absolutely right It was just again in eight and a half months, it happened only then it was given a date of eight and a half months so Do not know again did not show anyone around it (So, you were at home only) Yes No went there did not show up to M Hospital.He said that the child is right want to deliver it to be done here then we had come from there then went to S Hospital had said on the vehicle that blood There is a lot of lack, there is no blood in you (Is this during the time of delivery) Yes No we did not show No one was told There was a lack of blood He had previously told On the first day, neither did we go, he said that there is no blood in you two people need blood the doctor said that to me. (In M Hospital) No, I went to S Hospital for the second day His brother was gone and he went then he gave blood then we had blood transfused

(Mother) I do not know as soon as the blood starts rising, the child was moving in a good way, I do not know what happened as soon as the blood was going, the beating of the child on the second day On the evening of the night, the heartbeat is low in the child What are you

Hospitalisation period

2. Now we would request you to tell us something about the period of hospitalization for pregnancy, delivery or termination?

<Duration of stay> Stayed seven days (Communication by HCP) Then he said nothing that the pain will come only then the delivery of the baby will be the pain was not there at all It was good to come They were just and on the third day the medicine was created (mother), even though they were not even hearing seven days were like this our loved ones our housemates Is a friend of His father-in-law called that there is such a thing then he has come here then he has met the doctor.

3. Could you please describe your overall experience about the hospital care?

Now it is not possible to say that the child is short or finished like this he explained in such a way that your delivery will be correct your child will also be right and the daughter will be correct. Explained sympathy must have given the brothers not to panic they have talked they have taken care of them

again for the evening the medicine was put again, it took off immediately then it took it 1–1: 30 pm the boy is born
(Mother) Well I have not understood one thing I have it in the evening It told that the heartbeat subsided, then said that the child is finished If the child is finished then the child goes to the flower, it goes to the flower It deteriorates, then there was absolutely nothing of course, our is your skin. The face of my face is like yours.
It was clean and tidy our face is exactly like it was so it is said that it is a beat In the child I think it will be a beat we will Think that the day the baby was about to be born in the evening of the evening I had to move twice in the stomach the baby would definitely have a beating
<any investigations> one was done… not out. No it happens in the new building the investigation was done that they had extracted blood from their hands to see if there is no problem
<any treatment> yes medicine
He had given the pill, he did not give it to his mother, to eat He had given calcium and iron tablets everyone was able to get the same we got 4 points of blood. Gone 2 points 2 boys fired our boys removed our boys He used to come as a child he said that it was over, and then he did not see it for us No one in it, so in the delivery room, they did not let anyone go. A madam had come great doctors come when they came. They got me out. Was for outside.
(Mother in law) After the boy was born
(Mother) No when the blood was still kept in the same It was kept for three days. Where were you extracting it I was in it then Madam came, then she said that she did not have another one from here.
(Mother-in-law) Chai did not allow breakfast for anything We used to take our daughter-in-law how not to take her "Not now, no, take away take away" "Now is not the time"Now nothing will be eaten They do the same thing in many places We will let you take it again later
<Participation in decision making> No, I did not ask anything just used to check B.P as B.P. had increased my life just came to check the same and nothing Even if I used to speak, I used to give my teeth that you speak more What do you know more than us They used to remove them then we She did not speak more She used to check and go If she used to speak
<treatment given> Now what was our destiny right now Well, we have gone from here, so the child was right, he was wandering Now there is a lack of blood It is understood that someone or something has fallen down as if someone has read a foot in a dirty place or someone had a hand that’s why blood will be reduced in their body in just eight days there is no such wind, the blood sucks but there is no difference in the child, the child is not moving

The child has traveled a lot gone to M Hospital, gone to S Hospital when whatever has happened after the bleeding, his child Now they also know what has happened
(Mother) As if there were five bags of blood, they made blood like it was climbing two times a day and it was climbing two days in the night and there was a climb later like one The climb was delivered that day.
<cost of care> Yes He had brought what he had written to buy medicine He had brought it from outside for purchase He had brought what he had written from outside Have been sent have sent it outside too used to send it outside and check this report, it used to get its money too (Amount) Now this father will know that we have not asked So much.

4. **Could you please describe your experience about communication by the various hospital staffs during hospital stay?**

I used to come to the same just check and by the way no one came No one even asked if you were alright No, nothing just checked the check and went No, he did not speak anything Just keep silent check check done Just Didn't tell anything No.
(Mother-in-law) Four were known Son was born to them still asked in the morning for cots Three days have been admitted Used to give tea. Still returned meaning the tea used to get cold but I was not ready to give then I would have taken it back too Now it would have cooled so that I would have given it at my own wish Reach How will our light be got anything to eat, not even

(Mother) used to shout. And as they used to ask for something, neither they scolded

Stiilbirth

5. **Could you please tell us about the declaration of the event /outcome?**
   The one who had delivered the delivery showed me not the one who delivered the same but the girls were the doctor as you were wearing a white dress he said that seeing this your child was finished I had said so much
   (Mother-in-law) We told our elder daughter-in-law that she was ahead of us our elder daughter-in-law, we were a little bit behind so we said the elder daughter-in-law's name is heard. Did we hear a child, Sunita? How is our child? So our bahu said doctor, sir now how is the child? What is baby how She is not saying just ask that We said, what is the child, Doctor Sahab "Now I am not saying that the child is killed, the boy is born and is killed" "We said, show it I am showing you neither calm nor go Now I am showing
6. **What was your and your family member's reaction when you were told about the stillbirth?**
   Then I also told him to call my mother-in-law or my husband not called called at two o'clock.
   (Mother-law) We were our elder daughter
   (Mother) As if my stomach was hurting too much, I had told them to call my mother-in-law or my husband that my stomach is hurting so much He did not call anyone at two o'clock Called said that keep quiet and there is a doctor who does the delivery stay in the delivery room
   Now what do you feel We felt that the child died because of blood clotting or how it was finished I did not understand Now the child was right before blood transfusion was roaming Now after bleeding, the heart was telling them and their stomach was also pressing down like this Now I do not know for which baby I have gone Very sad Happened, I am getting If these people would say that we will do the operation, there is danger in the child To We were ready for that too the child is born like that so well The child is born
   As if there is no less beats ahead, the child becomes black or goes to blue Something happens at all Means one is born at 1: 1:30 pm on Wednesday night so something is lacking in the child Blood would go on or read blue or read black There was nothing It was absolutely beautiful. There is also a photo in my son's mobile His face is clean, his face is very clear, his face is like our face we feel it Ho (Mother - in law crying) such a big child hand legs were very fat We also used to say that there will be boy there are three girls who wants a baby more today It happens to us that girls are girls, so they go to their own homes some son-in-Law gets such asks, girl's parents Is the boy is just as right stays at home keeps on burning just like we wanted to do all the work right that is our only In mind.
   This was admitted our son used to bring shade for us we used to say, bring light to Jyoti He used to bring something for Jyoti, these people did not let it go inside Power came from me I can't even walk There is so much pain in the fall In the heat this recruitment so know where the strength came from, I used to run away in my life My heart was not recovered Stayed there for seven days I did not come home at home for seven days I did not even come to wash my bath.
7. **How was your experience about the procedure after death till handover of the body to you?**
   Same the boy was in the light wrapped in the clothes we saw the child. We also said that the child will still be seen. Want our family is at home away it will take time in the morning, we will be able to come at night so we said that what time will the child be told, then we spoke to them Will tell will say at five o'clock So our son said that father will give the child at five o'clock, if you want to come, then come from here.Yes, they made me sign when they said when Madam has said so much then they pulled me out They come from outside, Madam came Shifted to another room when the child was not born (after that when the child was not there) I do not know I was in and it was the mother and all I was born I was my husband and I was Jethani I was in the room I did not have the signal vegan I would have got them doneWe brought both the children (Whispering)(What did they say?) They only called us that, when the child has become whoever is there, who brings the child out, he was the one who called us who is with Jyoti Look, the boy is born Call them so baby is born at 11:30 am we call them at 2 pm We have gone again we have gone to our big multiplication he is a little ahead of us at the window This is the window We are a little here from the window.Because we are not allowed to

stand at the window We have said this to the daughter-in-law We have heard from the daughter-in-law to say whether the child has been born the child is born or not Are Madam what a child has happened said to whom Jyoti's so she remained silent She was writing something We asked We said something like Sunita said, "Doctor sir, what child has happened, where has the boy been born the boy is born dead I said that he was killed I said that there was no breath at all I did not Where are you Say calmly Take some time, keep your peace Just show it Have you kept the child Then this is the second time they have shown it Put it in the crate and wrapped it in green clothLike child gave it at 5 o'clock Nothing at 5 got our big daughter to sign wine and then they gave us the baby they said, take it away We had taken our clothes We asked that the ball which is a green cloth go in it or should we take it in our clothesThey said that you have brought the clothes We said that yes we have They said that take them in it only then this cloth will not go So then we bring our own clothes Yes 5 At that time, he said that he would get the child at 5 o'clock at 2 o'clock we were called told that the child has been born, the boy has been born then we will see You can take it right now Can't take it right now I said when you get doggy somewhere at 5 then call someone you say you I said yes you will call All the houses The family is.I said at what time do you kid at 5 o'clock I said maybe before 5 o'clock or at 5 o'clock I have not got my left, so I will come soon to give when our family comes

7.1 Who all from your family and community / neighbourhood were present at the hospital and how they supported you during this period?

<who all came> we were our son and we used to go and used to come used to come to meet us used to go to meet devar father in law used to meet No, they didn't go from the neighborhood Their mother's behen used to be there our daughter also met us and our sister also came

<who informed them> No we only Called this is such a thing and then they came to know at home too It comes that Jyoti has such problems and is admitted in S Hospital so many days have come Sister-in-law wins the phone and have gone to see once whenever she has gone when it is finished even when she has gone to collect the soil.

Post event procedures and coping mechanism

8. What all rituals and procedures did you follow after stillbirth?

Where children are kept, taken away S Hospital refused No place to keep children where you can take will Mother - Shiva took to the temple like let's bury it. (When you were coming back, where did you keep the baby, put it there) Here it is not the place to keep the child. Brought here for the same as they bathe the child to keep the child came there (in the hospital).

Refused to the hospital had brought it here We have it only a little distance away Shiva speaks the temple Jungle area is there the child who came from buried Mattress Dig it and come there

Do the same thing as they are buried now the child is not burnt this is where the mattress digs the clothes the cloth must have been laid or wrapped in cloth

Brought here the did not bring home when we used to bring home at home who would take care of me here again while taking two hands

<any prayer> no we do not come Panditji

(Mother) comes later

(Mother- in- law) Naming takes place or not it doesn't come before (voice break)

<rituals and timing> So much Can't tell the time It is there that you get trafic too Now these people will have come Got the trafic too The traficis not available, but if it is a thing, then it comes in 1 hour If you get a trafic it takes 1 hour or 2 hours.

(Who all went?) Now I do not know His father-in-law was my younger son was three brothers our brother-in-law was and will be known to know now who is Patan Who's gone

9. How did you, your spouse (husband / wife) and immediate family members cope with the difficult phase?

Madam is the same no one has forgotten Keep it in the stomach for 6 months If it happens then no one has forgotten

Now it is like those who come to meet, they give peacefully, and they will give it to you and they will give God does not want to have luck it is not like luck is it as if now seen He has come to give it now he explains that all are like this is what comes in the mind that where it is not taken
Even if I had to do private things etc as if the pain was not happening etc so do you know that the child would be corrected by the operation it feels a little bit Now, where is the time when I remember you still speak in your mind why did you know why this happened to us you would have shown it in a little private place there is no end right you think whether the baby will be born by the operation or not Will be born, so it is okay, a little money will be spent then someone said that S Hospital will not be a good job care will also be good will take good care. Goes home

Is/ was there any change in the interpersonal relationship between you and your spouse and other family members after the event?

They do not talk to me more than this, they say you will feel sad and they say that they have three daughters they will look at them they will teach them First thing, my husband does not say anything to me does not speak anything they say that they now have children, so they have to focus on them They say that they now have children, so they have to focus on them Yes, the support was given to me, it was also explained to me no my husband never leaves me even then I was in the delivery room even then to meet me to ask How are you like, how are you not? Even then they used to come even then they did not allow me to meet Just go to that room or come out of the dead or come out alive That's enough get someone or you to see us or we see you they are don't let anyone see don't even let anyone see you were taken to do so, I was very comforted that nothing will happen, there will be ultrasound everything will be safe you are everything there are no children that you are We just want to save you No, nothing like that No one has said anything Neither our in-law father-in-law speaks wrong to us nor our No brother-in-law, brother-in-law or Jethani says anything to us He does his own He eats his own There is nothing for anyone Nobody speaks wrong It seems like our child, we would do this for him He would do what to do.

10. **How did family members support you and your spouse (husband/wife) during the difficult phase?**

    Now it is like those who come to meet, they give peacefully, and they will give it to you and they will give God does not want to have luck it is not like luck is it as if now seen He has come to give it. Now everyone is just like explaining it this is what comes in the mind where it is not taken whether it would have been done in private things as if pain was not happening do you know if the child gets our right by operation It feels a little bit

11. **What were the reactions / counselling and suggestions given by the relatives/neighbours/ Community after the episode?**

    It is a matter of satisfaction that we are next to it there are others next door this poor person has not been able to come next to us her husband is over it has been two months Yes How will you come now (Voice break) We saw the photo of the child, the child is right After the end of the husband, there is such a thing that we will not go to someone's house let's see how Jyoti is (Other Suggetians). Nothing like that they say this your many children have come safe and will be a very good child Now we do not even know where to find such things look, their daughter was three months old when there was no problem the elder daughter-in-law has two daughters, she has a beta; she did not have any problem the younger daughter is definitely a S Hospital girl, the first one she was born at 12 o’clock It has been a full day since 12 o'clock, she did not cry she was born in S Hospital she She was alive for 2 months, she was finished.

    (Mother) My brother was gone, his girl too was finished, and I had gone, so my boy too was over there

    (Mother - in - law) The first turn was over now see the girl's house But he is right, he is right all three of your children were at home) Yes, they were at home.

12. **Do you think and /or discuss with your spouse/ family member about the loss and related events and blame someone/something or feel guilty for the same?**

<Blame anyone> Now what to blame, whom, who is to blame his destiny not even knowing that so can be called guilty in this case not only this, what will you say well there is no one in our house who said that this happened because of this, he says that it was not in luck, what will you do now he says
< What about you feeling guilty? Didn't get anything wrong, don't know what happened
Now we wonder why this happened who it thought that there must have been an affair like look, you may or may not have gone anywhere the soul that keeps wandering in it, etc., its cycle is over, your beta is over I think and nothing

13. **When did father and mother (if working) return to work after the event?**
He went back in eight days yes I stayed with him I stayed with him for seven days.
When did not come on my leave He did not even come home Now I have to pray, what to do I went on Saturday (talking among each other) When you stay in the house, you will be this bread, that's why you went to work

14. **How your and your family's life has changed after the event?**
Changes are nothing Now those who are Now they say that they are not there, what is the use of them you will also be happy, I will also be happy, whatever will happen will be seen

Knowing the cause of stillbirth in detail – autopsy and MITS

15. **What was the cause of stillbirth mentioned to you and what is your perception about the value of knowing the cause?**
Now they did not tell anything we were the only people, they said that they did not give the blood to the daughter, or for which the child was finished Now Pathan remains the same in our mind that he was right when he was not there when the blood has gone, when the blood has gone, the child has lost so much that the heartbeat is reduced And Kukuh has seen his stomach pressed by the people even if they refuse that the stomach hurts, the stomach hurts, they still do not agree Now what can you say, do you know whether it is different from suppressing their stomach or speaking to us now they are thinking a little bit that your stomach is looking a little looking at everyone do you know what happened to the child? It is not like that the midwife came to our house she did not press her stomach no she was watching (mother) she used to press this thing very fast. She is born here, children are born, even at home she did not press on her stomach she was not pressed at all (mother-in-law) and used to see if your child is right It will be done now you must know why we pressed the stomach for the teen. Now we will not know all this now this will be known.
<willingness to know the cause> Yes, tell me if you know then
In your view, what more could have been done to know the exact cause of stillbirth and the reasons causing it?
We have no information we also asked them how our child finished It is said that he did not even say that the beating in your child has ended, that's why he died.

16. **Are you aware of any method for identifying the exact cause stillbirth?**
No, I don't know (Do you know anything).

17. **What are your views about the autopsy/post mortem for identifying the cause of stillbirth?**
Now I do not know to beta, I cannot tell as it goes on like this, we should see why our child was finished, what was lacking in this <postmortem, acceptability> How can we tell now how can you tell without taking the house without a householder now someone in the house gives advice, so they think, tell me, you will find out so soon how can we say such I do not know (Whom do you ask at home)? Now as our boy is, his father-in-law can take care from him.
<religion> See, if we do some work, then we advise only that some work is done at home.
They ask that they do not get the work done we have some caravan, so we ask what you are saying like so, we can tell ourselves only after giving advice like this now, as we say something, right, children, tell the father of our girl, what did you say, why did you say wrong, it is also good does not happen A tongue stays in the house. It is good. It is good to get someone to work and let us not say, we are working with our stubbornness, it is a good thing how will it work like that I will say that you are ready to do as you wish, we are not ready how will it go, son will not work.

18. **What are your views about collection of the tissue and body fluid samples for identifying the cause of stillbirth?**
    We can only tell it by asking the family members we will say that the doctor came and was asking like this you tell me what you want tell me not to say that such work is ever done, please do not get it done
19. **How parents and family members can be approached for the tissue and fluid sampling?**
    Look at boys and by asking his father-in-law, then only we can tell well it is as if you want to check the blood and then it is correct. Do you have video Just like you want to show, you will see that it will understand more about what happens
20. **In your view, what factors are important for acceptance of the tissue sampling by the parents and family members?**
21. **In routine day to day operation who takes decision about the major activities about the family?**
    By the way, our father-in-law is there, he takes opinion from them, mother-in-law asks him here too His father-in-law is only in these things our father is (so the decision is to look by father - in law only) And then such opinions are taken the elder son is the elder is the daughter, he is yes, he asks it is what we understand that we do yes and those who do not understand It seems that it is going wrong, so let's say that this work is wrong, do not do it.
22. **During the hospital stay who discussed with the doctors / nurses to make decision about the treatment and other procedures?**
    By the way, our father-in-law is there, he takes opinion from them, mother-in-law asks him here too His father-in-law is only in these things our father is (so the decision is to look by father - in law only) And then such opinions are taken the elder son is the elder is the daughter, he is yes, he asks it is what we understand that we do yes and those who do not understand It seems that it is going wrong, so let's say that this work is wrong, do not do it
23. **After the delivery, who decided about the burial and other post- death activities and rituals?**
    His father-in-law had said he only said now there is nothing worth keeping how will it be kept there We would have brought it home for half an hour the watchers come to see now we were not the only ones, what was the benefit of bringing it the younger daughter was there our elder daughter was there in S Hospital.

In-depth Interviews of parents who had Stillbirth-08

**1.1**	**Primary respondent (relation with the child)**	Father, Mother
**1.2**	**Age of the primary respondent**	Father- 35-40 years<br>Mother- 30-35 years
**1.3**	**Other member present**	None

2. Events that led to the stillbirth
Please let us know the course of pregnancy and events around the delivery/ termination.
(Please document as the event were told by the respondent)

Father- Now he picked up and transferred us there in the night, let me have a car. Took, what would he do if someone else was a prisoner, then there he would have done pohoke at 12 o'clock He played 5. Going to see at 5 o'clock, at 11 o'clock he said that there was no movement of the child.
Mother- While I had done full time treatment.
Father- made full time treatment of two children at LH. Mam Looked low, did not give any antibiotic medicine after applying the sack, nothing but you Wash him, wash the story, when you put something in your lock, you check it, antibiotic medicine will not cause any injury inside, that wound has spread, B.P has increased we give the first 2-3 medicines of B.P. After that gave deep medicines, then Said B.P is control, I said leave, leave we are also getting worried,
My baby too two of my children were going to study in school, meaning on the 5th day we left paper wrappers again, but walk Now we will give the leave vote, it has been 5 days, not given he is engaged in making his brother subject. The big doctor tells the younger doctor, he is the subject for that, he became the subject B.P. How much are you offering, what are you doing? Baby in front It is not to be seen above the client, then go there and get it done in private, lock even went then showed the doctor again, he wrote antibiotics, hey hospital, tell us Let me bring antibiotics, write, while two my children in LH, every time they Put the sack, and they have written antibiotics, no antibiotics nothing, if someone's bathroom space deteriorates, one lives it will be difficult.
Mother - and what I would have done, there was no care, I went for the first time and did not get so upset too much.
Father- In this case, LH very well, I say, the best hospital there is a lot of care there is no way in S Hospital. Is that the only one there is a load, look there was a hospital of the near.
Mother - When the people of Malviya town told me to pay the discharge here, you said there go, write the referrer from there again, see my paper in referring, when my emergency It is the case that you handle me, do not make me, I stand in line from 12 o'clock, my number at 5 o'clock are there When I have a full pan, there is so much trouble, my child stops walking going I got treatment all the time, wait now, I see the paper itself, all the papers, every month who has to show the date of the 4th date, I go to the dispensary to check every month. Does Dispensaries did not tell you to go to the hospital and get your papers made Once even the doctor did not tell me that you are important, go to Malviya Nagar once Get the paper made, they did not even tell me when I kept going for 4 months, they gave the check up there Keep doing mine. Or you can get me ultrasound done outside. Report is being done from here, got it done from here now, got the entire check sub made regular, do not let it go today I am not going tomorrow, I go to every churn, refer here in Malviya nagar S Hospital those who did not care, Then the next day when my 5 o'clock, meaning my number came At the time they made my check ultrasound, pressing their stomach and then saying that your children It is not possible to make movements, when my child's movement is working, when my Do the operation, when I say do the operation yourself, why don't you pay attention to me are. I am standing there yearning there is something there, in whatever condition it means anything. I could have died too, My B .P was not so high, nor was it too high, that could have meant anything to me at that time. My hand pair was so swallowed up.

Father- Then you will get patients then you are saying that you give blood, I also gave blood, and blood after delivery of blood, the dead child was delivered.
Mother- When the children, 3 children, were delivered at 3 o'clock, my delivery was done at 3 o'clock.
Father- At 3 o'clock, I gave blood at 2 o'clock at 2.5 o'clock, they made it at 4 o'clock, 11 o'clock Slip, he said, give blood, it took me 3-4 hours to give blood, after giving blood, he Deliver, and the blood doesn't move, Mother-blood has not risen to me, nothing
Father- I asked for blood, I said that the blood did not rise at all. I asked, at the same time, I again later, he asked me, he told me later, it was inside, at that time, he did not even meet. Mother - I did not even meet. They thought that I must have climbed, they, meet me Neither was given Did not come in my mind that something like this will happen, that time Something like this, I had two children, there was no problem in LH, as much trouble here I did not go to life in this hospital. It is such a bad situation. Very much. (In a much raised voice) I had no problem, I had no problem, I had this child there was no problem there was no problem. Mother- mine is mine, who has kept it for 6 months, if its thing goes away how sad it is, when there is trouble when I say that no one is able to do anything. So this meant that I was having so many tests, I had no problem with me, the whole movement was doing baby, and at the end of time when I got in trouble, they didn't see much, within 2-3 hours, my whole case got worse.
Father- We got all the tests done.
Mother - Due to date did not come out, as soon as I started for 9 months, the pen was done. Yes, as soon as the pen was done, as soon as the pen happened, in the evening I got a pen, in the afternoon the pen started getting lighter. Light, if in the evening, as the pen is increased, then around 7-8:30, I call them, my pain husband has the job of transport, so he is saying that I am chariot. They made 11 turns in coming, by 11 o'clock, meaning the pain had increased. As soon as I went to Malviya Nagar, there was a pain. They Said that the child's hand is working, you are like this, you have it quickly so from here we let's do an ambulance, if you don't have any means, then I said no we will have come So you are saying do this and go to S Hospital. They wrote down we went, S Hospital left at the same time. Only after going to S Hospital did all my work go bad. S Hospital was spent from there.
Father- It was 12 o'clock.
Mother - It was 12 o'clock. From 12 o'clock I was in line, just there, at 12 o'clock line
My number arrived at 5 in the morning. When the doctor provided the check, sign it and do it there. Standing in agony, nothing meant that there was a crowd like a cow buffalo in it, so the place was so crowded.
Mother - In that condition all the work is done. First Malviya nagar half there
The number is chanted after the hour. When he came there, then went there again, take time there come and go. And the number did not come there my condition is such that everything should be done now Yes, they are saying come after 2 hours.

2.1 Can you please let us know about the problems, illness and events immediately before the delivery?

Mother-fever was nothing, just B.P was high mine was B.P high in this dispensary.
Mother- I was not crying in the car, when they put gas right, Malviya city people if it is written, he started saying that he referred for S Hospital. I said there is a problem they referred to me so I said that I will go home I am not in the hospital will go S Hospital. So they are saying no, I said either take me to MH or else then go to LH. So you didn't say if there is such an acquitted machine then why not see it. Will see you there
Father- I said the reference, now he wrote the reference, took it there. Malviya Nagar has given the reference to S Hospital, he will take he will not take.
Mother- They will admit after seeing the paper saying, they have not seen the paper, that paper so, watching from your time. There is a line as much as the public, the number is tied to the number whose number you are taking, you are taking from it whether it is a pain or not the child is sitting. This condition is bad. What to do if you see others Used to be.

2.2 Where did you go or whom did you consult for any problem during the last pregnancy?

Mother - This dispensary, near dispensary, near our police station, at number 17, Government dispensary, I have their paper.
Father- It is Government dispensary at number 17.
Mother - No ultrasound, he had written outside, I had done it outside only outside Aman is a hospital you are saying that you get it done privately.
Father - outside is private ultrasound, saying that you get ultrasound done outside.
Mother- He said only once, I had him done once, he gave me in the 9th month. Had said to get it done, had it done there, he did not even speak again asked the doctor now If ultrasound is not needed, then it is said no. There was a normal child in the report. Used to move when I used to move, I used to feel that the movement is happening. I was perfectly fine at the time when there was a pain, even at that time my child was fine, roam I was feeling like I was feeling Hanaji was completed (asked 9 months were completed).
Father- He was a child of all time, when the doctor took it in hand, saying that you're whole Child of time. Mother was a child of all time.
Hospitalisation period

3. **Now we would request you to tell us something about the period of hospitalisation for the pregnancy, delivery or termination? (Probe:hospital, ward, duration of stay, course of illness)**

Mother- I stayed 6-7.
Father- I was born on 11th Tariq, I went back from August 15-14
Mother- Yes, it came on August 14, meaning it came only after August 15, it was on August 14, and I had come
Father- came on 14 August.
Mother- They is saying nothing just to delivery
Father- 11 o'clock he said, my wife, my sister, when she has gone inside, she has blood It is written that, he said that there is no movement of the child, at 11 o'clock he said, he says Blood will climb your wife, you take this slip, go to the blood bank, so I take that slip directly and He went to the blood bank, after that when he went like blood bank, I gave blood till 3-6, 3 o'clock.
Mother - In August, 3 o'clock happened on 11 Tariq, 3 o'clock delivery of the child.
Mother- I was on 17th (discharged). I was on 10 (injured). Took place on 10 Means the night took only 11 (all 4 days).

4. **Could you please describe your overall experience about the hospital care? (Probe: investigations, treatment given, involvement/ participation in decision making, satisfaction level, quality of care, cost of care)**

Father- This madam actuallly is very bad for S Hospital here. S Hospital Hospital I have a lot of load again, the load will be so much, and secondly, when the patient goes out, there are no doctors, Doctor is not that much, a doctor will only see two, three will not see him, there is a shortage of doctor, Brother can't blame a doctor, so man is a doctor's man short, the other is one thing That there is a hospital like Madam Mohan, the government has made hospitals, but there are machines No, brother, no one will have weapons to fight, so how can that war Will fight The father-system is like this, Madam, (overlapping few words), open the hospital there. There is no senior doctor it is not a senior doctor. What junior doctor again if he is not in his hand, he will write further, then there will be no machines, no more stuff will happen.
Father-11 Tariq on August, and after delivering blood, he delivered, what am I Delivery will not be told, you are not saying, I said you did not say when, Then the doctor was silent, then this is not for me, I said that you have offered blood, it said that someone is blood Did not climb, did not even meet that day, then they gave the baby in their hands, then the children I came and buried him as soon as I came, I went in the evening, so that day I said blood rose If it is said, no blood is offered.
Mother-nurse used to come one day medicine also means medicine and BP The doctor said that it is very high, the doctor has written to give him the medicine of BP and the nurse is arahi means that even medicines are not given, and they are saying that your B.P is high, I said that I did not get medicine at all.

If you get medicine, then b.p will be down and I had kept taking me in a nice room, Sister came from there saying that you Mother-child go to the ward mother-child was so much in the ward that the children lie in Persia. 3–3 ladies lying in Bed and a patient with a BP are likewise the person of a stop.
It is troubled and the pitch is so cramped, you will sleep for a while only then sleep is complete BP.
Father- BP person will take his sleep, or the mother will stay in the mother ward. One is not a child of It is kept in the child ward.
Mother - meant nothing there. Never getting medicine never getting ever doctors is not saying they are not then at night they are saying that they should bring this check Bring, hey you will tell me when you feed me, then they mean me like this My BP down meant giving me a normal tablet, BP was not my normal one. BP Then the doctor came then he said that it is not normal BP. Night day check
The nurse is coming and the nurse is checking and is checking at midnight. But it didn't happen again they sent me to a doctor in the upper floor, that doctor again BP me the tablet is written. Then after writing that tablet I went home, meaning after 6-8 days I flew home, I had too much trouble there, meaning it was there The nerve of the brain will be fat. Did not see anyone there at all.
Father- BP means rest should be closed.
Mother- I did not like anything, I am telling you that even my strings were also caught, I so he got treatment in private. This was the condition. Nothing but urine tally was on me, taking a bag of urine I was wandering when you passed the urine my bag did not come out of you I carried my bag for 5 days and roamed in the park. I said, get this bag out of me, then the doctor is saying you did not take it out, I said when Delivery is being received, you should remove it, it is not removed. Walking around carrying bag is, the day before my arrival, the bag came out of my urine. Not mean of something. Listen to any problem in which condition the patient is lying No.
Father- Now tell me, antibiotic is given to dry the tablet, neither ma'am or no Amoxycillin is still available drying Amoxycillin 250 ml 500 ml is the cheapest It is 10-12 rupees, they give normally, they give it in LH, or it is good He is 60, he comes with an antibiotic he was also a doctor here. Wrote it.
Father- expenses, Madam, the expenses are just what I did not go to work, it is everyday work. The expenses then later it was thought that later we had to spend 1500 rupees, and around 3000 to 3-4000 was engaged.
Mother- It is our daily job. Earn and eat just that. Such is the situation.
Father- He said this when he admitted that B.P. of your wife is a great person
Your child can also go, mother's life is also in danger, and it was written, got it signed Were, when I did not admit, then at 5 am, the number came.
Mother- had signed up earlier. When admit took place, only at 5 o'clock when my number came. The doctor saw me inside as well, he has such a condition he is a pain, what is a pleasure he has at the same time he got the paper vaper signed. Yes, it was written that this is so B.P. Can read the tour, can never escape or cannot escape, it is your responsibility. I had read it, it was all written in it.
Mother-doctor was not a doctor at that time when the delivery started, which I did not leave the gents who were doctors. I said that I am having a lot of trouble, You look at me, I have a lot of problem my brain will explode, like this I mean that I started being like this, B.P was high due to this reason, then that doctor Meaning then, as soon as I started having pain, it started hurting. So as the pen increases more then those gents the doctor called the lady doctor from the gate, then she was delivered by delivery has gone.
Father- Now tells each child, they said turn off the AC, not turn off the AC, The bicameral AC ended on the same day. Baby delivered baby normal, baby hand I gave it, they said, feel cold, turn off the AC, they did not stop the AC. That conversation ended like that.
Mother - One's child ends up like this, much more, women by delivery is on Persia, he has been lying down, he does not have a bed, You have beds of your staff, you are keeping it empty, which is a bed for the public You have saved on Persia. Look at them, right, 3 to 3 ladies on a bed, they also take the child. Taking it like this (unhappy and disappointed).

Father- 3 ladies one bed. It was on the bed, so a lady was taking it like this, under the bed, now she was sitting with a child like this and she was feeling like seeing her child. They lie like this, I said you don't take any legs over a child with the help of it, the child is not visible is.
Mother - 3 ladies on one bed and lying on Persia, she was saying now labor When there is a child in the room, you lie down with money; if we were born a little, then we will go to private. Brother we like to bang so, there is nothing that means the patient has come, let me not say that in the government It is a lot of work, but look at the occupant from work, not the child, but the child Look. He will have an infection that is lying on Persia. Let's ladies we want to own we should stand up, but do not put the child on the ground.
Father- This is an abandoned child and throws the child into the ground. Work from Work is not Persian, there is nothing, make any mattress arrangement.
Mother - There is nothing, nothing, Ladies are sitting there like this.
Father- poor lady who does not have knowledge, she is lying down like this, I took the child you told him to stay on the side, someone's leg would be hit. I am looking at the child by looking at myself seeing.
Father - I have brought those days, when they have not given leave, are not doing it (Discharge). They are saying B.P., B.P. B.P know at home when everyone will lie down comfortably will take full sleep when B.P control comes.
Mother- I have come down on my own or else he was not discharging at all, B. P If you are not giving tablet, you are not doing anything, I am not paying attention to me then how will I be down?

5. **Could you please describe your experience about communication by the various hospital staffs during hospital stay? (Probe: who all communicated, who was the primary communicator, nurses, doctors, other staffs, frequency of communication, mode of communication, details, completeness of information, skill of communicator, consistency in communication)**

Mother- Hey, they don't have the means to talk (sarcastic smiling). Such Scolding reads with scolding ask anything, you have not even seen it. I am standing up to everyone. See, you are doing that on your own, all the people are meeting, working from business if the patient asks the right person then what is shown right. I said ma'am, check me out a little the problem is, my baby is there, not my B.P down, don't write me any medicine. Not paying attention, Dont see you done, you will not have a holiday, just like this you will not say leave, you will not take leave, work with me, check my work, What problem do I have, my baby is not with me, my BP is not down, I have someone Write down the tablet when I get it from Bahar but I am not there. Nothing but me, senior The doctor was speaking well, he had come round one day, meaning after that he was 15 again the August holiday was an affair with Because at that time, baby is around he had a time and did not see the meaning again, and there he was a sister to check my BP again and again.
Mother- He was saying that if you mean, look at the number, then you will keep coming, and while there she cleans the entry and does the entry there, she is speaking like this the crowd keeps on coming, as soon as it starts to see, what is it that is said today? Do not find what day it is, women have gone since morning. Tell me that it's not in anyone's hand is. The time will be at the same time. You are ladies you can understand Someone's problem. Means such scolding reads like scolding, as it cannot be found, it is not possible for them is everything.
Father- Madam, when delivery is done abroad, in Canada and in contracts like America. In it, their family is together, husband is with them, when baby is seen at the same time let's give it to you. I have an uncle boy who says he had children inside Canada when his daughter was delivered, she was told by the doctor, and I had a son at the same time, as soon as it happened, I gave it to my hands. Saying, look like this happens in foreign country. Here it is straight out.
Father- Madam, when I admit them there, not on the sign given there if she is not a nurse who is a change of duty, then I am telling you the client went in, in the labor room, so you had to take some paper at that time, so I said what if something, about 20-25 people were standing there, when I spoke to him or not, what all people I said, bring the paper. Just like I told him silently, he quickly ran inside.

Mother-child is over and she was asking for money from sister said if the child If we had continued to share with everyone, now that our child has become like this, what will we give to anyone you will be ashamed while taking it the child is finished, the dead body is kept inside, and you are asking they felt that the towels are lying in, meaning the delivered child is safe.
Father- before the Admit, not before the Admit this is what I said, I said something Happen, you mean something, don't you then it is quiet. Madam who agrees If a little person gives money, he gets taken care of in a government hospital, The poor, who do not have knowledge, throw it away in such a way, because in the corner, Not everyone sees the same, who pays the money is taken care of, this is inside the system.

6. **Could you please tell us about the declaration of the event/outcome (stillbirth)? (Probe: who declared the outcome, cause explained, the body language and expression, preceding events before delivery, meeting with any senior doctor/member, attitude/body language/expression/skill of the communicator, expression of empathy/sympathy, language and simplicity, perceived completeness of information)**

Mother- She did not deliver at that time she showed that she signed your baby.
Father- No, he had told that the child at 11 o'clock did not agree. My sister went They were not letting me go inside, then they had called them, saw my sister in law You, the card was made, then went in, told Bhabi that blood is not there, baby is not there, baby is there is no movement, the child is finished. Yes (nurse said to bhabi).
Mother- I was speaking with love in the same way that I am not crying, it means that Done, who knows that he was the one who cleaned me, gave me a bit of comfort. And that the child did not tie the child, he said to me, do not cry beta, the doctor has done nothing said, he said, look at what has happened to your baby and is in such a condition.

7. **What was your and your family member's reaction when you were told about the stillbirth? (Probe: what was the reaction of mother, father, other family members who were present, did someone try to console you or your family member)**

Mother - did not allow to meet, do not let us go to the labor room, and the timing of the gents at 4 o'clock In the evening, there is no time in the morning to meet the gents, in the evening, they send the gents at 4 o'clock can meet only at 4 o'clock. So late in seeing his own patient, he gets arrested let's check it out.
Father- She was crying and telling me, what is it, after 11 o'clock I lay down for the night Was awake, so I went and sat in the waiting room and slept for 5 minutes, in that case A bhabi says that the child is not No matter what the child is saying, you give blood Come, blood will rise.

Mother - no help, I told my husband, the time when I entered, then I had to be pained, I did not see anyone, at the time of admission, so I said that my Call me husband, call me, no one looks at me, I have been suffering for so long, say Gents is not allowed. Inside the Labor Room, where the entry is from the gate, the Gord gave them did not even come in were not letting anyone come.
Father- Say gents are not allowed, so I took my Bhabi along, she is also not allowed.
Mother- I was in my shock, I mean it happened to me like this and what has happened to me even though the whole time paid treatment was nothing like this happened? I am not in my own senses only.
Mother- When so much time is said, what happens in the beginning is a different thing, and a person does not feel sad if there is such a thing after months.
Mother- I feel that my child is finished and no one else go. In my own mind, the meaning kept revolving I had so many in the hospital did not raise the baby to mean something likes that. Our baby clothes were new when I took them she did not even wear it, so I told one of the ladies that her children were not twins Were, so that means they were lying naked like this, ac was going on, so I said my sister to, I said ask this ladies, if it takes clothes, I will give it, Children are bare, they will take I said that again but to tell her our baby has become like this, tomorrow someone Lets talk, try it in my mind, if you tell me, it is not good, that ladies means she brought clothes at the same time, and then I started crying, I said, tell me sometimes

Whenever a child is finished, a person becomes so much, whose child When it is over, it is not that my child is not there, my own two sons are so big Have been Look, show it, I said people like this can think, thinking means their such Means thinking, no one can say anything.

8. **How was your experience about the procedure after death till handover of the body to you? (Probe: processes asked to follow, documentation and papers given, interaction and behaviour of the staffs, respect for religious norms, time taken, cost/payment, assistance from the staffs)**

Father- gave in my hand, yes, nurse handed over. No, they are from me Earlier, we had asked for towels, then one cloth is blue in the hospital, and Later, he said that you have brought some cloth, you have brought towels, I said that I had brought the child So, I had given a towel, they had tied the child in the towel.

Father- At first I was seeing the same in S Hospital, I told a driver how many people, he thought that he is not a man by knowing, I was born in Delhi, one in Delhi I do one corner every job, so I said brother, how are you taking money, said brother Of ambulance, I said, I am a driver myself, saying so much money, I said, I am a driver myself, saying It will take up to 1100 rupees. I do not think so much. Where am i going now if I made the phone at home, my friend also, then the mother also said bring it home.

9. Who all from your family and community/neighbourhood were present at the hospital and how they supported you during this period? (Probe: who informed them, who all came, what type support given)

Mother- No, she was Bhabhi, she is here Father- all were born, no (asked anyone other than bhabi), that mother was at home, mother was looking at children. Mother-child is not it. Father- No No (asked for neighbors, friends), just three

9. **What all rituals and procedures did you follow after stillbirth? (Probe: body preparation, who prepared burial / cremation, other rituals and timings, who performed them according to the rituals)**

Father- I did not open the towels here, brought home, brought here, then my mother said, Open your mouth and then show it, then the mother did not bring it here, nor did the mother bring a little mouth again Washed, buried. The entire locality had gone to bury.

Mother - We had gone to the entire locality, gone to Surrey.

Father- If you found out, there were all the fires in the locality, not all the neighbors were ours all coming. Just they gave me at 5 o'clock, I came, and hour took me till here, 4- by 7.30, I was buried. Method, because Madame was a small child, then let her stand in it, no The Pandit was not called, our Pandit was one, he had taken them together, so he had said Thing is, when that hospital was drunk, blood was given, then fruity was found, and biscuits I had it in my pocket, it was a biscuit, and I buried it in it. There was no such, they said, I asked, not saying no, there is a small child, just like that buried, the rest went to Panditji as well.

Father- No, the mother had spilled milk that day inside a bowl in a clay pot Poured and had taken out, was feed.

Mother- had fed the food the mother had fed the children one day, in her name.

Father- No, no man had gone there, no woman had gone.

Father-mummy has washed her face when sent from home.

10. **How did you, your spouse (husband/wife) and immediate family members cope with the difficult phase? (Probe: any change in the psychological state, physical state, engagement in daily work, spiritual/religious activities)**

Mother- What could emerge, I still remember.

Mother- means thinking it means that it keeps on rotating in the dim, means that I have not already I would have survived when I had not gone.

Mother - Sorrow is always there, but it keeps on thinking.

Father- Look madam mind, look madam mind, but it is our job now turning the thing on the road, we have to pay attention. Had to read about taking the life of others that is the work of a driver, he has to read

the road and take care of it. At work if you don't go then don't go home. It is a daily base job. The vehicle is everything, and that is how the mind turns. Then the man has to read his heart only at work.

Mother- Oh my time, I still cry. Everyone scolds me at home like this if you stay how you will live, will you have two children, who will see it I still have a lot of mind I feel so sad I sit and cry many times. 7 days, I did not leave my room. I am Used to cry like this while sitting inside. Meaning I used to remember the same thing again and again, something like this done, today like this never thought that something like this will happen to me. Give of There is no problem in the child, there is a problem in this child. I think this to me No problem, despite getting complete treatment, this is happening to me.

11. **Is/was there any change in the interpersonal relationships between you and your spouse and other family members after the event? (Probe: relation between husband and wife; relation and attachment with other children, is present; relationship with other family member who are physically present; contact with members who are physically not present but contacted over phone)**

Father- Secondly, Madam, no one says no to drink, I lack any drink does not contain fruit, dry fruit everything, milk shingles, there is no shortage of anything. Someone say there is lack of food there is no shortage of that thing either.

Father- I am not the only one to beat me, Madam, I do not leave anything short for food.

12. **How did the family members support you and your spouse (husband/wife) during the difficult phase? (Probe: burial rituals, community norms/rituals, coping with the phase, financial)**

Mother- No, my mother was here, yes. And aunt-in-law had come, she had also come.

Father- Mother was the rest of the neighborhood, and my aunt had come, below my aunt's house is the same.

Mother- They were saying that why did you go to that hospital, everyone means anyone who knows did not think, he said that why S Hospital went. While here I live in front of me that is their pharmacy job of Apollo, if they come to the hospital to give medicine, etc. He also said that you would go to Malviya Nagar for a time, when I said he kept me If not what should i do so saying why did you go to S Hospital. Such wonderful care of Bhabi does not happen. Saying that we know that we take medicines, we also meet with the doctor. There is a lot of trouble there I said I did not know that I was such a first time have gone to S Hospital, that something like this will happen. I did not expect that we are saying that big the hospital is.

Mother- My mother had been living for 15 days, now my mother-in-law has left. Now my mother in law she had gone here, she lives nearby. Mom, I stayed with me for 15 days. Yes He asked (she helped in death rituals), yes it was here.

Father- Then took the ladies to a doctor then they gave a quote Family planning, she started hurting family planning, she did not suit at all. Go that too go to the doctor for a separate opening. She does not suit anyone saying. They are saying the swelling is going on right now blood was coming to a halt. So he went and gave medicine Ladies Doctor, below.

13. **What were the reactions/ counselling and suggestions given by the relatives/neighbours/community after the episode? (Probe: what to do after death, following the rituals, knowing the cause of death, any other)**

Father- Aas neighbor madam this is what people come for 5 - 10 minutes then after that sit who comes for why is it so difficult for people to fill the ego, ego at home? The child has died, it has happened, it has become a thread, it has happened.

Mother- People were saying that its children, the child has deteriorated even many hands did not allow his child to be installed. Its child is over, our child is not finished Go, meaning we think of such a thing. Neighbors say that it was not yours, Gone and then say why did you go to S Hospital, then you say you did not do it Will have done something in eclipse.

Mother- Many people were saying, the body was hot there was no blue, there was nothing, the time the body has been delivered, it was shown to the child, I have about half an hour it has been until I have

started talking. The body was hot, there was no blue pan too, there was no hard water in the child, and everyone was saying many people by the doctor Said that if the child was full in the stomach then the delivery would not be normal, there would be no normal delivery and I said now I do not know whether it is normal or not my first time baby In the family, no one in the family has such a chance that this happened to me, and to me don't know about this that happens. And while it looks like it was such a hot body, it seemed that the child would read this song right now.

14. **Do you think and/or discuss with your spouse/ family member(s) about the loss and related events and blame someone/something or feel guilty for the same? (Probe: with whom; how frequently; the context of discussion; contents of discussion; whom do you/others blame for the death or what about you feel guilty)**

Father- silent

Mother- I feel so much, I do not know if I have made any mistake, I mean something like this happened. That day a lady came from the dispensary, she also said that it was not my duty, he should tell the ladies. I got so angry at him too what I should say whose thing is farming, one feels sad. Do not understand what someone said.

Father- silent

15. **When did father and mother (if working) returned to work after the event? (Probe: father, mother, loss of job/ resignation from job/change of job, was there any change in the mode of functioning)**

Father- I have gone to work after two days, I have gone after two to three days.

Mother-not gone (asked if he went to work for those 7 days when wife was in hospital after delivery)

Father- Yes, he did not go. Yes after discharge 2-3 days more he did not go to work). Taken on 14, then on that day then another two days off It had happened, then one day after that, the vaccines were swollen, the swelling was there, red the pus It seemed that the antibiotic was written, it was taken again.

Mother - I was taken to hospital in that

16. **How your and your family's life has changed after the event? (Probe: what has changed, what is the major change, what do you care more now than before; plans for future pregnancy, any substance abuse- new behaviour or increase in the existing behaviour)**

Father- Every single child was finished in some Gurgaon, so I did not leave Bhabi so I was entering inside S Hospital by taking the car, so do not see the car or taxi seeing it did not come on my side, then brother said, August 15th was the day, no vehicles were found Was on the 15th of August, then he started saying, brother, you have to leave the Gurgaon, I said to leave Gurgava, and he was saying how many people, I said 500 rupees, then I sat, When she sat, she started crying, now she started crying, I said what happened, saying brother Sir, my child was born, the child was finished, he was at home, brought here, S Hospital is saying right and safe, and inside it was glass, Emergency does not know ICU I kept giving the child in the next half yen hour, saying, The child is finished. What was his own grief, I looked at his grief and started thinking, he was a mess, he was so damned Those who do not foil, do what they can get in Dehri, who get 4-500 rupees, inside gurugaon, there Purani Gudiya Mata went to leave there, so seeing that 500, did I reverse, I said brother Hold 100 rupees you work, I said you keep it back, I give it 100 rupees back and work Got it done I said keep brother, my mind got upset like that. I thought there would be someone else. Patient will let me take all the money, seeing that my mind got upset. To many such prisoners I leave you free. No one is sitting in a car I leave free like that, humanity there is something called a name. The children are over in those days.

17. **What was the cause of stillbirth mentioned to you and what is your perception about the value of knowing the cause? (Probe: knowledge about the cause of stillbirth, willingness to know the detailed cause, potential factors that led to the illness/death, potential impact on the other family members or next pregnancy)**

Mother-no didn't tell anything.

Father- He said that he was your child this week.

Mother- They did not ask, they did not ask, they were fighting with me, they would have asked once. What was the meaning of the problem I have given full time, they have not said anything, they are saying now what will I get back, start fighting with me, now what will I try back? Boy story sir Nobody will do anything. The thing we knew was gone they kept telling me like this I said that you speak so much at home you speak to them like this. I speak to the doctor was meant to be my baby wandering I feel it was not the first child I don't understand, I had a third child, I understand, how, it means everything at once It happened, sometimes they are going to get ultrasound, sometimes they are taking it there, lying down, Doctors are talking about themselves, no one is there, meaning they did not have anything. Means night, Within 2-3 hours, my whole case got worse within 2-3 hours.

Mother- He did not ask, he did not ask anything.

Father- Saying, the child saying in the last was complete, then later saying your child
Was weak, his heartbeat was done.

Mother- I also have a sticker which binds the child, no I have not (about getting information on cause of death), I did not get that much.

Father-point turns around brother, when you understand it means (overlap)

19. In your view, what more could have been done to know the exact cause of stillbirth and the reasons causing it? (Probe: investigation, autopsy, any other)

Father- (initially silent) No, not like this.

Mother- silent

20. Are you aware of any method for identifying the exact cause stillbirth? (Probe: autopsy, investigations, etc.)

Father- yes (probed-aware of post mortem)

Mother-silent

21. What are your views about the autopsy/ post-mortem for identifying the cause of stillbirth? (Probe: necessity, religious aspect, time needed, disfigurement, etc.)

Mother- Yes, if we had someone, if we had it in our mind, we could have it done Was it, I would have known what the reason was. Incision is right, but the cause of the disease would not have been known, either from this problem or from our mistake, or from them. Happened by mistake

Father- Well, Madam Post Mortem should be done, but at that time no mind. Was ours at that time.How should be the death of the prisoner. This also says rule. Anyone becomes an accident, due to which reason he becomes an accident, also of post mortem can not happen without His dead body does not give him government without post mortem. Baby on I am listening to this post of Mortem's puzzle this time. I did not know this thing there was no knowledge.

22. What are your views about collection of the tissue (like done for biopsy to diagnose diseases) and body fluid samples for identifying the cause of stillbirth? (Probe: necessity, religious aspect, time needed, disfigurement, etc.)

Father- MITS. When blood samples are detected by taking out, when the meat If the piece is being detected, then what is needed to tear it apar no one in religion is not denied.

23. How parents and family members can be approached for the tissue and fluid sampling? (Probe: who should approach, whom, when and how should approach; what should be told and in what detail; should some picture of the method be used to explain)

Father- If someone knows about the knowledge, then take the decision quickly, who will have knowledge, if he tells us, and he will definitely take a decision. Now came rotavirus, in rotavirus It was starting that child for one year means you start treatment, one year, then he had said rotavirus, meaning the first child did not have the time of older son rotavirus, and my younger son had rotavirus on time, so he had to take full care of the child fever feverish, he used to check and used to do everything, also gave medicines, meaning his teeth It turned out that he did not get latrine inverted vulture, the tooth also came out easily, he Vaccine looked different, it looked like vaccine.Now they gave information about that rotavirus, we must have seen the doctors, the senior doctors were good doctors. So that too NGO the NGO was run by

the rotavirus, so the dispensary was there, so they were also targeted had rotavirus with so many children. He started fighting saying that one, I said brother, Medicine, and children like him also used to suffer, admit the child till Batra Hospital He got the treatment done he used to see all the treatment.
Father- There is no such thing that I can take a decision, even with my wife (decision), now we had the first father, the father was over, now he is the responsibility, now I am the elder the decision will read me only.

24. In your view, what factors are important for acceptance of the tissue sampling by the parents and family members? (Probe: desire to know the cause, trust, confidence on the health care provider)

Father- Then Madam would take the report, then it would be complete, the trick would be, then the man would get it done Neither, then, will take the full report. Of course, until that report comes, if these reports continue to come in, then whatever crime is happening in the hospital, Crime will stop. The client will have the same effect, not the client in front of us. Could ask, why it happened. So what doctor will do in his own way, uncare Will not do. If you are afraid of this thing, then the time ahead will also be right.
Father- Then Madam would take the report, then it would be complete, the trick would be, then the man would get it done Neither, then, will take the full report. Of course, until that report comes, if these reports continue to come in, then whatever crime is happening in the hospital, Crime will stop. The client will have the same effect, not the client in front of us. Could ask, why it happened. So what doctor will do in his own way, uncare Will not do. If you are afraid of this thing, then the time ahead will also be right.

25. In routine day to day operation who takes decision about the major activities (purchases, treatment, spending money) about the family? (Probe: involvement of the mother/women in the family, any other family member, consultation with any other member)

Father- Madam, look at the business that is out of business, I only see, whatever you have to bring the goods you have to do something she brings my wife on her own, even to the clothes the children who want to do marketing have to do it on their own. Yes wife does purchase and spending money). Have to go with the rest I would go to the grocery Yes, because Reliance is not fresh here, Grossree then I pay with the card, like, so grocery got up from there and gave the grocery lock.

27. After the delivery, who decided about the burial and other post-death activities and rituals? (Probe: father, mother, any other family member; did you/your spouse talk to someone in your family like elders or relatives to take critical decisions)

Father- Mother, Mother said, bring home, Mother said, bring it will have to do this.
Mother- Yes, earlier he was saying the same.

28. Now, we are completing the interaction/ interview. Would you like to add anything?

Father- reformer Madam in Father's Hospital, I want to say that all the area wise our Hospital, whether it is after small dispensary, area wise after dispensary Shift in the hospital, to be a big senior doctor inside those hospitals at night Should be there, and there should be machines, which do not have to run away after picking up the patient from there. Because if there are no machines, then the patient will be finished. To shift It is over, the patient ends up shifting somewhere. After stopping at one place, after stopping, then after stopping at one place, then the same thing happened, In the old times, this police station does not come in our area, you write the news there, then There, he would say that he comes in that area, go there, what would have happened in that Again. Treatment of Bandhan was not done on time.
Father- Now suppose that I am a driver, I am taking you somewhere, my car 5th stepney is puncture, and I did not notice and the patient is in my car, a stepney and If it is punctured, what is the fault in it? It is said that the driver has the highest responsibility. The driver is the biggest wayfarer, the driver who does not want to be a guide in real How are you And if the same is not true, then how will the train go ahead. That is Madam Government Whatever your NGO is, tell those who are near the hospital there medicine, not this Go get the thing, the thing that requires gloves, everything you need, the operation Should be in the theater, machines should be yours. Who took the patient here and there does not run. May the next one's life be saved Life is one time.

In-depth Interviews of parents who had Stillbirth- 09

**1.1**	**Primary respondent (relation with the child)**	Mother
**1.2**	**Age of the primary respondent**	20-25 years
**1.3**	**Other members present**	Grandmother

Events that led to the stillbirth

2.1Can you please let us know about the problems, illness and events immediately before delivery?

Was hurting then went (After completing 4 months) Then we don't know we were unconscious (No one was with you) No we were. As the pain in the night was a word now the night was like water, it was full now the road was too much, I brought it once and showed it to the Dai It is fine and then in the morning I went to S Hospital again with the morning I had gone here before Where did MN first take the from here again what did the doctor tell the doctor Then wrote to S Hospital Then he went there and took pain in the night. We did not know we thought, what kind of pain is there Now what was the day like in a compliment how was it not According to this, we thought that we would never lie Is So I thought that maybe this kind of pain is going on How come Yes, I saw the child, that it was over 4 months. But according to the date and according to them, they were saying that there are still a couple of months left so According to that we thought that there was similar pain, how it hurt we understand this Could not be found due to this again, came here from the morning it had happened there was no fever there was no fever no such illness (I am talking about when I was pregnant) I came a couple of times (You showed it to the doctor then) Here at the dispensary I had brought the medicine Do not keep it as it was shown (You did not do anything for 8 months ultrasound etc) (while shaking her head) No (also in some dispensary etc.) came from the village early

It was not even ther We said, I will get it done here I have not gone again I came here for a month or two Like I came from the village I said that we will get it done now think for two months, I said, I will get it done Then the same pain started in the evening on the 21st at nigh and the rest of the child was all right I had gone to see the babysitter in the village where she came She also went to see in the evening.

(In which month) were shown on six months and even when there was pain, she came to see here They have children, they come This is what they are doing, they know that because of this, they came That's right, the boy has no problem He also said that he should take it there.

2.2Where did you go or whom did you consult for any problem during the last pregnancy?

Here in Malviya Nagar, it was written that there Now about MN our landlord had to go to the place to go and take it there and my aunt used to live there and tell me to go there Then I said that I took him there There had become serious He said that it is more serious will not be able to be here You will have to take S Hospital There I am probably 7-9 o'clock 9 o'clock in the day yes in the morning we left here at 9 o'clock must have arrived at around eight o'clock S Hospital may have reached at eight o'clock (How many hours will be spent in MN). There may be half an hour yes we stayed half an hour Who after reaching S Hospital I saw them first) First of all I went here with them there are no good guards there so guard inside Entry has been ventured and everything is done. No longer there was mum etc then again. I saw how they did it. They said that they told me that mother I said what has happened to the mother they said that their child is torn and they will have to be operated. Did not come then said what you say I said ok, now they have to save it, now they have to do operation to save it. He then took them again and went there then took us there, where there is blood and blood then he started the operation here.

This was the only thing that happened to us and the rest is right there. Everything was fine there was no such thing There is any problem or anyone there was no such thing Where in S Hospital, the treatment was well treated everything is done our thing then everything is complete it is to be spoken again then we have come paper, etc, everything made, paper etc still keep the paper, etc. of S Hospital there .

Hospitalisation period

3. Now we would request you to tell us something about the period of hospitalization for pregnancy, delivery
Or termination?

I probably stayed there for five days or six days I stayed five days On the sixth day, there was an evacuation from all of us only then were you told that the child was no more (Then tell Did not give the child no)

4. Could you please describe your overall experience about the hospital care?

Nothing was felt there No, no, no They had said to give blood and then some had said some blood, they had said to take and then they had offered the whole thing on their own yes a little too much blood was taken When I had gone to the hospital with them I was told then then I had gone there

5. Could you please describe your experience about communication by the various hospital staffs during

hospital stay?

The nurse told ... the doctor had told me .... hmmm ... yes the nurse told me ... who do not see it there ... takes it to the patient ... now it I do not know who are the doctors there but who is like that… is not… it is a little different…

So who saw them, they said to me that there is such a thing ... It was told to me that there is a baby rash that is

Bursting. Do you know the reconnaissance… Yes, when their operation was done… .. We have left since 11 o'clock… Operation was done…

The operation was done when I brought the child from me, the doctor… the nurse was probably there… to tell you that your child is very well… and had already told…. Do not do anything to the child and whatever will happen after three years, you will do it… Do not do anything before that…. Who has given me a child… He told me…

Stiilbirth

6. Could you please tell us about the declaration of the event /outcome?

Attitude and skill of communication told me that your child is not like this ... and your patient is fine

It is right, is right ... and do not do anything further from some three years before ... was told like this ... & lt; language and simplicity yes, I understand ... .. yes ... okay Thak told .... & lt; sympathy & gt; No no, I did not say in anger .. I have told you in the same way .... (Treatment) Yes, it was okay over there ...

7. What was your and your family member's reaction when you were told about the stillbirth?

My sister, when she came from there, is fine ... Operation vaporisation is happening right now .... When I came from there, it is being said that the operation is going on for a while. Say ok… just no one else… medicine kept happening… we stayed there for 5-9 days then…. .Boss was explaining in front .... just like ... (Mother crying) .....

Mother - Everyone used to speak lovingly… hmmmm…

8. How was your experience about the procedure after death till handover of the body to you?

He had given it to his mother .. where he was a nurse and so on….

Boy ... boy has happened .... I said let's be fine .... then I saw him ... after seeing him, he was taken away again ... just like he used to bring

It is not for anyone to put it in and then bring it to me… He has signed it there, no one has done it.

Were not

9. Who all from your family and community / neighbourhood were present at the hospital and how they

supported you during this period?

When my body was found, it was my behen only there… I am not here… the child again… with us who… our, their… Behen was there, was Behen's man. .. gave it to the child again in his hand ... Now I thought that I will leave from here .... You have to bring something or do anything, who will bring it ... So I was there again .. . I say as it is .. here everything is done, it is not…. So I said go and give it away… .. It is a cremation ambush here and not here, but a little bit far away, we were taken there… it is here only in Sangam Vihar…. After doing all these things, then they came… Yes, they had brought the whole thing from there… When my sister came with the child .. Then again… When she brought the child…. My maternal aunt has gone here and there… she lived there, she lived there… she came to wife… she lived… we lived…

Post event procedures and coping mechanism

10. What all rituals and procedures did you follow after stillbirth?

Yes, we have done like this… Now, we were on the same place… but those people have come here… they had brought them… (no pandit or pooja recitation). ... Now those people had brought them now ... Now we were on the same place ... Now we would have made everything done .... Now we would have stayed here only for two days, because of this we are there But because of this ... so I said, take the child to pay tax ...

So was brought again…. And before that we used to live in the villages. Toh was there in the countryside as well as a patient… Toh had happened there too .. He was taken before that and also there It had become a girl ... she has also done it from the operation like this ... Now she had got it done in other operites .. The second child was). Yes .... It is like that ...

11. How did you, your spouse(husband / wife) and immediate family members cope with the difficult phase?

N/A

12. Is/ was there any change in the interpersonal relationship between you and your spouse and other family members after the event?

N/A

13. How did family members support you and your spouse(husband/wife) during the difficult phase?

N/A

14. What were the reactions / counselling and suggestions given by the relatives/neighbours/community after the episode?

Yes .. Our landlords are asking for money from them, so they also gave money..and where they kept the money wherever they were, these people gave… (suggestion) .. to come here. S Hospital, to be taken for here .... Malviya Nagar …… No, no one would tell such things… All of them used to say that they should be checked… they should be checked, but we have noticed Did not phir, then get it checked again, so make it a little late ...

15. Do you think and /or discuss with your spouse/ family member about the loss and related events and blame someone/something or feel guilty for the same?

Whom do you / others blame for the death (Father) No, whom do I blame? No one is to blame .... (Mother) No ... (Father) Yes, yes, no one has any fault .... No, sir .... We are shocked that what to tell now .. The same toh is now the look of the first girl, it was not even there ... Now it also came, it has also happened ... Now toh we are the only two ... And after that, look at the operation too They have become one or two, now there is no chance for the future… To be seen, now we are the only two… See…

16. When did father and mother (if working) returned to work after the event?

No, we do not find any problem in making work .... We go to work ... go at 4 o'clock .... There is no problem in working copy .... (Fathor) almost ... 7 months… 4 months I was in the village… And after being a middle girl first, if I went to work after a month… No, no… Now take the water from here and there Then he gave it to him later ... (Mother) after 10 - 15 days ... No at this time after 10 - 15 days ... (when it happened) It happened in October ... Yes in October…

Went to 22 way in October, but yes, in the last month .... It was operated on 22 way… Yes… Since the operation, it has not done any work yet. .. (You started doing only after 7-9 days)

Mother - Hmmm ... I mean at this time, now we do .... but that is not so much ..... Father - We do not allow to do more work in the evening ... nor to lift a heavy bucket .No make a flour… don't let us do anything like that… right now we do not let them do it… .then we are both here… yes there was trouble… but we again Have taken it from ... but then gave it to him later, did the work in the evening and then gave it to him ... There was a lot of trouble ... There was a lot of trouble ...

17. How your and your family's life has changed after the event?

N/A

Knowing the cause of stillbirth in detail – autopsy and MITS

18. What was the cause of stillbirth mentioned to you and what is your perception about the value of knowing the cause?

No no..then I haven't ... yes why is that goes....

19. In your view, what more could have been done to know the exact cause of stillbirth and the reasons causing it?

N/A

20. Are you aware of any method for identifying the exact cause stillbirth?

No, we do not know such… No (Post Mortem) No…

21. What are your views about the autopsy/post mortem for identifying the cause of stillbirth?

No, now, we do not have a gift here… Yes, yes… Patients… No… and there is no reason… Now we do not do this here… whatever happens now There is no one now .. He has no one, no matter how he does, he is his wish, we cannot do this instrument ourselves….

22. What are your views about collection of the tissue and body fluid samples for identifying the cause of stillbirth?

Yes, on seeing him, how do you check what he does not have to do… He would have to do it… (Mother) We were unconscious… not as if the child was over How are you done ..

So for him, like our brothers are not there in the house, there is no one to tell him how what happened and what did not happen .. Then he would have told me if he had to say it… or I would have been, if it seemed right then Get it done .....

23. How parents and family members can be approached for the tissue and fluid sampling?

Now as the videos are there, it contains it, it is clear that it is clear how it is happening, how it is not happening… it also looks… it looks fine .. So yes, it is right, it is right… .. It is known from the right… Now, by telling like this, now it seems in the heart that how are the daughters telling you…

How are you not telling me ... it appears. This is the right thing… it gives… how is it happening? Even in the heart it seems that how the sons are telling you, how they are not telling you, it looks right ...

24. In your view, what factors are important for acceptance of the tissue sampling by the parents and family members?

Decision making dynamics

25. In routine day to day operation who takes decision about the major activities about the family?

No, I do the whole thing here, whatever happens …… to see… bring… everything, I do everything by myself… yes She buys a little, now lets not go out too much. We live in the house… we have to bring whatever is to be brought… now it comes from their choice, whatever they say, they also bring it with themselves, there is no such thing.

26. During the hospital stay who discussed with the doctors / nurses to make decision about the treatment and other procedures?

No, it was mine here, because when it was said that they will have to be operated, to save them .... So I did not see anything to ask the housemates whom to ask ... So my heart I came to understand that it is to be saved, so I do not have to ask anyone…

Now it is the will of the top who will do it… whatever will be fine…. So we said that just to save them, you have to do the operation… You said OK… Signed, said sign, then I sign Do… I did not ask anyone I did it myself…

27. After the delivery, who decided about the burial and other post- death activities and rituals?

No, I only said that the child has to be taken and taken… and you can do it the way you are, because we cannot leave them, there is no one here, as soon as we can, I will wait for them, I stayed there… So they brought everything here at home… It felt right there for all the days we stayed there in the hospital…

Doctor people too, He used to come to see on time, he also used to check .. Time, time was fine, there was no problem. .... yes, he used to let me get ....

In-depth Interviews of parents who had Stillbirth– 10

**1.1**	**Primary respondent (relation with the child)**	Mother
**1.2**	**Age of the primary respondent**	22 years
**1.3**	**Other member present**	Grandmother

1. **Events that led to the stillbirth**

2.1 Can you please let us know about the problems, illness and events immediately before delivery?

When child is in abdomen I get lot of problem. Lot of vomitting..I cannot eat food. I have dizziness. So I need to have full treatment for nine months during every pregnancy….In January, She was nine months old when she was dead. In. January It It was six months…She died in Abdomen only. I got treatment in every pregnancy… I had got it done earlier in MN ..this year in S Hospital ..... No, I get it done in full. Complete check up. I don't give up the treatment….Right now I don't want it.

My body is already weak ... Hmmmm .... maybe ... I don't want to do it anyway. No one has yet complained ... Well, before marriage there used to be pain when during monthly cycle. There was no problem like that .. Now as the blood comes more, whenever the MC was there, neither the blood came or not… And there was no problem…

Then at the end. Then one day in the night, suddenly, when the sixth month was going on, I had very bad health in the night…. Bleeding started…. So sudden bleeding… I laid in the house at night. …..... As soon as I laid down the water from water bag started coming out. All my clothes got wet. Then bleeding started. Then bleeding started …so much that I changed clothes at around 10 to 11 o clock. Itook auto from here and went to hospital. They were not listening to me. After whole night they admitted me in morning. Then I took admission in the morning… which room is it… in the labor room… I took admission in it… the whole night has passed then at 6-7 a.m I went into labour room

2.2 Where did you go or whom did you consult for any problem during the last pregnancy?

Yes, it was bleeding again, so I went to MN, to show it ....So they were not listening fast. I have having too much of bleeding, too much bleeding. All clothes had become dirty. They were not listening to us. So all ladies were saying there that you are facing so much trouble and are not listening to us. Why are all these doctors sitting here, only to see the patient they are sitting here. Someone was there with us, so they had called big officer. So they said that now we will see them. Then also they didn't saw us. They only said that get the ultrasound done. Niether they did my ultrasound nor any medicine for stopping blood. So people who do ultrasoud I told that person.

So I told the brother who does not sleep, brother Will ... If the stent does not cure it, then how will the stool be closed..Then they put a needle in me, they injected ... They put a needle to rest, I .... Then I said, everyone started saying Do not show here, go to S Hospital, there will be good treatment for you… then I came here to S Hospital…

Then I was admitted here, I stared at it for two days, kept my admissions for two days, then the treatment was cured… then it was cured… Then I came home…. It was a month… after that I started getting treatment …… I was just undergoing treatment with S Hospital… So I said when I am having so much trouble. I will have to get my treatment

I get the treatment done in the hospital anyway, so I started getting treatment ..... Ever since when I used to call you, you have to come in so many days, week day, ten days, month and day Have to get the test done, it has to be tested… .which I used to do everything… but still I used to get bleeding between me once in a while…. Used to go… So I was bleeding once or twice in the middle… The fourth, fifth month was going on… Then I started bleeding… for two days…

Even then I used to go to show it too .. That test west again, do this much of the world, do it .... Still I used to do it ... Still my bleeding did not stop. Yesterday I moved from MN to S Hospital It was showing from the very beginning… Yes, it was showing in S Hospital itself from the beginning… First it was

shown in the first child in MN and then in the second child it was shown in MN…. I said that nothing is happening to me from Malaviya Nagar, then took S Hospital ……
So went to S Hospital, toh this last, this girl I started showing from S Hospital, from the beginning I… yes… left MN and went to S Hospital from the beginning…. No, no, no Seen in the way .... not doing anything ... nor should I put a needle ... Those people are not doing anything ... then I said, I will not listen even here, so I started speaking .. here is no hearing You are not going to go to S Hospital, there you will be treated from the beginning .....
Yes bleeding started, so I had gone there, MN, I said that I am bleeding, I am having so much trouble… .. So he started scolding, yes it will not happen, you guys pretend like this ... one happens, you bring it here by four ... We have come to harass us, we started scolding like this. .. I was not looking at you, I started going home again ... I started crying ... Toh there were 2-3 ladies, so what did you say, I said that I am having such problems. ....
Told that the problem is getting so big… You show me… I have no numbness here… So then they started speaking to the ladies, okay I will call the senior officer. They will call them Will check it out… So they called me…
So, the officer said, "OK, you go. I have spoken to the doctor, she will see you ... Then I went again toh again made toh paper and said go come ultrasound done ... Let me go to get ultrasound done today." You will not have an ultrasound tomorrow… then tomorrow I am done… tomorrow I am an ultrasound, in the line lying in the morning… at 12… 11 - 12 o'clock my ultrasound happened… after that .. . Then I went to show her the report to the doctor .. Look at this…
He saw it, did not even see it in such a manner ... and threw it away like this ... Speak go, you come to bother .... Go. It will be alright to sit at home .. It said .. .. but resting will cure itself a little… how will it be when the problem is not treated… .they did not say anything…. Then I told the brother whom I had done the ultrasound. Bid brother, don't look at me, I also have an ultrasound and bleeding
They did not give any medicine to them and drove me away, saying that go home, sit and go… what is going to happen to him …… Come on, okay, we inject needles, we have needles… ...
I got the needle injected ... Toh then I came to my house ... After coming home, the toh was reduced, it was not closed on the bleeding .... So I said it will continue to fall, toh How will it happen again ..... the child may be spoiled, the fall will not stop, so it will happen ... I will have to show it, nothing will happen by sitting like this ... then I To show that S Hospital has come, then I got treatment from here, for the last six months, all the delivery was done, it was here only .. Then I came home.
Hmmm ... went to the beginning .... then started showing it from S Hospital ... yes I had gone the same day .. came to ultrasound to get it done, and then I asked for a needle They kept me going… ..the second one, so yes, the second one was going on ……. Then I started showing it constantly. Then when I called, I used to go. ..... No, I did not go there ... I started showing in S Hospital .... I was admitted for 14 days .... Our goat does not come Eid festival, goat festival, it was in August not...
So I was admitted there a day before the festival… Yes… It was 14 days… On the day of admission, I stayed there… .. I said to rest… .. Rest here. Rest the bed. So I used to rest at the same place, I used to rest …… So there, I just used to give medicine to eat a couple of pills, get an ultrasound of the deli, just this, that is a needle A two-handed antibiotic, which needle was used ... Once he put needle and gave it a couple of pills ...
And always used to come to ask how much trouble bleed has reduced today, it was all that they asked .... and just used to give medicines ...
It was written out of medicine outside, we used to pay for our money… and used to give two medicines from inside… .. and there was just ultrasound, nothing else… and took blood From man, two hurry, two quick will be needed at the time of delivery, then from where we will give .... Yes it bleed in the second month, then it was also in my midst ... Admit was done I was admitted in the month too, then I used to go in the middle… and then my last month when it was gone, then I was admitted… It was done for 14 days… Then 14 days was taken for

Hospitalisation period

2. **Now we would request you to tell us something about the period of hospitalization for pregnancy, delivery or termination?**

Then on the same place, I was kept there for a couple of days… even after keeping there, the bleeding did not decrease…..Bleeding was going there. Then they shifted me to labour room. then as I was better they sent me to ward no. 5 and 4….. I stayed in the same place for 14 days ... Even then I did not get any treatment there.... Means nothing to stop bleeding .. Bleeding kept on going… Whatever was happening was happening.

No medicine to stop bleeding. Medicine was being given the same way. They were giving medicine. And the ultrasound in the same manner. They were taking me for ultrasound again and again. They were not giving any medicine to stop it, no injection, no medicine, nothing .... Then they said that we have to operate. I have to do the operation, we have to throw the uterus out. I was scared for 18 days…..And I was not well. I was in a bad situation there ....

Then, in the last, I started having more bleeding ... Then doctor said that it has been embroiled in bed so they said what is this. Then I siad Mam lot has happened to us. And I am resting here since long. I am not being given leave neither you are giving any treatment. My child is small, he is also getting irritated here and there without mother. Please give me leave. So they were not giving me leave. Niether they were letting us in.

Then I, when I started getting more bleeding, my mother said, "Let's take you to delivery room." Either you will have operation or it will be normal delivery because your child will not survive. It is 6 months old. And already there is so much bleeding. So they took us to delivery room. So she gave us two injections for the pain. So that I can bear the pain.

If not, then the operation will be done ... Then they gave me injection. Then second injection then after that I gave birth to a baby girl in the morning. She was dead in my abdomen. Then I came back at home in morning. And they took blood from my husband that you will be given blood. It will be needed. They did not transfuse the blood. Neither A bottle of water. I feel so dizzy.

3. **Could you please describe your overall experience about the hospital care?**

There was nothing special, someone, I was not treated according to the way in which I was having trouble, I feel that I was not given treatment accordingly. I was not treated there, in the way I had my problem. They just kept on admitting me…they used to come and go for 18 days.

And I was always , they used to see patients accordingly, They used to see me accordingly. It was not like that more to thers and less to others. It was not like that. They used to see the same way as they used to see others. And no much of treatment was given there…..the way it was done there it did not happen there. I told them that if you are not able to do it then please give me leave.

Instead of staying here I should go and rest there. I have to rest here too. I have to rest at home. And I have a small kid he is wandering here and there without mother and father. My husband is also here just because of me. They don't allow gents to go inside. They used to stay outside. I had to spend a lot. A lot. I stayed for 14 days, There was lot of spending. Whatever was said, that work was done. What should I say. Because my husband did all the spending. I was in hospital. They never used to allow us meet. I came at 4 o clock in evening. For one and a half hour. Then they went away. Then whole day from outside. I used to stay there whole night.

Now whatever is there….We have to get medicine from outside. Give her to eat this that , Juice and all. This has to be given to eat. The expense for going in and out.

Then my relatives had come then they took blood from my husband. That you should give blood then we will give it to her. So blood was nt given. Blood was with them. I still have weakness. I was saved from fall. There was so much weakness.

4. **Could you please describe your experience about communication by the various hospital staffs during hospital stay?**

Yes, they used to ask how much blood has fallen today… how much has fallen yesterday… show me the brother… all this… you used to ask only… I would not say that this medicine for your infection Will go ... to get this medicine, and you would keep eating it in the morning and evening .... I used to tell you to eat and write. And they used to explain ... that there is such food ... we will meet medicines outside here

.... I will meet you here ... I used to tell you all this .... Yes it used to say that the operation has to be done Your child will not survive, so let us tell you in advance…
Because then later do not say that ... My child is not saved because there is no chance of your child being saved .. So I said, Madam, the child will not be saved, I am here for so many days .... Then what is the use I am staying here ... when I am admitted here leaving my home door ... I have been lying here for so long leaving the child ... When my child will not survive, what should I do to stay here profit...
Better give me a break, I will go to my house .... but I will come to my house ... When I call, I will come ... or someone give me such injection or medicine So that my bleeding can stop .. so my child can also escape… I used to say rest here, when… if it will be okay then you will leave then go away…. It will be fine when the needle is not injected, not the medicine ...
No, we do not have such a needle, there is no medicine .... Just relax, it will be alright, so it is fine, otherwise the child will have to be done with the operation. Mam used to know. Doctor sir .. all ma'am used to speak ... who used to come to see little ma'am. Doctor saheb .. then big ma'am used to come ...... after a day or two ...
Or even used to come to the deli… always used to come and go… later she used to see everyone again. We used to see people from one side too… Madam, the elder one used to say the same thing, the younger one. Madam too .... Toam elder mother used to speak to them ... That too she used to speak with her own understanding ... My child has my nostalgia and not ... but resides there. I had left it with that one… .. My faint heart is bigger than me… I had left it with him….
And I was admitted on the same side .. there was no toe ladies with me… because everyone lives in their own family… .the child is in his own work… .who sees him nowadays with me No one had gone, just used to come to see, but there was no stopping with me… .I had called my Behen from the village… Then she came from the village later when I was about to leave. Mary ... one or two days ago - two or four days ago .....

Stiilbirth

5. Could you please tell us about the declaration of the event /outcome?

Showed me at the same time .... Doctor was there, was in another room ... next door ... in .. I was very upset with the pain, with pain ... then I was back there .. delivery. She went to my place .. Doctor sir was not there at that time… There were all the doctors in the next room… .. Then I was in pain, I screamed… I shouted that Doctor sir is going to make sense to me. My baby is coming out ...

So he all came running again .. Madam everyone all ... Then he came Toh he delivered again ... Then he cut the navel, he showed it again .. Look at what the girl is, boy. So I said girl He is there .. Then I saw ... Then those people took the child to another room and cleaned them cleanly, then they have been with me since then. I told that the girl who was born to you has not finished ... told me ..
Then my husband comes to my behen ... announces to him that I have been delivered ... finished baby .....

6. What was your and your family member’s reaction when you were told about the stillbirth?

Just don't ask, ma'am, I don't even know it ...One who suffers he only knows it. Apt from that no one can come to know about it. It happened with me twice not once. My husband and my big sister were there.they never used to allow us to go indside. Whn we have delivery they don’t allow us to go inside. We have to stay there outside. So whenever they want to ask anything, so call us. Then they ask that is anyone admitted here…What kind of problem, sign and all. They don’t allow us to go inside. They do not le us in.

7. How was your experience about the procedure after death till handover of the body to you?

After that, I was in the labor room…. they gave it to my husband, gave it at about 12–1 am… she was not giving… that there was a document made by the doctor that they had to get signed. And I don’t know she was saying something. Get it signed then only I will give baby to you. So in us we give some mud to children. We bury the child whether it is big child or a small child we bury it. So when they called us to give away the child so they told that give the child to us.

So, mam was not giving away the child. She was asking for some sign….She was saying someting… I don't remember. Then we insisted a lot that mam please give us, my sister was also insisting that "mam please give us, we have to bury all our family members are here, please give us. After lot of insistance they gave us. They let us make some paper. My husband and my sister were running. Then after they got the sign done they gave the child to us at around 12-1 p.m….my husband brought him home "My babu" ... and I was in the labor room itself ... I came from the labor room, I also came in the labor room at 1-2, He came to the ward… from the labor room when he was sent to the ward.
I came to the ward around 1–2 o'clock…. After coming to the ward, then my sister was with me again… Then on the same day on the 4th night….I came to my house at 7-8 o'clock.....I was discharged on the same day…….It happened at 4 o'clock in the morning my girl was born.

8. **Who all from your family and community / neighbourhood were present at the hospital and how they supported you during this period?**

No ... My sister also came later ... I was delivered on the 4th, so she came on the 4th ... Just came three to four days ago .... My sister was not with me. She was alone there .. Yes she came four days ago, then she was with me… It was with her… Then when she took me to the delivery room, into the labor room… don't let her come in… Ladies Do not even come to…. Yes it was at that time… but when the delivery was taken to the room, there was no time. That… at that time he had told her that you cannot enter the delivery room…
Did not allow anyone to come… .. Yes… I came later… When I announce that I have not been delivered… I have a child… .. Then I have my mother at 10-11 Sister came to see me..When I was taking a bed, I was delivering delivery .... So she came to give me tea and biscuits. My sister ... at 10-11.
No, nothing happened, just my sister just said that she had brought tea biscuits, le yellow .... So to me, she was crying, in tension .. So I told me not to drink tea, Leja Toh Boli Doctor sir, it is necessary to drink, yellow ... I started speaking my sister a lot..Then I drank a little bit of biscuits, then my sister-in-law, nurse was not there, nor the doctor started scolding all the time here. Don't wait ....
So my sister left… did not let her stop for five minutes, just …… nothing… no one did not allow me to talk… did not even give a chance… I started crying At the same time. Just after drinking tea, I started crying. At the same time, I started crying ……. They just did not let them talk… get out, get out quickly… with help The ones are… not to talk much…. Give me tea, just say shut up, just shut up and it will be five minutes.
My neighbor and my sister-in-law had also gone, my sister-in-law, nor the elder one who was standing here, had gone here with me…. Which I had gone on the first day or when bleeding was happening… 14 days which Admit was the first day that I went, when the paper was made, my toh was my sister, along with it, when I took the admit in the labor room, in the morning my sister came home again at night. I ... Since then I was alone again in the hospital .... My husband was down outside and I was in the hospital ... The rest of my neighbors or my relatives did not stay with me even for a day… just used to come to see, used to go ..

Post event procedures and coping mechanism

9. **What all rituals and procedures did you follow after stillbirth?**

We bury in Matti in our house, yes we bring a bus to the hospital, whether the child is big or small, whatever it is… we are given a mati… burial… no from the hospital They are brought to the house and then we are bathed in it.
Yes, they do the same, be it big or small, they do the same to everyone ..... No, I was not in the hospital, nor did I allow me to come ... No ... My husband had come home. Yes, my mother-in-law was bathing .. My Nand was bathing .... My husband had told me, I had brought me to the hospital… .. My husband took leave, took the sign sign and brought the child ..... Because we have to take a shower, we have to be buried, that's why I had brought it… I did not…
I was not in a separate ward, neither was I in the labor room, nor did I .. I would not allow my husband to meet me, nor would I ever meet when there were orders to meet…. Did not even know when he came up, my sister, Kish time was called… When did you come…

I did not even know so much .. because we do not let you meet .. We have a separate room .... We live in the same place .. Do not talk to a relative, nothing happens .. Whether you are hungry Stay, or thirsty, or suffer in any way, or live well, or live poorly. You don't get to talk to anyone, nothing happens when there is a need, when it is announced and called Goes yes your relative, behen, daughter ..
Whoever it is… they are hungry, thirsty, give them water, or give them food… then they come… they also give them to the nurse…. Those nurses come and do not allow to meet .. Whenever there is a need, they will talk at least two times, they are not allowed to meet outside ... that much I did not know, my husband, how they gave the child, how not Gave..they were just telling my behen that they were not giving it soon. They had given it to do so…
It is bathed in us, spreading the dhimkana… after requesting a lot, they gave up… .then my husband came to the house… so I was in the hospital… I told toh night I came to the house… they were in the morning, my husband brought me here, Nehla, went to Dhula again for burial… Then when my work was done here, my husband went again in the evening. Brought me to the night ……

10. How did you, your spouse (husband / wife) and immediate family members cope with the difficult phase?

That is only known by a mother. What a mother goes through. He has child. She keeps in abdomen for 9-6 months or so. So if a child dies after that then a mother can only feel the pain. A mother can only know what the pain is like. A mother can only say. I cannot say. I cannot say anything.
Whatever is being experienced by me. We are still. When I remember those things….How much work you would do I still remember that thing. Hmmmm….they also cried….They came at home. There is lot of trouble. Hmmmm…..lot of weakness…there is always vertigo…..I don’t feel like working….It feels like….

11. Is/ was there any change in the interpersonal relationship between you and your spouse and other family members after the event?

No…
Hmmm… not much, it is the same… they have this habit… they are just, they are just men… He has this habit…..No, they don’t take care like that. He has this habit only, what can we do….yes, they talk a lot. Yes, I talk like anything. I talk with anger. Then I talk nicely. I explain them a lot. They listen but then again the same thing.
The first the habit is like that only. He has this previous habit. I say that why do you do like this with me. My child is no more alive. My body is weak if I would get pregannt again then again I am going to suffer. Then again I will have to suffer. Because it should not stay right now. Right now my body is weak. So he does not listen. He has this habit which is wrong. Rest everything is good. Only this habit is not good.

They won’t see this. Rest will bring everything to eat… to feed… to feed, to mean… to take care… to do everything….If there is pain in head then they would press it. They understand everything but do not understand this. If he has to do this then he has to do this. .... they have to do it. Yes, when I came from the hospital, no. I have come back after 2 months ... I came in August… yess 2 months have been completed.
He had left it for few days… Did not get 10 days and 12 days… When I used to cry that I have such problems… I came from the hospital….after so much pain from hospital….My child is also not there with me…..Now you would do like this in weakness with me like this……I have to bear all this……What will I have to go through then he did not do it for 10-12 days. Then After that they don’t agree.

12. How did family members support you and your spouse(husband/wife) during the difficult phase?

Yes, my mother-in-law had come……Then she left…..where I had my house is in the south, she went back again……she has a goat at home, goat and all, so therefore did not stay here for long……My mother-in-law did not stay here for long. She stayed here for two or three days then went away. I did not get any help from my mother-in-law. My sister was there with me. When I came from hospital she was here. She stayed here for one month. She used to do all work. All things were done by her. I never used to do anything. I used to sit like that. Nobody gave single penny. I took loan…..I was not able to pay back

loan….These who helps others. These days people ere so tensed. They have their own kids. So expensive it is.

13. **What were the reactions / counselling and suggestions given by the relatives/neighbours/ community after the episode?**

Yes everyone says that yes you are weak, you do not conceive for a few years yet .. Because of weakness this can happen ... So you do not give birth to child right now .. Do not even think about the child. ..Everyone said to me, so much .... I used to live there .. Everyone there said this and all of my friends said to me, don't tell me now ...All of my friends said to me.
This has been said to me. So this time, now this has happened with you. This has happened with you twice. This happened with me before also, this time also it happened. So do not try to conceive this time. When your body is alright then you try to conceive. Then my body broke a lot .. There is no strength in my body because even before it happened, after nine months it got over ...
Hence we are thinking we do not want to do anything right now.

14. **Do you think and /or discuss with your spouse/ family member about the loss and related events and blame someone/something or feel guilty for the same?**
15.

Yes, we talk that now we do not have to do so, what is the solution to do this… I am in a hurry on this…… We are looking for remedies. Yes, I have a sister-in-law, she keeps talking to me... Someone says to take medicine, someone says,to get copper T inserted ... Someone says something, I don't understand what to do ... What should I say it to anyone…when god has written this in my destiny.
I think the this thing that has happened to me should not happen with anyone else. If what has happened, has happened ... it should neither happen with me, nor with anyone, I think like that only...... Now from now on, neither be with me nor with anyone else...... I think like that, god should not do it with anyone.
It is going the same way…What changes will be there….The way it was before it is going same way……It is just that there is lot of tension….I am thinking about that only……What should we do for that I am not able to understand.This problem again and again….My health goes down again…because this problem happens again and gain repeatedly… there is weakness that's why ... there is always a lot of tension in my mind……mind is always saying something…but there is nothing in this world….There us always tension in my mind but I think what to do…..Nothing happens thinking anything wrong in my mind about it….something, we will die, we will think that this happens to us..we, why did god made us like this .. why with us, Like this, I will die or I will do something, what is going to happen with that… Nothing is going to happen… Now what can we do… When the god has written like this in our destiny… We are struggling with our own selves……what to do…We just think that this should not happen with us…..

16. **When did father and mother (if working) returned to work after the event?**
    N/A

17. **How your and your family's life has changed after the event?**
    N/A

Knowing the cause of stillbirth in detail – autopsy and MITS

18. **What was the cause of stillbirth mentioned to you and what is your perception about the value of knowing the cause?**

No .. I have not been told any specific reason for this .... I had that too done ... What What you call, MRI and CT scan. It was of whole body. During this time only. When everything was done. But it was not known why my blood was falling. Everyone used to say…doctor used to say that the uterus is down, because your uterus is down so your child is down…that's why you have so much bleeding..It will get better on it's own…There is no treatment to it. Neither injection to it. That's why you are having so much problem. If this will stop then blood will also not fall. And your child will be fine…Particular reason was not told….We wanted to know a lot…and I feel like had I come to know about this I would be more careful…as of now I am scared to have a child….

19. **In your view, what more could have been done to know the exact cause of stillbirth and the reasons causing it?**

No .. I do not know anything like that ....Due to which reason this is done. We do not know anything like this.

20. **Are you aware of any method for identifying the exact cause stillbirth?**

No ... Yes, I have heard about it. But to this extent we also don't know what happens. Just heard that there is a procedure of post mortem, so we don't know what all are the problems and what not.

21. **What are your views about the autopsy/post mortem for identifying the cause of stillbirth?**

It should be investigated, yes, it should be done ... because it should not happen again If we get to know that why it happened we will get the treatment done in that way… we will take care of that too…..

22. **What are your views about collection of the tissue and body fluid samples for identifying the cause of stillbirth?**

I understand and I can say it is like taking your blood sample so you can get to know about it…..So may be we can get to know the cause of death through this…..but they are talking about flesh, I am scared about on listening to it…..I know about blood…..I have taken out the blood many times…..Till the time she was in hospital…my blood was taken out…..I cannot say anything about that procedure….I cannot say anything in terms of my child..I cannot say anything all alone…..Because in this my family….because the child is dead so nothing much can be done in this…..I cannot say anything on this all alone…..

23. **How parents and family members can be approached for the tissue and fluid sampling?**

Yes ....If you will tell just like this in a cool way so she will like it…..They can tell you their own way…..Because thinking of a man and a woman is different……Because they think in a different way……They think something else and they get to understand something else…..So he will tell accordingly…..Now I think that we should not tell….We should take advice of my husband also as he is elder to me…take advice of my husband….So hence I cannot tell..whatever he will like he will tell accordingly…..

24. **In your view, what factors are important for acceptance of the tissue sampling by the parents and family members?**

Decision making dynamics

25. **In routine day to day operation who takes decision about the major activities about the family?**

Because we…mostly my husband takes it….and sometimes my mother-in-law also tells and sometimes both of them together tells us. Whatever is suitable…..whatever is suitable to them and me….so we do it together…..Sometimes I refuse if I am not liking it…..sometimes I refuse and sometimes she refuse….And at times he is the one who only takes the decision…he is elder to me so so we should respect his thoughts It should not be so, it should not happen because they are older, my husband is, they should also listen to them, that's why ……

hmm ... they talk about this….. No ... my husband. They used to ask my husband whatever they want to ask….Doctor used to call so they used to come….Only my husband and my sister, they had come from village….I had called her….sister elder to me…..hmmm..What to do …what not to do…..

26. **During the hospital stay who discussed with the doctors / nurses to make decision about the treatment and other procedures?**

Hmm… They talk about it….No…My husband was there so they used to ask from them only whatever they wanted to ask. Docotr used to call so they used to come. Only my husband.

27. **After the delivery, who decided about the burial and other post- death activities and rituals?**

And one of my sister came from the village.. I had called her... She is elder to me.

In-depth Interviews of parents who had Stillbirth-11

**1.1**	**Primary respondent (relation with the child)**	Father, Mother
**1.2**	**Age of the primary respondent**	Mother-20-25 years<br>Father-25-30 years
**1.3**	**Other member present**	None

2. Please let us know the course of pregnancy and events around the delivery/ termination. (Please document as the event were told by the respondent)

When I took it with me, it was Thursday, then those people gave us a pill and sent it while I thought the treatment was wrong, according to me, in the rest of the patient they know more than me in the hospital for 4 days It has come here and it will tell what is there, what is its fault and what is not.

FATHER - Was I also (asked if he was present in S Hospital)

FATHER - Look, this is the treatment, we are fine. Look, I had a great passion for 4 days, it was inside, now they will know.

MOTHER - Oh, they must have seen right after having a child, they were told by the doctors, after that, after that, the child was gone, they had run away when they were in trouble, they should have taken them in the emergency. So, the doctor was not taking it, there was such a doctor there, no no I will not see, I will not see, go go, go outside, I will not see, she was doing like this, she was doing it in such a way. I was crying and I came I got an auto and hurried to sit down and then the pain did not get disturbed, then I went, then when I saw the child was dead, I had said, I also said, I came to see.

Father- Hey Madam, it was our day on Thursday and when we took her to the hospital at 5 o'clock, the child was exhausted.

MOTHER - We had ultrasound done. The problem had increased greatly.

FATHER - Actually, we don't even know if we are right. We also had a new address, whether the child was over or not. When those people gave us a tablet and sent them to sit there for an hour. (S Hospital)

FATHER - Yes and sent a pill to us (S Hospital) when it did not get rest after giving it, I made it ultrasound.

FATHER - We found it in a while when it was found out that, whenever we found a new one, a doctor sits, Doctor B, we ran away with it, so he confirmed us at 7:30 pm. I finished This is the evening.

MOTHER - In the evening, at this time in the morning, I was in S Hospital, those people had not taken it. Means were driving away like I go, I will not be lungi, I will not be doing it like this, doctor. My ninth month was completed.

MOTHER - I have a little heart problem.

FATHER - By the way, the first child, this is the girl I was with, was from S hospital.

MOTHER - There was no problem.

FATHER - Our case of this second child did not understand what had happened. Everything was going well at the last time, at the last time.Those doctors who were in the

MOTHER-emergency were not right, in the rest, they would have shown the doctor on the day of Mercury. They got good treatment, they all gave me medicine, they gave everything well.

MOTHER - Call S Chachi, and don't call anyone. Yes, she was with me, she was not a mother-in-law, call her because the people below were neither my mother-in-law, she was not with me. What did they know from them?

FATHER - The biggest main thing is that the day we ran for the first day, we only complained that we were negligent on that day. Yes, as it was a problem, those people who did not see the negligence of the doctors inside the emergency check, what was the matter, what is not the matter, they gave us a pill and sent us a lot of trouble.

MOTHER - I mean, the doctor was talking like this, the doctor was talking, in the emergency, he was out, I said madam look at me, meme look at me, I am suffering madam, no no no no no talking like this Was

going, no, I will not see, no I will not see I have shot, go to your house, go to your house and I am sitting crying, I said madam, look at me and I am saying madam my very trouble madam, crying And saying that there was no one with me in the Emergency, take someone inside

FATHER - madam I saw the negligence of the right in the ill, inside it, do not see the patient properly inside those people at all.

MOTHER - Dispensary here. (asked about antenatal care), hey dispensary is equal here.

FATHER - Our dispensary is in this block only. Yes Goremant is dispensary.

MOTHER - (during pregnancy) used to call there every week. Just had to show his weight and B.P check and the doctor, after seeing the doctor, he would give medicines, he was just iron calcium. Yes, ultrasound happened, this is my one. Look, I had got this done, (showing papers) came just normal, then after that, when I was having trouble, when the problem was increasing, the child came dead.

FATHER - This is an ultrasound from about 6 months ago.

MOTHER - 21 October was due, it was October or September, then ten days before that, the pain was ten, ie in the middle of ten fifteen. Everything was fine, I am not telling you the time when I had pain or I am sitting at 7, 6 o'clock, I have sat up and gone, I am a little bit pained, then the child was feeling mean. Means to move around again I called the one above, I said like this - this is my pain, saying, yes, a light flame is always there. I said good again, I am new again, I have a desired doctor here, there is a center market, she has gone in near me. In the trench, this quote is up to us, you do this, go S Hospital, then I am S Hospital After leaving S Hospital. Aunty was our aunt, she took the mother-in-law, after that, as the doctor said she looked at her mam, then she said, "I will see new relatives in the new baby Dani's mouth is new, meaning she started screaming at relatives, then later she went out" , I told you all the things, take me mam, I have a lot of pain, I am new, I will look new, I will look new, I will look new now, take a pill, I will show the pill, it is this pill Chest pain will be cured.

2.1. Can you please let us know about the problems, illness and events immediately before the delivery?

MOTHER - Anything new happened mam anything. There was nothing, I am telling everything everything was normal, Riya, everything, I had a little trouble in this, I knew anything new in this little girl.

FATHER - madam I went to work for two months in the care of these people in the affair of drinking.

MOTHER - In this girl, I did not eat anything, weakness was also very much, I ate something new in it, I have had everything in my second child, Allah I have eaten everything very well and everything went well then on N time and whatever according to me Something happened because of sadarjung, in emergency, if they used to take me there in the morning, there was an operation in it, then my baby would have finished. There was another story in the labor room with us too, so my child is dead, well, saying, your child is dead in the stomach, yes,

MOTHER - Yes, I go to the dispensary (asked where you usually go for problems in pregnancy) and there was no such problem. I used to give me iron, calcium tablets to show me that I was going every day. Any problem was new. Yes, free treatment goes here. S Hospital So I used to go every Mercury every Mercury day. Means used to say that every week we come in, I said okay, come every week I say ok.

I went, then I used to ask what is right in the child, yes the child is fine. Just used to say this to me and you are sick of heart, why did you take the child, I have taken the child because of my heart is going on because neither did they say that do not drop the child, take the child, The child will become like the first one. I used to call S Hospital on the day of Mercury. That means, they used to make this paper on paper paper, then after that, the doctor used to have the same on his B, where he used to be here (dispensary), so he should go and show it.

I have started going to S Hospital since the sixth month. In the sixth month, when I went on the day of Mercury, I came to my house and then came after it, then after 15, 22 days, Mercury means Mercury, then I went after it, it is shown in Safdarjan for two to three months. I have shown it till the last time.

Don’t say is also new (ultrasound), I had shown the same (previous ultrasound) saying, ok ultrasound, I showed. Meaning used to check. Yes, (checked by palpation), so when I used to ask mam, new new baby is fine (doctor replied).

3. Now we would request you to tell us something about the period of hospitalisation for the pregnancy, delivery or termination? (Probe:hospital, ward, duration of stay, course of illness)

Then, after coming from S Hospital, the pain meant very little newness and I also ate a pill. He refused not to take the pill, Hamai aunty, and then it did not mean that I was crying, so I said a lot of pain is having a child. This is the pain of my aunt, I said, "Come on, then come and go with these words," she went away again, yet she is sitting outside in the new hospital of the doctors, "What am I going to do?" The doctor will be silent, everyone hurts, You have such a unique pain, this is that, that is the talk of the world, yet in such a silent silence, I kept on being pained, I have made a memory of this emergency, for what have you made this emergency? Is, by the way, mam drinking plenty of tea then speak again, lie down, go inside and keep going for a long time, when new came Then I came out in the morning, try mam, try mam, doing like this, I am crying, saying then after that bid go in that room not in that room, meaning I was showing such attitude again in that room Even if I went, I would not lie down in the bay, I would lie in it for an hour, I would lie in it, I was sitting, I remained mam mam mam in such a way, I said yes, I am coming, I am coming and then I looked later, then I said, look, then wear a dress, wear it, give clothes and clothes, meaning one The talk was new in the right way, the employee who is the guard is there, so he says, take that dress, wear it in the labor room and you have to wear it, yes it is. Then moved to the labor room and then to the operation theater after two to three hours.

4. Could you please describe your overall experience about the hospital care? (Probe: investigations, treatment given, involvement/ participation in decision making, satisfaction level, quality of care, cost of care)

All I know is that she took me she was saying that she will become your normal child. , I said my stomach is new, you do not do normal with my operation, so they injected me, then they started doing their work, then the child was there. Then sent to his room. Just told me so much, when the child showed me that much, the doctor said in Operation THEATER that you know your child is dead. I said, yes, mam, I said, I know, then when the doctor was over looking, then in the viewing room, he spoke, why did the child die, I said mam, some doctors are just like that, The one who finished my child, a doctor… The doctors who were coming, the big doctors were saying, shagufta, why is your child finished, everything is fine, why is it finished in the papers? Trouble had driven me away from here from emergency. So who (doctor asked), I said that these doctors had driven away, I know it is new and yes, when the stitches went to get cut, neither was the doctor standing alone, thin thin, he drove away both times. Something new, she was feeling sorry (doctor), the child was alright and at the time the meaning was over what happened, I said why did this happen, so I told you what happened, whoever came, the big doctors changed. They used to ask for revenge.

FATHER - New expenses - Anything new.

MOTHER - Ultrason, this is what we did out of the spring.

MOTHER - If it happens, it will cost from outside.

FATHER - It's not like we showed it on Wednesday at S Hospital in OPD, so whatever happened on that. So whatever checks you wrote on that, we made them private. I did all the private checkups and I am sure we also got the benefit because after seeing those reports, again they took it by S Hospital people. After 4 o'clock it was a Thursday day, that day I was absolutely mad.

I almost feel that the child has finished at five o'clock but I confirmed that the correct one was found in the middle of the 7:30, 8 o'clock. I spoke to her on my phone after she left the clinic, and she saw the report that the employees she was with, If their computer is wired, then after looking at the report, they had told that the child is over and then talking to the doctor, the doctor told me that the child is over. I am a good man, he told me the expenses are very high, Dr. B has spent 50 thousand rupees in this - this is such a thing and this is that

MOTHER- Emergency very fast

FATHER - So what I am, it is not a matter that if I get here, I have a brother-in-law, I called him, he brought the car here on a red light, I put it in, took him to the hospital (S Hospital). Yes, after that, what happened inside, she will know everything she will tell, I am telling you what is the matter of spring time.
FATHER– (documents) Nothing happened like this.
(treatment / delivery decision making)
FATHER - not me.
MOTHER - Something new was asked about it. What is copper copper, what was it that was asked about copper copper? We just asked and asked something new. I asked new aunt, he told you something (decision making), saying that he told me something new. Just asked me.
FATHER - I was also told in an emergency. Yes, I also told me that copper T will start and operation (informed father about operation).
MOTHER- Hey, in emergency I am telling you that you were scared more. When I asked for blood boon from them, the mam who was looking at me, then bidding is high risk, is being done like this.
FATHER - The new doctor spoke (blood donation). The new reason, he told something new, give us empty blood.
MOTHER - All I said was to give blood.
FATHER - For some reason, my brother also sang for blood; Here is the younger brother, the blood from me is what he gave to me is healthy, one or two years younger, but I feel very healthy. So my condition was deteriorating at that time, then I will be able to give new blood. He gave blood (younger brother).
(Satisfaction level)
FATHER- Madam satisfy Do you think that after taking in the hospital in the same way as if now let's go to the report after we have given a private report from here, it is the law of those people to become my doctors According to the understanding, first of all the patients who are sitting there, that is the meaning of emergency, that the patient who is in more trouble and in trouble should be seen firstly, this is the right thing? My understanding is that this is the first thing to be seen, now let's say you are a doctor,
And there are four people sitting here and who is in the most problem, then first of all, we should look at it, this is the same thing, but there is something new, it means at 4 o'clock, it was half past six when we took the car with our brother-in-law Even after that, I still took it in till 5 am in my opinion, for the operation, he did some blood blood there and got a blood blood check for the sample, whatever it is there,
MOTHER- After going inside they did all the investigations. I showed mine, done in private still they did their own investigations.
FATHER - and asked us rightly, if you were a man we are a private person, we would have thought it would be very early, it is just from our side, think that there is a government hospital, neither can say anything, nor hear anything Can, this is the question.

5. Could you please describe your experience about communication by the various hospital staffs during hospital stay? (Probe: who all communicated, who was the primary communicator, nurses, doctors, other staffs, frequency of communication, mode of communication, details, completeness of information, skill of communicator, consistency in communication)

MOTHER - Used to talk a lot (senior doctor). She also used to talk well (nurse) used to give medicines, she used to talk very well with love, I used to talk to someone who was not in emergency: she was a little new, the doctor was right.

6. Could you please tell us about the declaration of the event/outcome (stillbirth)? (Probe:who declared the outcome, cause explained, the body language and expression, preceding events before delivery, meeting with any senior doctor/member, attitude/body language/expression/skill of the communicator, expression of empathy/sympathy, language and simplicity, perceived completeness of information)

FATHER - Nobody told us in S Hospital.
Mother- New, new in subdarjung, in S Hospital, I was asking you, your child is over, I said, yes, just finished, it is finished, yes mam I just showed reports, so I was talking about your The child is finished. (Empathy / sympathy) was right. Is your baby finished? I said mam khatam hai, that means your child is

finished in this way, that means you were completely writing in a nastily way, your child is over on paper, I said it is all.

7. What was your and your family member's reaction when you were told about the stillbirth? (Probe: what was the reaction of mother, father, other family members who were present, did someone try to console you or your family member)

MOTHER- New (asked chachi saas was there after operation), then after that my mother came, from Moradabad, living from Moradabad, My Mom. She had come she had been with me for 8 days (hospital). Mommy supported me a lot.

FATHER - As long as he is inside the hospital, till this time he remains there for 4 days, his mother, his mother is our mother-in-law.

MOTHER - She has been with us. Mommy, I have got a lot more. Means to raise. It was just this, that means that these relatives pick up their patient, walk, go outside, take them, all these things our mother was doing with us.

FATHER - It is such a madam, the matter of support is such that when we get to know when our housemates know that we are in a problem, it is like this, that after half an hour, our car is gone, our little brother whose I had just given up and given blood in front of you, I was carrying a car, I was a mother, meaning my father was my brother, my mother was my village at that time, all of them had reached that time, my whole family was on my own family, my family's relatives There were at least 35 people there.

Yes, everyone had reached who he was and after taking it in, the doctor sent the sample of blood which was taken by me, I was also new, what did I know, but what father did I take? He told me in room 108 that if we want to take this sample in room 108 I got to know my father who has been around so much, he has taken me through these things, he has taken me in my father, he said, son, I have moved the support of the family, this was my father's support, meaning here He had the support of his mother, for him inside, which he gave for 7 days, we will tell you the same thing that has happened to us which has passed, the incident which will tell the same thing, lie is more new than telling the truth.

8. How was your experience about the procedure after death till handover of the body to you? (Probe: processes asked to follow, documentation and papers given, interaction and behaviour of the staffs, respect for religious norms, time taken, cost/payment, assistance from the staffs)

MOTHER- In our mother's hand. Hmm had shown me in Operation THEATER. Was wrapped means meant to be wrapped, it would be known to them that the child came out, I was inside only.

FATHER- Was in clothes, in white clothes. Yes (hospital provided). The child was finished with the child, we had brought the child upright and our father had wrapped it in a cloth and brought it here directly. It was a report of the child's death that he had come here directly with us.

9. Who all from your family and community/neighbourhood were present at the hospital and how they supported you during this period? (Probe: who informed them, who all came, what type support given)

FATHER- I was my father, my father-in-law was my father.

MOTHER- Our father was Ammi.

FATHER - My brother was a younger person, who was talking about this: He came in a car with four people back here.

10. What all rituals and procedures did you follow after stillbirth? (Probe:body preparation, who prepared, burial/ cremation, other rituals and timings, who performed them according to the rituals)

FATHER- Look at the child with him, in Mushlim, you would know that if the child is buried, then we brought the children here and brought them to the cemetery and buried them in our community, 5050 people People followed him. Yes It happens that when the child is finished after coming into the world, then it is attained, everything is done at that time, then the child is not there, well after that I had come up, I know something new (what rituals done).

MOTHER - Yes, clean the baby a little.
FATHER - Yes, it was a clean oaf, something like that, it was a clean oaf. Those who have grown up will be big.
MOTHER - It was our mother-in-law, she was also our Fufia mother-in-law. The new still white cloth is our own which is white cloth and then we went and took it to the cemetery.
(Timing)
FATHER - It was such a body that we had got till about 7-7: 30 o'clock, it took time to get it. It took at least half an hour from S HOSPITAL HOPITAL Half an hour from the car at about 9:30 AM I think we took the child and buried it at 10 o'clock.
MOTHER – women not go
FATHER - no women go to the new cemetery which is new to Orto.
MOTHER - What's new in it (at burial site).
FATHER - The tomb we dig in the new cemetery costs, we also received the receipt of the tomb of the child, and the expenditure of Rs. 600 has been incurred there. This cemetery is here. Yes Yes (Madangiri). He had given a burial receipt but he may have been lying.

11. How did you, your spouse (husband/wife) and immediate family members cope with the difficult phase? (Probe: any change in the psychological state, physical state, engagement in daily work, spiritual/religious activities)

FATHER - initially silent, see
MOTHER - Yes, it took 15-20 days (in hospital), work was with me for 4 days and then came here and came here (home).
FATHER- New new (asked if started working immidiately), at least 1 week in Gee.
MOTHER- New new (asked if started working immidiately).
FATHER - Even before that these 4 days stayed with them inside the hospital. Yes, I felt even more then 1 week, now see if I am a man who will go home at all, then this is also a matter of drinking food daily, I have brought 200 rs worth of food and I have someone to eat. It is like that if there is a very long work, you should be a man of every day. See, the child is there and the child is suffering but now if I feel even more, then my child is not meeting Lot or I am in his sorrow

12. Is/was there any change in the interpersonal relationships between you and your spouse and other family members after the event? (probe: relation between husband and wife, with other family members)

MOTHER - No, they do yes (husband support after the incident). Keep it well Yes, I hear that people in their homes fight because of me. They get all the sports from their side and my girl who is a little mean negligent by their mother's ring, it stresses that the rent is my house, if you live in a rented house, then you get the tension in a little bit. It is forbidden that Shahgufta do not take tension in such a situation, do not take tension and in more ways I have seen that in such a situation, Do this work, this new means, this house has spread all over the world, so much money has been given to the world, so I have not removed it from my hands, then quote from the laborers, put it all here and put it on the mattress Put it all on the mattress, Malwa means that you hear this meaning with me; I also hear the meaning of this, it also means some raw ears. If their mother says to teach someone about me, then they are on their side, they also have no faith in it. His brother gave blood, so why did his mother cry, I gave blood, I am very much like this, even in a little tension my girl, I also feel that due to tension, I feel that you have asked for blood at all. How did you mean to say that I have seen where I have asked a new person, my son is very good, who would come on his own if my father was coming from Kashmir in the emergency. S HOSPITAL appears in the hour, half an hour.
(relation with other family members)
MOTHER- new new completely new means that two to three months of the day I stepped in here, just eat that thing too, if you eat that thing, then it will be easy to speak So it became absolutely bad after that, it was completely closed, now a girl has a lot of trouble, I am new to someone, I am new to them.

I said why I am hearing this in such a way, it is outside, it means that it is only that you are better than it is that it is a very mean nonsense people. Whatever is in the house, its father is okay, those who were sitting are very fine, otherwise it is all nonsense. Na nande, sister-in law speaks like a nand of her house, she speaks a new one, and she also asked a new one, why did the bhabi child get over, for which it was finished. Even in this matter, I mean how the child ended up at the time when our mother-in-law was gone, our mother-in-law had gone to the village at the time of the morning when the pain was dead, I had seen that time and then after that I the village had gone. Little by little this house is a little troublesome, it is not like open life itself Just live on behalf of the mother-in-law of the suffocating chute, I want to ask her to go there, I will give it to you. I had a problem with the doctor even in that day, I went here to take medicines, so I would beat him with a pinch, and his finger told me, it is such a behavior.

13. How did the family members support you and your spouse (husband/wife) during the difficult phase? (Probe:burial rituals, community norms/rituals, coping with the phase, financial)

FATHER - See the advice in this, what is the advice in this (friends / neighbor support), we also know what to do with it and when the child has finished himself, then it is the last account that it will be buried for the child. In our religion, it is only a matter of burial, if you are burnt in people, then the child will be buried in it, this is it. Help is something new, now whatever has happened, now that there are neighbors in it, it is just that it will be seen and the two will shed tears.

15. Do you think and/or discuss with your spouse/ family member(s) about the loss and related events and blame someone/something or feel guilty for the same? (Probe: with whom; how frequently; the context of discussion; contents of discussion; whom do you/others blame for the death or what about you feel guilty)

FATHER - This is what we will blame anyone. Now ask it rightly, today our child must have finished about one and a half months. One and a half months and we could not even know that everything was going well and it was done. So how? We are under suspense. Till date, how did this happen after the end of this thing? And why could not be found. No blaming anyone.

MOTHER- No, there is nothing (not blaming anyone). No mam is anything new (feel guilty). Mother-new (asked father if feel guilty).

FATHER - In Madam, I am telling you earlier that I got new thing at work for two and a half months, in view of their goodness, sometimes they bring some juice or apple or something and feed them, that too in my first child. It was weak, although it was weak even at that time, but we had knowledge at that time, so much was known at that time, every work has its knowledge slowly, so in our second child we took full care of them.

16. When did father and mother (if working) return to work after the event? (Probe: father, mother, loss of job/ resignation from job/change of job, was there any change in the mode of functioning)

MOTHER- If I ask about household chores right then I am not curry. Just take me comfort sister, I kept mine, she does the same. All the housework. So far I have not touched any household work.

FATHER - This is not so for the last two months when she was pregnant, she is my sister-in-law. I brought it from the village. He also has a problem with Hart's (for wife). The doctors got it to sit up.

MOTHER- in S Hospital, I had asked this thing that when you show the paper about the heart, I said that you have a hole in your heart and who does the housework for you. I do mam only. You said that you can do it? All work? I said, I live on the fourth floor, come and go, I said, do not take the meaning of mam heart in my heart like this, let me have some meaning in my heart, this is how a problem is new. Is, but I feel that the problem is new in my heart. I would have a problem with that. If yes, that tension if tension is there in the mind

17. How your and your family's life has changed after the event? (Probe: what has changed, what is the major change, what do you care more now than before; plans for future pregnancy, any substance abuse- new behaviour or increase in the existing behaviour)

MOTHER - Ask me rightly, when my child was finished, I had finished thinking that I am not doing any more. Our mother explained that do not get well according to your calculations, after five years, after three years, there will be a child with a child and if there are three children from the operation, then they started to refuse that the aunt worsens its condition. Now the child I will make new, now that I am still alive, I remember very much that I am a child, but if I do now I will laugh that you will do baby? I said yes I miss her girl. In subdarjung, she says that she is such a shahgufta child, if the third child does it, then the child Dani will burst so much that she is scared, the child Dani will burst. , I said mam baby for this, the senior doctor of the heart, my treatment has gone there, now I also say that Shahgufta do not finish the baby and do the child and the child does not have any problem due to this. Will come. When I was showing them, she was only saying that good means to have a child. I said that a meal is meant to spend your food in the morning and evening, this is the only work and if such a big work is new, it is definitely that their father has his own house, we have something new in it., Today, if you want to take us out, then what do we have right now, whatever they have now, the way they talk to us, now it is to say that I will do my in-laws I have two buildings inside Madangiri, do I have my own, have given me some of my own, they took from me whatever they gave me, so what should they believe in the house when I take it out, Remove them like dogs, they climb here, they also take it, they have taken it and how have I understood my children, how to get it, how I have taken the money to the doctor, I am the only one How have I been able to make money.

18. What was the cause of stillbirth mentioned to you and what is your perception about the value of knowing the cause? (Probe: knowledge about the cause of stillbirth, willingness to know the detailed cause, potential factors that led to the illness/death, potential impact on the other family members or next pregnancy)

MOTHER - (cause mentioned) they were asking us only and they asked us new, we asked them new, they were asking us themselves. I mean why your child ended, Shahgupta, why did your child end up, who were coming to the big doctors, know the memes of the commons, why the new memes are finished.

FATHER – (value of knowing the cause) Yes, I wish everything was fulfilled. Maybe this problem means if we understand it, then we should take care of this thing further, is it so.

MOTHER - Meaning everything had gone well so it was over at the end time.

19. In your view, what more could have been done to know the exact cause of stillbirthand the reasons causing it? (Probe: investigation, autopsy, any other)

MOTHER - I, where I was telling you in the center market that she was giving a private, I had also told her why I am a child, so she said that it is possible that the heart is over I have done something in my heart, something like this is new.

FATHER – Heart problem

MOTHER - If you are having trouble breathing in the heart, if the child is getting full breath, you have some problem, I said, I have a problem, I am new, I breathe well, I am also new as if the breath swells in such a good way. Laddis says, "I mean that I breathe, I am sick of such heart whenever my breath swells". Yes, everything remains normal at once. So said, it is good, I said yes, you will know that you are sick of heart, how do you live, I said, I am perfectly fine , I panicked, I said that panic is also when I have more tension in my mind when I feel that it is just nice to talk to someone, I feel like lying in a loneliness.

FATHER - It had happened that when all this operation was done, everything was done, neither the doctors who were emergency doctors gave us the report that they were in the last and told me to bring them to the doctor of heart They had asked for advice, they were asking for advice, it was written in it. So when I was taken there to the doctor, that doctor said to me that if you want to know for what reason the child died, the child died new from the problem of the heart. This is a doctor Amit, a doctor of the heart, he told us. Said his heart died because of the problem. Tell us this thing there. This talk.

20. Are you aware of any method for identifying the exact cause stillbirth? (Probe:autopsy, investigations, etc.)

FATHER - Postmortem, we keep watching TV and so on.
MOTHER - In it, it becomes known why the child is finished. Yes, this address makes sense.
MOTHER - It says that the heart comes out, it removes it.
FATHER - It is torn apart. This is a thing.
MOTHER - Yes, the incision is torn after tearing.

21. What are your views about the autopsy/ post-mortem for identifying the cause of stillbirth? (Probe: necessity, religious aspect, time needed, disfigurement, etc.)

FATHER -See doctor we do not get it done. If you want to know this from us, we do not make it happen. We do not do this because our daughter was there for how long. So we, at first it was over, now if we had done these other things on her, we would have felt a bit more oppressed. Looks bad We feel that we have done something bad with the girl. So we might not think so. And would not have done it. Now come the question to raise him, his life, so today we lack our child, the shortage results in a lot And I sometimes cry so much. Sitting too much and I cried the whole week. Remembering my child when I didn't go to work. Because when my child came here, when I took him by my hand, and I am not like you, I am telling you from above that the whole thing is a pity for the child from inside. There is also a problem. But now I think that if I look back a little now, that thing is not right Now I have a child and a wife as well, I have to watch a bit of work every day, but if I come home from work, I have a lot of work, and when I am meditated a little I have hours and hours It is a problem. Remembering that I would have another child. Well, two children, well, that other child is so sorry, I am very sorry.

22. What are your views about collection of the tissue (like done for biopsy to diagnose diseases) and body fluid samples for identifying the cause of stillbirth? (Probe:necessity, religious aspect, time needed, disfigurement, etc.)

FATHER - Yes, Madame would have agreed to such a thing, we would have agreed for this. Yes, we would have agreed for him because in this we told our child a lot of new ways, easy way, you have agreed that for him, we would have agreed such a thing is new.

23. How parents and family members can be approached for the tissue and fluid sampling? (Probe: who should approach, whom, when and how should approach; what should be told and in what detail; should some picture of the method be used to explain)

FATHER-Advice What to do when the child is in the father, he also takes the decision. What is the need to ask a new question, even if I would have asked Sayed, I would have asked my father. Because in these things, it was clumsy, but then it is that if there is any problem in the way in which you are talking to me, then I have a problem in my mind, then according to that, I am immediately If I do, I rarely ask everyone that I have a habit You will know the easy way, which will be the easy way. Yes, any such thing is understandable from the video. The new thing is also quite a matter it becomes a matter with the mind.
MOTHER - It is something new as you are telling.
FATHER - You tell me, I have understood this.
MOTHER - Video is important, that means Video looks like after seeing your Video, we feel that, you have told.

24. In your view, what factors are important for acceptance of the tissue sampling by the parents and family members? (Probe: desire to know the cause, trust, confidence on the health care provider)

FATHER - The way, doctor, sister, what is the way you are talking to me, you are talking very well, you people are also talking to me, so I would have done it like I told you so. Would have done

25. In routine day to day operation who takes decision about the major activities (purchases, treatment, spending money) about the family?
(Probe: involvement of the mother/women in the family, any other family member, consultation with any other member)

FATHER - It is like that now the family runs on both husband and wife as well, both have to take the decision.

MOTHER - for eating and drinking
FATHER - It is such a question, if I have to answer you on this question, then the answer is that I pay more attention to my work business, I was sitting to go to work, then I stopped you and stopped. So I am more at work and whatever he earns, he gives it in the evening, he sees the whole thing as the cloth of the child.
MOTHER - They also go along with the mill, if they stay outside at home, then I have to go alone then I have to take the above one again and then bring it back
MOTHER - Decision, mam it means more, someone is new to someone who is a mean person, who is someone who talks about it, someone who is new here, ask right here, whatever happens, neither my father lives in Kashmir. I call him yes he stays with me, he talks to the doctors, there is such a lot of talk here to talk to doctors, something like this is new.
FATHER - It is like this, in fact it is Dr. Sahab that our knowledge is new.
MOTHER - Our father comes, he means everything.
FATHER - But this is me.
MOTHER - They talk to doctors and they do everything.
FATHER - Some of his fathers are aware of this thing, but I know that the way in which you are talking to me, if someone talks in any way, then I am in a clear sense of the matter, now the affair in the S Hospital hospital is there. It is very important to talk to the witch, the question is this, if we go somewhere private, then there is a matter of money then the matter is comfortable again.
MOTHER - Understand that as people are here, they would not talk about it at all, if they would have called their father or they would have talked with our father in such a way that means they talk very well. My father is more than ours, whatever comes to me, whether it is hard or the child, our father takes more than anything

27. After the delivery, who decided about the burial and other post-death activities and rituals? (Probe: father, mother, any other family member; did you/your spouse talk to someone in your family like elders or relatives to take critical decisions)

MOTHER - They were all together
FATHER – No No
MOTHER - The people of the house were also very big and all were very big.
FATHER -See, Doctor Saab, it happens like some decisions, if there are people belonging to our own family, then when I am in love with them, I will take them only in the decision, but there are some things in which we have to walk with the elders. As long as my father did all that to bury my child there. Whatever he was down there because 4050 people went to bury the child There were some guests too, our relatives were those people had lost their drink, they had to have lunch after two hours, our father had done all that, then there was something in our elders and our elders had full sport. It is new, our mother's father had a full spot of our family, we live here on the 4th floor and our housemates live below.
MOTHER - It is just a little if you want to ask right, we have to know what to do when the child is in the stomach, or what is his mother who is a mother, who is a woman of some other brains, she is old enough to tell us otherwise. I had to know from here how what is there in this, what is new, then she used to say that Shahgufta, do not eat this thing, drink this juice, our mother-in-law told us new, in the first child, B told in it and in the second told new Negligence we have moved from here to home, yes.

28. Now, we are completing the interaction/ interview. Would you like to add anything?
FATHER - What is Dr. Saab S Hospital Hospital, which I think is a government hospital, is fine.
MOTHER - He is a government hospital
FATHER - You second - the second thing is that the doctor who is in the emergency is not me, but if I give you exactly two rupees worthless doctor, then because you have to be right there, this means that emergency means that we are going there. If you have a doctor, take that thing immediately and see it, but there they talk in the same way to someone, sit a patient out for hours and two hours, now we have a

problem for you It is like, brother, which we will spend four days or we will spend five days, it only means that we are in emergency, so if we have some value, we have some more problems, only then we will go into emergency Well then OPD is for him. So take a doctor for it immediately, burst it, but find out that brother.
MOTHER - Took it no
FATHER If the patient has to sit outside for two hours, then if the problem is so much, then it is negligent according to us.
MOTHER - He was the manners of talking, new means he was talking like this.
FATHER - Now if you ask right, I used to talk to the doctor in the doctor then, "A little backward, here and there" I saw a lot of attitude inside the doctors there. Inside Emergency S Hospital Hospital. Just because we want new that what has happened to us today, be with someone else tomorrow, and how many people will be like us with whom such incidents happen, We would like it every day throughout the day. It is just that the doctor who is inside the emergency, he should stop the lacquer and finish his attitude and pay little attention to the patients, then this should not happen to anyone. If you believe that it was a Thursday day and I went to the hospital locker at 5 o'clock, if only those people would have seen this thing for a bit. So I can be a child, so I know about the child, I know about the time of 9 o'clock, the child of eight and half o'clock time is over, the child may be alive at that time in the stomach.
MOTHER - Hey how does the child come to know after the ultrasound, I know that there is a child in my stomach, I can only understand that the child is fine, here means yes, there is a cry, how can you say that eight At the time the child was over, since I have been telling you that the child was there, meaning that the child was very well and then after that the doctor who sent the bullet from S Hospital again, did not send the child after that. Till the trouble was even greater.

In-depth Interviews of parents who had Stillbirth-12

**1.1**	**Primary respondent (relation with the child)**	Father, mother
**1.2**	**Age of the primary respondent**	Father- 25-30 years<br>Mother- 20-25 years
**1.3**	**Other member present**	Aunt (Bua)

Please let us know the course of pregnancy and events around the delivery/termination.

Father- No, MN was first taken to the city, then taken there from there.
(asked if labor pain started around or after seizure attack)
Father-inverse…
Father's sister- Nothing had happened in the stomach, I was together, nothing had happened in the stomach, neither There was pain, nothing else happened, meaning the child should be,
(asked water bag burst)
Father's sister- No, nothing happened. Just these doctors said that their delivery I would not have understood how delivery would happen.
Father's sister- We were taken because of the seizures, they have read the tour they had lost their lips Neither did they leave the blood etc. Will send more, we don't know, the doctor said that they will be delivered. Malviya said in the city It means that the child is right, but the work is being done by the child. Malviya spoke in the city.
Father's sister- Yes, later he did ultrasound. Did not do in MN, he I did it only in S Hospital Referred from MN to S Hospital there.
Father -medicine gave injection and did not refer there.
Father's sister- injected two in large clusters, both in MN. Then they will He referred to S Hospital. She went to S Hospital and told the doctor that she was delivered Will, admit them. There Now we said that they have read the tour, then they said that the child's Beats are like this, work is being done, then they will have to be delivered, so we said let's go What do we know, doctors only know, what has happened and what has not happened.
Father's sister- I was not there, so I said, what is the problem with the doctors?
If some work is being done, then the doctor would give medicines etc. So said that new. Nothing will happen except medicine They have to be delivered, admitted, I said as the doctor thought fit. admitted on 3 Day (7 days corrected by father), after 7 days, for 3 days the child was alive, in the stomach, there In S Hospital itself. Then on the fourth day the baby is finished in the stomach,
Father- Fourth Fifth Day
Father's sister- On the fourth day, then he said that the child was finished in the stomach. So if If they wanted to do that, they could deliver life. So why did he, even after he died I did not understand the affair, the S Hospital people, the Doctors.
Father's sister- He had produced a child only through medicines.
Father's sister- He was not well made, once he fell 3 months, once 2 months. These So the child was complete.

2.1. Can you please let us know about the problems, illness and events immediately before the delivery?

Mother - It was not 4 months, no, 4 months were going on, in 4 months (it was) no I have seizures Had been there, had read the tour, yes after that, what is it said, the condition became very bad, after 2 days I became aware had come. After that, for the delivery of the child, he was given injected medicine at the hospital fourth Delivery was on the day, normal only.
Mother- She came in thyroid, thyroid had become me. All the tests were done. No and no shortage Did not tell. No (anemic) ... The tablet that was given by calcium was running. From the first month, The child came from the time when she was coming. But there was no problem, nothing was
Mother - Done later

Father- It was now, now no more, dengue had happened, later it was done after having a child.
Father- I don't know now, he said that due to high blood pressure, it increased slightly, Fainted.
Father- Yes (asked if seizure symptoms like twisted arms, legs, formation of froth were seen).
Father’s sister- Anything inside, there was no internal problem, at all even I knew it all.
2.2. Where did you go or whom did you consult for any problem during the last pregnancy?
Mother- I showed here at K Colony. Same here.
Mother - not where it is.
Mother - had shown it to the doctor.
Father-No is private.
Mother-Pvt.
Father- No, it's not free
Mother - Ultrasound was done in it, there were only two, one happened in the third month.
Was and a seventh, in the month in which my child was just born, then it was made 8 days I had made it earlier
Father-called after 3 days.
Father’s sister- We go like Kabar, sometimes we go, like MN reads So we leave.
Father- We goes away, when there is any problem, we study nearby.
Father's sister- The main reason is that one gets to read, there is no other reason, what will be the reason, and the pass is read.
Father - the winning children will not be in the family, so there they are in the fish. There are more levels, Our Bhabi has children all are there, no problem.
Father’s sister- fish is the best, in terms of children, fish, everything in all, no problem.
Father’s sister- He: What is not his hospital, what do you call him, near JM, anything else
He speaks, Kasturba speaks, he is a hospital he is not government. Yes, this time it's just
We have seen his condition more serious, because of the pass, he studies away there, the accumulated mosque. It is far away, this is MN, the JM is double away, yes, because of the pass we took it, and then there we have taken
There is no jam But then the doctors also used to say, take this to S Hospital, where we are writing, the doctor people Had said, this is the man thing, it will be considered more by the doctors, your mind does not move At that time, where the doctor says, submit it.
3. Now we would request you to tell us something about the period of hospitalisation for the pregnancy, delivery or termination? (Probe:hospital, ward, duration of stay, course of illness)
Father’s sister- 3-6, then it has been admit, 8 to 10 days from work to work.
4. Could you please describe your overall experience about the hospital care? (Probe:investigations, treatment given, involvement/ participation in decision making, satisfaction level, quality of care, cost of care)
Father- At first it was like that, it was not right, then when a little force was given, one of our Also knew SHO, got them done, got them talking directly, when something went right. Else first It was like this.
Father’s sister- They were not paying attention: the people, they were not paying attention: the people.
Father’s sister- Then what is their name Head, Doctor of the entire labor room, our information is out
She had come we both went to Behen Bhai direct to her. When they called there, in the labor room,
Then he said, there is a patient named S, why did he not pay attention, what is the matter,
Father- Then something went right.
Father’s sister- Then they will have a boy after 1-2 hours. Delivery was made 3 hours later.
Father- Delivery was done after 3 hours delivery was done after 3 hours.
Asked how the response of the doctors was when you contacted them through internal approach.
Father- After that, delivery was done 3 hours later. Where they were not telling when they would be
What will happen, what will not happen, by the way, 3 days the baby was finished in the stomach, so not delivering They were: people. She used to say that 3 days the baby is over, we used to tell you that the baby is over. Delivered after 3 days. Were not paying attention that anything will happen today, it will be after 2 hours, 3 hours will be later

asked it was delivery was by operation
father and father's sister- no normal.
Father- He then paid attention. Then delivered 3 hours later.
Asked about cost
Father's sister- No expense was incurred. Father- Just the medicines he had brought: There is no other cost. The medicine cost 14–14 rupees Maybe. Later, a quarter of a month was given for 75 days (Rs. 1800), there is a bill etc. Mother- Yes, who was given medicines later, 45 days of medicines were given.
Asked if any Document was signed
Father's sister- Yes, they had the paper signed, they got it done.
Father- Yes, that is it, it was probably in it, there is a problem like writing in private Used to write, if something happens to the patient or the child, then we are not responsible,
Father's sister- Yes it was just like that.
Father- try your best, like this… this is what happens, they sign it and what.
Asked participation in decision making
Father's sister- New.
Father- nothing nothing this was the biggest drawback. Now she: Let me tell you in the beginning that you are somewhere And take it away, we would have taken it somewhere else, we would have taken it in private, that was just a problem, that once After leaving, understand that you are trapped in the jail, that now you will come out at the will of the jailer. And without Can not go by will this was the biggest problem. And there was no problem. Who is there it is their wish.
Father-blood was also given, not offered.
Mother- No it was not said that there is a lack of blood, blood is needed blood is also taken but plated No.
Father's sister- had taken a bottle of blood, not even offered it, left it there, offered it Not only a bottle of blood too.
Father- Come on, it does not matter to us he did not come in our work, and in the work of some middleman Will come. He: There is no problem, as if blood did not work in our work, I had given it to someone and the poor will work for the poor, in some of his problems, but the scene was not understood.
Asked when they gave blood
Father- On the very first day, the time when I was admitted, was admitted.
Father's sister- When, having a child, was taken before delivery, at which time recruitment was the first, delivery will be there, blood will come first.
Father- and at such times no man thinks anything. And at such times no man thinks Nothing is confused, what should we do.
Father's sister- blood giving come first.
Mother- He said, I had my ultrasound done many times there, even on the day Delivery was taking place.
Father- I do not understand the problem. Initially, ultrasound was done here, at the same time someone should have taken steps, this is the problem.
Mother-to-work, I brought her to an hour ago after being laundered, she did the last bar, the last bar Then after climbing the bottle one, inject it, then half, not even half. There was a slight climb, the pain started.
Father- As it was kept admired for 4-5 days, we thought that the child is alive, will only live when Was alive until they should have tried this thing.
Father's sister's son-faculty is also nothing special
Father's sister- In this thing S Hospital, I am sure, now even if there will be no Allah with us If you do this then this delivery is not to be known as S Hospital at all of course it is S Hospital to I do not like at all
Father's sister's son- The faculty there is very bad.
Father's sister's son- But here is not known what is sin, which is what doctors understand as our goverment is a job, so it means we are everything do we not have anyone ahead of us Now.
Father's sister's- Now look at the job, it is the fund that emerges from everyone, everything happens, Those people do take jobs, do they? No, it does not go, even the government people do not do it for free, they only take money, what is the medicine for free? He is the type ..

Father’s sister’s son-service is their, service look no, serve no
Mother-lie says medicine is available for free, no medicine is available for free, all have come from money. Medicine table
Father's sister's son- There is also an extra for stitches, etc.There is the same thing, then she also studies from outside.
Father- There is medicine, which is official, the way in which people should apply the medicine, they get it from outside, As much as the medicine is available, we get it from outside, it is not official, so let's take medicine, medicine is free everywhere at this time, but there are those who buy medicine from outside.
Father’s sister’s son- If we are a patient, we are looking at things, we are concerned and we are bad
Will not feel if they are behaving in this way.
Father- It is official that if they do not do so much then everyone will sit on their head, as much as public As public, they will sit on their heads, but they should do one thing, they Should be explained that sitting, whose patient is inside, should sit with love, should explain this is a problem This is a problem, it may or may not happen, they ask us to do the operation baby Will go, as if the operation would have survived Do not save the operation then the one above is in the hands But the child will be tired, some man will decide what to do and what not to do.Did not do anything like this.
Should have been father sister son-decision. Father- I was just thinking that we will give birth to normal, in the normal life of both the children we lost, He spent time, did not admit these 4 days, would have done the operation even then. Father Sister son- put too much time, new, take some action as soon as you go, something can happen Was Father- Later, after giving force, after giving birth to the child in 3 hours.
Father Sister son- When they delivered after 3 hours, after the source was found They could have done this thing earlier also, why didn't they do it earlier.
5. Could you please describe your experience about communication by the various hospital staffs during hospital stay? (Probe: who all communicated, who was the primary communicator, nurses, doctors, other staffs, frequency of communication, mode of communication, details, completeness of information, skill of communicator, consistency in communication).
Father-marriage is very messy, as much as the nurse was, it is very messy. This is what a man Give comfort, that it is very good, in this way treatment is going on, it is such a problem Do not take it, it is not that: it is their wish, you have to meet the patient, meet the patient It used to be the same thing, that yes, they are doing like this.
Father’s sister- Hey, I had said this to many, which means there is also a cleaning on the right, She is also a garbage collector: she also becomes Prime Minister there. It is a matter of the heart to ask if it is right. This is how we roam there.
Father’s sister- It is not that which is written. Senior doctor is not there, those who are educated people: Meaning they also talk well, they also talk intelligently they do not speak that way. Now as We went to his room, do not know what the name of Kiran was or even forgot his name, very well He gave us a guide, and said in front of us that son I will not give you the comfort in cheap. Just call now and ask what is the problem with your patient, what is there. They our Asked in front of all the doctors, who is this?
Father- He: Well (senior doctors), they talk in ways, they: talk in such a way is not.
Father's sister- Then delivery was done after 3 hours.
Father- What are you saying from above, your phone is very, why are you doing this? Will do it
Mother - I did not think of them any right, who were like, I used to tell my problem like Call the nurse, etc. Let the doctor have my pain, I said that my waist is very tight If you are saying this, it happens to everyone, what happens to you, will happen in 1-2 days I finished
Mother- No, I was unconscious for 2 days in it, in the delivery room, and after 2 days the other was referred to another room, after that I do not remember. No, the doctors were good, it was right those who used to come to check were right. Whatever problem she used to say, she would solve it. If not well, what would 1-2 tell like this, half that: Even, they never did the whole thing, The doctors there used to take a lot of difficulty even for 1 minute even for 20 seconds.
Father- Yes, just look, no life is there, let's do medicine, this medicine is finished. This is what would happen there Was I also heard father's sister- and the doctors that they would have an abortion, like that

doctor among themselves While talking, I heard that in this matter, I thought, when this happens, they will live again Why not make it to the child, like a child, the child is 4 months old, by the way, he was born dead He would have created it like this
Father's sister- used to say to me, man used to ask what happened, doctor, just come The doctor used to say sit down now, your patient is right now, we are giving medicines. Used medicine, used to speak just like this.
Father- Can't tell anything, what to do, what not to do, nothing is not responding properly, just right Right said, just did not say anything special, like you are asking, ask someone, ask in private Hey, what is the problem, what is the problem, how is it told in this way, it is nothing, Yes, okay, there they have their will, they will do it.
Father- After going in there, he is: whatever he is, he is: he doesn't talk to anyone, senior you cannot meet a doctor, you cannot meet him. Nurse Wagehra will get the same then she will Tells, run from stopper, go from here, here it is.
Mother-doctors come just to round.
Father-is not good at all (behaviour), there is no way.
Father's sister son- not at all good there (behaviour), there is a Gord to speak to them too The way is not right there is no way to speak to anyone.
Father- They has kept the phone set, those people, let's be good, someone is a mischief, they are: Safety It is a good thing, yet it is very inappropriate. If you speak to Gord once, do it this way He then leaves the arm straight. Meaning that I understand myself.
Mother- If there is also a mouser there, she: Sheeke reads it, that you should be harmed you have not explained this time. One time said that I have to meet my husband even then Not introduced Shouts there are women.
Father- shouting, shouting, be corrected, not improved anywhere, she: she does her will.
6. Could you please tell us about the declaration of the event/outcome (stillbirth)? (Probe: who declared the outcome, cause explained, the body language and expression, preceding events before delivery, meeting with any senior doctor/member, attitude/body language/expression/skill of the communicator, expression of empathy/sympathy, language and simplicity, perceived completeness of information)
Father's sister - The doctors called us,
(Asked doctor or nurse who declared)
Father's sister- No, doctors only called there, your child who is, first of all from the patient. He only told us, and told him directly, which should not have been told. Run out of stomach gone.
Mother- When the child was not mine, then said that look, your child is finished, see now, nurse had said Show me to the child that your child is not alive, your child is dead, and withdrew to the spring.
(Asked the way of declaration of the event)
Mother- No was not even angry, was right, after appearing, take it in your hand see your baby finished Happened, went back outside, after that the doctor was left with me again.
Father's sister- He makes an announcement, inside the labor room, to call. Like live outside is.
Father- announce itself, as we do not live in the labor room, outside live in the ground, Announcement, there is not even another waiting room, outside parking, stay in the open. the condition of stay), he had called there only, announcing it, and going up The doctor, the nurse who was sitting, had said that he had given the child, some doctor Did not give the child.
Father- asked the doctor, the doctors were not available at all, did they not have any problem, man, nor If she used to ask him, she would not answer.
Mother- I told how it happened, because when I came to my senses the other day, what happened to me, I asked Telling them that your baby is not well, inside your stomach.
7. What was your and your family member's reaction when you were told about the stillbirth? (Probe: what was the reaction of mother, father, other family members who were present, did someone try to console you or your family member)
The father- was sitting when he told that, S Hospital was sitting, he was not going to eat food, to eat food.

Were not going
Father’s sister- Now even if there is a conversation, there is an atmosphere of sorrow, when there is such a problem. And if someone new leaves his or her loved one, whether it is a child or anyone, then the atmosphere of sorrow remains It has There is no happy atmosphere, just the same atmosphere remains, all remain silent, what all Will in it
(Asked if someone console the family members that time)
Father’s sister- Yes, they say each other as soon as they say, they do it, they understand that now
What was to happen have happened, so what is there in it?
Father- give money, let's take money, introduce me once, I said take money, introduce two patients one times.
8. How was your experience about the procedure after death till handover of the body to you? (Probe: processes asked to follow, documentation and papers given, interaction and behaviour of the staffs, respect for religious norms, time taken, cost/payment, assistance from the staffs)
The father was a sister, no, we had taken our clothes, we had taken our clothes, they: probably in white clothes had given, no, he had taken his own cloth. They did not give anything. Father’s sister- took his own cloth, took the blanket, small.
9. Who all from your family and community/neighbourhood were present at the hospital and how they supported you during this period? (Probe: who informed them, who all came, what type support given)
Father’s sister- G. (asked father’s elder and youger sister present in hospital)
Father- Below, we were quite known when we came to tell, we had gone to pick up the child, I and my Sister-in law we had a mother-in-law in the father-hospital, the wife's Father's sister
(Asked for support from family/neighbours)
Father’s sister- Yes, just say, what was meant to be done, and will give, that's what it says, what else
You have to explain it like this. Yes, they only give comfort, etc.
Father- Consoles do just that.
10. What all rituals and procedures did you follow after stillbirth? (Probe: body preparation, who prepared, burial/ cremation, other rituals and timings, who performed them according to the rituals)
Father- We buried, (asked how much time it took) about an hour to get it. Don't give It took hours, (asked where they buried) here at Khadda Colony. (Asked about documents in hospital) Yes, paper, etc. is all I have.
Mother- Yes, I have them
Father- (asked about cost) No, there was no cost at that time (in hospital). Yes it takes a little In burial ground. (asked for documents in burial ground) there is space.
Father’s sister- No.
Father - (asked who all came to burial ground) was quite well known. Will be 25-30 people, man
All were friends.
(asked for neighbors)
Father- Yes, there were plenty of them.
Father’s sister- Yes, yes.
(asked for religious norms)-
Father-No Ladies Nobody Goes
Father’s sister- No, she stays at home, the ladies do not go.
father's sister- no no don't bathe Woh: If the child does not take mother-in-law on the ground then everything Then, while doing the bathing, we would do everything, like we have namaz and so on, and she would The child was exhausted in the stomach, so we did nothing for him, just wrapped him like Clad in cloth in white and buried him in the same way.
Father- No, don't bathe.

11. How did you, your spouse (husband/wife) and immediate family members cope with the difficult phase? (Probe: any change in the psychological state, physical state, engagement in daily work, spiritual/religious activities)
Father's sister - Now the shock emerges slowly, tell me that it is a shock but at any time it happens in the mind that the child is gone, the first child was gone. Regret it happens Father is sorry. Mother was silent.
Mother- When this happens in a puzzle, someone feels sad.
Mother- I think, how does she come how can she not find what, if she does, like my husband did
Said if my child was correct while performing that operation, then he (father) remembers anytime
My child would have been with me if he had done this.
12. Is/was there any change in the interpersonal relationships between you and your spouse and other family members after the event? (Probe: relation between husband and wife; relation and attachment with other children, is present; relationship with other family member who are physically present; contact with members who are physically not present but contacted over phone)
Mother-ji does so much, they do so much from the beginning, they still take care of me, very much
No one will do it, I think everyone's husband should be like my husband does. Because me They feed with everything they do, and I still do not have any kind of wealth or anything I bloom forcibly. Doing everything well, when I was crying I remember the baby Even then, this is what they do, hide me, give me so much love. Understand (husband).
Mother- Yes, it happens on the phone, though I have not been singing yet since my health has deteriorated, Everybody lives.
13. How did the family members support you and your spouse (husband/wife) during the difficult phase? (Probe:burial rituals, community norms/rituals, coping with the phase, financial)
Mother- No, everyone's support supports me. This was also my brother and my mother too my father was also my brother was. Everyone supported.
14. What were the reactions/ counselling and suggestions given by the relatives/neighbours/ community after the episode? (Probe:what to do after death, following the rituals, knowing the cause of death, any other)
Mother- No, they are also saying the same thing. Right now she is also refusing right now. Mother-new, He had explained that he had come to visit Bohot to meet me, he lives near me, Also, relations had also come, all of them explained, what is this, why is this, the child is gone now Is there a child back, your child, be patient, what is there, what do you give, all this remains on God Because of this
15. Do you think and/or discuss with your spouse/ family member(s) about the loss and related events and blame someone/something or feel guilty for the same? (Probe: with whom; how frequently; the context of discussion; contents of discussion; whom do you/others blame for the death or what about you feel guilty)
Father- no.
Father's sister- No, nothing in this.
Mother- No, what will happen due to someone, it happened suddenly, nor did I have fever or anything Was, suddenly she used to take breakfast after having a tour.
Father's sister- This is the only thing left in my heart, that while in S Hospital, the child was carrying her As soon as he was alive, why did he not give life to a child? Just when he told me to leave Had given that this child will have a delirium, it is just a doubt in my heart that the child will be alive Why didn't they do it, why should they do it after death only this remained and nothing happened.
Father's sister- Whether you are or not, I do not know this, I am also a mother of 5 children, this My beta is sitting for 7 months, it was also due to my shock baby, in fish, keep it in the machine They, well enough, see that it is the pleasure of Allah, sitting in front of me. But if their child Accordingly the child was saved, the child was saved.
Father- was a very good child to watch, 3 Tariq was over
Father's sister- There was a 900 gram baby in the stomach, well enough, just did not understand it
S Hospital people, why did they deliver only after death, when delivery, while they gave me had told on the go, then why did they not deliver on the live. I just did not understand it. This only my mind keeps

wandering till today. The child was right, this is now our number 1 street 7 month old baby will be there, 2 months old baby, both of them will be there. But in the mind of S Hospital people Tension remains sometimes I think, only after they died, when I already told them there was delivery of the child, so why not deliver the child's bus. It stays in the mind, No more complaints. If he had gone to father-private then there would have been a chance Father's sister's son- This is what doctors can tell, doctor.
Father's sister- My knowledge is that the child as if he has read the tour, any problem of the child they did not have any problem. Just the problem was the tour, because of the tour we They were taken to the hospital, now in the same middle, they said that they will be delicately settled in my mind So the only thing left was that if the child was to have a daily delivery, then why did the doctors know Why are these children producing them? They give tour medicine Bottling, whatever you do, same thing He had no pain while treating Nothing, there was no pan, nothing was there, of course There is no cold, no fever, there was pain in the head. If we went from father-private to private, our child would also be saved.
Father's sister's- We had gone because otherwise we would have got everything done by our private, Batra, Masjidia from the Holy Family, we all used to treat ourselves from here; We had gone, we were scared, what did the private ones know, then they would send it to S Hospital or the government itself,When we see the poor condition of his patient, that is why we are not taken directly on him We are not taken, we do it in private. We take full treatment from private we do not take government Sec

16. When did father and mother (if working) returned to work after the event? (Probe: father, mother, loss of job/ resignation from job/change of job, was there any change in the mode of functioning)

Father-time was taken, at work, 7-8 days later.
Mother- I had taken one and a half months to work. My sister was younger than me was handled
Mother- Sometimes it happens (thinking about the child), I just got it done, no so used to lie down comfortably then more attention was paid towards the child. But now when When I am in work, my mind wanders.Right now, since 7 days, because my sister has gone 4 days ago (a week ago), the child was dead on 3 Tariq, on 3 October (1.5 months), still There is not so much work at home, two to go, food is not enough because I have so much pain in my back Very much, I was thinking to go, it said that the number is not going to come in S Hospital, the line reads, when I went to Emergency, now to leave from private.

17. How your and your family's life has changed after the event? (Probe: what has changed, what is the major change, what do you care more now than before; plans for future pregnancy, any substance abuse- new behaviour or increase in the existing behaviour)

Mother-like still remains in my waist. It hurts so much that if I lie down for a while So, I am on my side it is hard here and there. Mother- After that stills not so much my heart stays in my waist.
Father- The medicine is going on outside. If you go there, spend the whole day there.
Mother- No, not now, I won't (asked for plans for next pregnancy), because my miss-Carriage was back, it was 3 months, now it is done, I do not have to. Now she knows that her It is a matter, but do not make any decision about the child yet, they do not think this thing, they would say If it happens then it will work. But I think this thing. Not now, baby To think about. Not now or not for now, they are also saying for 7 months but I think I have been working for a year, there will be nothing like that, because I am very shocked, right now My baby is over and before that happened, I was missed and this is why my heart is still Is not.

18. What was the cause of stillbirth mentioned to you and what is your perception about the value of knowing the cause? (Probe: knowledge about the cause of stillbirth, willingness to know the detailed cause, potential factors that led to the illness/death, potential impact on the other family members or next pregnancy)

Father- No, he did not tell anything till the last, asked a lot, he did not talk till the last told
Father's sister- No reason not to be mentioned.
Mother- Did not tell did not tell anyone.
Father's sister- But just why the doctors did not tell me the reason, just keep this in my mind why did they not live for the child, why should they only die. When i go all those people have told that the child will have to be a Delibhari, so they can also live a child were at that time. What not to do Only Karan gets to

know his work this doctor explains that Brother, we are not doing this, Delibhari, its just what is there and what is not.
Father- nor did he say that he had seizures due to this,
19. In your view, what more could have been done to know the exact cause of stillbirthand the reasons causing it? (Probe: investigation, autopsy, any other)
Father- No, only the doctors can tell this, such a thing had only happened unknowingly.
Father's sister- That: So the doctor can tell the reason, then why did we do delibri, tell the doctor can we tell what we can tell.
20. Are you aware of any method for identifying the exact cause stillbirth? (Probe: autopsy, investigations, etc.)
Father-don't know (about post mortem)
Father's sister- Yes, we don't know (about post mortem). Yes heard.
Father- I have heard the name but the doctors have not told,
Mother- No, I do not know.
Father's sister's son-post mortem consists of a test, etc., with an experiment on the body.
21. What are your views about the autopsy/ post-mortem for identifying the cause of stillbirth? (Probe: necessity, religious aspect, time needed, disfigurement, etc.)
Father- No post mortem is there, but whatever is there, I will tell them when you do this thing.
Father- If they are telling you because of region, we would have got it done. No child is forbidden, child It was small but Reason would have known there would be no further problem, she could have been fine if Reason shows, let's know that there will be post mortem, because of this we are doing this address, Look at what is causing the problem, children. Because he was the third child, he became It was finished, in this way, it was a complete child.
Father- If one leg was slipped, then there was a problem, yes, he would have said this thinking once it should be done. He did not say anything. The biggest problem That is, it does not tell anything. If you tell, think about the advice.
22. What are your views about collection of the tissue (like done for biopsy to diagnose diseases) and body fluid samples for identifying the cause of stillbirth? (Probe: necessity, religious aspect, time needed, disfigurement, etc.)
Father- I would have got it done then, if I had found the reason, we would have done anything, if we had done it, Reason shows that the child is finished by this region, you are taking samples in this way tell me what was the problem for father's sister- how many tests to sample, worldwide
Father- No, it is not what caused the child, what is the problem, if you tell this thing, then Sure do
23. How parents and family members can be approached for the tissue and fluid sampling? (Probe: who should approach, whom, when and how should approach; what should be told and in what detail; should some picture of the method be used to explain)
Father- is understandable by things, though not necessary (photo / video). I had older brothers, no he: If you said something, there was no problem. He: Even without asking me to say that this is happening, then someone there was no problem, he would accept what he said. Actually it shows that even if it is for this thing could have been alone or would have asked anyone. Do (advice).
25. In routine day to day operation who takes decision about the major activities (purchases, treatment, spending money) about the family? (Probe: involvement of the mother/women in the family, any other family member, consultation with any other member)
Father- I do only. He also brings sometimes (wife).
Mother - I will bring it sometimes, as it is not there, it is on the site therefore.
27. After the delivery, who decided about the burial and other post-death activities and rituals? (Probe: father, mother, any other family member; did you/your spouse talk to someone in your family like elders or relatives to take critical decisions)
Father-brother was the elder brother.
28. Now, we are completing the interaction/ interview. Would you like to add anything?
Father's sister son- first of all he: change the nature of there.

Father’s sister’s son- There is more government which is hospitals, but it also has faculty. Of there Look at the doctors how they talk with love, which is how well they treat.
Father’s sister’s son- better than the government, well, private is right, pay faculty there too so it is good, not only there, nature is also good, if we get it done in private Nature is also good in private, does not it rush in this way, right? He treats with great love.
Father’s sister’s son- Holy family is such a good hospital, the faculty there you see so much There is a good faculty there, go to any doctor, now my sister has been treated, to Doctor Vinod Agnihotri, what he is, he is such a good doctor, anyway, he too They are also working there. He too has something there. They give their time to patients let’s do all the things.

In-depth Interviews of parents who had Stillbirth- 13

**1.1**	**Primary respondent (relation with the child)**	Mother
**1.2**	**Age of the primary respondent**	30-35 years
**1.3**	**Other member present**	None

Events that led to the stillbirth

2.1 Can you please let us know about the problems, illness and events immediately before delivery? It was right, there was little bit of pain in pelvis. Otherwise , it was right .... (what was it from the starting?) Yes it was from the starting ... So I had gone there, they said...

treatment was done there from the beginning…. (In which month you came to know that you are pregnant)… .. By the third, third, fourth month… .. Yes Yes,we used to go from there… He also wrote for two ultrasounds from private, So I have that report about it too… I have two ultrasound report. (reports are normal) Yes everything is alright.

(Then in what month did you feel the pain) There was only a little pain in me… I used to go and show in OPD. They used to say that the child is weak… According to the pain, condition is bleak.… used to give medicine… calcium medicine and all….(Where did it get calcium medicine) It was given inside only… (in S Hospital) Yes… So she used to eat. Then some from outside, from private …… Had taken it from chemist here… and then ate from there too… By the way Fruit and all etc. .. (So your nine months were complete) yes Nine months were complete. There was no problem…No problem.

2.2 **Where did you go or whom did you consult for any problem during the last pregnancy?**
When she used to go there all check-ups were done. So as the delivery month was approaching there was a slight pain like that… I had shown it to the midwife….So midwife said that it is pain of labor. then we went there. We were admitted there. When she checked down she said everything is fine. Then we went upstairs and sent us to the labor room.

After sending it to the labor room ... Then there was no bed there. It was all like that. So the pain was increasing…increasing… They did not pay attention, Something like water. Had they given us injection or something then it would be done faster. Nothing...Pain was happening like that only. My child was also pushed down. then doctor came afterwards.

Hospitalisation period

3.Now we would request you to tell us something about the period of hospitalization for pregnancy, delivery or termination?

She was Saying that the child will not happen like that. when you were experiencing pain then you could have told me. What could I have said. There was no place to lie down. There were four-four, five five people on one bed. There was no place to lie down. Then delivery was done on the floor. I don't remeber it now, there was a girl of your age. She was very fat. she was a doctor, she was fat and there were two who are cleaners, one was two ... one was. (Not audible).…

.Three ladies were there. No, they don't allow you to go to the labor room. My husband was there….No one was there…..… and the one who went along with us. We took her from here. She was not allowed. Midwife was taken along. She was also outside..the same inside .... (did not allowed anyone to meet you) No ..No one was given……As soon as the delivery was over then also was not getting any place to lie down. At that time also like that. After delivery I was not in my senses. Then I got such a small place on bed. A little bit like this…

. Four people on a bed… Then we lied down like that….. Then I kept saying, that Shift me, Shift me… So at least after two to three hours, they shifted me. Stayed there for 5-7 days ... then got discharged ... <Any expenditure was incurred at that time> No expense was incurred ... No expenditure was incurred ... like of medicine…..

4.Could you please describe your overall experience about the hospital care?

Doctor used to come to check….If something used to happen…then the cleaner…..We used to say it to them….Doctor, it was told like that….If something used to happen….then the person who used to sit there used to say that please wait….Doctor will come in evening….He only sees..We do not see…..Doctor will cme in evening so we will show it to them……doctor will come tomorrow…
Like when he came then I was discharged ... I was given the medicine of 1500 rupees…Medicine was given…..Had to purchase it from outside….. I am still eating medicine from outside…. There is nothing like that… Well, there is no notice… The doctor will just sit like that… talk on the phone… it is just their work… They don't care much….Like at that time…..it was being done……May be my child was born had they done something at that time……
Had it been for More days in the abdomen, as it would have been a half a day, it was not dead...My child was fine….There was no problem in my child……My child was warm…..immediately……The time the delivery was done…..They made it lie down on my abdomen…..She told that your child is finished.
You knew it…..I told no It was not known to me…I was admitted down there……They were telling verything is fine…..they said no your child is not alive…..Child was fine …it is just it didn't cry……The child didn't cry after birth …that's it….

5. Could you please describe your experience about communication by the various hospital staffs during hospital stay?

...She said look at your child….Were you aware that your child was finished in abdomen…..I said no….I was not aware…..then she was screaming at me that such kind of people come here …they came after child was over in abdomen……speaking angrily.
(Who was speaking) There were doctors, The person who delivered, he was there. ….They said that the child was finished …were you aware..… I said, no, I was not aware….I just came after getting admitted there….They were telling it right…..So she said no your child is over. The child was absolutely right, but did not cry. He was fine, skin was hot….

Stiilbirth

6. Could you please tell us about the declaration of the event /outcome?

As the child was born…. after delivering and he was kept on my stomach….so they said Look your child….Do you know that your child got finished in abdomen…..I said no…..I was not aware… then she was screaming at me that such people come here .... The child was finished in abdomen…… Speaking angrily .... (Who Was speaking) He was a doctor, who got the delivery done, he was then given holiday…..Child wa given to them after one hour……
No, the nurse told me the one who comes there on the counter…It was told there (The nurse had called your husband) Yes (then you spoke to your husband) No, I was not able to talk…..... I came when they took the child for last funeral……

7.What was your and your family member's reaction when you were told about the stillbirth

It is right .. there is not too many members in my family.   . there are three brothers, his father ... two of our elder brothers and his father…..There is no sister-in-law. Mother-in-law is not there…..Only father-in-law…..Yes we talk on phone…..(So you get full support from them) ...ohh yes…It was like….You have got your child spoiled….Then you feel like that you have three sons I will put my mind on them…
(Or as before, you were paying attention earlier - You started feeling from inside that my child has been spoiled. No it feels like they all are gone but these all remain fine.

8.How was your experience about the procedure after death till handover of the body to you?

(How the body of child was given to you?) I don't know it was given to his father… I do not know this… I was inside… (You did not talked to him afterwards) .. when there was delivery then only it showed up after that I did not see.
Nothing ... Saying, in clothes, saying, that it was wrapped in paper, They brought him wrapped in paper that take it. take your child for funeral .... (Brother did not say anything, some sign was done) I did not asked this thing .... (Brother-in-law had met you) No ... Yes, they met afterwards when they had done his funeral.   I did not talk about it; They saw and went to their own houses on their own….

9. Who all from your family and community / neighbourhood were present at the hospital and how they supported you during this period?
His father was there. Then our relatives had come. It was our brother-in-law. They were all together. When he was taken there so what happened that there was baby's poop all over. So how could you know so when he was born then there was breath in that child. When his breath had come out. He was born well. I didn't see that. They only told who had taken her. His father was there along and my relatives were along.
Post event procedures and coping mechanism
10. What all rituals and procedures did you follow after stillbirth?
(Where did you buried the child?) CC ……
(So what happens in you people, when a child gets spoiled, So giving bath and all) Yes, we complete it, as we do it for other human beings , same way it was done or him. It was bathed. After giving bath the way it is cremated in us. Cloth was brought. So After giving bathe and giving them clothes.
The way it is done for all human beings, same way it was done for him. (Qur'an Sharif was read) No, it is said that if it gets finished in the abdomen. So it not read in that. If everything is right, so, It is read…It is buried like that in this and they come. No females don't go. A man goes. (neighbour and all had come, they went at the time of burial) No, they were there, his father. My sisters relatives they were there. There were 4-5 people there, in masjid. Hafiz ji were there. Nabiji were there. And one two more people.
11. How did you, your spouse(husband / wife) and immediate family members cope with the difficult phase?
(How could you emerge from difficult times) Have not emerged from it yet….It is the same everytime. It is like that everytime that I don't know what happened, what not happened. Had they seen may be my child would have been fine. .If emergency, then more trouble, Had they checked then you never know. Had they said that it is a operation so we could have got the operation done. Child would have been fine.
Now there is so much trouble, Now so grown up children, I am thinking… it will happen, there are two girls, had two boys been done. Then also there is no child then there will be tension. Till now I have trouble.
12. Is/ was there any change in the interpersonal relationship between you and your spouse and other family members after the event?
(Sister ji, is there still the same relationship between you and your husband) Change is that when delivery took place, then not after a couple of months, not with me…..Because I was weak. Now it is like second month (The way he used to care for you, still there is same relationship between you and your husband) But it is like, if they see anyone so he says it was your fault that the child got over. God knows what happened inside that you didn't see….
May be the child was exchanged. It is said like that. If they see anyone, if my daughter cries seeing anyone so they would say that It was all fine, your mother has done this. Now I said what I did, now that happened inside …if you had paid attention, had you seen it properly , may be it will be all fine, may be it was not done. He says who knows may be they have changed the child, who knows.
13. How did family members support you and your spouse(husband/wife) during the difficult phase?
(You got any support from your family) No, relatives .. We have two sisters, they are small. I was left alone… They were not being let in. The time you are here, she was stopped here. (What you talked about to your sisters) They did not talk anything. They were asking that was the boy fine. What happened, what not happened. I was crying, they went late night.
(was any of the elders there with you) No, there was no one among elders. Mummy came later, one or two days later, I live in the village, so came from village after one or two days.... No came from home then came home from hospital. It was like that my children were alone. So she supported, food and all of children. Next day she was not there with me. So she also came after staying there fr a night. Then I used to stay alone.

had come within three four or five days. I was left alone. His father also came at night. For One or two days I didn't get the bed. I was lying down there. Then my mother went so my mother told that her health is bad, she is weak .. the floor was flat ... no mattress ... was lying on bedsheet in the verandah. Then she came. There was a lot of trouble ...No one came. There are connections but these days everyone do their own things.

No one went from here .. You are seeing my brother-in-law didn't go. My brother-in-law. No one went away. No one went away. It is like men are not allowed inside. If my brother went also s they were sent back from there. Gaurd didn't allow. ladies,ladies go inside . (For burial) No, no one had gone from here… .. Yes, had given it ..It was taken from our brother-in-law .

Now they used to go to work. So it is not like government job. We have to eat like that. They had taken. Now there were males in the house. They used to live on rent. You need to have money. If you stay empty handed or you work. expenses will be there. (Rest there are relatives, from your mother-in-law's side how is your relationship with them?) it is ok…we do not have so much people, there are three brothers, his brother. ..my two brother-in-laws and his father….we do not even have a sister-in-law, we do not have a mother-in-law, only our father-in-law

(Do you keep talking to them on the phone) Yes, I keep on talking on the phone (you get full support from them) Yes ... Whatever happened, the child will be yours, so after that you feel like this from inside It seems that I will pay more attention to these three children… (or as before, you were paying attention earlier too, did you start to feel it inside after the child is over, not my A child is therefore spoiled.) Now it remains that he has gone but it is true that all three should be right…

(At this time, who is the person in the family who has been the most supportive) No, no, no one came in the family… So our mother came, they only explained…. 10 days It is a matter of…

14. What were the reactions / counselling and suggestions given by the relatives/neighbours/ community after the episode?

N/A

15. Do you think and /or discuss with your spouse/ family member about the loss and related events and blame someone/something or feel guilty for the same?

(Do you still keep thinking about the child) and will not miss your child…

Brother, what do you talk about, still about the child) Yes, and what (he has still forgotten?) No, they do not say in front of children, if they are alone, they say something, that I don't know what happened, what is it ... that the child was there, it got over, Had they seen it, had they refused so I would have taken it to private.

If, child had been in stomach, one - one and a half day, Had it got over in one night then we would have known. Had it got over then doctors are there living downstairs. Now they also say. They also checked. Atter check up they also told it is all right.

(Do You blame someone in this) What to blame, It is just, that had doctors been more careful then may be my child was alive.

(Brother, who do you blame ?) What will he blame…. he says that there was more carelessness there. If they had seen, had seen properly, now who knows what happened, when he was being delivered so may be his neck was gripped. There was baby's poop outside.

When the delivery was done, it was alright, so may be there was breath inside. May be after some time poop came out. I don't know whether check up was done by doctors or not. They have seen or not. May be they have given like that. Had you tried may be there was breath inside him. They have given like this.

16. When did father and mother (if working) returned to work after the event?

(When you came back from the hospital, then from when did you start taking care of the house) My mother stayed for 10 days. Then this girl. She does it. I still concentrate there. It is now two months.. in January… What will you take, go to Didi… Now, today, ten days since the delivery was done. Boy was born to our elder daughter-in-law. If she sees him then she cries. (Brother, after how many days did he start going to work) It took us a week. (did not go to work for one weak?) yes.

17. How your and your family's life has changed after the event?
N/A

Knowing the cause of stillbirth in detail – autopsy and MITS

18. What was the cause of stillbirth mentioned to you and what is your perception about the value of knowing the cause?

(Have you ever tried to ask the doctor,due to what reason your child has been spoiled) Doctors do not talk, To listen to them, they do not have time to listen. Doctor people don't talk to anyone. (Your husband asked) No, he had made us run away. He had asked that it is not possible. If the treatement has been going on here since beginning. If there was any deficiency, then they would have told…..they used to stand up together to fight.

He said that we have killed your child. They were saying like that ... no, there was no reason, and later, stayed for so many days. So no check was done. It happens like how the child got finished. Nothing like that….they just came…gave medicine for fever…gave small medicine and then left. No check up from inside. Any test like how the child is born, nothing like that.

(Do you want to know why your child died) Yes, we want to know… how it happened, Had they told then we would have done something. if there was a deficiency. Then they would have told what to do.

Now if someone asks, if someone had come that how the child was finished. Now tell, when there is no reason so what could I say. what can I tell how the child died? check up was being done, It was right since beginning, So how it happened like that. (who asks like that?) Now if someone comes, some relative also, people in locality asks us.

You what will you ask, how did the child got over , what was the matter… What will you tell them, you will tell them that it got over from inside. They are saying that how it got over from inside. Had it died inside then you would have faced some problem… the child was right, the delivery was also correct… the child was also right, but how did this happen, all of us make us mad.

Your child was exchanged, everyone says that your child has changed… Many people have said, our relatives have also said that it is a big hospital, now she is all alone,how would you know that child has been changed…. Everybody says like that…who all would you stop.…… that this cannot happen. what we will say that the child was born right, It was right…. It was right even when it was admitted. After getting admitted, after delivery it got over.

19. In your view, what more could have been done to know the exact cause of stillbirth and the reasons causing it?

People would have done it.… We do not know…May be this is right… because the child will also be saved, how much is there in the child… this is more right… if you will find out the reason, then the child's soil will be right ...

20. Are you aware of any method for identifying the exact cause stillbirth?

(You know some way, in which we can know the reason behind this) What way, It is like when there is delivery then only it happens. Now you live there so there are thousands of people. Now if you will ask gaurd also then they will give this address. If you will ask the address then they will send you there.

21. What are your views about the autopsy/post mortem for identifying the cause of stillbirth?

(Do you know what a post mortem is) No ... I know that yes, yes ... but do not know where it is done ... (have you heard about the post mortem?) Yes yes…I have heard... (I know what happens in that) It is like if a man is finished, in an accident, so they would take away the good things ... In the post mortem, the body is cut.

(In the post mortem, the body is cut off ...) Nothing like that happened in our child .... (If the doctor had advised you to get the post mortem done at that time - would you have agreed) we would have known the reason.... Now if there has been a deficiency in the abdomen inside, there has been a deficiency in the child .. You could have know then you would have paid attention.

Now you did not get to know in all this. That what happened, how it ended… but it happened there only. It happened due to negligence of doctor…the time I was admitted…it did not take too long….The time I was admitted at 8:30 a.m, at 5 a.m …My son was born at 6. And I the meantime there was pain of labor. Had they done it quickly, they would have seen, Had they checked ... If there was any deficiency, then they were also telling about operation.
Though they are Saying that do it fast .. If there is any problem in the child, there is some problem in breathing, he would have got it done…but had they told then only……they did not see it… The time it was admitted they said let it move, as if it is injection of water.
like Bottle of water ,as if there is a shortage of water… Had they done then may be it could have been done faster. . Do you know what happened to him when he was feeling in the neck, It used to be just like that, it was done the same way….May be they could have seen it…May be my child would have survived…..They come only after the delivery is done…..That is what I have seen there…
(Is anything forbidden in your religion, if someone does post mortem) No ... If there is any problem, there is something good in it, then what is there in post mortem .... The reason will be known in that. What is the reason ...as soon as the child was born it was known. It would have been known well, They did not gave any medicine. Immediately after giving birth it was said that child is no more alive…… <child crying>

22. What are your views about collection of the tissue and body fluid samples for identifying the cause of stillbirth?

People do it. I don't know may be this is right. … because the child will also be saved, because it is very small in child….so it is much better….. this is more right… it will be done through this, if you will find out the reason, then the child's soil will be right ...

23. How parents and family members can be approached for the tissue and fluid sampling?

(In what way we could have explained then you would have understood) No, you tell, if you are explaining from the front, then it makes sense quickly ... Now this photo ... Those people did not tell anything, Now they did not tell anything…..Had they told us anything then we would have done something, we will get to know the reason….They did not tell anything….Now they stayed there…..They did not take it for child, they could have done our test.

24. In your view, what factors are important for acceptance of the tissue sampling by the parents and family members?

So that we get to know the reason. What was the problem inside, what was the matter, how our child was born… what has happened…What did not happen…. This is the point… Now if someone ask's , so what to tell that what was the problem…...Now If someone will ask what will you tell that what happened inside
No one. If had they explained it to my husband, we would have discussed then this could have happened. You don't require anyone from outside. It is my child so I am understanding. Why would other person understand.
If she would have explained it to her…Had they told us…So I would have explained her. , but they neither told them nor me .. They did not tell anyone… .. gave child, and said that go and do the last rites .. .. did not do any check, etc., Had they done then we will not refuse, we would have known which language.

Decision making dynamics

24. In routine day to day operation who takes decision about the major activities about the family?

(Who does the shopping in the house, etc., who does it?) They both do it (both of them do it). Yes. (Who takes the decision) decisions are like, they do it, both of them do it together, if we have to go somewhere, If we have to give something to someone. We both go… In the matter of vegetables They don't buy it, I buy it. whatever we take on our own choice….
<Baby Crying and Making Noise - Lot of Disturbance>

25. During the hospital stay who discussed with the doctors / nurses to make decision about the treatment and other procedures?

(The number of days you stayed in the hospital, whatever your treatment will be, etc .. Even for this decision … that the doctors are saying like this so, did your husband asked someone) Yes, the blood was charged for once, For that, for the report ... I called his father, so he had come home ... He said that the doctor said such things, then I was in the hospital, There was ladies, there so I got it done with it .... (This is a matter of) When I was in the hospital, After getting delivery done. After one or two days. We take it togehther, whatever is there we take it together.

26. After the delivery, who decided about the burial and other post- death activities and rituals?

(When the child was given , when child's body was given to you. You have to bury the Child's body,You have to do the work ahead … So who decided to do all this) When they gave it, so his father did it (did Husband himself take all this decision, or did he consult anyone)…He consulted…. he had a call my mother, and said…I do not have mother-in-law, only father-in-law. Khali .... So called my mother, and said that I will come, It will be evening..... It is like this, takeit, then they took .. He was taken to chandni chowk. When asked Had told that It has been done.

In-depth Interviews of parents who had Stillbirth-14

**1.1**	**Primary respondent (relation with the child)**	Mother, grandmother
**1.2**	**Age of the primary respondent**	Father-28 years<br>Mother-28 years
**1.3**	**Other member present**	Grandfather

2. Please let us know the course of pregnancy and events around the delivery/ termination. (Please document as the event were told by the respondent)

Grandfather - get ultrasound done every 15 days, you have to get this treatment done, flower treatment is done, Never mind the money was going, never mind, but when the delivery was going to happen within the last time, from the morning Their health was bad, now it is ladies but you are my child, now blood was going on a lot, they had the same time or So you should have done surgery, or if someone is not handling you, then you do something, another doctor Send, but they didn't, Last evening at 7-8:30 pm, we were told that this caesarean Is the case. You are watching from the morning, health is bad, you are being injected on injection, but They did not see all these things, took delivery of it at 4 o'clock, within half an hour Inside, we came out completely normal and we could not save the baby, and the baby I saw that the next day I did not see the night that day, it was absolutely white, that is, its Whatever happens to the mother, He was bitten and all the blood that he got out of the baby's body It was missed, it was absolutely white, this situation was at that time.

Grandmother- In the last, when they got water leakage around 2 o'clock in the night, I gave them Told me at 10 o'clock, I left work and took them there, so there, admit, what now? It is said that a pen injected in glucose comes in, the pens did not exist, so the pain Injected, kept on waiting, they did not tell me that they would do the operation, at 5.30 am in the last you call your son, and my son will also take an hour to come, from work. What did they say that they do the operation? I ran away like this with no luggage, I came to the house to get stuff I am for the child and for these. Just came back and asked there again, he was bleeding so much Bleeding has been so bleed, till then the sight of the child was not right even in the last, I Said what they say, now take them, they said for the operation.

Grandmother- Then I have left them till the labor room, to the hospital, to the room, just on that Even the child had a heartbeat, it says that when he senses me, he says that my spine Injected into the bone, then said bend me, now I have three operations, three When all three of my children became caesarean, I also did not turn them away like that, saying my feet and Bended by doing this. Till then the child was normal, till then the child was normal. And injected, Saying that I am getting so much pain, injected in place instead of spinal cord, this thing is complete Listening happens. So he put it, he is not getting it, after that what is said, get the operation cracked, and baby Tell that death has happened.

Grandfather - I have been carrying clothes ever since.

Grandmother- And 5 minutes ago I told my son that if he went in, the shirt inside If you bring it etc, then it is the same market nearby, tell it to go, phone doctor The beta of Nickel came. I said there

Grandmother- Actually I had found out after 3-3.50 months, they probably knew about it.

Because of this, he did not tell me.

Grandfather- not told

Grandmother- So they came to know, me, I know a little Hindi, so they came to know he told me it is from South Africa (asked if she belongs to other country). Yes we People are Christian yes they do it in English they do it in beta English only. There is no problem. So I show it to me directly, first my son Bansal is here. Bansal not sorry someone lives in my ashram, she has a hospital, she knows very well, she Had shown. I got you ultrasound and so on. I said, son, do you have any does not live, the elder son goes away, the younger son goes away, the middleman is gone, all three leave is. My husband is that even if he used to go short sometimes he used to stay alone. So how are we taken so leaving the children away, the younger sister is a little bit, meaning her mind is of a little child. And they have their first son then I said to whom I will leave. So I saw this hospital. So they made me believe that I will have it, this will happen.

mother- (crying) no, it was also you know, because my last ultrasound the doctor told me that delivery be only on 5 th day, ultrasound test is on 25 th of October will be delivery, from 25 th to 30 th so I was just expecting in that time, so, on the 26 th I went to sleep, because I have a son, he is 5 years, that first it was normal. So, I never experience like leakage and things, so------- that night I was sleeping, and more is like, I want to urine, but go and is no urine, but my whole like this, so I didn’t tell my mother in law. Morning by 10 o’clock, it was ------, I have to tell because I have no experience, I don’t know the thing ----------we held like this but so I told so she said ok, that time, it was Saturday, I think Saturday or Friday, I don’t know I went to the hospital, I went to the hospital, we are speaking to the doctor and she told me that I am going to give you injection, but knowing that this condition is operation. Because it will not be possible because, for me the child is very big. That I was evenly, you see I am not able to do this or move like this, so she told me I will give you injection, while give you drip, for pain to come so I said ok, when there was 11 o’clock in the morning, I went to the hospital, she gave me the first injection and she left, she went to her house the doctor. She left me in the ease of the nurses. They give me many drip, 2-3 drips, said no pain, asked me pain, I said no pain, I was still leaking water (water). Stay calm. So I speaking to doctor, she said ok. After 3 or 4 like that, the doctor came, and she said call your husband. I call my, my husband came inside. All that time everything was ok, I was still feeling that my baby is ok in my stomach. So they gave me a drip and they take me to the operation theater, because at the beginning I said doctor she said she is able to do everything, she has everything for Operation Theater. And when we enter, at that time because my -------- is not in my --------, so I enter, they were trying to look for my ribs, to put a injection, and then they …………too many. You can see the operation theater so so big, those boys, I don’t know where they are from, where they working, where they were so many in a room. And then they took my head like this, and bring it like this from my back, and they see my waist, almost let’s say 5-10 mins. So I expect, and this, my stomach was very big, I expect my child to breathe, I myself was losing breathe, so after that when I put it there, I lay down and a injection ----------me, and then it took me I never saw anything. My eyes open I am not able to talk. And then a women when she put a cloth, kapra, over my face not to see anything. Baby come, no voice, no cry, I m not hearing anything, I want to talk but I am not, I am not able to say anything. So after that they take me, they care me in a room. So I m not in my sense. So after sometime, I ask myself, I gave birth like say almost half an hour, so where is the Baby and then first she was always with me. So I ask her where the baby is and she told me that oh the baby have problem, like this, they take him to a near hospital for treatment, like that. And they never wanted to say anything to me, I keep asking, no response, so after my husband came in the room, I ask him, he say, oh like this like that, the child never make it, I say, huh, after 5, maybe my baby is ok in my Stomach and I enter Operation Theater, they trying to tell me that the baby is not alive, so how.
Grandmother- This ultrasound is done in the last, it has also shown in full, the child is absolutely right, Dharkan is right, all right, everything is correct. Everything was right, and you guys know how to eat them, the eggs are choking the eaters, we used to make them on our own, used to do everything of our own was not. The doctor herself was surprised that she meant, I am being taken after 15 days, 2 kg tell weight, then after 15 days I am carrying again, then 2 kg weight, what It is said that it had become 60 kg, 60 kg. When they gave me time, they mean 25 more the 30th date was given according to ultrasound, it will happen anytime between 25th - 30th dates. Then the weight and weight of that child was also increased. Said for good sugar, also tested sugar made everything, nothing came out. Ok quite a lot was a healthy kid, everything was good, and the stomach was also big.
Mother- My weight is 40 kg. The baby is big in the stomach.
Grandmother- By the evening he is waiting so much, now his condition is getting worse. They are watching the check-up has been erected standing at once water has come out, a lot.
Mother- she check up, doctor check up on me, lie down on the bed, so I, full cloth
Went bad in water
Grandmother- that doctor, meaning the doctor who was a child, neither did I stand there, 10 min
Done, how much time it takes to operate, it has become 10 min, 20 min half an hour Gone. I said neither the voice of the child is ascending, nor something ascending, I kept sending my son Brother, bring them

some maxi and so, if he had gone to the market, then I am standing, of course In front of the door, no sound, nor anything, just like silence is inside.
Grandfather- and it was absolutely right, until the last was perfect, two days ago my son game He was kicking the kid, he was playing.
Grandfather- You is also a doctor, tells me one thing when you took me to the operation theater Very normal, the child is walking, watching is completely normal. Went to the operation theater you may have also seen children being born, there is no white child at all. Ok my grandson Secondly, color is like them, yes Savla is a little bit, that child is exactly what I can say, and it was white like milk. The blood had left completely.
Grandmother- Because all the blood had come out of her mouth, because she was lying on the ground, when Were taking it to the operation theater, then my elder son, his husband, he had put his hand on the stomach, so The child moves, does it, so he did. It is said that everything is correct, everything inside checked, labor Checked inside the room, even then the baby's clutch was all right, she says when I bend me neither my sauce stops Gone, its sauce has stopped, what does it say, something happened to the child. Immediately operated after that.
Grandmother- Because all the blood had come out of her mouth, because she was lying on the ground, when Were taking it to the operation theater, then my elder son, his husband, he had put his hand on the stomach, so The child moves, does it, so he did. It is said that everything is correct, everything inside checked, labor Checked inside the room, even then the baby's clutch was all right, she says when I bend me neither my sauce stops Gone, its sauce has stopped, what does it say, something happened to the child. Immediately operated after that.
Grandfather - by eating, never by anything, whoever wrote, ... Box, Horlicks, Bornavita, Joe Everything has to be eaten, whatever you want, whatever she is giving by writing, and here you take it.
2.1. Can you please let us know about the problems, illness and events immediately before the delivery?
Grandmother- Savita nursing home, yes yes (pehladpur)
Grandmother- White and 5 kg baby was 5 kg, she used to be surprised herself. What are you feeding the food, even the weight of the child was so much, 60 kgs, both of them, mother Son's
Mother- So, two more, the first place, she was not there, first place, me, my husband went for the test and then this ultrasound told me that this the baby moves, baby everything is ok. No problem, the next time I went to she told me because that place is far, I should so I can go there I have to come here and she told me, where we have to go hospital, and everyday every time I go no problem. This is my miracle, no Problem there is no anything like the baby has this or you have this that is going to affect the child in your stomach to give problem. No problem, everything normal. Eat my food on time, food I am not short of food. ……………everything, nothing.
Grandfather- There is not much baby in the doctor's say more than 3-3.5 kg. Any Not cheese (problem), 9 monthas means whatever, meat mutton job also has to be eaten, fish Eat the same thing. Milk everything.
2.2. Where did you go or whom did you consult for any problem during the last pregnancy?
Grandmother- savita nursing home (pehladpur)
4. Could you please describe your overall experience about the hospital care? (Probe:investigations, treatment given, involvement/ participation in decision making, satisfaction level, quality of care, cost of care)
Grandfather- Private, what is the hospital with shutter, puzzle saw it, I said you used to go in it?
Grandfather- you will go and see whether this hall is so big or not, there is a hospital inside it, this hospital is so big, when it is closed from the front, it opens with a shutter, and it is a hospital.
Grandfather - if and after that, I am going to see him, the hospital staff, you will not believe who is the sweeper, the one who sweeps, is standing in the operation theater, and my boy told me that my father Full blood like this, spread on the ground, and just like a small operation theater with nothing inside, someone No facilities, of course, nothing was this thing.
(asked about cost)
Mother- they said 30,000 operation everything nursing everything, in that 30,000.

Grandmother-expense, how much it cost.
Grandfather-spends, saying, how much did the spends cost. Do you know what i know its father knows
Grandmother- He told me that around 30,000 would mean the time of operation.
Mother-nothing is theater, same bed, nothing is theater.
Grandfather- I was surprised to see them, and the one who works, sweeper, He was standing inside, was over staff, in the operation theater, it was so.
Grandmother- normal, normal delivery
Grandfather- hey .... what is given to the child in the beginning, to the mother, nothing. It is normal. Now delivery in their bed, I delivered See, after two days, they become aware, they have nothing, this was the condition of that place.
Grandfather- He has another beta, another grandson of mine has gone to school, see him.
Grandmother- It was normal, normal delivery.
Grandfather- Now he was born there, now look at his country and we see the country of people, There is no facility, even then the child is delivered safely, well delivered, well done. Everything but after we are here, this is the situation.
Grandfather- a staff that is, staff you see. He is a sweeper who sweeps, what is his work in Operation Theater? You have also seen Operation Theater, You complete the entire dress, correct it then you are going to the operation theater, slippers Going to Pehenke This is messing about
5. Could you please describe your experience about communication by the various hospital staffs during hospital stay? (Probe:who all communicated, who was the primary communicator, nurses, doctors, other staffs, frequency of communication, mode of communication, details, completeness of information, skill of communicator, consistency in communication)
Mother – in the beginning I started only there, no problem, everything is ok, she is always you know tell eat this or this. We never had any, no problem. Even with the nurses in a place, we always talk, we always leisure. No problem, there is no change, always tell me. Her behavior was good but when I lost my baby, it was not good to me. During the time of my delivery when I went there, there was no any have communication, when I went there they treated me did everything, that was ok no problem. Like this like this, She is like this, everything was fine, no problem. We never had any, everytime I go there, no problem, no problem. I always, we leisure, of course they don't know me but I share joke with the mam, no problem, like this. during the time of delivery it was at that time it was like, you know, she left me in a hospital, knowing that my case is operation. And she went to her house, 3-4 hours, to go and sit. Before she come 5 o'clock evening, I left at 11 o'clock from this place and she go to her house and 5 o'clock she come back, in the hospital, 5 hours.
Grandmother- was normal, normal, there was nothing like talking rudely nothing Like this, but he made a little mistake that when he saw that leakage was happening, then only speak to me Did not give, was admitted, nor do we have to operate, we cannot give time. Did not say.
Grandmother- I kept asking them, Madam, I said, what is Sari facility?
I showed them here from Grandmother- Starting, I said let's go close to home, talk to the doctor, I all Some, they keep convincing me till the last time, that will be normal, there will be no operation Will not do.
Grandfather - it's like this, there is no delivery, I will do normal, don't worry, don't do it, man. A little faith comes in, let's brother is fulfilling his responsibility, okay.
Grandmother- So I mean, I made all the checks up, I showed everything there, I kept asking the same thing again and again. If you are not in it, Madam, then you should tell me, what did you say, whether or not your bus is there, then say No, we have operations here, we have normal here, so I said let's be normal is near the house The children would come, just by thinking this.
Grandfather- In the best hospital, Rohtak is inside Haryana, my children are there, Mehne se mehenge Hospital, you don't worry at all, the child will have nothing, it will not happen, normal delivery will happen. All three were Caesareans. Absolutely white huh hua kid, such a innocent baby.
Grandfather- Now the leaders had stood there very big, the Gujars had stood very big, now in front of them what to say, now I have one child, now I will not want to lose 3 children. What is their thing

One will be standing, 10 will stand with him, let's get him up and make it strong. Here it is. People are living here, but there is no place to live here.
Human beings have nothing to live here. In front of him, just in front of the hospital, his office, that is, what his name is, has been retained by the people of the Janata Party, all of them were standing, working, do it, do it, get it. Goes yes, my boy said that the younger brother would have been in your house. The police started speaking to us went. We have to suppress ourselves our child is gone, my boy speaks, do not speak otherwise Will beat you. Now what, one is gone, now I will not give 3 back a little. What is their thing
Grandmother- Look at me, I was also tricked because now you are telling me that the person in front
Ok, I can do it, I can do it. Now I will be your trust And in the last What do you say with me, I said this thing, I said I believed you till the last, I What did you get, what did you give? Yes, silent (asked what doctor's response then), there Was sitting in the room, now as it is a labor room, it is a room, a room has become such a ward Is kept Then there is a check in front, then such an entry, a long way is made in it. Till then he kept telling me that right is right, right is right. See how healthy a child is.
6. Could you please tell us about the declaration of the event/outcome (stillbirth)? (Probe:who declared the outcome, cause explained, the body language and expression, preceding events before delivery, meeting with any senior doctor/member, attitude/body language/expression/skill of the communicator, expression of empathy/sympathy, language and simplicity, perceived completeness of information)
(asked Who informed about the death)
Grandmother- Then he came to the doctor's part called your son, your son said. So i said now
Are you calling me, son, come soon, the doctors are calling, so he came, telling me right away
The child has died. I said how it happened we say what we did not know How it happened, the doctor says I am still angry, I told you I am, just 5 minutes ago Everything was right, everything was right. Still you guys did this. Are saying we try our best, we pumped it, gave it nothing, nothing happened.
Grandfather - now I started adding my hands and feet, now ours is gone, now what is this fight to fight? We do not have things in it. So God knows that he is okay, but it's wrong.
7. What was your and your family member's reaction when you were told about the stillbirth? (Probe: what was the reaction of mother, father, other family members who were present, did someone try to console you or your family member)
Grandfather- My boy till now, that boy has broken so much in our house till 24th It never happened, it was a human child to take care of even a small dog in my house It is keep tell, this is so bad we cannot tell you the whole family of the people. Too just casually.
Grandfather- We did not know that our child was gone.
Grandmother- We were not conscious, crying so much, handling the son, told everyone in the house
Such a thing has happened then my sister came from here to the ashram. She came, she again said that brother our child was absolutely right, everything was there, asked the son, I will do a police case. So police case we also had it made. So they made an agreement with us. So let's say my son
My daughter-in-law, we did not get anything at that time. So that people should compromise.
The son said that you do not answer me tomorrow my child will have something for my wife
If I had gone, then what would you have done, answer me of that thing, then she went, saying I am
I will put in embassy I will talk in their embassy. So those people went, they said agreement,
Then sorry wory asked.
Then it is said that money is given like that, money is given to us, children have someone
Do not pass. Because my children, all three were shaken, that they stopped crying of these people
It was a bad situation, how it happened.
Grandmother- Now this is asking me what is the mother baby now it's a situation, I call it
If I tell you it would be difficult to handle them, then I did not tell you, my son in the morning
Told.
Grandfather- The next day when I am there, when I saw the child, I saw the nails, that
It was completely white all these lips were completely white. This is what blood shows, from lips, our

From the ear, it was all white, this forehead was completely white. We still thank God Lets do nothing for the girl. Otherwise, do we see anyone.

8. How was your experience about the procedure after death till handover of the body to you? (Probe:processes asked to follow, documentation and papers given, interaction and behaviour of the staffs, respect for religious norms, time taken, cost/payment, assistance from the staffs)

Grandmother- no baby we were not given, just at that time we said that brother show us
How is the child, then he showed, completely white, completely white. So let's tie here, right?
Baby's chord, which is tied, it was not there at that time, so you see, baby, you see it, non also
Seeing that he was not his own, he also had tears in his eyes, he was such a lovely child,
Grandmother- Then at the same hospital again at 4.30 in the night, our burial ground in Burari, how can we Will take them, request them to keep the same child, you guys, then they kept the child again
At night. Then from 7-8:30 in the morning, I wrote the letter to the father and so on, we, the son, and these people, I had them, son, my husband was on fire. I was inside, I had not left them, I was there for a minute Also, (asking family member), everyone had gone, sister-in-law, gents, meaning ladies, daughter of sister it was mine to sleep.
Grandmother- she knows son, (asking her son how much aman (elder son) has paid in burial ground).
Uncle (chachcha) - has called him, will call back (aman), now guess, at that time Didn't notice, how much is going, how much is not.

9. Who all from your family and community/neighbourhood were present at the hospital and how they supported you during this period? (Probe:who informed them, who all came, what type support given) (asked about family members support)

No one has come from Grandmother-Bahar, because no one has told anyone, they do their work
I was there, no one came, only one of my brothers came that too after 3 days, then he sat for a while
She was gone, she was not well then, saying I am going to my house, then she is in her house
She had gone (support from family members) There was nothing, not as the sorrows were spoken, just
(Asked who were there in the hospital)
Grandmother- No, I was her husband, and my son-in-law's son was his elder, his younger The son was with me there.
(asked if the family members were allowed to visit the mother)
Grandmother- No, do not let the time of operation go in, do you mean even after the operation
Till he did not bring him to the ward, he was not allowed to meet. And in that my son, because I am
They had it my son went to the operation theater. Down so much blood, blood itself blood. This much
At that time there was blood.
Grandfather- He: He is saying this; He: He is saying, Everyone retracted from each other, that was the situation, that Of place It is not taking any responsibility that yes because of me.

10. What all rituals and procedures did you follow after stillbirth? (Probe: body preparation, who prepared, burial/ cremation, other rituals and timings, who performed them according to the rituals)

Grandmother- Yes, prayer is always there. 3 rd day, third day is in us. So father came In our house
Grandfather- 27 th ko… .. aww…. On 26th, he died, then the next day on 27th date Graveyard was taken, then 3 days after that, which is 3 days after death, prayer It happens to us, then after 60 days from death, once again there is love, then whatever you poor Want to give to …… is 60.
Grandmother- you must have 13 days, then we have 60.
(asked if bathing done, new clothes worn by baby)
Grandmother- They are all done, they were not brought home in the hospital, didn't we, from the hospital We were taken straight. No (asked if grandmother did the death rituals), I have a Relative, he did, it was him, and he had a daughter, clothes, sponge everything on the same, clothes After doing everything, he was not being seen by us, how, if he did, he said you guys Stay here, if I do, then wear everything, clothes, Blanket up in a new towel He did everything he wore.
Grandfather- No, we are the one, our one is there in Sangam Vihar, one is inside Burari, which
The one at Sangam Vihar is completely covered, there is no body there now… yes, it was spoiled there.

11. How did you, your spouse (husband/wife) and immediate family members cope with the difficult phase? (Probe: any change in the psychological state, physical state, engagement in daily work, spiritual/religious activities)
Grandma: We only know that emerged, yet, not yet, I would have missed the child We ask ourselves, how did this happen?
(Asked how did mother cope)
Grandmother- weeps, weeps, weeps, weeps inside. Now i leave alone No, I am a small sister now, we have not hired any servant. I see all the work, some of them If it does not happen, then I do not speak them like that, then I bring them here from this room, that you A little mind change will take place. The child has gone to school.
(Asked who's child)
Grandmother- Their beta is big, not 5 years old, there is a school in front of it, in ....
(asked mother how did she cope up or face difficulty in daily household work)
Mother- what can I do, its already happened. I just have to, always (think again and again about the child.
12. Is/was there any change in the interpersonal relationships between you and your spouse and other family members after the event? (Probe: relation between husband and wife; relation and attachment with other children, is present; relationship with other family member who are physically present; contact with members who are physically not present but contacted over phone)
Mother-No problem, the only problem was when I was speaking of the pregnancy I was shame, to tell my mother in law, I didn't tell her anything that I am pregnant like this, so after 3 months something like that, I then have to tell, because there is nothing I can do.
(asked inter-personal relation with spouse)
Mother- He is more caring, more than before. No, that's what I m saying, he is taking more care than before.
(asked relation with other family members)
Mother- they are give, showing me more, even not this baby, but even before. There is no problem, everything
(getting support from other family members).
14. What were the reactions/ counselling and suggestions given by the relatives/neighbours/ community after the episode? (Probe:what to do after death, following the rituals, knowing the cause of death, any other)
Grandfather- holy family is the best hospital, close to Jamia, best Hospital, this is what I told them in the beginning, Masjidiya is also a good hospital, you are in S Hospital Going brother, I was in the last, in the last 6 months, then I saw this hospital, all right See everything is right, there is no reason to panic.
15. Do you think and/or discuss with your spouse/ family member(s) about the loss and related events and blame someone/something or feel guilty for the same? (Probe: with whom; how frequently; the context of discussion; contents of discussion; whom do you/others blame for the death or what about you feel guilty)
Grandfather- If you see a child, I say such a beautiful child, such a beautiful child. Get out of hand Gone due to negligence.
Grandfather - be very careless, be very careless, don't do this to you, don't There is little of any Panwari shop that is not known, do not put another like this, do not you Is the life of man
Mother-I ask, like say, the doctor never told, but may be in a hospital, they are all tell me that this is not the cause of the doctor, who cause, am I the cause, am I the reason, so what happen, no response upto today, from 26 th of October.
Grandmother- So much water drained out at once, he started speaking even on the baby, whenever the doctor Did not say that the operation has to be done, at the same time the operation would have been done or not.
Grandfather- has opened small shops just for the money. There is no degree, nothing is there.
Grandma- While I am watching everything with my own eyes, for 5 minutes before going inside

The child was right I did not yet understand how this happened.
Grandfather- Now we are also as a doctor, we are also sitting right here man next too She does deliver, maybe it will also do well, but she did carelessness.
(asked who does she blame anyone)
Mother- Doctor, of course, because, I was ok, I had no problem, I went to operation theater, baby was still breathing, everything was ok for me, so after 15 minutes, why they did not tell me, what's the problem. What's the cause? She is the cause? In the beginning, she is not the cause, after go to the hospital 11 o'clock; 2 o'clock, 1 o'clock, operation finish, Baby is ok. She left me and go to house, 5 hours, so…..…5 hours, she left me in a hospital.
Grandfather - It was a small clinic, the biggest mistake was from my Mrs, I am their father in law, my Beta is the one whom V is married to. He was the grandson who died. So these people have not seen but she was a doctor He very much satisfied that I will do it, the puzzle bar was normal delivery, now don't worry at all, He saw that he is a foreigner, so that I can get good money, because whenever he used to go see it You have to have an ultrasound, now I have three children too, so the doctors don't get the ultrasound done again and again Even the rays reads above the child.
Mother-Of course, of course I am able (asked if mother is still able to speak her mind and share things with her husband), he is my husband, why should I. They are always tell me the problem is that, I want to know the cause of the death of my child, but after that I am not getting from anybody, even doctor what's the cause, what happened? What happened, child is ok, everything is fine. Then what happened.
17. How your and your family's life has changed after the event? (Probe: what has changed, what is the major change, what do you care more now than before; plans for future pregnancy, any substance abuse- new behaviour or increase in the existing behaviour)
Grandfather - let's be with our child, never mind I agree but there are more children, all of parents Hearts are not those.
Grandfather- Look at those stiches, just as you have torn an animal, like an arrow command Have been kept, see if their stomach is right, Poo was read in the middle, there is no explanation at all, like
Mother- No, for now I m not doing anything, I m not able to do anyting, because the pain is too much. She always tells me lets go to that hospital, I say I am not going to that hospital.
Grandmother- Now this rate is not going to the hospital, I will not go, I will not go.
18. What was the cause of stillbirth mentioned to you and what is your perception about the value of knowing the cause? (Probe: knowledge about the cause of stillbirth, willingness to know the detailed cause, potential factors that led to the illness/death, potential impact on the other family members or next pregnancy)
Grandmother- Did not tell anything, we do not understand ourselves, when our after the operation came, when we were called, we were all children and all were called I said to myself that how the child died, I said
Mother- It all my miracle, everything is ok, I never had any womb problem, when I go to hospital, like this problem, this that problem, anytime for my weight, everything is all ok, like this like this, so which problem, what's the cause, after that, I am not getting any response to tell me what's the cause of the death of my child.
Mother-I have no problem so what is the cause of, my baby 5 kilo, baby. What is the cause of the death.
19. In your view, what more could have been done to know the exact cause of
stillbirthand the reasons causing it? (Probe:investigation, autopsy, any other)
20.Are you aware of any method for identifying the exact cause stillbirth? (Probe:autopsy, investigations, etc.)
21. What are your views about the autopsy/ post-mortem for identifying the cause of stillbirth?(Probe:necessity, religious aspect, time needed, disfigurement, etc.)
Grandmother- how to post mortem of so many children One of my two friends for the grandfather-post mortem, said that you post mortem Make it But what did you say at that time the situation was such that

you would see his father, the child, he Saying that my child will tear and fly, then the policemen say, hand over us, 3 days After you take from us. So all these things are not seen, now one has gone tearing it By doing what you want to see, we know we have been negligent, now what will come out even after that, Man and gatherings will become their, you settle it. What will you do, take the money.
This is the thing, you want to give 2 lakhs, 4 lakhs, 5 lakhs, you want to give .... Now all these things don't happen Is it not, this is a small, innocent child, now gone from the world, now after tearing it, what will you see, big So, let the man become an accident, if something happens, let's get his post-mortem done, What should the child do post-mortem. This father was also told by his father what to do, I I said that I will not be seen that, because of this we became a little on the side.

22. What are your views about collection of the tissue (like done for biopsy to diagnose diseases) and body fluid samples for identifying the cause of stillbirth? (Probe: necessity, religious aspect, time needed, disfigurement, etc.)

Grandfather- if we had got a good result, we would have understood someone

23. How parents and family members can be approached for the tissue and fluid sampling? (Probe: who should approach, whom, when and how should approach; what should be told and in what detail; should some picture of the method be used to explain)

Had I been a grandfather- father, I would have been, if anyone would have guided that time. when someone Was not just going to guide. If someone at that time, like you are an NGO, if someone Come, if there was such a thing, we would definitely cooperate, the result would come out, something It will be good for our child. It would have been seen at that time, how the moment would have been, how This, which was the matter on 24th Tariq, is no longer a matter, so then to take a decision.

Grandfather- no, (picture be used to explain) so much madam everyone has knowledge at home Is, all the children are educated, well, my mother was also a staff nurse, my father gave two Marraige was my second mother, who was a warden in the S Hospital cancer ward. So since childhood, till now you remain in the doctor, then you have so much knowledge that if you understand At that time, it does not take much time, we support you. grandfather- First of all, you had to read to convey it to mother father, brother, because Main is the same, no matter how many people love their grandchildren, that is not the case, but, first parents If he is conveyed, well, he is satisfied with your words.
Grandfather- yes if at that time, mother, father, let me tell you, then you would go with people , Why not go, it is a good thing if medical science has proved so much, so much If we have advanced, we should also support.

24. In your view, what factors are important for acceptance of the tissue sampling by the parents and family members? (Probe:desire to know the cause, trust, confidence on the health care provider)

Grandfather- we will find out the reason why the child died, if medical Science has proved so much, is giving such a good result so well and good, very good The thing is for us people, now we are running a false thinking that, may be it happened Will, may be like that, but the main reason is that we do not know people yet. She is only the doctor who has done that delivery, she is public. Otherwise, it turns out See, the body of the child is absolutely white, it may have been accidentally cut, blood may have gone. Have not given oxygen time, or have given it to the mother .... they have given more, many questions, Meaning the mind had a lot of questions inside the mind, but the main result is not.

25. In routine day to day operation who takes decision about the major activities (purchases, treatment, spending money) about the family? (Probe: involvement of the mother/women in the family, any other family member, consultation with any other member)

Grandfather- I only take it, I am big (purchase). If there is talk of vegetable then it is ok Come and go again, now he is giving 10 rupees, he is giving 20 rupees, well as you bargaining Do it and bring it In it, all four fathers would sit, my Mrs. would sit, everyone would sit and take decisions. Decision is also seen by matter, what is the matter. Family no matter what No matter if someone is relative, then all that will come together is the same.

(asking decision on treatment)
Grandfather- The time you were diagnosed, from that day till the last, Who was there, I am a little daughter, but my daughter, but I can not talk so openly From these, I used to ask my Mrs., I used to ask my elder son what the doctor is saying. Ok, I used to see the report, as soon as I entered, I used to take the report straight Inside the room, I used to watch the whole. Used to call her Mrs. What is all right is a good hospital It is not a good doctor, if you change. But from here I got the same response that right This is it
27.After the delivery, who decided about the burial and other post-death activities and rituals? (Probe: father, mother, any other family member; did you/your spouse talk to someone in your family like elders or relatives to take critical decisions)
Grandfather- So he had to take the next day, the one who is going to walk, he is We have to do it, the last rites are all there, we have to do it.
28. Now, we are completing the interaction/ interview. Would you like to add anything?
Grandfather- My mother was also a staff nurse, we too have spent our childhood in doctors. On sight Did recognize that there is absolutely no blood in this child. The child that is with the mother, she has Is cut incorrectly and the blood is full of the child. The child was completely white, the blood finished. Dropped blood from child. The whole force is saying that such blood is filled inside him. My 3 Son, the doctor she was was our family doctor, she took us to the operation theater when Delivery was made of my first son, I say that not even a little blood had fallen in it. And in 10 min He delivered stiches after delivery, gave the child outside. Such people are like this inside.
Grandfather- Actually the belt that is here is not the belt of Gujaro. If we have a little bit Even if you pressurized, 10 people would stand up. They enter us to fight. Now our Rohtak would be on the side, if Delhi's main were in it, then no human voice would arise, Can not raise voice here. This belt is very dirty, it is very dirty belt, even if you are your cousin No, but you put all the sauces in it. The pass here is like this, it is asked here that Gujjar has made a mistake, So I will not accept his mistake. They can speak your mistake. So know in our Christian There is no one is happy to help anyone. Either just bible, church, We will spend 1 hour in the church too, so we are christian, come out anyway. bible is at hand then Is christian. Keep it bible then finish the matter. Nobody stands up for someone can. Call father, father says first you pay that money, then donate money then go grave will be found. This is a cleansing fact in our christians, in our christian. Nothing left. My mother died, phoned the father that this is how the father became, one year The donation has not come First you should donate My mother has died. You first do the work that is your responsibility: do the work. But he got me a donation of 5400 rupees before me. Church is being built in sangam vihar, it makes so much of your money, forcefully from me He took the money 11,001, giving him a 5400 one, then my mother had found a grave, then she I found a place, this is christian, what to do. It is said that when there is an hour in the church Everyone is christian, they consider themselves very good. But as soon as he came out of the church, nothing was over. I took christian hand in bible. After that no one is happy with the help is. These pastors this father, what shall I say to them, God has chosen you, has placed you at this place To you, you help someone, donations will continue, what God has said to give. If is You do not have two, do not have with you, do you steal and if you donate, what did you give?
Grandfather- biggest change, biggest change, what i see, you go with me, this In the street, in that street, in that street, there are satole doctors around the world who write Rx Its meaning inside the starting is not known. Okay, they have 3 tablet, one capsule, one red which is vitamin, and a white color, whether you have fever, whether you have enough There is something, they will give it to you and will take 35 rupees from you. So when will this system end? Write in Have kept MBBS, this is that, no where, london is written, they don't know London is London where. So you change this system.
Grandfather- madam, neither can you change this system, nor change this system I can. This system is very impaired. We can get all the paperwork done, how many Make a movement, but we do not change the system that has run from above Can. This will continue like this, such children will keep going, this way the world will keep going, that's all. Give 5000 rupees, make your license of the bus, sit in the bus, you do not know how to drive the bus, 58, 55 Man, you are leaving on the road, 55, 58, knowing you are leaving, you are 500, 1000 You missed by giving Rs. The system is running poorly from above. Add from

America is extreme Taking an add-on, you get a 5 lakh machine to write 10 million in paper and pass it If you are, then from the system, what will you change? Where will the house change?

There are so many, let me go, take the flag, I will also walk, if change happens. If the child survives, I I will leave But those who are sitting on top of it, their eyes are speech, blood and water is all over inside. Even if the children of his house die, they are not worried. The chair should not be worn.

In-depth Interviews of parents who had Stillbirth-15

**1.1**	**Primary respondent (relation with the child)**	Mother
**1.2**	**Age of the primary respondent**	20-25 years
**1.3**	**Other member present**	Sister in law (jethani), mother in law

2.Please let us know the course of pregnancy and events around the delivery/ termination. (Please document as the event were told by the respondent)
2.1. Can you please let us know about the problems, illness and events immediately before the delivery?
2.2. Where did you go or whom did you consult for any problem during the last pregnancy?
Mother: At first there was no problem, then after that I had problems in the seventh month, then lightly There was a pan in the stomach, it was piercing down here, it was hurting like this, then showed it in the hospital, then said The baby's heart rate is very slow (you had problems during pregnancy)
Mother: In M (Where shown)
Jethani: There is a hospital in M, VB was showing them from the very beginning, in the fifth month The fourth month had gone to the village, it was going there, so from there to the seventh, the seventh month was over, When the seventh started running, it broke here, after that what happened to you in the village, they had trouble within two-three days All done.
Grand Mother: We brought it (these villages were gone then after coming back)
Jethani: Arrived here on Sunday, Monday, they did not tell me on Monday, I asked them that baby move If you are doing, then you said that you are not doing it like didi was doing in the village, then I told my mother They said that if they have come from the journey, then there will be tremors, so it will be sluggish, if you do not go in this round On the second day on Tuesday, he said that my sister is getting tight and tight, So I showed anyone that there is no problem, there is a right in the village, nor did I call them and show them that if there is any problem then I will leave you Hospital, on Tuesday, she came and said that there is nothing, the child is sluggish because of the journey, so I said that right I have to go to the show on Wednesday, otherwise I would have shown it in the emergency, I would have known something When she went to show him on Wednesday, she told that the baby's heartbeat is low, when the ultrasound was done. After the ultrasound was told that the child had deteriorated, just someone more than this The problem did not just happen here; the seventh month, the seventh month was taken from 21 ways, and 24 This is the case with Tarikh (they went to the village then after coming back)
Jethani: Yes, he referred from there to VB then we referred to S Hospital.
Jethani: Knowing not only the right was a little bit like that, we had also shown us the mother and showed her (and the right one too)
Grand Mother: If they had two, they would say that it was okay or they would say that they are hurting now. Go, we used to go, but this big one happened there
Jethani: We also showed him, so there was a little faith in him that when he used to say take it, he used to take it, by the way They did not have much problem, so I said that if it is being tight and tight, then they do not speak, so new If there were more problems, then we would have gone immediately with them at that time, a little carelessness that happened If taken immediately, maybe something happens,
3. Now we would request you to tell us something about the period of hospitalisation for the pregnancy, delivery or termination? (Probe:hospital, ward, duration of stay, course of illness)
Jethani: In the hospital, we stayed till 29, from 24 to 29, admitted 24, yes (6 Day) On 27th, at 10 o'clock in the night, 10 minutes were left, the child was born, (so how long in the hospital are)
Jethani: Yes at 2 pm (2 pm)
4. Could you please describe your overall experience about the hospital care? (Probe:investigations, treatment given, involvement/ participation in decision making, satisfaction level, quality of care, cost of care)

Jethani: Yes, I was admitted on 24th, after being admitted there, at first I kept like this all night, nothing said No, kept like this all night, just checked, took the whole ultrasound room there and then Ultrasound was all checked up and then asked to admit at night,
Something at night after admitting did not happen then he gave medicine in the morning. Then gave the medicine, then in the evening the medicine started increasing again so that they had pain They came, then the medicine was administered three times, yet they did not feel any pain, then after that they gave them the pill To have more pain, gave them pill twice, then they got pain from right, they are normal like this Born a child (what happened during recruitment there)
Jethani: I was given two days, I used to come home, gave them two days, then they were shot and delivery was done, then They were delivered at night on the 27th, at the moment, it is fine, but they are not well Lives
Grand Mother: It is not right, nothing goes on or at home, then it hurts and hands and feet start tingling.
Grand Mother: Something is not right, nothing does (just started happening)
Jethani: From the beginning, where does something come from?
Grand Mother: Yes since (since delivery), not before, nothing happens
Jethani: Yes, yes, it has happened since then, sometimes it hurts in the stomach, sometimes in the hands and feet, there is a little bit of stomach pain, I said show it. Then do you think didi give pain in delivery pan because of that it hurts?
Grand Mother: Massage done, yes massage done for 10-15 days.
Jethani: Well there is not much problem, it keeps on moving, it is not that it is only that it speaks that I They have no pain in the stomach, pain in the arms and legs is due to weekness, no, I said slowly Will recover, food sometimes says never eats, I don't feel like it, eat later
Grand Mother: There is no life in the hands and feet, I get dizzy, this daughter does not eat food, I bring milk so I explain many things. Say this is the wrong place, the reverse is coming, the opposite is very brother She comes, does not eat or drink anything because of it, has been doing the reverse since morning, we said, "God, what should I do Do not do anything, we do not stop them from doing anything.
Mother: Doesn't feel like eating, what to do
Jethani: Don't know now why they vomit
Jethani: Yes everything was fine, yes everything was fine to see (everything was fine as long as there was)
Jethani: Yes, yes there is satisfai (all the doctors who were there saw it well, you have satisfai from them)
(asked about cost in S Hospital.)
Jethani: No - no expenses and water did not happen.
Jethani: Those who used to write medicines from outside, used to bring the same medicine, no medicine was written from outside. Only one medicine was written, we had brought 40 rupees from outside, the rest and those who wrote it were discharged. It was time that he came here from his medical store.
5. Could you please describe your experience about communication by the various hospital staffs during hospital stay? (Probe:who all communicated, who was the primary communicator, nurses, doctors, other staffs, frequency of communication, mode of communication, details, completeness of information, skill of communicator, consistency in communication)
Grand Mother: Did not tell (did you tell the doctor that this is the problem)
Jethani: They did not say anything like that, we had to go and speak, then we used to speak and shouted a lot,
Mother: used to shout
Jethani: There used to be a tension that we have such a case of Didi, do not know what will happen - what will not happen, something Go speak, you are more worried, we are not worried, we are watching, we will do according to our own, 20 20 days Till, nothing will happen for 15 days, the child has died in the stomach of the patient, which we have to do according to ourselves I will not do anything as you say, I said ok you do it on your own but we should be relaxed If you want, then he said that we are doing according to our right but you go and sit near your patient.
Grand Mother: Do not come again and again, sit quietly, I said come on, you don't mind.

Grand Mother: No, that is not all, liked daughter, life was saved we liked it very much, from S Hospital There is no heavy work, when it is not done anywhere, they tear it apart. Well done us Is it that we should not think that there is no tearing, how was it not settled, there is nothing in one half, shall we say God makes us do it.
Grand Mother: Children will happen again, they are not saying wrong, what if they do not have life From whom So we have no problem with anyone, even if she tells us anything, we would listen, we should There is nothing, meaning life is saved, eat and drink, be happy, be comfortable, then God will give, he is crying He is laughable, what is in our hands, Today we have made you laugh, the same is our fault and there is no fault of God, he sees the world and sees us as well, so there is no meaning in this, The doctor was all right in his place, when we go again and again, you work, then definitely say let's sit You will not see the patient again and again, Will say no, and nothing says that let's be patient soon Sit down, how did he save his life, no one used to say that take away from here was not a matter, on the third day Said, collect two blood, first collected blood.
Jethani: The blood was already deposited before then
Grand Mother: Our boy did not come out, for his friend, yes, he gave his blood, we said okay Have asked for blood, blood will be given to the body then we have no problem with this son, meaning If you are aware, then God will give us, then we will fulfill our hobbies, and the girl is our three first-born. Nobody is a girl, they do not even have a girl, and it is so sad that they cry too Cries we did not say in luck and we were very happy that the girl was born, But when the top is not happy So where will we get happiness, I understand this, do not take tension again and again, you have seen the world of feeding Goes on, we have not even been born, do not stress this thing, we only understand them, Do not take tension at all, eat and drink if you are in your body, now they will cry again and then they will laugh, that's why not tension Take it you will tension your body and your eyes will go away. When someone calls, we said no to this. I think don't cry yet, you stay with your body, eat and drink and stay, then God is up We will give you, we do not have your emphasis in it, what they do will happen, we will accept them, I just understand that Don't take tension at all tension will reduce your mind.
Mother-crying after listening to grandmother.
6. Could you please tell us about the declaration of the event/outcome (stillbirth) (Probe:who declared the outcome, cause explained, the body language and expression, preceding events before delivery, meeting with any senior doctor/member, attitude/body language/expression/skill of the communicator, expression of empathy/sympathy, language and simplicity, perceived completeness of information)
Jethani: He only spoke during the ultrasound that he had asked everything (that baby was upset in the stomach It is done).
Jethani: They had written that go get ultrasound done immediately. There was also Ultrasound and ultrasound also happened here (in S Hospital and VB) Mother: When he went there, neither did he show up in the emergency, went to S Hospital from there. Ultrasound done
Jethani: Yes-yes (asked if they went to VB and then S Hospital)
Jethani: Yes, he referred to S Hospital from there
Jethani: Yes, there was an ultrasound too (S Hospital)
Jethani: No-no from here (asked if in VB, ultrasound reports showed that baby was live) that is why he had referred there, he (VB) had told that the child was spoiled and Now we cannot do anything here, you have to go to S Hospital with them, then we took S Hospital Immediately went away from there and then did not come back.
Jethani: Yes, yes, Sarah said, the doctor said that you have been delivered, their patient, baby You are finished, after a while we will give it to your child, after this we will let you see our patient, then We were asked to bring clothes, took them away, showed them to the child, got them dressed, then after that the child took the clothes Wrapped in cloth.
Grand Mother: Behavior is fine, behavior is good for everyone.
Jethani: The behavior was right, by the way, the behavior was right as they told us, someone's behavior was bad. Did not feel

Grand Mother: No - no feeling nothing (about the behaviour), this is what we had when the child went Mother was saved, all her behavior was correct, that we left our life saved, we all felt right Behavior, we do not hate business, which has happened. (after sometime) everything is fine
Jethani: everything was fine
Jethani: There were no big doctors, there were small doctors, the big doctor used to come in the morning then we would not have been there at that time. Used to exclude us
Grand Mother: No, not the big doctors, those who were small, we said let's come to the hospital and say something Do not bring it to mind (Who said big doctor or small doctor)
Mother: When the doctor came, neither did she say that son baby is not right for you, there will be a problem No, don't panic, it will happen.
Jethani: used to comfort, yes, talk to love
Grand Mother: Yes used to talk with love
Mother: No, knew (asked who said after delivery that the baby is finished)
Jethani: He already knew
Mother: Yes, it was shown right away, but it was immediately shown that your child is already dead and Look at what the girl is, the girl is the boy;
Jethani: He had shown it immediately, got it, showed it to us and then showed it to us.
Jethani: No, the reason was not given (what is the reason the child has died) Mother- No Grand Mother: No, this is not even a tail but no wisdom angel did not ask. Jethani: There was no tail. Grand Mother: No, not even us, not educated
Jethani: We also got the news of Didi from VB here, so what was the bid, we said that If you had traveled, then you must have been shocked due to the journey, then bid you first here Had to show, you shouldn't have done that, but then it will happen, then show beforehand, so why Happened and we will treat them accordingly from the beginning
Mother: That quote is because of the journey, you didn't have to travel
Jethani: Because of what has happened, no one has spoken, did not even speak here and did not speak there either. This jerk- Who got registered at the hospital there, they asked us what happened to you We said that this is how it happened, bid not to slip, show me what happened, show the report, I said like this, then why did the doctor say this, the doctor said? So I said nothing like this, the doctor only came to visit Were, that's why no one told what happened, neither told here, nor told there, nobody else Told.
7. What was your and your family member’s reaction when you were told about the stillbirth? (Probe: what was the reaction of mother, father, other family members who were present, did someone try to console you or your family member)
Jethani: We were, their husbands, and together they let the ladies stay, we were the same and in the day Mother used to live.
Jethani: We already knew that the baby has died Didi was there when the post-mortem took place. Before admitting, we had told us who you are you know what has happened to them, Their baby was finished in the stomach they had already given all the information, their child is over, You know all the signatures were done all this information was already there during the treatment. Said nothing.
Jethani: What to feel in this, Didi, he said that S Hospital is carrying on, told here that the beat It is closed, maybe if you do it in S Hospital, then maybe something happens if the heartbeat is still a little, Went there, Didi was not able to understand us at that time, what to do and what not to do then we Ultrasound told that the baby is over.
Jethani: That doctor had given them talk here, she was crying and what was her nature, she was crying, she was also crying He was giving the paper at the time when he got this news, had to fill the railway recruitment form The day was his paper.
Mother- crying
Grand Mother: He was very sad, had gone to deliver the paper that day, yes he had come by evening.

8. How was your experience about the procedure after death till handover of the body to you? (Probe: processes asked to follow, documentation and papers given, interaction and behaviour of the staffs, respect for religious norms, time taken, cost/payment, assistance from the staffs)
Jethani: No, nothing had happened (asked if the signature on any paper), no, there was nothing like this
So before getting all the signature admissions done, your child was finished, you got all this
It is known about the thing, before conducting the check, we had got all our signatures done.
Jethani: There was 10 minutes left at 2am when the child was born and the child got me at half past six in the morning I got a baby at 6:30. No, he himself came and said that whatever is your patient is right and your the baby is born, the child who is finished is gone, now you will get to know how your child is
What is the whole thing then I showed the child at 5 o'clock that now you go get the clothes and come at 6:30 then we will give you the baby, after giving the baby then you will give it to your patient, then we will give the cloth at 6:30.
When we were taken we gave the child, after giving the child, we came down with the child, after coming down we gave the brother-in-law gave the child, those people took away, everyone took them away
Grand Mother: Where everything is done, the cremation grounds were taken to Dakshinpuri
Jethani: Dakshinpuri, taken to the crematorium, was probably taken, then we went upstairs to the hospital and gave it to him He brought them out of the labor room, brought them back to another room after 2-3 hours.
Grand Mother: Yes, immediately taken from here to there
Jethani: We did not bring them here, they had gone with them, we have taken them to the hospital, then this from there till evening the mother and our husband took the goods which were to be taken with them.
Grand Mother: No, baby, we have not done anything before, we said that our lives somehow
First of all, then we will think what to do, we said the same doctor how can we save his life by saying, Lage wants what should I rip? We said no, I don't want to, (Have you prepared for action Initiated)
Grand Mother: No, we did not (asked someone to bury the child)
Jethani: No, there was no such preparation (asked whether there was any preparation to bury the child)
Grand Mother: When we got a call, then we said, this is such a thing, you guys go whatever happens
At home, by calling from home, she said cloth, so we said that take the cloth one meter, then took the cloth Means both brothers and one who remain behind, the brother-in-law came with everyone, then he came at 8 pm on duty time. After doing everything, he came to duty again.
Grand Mother: No, we did not (asked someone to bury the child)
Jethani: No, there was no such preparation (asked whether there was any preparation to bury the child)
Grand Mother: Yes, come back here and then left again. And the boys were both the day she was in the hospital, The brother-in-law and his two brothers used to stay there, they used to stay there day and night, and the owners used to come like this in the evening Used to come here and come here and bring us, so it used to stay in the night, so much time was spent from there Used to come, bathed and eaten, then it used to come, then I used to stay in the day again in the evening, meaning 2 o'clock, 3 o'clock, 4 It used to go from here at every hour. So we used to go at night, it used to stay at night and day. I used to live, I was not educated, I used to go to get water myself, if I forgot the room, I came to ask someone, So it said mother, you should not stay up at night, you are not educated and if you have a chance at night, I went
I will go, where you will go, so I said okay so I used to live in the day and take it and it would stay overnight If I was a goddess in the morning, she did not cook for her boys,
Used to take school again, all business It used to be that I would get that food again in the morning, give lunch to everyone, and leave the boys to school and tell the other that my The boys used to get away, I would have reached there at 8-9 pm, used to do this and what should I do now We did not have what was possible in our hands, we did it in a round, now there is sorrow, sorrow is still there If the cry cries the heart bursts, but it is our duty to explain, is it done by the mother, the father did, the brother did I did it, my aunt did it, so I say, don't cry, daughter, this (head) will be stressed, The skull will weary, Do not drop your eyes, see all of them, two are fine; God will also give you, He is the one crying, He is the one who laughs, You don't do anything, we just got here that life is saved, I just understand here and what to explain.

9. Who all from your family and community/neighbourhood were present at the hospital and how they supported you during this period? (Probe: who informed them, who all came, what type support given)

Grand Mother: No, there was no one (someone from nearby was also in the hospital)
Jethani: The housemates were
Grand Mother: Yes, everyone has come, had also gone to the hospital, everyone had come to know who was there the ones also came to the house)
Jethani: Yes, yes, I came to the house, and there was also a hospital to see, when I brought them here at home everyone came here too.
Grand Mother: What did they suggest.
Jethani: What did he suggest, what did he say that what had to happen now has now served him well Does it so that it is correct, as much as possible to maintain it, all the family members do what was not yours you did not get it explained.
Grand Mother: Help, Look-Explain
Grand Mother: No, it is all said that you had brought it, let you stay in one place (to keep daughter in law in village) and did not say anything, do you know what happened? It had come on the sheet, had brought it from Anand Vihar from there on a scooter, brother, what can we know now, just This is what everyone says, that means you are shocked, this is what you say and you don't say anything.
Jethani: Everyone says that it can happen because of movement
Grand Mother: Yes, because of commuting, so we said bring them and go, so let us know now
No, now let's go to the top, what happened, what happened.
Jethani: When we were the first child to grow up, when we were in the stomach for the fifth month, then we also went to the village. Were, when the sixth month was going on, we had come back, we too have grown up in S Hospital, but we There was no such problem, so there would be such a problem, they did not feel like we were not aware. That it will happen to them due to the movement and otherwise, they would not have brought them from there.
Grand Mother: And then baby, there were no men there, who would run with them, thinking that everyone was here, so Who used to run, where would I have been taken, this is why we said that there is a hospital there, all the facilities are there, Will run and what will I do, if there is a buffalo cow in the village, then I would continue to work in it, I told them I used to run, thinking that I said that I will bring you there, I had brought with me, Boy too I went, brother, if you want to let it stay, then you too stay for a month or else keep it there. Government facility is there, I said let's take it there brother In the same circle, that's why we brought them here, And both of us were there in our stomach, we are saying that there was not much water in Sangam Vihar, where Where did we get the stuff after filling the bucket, now it is no longer freezing, now do not get the dough soon, Doctors do not allow us to work hard, we do not even let them do that you knead dough, bread Get it done, whatever you can do, we will all do it ourselves, we are going to do it, it makes bread in the morning. (elder daughter in law), we have nothing that she is not doing it, she is not doing it, she would have done everything comfortably It is not a matter of us, when they got married there was no home, there was so much pothole when here, in that One day the doctor came to inject and said that you are not getting the house built, now you have got many sons, plenty This pothole was deep, but the lettering remained in it, when he was in his belly (elder daughter in law pregnant) I used to take it to the forest at night, then made this lettering, then made gold here, Not long, three- It has been four years, meaning it should be made, it has to suffer a lot but nothing happened to them (elder daughter in law delivered safely), then it is the same thing that in the forest, there are children here and there, then they see someone If a man does not see, then whatever is written in his destiny, he will get it, something must have been written in luck, not You have written so much pain in so many days, and no one wants our family to do anything, same day and night I ask that there should be three people, God forbid anyone, everyone should be comfortable in the house
Jethani: There are also caretakers here. If something happens, take it here immediately, that everybody here Together we will take care.

10. What all rituals and procedures did you follow after stillbirth? (Probe:body preparation, who prepared, burial/ cremation, other rituals and timings, who performed them according to the rituals)
Jethani: No - no, do not burn the child (asked whether you buried or burnt the child)
Jethani: He was taken immediately after being brought from the hospital, he immediately adopted himself, within 1 hour at most he all work (buried within how many hours)
Grand Mother: We burn only married people, do not burn them
Grand Mother: Yes, they do it all (asked if performed Pooja by calling Pandit-Vandit)
Grand Mother: Yes, we got it done, which was done, now it was in the small stomach, then it would have been bigger Without marrying, they do nothing else, they will do whatever is going on here, they will do it all with a tail from the priest.
Grand Mother: Yes, with this same pundit of the tail, they tell us that this is a house, do it, do it, do it. Pundits talk about it, and they live there a lot, how is luck, happiness is sad, there they go in misery
They do not go in happiness, but there are also pundits who do their own worship, which has to be done where taken were, they give what is Dakshina-Vakshina.
11. How did you, your spouse (husband/wife) and immediate family members cope with the difficult phase? (Probe: any change in the psychological state, physical state, engagement in daily work, spiritual/religious activities)
Mother: mam, so sad that you don't know how mam, nothing is understood, so sad Had spent so many days and suffered so much (crying), I then went there and showed them like this It is done, come by ultrasound, I went there to get it done immediately, then I brought the nurse here. He said that son baby is not right for you, I cannot understand anything, So I told the big doctor if I said Son, we cannot do anything, your child has become like this, now you do not do this from the front, do not travel Do not stay in one place, do not come and go anywhere and show from the beginning, do not carelessly Said ok mam
12. Is/was there any change in the interpersonal relationships between you and your spouse and other family members after the event? (probe: relation between husband and wife, with other family members)
Mother: No-no nothing (asked change in inter-personal relation with spouse). no it's fine,
Well, yes it is not the same as before, but right now they do nothing, meaning they do not make a relationship like that. Right now it is said that you get well, just after that will do 8-9 months, after one year, in it nothing will do. You get well your tension you get well
Mother: No mam, no problem, nothing says mother-in-law says, stay fine whatever you do and
Do not take eat-po tension, it is said, no mam, nothing is said from the beginning, only then do not say anything they say, do not work too much, do not do anything that will happen, we will do the work.
Eat-po bus, there is no tension, but I wonder throughout the day how I don't know (crying).
Mother: Nothing else, from the very beginning the child was perfectly fine, moving at the right time, but here Trouble has come since traveling for two-three days, calling from here and staying next day
If shown, this is the reason why this has happened, it will be okay, then on the second day it started to pan, then it said It will be alright, then said that we have to go on Wednesday and did not know that this will happen and Had it been known, we would have shown it in the emergency at night.
Then on Wednesday, he showed that the child is not right, Go do an ultrasound in emergency, when you come there, the ultrasound people also said that your The child is not well, it has deteriorated, there is no problem from home, mam, in eating and drinking.
15. Do you think and/or discuss with your spouse/ family member(s) about the loss and related events and blame someone/something or feel guilty for the same? (Probe: with whom; how frequently; the context of discussion; contents of discussion; whom do you/others blame for the death or what about you feel guilty)
Grand Mother: We do not hold anyone responsible, we will not tell anyone, that nobody did it,
Even if you want to stay till evening, when you feel like doing it, we don't need us, just a bread and a dhoti if necessary, do eat and stay for yourself.
Mother- no mam (crying).

Grand Mother: Will not be able to talk, just everyone is happy, we are also happy, we will in five-six months anyway She came, had been home for five-six months, she is a buffalo, a cow, a mother-in-law, a prostitute, a devrani is here, we are Five-six had already come, they had brought them, otherwise we have a lot of work at home, we have farming and fencing. She lives

17. How your and your family's life has changed after the event? (Probe: what has changed, what is the major change, what do you care more now than before; plans for future pregnancy, any substance abuse- new behaviour or increase in the existing behaviour)

Grand Mother: The problem is not from anyone, baby, we are running a little medicine, I tension is there, all of them remain ill, due to this, all of them become tensed as to what God has become in our house

In, this is the tension I get and nothing else (how much has changed in your life after)

Grand Mother: No, there is no change.

Grand Mother: It is going on like that, everyone is just happy.

Grand Mother: This (mother) has to be named from the beginning (plans for next pregnancy), speaking incorrectly And to be checked on the month or ten days, this is what we need to get done, one place to stay, another place Don't know, this is our plan, just like here, stay here and stay there, stay there, this is our plan Stay in place, ok, maybe something has happened in the coming and going. Now this is what our law says, know what happened this is the only thing that has come from here and there is nothing else.

Mother- not well, vomiting

Grand Mother: Their grief went away there, every three months of a month there, when they saw I felt nothing but his grief then, I said look, the whole month is gone, seeing the sorrow of the miserable The grief comes, when I have seen all of them, then I said that nothing has arisen before, when two- If the mother of two or three boys becomes her, then the one is going to do the above, So look at their sorrow and see their sorrow Done us, I said tell them it was a full day, it happened on three lives, that means next, so baby I was saddened to see them, see that they are the mother of two-three-three boys.

18. What was the cause of stillbirth mentioned to you and what is your perception about the value of knowing the cause? (Probe: knowledge about the cause of stillbirth, willingness to know the detailed cause, potential factors that led to the illness/death, potential impact on the other family members or next pregnancy)

Grand Mother: Why do not you want to know if we have anything, consult the doctor first

Grand Mother: Yes, you should know, you should check, yes what happened

Jethani: No reasons not mentioned

Jethani: Yes, we should tell, yes we want to know then their next time should be monitored accordingly. Caste

Jethani: So didi know how the death happened in the stomach? For example, the child knows us by postmortem Moves

Jethani: If we knew about it, Didi, then the next time treatment would have been done accordingly.

19. In your view, what more could have been done to know the exact cause of stillbirthand the reasons causing it? (Probe:investigation, autopsy, any other)

Jethani: No, there is no information about it.

20. Are you aware of any method for identifying the exact cause stillbirth? (Probe: autopsy, investigations, etc.)

Jethani: Yes

21. What are your views about the autopsy/ post-mortem for identifying the cause of stillbirth? (Probe: necessity, religious aspect, time needed, disfigurement, etc.)

Jethani: Find out but not get the post-mortem done

Jethani: No, no doctor told us that for such information

Grand Mother: No, they told us to come and check on Monday within eight days

Jethani: She (mother) for her check up.

Jethani: No, there is no other way. How about a small child, I do not think of such a small child

Get the post-mortem done.

Grand Mother: We do not understand daughter

Grand Mother: Now remove what happened to the daughter, we will not get it done, now it should be safe, further If you get some information, if there is a chance, now what was to be done, now what should be done post-mortem, now it is our fault that We brought here, we brought here, maybe we were shocked, now it will not return to us, we will Do anything, if you do not return, then what is there to do, leave nothing to it, now think of them Tell me how their blood will be formed, how their body will be cured, we have to think that this Feed - feed them, make them healthy, explain to them what to do, and nothing to do, daughter Which will happen later, I will come back to you again and I will inform you, yes, I will check it again, as soon as in S Hospital His writings will be written by him, this should be done

22. What are your views about collection of the tissue (like done for biopsy to diagnose diseases) and body fluid samples for identifying the cause of stillbirth? (Probe: necessity, religious aspect, time needed, disfigurement, etc.)

Grand Mother: Yes, we will gladly take that thing

Jethani: For that matter, we will do yes

23. How parents and family members can be approached for the tissue and fluid sampling? (Probe: who should approach, whom, when and how should approach; what should be told and in what detail; should some picture of the method be used to explain)

Jethani: Watching the video

24. In your view, what factors are important for acceptance of the tissue sampling by the parents and family members? (Probe:desire to know the cause, trust, confidence on the health care provider)

Jethani: No one should explain, anyone should understand, understand, but now how will you do

Jethani: Our father will have to talk to them (whom)

Jthani: Doctor (who), yes,

25. In routine day to day operation who takes decision about the major activities (purchases, treatment, spending money) about the family? (Probe: involvement of the mother/women in the family, any other family member, consultation with any other member)

Jethani: Father, father-in-law

Jethani: Whatever they say, Papa believes, they ask for

Grand Mother: They see everything reduced or increased, Papa sees everything, they see less and less, whatever they see Have you bring them to yourself, when do you bring boys, when you have to bring lani, you bring boys Like we have brought some information, now something has happened, boys come on their own

26. During the hospital stay who discussed with the doctors/ nurses to make the decision(s) about treatment and other procedures. (Probe: father, mother, any other family member, did you/your spouse talk to someone in your family like elders or relatives to take critical decisions)

Jethani: They used to talk, talk to them (with mother)

Grand Mother: Nothing was spoken to us (with mother)

Jethani: Nothing used to talk to us, any doctor used to come and immediately remove the relative, these Used to talk to

Grand Mother: No No No Complaint

Mother: No, no, no complaint

Jethani: Nothing like this (asked if mother discussed with family about taking decisions on the treatment)

Grand Mother: They were saying that if they mean normal then it will become normal or else they will be mean If you want to leave then there will be no child then you will see again

Jethani: They were saying that this is their first child, it would be better if they were normal.

27. After the delivery, who decided about the burial and other post-death activities and rituals? (Probe: father, mother, any other family member; did you/your spouse talk to someone in your family like elders or relatives to take critical decisions)

Jethani: Our father, got a call that he said that we are coming from somewhere and then we are coming here From whom everyone wanted to go, they were taken by father.

In-depth Interviews of parents who had Stillbirth-16

**1.1**	**Primary respondent (relation with the child)**	Mother
**1.2**	**Age of the primary respondent**	Mother-35 years<br>(father-40 years)
**1.3**	**Other member present**	Sister in law (jethani)

Please let us know the course of pregnancy and events around the delivery/ termination.
(Please document as the event were told by the respondent)
Mother: He had stopped walking, he was not moving in his stomach. In the entire 9 months, it means two to four days. Gone (asked what month are these)
Mother: Closed a day ago, then we went here to show private, from there, we checked it and they said that The child is weak, has low heartbeat, then we have come from there given medicine, lie down for three hours, it will last, come did not eat medicine and did not rotate at all, then we went to the hospital in the evening. MN, yes, when they went there People sent there (S Hospital).
When he went there, he told that the child is over you, you go home, took admission, but said that go home you will stay here overnight, but you will not do anything, go home and rest tomorrow.
Come at 10 o'clock, then we will give medicine - then we don't understand we have left then people start speaking You will admit and give out, then tomorrow you will have trouble, then you will run here for two-three hours, then if you go in They said, sit, sit on the jacks, sit here and do nothing all night, stay up all night, stay in the day and take medicines in the morning I gave them the medicine, then I gave medicine in the afternoon, then I ate it, then my pain started, it started hurting. The child was born until 7:30. Yes, Normal (asked Normal).
2.1 Can you please let us know about the problems, illness and events immediately before the delivery?
Mother: (asked where she went for antenatal care) In MN, right from the beginning, everything, yes.
(asked about cost) No, nothing happened.
(asked if there was any problems, illness before the delivery)
There was no kind of weakness, no problem.
(asked if all tests, reports were normal)
Everything was good, yes everything was good, we had ultrasound twice, we got it done they took place in 7 or 8 (month).Yes, all came well. He wrote that get it done wherever you want, If it was given by the government itself, it was done in Majidiya, everything came right. Don't know what happened suddenly when I woke up, I realized that they are not moving, so I immediately called and showed it to the midwife, He also said that everything is fine, he will never roam a little, he has given out then went to the doctor, he said the children are a little weak, so they are running a little, will work. Yes, I went somewhere (private clinic), this big Are on the market side, yes they are private all the delivery-delivery doctors do it.
3. Now we would request you to tell us something about the period of hospitalisation for the pregnancy, delivery or termination? (Probe: hospital, ward, duration of stay, course of illness)
Mother: (hospital, duration of stay) Stayed at S Hospital for two days and was discharged for the third day, from there on Sunday we had come He checked up (MN) and said that your child is not getting beat Go to S Hospital then we went there. Yes, two days stay, went on Friday, it's Saturday at 7, then we were discharged at 8 pm on Sunday
4. Could you please describe your overall experience about the hospital care? (Probe: investigations, treatment given, involvement/ participation in decision making, satisfaction level, quality of care, cost of care)
Mother: (cost of care) No expenses were incurred (S Hospital)
5. Could you please describe your experience about communication by the various hospital staffs during hospital stay? (Probe:who all communicated, who was the primary communicator, nurses,

doctors, other staffs, frequency of communication, mode of communication, details, completeness of information, skill of communicator, consistency in communication)

Mother: Behavior is not right, people do not see it quickly, and as if you are having problems, call them on the street Runs, I was in trouble, my pain was getting too much, I was not getting hurt, when I was calling, abusing me Shit, go from here, let's talk.

Bhabhi: Batmiji, he says when I have seen once, why do you come again and again to speak

You have not produced children, which you are in trouble, now go let it be pain, so I used to say that their stomach the child is finished, if something like this happens, then who will be responsible, then they are telling the case of 15 days Even we manage, no poison will spread from it, you go and sit comfortably, we are not the same patient that we are a Look at the same, then I went again that such things have happened, see the pain is increasing more lately, So how long did you say I have been saying pain, I have been coming for 5-10 minutes. Pain, then go and sit comfortably, when my time is up, we will I will do it myself, when not everyone listened, I brought it from another place, which produces children, then I come from another department She had brought it up, that didi little, let's say Samson Nisha (name of mother) that things are getting worse, If she is becoming unconscious, then I am not of this department, I am someone else, so I said anything but empty See this, come with me so much that the doctors go a little and tell that the Bo girl is upset, the doctors are not agreeing She is saying to me that you are lying, by the time we brought her child was already in bed, when Ran away with it Yes, she was a nurse, when she came, when it was done, then she came from the race.

Mother: When the child did not come to the mouth, then those people came, they had brought them and let us see, the child was born

Bhabhi: No (asked HCP came after the sudden delivery), I had brought it, brought the nurse The child who produces a baby, Didi, let's go with me to see what is happening to the patient, and So just tell the doctor that yes, we are lying that we are telling the truth. So bid (supporting staff) I I am not from this department, I am from there, I said wherever you are, look at the empty patient, tell me this much, When she came, she said that baby has arrived, baby has arrived, when she came running doctor, bring the file and bring it soon.

……… to take it, take it fast, then the ladies started saying that they should not be here, if not the room Will be spoiled (obs doctor), when I said why should you come now, I should not come now, now I am in other department It will take care of calling from you, when you bid me bring the file, then turned us around, till Gone, there is negligence in it. She was a senior doctor (obs doctor), she did not talk much, she came check up after leaving the bus.

Bhabhi: We were outside, we were not inside, when she came inside to check all of us we were out removed.

Mother: Those people were saying that you have come by yourself you have not sent them to the hospital, you yourself I have come, I said they have sent us, I have not come myself, I have shown the paper, saying that this paper there No, this paper was in the same, saying that, no, they are not paper, you are telling a lie, one is saying that you are till you do not give blood, you will not do anything. You remain seated like this, but you also took blood but did not offer it.

(frequency of communication)

Mother: Telling that when the child started coming, then shout, then he took me on taking it, the child was born.

Bhabhi: The night nurse was right, she was right to think, but the day shift is right No, they are a little tough.

6. Could you please tell us about the declaration of the event/outcome (stillbirth)? (Probe: who declared the outcome, cause explained, the body language and expression, preceding events before delivery, meeting with any senior doctor/member, attitude/body language/expression/skill of the communicator, expression of empathy/sympathy, language and simplicity, perceived completeness of information)

Mother: That quote (MN) is not getting beat, baby's go there soon (for ultrasound). It was said that the child is not getting your heartbeat.

Bhabhi: Nobody said that
Mother: Yes, meaning they said that if the child is not getting a beat and the machine is installed, then the machine also told So he said that go there, get ultrasound done soon, if you get ultrasound you will find out, sometimes The machine also tells something wrong, there will be ultrasound, then we will know if we have children or not, then we will Went there
Bhabhi: When it was done (baby delivered), the nurse was (who declared the outcome). Told me to come and said What will you do for the child, the child is over you, the nurse came first and told me that you should not worry gone Samson has been done, so I bid what has happened, then I will not tell you that they will come from inside. We said ok I was standing at the door, then she came one of the ladies said, "Nurse, baby is done, I said what happened, then Will you see the bid, I will see the bid yes, then she brought it to the basket, look at the bid then I see that she is a boy, she said Baby, I said, yes, we will have a child, so if you bid or throw it somewhere, why should we throw our blood, I Why will I throw the child then you are right to take it at night, then we suggested that we take a small child at night Where we will sit, we will take it in the morning, then gave it to us at 5 in the morning.
Mother: Did not tell me (declaration of death), when I went inside after ultrasound, they spoke had given, the doctor (S Hospital) had told that your child is finished (who declared). Said that your child is not in ultrasound, now you are finished in your stomach.
(asked attitude / body language / expression / skill of the communicator)
Yes, it did not matter to me comfortably told.
7. What was your and your family member's reaction when you were told about the stillbirth? (Probe: what was the reaction of mother, father, other family members who were present, did someone try to console you or your family member)
Bhabhi: How will it feel, if the lamp of the house goes, how will it feel if a man lives in his hands and feet If a man cries, he cries, but now he was not going to meet, yes he would slap it there, We have not forgotten that child yet, she is deteriorating the condition of her mother, do you Leaving all business, I sit here, keep thinking and keep getting extinguished.
8. How was your experience about the procedure after death till handover of the body to you? (Probe:processes asked to follow, documentation and papers given, interaction and behaviour of the staffs, respect for religious norms, time taken, cost/payment, assistance from the staffs)
Bhabhi: Then he took it in, said that he brought the cloth? I said yes, I brought a towel, so bid me bring the towel again She took a towel, then packed it away and then gave it to me in the morning. I brought it home, then home But brought her, bathed her, washed her again, took her to the cemetery, she was given mud-witty. Bhabhi: We People burr on the hill.
Mother: (documentation and papers given) Medicine was given.
Bhabhi: Not done.
Mother: No (papers given at burial site)
9. Who all from your family and community/neighbourhood were present at the hospital and how they supported you during this period? (Probe: who informed them, who all came, what type support given)
Mother: Sister-in-law and one more were our brother-in-law and our man. She lives a little far away (Devrani), Shahdara She lives. He (husband) was outside, where did he let go inside.
Bhabhi: They were not allowed to go inside. We were both men, their ignorance was again on fire (inside the ward), night we remained the same for the entire time, in the morning their devotion came, so both of us were taking care.
Mother: Sahara met these people (dewrani and bhabi), Sahara met these people, whatever they had to do These people did it dare to see that they are with us.
10. What all rituals and procedures did you follow after stillbirth? (Probe:body preparation, who prepared, burial/ cremation, other rituals and timings, who performed them according to the rituals)
Bhabhi: No (asked if Quran was recited), we cannot do that for this child because in the world He would not have opened his eyes, he would have made us do everything, we had talked to Hafiz ji, he said this

It is not fair, if the person, who opened his eyes in the world everything would have happened, would have to do it, would have mixed it, Happens, nothing like this, there is no prayer for Janaaja, by the way, the man went, 20-25 people went to the burial. (timing) When we left the hospital from there, it had come at 6 o'clock; they had given it at 5 o'clock, so we hurried three. Wheeler came home at 6 o'clock. By 10 o'clock it was all burial time and his brothers had come a little It was not late, otherwise the work of our people was done soon, now we will come, only then we will take it, it was from their home. (religious taboo) The woman does not go. No, only the gents go.

11. How did you, your spouse (husband/wife) and immediate family members cope with the difficult phase? (Probe: any change in the psychological state, physical state, engagement in daily work, spiritual/religious activities)

Mother: Where have I emerged now, (any change in the psychological state) in my mind, he used to roam the same Is, there is pain in the head, (engagement in daily work), children do work.

12. Is/was there any change in the interpersonal relationships between you and your spouse and other family members after the event? (probe: relation between husband and wife, with other family members)

Mother: Yes, still understand, they are taking more care than that, they come and keep looking at my mouth, whatever Neither do you have anything, if you have pain in your head, you just press it right.

13. How did the family members support you and your spouse (husband/wife) during the difficult phase? (Probe: burial rituals, community norms/rituals, coping with the phase, financial)

Mother-support is available (family members support), come and sit for two-three hours, it feels good
It is until these people live, there is no realization and if they do not live then there is a feeling, I remain roti all day.

14. What were the reactions/ counselling and suggestions given by the relatives/neighbours/ community after the episode? (Probe:what to do after death, following the rituals, knowing the cause of death, any other)

Mother: I was in the hospital I do not know (support from neighbor)
Bhabhi: There is support from neighbors, without support there is nothing, now the husband is on duty, no matter so the neighbor will come to the race, he will take care. Now all the men took the mill (burial site) met. (Financial support) There was no such problem, everything is from the above. Now this is a husband Will tell (expenses at burial site), we do not even know people there.

15. Do you think and/or discuss with your spouse/ family member(s) about the loss and related events and blame someone/something or feel guilty for the same? (Probe: with whom; how frequently; the context of discussion; contents of discussion; whom do you/others blame for the death or what about you feel guilty)

Mother: No (discussion with husband), he is sad, and will remain, he speaks.
Mother: Now who to blame, now who should we blame, what will we do to blame someone
Bhabhi: Now who will be blamed, will only blame Naseeb that was not in our destiny, God gave and Took, who shall I tell, we cannot even say to anyone, from the beginning to the end, so much care was also provided, What did not happen in these three children, it happened, that let's be the child of the last

16. When did father and mother (if working) returned to work after the event? (Probe: father, mother, loss of job/ resignation from job/change of job, was there any change in the mode of functioning)

Mother: Went there after two-three days (father), (there was any change in the mode of functioning)
No, they do not say anything, they come and sit quietly.

17. How your and your family's life has changed after the event? (Probe: what has changed, what is the major change, what do you care more now than before; plans for future pregnancy, any substance abuse- new behaviour or increase in the existing behaviour)

Mother: (plans for future pregnancy) do not do children now, if it had come by mistake, I did not do medicine Ditch, had eaten medicine before, then there was a lot of trouble, meaning the blood had started urinating, there was a problem. In cleaning, then it has come now, then they said eat the medicine, I said I will not eat the medicine, whatever happens Will go, I will not eat medicine, then the same problem will

come, because of this I did not take medicine, so keep it Were taken Yes, what is happening should not happen (for other pregnant women losing their newborn), my Whatever was meant to be done, and others should not happen.

18. What was the cause of stillbirth mentioned to you and what is your perception about the value of knowing the cause? (Probe: knowledge about the cause of stillbirth, willingness to know the detailed cause, potential factors that led to the illness/death, potential impact on the other family members or next pregnancy)

Bhabhi: no (cause mentioned)

Mother: No-no (asked if she asked the cause to doctors), Mother: what will we do now

Bhabhi: (perception about knowing the cause) Now what would we do by tail, my boy got (dead)
Child) I came and took them, they were also discharged at 8 o'clock in the night. Are, the cause of death to Doctors.

19. In your view, what more could have been done to know the exact cause of stillbirth and the reasons causing it? (Probe: investigation, autopsy, any other)

Mother: No investigation

Bhabhi: (investigation) we do not know all this, we have three children all three were in the house I have gone once. No, no ultrasound was done, no x-ray was done, nothing was in the stomach, happened in the house, I did not have any problem, So we do not even know all this.

20. Are you aware of any method for identifying the exact cause stillbirth? (Probe: autopsy, investigations, etc.)

21. What are your views about the autopsy/ post-mortem for identifying the cause of stillbirth? (Probe: necessity, religious aspect, time needed, disfigurement, etc.)

Mother: We do not speak (disagree for post mortem), we did not have the courage, I did not have the courage inside.

Mother: discuss about it

Mother: When the baby is exhausted in our stomach, what is the benefit of getting it squashed, the child is a little bit It is good if you tear it apart, it will not like it.

22. What are your views about collection of the tissue (like done for biopsy to diagnose diseases) and body fluid samples for identifying the cause of stillbirth? (Probe:necessity, religious aspect, time needed, disfigurement, etc.)

Mother: Even then we do not disagree (MITS), now it is not even the child, so what will we do to know him? It is over, what happened, how it happened, there is wind, there is some air in the stomach, it can happen.

23. How parents and family members can be approached for the tissue and fluid sampling? (Probe: who should approach, whom, when and how should approach; what should be told and in what detail; should some picture of the method be used to explain)

(who is the elder member whith whom husband takes suggestions on treatment) Bhabhi: now this
We will know all the things, so that the heart will suit them, they will take advice from them, now not all humans have Will go to tell your heart, nowadays you know how it is

Mother: Whom to consult, if there is any such work, then we advise (husband-wife), and we do not
So tell from brother.

Mother: (What should be told and in what detail) No, we understand this, but will not get it done (MITS), What will we do, and we will feel sad.

24. In your view, what factors are important for acceptance of the tissue sampling by the parents and family members? (Probe: desire to know the cause, trust, confidence on the health care provider)

Mother: What will parents benefit from this, (desire to know the cause) No, I don't think there is any benefit in it No one is going to leave, now what will we do even if we know and if it is not so - otherwise it will be more sad, It would be sad that if we did this, it would not have happened.

25. In routine day to day operation who takes decision about the major activities (purchases, treatment, spending money) about the family? (Probe: involvement of the mother/women in the family, any other family member, consultation with any other member)
Mother: They take the same.
26. During the hospital stay who discussed with the doctors/ nurses to make the decision(s) about treatment and other procedures. (Probe: father, mother, any other family member, did you/your spouse talk to someone in your family like elders or relatives to take critical decisions)
27. After the delivery, who decided about the burial and other post-death activities and rituals? (Probe: father, mother, any other family member; did you/your spouse talk to someone in your family like elders or relatives to take critical decisions)
Mother: Yes, in the husband hospital, I was inside, they were outside, they must have asked in the outside (to elders). They were our brothers, they are also brothers and they are brothers, they are older than them.
28. Now, we are completing the interaction/ interview. Would you like to add anything?
Mother: Say what was meant to be done, it is just that care is not right, the care of these people (HCP's) is right Is not and even talk is not good, somewhere they will kill, some will abuse, some will say, they do, the desire is Be good with what is going on (discharge).

In-depth Interviews of parents who had Stillbirth–17

**1.1**	**Primary respondent (relation with the child)**	Father, Mother
**1.2**	**Age of the primary respondent**	Mother- 30-35 years<br>Father- 35-40 years
**1.3**	**Other member present**	None

Events that led to the stillbirth

2.1 Can you please let us know about the problems, illness and events immediately before delivery?

Father: Then after that she is pregnant after 3 years, a little in 3-4 months after getting pregnant.
His legs became problem, he started having pain in one leg, so much so that he was upset while walking. Gone alone, everyone has children after having a test here, it is not that we still deliver At the time we were ready to come here!
Mother: We thought that everyone's children would be there and that we too would become the son, when we have another son Was, we were not here by ourselves, then, our son suddenly became completely, and that means we were dead Was
Father: And this problem started telling us that Delhi will run later, our packing is already done. Once treatment or checkup is done, tell me actually for you under Pulse Polio Program I am also a supervisor since 2003, so all those P.H.C. And chc know all and nurse It is known only from other things, after going there and getting the check, he said that it will take hardly an hour and It didn't really take an hour and delivery was done, but as soon as the baby's head comes out Kilo's baby was there, as the watermelon bursts or not in the same way from behind the head, I will show the photo now you,
Mother: Both of our children have the same condition that both of them have problems, when they are, no Not at all, Massa: Allah, there is no problem in that, I can see you now, where has he gone, yes, yes Since then, there have been two, my son Mohammad. Aas!
Father: Now it has started speaking of stuttering, it is not yet 4 years old, meaning 4 years Will be done in february
Mother: When both of my children are born, I have become both in one year, one of my sons was born on October 5. Last year and this girl of mine was born in S Hospital on October 12 was only weight, my daughter's weight of 1 kg and the boy was two and a half kilos, the bus has been a problem in both heads, that's all I have to know now that this How do our children become outside?
Father: This girl who is on October 12, these are the heads I took photos, here I am, this head increases, which is soft. It is known that these are stains, it is coming out of the head and pus, this is the show of the girl and this boy, it was told He told about treatment for six months!
Mother: Their head grows, yes, yes, to find out what is the problem in me that the children should be like this goes out, now I went to see tomorrow, but now they are saying that there is no such thing, then they If you say good then get this test done
Mother: Yes (test report)
Father: Bring the report, remove, this is Madame, the boy's photo, this head has increased,
Mother: Yes, it goes like this
Father: It did not increase much because its weight was low and it was two and a half kilos (boys Of) Just like bleeding happens at the same time all the pus came out
Mother: The daughter did not have this much and this time the child was very weak, my 5 days admit here she was in the hospital and people were getting normal delivery and there was no problem.
Mother: Here we had come to see, it was a week, sir, when we had a daughter we had gone a week After delivery, and now has come, had not called, or had come now, and said that all Test and mean
Sister: It was said to take all the tests
Mother: She had given this slip and said that after doing this test, she will come to see that your What is the problem inside!
Sister: Senior doctor, who is doing treatment, is senior

Mother: What happened that both my children were like this, how is this happening, this is what my first son was right It happened, it was normal, there was no problem, now it is not known what the problem is now is
Father: This older son has become two or two, three days, he did not know at all. Did it or not
Mother: Did not know whether it was two days or not
Sister: And as if your daughter was not feeling this way too
Mother: They didn't notice it
Father: Just, on Monday night, he has sweated
Sister: And it ended on Monday, they were also suffering, there was trouble, panic.
Father: They have sweat on Monday night
Sister: So he said that I could not sleep at night, and I have been sweating a lot and if I am nervous, then I said, you come, so I said that your brother should come from school, then I Will come, they came again on Tuesday night but they did not even realize that Baby is over inside!
Mother: Because there was no problem at all
2.2 Where did you go or whom did you consult for any problem during the last pregnancy?
Father: I tell the whole thing in detail, I was married in 2014, after getting married, within a month and a half Pregnancy done! After pregnancy, it happened that they started getting feverish a little bit, got typhoid, The condition became such that it was 68 kilos when they were married, then there was a lot of personality, then fever after this It happened, got typhoid, we kept showing it to the doctor, even her mother was taken away, so she too had 20-30 or 40 thousand He put the money, that is, the condition came here that he stopped breathing, and coughed, So some people said that at that time when the cough intensified, it was 3 and 6 months or so, then their condition was that they had come within 50 kg so that the weight was reduced, then we brought them here, dispensary Shown here, two months so bad people of our Malaviya city, so the response here Not found, the condition kept falling throughout the day, actually I was on duty, when the condition continued I left it to them, the pregnancy was about 6 months old at that time, after that our Brother went to see some mean patient who was admitted there in S Hospital, his voice stayed there Gone, when the voice stopped, they used to stop breathing like this, then they called, they called It is said that if you bring S Hospital and bring all the papers, then when S Hospital goes
He looked in the emergency, and after seeing it, he often did it by applying a wooden slab.
He said that T.B. Is, and has become so dangerous and their lungs have deteriorated and 2%
There are chances of survival
Mother: Some people said that cough occurs when you have children, not enough time was due Showed, showed dispensary but there was no response, response was not received, yes In MN, they had so much cough, so much cough but no response to cough Got it, now it has been fixed, it has been 4 years, so tell me yes!
Father: Then after that he referred to here, after this he referred us to Mehrauli Referred, stayed there for about ten weeks in Mehrauli, he said that of course the child Do not think at all, it is difficult to save them too, they said the same thing, however, T.B. Treatment of going on, this also came true, when he took leave, referred him to S Hospital, our baby
Okay and now 8 months old, I was late and today Massa: Allah is all right, baby Our right had 4 injections to him!
Mother: Yes the boy was born in S Hospital, and he is now fine and 4 years old, and is still fine, and now I am 3 I became pregnant after the year, and one of my legs started hurting,
Father: After 3–4 months, there was a slight problem in his legs… he started having pain in one leg… having pain It felt so much that this move made the whole person nervous… and once here, they were taken for testing by everyone, everyone There are only children. so .... It is not that we were here to come here at the time of delivery.
Mother: We thought that everyone has a child there we too will become a child.
Mother: We thought that everyone's child would be there, so we too will become another child.
When the second beta was about to happen, we did not come here on our own, S Hospital… our son, suddenly it was there. And he was killed .. and he was killed .....

Father: Started saying that Delhi will move later… Come on, our packing is done….
Mother: Did not go to MN and other than that, did not show there either (in U.P.)
Father: See, Madam, there are only normal ace which means delivery in labor room. Ladies means nurse, they used to check them all, there is no problem, any kind, right In the ultrasound there, also understand that when the child was finished, the boy was my child 4-5 days ago. Had it made, then the child was given the normal, well then everything is wrong there, only they would check it are that we had read the need for a treatment, we had read the requirement of a test, then we will take it here Have come to S Hospital or MN,
Mother: We have shown it in S Hospital itself and here we show
Sister: They came to Malviya city when the fifth month was going on.
Mother: Second-third month
Sister: It was the second month,
Father: She had a problem.
Sister: Yes, it came then, it was shown here, and it showed here that it was said that after a month to show you, then they went home, then they relaxed, they did not come, when they came showed right then told
Father: When the ultrasound happened, then it was right, they said that it was right to take ultrasound We assume that you have an ultrasound, but there was a slight mistake here. Ultrasound, if they don't believe ultrasound, it was just a matter of beating
Mother: It is a matter of two months, what does it show in 2 months
Mother: yes (current delivery se related)
Hospitalisation period
3. Now we would request you to tell us something about the period of hospitalization for pregnancy, delivery or termination?
Mother: 7 days, the delivery day was in S Hospital
Sister: They did not come to the village, on Monday the daughter did not know that our children are not bursting. If they were bursting, they did not come on Monday, they had come here on Wednesday night, so they told them (Wed On the night of)
Sister: So I asked him to go to the hospital, so he said that right now there is no problem, now he is not going We will go to the hospital in the morning, then yes we came on Tuesday night, then on Wednesday we went here, not S Hospital, MN he said, first you go and get ultrasound, we When ultrasound was done, the doctor told them in the ultrasound that you got water due to fear. Is on the child and you go again quickly check its beat and check the beat again, We got the beats checked, the beats did not come to the child A little bit that would be engaged but Then we got the ultrasound done again, she showed it to the doctor and went away and started crying. There is no such thing, I said nothing, there is no problem, it happens, it I get scared so I did not tell you! Then I asked the doctor, He said that your child is dead and
You have died many days ago, you take it to the labor room quickly, then we take it to the labor room
When he went, he transferred for S Hospital, said, call the ambulance and you take S Hospital
Go away, come to S Hospital, it takes two or three hours.
Father: Arrived around two o'clock at half past seven o'clock
Sister- At seven o'clock he had shifted us to the ward ....
Take the blood, do it, do it .. I said, we will give the blood, the child is now over, so you can admit us
Lo ... Toh saat baje hame kara hai hai ... We went to the ward at seven o'clock .... The doctor has seen the meaning .... said, bring blood
We said yes, I will bleed .... Toh said, Sir, our father is speaking, it has been four days, the daughter is finished,
The child will have problems…. You do something fast, bid you have to do the operation, then you speak like this… ..and my
You have come close, do not take tension, I will do it myself, whatever I have to do ... I said ok mother do you Then on Friday evening, at four o'clock, he was also normal …… in the morning he applied medicine… then again medicine

The daughter had already been made before it was not planted…. She had taken it around 3:30 pm and the daughter had become our daughter….
It was normal…. He had forbidden that no children should be children for six months…
4. Could you please describe your overall experience about the hospital care?
Mother: Well everything is fine there, we didn't face any problem, and mean
Mother: No, nothing happened to us (kharcha)
Father: What is the cost of water? It is just a little, see medicines prescribe outside
Mother: Write the medicine out a little, and there is no other expense
5. Could you please describe your experience about communication by the various hospital staffs during hospital stay?
Mother: No, that was good
Sister: No, not that, I used to speak more, when I ask anything, the doctor is the same, I used to ask Doctor, it is such a thing that if we are a doctor or you are a doctor, we are seeing ourselves.
Once you go, he shouted at me that this was me, I mean three to four days baby
Gone quickly, it is good, If the infection does not happen again, I had this rate, then you go
We will see for ourselves, we will get angry once, let us not worry about our problem
Was, then the mistake was that we did not tell us that the baby is over, we knew that this elder son
Haven't they also known these two days?
Mother: Everything felt right (behavior) Not everything felt right
Mother: Because my two, I mean older boy, son happened on the same place a week ago
I had no problem doing anything there
Sister: First of all you said that you will not give blood till the first two, you will not go, you went to the labor room before giving blood When I was showing, the doctor did not talk, he said, just wait - stop now, so he went to give blood They had friends, they were in the village, they could not come, so call me, my husband was not even, So we had sent it to our friends, they came and said, "I will give it in law, tell me where to give blood, then I told them our The blood giver has come along, take the blood, fill the form, then say, wait now, don't see the patient, their staff Had come two to three times, he had to go somewhere fast He had a flight, he was gone, could not give blood, then we had a Friday, then my husband and These bids will not be taken away until blood is given, I said to my husband, let's talk No i will give
Mother: It was at that time
Father: Like this, unless you take blood four six days ago
Sister: Won't Move
Mother: They said that like this is your third child, now your second child in a year
This is why we had a lot of trouble, where will we take the blood from that time, then we need blood
Father: We will not give you blood till we do not have delivery
Sister: I went to ask for blood, our husband gave the blood, I brought the card, before that, take it
Had Labor
Father: And not even blood, not us
Mother: Not even blood
Father: While the blood was so much needed, it was needed, but they should have been offered, but
Also taken blood and not plated
Mother: If they don't think now, don't offer it
Father: By the way, in S Hospital, everything means everything, everything is right, everyone sees it correctly because I also had my treatment with treatment, medicines prescribe outside.
Mother: No, no, said it very well, understood that he had spoken with love and said that meaning also
It was said that after six months, do not try to have a baby yet, there is no problem right now.
Treat yourself
Stiilbirth
6. Could you please tell us about the declaration of the event /outcome?
Sister: Yes, who did the ultrasound, MN, doctors were ultrasounds, gents They told
Mother: Yes, in MM Hospital

Sister: GG (in MM Hospital)
Sister: After that we went there again, the labor room, MM Hospital's labor room, doctor sir
In such a way, he has sent from below, he told me that you take it to the labor room, do not take them any longer, I went upstairs and said to me, doctor, this case is no longer in our hands, you go to S Hospital,
He said that your child is very weak, we cannot produce here
Mother: Your child is very weak, we cannot produce here
Sister: He said you gave the number of S Hospital Jao Ambulance, call 102, call on this Ambulance will come, it will be taken to you, so I said good sit outside, do not say it will not sit outside Let them sit here, when your ambulance arrives, then take them, then ambulance arrives, we bring them Went, she was unconscious on the way, listening
Father: These ultrasound (stillbirth)
Sister: Those people had spoken after seeing the ultrasound
Mother: No where,
Sister: Yes, did not do ultrasound in S Hospital in the labor room, he saw it and said
Father: The second day he got it done
Sister: The second day was done,
Mother: Yes, baby there
Sister: No - no, he said that the doctor is finished, I sent you from MN Have told you
Mother: They took me to the emergency labor room and checked, saw my heartbeat, checked my stomach
Did you mean your child does not mean yes baby you are over
Mother: Yes, the doctor said, who sits in the emergency, the doctor said, that your child is not is
Mother: She was alone (doctor)
Mother: No, no, very well
Understood that I had spoken… with a lot of love… and also said that after six months you mean having a baby… now
No problem ... you get your treatment here

7. What was your and your family member's reaction when you were told about the stillbirth?

Sister: Yes, the feeling is nothing, the hand, then we saw when he was born, he had not already told
I have not told them that the child is spoiled, I had started crying, the doctor said, you will be upset.
So it will not be much trouble, I did not tell, but they felt that there is something.
Sister: When he came to know, Mem in had thought, the doctor had told him like Malaviya
When she started crying more in the labor room in the city, I said there is no point in crying, which had to happen We took them in an ambulance, they had become there in the ambulance, they had fainted, they were not conscious, Eyes were torn, sir

8. How was your experience about the procedure after death till handover of the body to you?

Father: Didn't even show them
Mother: Didn't even show me
Sister: Initiative to bring them to ward
Sister: No, nothing was given at that time
Father: Did not give the time when the child was given a paper
Mother: That's
Father: Rather, he had given a big register, I went to the entire number four counter by making an entry in the emergency and then
Sister: The file was given by him
Mother: This paper was given
Father: This paper was given, Baby's
Sister: It took some two hours or no sister
Father: yes (baby handover)
Sister: They gave us around seven o'clock
Father: Get it
Sister: gave at around seven o'clock

Father: Had child at four o'clock
Sister: Yes, it was seven o'clock, seven o'clock, half past seven o'clock,
Father: It was dark, in the evening,
Sister: Yes, the light was burning, etc.

9. Who all from your family and community / neighbourhood were present at the hospital and how they supported you during this period?

Sister: I met them right there, I called them from old Delhi, they reached there from there,
Sister: Yes, they had gone somewhere, they got these hospitals from there, we got them standing there, yes their condition was bad, they became more upset
Sister: No, I did not say anything, I did not say anything, I said that my aunt was my mother-in-law, she said I Seeing that he was not going to let me go in the labor room, then said, I will give you tea now and I have read it If the bride has more trouble with tea, then she stands Gord, she is going to take her to the labor room. She went and said, "Come, give me, come, tell the name, we told the name, she went to take tea and then came to take tea" Le is now giving tea even when her baby is gone
It did not say whether it was alive or dead, boy and girl did not tell anything, one and a half, two
Took hours, we have thought that the above has said, "You know something, you put it in the machine." If given, we have not told you yet. We waited. After waiting, the doctor called us.
Come here, look at the child, when I saw my husband and my mother-in-law all of us

Post event procedures and coping mechanism

10. What all rituals and procedures did you follow after stillbirth?

Father: Yes, he brought it here
Sister: They brought it, I stayed with them
Sister: That means buried, we bury people
Sister: Yes, yes
Father: Yes, she was bathed
Sister: bathed him, then buried him in a cloth
Father: Like Nahlake, he was fed Nahlake's photo.
Mother: We do it in white clothes don't we
Father: Then after that our uncle was here, uncle live here
Sister: They were also gay after listening
Father: They also came
Mother: We had uncles, aunt was mother-in-law, and our daughter-in-law, Devrani was with us, and our two Nandas Has been married, Fufia's mother-in-law's girls, both of them had come to their husbands, we had eight people Of family
Sister: We only called
Sister: They just said that they were crying, we were crying, don't bother, those who are above
If it is written in luck, there will be no benefit from crying, explained as if we understand the vehicle
Sister: The uncle who was ours started crying, saw the girl, that the one above gave two and took both for
Father: This is the cemetery, this is where you were coming from, behind that
Sister: Petrol pumps are there in Saket, yes, right here
Father: When there is prayer, when we have breath in the child here
Mother: Breathe
Father: When a child is born alive, then it ends, if the child already expires
If he does, then he will not pray
Mother: As soon as we see that the breath has come
Mother: No, later on, our handmaids, etc., they get installed later. Time is the same
Sister: No - no
Father: As if that means, there is nothing like if the child is alive, then its more It means okay, well, when the third day is bright for the second day, then our eight is bigger than ten If he is a child
Mother: He has nothing to do with the baby

Father: If there is prayer then it is the life of the child as if it is alive then it ends
Sister: Should have breathed a little
Fahter: If the breath comes even a little bit, after it is born, then its prayers are valid,
If the rest is cloth-unoccupied, then put it on the wood etc. Let's bury
Sister: No - no
Father: As if that means, there is nothing like if the child is alive, then its more It means okay, well, when the third day is bright for the second day, then our eight is bigger than ten If he is a child
Mother: He has nothing to do with the baby
Father: If there is prayer then it is the life of the child as if it is alive then it ends
Sister: Should have breathed a little
Fahter: If the breath comes even a little bit, after it is born, then its prayers are valid, If the rest is cloth-unoccupied, then put it on the wood, not from above, dig the pit and put it in the grave Let's bury

11. How did you, your spouse(husband / wife) and immediate family members cope with the difficult phase?

Father - We have… Our thing is that any member of our house or any relation, whoever came to them
It is explained that it does not matter as they continue to explain for them .... Most of all, this is the fact that it is right for us
For .... the most important thing for us is that it is okay .... child, if it is right, it will give it again in the coming time .... someone
This is not the case .... it is just that they make the heart understand that it is okay .... no problem ....

12. Is/ was there any change in the interpersonal relationship between you and your spouse and other family members after the event?

Mother: All right (relation)
Mother: Yes it is
Mother: No, nothing (change) is the same, no problem.
Mother: No, that is fine too, I understand, if I cry, everybody is ready and my husband also means
It doesn't matter, when you get well, the one above will give more, that is not the case, nothing else is
Mother: I am having a problem, I am having a lot of swelling ever since my daughter was born.
On my whole stomach, on my legs, all my legs have started coming, not before, now it has started happening, since two months

13. How did family members support you and your spouse(husband/wife) during the difficult hase?

Father: Look, this is the most important thing
Father: See, we have said that we are any member of our house or any relation
Whenever anyone comes to the house, they have to explain it, meaning they keep understanding for them that there is no problem The most important thing is that they are right, the biggest thing for us is that they are right, they are right
Father: If the child is right then he will give it again in the coming time, then it is just that he understood the heart Let's say it's fine, no such thing

14. What were the reactions / counselling and suggestions given by the relatives/neighbours/ community after the episode?

Mother: Yes, I cry now I just feel nothing else, say why do you cry If it is ok then I will give it to the above, no my husband takes care of me very much. I was sick when my son was born, I was staying with me for a week, used to sleep there at night in Mehrauli, If I used to go somewhere, he used to go with me too, he used to take care of me a lot, still means a lot Take care of me

15. Do you think and /or discuss with your spouse/ family member about the loss and related events and blame someone/something or feel guilty for the same?

Father: How much problem is there on delivery time
Mother: That's why I think about this again and again, and even now when I think, I

It is sad from inside that tell me nothing has happened even in such a situation, it happened both times.
Sister: The first baby was born, it would not go away, they used to get so upset, now so much
Did not happen even before there was a lot of trouble
Father: Even after taking nine months of trouble, the baby which was not the last month was the one who was hurt she used to walk, she would not have walked
Mother: Nothing found after taking trouble Father: So now, after delivery, you get nothing, what a feeling it will be Mother: And everyone's children were watching there, and I empty my lap, I don't think of myself that’s why I still think about this
16. When did father and mother (if working) returned to work after the event?
Father: The work, the work, they have not yet
Mother: I am not working at home right now
Mother: yes a week
Mother: Did not go to school, had to leave duty
Sister: No, school did not go for ten or twelve days, it has come now, has been off duty for a week.
Father: Understand what Dudi is
Father: On the day of Mars, then there was delivery, their delivery on Saturday after delivery
It happened from there, they stayed here for a week, because people are coming to their place and they have there was no condition to wind up from here
Sister: In a journey, it takes seven hours to go, sitting seven hours
Father: It takes six-seven hours from here, now to go, I thought that at least they should be able to sit, Then we took them there after we took them
Father: So since you've been resting
Sister: Ever since she is resting, now she is rested, Sister says that I am tired now
I will not work, I do not say no, not now, when it is done then do it
17. How your and your family’s life has changed after the event?
Father: No sister, there is no such thing
Mother: Just I get angry more
Father: Just a little bit more, they mean a little bit
Sister: They start crying, they get very angry, they start crying, thought of thinking quickly
Two will be given by the above two and take both

Mother: My son is alone and he would say, if he sees everyone, he asks me to give me Babu La, then I
This is why weeping again and again means that the above person gave and gave me both times
Mother: Yes it is (atmosphere), no, no change
Mother: No, nothing is said by someone
Knowing the cause of stillbirth in detail – autopsy and MITS
18. What was the cause of stillbirth mentioned to you and what is your perception about the value of knowing the cause?
Mother: Yes, I want to know what it is that the child gets spoiled, I have to find out what
What is lacking in me, because only then will I be able to get treatment
Mother- They had said that at that time, the infection had told that the uterus has infection and its
There will be treatment, there is nothing to be disturbed… It was just told… We focused on the first one.
No .. our child was there before, neither did we notice because it was close to four months
It had slipped and fell… due to its strength, it… has not left any pain in their feet…
Even today there is pain…
Mother - Treatment is still underway for the feet ...
Father- Toh we stayed in it that it had fallen, it may have been in the head of the child…
Now this is what happened to our second child too.
19. In your view, what more could have been done to know the exact cause of stillbirth and the reasons causing it?

Mother: Ultrasound be mine, so that I know what the problem is in me
Mother: No, I do not know
Father: They told that if they want to do ultrasound then they have to do pregnancy then after that every one and half Behind the months, who speaks, who told the names of the children, they got removed from my mind, color Doppler,
Sister: No, (for ultrasound)
20. Are you aware of any method for identifying the exact cause stillbirth?
Mother - No, I do not know
21. What are your views about the autopsy/post mortem for identifying the cause of stillbirth?
Sister: Not post mortem, it is better if done by needle
Sister: No (postmortem)
Father: No, see now
Sister: If done through needle, it will not be much better than taken out by needle
Mother: Heart will hurt, she is speaking from her heart that it will not
Father: Well, that would have meant that but according to religion, we should not have post mortem here, According to us
Sister: By the way, everybody does what is better
Father: forbidden, mean
Sister: It is our decree from above
Father: That is what we mean by Habibs, as far as I know it is that after death
Don't want to move too fast, it should mean for his dead body, now for his body
If he does post-mortem, how much, what will happen to him
22. What are your views about collection of the tissue and body fluid samples for identifying the cause of stillbirth?
Mother: That means that his problems
Sister: It is in us that the fly does not sit nor does it weigh, we believe it
Sister: So that's why we said this
Sister: Ji (to take Tisu)
Father: Yes (MITS ke liye)
23. How parents and family members can be approached for the tissue and fluid sampling?

Sister: The one who is together will tell you, either there will be husbands or we will be, there will be all the neighbors.
Father: who will be together
Father: See, the thing to understand is that if you (I understood) see you will understand this, then
An imaginary fine and as you are saying that you are looking in a way through the video, that this
This is how there will be more knowledge than told
Sister: There will be more knowledge than told
Mother: You will get more knowledge
24. In your view, what factors are important for acceptance of the tissue sampling by the parents and family members?
Decision making dynamics
Father: See, the thing to understand is that if you (I understood) see you will understand this, then
An imaginary fine and as you are saying that you are looking in a way through the video, that this
This is how there will be more knowledge than told
Sister: There will be more knowledge than told
Mother: You will get more knowledge
25. In routine day to day operation who takes decision about the major activities about the family?
Sister: Yes, I agree, it is raised in the house, as is the case, but when the time of trouble is there
This is what will make sense, do what is good, what is better, whatever is there, say it right there.
It will not matter in this, now that we are together, we will say that do it

Father: Now as they are, they have been told any thing from inside, they will immediately pick up the phone, hook us up Or will we put our brother in such-and-such a thing, if we do not even understand then our daddy We will use the phone right from Daddy, and if there is an internet immediately, the better we think
Sister: That's such a thing, and if not so much time, then you have to take the option
Sister: Will take that immediately (right decision)
Father: No, see, this is what I understand in this, that they are pregnant, okay and their treatment
If you are undergoing treatment, then if you ask me to do this then I am the most I would like to ask you first and the opinion will be yours
Mother: That is our opinion
Sister: The same opinion will be ours
26. During the hospital stay who discussed with the doctors / nurses to make decision about the treatment and other procedures?
Father: Because I am not a doctor and the treatment that I will do from the beginning is the best public Will happen
Sister: The best public would be
Sister: Now we, when we went at night, the doctor here, we could not find the doctor who
He had treated them, he said that you came to show me and then we went to someone on Monday
Did not show there because he said that he came to show us and we left all the doctors
Come on, and gone yesterday, got a doctor, she is a big doctor,
Then he showed them on sight
Recognized that yes baby, you were like this, you will have a checkup then you can tell which
Karan has happened, and you will be treated for this reason, will the treatment go, said, Now let us see and check
Then we will tell you when your treatment should start
Mother: I do not know yet why your child is deteriorating both times, now all your tests
After getting it done, then you will tell what the problem is, what is the problem, now these tests will be conducted by Monday
Sister: First we are the mother-in-law in the village, we ask them, and what about our Mian Biwi
Ask Husband, Husband will ask his father that this is the case
Father: There is something that is related to the whole family, well, we will dispose of our parents.
Okay, something else means that we are older when it comes to husband wife
Sister: Husband will say that if you do it or not, then it is
Father: For the treatment of these, do not see, I will get the masavra from my daddy or not, but from his mother or Must take from dadd
27. After the delivery, who decided about the burial and other post- death activities and rituals?
Mother: Have to take from my mother, because my mother is very dangerous
Father: because
Sister: She does not speak, son, which looks good, does not have to ask, it is just this
Mother: Ask my mother before doing anything Leo
Mother: They only do telll

In-depth Interviews of parents who had Stillbirth– 18

**1.1**	**Primary respondent (relation with the child)**	Father, Mother
**1.2**	**Age of the primary respondent**	Mother- 20-25 years<br>Father- 25-30 years
**1.3**	**Other member present**	None

2. Events that led to the stillbirth

2.1 Can you please let us know about the problems, illness and events immediately before delivery?

Mother: No (no problem), no

Mother: When I meant the 9th month was about to start, I went to the doctor here, 13 number street, they said that your blood is low meaning it was 8 grams. baby's Weight was also less, it was increasing, now it has reduced very slowly, it was said that just after that Then I showed up here in S Hospital, I checked there and it was just right there and told me something.

Mother: It's around the time of 8th month   Mother: Yes, it was ten days ago

Mother: No, no, I was doing all the work normally, there was no problem for me, everything was normal, I showed it on 19th, I was having pain at night, so I thought. This is just a normal pain. It was for the first time. So it was auumed that may be there was problem in normal day to day routine. So I slept like that so at day time. Around 11 o'clock I was completely cured, the pain subsided, I became normal, I cleaned the house Diwali was near , I and I had pain at 20th night. It started around 1-2 p.m in afternooon.

And during that I kept on controlling that may be it is a normal pain, it is just happening like that but nothing happened at that point. Then I called them at 9 o clock. I called at around 8-9 o clock that pain in unbearable. let's go to the doctor now let us go to doctor now. So bhabhi ji saw my abdomen and said that everything is fine.

Show the doctor what is there, I reached there at 11 o'clock and I was checked there at two o'clock, yes it was just this in emergency, but there was no problem there I started bleeding on arrival there. Mother - Nothing: They said that the child is not breathing they did my Ultrasound they did everything they said that baby is finished inside. It was already gone a day ago.

2.2 Where did you go or whom did you consult for any problem during the last pregnancy?

No, it was 9 th month.

Mother: I got it done from outside. One was from   S Hospital and other I got it done here in number 13 street In Govindpuri, I had hired a consultant. (in which moth did you start the treatment) In third month . Since starting you are showing in S Hospital? No I showed there in I think 5th month. they told me that there was a problem there so I could get it done There was something, just you have to fill the form, you fill the form, get it checked, You will get a file. So your thing will be done fast. So I got the check-up done here also and outside also.

(where did you go) (nearby)

Father - 13 number street Mother - 13 umber street.

(everyone from your locality goes there only) No there are a lot, 1 number , 2 number.

My mother goes there always. No doctor is there. there is a hospital.

Father - Mummy always go there, They get their check up there only Mother - his friend is there someoone known is there.

Mother: No, bhabhi lives there, meaning, aunt, whoever she is, her baby was born there.

He had said that is a right place there, He does the right treatment. correct treatment. Father: Their delivery was done, He has a child, 1 year old. He had just come. So he was saying how does it matter.

Mother: And this is what happens people send in an emergency, and this is what happens when people send them in emergency, There are private hospitals. They only send. so first go there this was in my mind.

Hospitalisation period

3. Now we would request you to tell us something about the period of hospitalization for pregnancy, delivery or termination? (duration of stay)
Mother: It happened on Sunday at 2 o clock. And I was free on Monday, three days
Father - had stayed for 2-3 days Mother: Yes it was on Saturday, around 3 o'clock in the night and I came back on Monday at home. Doctor did not come on Sunday so she had to stop there check up was done on monday.
Hospitalisation period
4. Could you please describe your overall experience about the hospital care?
Mother: It was 10-15 minutes later, It was a normal delivery was done
Mother: No, no expense.
(had a document, etc., signed, etc.) gave some paper
Mother is asking Father ... where are the documents? Father - in the bag
5. Could you please describe your experience about communication by the various hospital staffs during hospital stay?
Father: Only one doctor who got my signature. Rest I was not able to talk to anyone. Nothing, they just told that I am not able to breathe. He got my ultrasound done. Then said that nothing. Baby is no more alive inside. It was over a day ago. Doctor had told. A person who checks. Yes first they checked my abdomen. Then they took us into ultrasound room. Then they checked on their own. He himself checked that then he said that there is no chance that the child is there or not, Mother - no not anyone patients were there I had talked to them only. I was just talking to patients. No one else. There is a ward they come to give through that ward.
Everything is fine, it is right… It is all right, because when you go to emergency so they don't pay attention. They care a lot after delivery. they also take care of eating and drinking, Give medicines on time .... everything is fine
Stiilbirth
6. Could you please tell us about the declaration of the event /outcome?
Mother: The doctor had said that those who check all that Mother: The doctor did this, we showed it to them that see baby is no more alive.
Mother: Nothing, just held in my hand and showed it to me that here is the baby.
Mother- Doctor had said that look here is the baby. He is no more alive. Nothing just held in my hand and told me. Normally they interacted very politely.
7. What was your and your family member's reaction when you were told about the stillbirth?
Mother: Nothing, nothing what they would say… No, they did not talk to me… whatever they said should happen nicely. When delivery was done, then baby was given in their hand. So they said nothing.
8. How was your experience about the procedure after death till handover of the body to you?
Baby was given to mother-in-law. Mother - yes (baby handover by nurse)
Mother - no, I don't know, after delivery I was made to lie down after that what happened what did not happen that I don't know.
Father - It was given in white cloth then my mother brought to her. Then I kept sitting there till 4 o clock.
Mother- gents are not allowed so whatever was there was done by my mother.
Father: hmmm (baby handover by nurse) Father: I kept on sitting there till night.
Could not do Father: Yes, (delivery time) Father: Delivery was done, so he came about 3 o'clock came to me, first they were looking at me. They had told me to sit at one place. So I kept on sitting there. My mother was searching somewhere else. First our time was wasted in this. Then she came and said that child is dead. Then they let us made some tea. He was buried till
Father: My friends had gone, I had called at night then they were saying that they cannot come
Then in the morning at least till 6 o'clock in the morning, everyone had reached. It was around 3-4 o'clock, all had reached there.
Father over there: Yes Ji (Hindu dharma) Father: Did not do anything at that time just at the time of burying saw the face and the people who are there in graveyard they did everything.

9. Who all from your family and community / neighbourhood were present at the hospital and how they supported you during this period?
(Who was with you in the hospital) Mother: My mother-in-law was there. Mother: No one else (YOU'RE FATHER) Father: I was out Mother: They are not allowed. Mother: Yes-yes (outside)
Father: No, when I left them at 11 o'clock I was standing outside the gate and my mother was also there she was saying your name will be called and you have to come inside. Then they called suddenly saying that you have to give blood. And have to sign, that's all. Then they called us, we signed and told us to go outside. Then I came to stand outside then not around 1-2 around 3 they called my mom and told that child is no more with us. No, I did not say anything I have to give blood or no need to give.
Father: I got a lot of support, my brother, My mother, my brother also went, is handicapped, he also went, together I told him you go away overnight As I have to stop, if you go to the house, if anything happens, we will call you, brother gone, mother went away. Four friends went from here, four friends went from here. then they saw us sitting there then said to all friends What will you do, will let you know on phone, then those people come home, then I, my mother and these three people And everyone has supported
Father: brother did it, nobody accept him. (Paison se support) Father: yes-yes
(Chid burry support)
Mother: One was my mother, only mother-in-law was with me, she supported me, to eat and drink. Also took great care of me. Mother: No, they were not told at all (nebhiours come)
Post event procedures and coping mechanism
10. What all rituals and procedures did you follow after stillbirth?
Father: Yes sir (Hindu dharma) Father: Did not do anything at that time, just saw the face while burying. There is pandit in cementry so whatever they told we did that.
Father: No (Bathed)
Mother: Well he cleans up (did your mother accompany you for burial)
Father: No Father: No, she was with them we went to the hospital again after bathing. So we came in morning. She was with mother
Father: No, the ritual was done for the peace of the house
There we have. Father: assuming 7 or 8 days and 10 days (pooja path) Mother: Mother said to do in thirteen days I said hurry up because I had to go home myself, my mother scolded that come home soon, I was not well. Then the mother had called, come to Patiala Punjab That's why mummy please do in hurry I have to go back home. I will not stay here, I don't feel good. I live alone at home all day, that's why I got it done quickly, we were only 4-5 people, and got it done at home
11. How did you, your spouse(Father / Mother) and immediate family members cope with the difficult phase?
Mother: By the way, what can I say just, it doesn't matter, next time it will be ok. Twins will come, that's the Hope
Father: grief is there madam, as we were thinking that it is our first child and so on
12. Is/ was there any change in the interpersonal relationship between you and your spouse and other family members after the event?
Mother: No, there is nothing Father: No, mam, otherwise not at all. Nothing in my mind Mother: Yes, now I pay lot of attention.
Mother: Yes, I say everything openly
13. How did family members support you and your spouse(Father/Mother) during the difficult phase?
Mother: I had full support, no one, not even my mother-in-law, whatever father-in-law was at that time, they were very caring
Mother: No, they are like that, no one is there, they themselves come and speak, that means that is in our house. Man is being built in Jaitpur, so he is saying that it should be made soon, when my mother-in-law felt that I was there I used to think that I was the only one I meant to be a caregiver, If I wasn't going to tell Mummy feels the same mistake is ours that we did not pay attention to the children, that too sometimes He used to come and meet us, that is that we lived together, someone lived with him, it does

not happen What has happened now, the only thing that happens in their mind was that I left the children alone.

14. What were the reactions / counselling and suggestions given by the relatives/neighbours/ community after the episode?

Mother: Yes, he said that he should not do any work for at least one and a half months stay away from from water as far as possible as much as eat all dry fruits are, juices are all food and drink, it should not be like that leave food to eat and drink Give up, don't cry, eat or drink as much as you can, be happy, you will concieve you have age. This much was said, take care of food, stay away from water, get massaged then they also massaged a lot mother in law also massaged a lot.

15. Do you think and /or discuss with your spouse/ family member about the loss and related events and blame someone/something or feel guilty for the same?

Father: No (kisi ko doss dete hain) Mother: No (kisi ko doss dena)

Father: One thing is there I reached there at 11 o'clock at 2 o'clock checkup was done what is the emergency all about.

Mother - took so much time Father - there was no Emergency again.

Mother: Because I have a lot of pain, at that time it was very fast, so I said that see me soon .... I was also sitting there, I am not able to bear the pain, they said that everyone is sitting patient and all. You also sit even she was on number one….they do formality with us…..They make us write…she took time in that only….. It seemed that there is only a little bit that why they did not do the check-up earlier, he went to Emergency Hum Father: No, I never get into a conversation with friends then my mind gets upset.

Mother: If they say that it happens to everyone, even to poor people, then why not ours

Happened, they say Mother: No, I started keeping myself busy , that the more I stay busy, it is good.

Right: Mother: No (bachhe ko yad karte hai) Mother: they don't mean they drink At that time

16. When did father and mother (if working) returned to work after the event?

Father: On work, I have my own business. They stayed with me for a month. Well I did not stay together for a month

Father: No (1 mahine kam par nahin) Mother: I think baby's 9th month He was already with me, left the job and he said that I have to work as a mechanic. Goes, they were with me Father: So far I have left work, I just sit empty, just Sometimes, if you get a small work, then go for 1-2 hours. Just Father: Work gets noticed.

17. How your and your family's life has changed after the event?

Father: The change has come that I have to go to S Hospital or not to go next, everyone is saying you are wrong Gone if I spent a little money there would be no problem

Father: Those who are private hospitals anywhere and my friends also have their Mother, all that private I do, there is no S Hospital, no line there is no problem of such a line

Knowing the cause of stillbirth in detail – autopsy and MITS

18. What was the cause of stillbirth mentioned to you and what is your perception about the value of knowing the cause?

Mother: Yes, told that the placenta was stuck, because of it Father: Yes, it seems (cause of child death)

Father: He only told (bacche ki mrityu ki vajah) Mother: Doctor

Those who had seen me had told them Mother: No-no (brain problem) Mother:

Was not scared, that it is something like Mother: doctor (cause of child death)

19. In your view, what more could have been done to know the exact cause of stillbirth and the reasons causing it?

20. Are you aware of any method for identifying the exact cause of stillbirth?

Mother: No, do it when it's not there

Father: What is there to do in this, Madam, which is not there then what is the benefit to her?

Mother: Yes, we live that yes, we have made a mistake, we have seen what is there, do not know ourselves

No, how did the baby go, it makes no sense to tear it?

20. Are you aware of any method for identifying the exact cause of stillbirth?

Mother: No, no, I was told that I was scared that this one of yours is over
Is, and why didn't you come before, scolding my mother-in-law, scolding mother-in-law, why you were the first I did not come, so I said Madam, when we have been sitting here since 3 o'clock, why did we not come, then why did not I see They are telling me that no, you should have made a mistake earlier, your condition is bad. Has been, meaning my condition was getting worse, my breath was also stopping at that time, they say that You were not taken care of, you were brought very late and said that what is your baby The head is separated, is split in two, now how will delivery be intimidated by all such people To the mother, the mother was crying there anyway, so that was it, so the mother says as it is Save it just means me
Father: Yes (postmarterm jante hai)
Mother: yes (postmarterm jante hai)
Mother: Not so much, so much that they see the body tearing and tearing it all
21. What are your views about the autopsy/post mortem for identifying the cause of stillbirth?
Father: Yes (postmarterm jante hai) Mother: Yes (postmarterm jante hai)
Mother: No, not so much, they see their bodies tearing and tearing it all so much Mother: No, when
If it is not there then what to do Father: what to do in it, Madam, who is not there, then what should she do What is the use of ripping Mother: Yes, we have lived that yes we have made a mistake, so we have seen whether Don't you yourself don't know how the baby went?
Mother: No, when it is not there, do it, Father: what to do in it, Madam, who is not there
So what is the use of tearing him? Mother: Yes, we have lived that yes, we have made a mistake.
Have you seen what is not, do not know how the baby has gone then there is no meaning of tearing.
No
22. What are your views about collection of the tissue and body fluid samples for identifying the cause of stillbirth?
Mother: Yes, it is okay for it Father: I would also speak, my Father: because normal
Checkup is happening, as if there is no tearing, it is nothing normal, then we get baby full without tearing Of
Mother: He is not suffering the same for this too: Mother: Otherwise it happens that this much stay ahead Ho, because my aunt had expired, she had done the post-mortem so that everyone thought it was useless. If this should not happen, then the meat is right, or the problem is not happening, at least to the human being. Why is it not dead, we think it is okay as well.
23. How parents and family members can be approached for the tissue and fluid sampling?
Father: What about (MITS) Father: Used to tell friends then what they would say
If something like this happens next, Father: Yes, I am (special person) Father: My 3-4 friends
Yes, all is special, the most special advice, plus there is also a doctor in AIIMS, which means my friend
They are in the technician line, their car is right there, so if their friends circle, then whatever they
The doctor takes the name of every doctor who comes to check, which doctor went to check them,
He simply speaks to AIIMS, what is the name of the doctor, he tells the tail, which one is giving Medicine
After sending him a WhatsApp photo, I told me that it was okay I asked him that it is right.
Mother: Video is perfect, quicker than video makes sense
24. In your view, what factors are important for acceptance of the tissue sampling by the parents and family members?
Father: What about (MITS)
Father: Telling friends then they tell me what to do next if something like this is there.
Father: Yes, there is (special person)
Father: I have 3-4 friends, all are special, the most special advice, plus AIIMS also has a doctor,
My friend means in the technology line, his car is right there, so his friends circle So whatever medicine the doctor comes to check on them, asks which doctor Went to check, he only speaks to AIIMS, what is the name of the doctor, he tells the tail then who is the medicine I was giving him whatsapp photo by giving him something, I told him ok, I asked him that it is right Is not it.
Mother: The video is right, the video quickly makes sense: Mother: That's what no one wants

Rip off .... my aunt was expelled toh, she was done in the same way.
Meaning that it should not be so… .. but it is not right…
The problem is not happening, at least that person may be dead even if it is… we think so
Ok brother now as is

Decision making dynamics

25. In routine day to day operation who takes decision about the major activities about the family?

Father: they only do

26. During the hospital stay who discussed with the doctors / nurses to make decision about the treatment and other procedures?

Mother: Gents are not allowed. So mother has done whatever was there. Mummy did all. Father: I sat there I couldn't do anything at night

Father: Delivery was done, so at around three came to me at around 3 pm, first they were looking at me. Where they had told me to sit. I was sitting at that gate only. My mother is looking somewhere else. First I lost time in this only. Then when she came, she said hat the child is dead then first they sent tea

Then he was buried at 4-5 o clocks.

Father: My friends had gone, I had called them at night then they were saying that I cannot come at that time. Then in the morning at least till 6 o'clock in the morning, everyone had reached 3-4 o'clock, all had reached.Over there

27. After the delivery, who decided about the burial and other post- death activities and rituals?

All friends took it, where to go, where it is good, where it is bad, they went there too That there is a cemetery next to S Hospital but do not give permission for burial.

In-depth Interviews of parents who had Stillbirth– 19

**1.1**	**Primary respondent (relation with the child)**	Mother, Father
**1.2**	**Age of the primary respondent**	Mother- 20-25 years<br>Father- 25-30 years
**1.3**	**Other member present**	None

Events that led to the stillbirth

2.1 Can you please let us know about the problems, illness and events immediately before delivery?

No, nothing like this happened.

I used to get nauseous even for the 7th month, my vomiting also stopped.

Yes, they used to say the same thing, it happens in pregnancy.

Mother - water had come out… .. I was admitted to the hospital at home, I was feeling well too.

2.2 Where did you go or whom did you consult for any problem during the last pregnancy?

Mother - Madam, we used to go to the dispensary in F dispensary where there was a checkup.

If we wanted to get it done, we used to go for the same ultrasound and she used to see it.

Mother - Yes, it is different from here, all the people of and F Dispensary go there.

Mother - meaning she comes from the first house, she spoke of the ladies dispensary, if you are pregnant, then you get the card made take it.

Mother - No, there is a dispensary, not a hospital.

Mother - I mean, we go to the Anganwadi or not to get food, we tell them to go and tell the dispensary Let's give.

Mother - the children of food are my firstborn children… 2 I go to leave them.

Yes, I was last seen when I came here. In the seventh month. He was a 6th cow.

Hospitalisation period

3. Now we would request you to tell us something about the period of hospitalization for pregnancy, delivery or termination?

Mother - She had gone to the hospital for a check, Madams had not called me after a month. That a month later in medical I have to show

Mother - I went to show there. So it was not the heartbeat seen by the child. He said go and show where These recruiters do it, get it checked there, showed it there and go to A Hospital and say there is absolutely no beating. He wrote an ultrasound and got an ultrasound done in the evening and said there the child was over.

Mother - Did not go to medical.

Mother - They asked that whatever is right is right, but now they have not told anything .. Medical people.

Mother - He had spoken to me Ultrasound, to write it, it was done in 112, let us do it in 112

He showed that there is water in your stomach, if you go to S Hospital directly, we went straight to S Hospital.

Mother - Bard then 10 number of bards where they stayed there, they stopped till evening after delivery, they discharged.

So what did you give that bed to you immediately?

Mother - Yes. Or did you have to wait in line

Mother - Yes, we had to wait in line and had to wait all day when the slip was entered.

After the mother's entry, he had called in. After the investigation, he had also spent time there in delivery. My It was hurting here, he was going to faint here, even then he had not noticed the doctors. Means much later It was noticed when the watery voice came out completely when they took my number.

4. Could you please describe your overall experience about the hospital care?

So when you went to medical for the first time, the ultrasound did not do anything

Mother - Make anything new How did the check

Mother - This is how he felt that he had just checked in his ear

What did they say then the mother had given medicines and the big memes of recruitment had also spoken, so they refused not to recruit, then they said that
Your child can also die, there is no idea through water there is no hope that means we will investigate on Wednesday. How will you not make water in your stomach?
You went on Wednesday then
Mother - Yes, no Madam was found there, then I showed the other Madam, even if I told them, they wrote me in the slip had given medicine to eat this medicine and come after one month.
Well, you left after a month or you are in the middle of the baby
Mother - No, we went a whole month later.
Mother - till then my baby's heart was not missing from me, my stomach was getting tight, so brother said, don't stop now Are going on when he went there, he refused and said that the heartbeat of the child is not over. They still in the evening I was admitted Did you spend any money there in S Hospital?
Mother - Papa and you will know this. I did not spend any money…. Have you had any problems
Did he come to call once when there was a need? How many times did you come / Satisfaction level & gt;
Mother - Madam, when Madam was in pain, Madam did not come on call. When my water ... was torn when it came to see the big one. The rest is hurting or something is happening, they have not paid much attention to what they mean the pain is going to be unconscious. He had not noticed this. Means I must be unconscious was when they were taken inside.
5. Could you please describe your experience about communication by the various hospital staffs during hospital stay?
Mother - Madam Ji was right.
Mother - used to speak to the nurse, the nurse would shout. Question - What did you say?
Mother - that means pain, tolerate the pain inside, it is a matter of the world.
Question - Did you speak anything else? Do you remember anything else?
Mother - No.
Stiilbirth
6. Could you please tell us about the declaration of the event /outcome?
Mother - was a doctor. Madam, the doctor sits in the OT, he did not tell me.
Mother-in-law, who was doing ultrasound, did not tell me that she had told sister-in-law
That means I will get nervous, I went there in Emergency. I was told in Emergency. You know your baby It is over I don't know that Madam. That your child has finished filling up the form for your recruitment Give. Tell your husband to get the blood stored. I gave that good form to my father, after filling the form then later He had recruited me. Mother - went out to give blood to fill the farm.
Question - have you gone then they came to give blood?
Mother - blood was refused. Less weight was lost and then father started giving. Did not even take fathers. Papa's BP High It is neither that the father's blood was not taken for this reason.
Mother - Doctors said that if blood is needed, we will give blood ourselves.
7. What was your and your family member's reaction when you were told about the stillbirth?
8. How was your experience about the procedure after death till handover of the body to you?
Question - So who should the child be given after that?
Mother - I don't know if I was inside. Was inside
Question - You do not know who brought the child and then?
Mother - Did not tell me. I was inside when the delivery was not mine, so I was sent out from inside on the morning guard. By the time my child was shipped. I had asked for my baby too.
Question - Haven't you seen your child?
Mother - No. Not even shown.
Question - You spoke to show?
Mother - I said. He did not show his child. It was just shown that your girl is a boy. My girl had shown all I had. I also said my children have a little heartbeat or something! Anything is now so you are finished baby.

Mother - She was my mother-in-law and was my mother, she must have given it to my mother-in-law or she would have given it to her mother. At home.
Question - Your sister-in-law is here who went?
Mother - No, she lives in Fatehpur.
Question: Any paper, some sign or wine, some paper is done.
Have you signed in? You all know that whoever was your sister-in-law or your husband was that they
Have you done some sine wine?
Mother - I did not know that I had taken all the forms from Mer.
Question - Ok what did you get done?
Mother - had the form done, that means the child is over and your delivery is over, fill this form and go
9. Who all from your family and community / neighbourhood were present at the hospital and how they supported you during this period
Question - So Ibrana ji, like after you reached there, how did you mean which corner was there with you?
Mother - I had a sister-in-law
Question - and?
Mother - Big bus.
Question - someone else was your husband?
Mother - He came in the evening.
Question- Well, they had come in the evening, just as you reached there, what did you have to do? When a month later have you gone with her sister-in-law, where did you stay? Which ward stayed in?
Question - At that time your sister-in-law was inside the ward with you.
Mother - we
They had got a lot of support they had made me all I don't even know
Was. I did not go anywhere. He was the first child of theirs in the building, so they got me all done.
My sister-in-law got everything recruited from the form
Mother - Yes it was spoken because of water.
Mother - Did not tell the reason, just spoke water. There is water in your stomach, but you did not say through which water is made
Through which we went to show at the OT that day. Was refused.
Question - Okay.
Mother - Yes.
Question - Did you meet him when you were admitted in the hospital? Or were you involved in the trouble?
Mother - I was involved, my mother-in-law was also, my mother was also, my father was also, my sister-in-law was all. Post event procedures and coping mechanism
10. What all rituals and procedures did you follow after stillbirth?
Question - Well then what happened to you - like you bury in your religion?
Mother - Yes, was buried.
Mother - meaning we had buried him.
Mom - I don't know.
Mother - My brother went. I had a husband, I was a mother-in-law, I don't know my sister-in-law or hers because I was at home was in the hospital.
Sister-In law - He was not buried here in Fatehpur.
Sister-In - Law - Mother-in-law came. Sister-in law - went to the house. Sister-In-Law- Now that mother-in-law knows will happen. I had not gone there, but my father went empty-handed. Men had gone, all the people from the house had come to buried.
He does not know.
Mother - I did not know Madam, I was in the hospital. I don't know, my daughter-in-law must have also read that because she have read and written. Sister-In-Law - She is educated. Mother - Yes, my daughter-in-law has read - written, she also reads.

11. How did you, your spouse(husband / wife) and immediate family members cope with the difficult phase?
Question - Okay. If your husband or your mother-in-law or whatever your family members are, what do those people mean?
Can emerge? With this thing? Mother - He was shocked.
12. Is/ was there any change in the interpersonal relationship between you and your spouse and other family members after the event?.
Ibrana ji was the first relationship between your husband and you Is it still what they still care about you as before
Mother - Madame, do you talk freely All about your husband
Mother - Madam, we do it right now but do not do so much because we are still stitches, but if we come so much, we can sometimes do things like a wife can tell her husband.
Mother − Mam means what has changed after the child's death
Mother - No, nothing came. They have just said that it has finished and they will come forward. They explain to me that there is no need to take a tension. All that happened is the will of Allah. They only explain to me
13. How did family members support you and your spouse (husband/wife) during the difficult phase?
How much sports you got from your family from other family members, how much support did you get at that time
Mother - was found.
Mother-mother was his.
Mother - Yes, my father was also there to support me, I was also my daughter to support.
Question - Have they emerged from this thing yet
Mother - They cry.
14. What were the reactions / counselling and suggestions given by the relatives/neighbours/ community after the episode?
N/A
15. Do you think and /or discuss with your spouse/ family member about the loss and related events and blame someone/something or feel guilty for the same?
Q: Do you talk to your husband about all this? .. there is a talk about the child
Mother - Madam is better than forgetting it now. Now there is no use in remembering, even if you remember it, it will feel equally sorrowful.
Do you still keep thinking about the child or do you think that is not done
Ok ok move on
Mother - It is not like Madame Children is children, now they were giving birth for the first time.
What do you still think of you still remember
Mother - No.
Mother - Now I am done, I am Madam. You also blame sometimes, sometimes - give.For what
Mother - Ultrasound time was done at that time, however, whatever was to happen, it happened.
Question - How much sport did you get at that time? Or what you say
Taking a child in the midst of a husband wife, do you mean if there is no further problem, then the family planning that speaks about all of these talks, talks about all these things or thinks?
Mother - Yes, I think absolutely not so, from now on, let go of thinking, now I have thought a lot. If we still did not go to get a medical check-up at night, so he refused to get the stitches cut. Right now the stitches are cut, so he refused.
16. When did father and mother (if working) returned to work after the event?
Question - If you are doing some work left at home, do you have trouble in working?
Mother - I feel very much like weakness.
Question - Your husband, when your child is finished, how many days after that he started going to work
Where ?

Mother - He still has his accident, he still goes and had become an accident by motor cycle.
Mother - It has just been 5-7 days, they had an accident. He went to work for 2 days, then he was an accident again
No .
...... .I went to my mother′s house more than my in-laws, I stay here, mother′s. ...... Madam sometimes do.
Question - So, are you going to stay here or are you going to leave?
Mother - Madame ji will be in 1-2 months.
17. How you and your family's life has changed after the event?
N/A
Knowing the cause of stillbirth in detail – autopsy and MITS
18. What was the cause of stillbirth mentioned to you and what is your perception about the value of knowing the cause?
Mother - No, I ask the doctor myself, why the water ended up causing the child to die.
Mother - Yes, father had asked. My sister-in-law had also asked, what is the reason, what was the water, through which, how was it finished, how did the doctor not tell that only through water, its root will be removed, how is the water lost, how in the stomach.
Mother - Madam, you had to know because there is no danger for the future. If we are ready for the next delivery, then if there is another child, then there is no danger to him. Had to get it done.
19. In your view, what more could have been done to know the exact cause of stillbirth and the reasons causing it?
Mother - Madam, when the post-mortem is over, it is over.
Mother - I do not know.
20. Are you aware of any method for identifying the exact cause stillbirth?
Mother - Madam, he did not ask me if something would happen after the post-mortem, we told you too.
Mother - it would have been right because you would have known why water was made for what reason and not.
Question - At the time when talking to someone?
Mother - Yes.
Question - Who is like this?
Mother - Do it with your husband, do it with your parents, do it with your mother-in-law, because it is the main thing, it will speak whenever it happens.
Question - What do you think about postmortem in your religion? In your religion
Mother - our post mortem is not done.
Question - No, why do we refuse?
Mother - Our body gets spoiled, get such post mortem done.
Question - well and what happens with that?
Mother - meaning the body will be removed from inside the body, the body will be spoiled.
21. What are your views about the autopsy/post mortem for identifying the cause of stillbirth?
Question − Ibrana ji, if you think that there are other parents who also think that if the child is spoiled, then what do you think that they will benefit from having these tests done to the parents whose children have been spoiled. Will this test be good for them?
Mother - This will happen because that child is finished for what reason, not for what reason. You will find out what happened because of which end.
22. What are your views about collection of the tissue and body fluid samples for identifying the cause of stillbirth?
Question - Asking your father or your husband, or asking your mother-in-law, who means that such a person would have got this job done quickly?
Mother - he would have spoken to his father or spoken to his mother-in-law.
Mother - Madam, you had to know because there is no danger for the future. If we are ready for the next delivery, then if there is another child, then there is no danger to him. Had to get it done.23. How parents and family members can be approached for the tissue and fluid sampling?

Mother - There will be no such benefit for the daughter, the parents will see that the daughter is right, the daughter and who
The child is finished. My parents loved it so much that even my elder sister did not have children yet. It has been 4 years since her marriage meant that my child was the first, they were very happy.
Question - Okay.
The same happened with my mother - and my child.
Mother - Madam, you are talking and telling me that because it removes blood, only blood will be extracted from the body.
Question - If we find this method of the mitts which we have told you, what is the test done by needle, then which member is there in your house so that by speaking to whom it will be possible to do this work?
Mother - take my father.
24. In your view, what factors are important for acceptance of the tissue sampling by the parents and family members?
Decision making dynamics
25. In routine day to day operation who takes decision about the major activities about the family?
Question: Do your husband take all your decisions on their own or do they ask someone else
Mother - Do it with your mother.
Question - Whatever responsibilities of a good household such as whatever deli which are cost dominated, who decides all these?
Mother - takes my mother-in-law.
Question - takes your mother-in-law. Whether to bury the child now or to take a bath, it was an important decision, who took all this for the child at that time?
Mother - My mother in law must have taken it.
26. During the hospital stay who discussed with the doctors / nurses to make decision about the treatment and other procedures?

27. After the delivery, who decided about the burial and other post- death activities and rituals?
Summarisation.
28. Now, we are completing the interaction/ interview. Would you like to add anything?
Mother - Whom should I say Madam?
Question - Anything whatever is on your mind, if you have to tell us something separately?
Mother - Madame would have been alive, if the child had survived, Madame would have admitted on the same day, on the day she had gone to show earlier then there was a lot of water when she was called after a month or when there was a great danger. If they had recruited, then maybe something would have happened.
Question - What do you want to improve inside the hospital?
Mother - it should be improved, it is being checked, then they should take the first recruitment, they were looking at water, they were saying that now water has become water in your stomach means that the child may not survive or can survive. Should have admitted. Do not refrain, he is in the upper hand, but he could have survived even if he had admitted. If he used to write some medicines, then he would have survived. I come home and see the house cone. At home, if you check the food and drink, it is said. Still that did not happen to him. He called a month later. I had given dispensary medicine. Even if they had the strength, they could have recruited. They did not get admitted.
Question - It happens immediately, the test means that you have to test within a few hours, if you have died today the sample is taken within a few hours on this day, then go inside the body only. The real address is that which is found out in the investigation or the reason it is not known, then it happens on the same day in the same few hours.
Mother - Madam, my child was finished since morning, meaning the madams did not say anything that you will be examined.

Question - Yes, did not say at that time because so much has not come at that time yet in the hospital. Neither in the hospital, which I told you the way of the mites, it is not yet started in the hospital, but there is a lot of effort to start it in the hospital.
Mother - And Cold Pepsi Coffee Anything?

In-depth Interviews of parents who had Stillbirth– 20

**1.1**	**Primary respondent (relation with the child)**	Mother
**1.2**	**Age of the primary respondent**	Mother- 18-20 years<br>Father- 20-25 years
**1.3**	**Other member present**	Grand mother (Nani)

Events that led to the stillbirth
Please let us know the course of pregnancy and events around the delivery/ termination.
(Please document as the event were told by the respondent)
2.1. Can you please let us know about the problems, illness and events immediately before the delivery?
2.2. Where did you go or whom did you consult for any problem during the last pregnancy?
Hospitalisation period
3. Now we would request you to tell us something about the period of hospitalisation for the pregnancy, delivery or termination? (Probe:hospital, ward, duration of stay, course of illness)
I came here at 8:45 a.m and in morning 9 months were completed. Here there is a dispensary in GP. There we get iron tablets and all. Injections and all ultrasound was done.
My mother-in law, my mother and my husband had come along with me. I was having severe pain and bleeding was there. Then I was shifted in labour room, then I ad a normal delivery. Delivery was at 10:10. I don't know what happened.
When I came then there was so much bleeding, there was so much pain so I was shifted directly….rest of house members filled the form.
4. Could you please describe your overall experience about the hospital care? (Probe:investigations, treatment given, involvement/ participation in decision making, satisfaction level, quality of care, cost of care)
We got investigations done every month. It was good. It was a government organization. (Money Spent) No money was spent there. (investigations) We used to get injections in dispensary. They used to give iron tablets and all. They used to check B.P and all. They used to check weight. They used to do entry. They got the ultrasound done.. This is that report. They told me to get the ultrasound one in 3rd Month. Rest I got my ultrasound done in 7 and a half month.
I showed it to my midwife, showed it once or twice at that time child had come down slightly.
5. Could you please describe your experience about communication by the various hospital staffs during hospital stay? (Probe:who all communicated, who was the primary communicator, nurses, doctors, other staffs, frequency of communication, mode of communication, details, completeness of information, skill of communicator, consistency in communication)
At that time no one else came to check up. When I entered there was a doctor mam. She checked me. She checked inserting her finger to check whether my body is ready for delivery or not. Then she took me directly for delivery.
Then I was made to lie down and sit down. I was having pain then normal delivery happened on it's own. Sister was not coming at time of pain. She was just saying me to push downwards. I was having lot f pain, my child came out. Then after delivery the area was cleaned and stitches were done.
(any interaction with support staff) We did not talk to them. Madam when my delivery is over, after the child birth. After cleaning they made me lie on bed just like that only. I was shivering in cold.
They did not give us blanket for long. The after a long time they gave us blanket. There were three people on one bed.
(family member present) there was no family member
(behaviour of Staff)

Mam what we have to do, we have to stay here for two or three days. How can I tell that what is missing here, how it matters, only my words are not going to matter. Mam I don't know anything, I don't pay attention to all this.

Stillbirth

We would like to know about the events around delivery and after that in the hospital.

6. Could you please tell us about the declaration of the event/outcome (stillbirth)? (Probe:who declared the outcome, cause explained, the body language and expression, preceding events before delivery, meeting with any senior doctor/member, attitude/body language/expression/skill of the communicator, expression of empathy/sympathy, language and simplicity, perceived completeness of information)

(who told you about stillbirth)

We came to know at the time when doctor had come to check.

I called Doctor for check-up. I called them "Sir Child is born" When doctor came at that time child was not crying. I understood, No Doctor had said that do you what has happened. So they showed us, I saw that a boy was born so they said that your baby is born dead. Before that also we came 3 days ago to get blood transfusion. That dispensary where I go for blood transfusion they told that you have to S Hospital for blood transfusion.

I need to make my card therefore I came here.

I told mam that I need to get blood transfusion I have come from dispensary, they have sent it. So they refused that you don't need blood transfusion. I have spoken to me in my heart. I have been sent from here by the dispensary so they refused me That there is no need of blood transfusion., So there was no transfusion, there been transfusion. (what was their way of saying) They had said it right that your child is no more alive. There is no heart beat meaning he is born dead. First it was not moving, How can we know that child was not moving.

They had already told that the child in your daughter-in-laws abdomen has already been finished 3-4 days back . but I was feeling movement f my child, I could feel the movement, had there been no movement I would have reported earlier to my mother-in-law. At the time of delivery there was no one frommy house, but there were people of hospital. Lot of people were there.

Doctors were there (who did the delivery) I don't know the name as such. There were two or three people. Gents were there, ladies doctor were also there. They were asking me, telling me to lie like this like that. To push downwards

The baby was born at 10 minutes after 10 a.m. Time is written there. Time is written I told that we can see the time. Child was dead. Chils was jumping. After sometime they told that child is dead. Child was jumping, There was pain. Bleeding was also there. Baby was orn dead. They handed over the child to us that child is no more alive you can take the body from here. They took it in next one and a half hour. I had stayed back with my daughter. There was child's Mother-In -law so we had handed over to her only .

7. What was your and your family member's reaction when you were told about the stillbirth? (Probe: what was the reaction of mother, father, other family members who were present, did someone try to console you or your family member)

Right now my mother is with me. She is giving it all right. She is giving good to eat.

8. How was your experience about the procedure after death till handover of the body to you? (Probe:processes asked to follow, documentation and papers given, interaction and behaviour of the staffs, respect for religious norms, time taken, cost/payment, assistance from the staffs)

After delivery, the baby was given to my mother-in law. They had taken in front of me to hand it over to my family. Wrapped in cloth. Then doctor gave it to mother-in law. Then mother-in law took it back home. Then only my mother-in law and husband took it home. My mother stayed with me.

(after delivery, interaction with baby)

Nani- We have been staying here since one and a half hour. Let us see if they give leave to us till evening. My body is also very weak they told me that we need to have blood. They told us to get blood transfusion, they wrote in a paper that first you go get the blood. We asked that from where we should get the blood,

you can tak e my blood. So they didn't took my blood. They told us that please give us money we will get the blood from outside. They have not come to check up since yesterday.
Till now there is no blood. Neither had they asked that are you giving some money for blood or not. Or anything related to it, they have asked nothing. Now let us see that whether they will give us leave in evening. My girl is very weak, there is swelling on whole face. It was too much yesterday. Let us see if we will go back home, there will be care. She will regain health, if she is able to get healthy then it is her fate, maybe she will be pregnant again.
Grandma- They did not give us the file. Madam was shouting they made file after I told them.
Maternal Grandmother- then file was made. Where the child, they brought the child here after that file was made. It was made in morning time meaning not in morning. They told that you are lying like that only. Report was also not seen.
They explained nothing. They were only saying that there is lack of blood. You should sign it. When blood is available so I signed it in slanting manner. She had put her thumb that child is no more alive. Nothing else was told.
9. Who all from your family and community/neighbourhood were present at the hospital and how they supported you during this period? (Probe:who informed them, who all came, what type support given)
Now I have my mother and my Moher -in - law with me.
(any support from other member) No
Post event procedures and coping mechanism
10. What all rituals and procedures did you follow after stillbirth? (Probe:body preparation, who prepared, burial/ cremation, other rituals and timings, who performed them according to the rituals)
What they did that I do not know. Last rites were done. We bury the child and adults are burned. We gave them bath. We had put some clothes.
Maternal Grandmother- We were ready to give the child, had he been alright so we would have been happy, we saw at last time that child is ok
Grandmother - We did not do anything then we took the child to our house. Yes we got it done the way it happens we got it done. We got it done yesterday. We could not keep it in house so we got it done yesterday. We got it buried we could not keep it in house so we had dug the ground and buried it deep. Like the way pandit had said we got it done.
11. How did you, your spouse (husband/wife) and immediate family members cope with the difficult phase? (Probe: any change in the psychological state, physical state, engagement in daily work, spiritual/religious activities)
12. Is/was there any change in the interpersonal relationships between you and your spouse and other family members after the event? (probe: relation between husband and wife, with other family members)
13. How did the family members support you and your spouse (husband/wife) during the difficult phase? (Probe:burial rituals, community norms/rituals, coping with the phase, financial)
14. What were the reactions/ counselling and suggestions given by the relatives/neighbours/ community after the episode? (Probe:what to do after death, following the rituals, knowing the cause of death, any other)
15. Do you think and/or discuss with your spouse/ family member(s) about the loss and related events and blame someone/something or feel guilty for the same? (Probe: with whom; how frequently; the context of discussion; contents of discussion; whom do you/others blame for the death or what about you feel guilty)
Yes madam, these doctors re the culprits. When I had come for transfusion they did not do the transfusion. Had they done the transfusion my child would have been alive. I came here three days ago. They refused. I don't know why they refused. I had a word with doctor so she refused.

16. When did father and mother (if working) returned to work after the event? (Probe: father, mother, loss of job/ resignation from job/change of job, was there any change in the mode of functioning)
17**. How your and your family's life has changed after the event? (Probe: what has changed, what is the major change, what do you care more now than before; plans for future pregnancy, any substance abuse- new behaviour or increase in the existing behaviour)**
Knowing cause of stillbirth in detail – autopsy and MITS
Many pregnancies in our community end with stillbirth due to various causes and in several instances the exact cause is not identified. We need to know the causes of stillbirth in detail. We want your views on the options of knowing the causes of stillbirth in detail.
18. What was the cause of stillbirth mentioned to you and what is your perception about the value of knowing the cause? (Probe: knowledge about the cause of stillbirth, willingness to know the detailed cause, potential factors that led to the illness/death, potential impact on the other family members or next pregnancy)
(Probe: knowledge about the cause of stillbirth, willingness to know the detailed cause, potential factors that led to the illness/death, potential impact on the other family members or next pregnancy) Because many child , they used to say that 3-4 days back. It is not moving. Had I known like the way they are saying that I has died 4-5 days back.
When I came here for delivery at that time it was moving. If we come to know the cause then it will be good.
19. In your view, what more could have been done to know the exact cause of stillbirthand the reasons causing it? (Probe:investigation, autopsy, any other)
Can't know how to know how
20. Are you aware of any method for identifying the exact cause stillbirth? (Probe:autopsy, investigations, etc.)
In several instances, doctors advise for autopsy/ post-mortem to find out the causes of stillbirth so that appropriate action can be taken to prevent recurrence of such event.
21. What are your views about the autopsy/ post-mortem for identifying the cause of stillbirth? (Probe:necessity, religious aspect, time needed, disfigurement, etc.)
If doctor asked you to perform post-mortem, then you would have Why you are not Don't know about post Mortem If doctor asked you to perform post-mortem, then you would have Madam Children post mortem what was profit Child is not devoid of postmortem. I will not be unconscious.
In some instances, autopsy/ post-mortem is not possible due to various reasons. To find out the exact Cause of stillbirth and underlying disease, some tissue biopsy (collection of very small sample using Needles, known as biopsy) and fluids (blood, urine, CSF, etc.) from the body, as done for investigation To identify and treat the diseases. But these samples are to be collected as soon as possible.
22. What are your views about collection of the tissue (like done for biopsy to diagnose diseases) and body fluid samples for identifying the cause of stillbirth? (Probe: necessity, religious aspect, time needed, disfigurement, etc.)
Yes, I am fine. Yes, I agree
23. How parents and family members can be approached for the tissue and fluid sampling? (Probe: who should approach, whom, when and how should approach; what should be told and in what detail; should some picture of the method be used to explain)
We have mother and father-in-la in house, They have to be explained. First those who all are elder in house. We will say to them only. So my mother-in law should be told.
24. In your view, what factors are important for acceptance of the tissue sampling by the parents and family members? (Probe:desire to know the cause, trust, confidence on the health care provider)
Decision making dynamics

25. In routine day to day operation who takes decision about the major activities (purchases, treatment, spending money) about the family? (Probe: involvement of the mother/women in the family, any other family member, consultation with any other member)
My father-in-law and Mother-in-law
26. During the hospital stay who discussed with the doctors/ nurses to make the decision(s) about treatment and other procedures. (Probe: father, mother, any other family member, did you/your spouse talk to someone in your family like elders or relatives to take critical decisions)
Maternal Grandmother- They did not let us enter inside they used to tell us to stay outside. I asked them that "please let me stand inside, my child is weak, No not at all , Child was born in one and a half hour, then they told after one hour around 11 they told. They did not let us in.
Grandmother- They did not let us in. The paper that they used to get our sign on, who did it. I did not do it, he did it. Our work was over in emergency department. They submitted the emergency paper. They let us submit the emergency paper. Where my child was born
Granny **-** The child is no more alive but we will do your normal delivery. The big Doctor in emergency had said.
Grandmother- We lost the child , we are there empty handed.
27. After the delivery, who decided about the burial and other post-death activities and rituals? (Probe: father, mother, any other family member; did you/your spouse talk to someone in your family like elders or relatives to take critical decisions)
My father-in law and mother-in-law
Summarization
28. Now, we are completing the interaction/ interview. Would you like to add anything?
(Behavior of doctor after delivery)
Granny-They were right, where they were born it was all right there. Here nothing happened here. Now what should we say, Nothing happened with us.

In-depth Interviews of parents who had Stillbirth– 21

**1.1**	**Primary respondent (relation with the child)**	Mother, Father
**1.2**	**Age of the primary respondent**	Mother- 25-30 years<br>Father- 30-35 years
**1.3**	**Other member present**	None

2. Please let us know the course of pregnancy and events around the delivery/ termination. (Please document as the event were told by the respondent)

Mother- My baby was fine till yesterday afternoon, and now when they gave medicine this morning, they gave medicine four times and then told that my baby is not aligned properly. When I went into the delivery room, baby's leg came out and told me that you are not aware that your child is aligned in a wrong way and you are asking for normal delivery.

I said, how I will come to know you are a doctor, you should know, the time the child's leg came out. He lost consciousness the doctors are very careless, nobody pays attention here.

Question - Yes, did not say at that time because so much has not come at that time yet in the hospital. Neither in the hospital, which I told you the way of the mites, it is not yet started in the hospital, but there is a lot of effort to start it in the hospital.

Mom - And Cold Pepsi Coffee Anything?

Question - Nothing but thank you so much.

Yes, this is what I (treatment) from the beginning, I did everything here. I got everything done here, My seventh month was going on.

So all water from water bag came out so they admitted me. it must have been 8–10 days since the time of my admission. Every day they used to say, I used when I get relieved, so they used to say that there is still time. You have to stay here for long. They kept on saying this and then it happened like yesterday…from yesterday to tomorrow. Then they took signature from my husband. Rest they told that your child is not in good health. It is like either you will survive or your child will survive. Your child cannot survive.

They were saying like this, so my husband and I signed up and said that the baby is premature, I said okay, keep it in the nursery. So they said this type of baby will not remain alive in nursery. He took the sign (information) and started giving me a dose of medicine from four and a half in the evening, there was so much pain, so much pain, I said if it is not okay then you do the operation and remove it

He did not listen to me, then they gave me pain, they gave me medicines four times every four hours, and there was lot of pain then they took me next day at five o'clock there, Then during normal delivery I kept on applying pressure and my baby was born upside down and at that point of time they are blaming me that you don't know that your child is aligned upside down, you are going for normal delivery instead of operation.

I have said, I do not know, the doctor should know, how will I know my child is aligned upside down. Yes, a Ultrasound was done here, I don't know whether they have come to now or not I had shown it. My 7th was going on, yes, 7th was not yet complete, I had come in Emergency, there was water leakage in emergency. I came in Emergency on 22nd. Yes, at the same time my ultrasound was done on priority, then they admitted me on priority that please go in labor room.

I was in the labor room for 3-4 days, I was having a water leak. So they kept me in in the labor room for 3-4 days. Yes, it had started from home (water drainage). On the 22nd, as soon as it started I came here quickly, It was going on too much. They had admitted me, then at night I was admitted for 3-4 days in labour room then they shifted me to ward number.

After 4, put me here after that, they gave me medicine, they did not even wait for that. My 7th month was not even completed, they said that whatever is there we have to take your baby out. There was sugar, but all the reports came normal, yes, yes (No child is born in 7th Month, It is born in 9th Month) So they took out very early, I had asked them.

Yes. They were telling me yesterday that mother is in danger, but they should have known that my baby is upside down. Now as soon as I went there in the delivery room, my baby's foot came out, Usually

baby’s head come out but my baby’s head came out. No one listens here, at all, there is too much carelessness here, no one listens.

2.2. Where did you go or whom did you consult for any problem during the last pregnancy?

Mother- Yes, my first baby was delivered in S Hospital. CTC / CPT repair was done by those people. They do not see it. My delivery was also done by force. They had climbed on me a year and a half ago, I have a one and a half year old daughter in my house. That too has happened here.

2.2 Mother- Yes, yes, (came to S Hospital during first baby), everything (consulted in S Hospital in last pregnancy). I get the name registered in third month.

4. Could you please describe your overall experience about the hospital care? (Probe:investigations, treatment given, involvement/ participation in decision making, satisfaction level, quality of care, cost of care)

Mother-don't know (documentation), yesterday, I got it done on plain paper, both of us Husband and wife got it done and sent it to us. Yes, on a plane, white paper, to sign there. They were writing in hindi, then I came out. No, (asked why doctor has taken sign on plain paper) because I had to get medicine so they told me to go away and get medicine. My husband had come here.

5. Could you please describe your experience about communication by the various hospital staffs during hospital stay? (Probe:who all communicated, who was the primary communicator, nurses, doctors, other staffs, frequency of communication, mode of communication, details, completeness of information, skill of communicator, consistency in communication)

Mother- Nothing comes, they see it just like that, you have been regular, they see that heart is beating normal. That’s it we can go. They never used to take care. They never used to come. They used to come at their own time. Right now there is n doctor. This and that.

6. Could you please tell us about the declaration of the event/outcome (stillbirth)? (Probe:who declared the outcome, cause explained, the body language and expression, preceding events before delivery, meeting with any senior doctor/member, attitude/body language/expression/skill of the communicator, expression of empathy/sympathy, language and simplicity, perceived completeness of information)

Mother- there was a doctor. She comes in evening, she called us inside. Me and my husband and talked to me and she took a sign from us.

Mother- Yes, the child was dead, he had said this, he had said at night, when the medicine was being given to me at night, then I asked that you are putting so many medicines for me, will my baby not get affected? She said, you don't know your baby is not inside. He told me at night, she is a doctor, here it is, a junior doctor or what (who told), she wears specs, very thin.

Mother- She was checking again and again by doing like this. She was saying that she is not able to hear the beat. Then she went into delivery room. She was not able to listen anything. She was saying ,not able to listen to anything, there is no child.

Mother- They talked very rudely.

Mother- They talk rudely. Too rude. No, not the cleaners and sweepers. Doctors, they wear blue dress, they talk in a rude manner. If we ask anything politely then they say No no , sit down, go inside. I am having pain. My child is getting out. She concentrates on her work. That’s it.

7. What was your and your family member’s reaction when you were told about the stillbirth? (Probe: what was the reaction of mother, father, other family members who were present, did someone try to console you or your family member)

Mother- they told my husband, he was shocked. He was not able to believe because my child was moving It was ok till evening. Baby was moving. Everything was fine. And as there was evening, as soon as medicine was given, no movement was there. My husband says that how it is possible. Till now baby was fine. So they said that it happens like this here my husband said that even five-five-month-olds live their lives, they put them in nursery, they do it. They did not agree, say no, no, it does not work here, your baby is no more alive. You should sign.

Mother-Mother has not yet come or she did not know.

8. How was your experience about the procedure after death till handover of the body to you? (Probe:processes asked to follow, documentation and papers given, interaction and behaviour of the staffs, respect for religious norms, time taken, cost/payment, assistance from the staffs)

Mother- gave it to my husband, They showed it to me in delivery room. After that they did not showed me. I went to ask for my child, then say, "Your child is not there, you go from here. , your child, is in the nursery. They said like that. Doctors, there is a tall doctor she told that we don't have children here, children are in nursery Go inside. Your child is in nursery. Your child is not here, go inside. I went to ask for my baby, to see him. I had given birth to baby boy.

(asked what husband did after body handed over to him)

I don't know, they gave it to me just a while ago. My child was born at 7:15 a.m and they have given it to me right now. I don't know here they took it. Right now they gave it around 3-3:30 a.m. I don't know why it took so much time for them.

(asked why it took time to handover the body)

I don't know why it took so much time. I don't know.

9.Who all from your family and community/neighbourhood were present at the hospital and how they supported you during this period? (Probe:who informed them, who all came, what type support given)

Mother- No one was inside, my husband was there, husband used to stay down, he used to come, at 4, no, my husband was there and my mother had come outside the labor room, so they do not let anyone come there. They were sitting in park.

10. What all rituals and procedures did you follow after stillbirth? (Probe: body preparation, who prepared, burial/ cremation, other rituals and timings, who performed them according to the rituals)

Mother- This I don't know (rituals after newborn death). 10. Mother- do not know where they have taken, they did not even showed it to me, packed them and gave it to them.

13. How did the family members support you and your spouse (husband/wife) during the difficult phase? (Probe: burial rituals, community norms/rituals, coping with the phase, financial)

Mother - Here there is just my sister and my mother and my husband I don't know where he is gone. (asked what support you get from family members). I get lot of support here (at S Hospital), they have just come, they did not know, so they have just come right now, they still support a lot, no matter at least you are alive we do not need anything.

18. What was the cause of stillbirth mentioned to you and what is your perception about the value of knowing the cause?(Probe: knowledge about the cause of stillbirth, willingness to know the detailed cause, potential factors that led to the illness/death, potential impact on the other family members or next pregnancy)

Mother- Yes, very (willing to know cause of death), because my baby was perfectly fine, I know the movement was all right till noon.

19. In your view, what more could have been done to know the exact cause of stillbirthand the reasons causing it? (Probe:investigation, autopsy, any other)

Mother- no

20. Are you aware of any method for identifying the exact cause stillbirth? (Probe:autopsy, investigations, etc.)

Mother- Yes, I know (post mortem), They cut your body and look inside in post mortem. I just know this.

21. What are your views about the autopsy/ post-mortem for identifying the cause of stillbirth? (Probe:necessity, religious aspect, time needed, disfigurement, etc.)

Mother- no, she was very young

22. What are your views about collection of the tissue (like done for biopsy to diagnose diseases) and body fluid samples for identifying the cause of stillbirth? (Probe:necessity, religious aspect, time needed, disfigurement, etc.)

Mother- yes (agree for MITS), she is better with MITS.

23. How parents and family members can be approached for the tissue and fluid sampling?(Probe: who should approach, whom, when and how should approach; what should be told and in what detail; should some picture of the method be used to explain)

Mother- Whose (sample) she wants. He must have taken the child along. Yes this point of view is good. (parents view in willingness to know the cause). (whom) He is my husband. (If her husband can take decision on his own or need elders support) No, he can take it. It is not like that. (when) when we are

admitted (counseling at admission). (awareness through T.V advertisement for MITS) Yes, it would be correct.

26. During the hospital stay who discussed with the doctors/ nurses to make the decision(s) about treatment and other procedures. (Probe: father, mother, any other family member, did you/your spouse talk to someone in your family like elders or relatives to take critical decisions)

Mother- husband

27. After the delivery, who decided about the burial and other post-death activities and rituals? (Probe: father, mother, any other family member; did you/your spouse talk to someone in your family like elders or relatives to take critical decisions)

Mother- My husband will take them only (decision to perform rituals). Yes, I will do it with my family. (asked if in joint family) No, we live separately right now no one is there yet.

28. Now, we are completing the interaction/ interview. Would you like to add anything?

Mother**-** No mam, nothing else, but I am sad that my baby has become like this and I don’t want to say anything.

In-depth Interviews of parents who had Stillbirth– 22

**1.1**	**Primary respondent (relation with the child)**	Mother
**1.2**	**Age of the primary respondent**	25-30 years<br>(Father 30-35 years)
**1.3**	**Other member present**	None

Events that led to the stillbirth
2.1 Can you please let us know about the problems, illness and events immediately before delivery?
Yes, nine months. My date was for January 4… (any problems, illness) Trouble is that my two conceptions had been spoilt. One was of three months and other last one for two months. Then I had undergone the last treatment in private then it stopped in between. it was a complaint of heartbeat too… ..I had no problem
I remain alright (any weakness) No, all this does not happen…since stating it was fine.
2.2 Where did you go or whom did you consult for any problem during the last pregnancy?
There was a doctor in private. He used to give injection. A doctor in private. I was not able to conceive so they gave us medicine.
Hospitalization period
3. Now we would request you to tell us something about the period of hospitalization for pregnancy, delivery or termination?
It has been two days, today and tomorrow, total four days. No, first they went to jafarpur. I had card there, they sent us to deen dayal. They delayed for us. They told that you will get the treatment there and they dropped us there.
4. Could you please describe your overall experience about the hospital care?
There was no treatment here. If you will give your blood then treatment will be done. We are giving you blood. We can get the treatment you will not get the treatment. Yu will not give blood, one has to give blood.
I had a problem, everything is not right, patients are dying twenty four hours, three days have passed Away children are lost…. Four days a doctor should be there, why is he missing when he knows?
If delivery is to be done, get it done because inside I am also suffering.
My blood loss has been there since the beginning…. Blood does not live in me… Where once I collected the file now the same thing will be done once they are finished
5. Could you please describe your experience about communication by the various hospital staffs during hospital stay?
She also talks like this loudly brings blood it will be done right-away.
So, it's ok when the blood will come then only it will happen.
Stillbirth
6. Could you please tell us about the declaration of the event /outcome?
(Who declared the outcome) They have told me here I came to know here only. Person who did the Ultrasound, he only told me. The child that has been spoilt has to be out either through medications or operation.
7. What was your and your family member's reaction when you were told about the stillbirth?
Yes, they told him what to say and then they said nothing
8. How was your experience about the procedure after death till handover of the body to you?
9. Who all from your family and community / neighborhood were present at the hospital and how they supported you during this period
I was there, my sister-in-law and my husband was there. She is our niece. This is not the kind of treatment. Today 24hours and since 4 days there was no treatment. It doesn't happen like that within four days. Poison is also there that spreads. Poison also spreads, the government does its work, they do their own work.

Post event procedures and coping mechanism
10. What all rituals and procedures did you follow after stillbirth?
N/A
11. How did you, your spouse (husband / wife) and immediate family members cope with the difficult phase?
N/A
12. Is/ was there any change in the interpersonal relationship between you and your spouse and other family members after the event?
N/A
13. How did family members support you and your spouse (husband/wife) during the difficult phase?
N/A
14. What were the reactions / counselling and suggestions given by the relatives/neighbors/ community after the episode?
N/A
15. Do you think and /or discuss with your spouse/ family member about the loss and related events and blame someone/something or feel guilty for the same?
N/A
16. When did father and mother (if working) return to work after the event?
N/A
17. How you and your family's life have changed after the event?
N/A
Knowing the cause of stillbirth in detail – autopsy and MITS
18. What was the cause of stillbirth mentioned to you and what is your perception about the value of knowing the cause?
He said your BP is high, because of this my BP is not high, it is necessary to know that what exactly happened. One should know that exactly what happened. You should take care.
19. In your view, what more could have been done to know the exact cause of stillbirth and the reasons causing it?
How would I know this…. He would know this…. have you heard about autopsy No
Yes I have heard that everything comes in it, now I do not know it
Sister- in- Law - All the bodies are examined in the postmortem when nothing is known,
Mother / Patient - In the case of the police, my husband has also called
He had called them, now if they are not getting treatment, then what to do?
We will remain for four days, if we poor people had more money, we would not have got it ourselves
Even the need to eat in private
20. Are you aware of any method for identifying the exact cause stillbirth?
How would I know this…. He would know this…. (Have you heard about autopsy) No,
Yes, I have heard that everything comes in it, now I do not know
Sister- in- Law – Whole body is examined in the postmortem when nothing is known,
Mother / Patient – We do it in police case. My husband has also called police people
Now they are doing treatment then what to do. We cannot sit there for days, we are poor people had we be having money more money, we would not sit here for long. For four –four days. We are poor people had we be having money we would have got it done in private.
21. What are your views about the autopsy/post mortem for identifying the cause of stillbirth?
No, my husband will tell.
22. What are your views about collection of the tissue and body fluid samples for identifying the cause of stillbirth?
What to know in a small child…. Well, let's take injections and see, it is right?
23. How parents and family members can be approached for the tissue and fluid sampling? (Should some picture of the method be used to explain?)

Video, photo is not with us. This has to be done by you.
(How should approach) This is how I am able to understand, you are talking to me so I am able to understand. I am there he is there, my husband and my mother-in-law.
24. In your view, what factors are important for acceptance of the tissue sampling by the parents and family members?
Decision making dynamics
25. In routine day to day operation who takes decision about the major activities about the family?
My husband sees it. My husband only takes it.
26. During the hospital stay who discussed with the doctors / nurses to make decision about the treatment and other procedures?
My sister-in-law
27. After the delivery, who decided about the burial and other post- death activities and rituals?
Now, we are completing the interaction/ interview. Would you like to add anything?

List of In-depth Interviews-Community Leaders

Sl. No.	ID	Occupation
1	Participant 1	Pradhan (Area chief)
2	Participant 2	Resident Welfare Association president
3	Participant 3	Pradhan (Area chief)
4	Participant 4	Pradhan (Area chief)

In-depth Interviews-Community Leader-01

1. **Basic demography**
   1.1. Designation– Pradhan.
2. **Care seeking practices**
   2.1 Where do people from your community/ locality usually go or whom do you consult for healthcare (all types of patients/illnesses)?
   We have two hospitals nearby, one is MB and other is S hospital where mostly people go. Only few of them go to a private doctor in our own locality. Those who have known some private doctors prefer to go to them.
   2.2. Which all health facilities are usually accessed? Which ones are mostly accessed?
   Few people go to S hospital who wants to take major treatments for technology specialist doctors are only available in S hospital. Those who have minor health issues, like cough or cold , they go to the nearby available doctor in our locality and take treatment from there only.
   2.3. Which health providers are usually accessed? Which ones are mostly trusted?
   Most people believe in S hospital, A hospital, we believe in the government hospitals.
3. **Where do people from your community/ locality usually go or whom do you consult during illness of children and new-born?**
   In that case also, all go to there only (S Hospital). Child specialist doctors in private, people go there also. few people have fixed private doctors, one is Dr. M, Dr. AM, people also visit her, she is child specialist. Then there is Dr. P, there is Dr. K, like wise there are many other famous doctors where people go.
   3.1. Which ones are usually accessed for non-critical illnesses and why?
   In minor health issues go to private only.
   3.2. Which ones are usually accessed for critical or severe illnesses and why?
   In major health issues all go to S Hospital big hospital. because at last small hospitals refer to S Hospital only due to lack of facilities. When situation is not in their hand then they refer to S Hospital or A hospital only, telling to go there only.
4. **Where do people from your community/ locality usually go or whom do you consult during pregnancy and delivery?**
   4.1. Which ones are usually accessed for routine pregnancy check-ups?
   In that case, few go to nearby locally available Dai (Traditional Birth Attendent) for check up. Rest of them, mostly people go to S Hospital, mostly people started going to S Hospital. They do their registration and go for delivery there only. Here we have ASHA workers also.
   4.2. Which ones are usually accessed for delivery or pregnancy with complications?
   During the time of delivery, it depends on the situation when case is complicated then most people go to Dai for delivery means when it is emergency and people are not able to go to hospital. In complicated situation they are bound to go to hospitals only.

Illnesses and causes of death

5. **In your view, what are the common illnesses and causes of child death in your locality?**
   In our area, most common are loose motion, diarrhoea and vomiting. Cough and cold are now a days more common and accordingly no child has dengue like that, not even malaria from long time. Cough and cold are more common.
6. **We are trying to identify the causes of death in children, so that something can be done to prevent and reduce the number of deaths in children.**
   In your view, would this information be valuable?
   (If valuable/ useful, why? If not, why not?)
   This is very important if you people are doing such kind of activity, then it is very good this should happen. this is very good. The children who are dying because of any disease they will get treatment, it is very important. Someone's life will be saved according to me it is very important.

Knowing cause death and implications of procedures

In some cases the children die before even the exact cause of illness and death is identified/ investigated. In such cases, doctors suggest autopsy/ post-mortem to identify the cause(s) of death.

7. **How autopsy/post-mortem is viewed by you and community members for knowing the cause of death? (Probe: explore the views, concerns and believes in the community regarding autopsy)**
   I don't know anything about post-mortem. I believe the one who die lose their life and then the family are only to be consoled. But for the next child, if we wish to have no disease then it is important to know the cause of death. Suppose, the infection of this child does not spread to the other child, so it is important to know the cause of death which will help other parents to become more active. This is also better, it is post mortem or it is autopsy which you have said, according to me, both are better, however, autopsy is far better than disfigurement of the body. The people here believe that if my child is dead, we will not go for disfigurement of the body. The child is dead, it is alright, these people will have this kind of perception, as long as I understand and know them.
   <death rituals after PM> Yes, there is a difference as someone wants to give shower to the child, wants to pray for the child, few people want to bring the child home. They say that post mortem was done to the child, so take the child straight from the hospital to the cremation centre. In that case child is not given shower and there is a short prayer, lots of changes when you do post-mortem. Everybody has their own perception according to their own religion. Neither we can give shower to the child nor we can shave his/her hair. There are many people who do lots of things according to their own religion I can't say about everybody. Everybody has their own religion. Some are Hindu, some are Muslim, some are Sikh, some are Christian, some are sardar, means everyone has their own religion. They do according to their own religion. So, they think that now no rituals, no prayers can be done for the child, no shower can be given to the child in post mortem. So these changes come in post-mortem, according to me.
8. **In some instances, autopsy/ post-mortem is not possible due to various reasons. To find out the exact cause of death and underlying disease, some tissue biopsy (collection of very small sample using needles) and fluids (blood, urine, etc.) from the body of the child after they die, as done for investigation. But these samples are to be collected as soon as possible after death.**
   How do you view minimal tissue sampling procedure as a method for knowing the cause of death? (Probe: explore the views, concerns and believes, religious aspects, comparison with autopsy)
   This is far better, for community also it is better, people will agree. For once they will not agree for that (post mortem) but for this (MITS) they will agree.
   <about MITS> Here people should not have any objection for injection according to me, they will not have any problem. At least they will get to know the cause of death of the child, about the disease. It does not happen with the second child and so for this they should agree and they will agree.
9. **How the parents/ families are likely to view the minimal tissue sampling procedure as a method for knowing the cause of death? (Probe: anticipated views, concerns and believes, religious aspects, rituals, comparison with autopsy)**
10. **How the parents/families of a child/newborn who has died, should be approached by the hospital doctors/staffs for the minimal tissue sampling procedure?**
    (Probe: who, when and how and whom should approach, who can be key influencer)
    Yes in that situation parents are not able to take decision. The Pradhan of the colony might be called, elderly people of the village might be called, one or two people from the village might be called who can give the parents support, and make them understand. People understand for animals, and it is a case of a human being they will understand. Everyone faces sorrows, I agree, parent’s situation is bad at that time but if we talk then all parents agree. Parents are not in their senses at that time but elderly people of the house can be approached by the Pradhan of the colony and make them understand. If the Pradhan talk to the father’s elder and younger brother then they can understand everything. Slowly with time everyone agrees. That is done, they agree, it does not matter. I feel, it is my personal feeling if you want to do that test without letting the parents know also you can do it. Like I said, I am the Pradhan of the area, so people will agree with me. Two to four elderly people may be approached we trust, they can be requested to come then the

parents will definitely listen. Two to four elderly who are our father's age, if we take them along with you all then they will definitely agree.

11. **In your view, what factors are important for acceptance of the minimal tissue sampling by the parents and family members? (Probe: desire to know the cause, trust, confidence on the health care provider, who can be key influencer)**
    People wants to know the reason why and what is the cause of death, from which disease, many people say why the child died, may be due to cold. Many people say due to Pneumonia and thousand more disease which were not known previously. In this era also, many people in a family use to cut beard from a single blade. Many times skin is cut during the process, and all members, like father, grandfather and uncle used the same blade. If anyone had cut his skin, and if there was blood infection, we never get to know. Later we come to know, because of the cut the infection got spread. But that will lead to cancer or AIDS, through blood, we never knew that much. But in the present time, we have facilities, so we should know the reason. Nowadays, people believe in these things that cause of death should be known. I think they will get to know the cause.
12. **In your view, what would be the most suitable method(s) of educating the community about the minimal tissue sampling? (Probe: medium, methods, place and target audience)**
    Through video, my community people will understand better.
13. **In your view, what are the challenges for conducting the minimal tissue sampling at the hospitals? (Probe: acceptance by community and families, religious aspects, burial/cremation related)**
    Challenges will be that if father and mother are in this situation then they will not understand what to do and disagree. But see most of the work have challenges in the beginning and if you want to do this type of work, then problems will definitely arise. Negativity is everywhere, but to bring out positivity we have to face negativity. If we make the elderly people of the village understand about it, then they will agree for this.
14. **In your view, how the community leaders/influencers like you can contribute/ participate in such activity? (Probe: who or what is it, what did you do in this context)**
    Whenever you will call me for help, whatever possible from my side I will contribute. We will provide you the necessary help.
15. **Any other specific comments/ observations.**
    I want to tell you that the work you are doing is very good I will support you when you will take the work forward. We will make people understand and they will definitely understand that. Country is changing and people are now more understanding and intelligent by nature.

In-depth Interviews-Community Leader-02

1. **Basic demography**
   1.1. **Designation/ Role-** RWA President
2. **Care seeking practices**
   2.1. **Where do people from your community/ locality usually go or whom do you consult for healthcare (all types of patients/illnesses)?**
   Those who are in government, they go to CGHS, those who are government employees. In private there are different levels, people who are little well off go to somewhat good hospitals, private hospitals like there is B Hospital and another one is S Hospital.
   2.2. **Which all health facilities are usually accessed? Which ones are mostly accessed?**
   Among the private ones, Dr NP is the most famous; few people go to government, like the M hospital. Depends on the capacity (financial status) of the person if someone is not serious then they go to the local doctor.
   2.3. **Which health providers are usually accessed? Which ones are mostly trusted?**
   Nil
3. **Where do people from your community/ locality usually go or whom do you consult during illness of children and new-born?**
   3.1. **Which ones are usually accessed for non-critical illnesses and why?**
   If the child is not serious, then they go to the local doctor. And the new-borns, who are born in the nursing home, usually the baby's mother prefers there only so they take the baby there. And if there is someone in normal condition, then they take the child to the local doctors who are here. There are doctors nearby too, also from A Hospital.
   3.2. **Which ones are usually accessed for critical or severe illnesses and why?**
   If the child is serious then in the nearest hospital, mostly people go to private hospital.
4. **Where do people from your community/ locality usually go or whom do you consult during pregnancy and delivery?**
   4.1. **Which ones are usually accessed for routine pregnancy check-ups?**
   In the case of pregnancy, it is all different. Most of them go to private hospitals, and those who prefer government, they go to the government hospitals only. Some people get registered in government in advance otherwise they become members in private hospitals. They remain members till the delivery time. People have different choices of locations.
   4.2. **Which ones are usually accessed for delivery or pregnancy with complications?**
   In that case, where names were registered or where they were already showing, they go there.

Illnesses and causes of death

5. **In your view, what are the common illnesses and causes of child death in your locality?**
   Diseases are mostly cold, cough, loose motion and stomach diseases, and rest are not that serious problems.
6. **We are trying to identify the causes of death in children, so that something can be done to prevent and reduce the number of deaths in children.**
   In your view, would this information be valuable?
   It is very much important, but in our colony, there is zero percent death of children. But the colonies that are little away from ours, where people are from the lower category, death of children happen. Deaths happen there because their income is less. If children suffer from major illness, they are not able to spend much on the treatment, for this reason they take the child to a government hospital. If the doctors write for any test, they do nothing, they don't understand the need and the child die. Then they will have another child but will not go for the tests (autopsy). So, such kinds of problems are there, but where there is an educated colony, whose source of income is little better there the cases are less or there are no cases at all.

Knowing cause death and implications of procedures

In some cases the children die before even the exact cause of illness and death is identified/ investigated. In such cases, doctors suggest autopsy/ post-mortem to identify the cause(s) of death.

7. **How autopsy/post-mortem is viewed by you and community members for knowing the cause of death?**

(Probe: explore the views, concerns and believes in the community regarding autopsy)

Post mortem is done in cases such as accidents but not done for knowing the cause of death. If they are familiar about the cause of death then they do not go for it (autopsy/PM). In many cases, the reason is already known since the child is admitted. <Concerns and believes> People according to me will not understand for post mortem. It is that our child has left us in front of our eyes, mostly they know the reason of death. So there is no role of doing post mortem here. Where there is murder, there is an accident, in those cases it is the formality to do PM, or where there is a police case, people go for PM and there it is necessary also, it is important. But for these kinds of deaths (disease related), then they do not do post mortem. People actually do not want to do post mortem because on one hand the child has died and then there will be disfigurement of the body, in our words it is called ‘Gijan’ (handling badly) so people do not believe in it.

8. **In some instances, autopsy/ post-mortem is not possible due to various reasons. To find out the exact cause of death and underlying disease, some tissue biopsy (collection of very small sample using needles) and fluids (blood, urine, etc.) from the body of the child after they die, as done for investigation. But these samples are to be collected as soon as possible after death. How do you view minimal tissue sampling procedure as a method for knowing the cause of death? (Probe: explore the views, concerns and believes, religious aspects, comparison with autopsy)**

   Maybe, for this thing (MITS), they will agree. As there will be no disfigurement of the body, so they will agree for this. But for this, your organization will first have to bring this into the knowledge of the people though door to door. This will happen here only, if they think they will search for you and call you then that will not happen. If you make them aware that you call on this number, like helpline numbers are available for other things, then you will get some percentage of people. In that also, involvement of a particular hospital is necessary, they will only process it. <Religious aspect> if there is no disfigurement of the body, then there will be no religious objections.

9. **How the parents/ families are likely to view the minimal tissue sampling procedure as a method for knowing the cause of death? (Probe: anticipated views, concerns and believes, religious aspects, rituals, comparison with autopsy)**

   Parents can do door to door campaigns, or you can do a camp or if your organization does any program, with the help of RWAs or an awareness camp. <Religious aspects, rituals> Like I said before, they will agree as there is no disfigurement of the body and the sample will be taken through syringe, and then there should be no problem.

10. **How the parents/families of a child/new-born that has died, should be approached by the hospital doctors/staffs for the minimal tissue sampling procedure? (Probe: who, when and how and whom should approach, who can be key influencer)**

    These things should not be at the time of death in the hospital but before the death, like it will have to be campaigned in every society before death. Facilities like you are talking today, you can first tell this door to door that we have such facility, and you will be benefitted to know the cause of the child’s death. In future, these things will not happen to the other children, like this you will have to make people aware by campaigning. If they feel those who are intelligent and educated people, or those who believe this is right, those people will participate. If they are given a help number, then maybe they can call themselves. If you say this will work then you inform me.

11. **In your view, what factors are important for acceptance of the minimal tissue sampling by the parents and family members? (Probe: desire to know the cause, trust, confidence on the health care provider, who can be key influencer)**

    Parents will agree only if they see any benefit, the parents will want that the same disease/problem does not come in their other children and that the children remain healthy in future.

12. **In your view, what would be the most suitable method(s) of educating the community about the minimal tissue sampling? (Probe: medium, methods, place and target audience)**

    Like I said by campaigning telling them door to door or with a help line number. But you have to start all this before the death, where you can include the mother, father, grandparents of the child.

13. **In your view, what are the challenges for conducting the minimal tissue sampling at the hospitals?(Probe: acceptance by community and families, religious aspects, burial/cremation related)**
    Challenges It is all your efforts that how much you can convince them, how is your team, what is your main purpose you will only have to do it. And the rest will be known only when it starts. Rest, by going door to door only we will get to know what people's perceptions are. By sitting over here or as a society president we can't tell like that.
14. **In your view, how the community leaders/influencers like you can contribute/ participate in such activity? (Probe: who or what is it, what did you do in this context)**
    We can help in such a way that we can grant you permission, rest campaigning can be done or you bring any pamphlet, then you can distribute it yourself, otherwise we will get someone to distribute it. We have certain charges for it, Rs.500-1500, then we can get it circulated, door to door or whatever way we can help in it.
15. **Any other specific comments/ observations**.
    What facility you are giving will have to be highlighted, people want free service, if you are giving free service then tell the people where they can join.

In-depth Interviews-Community Leader-03

1. **Basic demography**
   1.1. Designation/ Role- Pradhan
2. **Care seeking practices**
   2.1. **Where do people from your community/ locality usually go or whom do you consult for healthcare** (all types of patients/illnesses)?
   Like few people have made their cards so they go there. Delhi government has made Mohalla clinic (area wise local clinic) so they go there. If there is serious issue then they go to S hospital, but for small problems they go the small local clinics.
   2.2. Which all health facilities are usually accessed? Which ones are mostly accessed?
   2.3. Which health providers are usually accessed? Which ones are mostly trusted?
   Clinics are not like MBBS Clinics where there are educated doctors. Few clinics are also with quacks who open their small clinics, all the clinics are like that. P clinic is little big where MBBS doctors sit. Means those who are intelligent kind of people they go there, but those who are less educated, like normal, who try to save money they take medicines for 20-30 rupees.
3. **Where do people from your community/ locality usually go or whom do you consult during illness of children and new-born?**
   3.1. Which ones are usually accessed for non-critical illnesses and why?
   Means understand that when more than 80% of the condition or health starts worsening, then they go to Safdarjung. Rest, there are camps, like for eyes, or for sugar, or for BP, etc, for those things people come. There is a government organization named SB, there are such organizations two to four that keeps on visiting every month, such camps are being organized for check-ups.
   3.2. Which ones are usually accessed for critical or severe illnesses and why?
   If the child is more ill, then they take him to S Hospital or M Hospital.
4. **Where do people from your community/ locality usually go or whom do you consult during pregnancy and delivery?**
   4.1. Which ones are usually accessed for routine pregnancy check-ups?
   There is a government dispensary, first they visit there normally it continues till 6–7 months. During delivery time they go to S Hospital or they go to a hospital in MN.
   4.2. Which ones are usually accessed for delivery or pregnancy with complications?
   Illnesses and causes of death
5. **In your view, what are the common illnesses and causes of child death in your locality?**
   There is less cleanliness here and the drain is dirty too. It is very much dirty from inside. This area is still clean, if anyone comes, there are many small lanes where there is no sanitation. Illness is due to non-cleanliness. <causes of death> Most people whose stomach gets upset quickly, have Diarrhoea, mostly people become ill due to this. Few people have less issues but the rest face vomiting or Diarrhoea.
6. **We are trying to identify the causes of death in children, so that something can be done to prevent and reduce the number of deaths in children.**
   In your view, would this information be valuable?
   (If valuable/ useful, why? If not, why not?)
   This will be very important will be very good. This is because suppose this is happening to someone, you sit and discuss with them, you ask them how it happened, what happened, what health issue was there actually, earlier what was the problem, what was the matter, where you went, then you will know that due to these reasons health got worsened. If you had done this, then the child might have survived. If you gather such information, then through any source you can pass this information to the people. When the child does such-such things, gives such hints then at that time you should do these things to make it under control. There should be doctor's involvement to make that under control. Suppose someone is getting sick due to vomiting, then at first people with Ayurveda knowledge in the village is consulted. They used to eat vegetables, they used to eat fresh things, they used to eat food without any chemicals, meaning their routine was good. Nowadays everything has chemical in it, I am saying that 40% of the vegetables have chemical in it, according to me 20% vegetables coming from the banks of Jamuna river has poison in it, chemicals used for the dirty drains. That is why we have noted two things that the

vegetable that comes from OM and the vegetable sold here, and some vegetable that comes from F Old Market, its freshness is different, meaning its taste is different, the energy of the person eating it is also good, and the vegetables coming from the OM from Yamuna river, because of that people have health issues. So, if you share this information with people then people will be worried that they are eating vegetables with chemicals. If all these things help to control their health, this is a very big job, it is very important. That day a vegetable seller came with such a thick brinjal, first time I saw it in my life. He also brings fresh cabbage. If you come in the evening, then I will be able to give you demo and a vegetable seller comes from Faridabad, and the rest of the vegetable comes from OM, just pick one cabbage you will know yourself, how much taste is there in it. So this is very important. What we are eating the most, we are eating grains, from that we are getting the problem.

Knowing cause death and implications of procedures

In some cases the children die before even the exact cause of illness and death is identified/ investigated. In such cases, doctors suggest autopsy/ post-mortem to identify the cause(s) of death.

7. **How autopsy/post-mortem is viewed by you and community members for knowing the cause of death?**

   (Probe: explore the views, concerns and believes in the community regarding autopsy)

   See what happens, there are some emotions and people will never like the disfigurement of the body. I am saying this in this way because those people will think the same way. We say that there should be post-mortem but those who are less educated mostly it seems that they do not want their child to get disfigured despite that the child is no more. It is a negative point for them (PM), it is bad for them and they will never want it. Mostly, in less than one percent cases it happens that due to someone else the child has died, fed something wrong, otherwise death happens only when the child is not well. So people do not like the child's post-mortem.

8. In some instances, autopsy/ post-mortem is not possible due to various reasons. To find out the exact cause of death and underlying disease, some tissue biopsy (collection of very small sample using needles) and fluids (blood, urine, etc.) from the body of the child after they die, as done for investigation. But these samples are to be collected as soon as possible after death.

   How do you view minimal tissue sampling procedure as a method for knowing the cause of death?

   (Probe: explore the views, concerns and believes, religious aspects, comparison with autopsy)

   <comparison with autopsy>

   That you can do, it is very right, it is great. If we explain to people in the manner that it is important to know the reason, so that other children can be saved, then maybe they will agree. They will accept MITS.

9. **How the parents/ families are likely to view the minimal tissue sampling procedure as a method for knowing the cause of death?**

   (Probe: anticipated views, concerns and believes, religious aspects, rituals, comparison with autopsy)

   What people think will be the same and will go on like that, it takes some time, we will have to explain this at that time, have to make them feel that they will be benefitted if they do check up and will know the reason. We will have to explain for that thing, because otherwise they will not agree directly, will have to explain.

10. **How the parents/families of a child/new-born who has died, should be approached by the hospital doctors/staffs for the minimal tissue sampling procedure?**

    (Probe: who, when and how and whom should approach, who can be key influencer)

    <how to tell them>

    Those who know them, who are in touch with their family but not a member of the family. Suppose a child has died, it should not be the child’s mother, father, grandfather, grandmother, uncle, brother. But some friend, some neighbour, you should explain to these people. You explain to them that may be someone has done something wrong with the child like black magic, you should know the reason of death of the child, for that we will have to do this. So you please explain to your mother and father, then you can order them to go for the procedure. Suppose in the compound, we have chairs, whenever someone comes from outside, any person, any madam,

they sit there. Then one or two people definitely come to ask. By seeing that others will also go to ask, like this 10-20 people will be there. Then here we can promote and tell people, advise them that why we have come, why we are sitting, then we will tell about that thing. Those who liked it will get it done, those who do not like it will definitely ask what is it and someone will tell them why we have gathered here, whether they do it or not. Madam as you are saying that according to the procedure when a child has died, only at that time you will have to do this procedure, right? So, you are talking about that time, what should be done in that situation? You are strange for them, when you explain this to them, why would they agree with you, you even say that you are a doctor, then also why would they agree. They will be in anger, crying in tension and you will be doing the testing procedure in that situation. They might be angry on you on what you are saying. But suppose there is such a problem with me, I know him, I respect him, if he comes and explains me that son you should do like this, then even if I am crying but I will listen and do that. I will think that they are right, then I will say yes brother, please do it.

11. **In your view, what factors are important for acceptance of the minimal tissue sampling by the parents and family members?**

    (Probe: desire to know the cause, trust, confidence on the health care provider, who can be key influencer)

    Means it has two reasons, one that people might go for check-up by to assure that there is no black magic done on their child, because of someone else my child is not dead. The other reason they can think that this has happened to my child but should not happen with anyone else. So, to know why it has happened so that it can be prevented. It has happened to me; it should not happen to others. But to get these done, only they will agree who have faith in it.

12. **In your view, what would be the most suitable method(s) of educating the community about the minimal tissue sampling?**

    (Probe: medium, methods, place and target audience)

    It depends how much time you have, and in how much time you want to explain. If you explain through social media, or you are advertising, promoting, then those people will come in a small range who are little educated, who know how to run a phone, who are involved in social media. But this issue is not for these people, because they are a bit intelligent, they are educated and for them this problem is negligible. This problem is for someone who does not have any support from anywhere, those who do not have the medical knowledge, who does not know what should be done, who is poor, who does not have money to get treatment, problems are for these people. They are emotional people, meaning they are sensitive, even get angry on small things. Even today, suppose if we say to them that some people have come, so they will not sit on the chair, they will sit down. These types of people have these problems because they do not want to understand things. If you are discussing with us, you should make a group of 5-10 people. Suppose think that this is a compound (Chowk), and you asked the Pradhan that we have to explain this for 10 mins, please call all the ladies and gents. We will sit with them and will explain the whole thing. So, you can make a group of 10-20 people and compound wise we can ask people to gather so that everyone attaches with you. This would be the best way to make someone understand.

13. **In your view, what are the challenges for conducting the minimal tissue sampling at the hospitals?**

    (Probe: acceptance by community and families, religious aspects, burial/cremation related)

    The challenge will be that I will not get it done for the children, will be angry, will be crying, such feelings will come from inside. There are many other people of different mentality, you know science, it is not that they are of wrong mentality. But we have two types of mind, conscious mind and subconscious mind. What our subconscious mind absorbs, it is very difficult to forget things. And those who are of that type, like that of subconscious minds, are very powerful. Our people who are little educated, their conscious mind works, but those who are illiterate type, they are stubborn type people and their subconscious mind is working more. Once things are fixed in their mind, they do not want to understand anything else, these people are of this mentality so we face problems.

14. **In your view, how the community leaders/influencers like you can contribute/ participate in such activity?**

(Probe: who or what is it, what did you do in this context)

You can tell them, those who understood you, those who listen to you and understand you, those two-four-ten people can be first made understand. We can say that doctors are coming, your children will not feel sick, no tragedy happens with your children so in order to prevent this, these madams will explain you something. So like this way we can make them sit and explain them.

15. **Any other specific comments/ observations.**

I want to say one thing you should explain this to people before there is some accident or tragedy. Suppose we spread this. We get to know that by chance something happens with someone, then we will definitely do it because you have explained it to us. I would like that if these things can be started even before some accident happens, so that everyone knows. Then the difficulties that you were saying (for consent) may not come or that will be reduced. Means your number will be there, you will have a meeting, if someone faces some problem then we will have to call you, otherwise you will not know about it (death of any child). Suppose something happens in the evening, they do not want to hold on and want to leave soon for cremation, means people bury soon so that there is no crying in the house. So, for this reason if we have your number then you can be informed.

In-depth Interviews-Community Leader-04

1. **Basic demography**
   1.1. Designation/ Role- Pardhan
2. **Care seeking practices**
   2.1. **Where do people from your community/ locality usually go or whom do you consult for** healthcare (all types of patients/illnesses)?
   They go either to S Hospital, or to private.
   2.2. Which all health facilities are usually accessed? Which ones are mostly accessed?
   Most of the people visit government hospital because of money problem but if children are taken to private, they charge 30-40 thousand.
   <any clinic nearby> for cough and cold they go to private. AS dispensary is there, where the treatment is free and if they do not have any medicines, then they write it to get it from outside.
   2.3. Which health providers are usually accessed? Which ones are mostly trusted?
3. Where do people from your community/ locality usually go or whom do you consult during illness of children and new-born?
   3.1. Which ones are usually accessed for non-critical illnesses and why?
   Small children are taken to the nearby doctor we have dear doctors here to visit. There are 3-4 quacks here, everyone goes to them. Few visit doctors in VV too.
   3.2 Which ones are usually accessed for critical or severe illnesses and why?
   Children are also taken there only in NP at MB and if they disagree then go to S Hospital. In MB they give good treatment.
4. **Where do people from your community/ locality usually go or whom do you consult during pregnancy and delivery?**
   4.1. Which ones are usually accessed for routine pregnancy check-ups?
   There is no such government midwife here, but one or two ladies are there in the area. The check-ups are mostly done by the Asha worker, she only roams around and have gathered all details of everyone. There is also a CGHS dispensary, so most of them go there. All Asha workers here with their respective groups, get a free ambulance then they get it through S Hospital.
   4.2. Which ones are usually accessed for delivery or pregnancy with complications?

Illnesses and causes of death

5. **In your view, what are the common illnesses and causes of child death in your locality?**
   The reason is the negligence of the parents, they go to work leaving the child behind, even the ladies start working when they have three-day child. They either give their child to the neighbours or to some other small child to take care of them, as they have to work.
   <What is disease> Not all children get sick 1-2% gets ill. Mainly there is fever which sometimes gets to the head, but not diarrhoea like problems.
6. **We are trying to identify the causes of death in children, so that something can be done to prevent and reduce the number of deaths in children.**
   In your view, would this information be valuable?
   (If valuable/ useful, why? If not, why not?)
   Many times the reason is not known, it is tough to understand what they are telling about. It is not known what time they fall ill, sometimes fever, sometimes they get diarrhoea, but it is not known. It is very important to know the reason, if we know the reason, then we will get treatment according to that. In future there should not be any disease, for that it is necessary to prevent.

Knowing cause death and implications of procedures

In some cases the children die before even the exact cause of illness and death is identified/ investigated. In such cases, doctors suggest autopsy/ post-mortem to identify the cause(s) of death.

7. **How autopsy/post-mortem is viewed by you and community members for knowing the cause of death?**
   (Probe: explore the views, concerns and believes in the community regarding autopsy)

This (post-mortem) is wrong. It is wrong as the doctor has given full treatment for the disease on his behalf, but if it happens for some reason, then it is not the doctor's fault. There is nothing wrong to get the post-mortem done. It is not that the doctor has given some wrong injection to the child. Post-mortem is not the right thing to do on the child. <Religious aspect> We believe in all religions. There is nothing like that. <reason for not going for PM> Firstly, the child has died and again they are disfiguring the child and ruining his body. The child will not come back by doing post-mortem, and they take out and keep his body parts on the side. <Believes in the community> We think it is wrong, everyone says not to get post-mortem done. People who are crazy will do post mortem. What will they get from post mortem, nothing, nothing is going to come back.

8. *In some instances, autopsy/ post-mortem is not possible due to various reasons. To find out the exact cause of death and underlying disease, some tissue biopsy (collection of very small sample using needles) and fluids (blood, urine, etc.) from the body of the child after they die, as done for investigation. But these samples are to be collected as soon as possible after death.*
   How do you view minimal tissue sampling procedure as a method for knowing the cause of death?
   (Probe: explore the views, concerns and believes, religious aspects, comparison with autopsy)
   What was the reason, because of which disease the child has died? The child should be treated when he gets sick, then only the disease will be known. When it is difficult to know about the disease, that time only all tests should be done. There is no use of doing the treatments later on. What was the cause of death? What is the need to find out the cause of death when the child is already dead? Like the child was sick for many years, one year or two years, so to know the reason, what disease is this. No one will do this (MITS) after the death, no one will get their child's sampling done. According to me, not a single one out of 100 will do this. After the death, they will give or not sample or something like that. What God has done it is right, that it. I think there is no use on doing this.
9. **How the parents/ families are likely to view the minimal tissue sampling procedure as a method for knowing the cause of death?**
   (Probe: anticipated views, concerns and believes, religious aspects, rituals, comparison with autopsy)
   If we talk to someone in advance or discuss about it then anyway, they will feel bad, about the child like about the death of the child. They will say you are killing our child when he is alive, here mostly people are of different mentality. The tests that you are referring will be done only after the death, when you will get to know. You will have a worker here who will send you message then in five minutes you will come and explain them and ask if they want to do it or not. When you will come, then only the parents will give permission to get their child checked.
10. **How the parents/families of a child/new born who has died, should be approached by the hospital doctors/staffs for the minimal tissue sampling procedure?**
    (Probe: who, when and how and whom should approach, who can be key influencer)
11. **In your view, what factors are important for acceptance of the minimal tissue sampling by the parents and family members?**
    (Probe: desire to know the cause, trust, confidence on the health care provider, who can be key influencer)
    <whom should we approach>According to me, nobody will give the permission. Whatever questions you have asked, no one will discuss about it.
12. **In your view, what would be the most suitable method(s) of educating the community about the minimal tissue sampling?**
    (Probe: medium, methods, place and target audience)
    <method of communication>
    People will say you are asking so many questions, what will they get from this, check-up will be done, our child has gone and you will do these things on our child. There is not much time required, after the death the body is kept in the house for 10-15 minutes. After that people say let's go, this happens brother. <who takes the decision about rituals after the death> The family, the parents. What the mother does, she does not know where the body has to be taken. There will be father, brother, or uncles, who do all the rituals or else there will be some neighbour who gives advice. Mother only cries all the time and nothing else. Rest you take opinion from all sides, take

someone along with you. Wherever there are two or four ladies sitting together take their opinion, whether they will agree or not.

<what will be the suitable method to educate community about MITS>

The way I am talking you will have to do it the same way, going door to door, or else we have to do in a way that you sit in one place, then ten people will come and ask what is happening. Then you can sit together and talk about it that we will do these things in it. Then you don't have to go door to door. If people are sitting outside, 50 people would naturally come to ask what is happening, Mr. Pradhan (head of the area), they will ask you what is it. They will ask you to identify and prepare 10-20 main places, then from one another everything will be known as I am there, I will tell four more people, like that.

<method of communication>

will understand verbally.

(Probe: acceptance by community and families, religious aspects, burial/cremation related)

What will be the problem when the parents' have given consent? Suppose people from this street have agreed and they understand the thing, then they will come and stand on their own that yes, the matter is ok and will help you in this.

13. **In your view, how the community leaders/influencers like you can contribute/ participate in such activity?**

    (Probe: who or what is it, what did you do in this context)

    I am far away from this thing, otherwise I would have opened ten NGOs at my house today, nowadays people are taking more advantage. People have making their child, their wife work in almost ten places. In a single room, they tell one NGO to come, at 2 o'clock another NGO, 3 o'clock another one, like that they are using 8 such NGOs. It is not possible for me, what to say the Pradhan (head) himself has opened one. I do not even speak about it in Anganwadi, I am far away from this thing.

14. **Any other specific comments/ observations.**

    Here people come from all areas they don't get benefit from here. There are lots of NGOs here, who are looting the public. Not the public but the government from where money is coming. Right now, there is a NGO which has opened, there are 143 teachers, they do not know which charity is that, salary is not less than 15-20-40 thousand, how much money is going there. Visit the place one day, if you find any two children sitting there to read. They are teaching in three shifts. Three lakh rupees goes in a month and there are total 7 children. They could have spent three lakh rupees in the attention of another child or for any girl's marriage then there will be much benefit. There are so many people like this who are sitting. We can say that if you will do this then it should give them benefit. Benefit will be that the cause of the illness will be known for the other children, what is the reason of the occurrence of this disease.

In-depth Interview -Community Health Workers

S. No.	Participant	Occupation
1	Participant 1	ASHA Worker
2	Participant 2	Anganwadi supervisor
3	Participant 3	Anganwadi Worker
4	Participant 4	ASHA Worker

In-depth Interview -Community Health Workers-01

1. **Basic demography**

1.1. **Designation/ Role-** ASHA worker

2. **Care seeking practices**

2.1. **Where do people from your community/ locality usually go or whom do you consult for healthcare** (all types of patients/illnesses)?

People get such check-ups done in private only and if there is something more important then they go to S Hospital but for little things there is a M clinic and our dispensary is PP.

2.2. **Which all health facilities are usually accessed? Which ones are mostly accessed?**

<Private> Many go to private. Now, if anyone does not want to go to the moholla clinic because of long queue, so they go to private. There is no hospital here.

<Nearest health facility> PP Dispensary (nearest health facility), it remains open only till 2 o'clock, and then it closes.

2.3. Which health providers are usually accessed? Which ones are mostly trusted?

People rarely go to anyone with a degree.

<who are they> There are many such people in our area, who go for small check-ups. In emergency situation the doctor asks to get admitted when there is serious condition. There was a case of pregnant women who was serious and then they have taken her to S hospital and got her admitted since day before yesterday.

3. **Where do people from your community/ locality usually go or whom do you consult during illness of children and new-born?**

3.1. Which ones are usually accessed for non-critical illnesses and why?

In that case, they go for taking some nearby local medicines, depends how far they want to go. They don't go to the dispensary; they show here only nearby. There are not many doctors here, everyone is practicing here and they are basically compounders who with experience do the job of a doctor.

3.2. Which ones are usually accessed for critical or severe illnesses and why?

Mostly people take the small children and go to MN or S hospital from here.

4. **Where do people from your community/ locality usually go or whom do you consult during pregnancy and delivery?**

4.1. Which ones are usually accessed for routine pregnancy check-ups?

At PP Dispensary

<test done>There we get the HIV test done in the blood from outside as no test is done there yet. When they know that she is 2-3 months pregnant then the test is done there at the same time. By the way, 4 visits have been said. There should be 4 visits for the pregnant women by the way we are called every month for check-up. Like for blood test, there is BP, weight, how much is haemoglobin, everything. Everything is treated for free. Yes, it is government clinic it is of Delhi government's. At the time of delivery, we call the ambulance by calling at 102 numbers who wishes to go, they go to S Hospital or go to MN hospital.

4.2. Which ones are usually accessed for delivery or pregnancy with complications?

Everyone does like this, there are one or two people who do not go and get vaccinated. It is their wish we have said many times, now we are tired. They don't come out and go for it. There is a new born baby in our area, near R's home. The lady never gets her children vaccinated, she has 4 children, never gets vaccinated. People from Delhi government are tired of saying that, our Madam ji is also tired of saying. The lady's delivery has been done on the 25th but then also she is not going for vaccination. She delivered at home but she is not doing anything for it. When the case is critical then people go to S Hospital.

Illnesses and causes of death

5. **In your view, what are the common illnesses and causes of child death in your locality?**

Here malnutrition is common here children do not get food in a proper way. The children are fed other milk mothers do not feed their breast milk. Weakness is common there can be any disease, nothing much is heard about it. Cold, cough and fever mainly occur here. Jaundice does not happen right now. Yes, children suffer from pneumonia, cold can cause pneumonia. Her mother works too much at home, does touch water or do not maintain cleanliness and works a lot at

home. Even now after she recently delivered her child, she started working at home, we see that and naturally the children will become tensed. When she works, our body gets numbed as yesterday only she delivered and she started working at home, also touches water. The mother-in-law is not there with her or does all the work alone. All because of that, the child suffers more illness this is the reason why food and drink are not done properly. This is the reason for infection, does not keep cleanliness, yes there is lack of cleanliness.

<how can disease be prevented> For that, we will go and explain, it is not that we do not explain. We explain them to keep cleanliness, to keep the baby clean, give shower to the baby in the sun, to put Dettol after washing the clothes and dry it under the sun. These things we can say. To prevent diseases, cleanliness should be done around the house the room where the child is kept should be cleaned. These things only we can say.

<any death due to illness> One child has just died 4-5 months back, they said that my child has remained asleep. The child was of 3-4 months, nothing has happened to the child just remained asleep. It was cold the child might have caught cold. Something must have happened; she is not revealing.

6. *We are trying to identify the causes of death in children, so that something can be done to prevent and reduce the number of deaths in children.*

   In your view, would this information be valuable?

   (If valuable/ useful, why? If not, why not?)

   We have asked, we tried to find out, but she did not say anything, then we informed our madam. No reason was found, she did not take her child to hospital.

Knowing cause death and implications of procedures

In some cases the children die before even the exact cause of illness and death is identified/ investigated. In such cases, doctors suggest autopsy/ post-mortem to identify the cause(s) of death.

7. **How autopsy/post-mortem is viewed by you and community members for knowing the cause of death?**

   (Probe: explore the views, concerns and believes in the community regarding autopsy)

   Yes, due to whatever disease or due to any other reason, everything will be known. Why did the child die, what has happened or something is happening, everything will be known. You will have to take the child to the hospital, and then only you know what is happening. You have not taken the child and at night the child has died, and in the morning started crying how the child has died.

   <believe about post-mortem> should be done as you know how the child died, should be done. There will be benefit, the reason will be known. Child die, we can reduce it, that the reason of death is this so we can look for the measures to prevent it or can also tell that keep the child in proper way. Or can tell the why child die, or can tell about the disease they are suffering from, these things can be told. We people will know the cause of death through post-mortem so that it can be prevented.

   <community believe> mostly it is known (about PM), maybe it is known or may not be, I never asked and talked about it. Yes, many people do not want to do, what will be the plight of a man after his death.

8. *In some instances, autopsy/ post-mortem is not possible due to various reasons. To find out the exact cause of death and underlying disease, some tissue biopsy (collection of very small sample using needles) and fluids (blood, urine, etc.) from the body of the child after they die, as done for investigation. But these samples are to be collected as soon as possible after death.*

   How do you view minimal tissue sampling procedure as a method for knowing the cause of death?

   (Probe: explore the views, concerns and believes, religious aspects, comparison with autopsy)

   This one is better; they disfigure the body in post-mortem.

   <concerns and beliefs> This will be done as we will explain them that they do not make an incision like post-mortem. Only this much if they get to know why death has happened, and there will be satisfaction that there will be no disfigurement.

   <religious aspect> No, it is not like that, in Muslims they disagree with post mortem but not so in Hindu.

9. **How the parents/ families are likely to view the minimal tissue sampling procedure as a method for knowing the cause of death?**
(Probe: anticipated views, concerns and believes, religious aspects, rituals, comparison with autopsy)
10. **How the parents/families of a child/new-born who has died, should be approached by the hospital doctors/staffs for the minimal tissue sampling procedure?**
(Probe: who, when and how and whom should approach, who can be key influencer)
To convince the parents (for MITS), we will have to talk to the elders at home. Then make the child's mother understand and then will explain to the rest of the members. First make the in-laws understand it because we first take permissions from the elders to go ahead. <in case of hospital death of child, who should be approached> The main person from the child's family, father, father will be explained, as he will only agree and not anyone else. <when should approach> When his (father) mind is calm. If they will perform tests, they will explain about it only after death but also can be explained even before death. Before death, how can we ask them, how can we say like this, means directly it can be said that yes you know that the child is going to die, he is not going to survive, or the child has some disease, we can take opinion from someone and talk, yes, that would be right.
11. **In your view, what factors are important for acceptance of the minimal tissue sampling by the parents and family members?**
(Probe: desire to know the cause, trust, confidence on the health care provider, who can be key influencer)
At that time, the parents will not be benefitted from it, but if they are informed about the reason of the child death then they can share the information with others. When they will find out the reason then they can tell others about its benefits, society will benefit. We will get to know what is the reason, why the child is dead, then we can make them aware to be careful with the child who may have such problems.
12. **In your view, what would be the most suitable method(s) of educating the community about the minimal tissue sampling?**
(Probe: medium, methods, place and target audience)
<mode of communication>
By verbal communication or by proper planning, it will be explained by the poster or picture. It will be easy, it will be better than speaking. They will see through the posters what size incision will be made, then that will be better.
<Medium> first you will have to promote it through TV, promote through meeting in the community then only it will be possible.
13. **In your view, what are the challenges for conducting the minimal tissue sampling at the hospitals?**
(Probe: acceptance by community and families, religious aspects, burial/cremation related)
It would be troublesome to make them understand, the parents have to understand right. Challenge is if they do not understand.
14. **In your view, how the community leaders/influencers like you can contribute/ participate in such activity?**
(Probe: who or what is it, what did you do in this context)
We can only explain this, we can work with you. We can roam in the field, motivate them in advance, and can make them understand that if there is some problem in their locality, then we can tell them to get this done. Everyone will have to work together.
<You can help us> yes, tell me.
15. **Any other specific comments/ observations.**
There is no health facility for the children here. After our efforts also, people do not come out from the colony and that women do not come out of the house for vaccination. We have told them a thousand times to get the vaccination but they don't understand. The child gets fever because of the vaccine, and so we say that two days will be troublesome. But for this reason, they do not go for the child's second vaccination they say my child is not well I will not go for vaccination.

By the way, everyone listens there is one or two who do not listen. One lady has only one baby girl but not a single vaccine has been administered to her daughter. They take help of the midwife for delivery at home. In one family they had faced trouble after being delivered at home. She is admitted to S hospital from 2-3 days, blood transfusion is going on. They repeatedly said home delivery will be done, they don’t listen to us.

In-depth Interview -Community Health Workers-02

1. **Basic demography**

1.1. **Designation/ Role-** Anganwadi supervisor

2. **Care seeking practices**

2.1. Where do people from your community/ locality usually go or whom do you consult for healthcare (all types of patients/illnesses)?

Dispensary in MN or MM hospital and then after that S hospital.

2.2. Which all health facilities are usually accessed? Which ones are mostly accessed?

Rest of the people go to small doctors in the private, who have opened up their shops, most of them go for fever, cough and cold, but many go to the dispensary also.

2.3. Which health providers are usually accessed? Which ones are mostly trusted?

What they think is correct accordingly they go there.

3. **Where do people from your community/ locality usually go or whom do you consult during illness of children and new-born?**

3.1. Which ones are usually accessed for non-critical illnesses and why?

First, they go to the dispensary here and if they say not here and they send them to MN Hospital.

3.2. Which ones are usually accessed for critical or severe illnesses and why?

If the condition is worse than we will refer only to MN Hospital.

4. **Where do people from your community/ locality usually go or whom do you consult during pregnancy and delivery?**

4.1. Which ones are usually accessed for routine pregnancy check-ups?

First a card will be made from their dispensary, only then they will be taken by the MN Hospital Hospital. By the way in MN Hospital they have everything, if they find anything severe, then they send to S Hospital.

4.2. Which ones are usually accessed for delivery or pregnancy with complications?

If the problem is severe then they (MN Hospital) will send to S Hospital only, they will refer from there only. But everything is done in MN Hospital, but firstly, name should be written in dispensary. Then the ID that you will receive will be seen further in MN Hospital.

Illnesses and causes of death

5. **In your view, what are the common illnesses and causes of child death in your locality?**

No, it is not like this, to the maximum they get cough, cold, but diarrhea is not much but when the child's teeth start to coming in or sometimes according to the weather, they get fever. Like the child who came in our Anganwari, he often gets fever, means he has come from last 10-15 days. Even we got him vaccinated for 3-4 times. Whenever we take him, they say Aunty he is having fever, then vaccination is due. One family came from village, I told them first cure the child's fever, take him to MN Hospital, when the fever is gone then only he will be vaccinated and get it from there only.

We are trying to identify the causes of death in children, so that something can be done to prevent and reduce the number of deaths in children.

In your view, would this information be valuable?

(If valuable/ useful, why? If not, why not?)

It should be that why are the children getting the issues, how these diseases are forming. In my area there are two deaths, one child had a hole in the heart as the child was a girl and the other was among the twins. Due to weakness their heart beats were short, one is now alive and other has died. By the way, never it happened that due to lack of blood or from any reason, the child had died. Child dies due to absence of something in his own body. Mortality rate is due to lack of blood or due to improper investigation or sometimes when child is delivered at home. It is important to know the reason, we need to give the reason of death, about the place of death of the child.

Knowing cause death and implications of procedures

In some cases the children die before even the exact cause of illness and death is identified/ investigated. In such cases, doctors suggest autopsy/ post-mortem to identify the cause(s) of death.

6. **How autopsy/post-mortem is viewed by you and community members for knowing the cause of death?**
   (Probe: explore the views, concerns and believes in the community regarding autopsy)
   That thing (Post mortem) should not be done for the little children doctors give them those slips where it is written. See, usually post mortem is mostly done when they think that there is a doubt. By when own mistake the child has died then mostly in that situation it happens. But no one will agree for post mortem for his child. If the doctor tells them that we have to do it, then parents are helpless in front of them.
   <Why parents agree for post mortem> everyone thinks that why should they do this for their child, does not want disfigurement for a small child.
   <Religious aspect> no religious objections are there.
7. *In some instances, autopsy/ post-mortem is not possible due to various reasons. To find out the exact cause of death and underlying disease, some tissue biopsy (collection of very small sample using needles) and fluids (blood, urine, etc.) from the body of the child after they die, as done for investigation. But these samples are to be collected as soon as possible after death.*
   How do you view minimal tissue sampling procedure as a method for knowing the cause of death?
   (Probe: explore the views, concerns and believes, religious aspects, comparison with autopsy)
   After the death right, otherwise they will think that when the child is dead, when the child is no more, then why to do the tests, parents will think like this. If we make them understand that for future this is beneficial for the children that these things do not happen in your other children, so we should do these tests beforehand. We will take extra care of the children and that this thing should not happen in the next pregnancy. But when you will make them understand about this then they might be ready.
8. **How the parents/ families are likely to view the minimal tissue sampling procedure as a method for knowing the cause of death?**
   (Probe: anticipated views, concerns and believes, religious aspects, rituals, comparison with autopsy)
   I told you that you have to tell them that for your child, for future it is beneficial and then they might get it done, otherwise they will not.
   <Religious aspect> There is no religious objection in this.
9. **How the parents/families of a child/new-born who has died, should be approached by the hospital doctors/staffs for the minimal tissue sampling procedure?**
   (Probe: who, when and how and whom should approach, who can be key influencer)
   Only doctors can explain about it. If the doctor says then they will still agree, if anyone from you tells then they will not agree.
   <Whom should be approached> Parents are not in that position to listen, either we can tell the head or anyone who is present from the family at that time, suppose head of the family is not there so we can speak to any of the elders of the family. When you feel, then only you can say because we think our patient is fine.
10. **In your view, what factors are important for acceptance of the minimal tissue sampling by the parents and family members?**
    (Probe: desire to know the cause, trust, confidence on the health care provider, who can be key influencer)
    Suppose, if the child has any problem, then only they will do but otherwise if the child is normal, then no one will do it. For the next pregnancy, they can get it done as the first child had issues and so the next one should not suffer from the same thing.
11. **In your view, what would be the most suitable method(s) of educating the community about the minimal tissue sampling?**
    (Probe: medium, methods, place and target audience)
    <medium/methods>
    Firstly by showing them video and also by showing in the TV etc. They should also know about it, because if we say it directly then they will only fight with us. If we say direct then they will fight with us and would say how you are talking.

<Target audience> by gathering women together or by showing a poster Posters should be put on like the MR's posters are being distributed, cards are being distributed and rallies are being carried out. They are showing that get this injection, and it is also coming on the TV as well, so they all know that we have to get this injection. So, like this they should know about this technique first, but it is a later process, when death has occurred then what the benefit of it is. But if we tell the benefits, its advantages before anything happens then they will still understand.

12. **In your view, what are the challenges for conducting the minimal tissue sampling at the hospitals?**
    (Probe: acceptance by community and families, religious aspects, burial/cremation related)
    Challenges will be there, because it is very difficult to explain a mother about child's death. If before-hand someone can explain that brother, this will be beneficial for your child, then also they will still understand the matter soon but this is after the death so it will be very difficult.
13. **In your view, how the community leaders/influencers like you can contribute/ participate in such activity?**
    (Probe: who or what is it, what did you do in this context)
    Help, one is your doctor, and next we are here Asha worker are there. MLA will not be able to do that much anyway.
14. **Any other specific comments/ observations.**

In-depth Interview -Community Health Workers-03

1. **Basic demography**
   Designation/ Role - Anganwadi Worker
2. **Care seeking practices**
   - **2.1. Where do people from your community/ locality usually go or whom do you consult for healthcare (all types of patients/illnesses)?**
     Like there are ladies, they ask us too. Why, they come to us. Pregnant women come to us, and children from 0–6 years also come. So they ask us whether to have to go for operation or have a baby in the womb or what has happened. Just like yesterday one lady was saying that I had a 3 months injectections but it was not even three months, she was saying that I have become pregnant again. I said that it would have been a long time, so you would have asked us or any Asha worker here. And there is a big dispensary here. (Where) There, near the roadside, near shiva temple. Like when we send then we send there only. We will also send them there first then we will send to MN.Yes, they go to S Hospital also from here.
   - **2.2. Which all health facilities are usually accessed? Which ones are mostly accessed?**
     People go to private. The villagers go to the private too, well those villegers are rich, they go to private only. Except for a few, they mostly go to private. Rest of them are tenants, these poor people pay their rent, and most of them go to the dispensary. And some have faith, people like us have faith in the government, so we have sent our daughter-in-law, wife in S Hospital only. We showed in the Holy Family hospital, and then don't know we did not like it to go there. Every month she went two to four times for ultrasound so later it was done in Safdarjung. Although the problem would have been more due to the crowd there.
   - **2.3. Which health providers are usually accessed? Which ones are mostly trusted?**
     Yes, there are private doctors but there is no good doctor here nowadays. Now they go to the dispensary, now they go to private only. When nothing is found, they will go there because the everyday the dispensary closes at 2:30 p.m and after 12 o'clock they do not give the prescription. Yes, there are 3-4 doctors here, minimum there are 4-5 doctors (Qualified Doctor). Everone writes that. Now there is SS and a doctor here named A. Previously there was a Doctor V who was very good. Whoever goes to him once, he would be fine.
3. **Where do people from your community/ locality usually go or whom do you consult during illness of children and newborn?**
   - **3.1. Which ones are usually accessed for non-critical illnesses and why?**
     In that case, as it happens, someone goes into private someone goes to MN hospital, if there is such emergency.
   - **3.2. Which ones are usually accessed for critical or severe illnesses and why?**
     They go to MN hospital only.
4. **Where do people from your community/ locality usually go or whom do you consult during pregnancy and delivery?**
   - **4.1. Which ones are usually accessed for routine pregnancy check-ups?**
     They go directly and also come to us. Some to go private or some go to dispensary. When they do not get relief anywhere then in that case, they also go to MN hospital too.
   - **4.2. Which ones are usually accessed for delivery or pregnancy with complications?**
     They go to dispensary itself, dispensary people if find any problem, then they send it there in MM Hospital. Those who go to private, they are refered to go to Parvatte. They even ask us, they also they go there only.

Illnesses and causes of death

5. **In your view, what are the common illnesses and causes of child death in your locality?**
   See, most of the rooms are so small, that there is neither wind nor sunlight. So if they will be inside always, they will be sick anyway. Like they catch cold or many times they face breathing problem. Because they roam sometimes here sometimes there. They say Madam my child had become ill. I said, put the child under the sun daily, take the child somewhere take him to the park. There are small rooms, in every room there is one tenant and they do not even come out of the room. We have big house, but inside the streets there are so many rooms.

<Cause> Death is decreasing nowadays, but also it is happening sometimes here although it is less. Death decreases, suppose when a child is premature, causes of disease are less known, if the child is unwell, we see child is born weak or premature.

6. **We are trying to identify the causes of death in children, so that something can be done to prevent and reduce the number of deaths in children. In your view, would this information be valuable? (If valuable/ useful, why? If not, why not?)**

Yes, but not much, there are not too many deaths nowadays, yes it would have been in the slum but it is less here. Deaths do happen, not like there are no deaths, one or few deaths might happen. Few reasons are that the child was born premature.

<Valueable, useful>. The child becomes weak, as women are not able to take care of her. Many times, their husbands do not earn that much so they do not get that much to eat and drink. And some have two to three children and it becomes difficult to handle them in a single room. So, they get sick.

<Valueable, why> No, we can only say and it also has a little effect. Now it is not that we keep on saying that take her outside or feed the Anganwadi food, the child is now six months. We get 'panjeeri' (mixture of wheat flour, sugar and dry fruits) in the anganwari, so feed them this only. Many times, we sit and feed them there only, and the children become healthy right away. Like some children are weak and the mother will say that the child eats nothing. So, I said leave the child here in Anganwari centre, you sit for sometime and give us time too. And feed him whatever is coming here. So, they don't feed them we sit and directly feed them. Then after two to four months, they really become healthy.

Knowing cause death and implications of procedures

In some cases the children die before even the exact cause of illness and death is identified/ investigated. In such cases, doctors suggest autopsy/ post-mortem to identify the cause(s) of death.

7. **How autopsy/post-mortem is viewed by you and community members for knowing the cause of death? (Probe: explore the views, concerns and believes in the community regarding autopsy**

They won't agree for this thing. They will say that my child does not have anything. When we say, they say that the child plays well. Nobody will do such things here. Suppose it happens (death) at house but when taken to the hospital, they will do it. It doesnot stop then. But if we ask to get it done at home, that the child is dead and we are coming, let us see what has happened to the child, so if we say like this no one will agree for this. We have such talks here that if suppose the dead body is delivered late in the evening then people say they definitely have disfigured the body. While they don't take out anything. People think for post mortem but same time they also think why we should do this when the person is no more. Now our child is no more, why should we go for post mortem. Even older people die, no one takes them for this thing. Even when there is any young person who passed away and by chance people have taken the body to the hospital and say that please see the person is dead, then by evening people think that why we had taken him there, the whole day has passed, and they must have disfigured the body.

In some instances, autopsy/ post-mortem is not possible due to various reasons. To find out the exact cause of death and underlying disease, some tissue biopsy (collection of very small sample using needles) and fluids (blood, urine, etc.) from the body of the child after they die, as done for investigation. But these samples are to be collected as soon as possible after death.

8. **How do you view minimal tissue sampling procedure as a method for knowing the cause of death? (Probe: explore the views, concerns and believes, religious aspects, comparison with autopsy)**

Now this has never happened, but according to me people should accept this much. Yes, because whatever we say to them and in any way, people accept it. Nothing like that. Like for polio, there are many Muslims they do not permit us to give polio drops as they think they won't have children in future. Even if they have their own perceptions they agree and listen to us. People listen to us even if there is an emergency. We say that nothing will happen, we are here only near you people, call whenever you think it is required, we will even stay at your home. Whenever you will say we will come. However, some fall ill due to other reasons. Such situation also arises. There was a child, very healthy but had hole in his heart, and we gave polio to the child on the

first day. We gave polio drops to many, not only to that child. Now there were not many cases like this, and we also had to listen to them. They said these people gave polio drops to the baby, these people gave polio drops and so the baby died. The baby died that evening but there was a reason, the baby had hole in the heart, although they too did not know about it, otherwise they would have created more problem. But they imposed their excuse on us. This is what happened in front of me. I said that this is what happened by me in 25 years, right. While I have also told that sometimes polio drops which are with us, sometimes the lid becomes loose when they are opened. Even if it comes out more, then also there is no harm. I said, I have given it to all the children it is not that we have given it to your child only. But after that they did not argue much because they knew the child was ill, but otherwise the child was completely healthy, and used to come to Anganwadi everyday.

How the parents/ families are likely to view the minimal tissue sampling procedure as a method for knowing the cause of death? (Probe: anticipated views, concerns and believes, religious aspects, rituals, comparison with autopsy)

According to me this can happen, by the way, people can believe this much. About disfigurement, when our child is already gone, then why we have to go for disfigurement, everyone thinks like that. Yes, for injections they think they are doing some tests. Nowadays there is awareness among people. Even people who came from outside, have awareness after living here from long.

9. **How the parents/families of a child/newborn that has died, should be approached by the hospital doctors/staffs for the minimal tissue sampling procedure? (Probe: who, when and how and whom should approach, who can be key influencer)**

   If the child dies in the house, then the doctor will not know it. We too, suppose the death happened in the evening and the next day early morning, the rituals are done, then we will also know about the death on next day only. If they go to the doctor, in the hospital, the child will be there, so the doctor will know about everything. Now we cannot say beforehand that your child will have this problem, and so you can do this. These words cannot be spoken to anyone. If someone's child is ill, these words can be said to them only. But even if the child is severly sick, we cannot say this that if something happens then please inform us. Yes, at least we can say that if something happens, please let us know immediately. But even if we say, people will not agree. See, now you can say only this that whose child is very ill, like someone has cancer, or someone has jaundice, and doctor has given up all hopes, to them we can say that if something unfortunate happens then also please inform us. We need to see why this has happened. It is also the matter of area, now there are different ways to talk to the slum people. They will take our food so comfortably. Here, means, some people have full knowledge here, if not that much then they know something on it. In the hospital, who so ever doctor tells about it, ladies will not understand the matter, will not understand it at all, but gents will tell you that the elders should be asked about it.

10. **In your view, what factors are important for acceptance of the minimal tissue sampling by the parents and family members? (Probe: desire to know the cause, trust, confidence on the health care provider, who can be key influencer)**

    No, it can only happen that they also find out why it happened with their child only. Because when something happens, it seems that why did it happen with us only. So, for this thing, people might say yes. Everybody says everything to the doctor. Whatever doctor does in the hospital, no one will say anything.

11. **In your view, what would be the most suitable method(s) of educating the community about the minimal tissue sampling? (Probe: medium, methods, place and target audience)**

    Mahila Mandal (Women committee) or grouping of women (2 Anganwadi talking among them). Like it used to show in T.V. that keeping the navel part is okay. That thing we or anyone else were not able to accept that it can be kept or stored. But now that such big things are happening through T.V, that people, like today’s children, our daughter in laws, they also think that yes, we can keep that thing, which will be useful in future. So in this way, through the video it can be done. And the same thing can be conveyed to them in twisted way or other way, to make them understand. By forming the women committee, like ask two women to sit and request them not to say that such thing will happen but if such situation comes, then we should know it from inside. But we cannot talk about the death of her child in front of a woman, it can’t be said. For once it is

possible to talk to the gents but not with the woman at all. <Target audience> for the child, the parents would be right. The mother will be right, the father will not be available in the house because the father goes to earn, it can be done only on holiday on Sunday, but the father also does not understand so much. The father does not. Mother understands more about children, father also loves and care for them and fulfills their needs.

In your view, what are the challenges for conducting the minimal tissue sampling at the hospitals? (Probe: acceptance by community and families, religious aspects, burial/cremation related)

Challenges, with which ever we will share this they will have problem only. He will start abusing you as soon as we arrive. First, we will tell about the benefits, that this will be helpful. They will spend a good life and suppose he goes and tell his sons to do it, but they will not agree for this. They will stop talking. Yes, if before going to them if you people come to us and tell about the benefit, like we have come for your child and we will take care of everything, whatever you need, we will fulfill it. Like in treatment, we will give you to eat and even after this if they have any disease, if something happens then we can do this test. It can be said in this way but not in any other way.

12. **In your view, how the community leaders/influencers like you can contribute/ participate in such activity? (Probe: who or what is it, what did you do in this context)**

    Listens to Asha workers too MLA and all, they can not do so much or go to them. Most of them listen to Asha worker and Anganwadi. There are doctors in dispensaries, if you can go till there. If this is explained by the doctor, it is more understandable to the people.

13. **Any other specific comments/ observations.**

    Yes, it is just that if you have to do this then you will also have to come a couple of times you will also have to see how their children stay. Some children become weak for some time, and it looks like what has happened to them. Like when they have growing teeth, then for two to four days they suffer from loose motion. They will be weak; we cannot say that our child is suffering from this.

In-depth Interview -Community Health Worker- 04

1. **Basic demography**

1.1. **Designation/ Role-** ASHA worker

2. **Care seeking practices**

   2.1. **Where do people from your community/ locality usually go or whom do you consult for healthcare** (all types of patients/illnesses)?
   People go usually to private for immediate care. If anything serious occurs, they go to S hospital. But for small things, they go to Mohalla clinic and nearby dispensary.

   2.2. **Which all health facilities are usually accessed? Which ones are mostly accessed?**
   Many usually go to private. Anyone, who does not want to go to the moholla clinic due to long queue and time constraint, they go to private. The dispensary (nearest health facility) remains open only till 2 o'clock, so people many times prefer to go to private.

   2.3. **Which health providers are usually accessed? Which ones are mostly trusted?**
   People rarely go to any formal doctor. There are many such people (informal practitioners) in our area, who go for small check-ups. In emergency situation the doctor asks to get admitted when there is serious condition. There was a case of pregnant women who was serious and then they have taken her to S hospital and got her admitted since day before yesterday.

3. **Where do people from your community/ locality usually go or whom do you consult during illness of children and new-born?**

   3.1. **Which ones are usually accessed for non-critical illnesses and why?**
   They don't go to the dispensary; they show here only nearby. There are not many doctors here, everyone is practicing here and they are basically compounders who with experience do the job of a doctor.

   3.2. **Which ones are usually accessed for critical or severe illnesses and why?**
   Mostly people take the small children and go to MN or S hospital from here.

4. **Where do people from your community/ locality usually go or whom do you consult during pregnancy and delivery?**

   4.1. **Which ones are usually accessed for routine pregnancy check-ups?**
   At Dispensary Center. There we get the blood tests done. When they know that she is 2-3 months pregnant then the test is done there at the same time. By the way, 4 visits have been said. There should be 4 visits for the pregnant women by the way we are called every month for check-up. Like for blood test, there is BP, weight, how much is haemoglobin, everything. Everything is treated for free. Yes, it is government clinic it is of Delhi government's. At the time of delivery, we call the ambulance by calling at 102 numbers who wishes to go, they go to S Hospital or go to MN hospital.

   4.2. **Which ones are usually accessed for delivery or pregnancy with complications?**
   They go to dispensary itself, dispensary people if find any problem, then they send it there in Madan Mohan.

Illnesses and causes of death

5. **In your view, what are the common illnesses and causes of child death in your locality?**
   Malnutrition is common in children here, they do not get food in a proper way. The children are fed other milk, mothers do not feed their breast milk. Cold, cough and fever mainly occur here. Yes, children suffer from pneumonia, cold can cause pneumonia. This is the reason for infection, does not keep cleanliness, yes there is lack of cleanliness.
   We explain them to keep cleanliness, to keep the baby clean, give shower to the baby in the sun, to put Dettol after washing the clothes and dry it under the sun. These things we can say. To prevent diseases, cleanliness should be done around the house the room where the child is kept should be cleaned. These things only we can say.
   <any death due to illness> One child has just died 4-5 months back, they said that my child has remained asleep. The child was of 3-4 months, nothing has happened to the child just remained asleep. It was cold the child might have caught cold. Something must have happened; she is not revealing.

6. **We are trying to identify the causes of death in children, so that something can be done to prevent and reduce the number of deaths in children.**
   Few reasons like pneumonia, malnutrition and if child was born premature.

In your view, would this information be valuable?
(If valuable/ useful, why? If not, why not?)
We have asked, we tried to find out, but she did not say anything, then we informed our madam. No reason was found, she did not take her child to hospital.

Knowing cause death and implications of procedures

In some cases the children die before even the exact cause of illness and death is identified/ investigated. In such cases, doctors suggest autopsy/ post-mortem to identify the cause(s) of death.

7. **How autopsy/post-mortem is viewed by you and community members for knowing the cause of death?**
(Probe: explore the views, concerns and believes in the community regarding autopsy)
Yes, due to whatever disease or due to any other reason, everything should be known. Why did the child die, what has happened or something is happening, everything will be known. You will have to take the child to the hospital, and then only you know what is happening. You have not taken the child and at night the child has died, and in the morning started crying how the child has died.
<believe about post-mortem> should be done as you know how the child died, should be done. There will be benefit, the reason will be known. Child die, we can reduce it, that the reason of death is this so we can look for the measures to prevent it or can also tell that keep the child in proper way. Or can tell the why child die, or can tell about the disease they are suffering from, these things can be told. We people will know the cause of death through post-mortem so that it can be prevented.
<community believe> mostly it is known (about PM), maybe it is known or may not be, I never asked and talked about it. Yes, many people do not want to do, what will be the plight of a man after his death.
In some instances, autopsy/ post-mortem is not possible due to various reasons. To find out the exact cause of death and underlying disease, some tissue biopsy (collection of very small sample using needles) and fluids (blood, urine, etc.) from the body of the child after they die, as done for investigation. But these samples are to be collected as soon as possible after death.

8. **How do you view minimal tissue sampling procedure as a method for knowing the cause of death?**
(Probe: explore the views, concerns and believes, religious aspects, comparison with autopsy)
This one is better; they disfigure the body in post-mortem.
<concerns and beliefs> This will be done as we will explain them that they do not make an incision like post-mortem. Only this much if they get to know why death has happened, and there will be satisfaction that there will be no disfigurement.
<religious aspect> No, it is not like that, in Muslims they disagree with post mortem but not so in Hindu.

9. **How the parents/ families are likely to view the minimal tissue sampling procedure as a method for knowing the cause of death?**
(Probe: anticipated views, concerns and believes, religious aspects, rituals, comparison with autopsy)

10. **How the parents/families of a child/new-born who has died, should be approached by the hospital doctors/staffs for the minimal tissue sampling procedure?**
(Probe: who, when and how and whom should approach, who can be key influencer)
To convince the parents (for MITS), we will have to talk to the elders at home. Then make the child's mother understand and then will explain to the rest of the members. First make the in-laws understand it because we first take permissions from the elders to go ahead. <in case of hospital death of child, who should be approached> The main person from the child’s family, father, father will be explained, as he will only agree and not anyone else. <when should approach> When his (father) mind is calm. If they will perform tests, they will explain about it only after death but also can be explained even before death. Before death, how can we ask them, how can we say like this, means directly it can be said that yes you know that the child is going to die, he is not going to survive, or the child has some disease, we can take opinion from someone and talk, yes, that would be right.

11. **In your view, what factors are important for acceptance of the minimal tissue sampling by the parents and family members?**
(Probe: desire to know the cause, trust, confidence on the health care provider, who can be key influencer)
At that time, the parents will not be benefitted from it, but if they are informed about the reason of the child death then they can share the information with others. When they will find out the reason then they can tell others about its benefits, society will benefit. We will get to know what is the reason, why the child is dead, then we can make them aware to be careful with the child who may have such problems.
12. **In your view, what would be the most suitable method(s) of educating the community about the minimal tissue sampling?**
(Probe: medium, methods, place and target audience)
<mode of communication>
By verbal communication or by proper planning, it will be explained by the poster or picture. It will be easy, it will be better than speaking. They will see through the posters what size incision will be made, then that will be better.
<Medium> first you will have to promote it through TV, promote through meeting in the community then only it will be possible.
13. **In your view, what are the challenges for conducting the minimal tissue sampling at the hospitals?**
(Probe: acceptance by community and families, religious aspects, burial/cremation related)
It would be troublesome to make them understand, the parents have to understand right. Challenge is if they do not understand.
14. **In your view, how the community leaders/influencers like you can contribute/ participate in such activity?**
(Probe: who or what is it, what did you do in this context)
We can only explain this, we can work with you. We can roam in the field, motivate them in advance, and can make them understand that if there is some problem in their locality, then we can tell them to get this done. Everyone will have to work together.
<You can help us> yes, tell me.
15. **Any other specific comments/ observations.**
There is no health facility for the children here. After our efforts also, people do not come out from the colony and that women do not come out of the house for vaccination. We have told them a thousand times to get the vaccination but they don’t understand. The child gets fever because of the vaccine, and so we say that two days will be troublesome. But for this reason, they do not go for the child’s second vaccination they say my child is not well I will not go for vaccination.
By the way, everyone listens there is one or two who do not listen. One lady has only one baby girl but not a single vaccine has been administered to her daughter. They take help of the midwife for delivery at home. In one family they had faced trouble after being delivered at home. She is admitted to Safdarjung hospital from 2-3 days, blood transfusion is going on. They repeatedly said home delivery will be done, they don’t listen to us.

In-depth Interviews-Religious Leaders

S. No.	Participant	Religion
1	Participant 1	Hindu
2	Participant 2	Muslim
3	Participant 3	Christian
4	Participant 4	Sikh

In-depth Interviews-Religious Leader-01

1. Basic demography

1.1. Religion served/ followed – Hindu

1.2. Designation-Pandit

2. Please describe the rituals and practices followed around and after death (Please describe these day-wise and duration). (Probe: for adults, children, neonates aged < 1 month, still birth and abortions)

See, the biggest thing is that we are connected with religious things which say the book, in which Veda is astrology, just like you must have read generally, there is commerce, science, arts, and there are 4 subjects in our Sanskrit. Vedic astrology grammar fine. In which Ayurveda, also comes, Sastra also comes in all kinds of studies. This is the knowledge that happens, when we have got to see this, whether there are children or young people or old people or whatever, when they come under the circumstances, as if you are saying about death. So, if we come here to the people, then people come to the court of the mother or they visit the court somewhere. In that position, when it comes here, then the doctor says that the medicine we are doing will be prayed from above. In that position, people reach here, then we pray and chant in his name, perform haven, worship and the same person comes here and heals that here we have got work in medicine. There is no such power, and with full faith we say that there is someone who is admitted in the last position of medicine doctor in the ICU , not one but many people have come in front who are the last Stays in time and then comes to the end here According to the scriptures of the people, if a child is born about 10 years of age or dies, a child who is born is dead, then according to the custom of the village society, people suppress it in the soil. If 3 years or year old or congenital death occurs. If you have grown children for 3 years or a year, then the fire is given by Hindu custom. After 3 years, if you have children older than you or if you are born or have an abortion, then it is suppressed in a sweet way. If above a year or have grown above 3 years, he is given fire. And if older than 10 years, or old dead or young dead or premature death dies, or any kind of death, the position is 13th, the body is donated, and the shraddh is done. For peace we have a book of people Garur Purana, people of Garur Purana listen to it, many people listen to Gita and after giving mouth fire for 9 days, karma starts. Agni karma has been done, taking it on the banks of the Ganges or where the custom is, according to which we are ganged with people, we are from Bihar. 9 days are mass donations, pundits come and 9th day means 10 days which is called 9th hair, we have hair shaved here, hair stripes are all shaved. On the 11th day the body is donated, then on the 12th day the bodies are donated, the Brahmin feast, and offer salute to their East.

3. Please describe the differences in rituals and practices according to the place of death and natural versus premature death like a child and at the time of delivery/birth. (Probe: death at home versus death at hospital)

Pandit **-** no one is different, it is the same for everyone, for that also you will have to do so much for peace, yes (bury), see that there are many places in the water, but according to the custom of our people we are not buried in water , He is buried in the water who is a saint, meaning a saint who does not have children, fire can be given by anyone, brother can give, someone can give but he is buried Yes, in water, what is said, fill the sand in the pot and make it into the Ganges, but according to the rites of our people, the mouth fire is the biggest.

Yes, we do it from there to the hospital (bury) Yes it is like if we are in the hospital, then it is near from there, suppose that it is now Delhi, people coming from the village in Delhi live in Delhi, or Somewhere outside, if some kind of incident happens, from now on, it will be said that you have to take UP, take it to Bihar, or take it to Bengal somewhere, tough work is there. It is here that he did this here, whether going to the cremation site or going somewhere, he did the deeds, if he goes to the Ganges, but that person will try to do the deeds, we should go to his birth place and after getting the information here his house But that karma will start, it is not that if it happened in Delhi, then its effect is not in the house because there is a relation of blood, no matter what happens it may happen, but karma has to be done, and everyone participates in it.

4. Please tell us about the requirements for burial or post death rituals in the religion. (Probe: death certificate, timing, presence of relatives, cost of the procedure)

(Death certificate) If this happens, see we have not got such an opportunity to go to the crematorium in Delhi, then what is the system there, we cannot tell, here we ask for a certificate? Asks for Aadhaar? It is not like that in the village, there are those who go to the Ganges, those who are there mean those who are on the banks of the Ganges, they take their expenses, whatever it is, do not ask for any paper, if it happens here in Delhi, then there is an accident. Or if it is covered and faked then burn it anywhere in a hurry. If there is a certificate, then there is no entry in the crematorium without a certificate, then they are burnt.

(presence of relatives-woman) There is no meaning with the man and woman there, we do not go to the village of the people in the village, but in Bihar itself, in Darbhanga, it is fine, there people do not go to the Ganges. But it is buried, beside it means inside the village-society we are not allowed here but go there This is according to their own custom, and in Delhi, we see a lot of people coming in the car, yes women also come. We see that they have never gone inside

(cost) There is no limit to it. It is not right that people spend more according to their power, they spend more in pretense, what is the practice, what is the practice that if they die, then their What do we do for liberation So it has been said that do the work, donate something, and charity does not mean that you donate in lakhs, charity means that the person who is killed is our feeling that we donate to him daily. According to the peace of whoever is there, Pitra Paksha is going on right now, the month of Shraddha comes for 15 days in a year, though people do it on Amavas, why do they surrender now, why donate now It has been a year of death, it has been ten years, it has been five years, in spite of that, the Brahmins are feeding and donating, why is there somewhere that the ancestor is dead For their peace, this is the year in which a man takes care of the child, parents do that after my death, at least I will give water in my name. So whatever he is doing now, someone is making his money in the name of his parents, grandparents, in the name of him, then whoever is dead, whether it is a child or a child, is going to donate five pots. Doing or donating clothes or doing something in the name of it, he will get the fruit of it, now the cost of spending is now spent, who feeds the person who eats, We will believe that if you eat 10 rupees a day, then if you feed someone, then it costs 10 lakhs, it does not make any sense, you feed the person who you eat, whether fed 200 man, fed 500 man.

(Timing) This should happen within 12 hours, Sun rise and sun set, suppose if a person died in the sun at 8-9 in the night, then close the crematorium at 12 o'clock then morning Will only open, the problem of commuting, the problem of car and horse, assuming there is a town here, everything is arrangement, we are talking about everywhere, so what can happen in that position If the person who has died, will not have any news related to him or his boy is the priority of the child of the fire, if someone dies, it is the priority that his children will give fire, then it may be that he is out of the house. It will be news here that people may be late in reaching it; Sometimes people wait, sometimes it also happens to be out of place, and if it is in the army, it takes two days to come from there. So for those who go from Delhi, it is for them that lets brother is a boy or his boy will not come, in this position, the man keeps on ice and arranges and waits for one day that he will come, then try that if at night If done, then at 10 in the morning till 12 o'clock.

5. In your view, what happens to the spirit, when a child/newborn dies? What is usually done by the family and/or community to help this happen? (Probe: what happens, what things are done, what are not done, what happens if they are not done)

Pandit- Yes, we do humble prayers, not for those children who have been born, who have not come here, who have been below for a year, who believe that they have not come here, in 9 months or 10 months of some kind. They go before the issue, but people do it. The women of the house or the gents who do the work do not work for 13 days, but according to the rituals which are not done, cutting of nails, turning hair are all these. After 3 years, it is not a matter of deeds it is also for that, there are not 13 days. On the third day, whatever is in his name, Mahapatra Pandit, well there is no atmosphere of happiness, the elders die, and the old ones die, they do charity in a lot of ways, but if a child becomes like this then by calling, formality In charity, for his peace, by worshiping the pandit from the pandit.

Pandit- See premature death whether it is in child or youth in youth, this is called premature death. The soul that is the biggest thing is the emotion which is sitting inside, which has been happening since before such premature death has occurred, for this we should go to such a place of pilgrimage as we have gone to Bihar, here is Haridwar, If it is Kurukshetra, it happens that by going to this Dham, donating the body gives peace to his soul and attains salvation.
Yes, there is no such birth in our religion, but we have read that "Sanskrit slok" is considered to be a rite of pre-birth, education and wealth, we are the only ones who have to suffer in the present life. As soon as you will ask how in any way it is seen from you that there is a great person who has read and written a lot, and who is illiterate who is new to my hardworking, he is also increasing fourfold, day and night. It is a matter of thinking that works hard He does not get bread and vegetables in his stomach and if he goes ahead without much effort, then this is the deed of his previous birth. Regardless of the work you do in your previous life, whatever the person is in you, we get the same amount of hard work and remain happy with happiness, despite our hard work in front. The time that changes when the cow of the moment comes, when the karma of previous birth comes to an end and we who are doing good deeds right now, then whatever is in this position, then you get happiness. For the peace of the soul, the first thing that has been done is that its karmas are very small children, they were told earlier that we are cremated or else they are buried inside the soil, which is such, but karma is for them Quit deeds are also done, people do not turn hair, for the sake of the saint, and after doing what they do, and if they do not feel peace in the end, then they went to Haridwar and donated their bodies. They give place of worship there.

6. People are usually sad when child dies. How do people in the community show their sadness? (Probe: what is done by the parents, immediate family members, how does the community and relatives support in this)

Pandit - He is here to express his sorrow, no one can stop, he is not guilty in this when the incident happens or God is not guilty in this. Is that God killed someone killed Wrong insulin fell, wrong needle was injected, due to the negligence of the doctor, if he fell, he died because of it, he would have been given some excuse, due to which he is comforted by beating himself and other people.

7. People are usually sad when a pregnancy ends with birth of a dead child (still birth). How do people in the community show their sadness? (Probe: what is done by the parents, immediate family members, how does the community and relatives support in this)

Pandit- The biggest thing is sadness, it is sad that everyone appears, old people die in it, or sad people appear in it and no young children die, they are also in pride and they are equally proud. If you are in your own pride, then it happens that even if I see it, it is as new as sadness is as much in old age or in a child, death is death, whether it is in any condition or someone. (Community and relatives support in this) See this is the case. It can happen or happen on anyone, that is why the society is such that friends are there and friends are there to support them in sorrow, someone is a friend, is a friend, So when someone catches up with the help of each other, whether it is physical or financial, mental or mental, the people of the whole society are standing at the time like this, no matter what kind it is at that time, whatever the society is from everything Sport him, today he will do it, tomorrow the incident happens with him, those people will console him.

8. How do the parents and families cope after death or loss of pregnancy/death of the child at birth? (Probe: behavior, spiritual practices, family and relatives role)

Pandit- See death is true, everyone is aware that one who is born does not die but as far as consolation is concerned, it slowly wears out, eats new for two days, will eat new for three days and then sir They will bang their hands or do some kind of work, but gradually they are the same people of the village who are the people of the society, the women, the people are very much, the men come to console them and make them patient and then slowly forget them to talk.
(Spiritual practices) Yes, there is a reason for inclination in devotion, even mother ladies do fasting in such a way that they do this fast only for their children and in our Hindu, we are fasting for the son of the daughter. It is done specifically for the special child, for the long-term wish, and even then, if any kind of incident happens, then people of a birth say that it is karma.

(Family and relatives role) Cooperation gives people from all over the family; Even the grief of one parent is new; The grief of one parent is new. An entire family, society becomes sad, so one's home In the event that happens or after someone is good, there is a fight, each other's fight, fight and fight happens but after an incident happens, the fight ends, everyone is standing.

9. Is there any social stigma or taboo related to family or woman in relation to her child's death or stillbirth? (Probe: blame, spiritual factors, effect on family or children or subsequent pregnancy)

Pandit- See Dosha, there are many types of Gods and Goddesses in the countryside in the village society, either too many, for Dosha Ropar, one thing is to say, God does not have any blame, God is killed and God is God If you kill someone, then in this position, no one will accuse anyone.

(blame parents) Look, if there is negligence of mother and parents, if there is negligence then there is fault. There are many types of faults. One tells God at that time that it has not been done because of it, or whether any children are living in the delivery hour, then at that time women are often in contact with the doctor. Often women keep in touch with the doctor, whether it is village, rural or government, wherever. So if there is any kind at that time, the doctor would give him satisfaction beforehand. This was the problem this was the one, because of which it

(Spiritual factors) See the thinking that is in society, there is a lot of different kind of thinking. The greatest happiness is said to be physical pleasure. Pleasure of the body If it happens to someone, then it definitely comes to mind that somewhere a mistake has been made from us, which has resulted from us. Be it any kind of event or physical or mental. So little happens on the fact that somewhere we from us we have done wrong only when such a situation has happened to us.

(Effect on family or children or subsequent pregnancy) Every parent thinks, everyone thinks, after thinking that after being one, and then there are more vigilant people. Whether it is by about the doctor or for it people from God or going to the court and ask for blessings. Effectively is vigilant, one that has happened which should not happen again, so effectively is alert.

10. In your view, how important is to know the cause of death? (Probe: how this can help the parents/family and others, how is seen in the community)

Pandit- We has already told you, parents, what was there earlier, people were not comfortable in the village society. So first, there are those who are from such villages who used to come to the village, who are called nurses who say, who used to deliver delivery at home Today, the government has provided facilities for that too, they also give money, etc., so now whatever delivery is done from the village to here, we feel that most of it is being done through the hospital itself. So those who are going through the doctor and the doctor inside their satisfy that such a situation happened for this reason. Then there is nowhere to blame. By the way, many kinds of rumors should be doubted by someone, you can tell this later, but when most of the people are now in contact with the doctor.

(Benefit after knowing cause of death) If you get information then you will be cautious with it. If we get the information, we will keep it to ourselves, it keeps the facilities that it has. It is the mother-father the mother has the most loving son, whether the child is a son or a daughter. If this happens no mother will not want it to happen, for whatever reason. So that is why whether one visits the court of God or goes to the doctor. It remains the same to everyone.

See, it is our position in the position that those who are above in that position, God never takes the blame on himself, no one has ever listened to this day, God has killed, no one speaks, died due to treatment, doctor negligence, carriage The person climbed, fell, it was as if some excuse was needed. See, we, not the general man, think that no one can stop being there, second is that some men go astray in darkness.

11. What are the beliefs about post mortem (or autopsy) in your religion? (Probe: acceptance, religious and spiritual concerns)

Pandit - There is no such belief anywhere. But one has to say that when the event happens, some of our laws here are also very flexible, loose. A simple brother is giving so much news on TV, that if he is in an accidental, no lifter is found. Why should the police go to the police station, tell everyone your name, address, write mobile number, it bothers him. When you call again and again, you have to come. Etc. - Etc. So one sad incident with him sad event happens. After that it is yes postmortem accidental of the

child. Or someone commits a crime with someone, kills, then the post-mortem is done as per government rules, when there will be an FIR then a post-mortem will take place. But no one would like to do that. There will be an FIR then a postmortem. Nobody would like to do this. After the death, we do our post-mortem to the child, why do we know the condition of the child and how he killed, he remains in mourning that post-mortem does not have any meaning with the child. (Religious concerns for post-mortem) There is no such. Is not denied

(religious norms on post mortem) There is no such refusal but postmortem report belongs to someone who is having some kind of accidental or accident, if you are also involved in medical matters, neither administration, nor any arrangement here. , In this position, even the people of the house are upset, no one is untouched, and they are not even supported. Now, until someone goes to the hospital to call the police to say that it is nothing, if we believe that there is You would say that without the police we cannot do anything We ask that when someone goes to you, you will say that you have made an entry in the police station, you have told the police that the son will die; I cannot blame anyone but my next person is killed, the police will come. Will question a hundred, how did you die, where were you, how did you eat what happened in the night, don't you drink a hundred lofty, nobody can trap that the next person killed See my boy is well healed and slept in the night, then it has become untoward and after that you will go to you, you will bruise the needle, rip it, lick it in a lot, that people don't want to do, that's why they don't want to If a tragic incident happens to him, he feels in front of me that my boy is right, yes something happens, during treatment, he is satisfied that he was ill before or he had someone already, you will tell correct If this happens at that time, then the doctor goes to the doctor, then he says that it is finished, whether it tells the local doctor or MBBS, because he has told that it is finished, after that he is satisfied that now it is over . No, (willingness to know the cause) then after that you start in your own world in crying and beating.

12. After a post-mortem is done, are there any modifications in the rituals done in the funeral process? (Probe: body preparation, mode of burial/cremation, post burial/cremation practices, related cost implications)

There is no point in worshiping the Pandit-postmortem. Karma will be his, whether it is post-mortem or not. The person who performs karma will do it socially or sacra mentally. There is no point in postmortem. There is no such thing (change in rituals), everything is done (cleaning of the dead body) all will be in the same way, there is no one in it, but people do not go for this (for post mortem) because everyday police Will come to the door, the neighbors will say something upside down, then they will also want to take advantage of it, that's why people do not want to get crooked. We see whoever they are.

13. What are your views about collection of the tissue and body fluid samples for identifying the cause of death? (Probe: necessity, religious aspect, time needed, etc.)

Pandit- See until people are not aware, when the minds of the people will not be understood, no one will grow in it. So until today, when it came to talk of donating blood, it was said that no one did it or no one is doing it. Somewhere, someone will have to move forward. For any such test, after giving the needle to your child, after dying, do not tell him the post-mortem.

See when delivery happens when you say that the birth of a child in the womb ... is considered death, assuming that if someone dies in the womb, then most of it is in contact with the doctor. There is no death in the womb without a doctor here. When the patient does not live, that is why people reach the doctor even in the last position, even if they are not in treatment from the beginning, but they reach the last position. Doctors say that its children have deteriorated. So there should be a law that if there is a post-mortem in this position, then let the children be taken from here. As the accident-prone police station says, first do an FIR, there will be a post-mortem then you can take it. Only then it is possible. If a child dies in such a house, people will have to become very aware of it by doing needlework and checking here. Will happen

(agree for MITS) Yes, to know the reason, all needles are medicine needles, etc. if it is good.

14. How parents and family members can be approached for the tissue sampling? (Probe: who, when and how should approach and whom)

(when) Now look at the main point, when the delivery takes place, the list of everyone is there, now at that time you mean to go to that time that you tell someone that if a child incident happens to you, then you get the post-mortem done. Taking it, or getting it injected, second we think that he will keep everything in your mobile. If someone goes to tell you that if an incident happens to you, then you get it done, then it will make you sit first at that time. You will forget that these things have come for your interest. Nobody will tolerate this. That is why the collective will not make you aware, until people become aware neither people will not understand the matter.
(whom) bitter thing comes true. Truth is bitter. If the truth is known to his grandmother, parents or anyone, then it will not be good at that time. Will never accept you. Yes, by going to a group house, you say to a particular person, he will not tolerate it. If the collective disseminate this, what is happening, why it should be done for awareness, then people will understand. If you want someone to go and explain to someone, you will think that I have come here thinking that such an incident happened in his house. So you will not be able to tolerate so much, someone's mother and father are grandparents.
15. In your view, what factors are important for acceptance of the tissue sampling by the parents and family members? (Probe: desire to know the cause, trust, confidence on the health care provider)
See, the first thing is that in recent times there is a media. First of all, it should be widely publicized, make people aware, people should understand. Today, some disease comes, new people, we have come to Delhi, we are from Bihar. Those who come here never hear the name of the disease and every year new diseases keep coming. It is amended, people are aware of it, so they have already propagated that this swine flu is coming, so it is happening, it is happening. When people are aware, they do too much about it. For this, make people aware beforehand then people will move slowly.
16. In your view, is there any modifications in the rituals done in the funeral process needed with the minimal tissue sampling procedure? (Probe: body preparation, mode of burial/cremation, post burial/cremation practices, related cost implications)
Pandit **-** No customs. Nothing

In-depth Interviews-Religious Leader-02

1. **Basic demography**
    1.1. Religion served/ followed
    1.2. Designation –Imam

    Death and related practices
2. **Please describe the rituals and practices followed around and after death (Please describe these day-wise and duration). (Probe: for adults, children, neonates aged < 1 month, still birth and abortions)**

    If you will be said to keep the body in your house, then no one will be ready to keep it. After some time, smell will come from it. Every religion has its own ritual and practice of burying, cremating or burning the body. They do their work. It is said that person has connection with the soul and not with the body. When the body is separated from you or your soul, it automatically starts tearing from the eyes of the people. People start crying. There is feeling to sadness. This feeling comes from inside of the heart of people. After relatives come after the death of the person, the first thing they do is that they inquire whether the person who died had taken any money or loan from any other person. Have they borrowed money from someone? If it is written somewhere, it should be taken. Whatever is given by the goods, it should be given. It should be paid, it is tried. Then it is seen who is among apostates, whether it is a boy or a girl, then the value is calculated. And it is said that women are not given the right in Islam. Women rights are killed I am not free from this. In whatever form the woman is, whether she is face of paternal grandmother, maternal grandmother or mother. She is given right in Islam. It is called a mirage, the person who dies leaving the goods behind. Everyone has a share in those goods. A thing comes into existence. There are many people in our religion. The girls are given share. It is called Tiraka, it is called Mirage. Where it happens. The women there are absolutely self-dependent and they have no shortage of money. Anyone can do business in our country. People get married and marry their daughters. A lot of money is spend upon in name of dowry, in the name of food and think that they have paid the right of our daughter or girl, although they did not pay for their daughter's rights. If we think you will not have to give mirage after death then you are thinking wrong. If you have spent Rupees 5 lakh today, then in future your budget might become rupees 25 and 50 lakh, then the girl rights might be killed. You have spend rupees 5 lakhs and but she should have got rupees 25 lakhs, then 20 lakh rupees remained. Then it is seen that what the man who died have left behind. Then from that money or good mirage is calculated. There is a verse from the Quran Sharif that it is said that whether it is men or women, the double part is for woman, for example, in a house. The father has died or departed from the world, the money he has left behind. For example, there are 2 brothers and 1 sister in house, and the money father has left is rupees 3 lakh, then it is divided in 5 parts, one boy will get 2 parts, other boy will also get 2 parts, and one part which is left is given to the girl, this is how a mirage is divided and distributed. This depend how much the person has left behind when he died. In that way mirage is calculated. And where it is being implemented, then it is tried. Then the shroud is brought, it is choice of the family to select the color, but white color shroud is preferred as it depicts peace. For men 3 clothes are brought, and for female 5 clothes are brought in shroud. Then comes the bathing (gusul) part in male or female, then water is heated for gusul (bathing), it is preferred body should be bathed with water having berry leaves, leaves are boiled in water then body should be washed with that before reading namaz, wazu (cleaning of body) is preformed. Wazu is tried, because the person is dead, his body becomes tight. Proper cleaning of mouth and nose could not take place, then mouth and nose is cleaned with cotton ball dipped in water. Then his face, hands and feet are cleaned and bathed. For bathing male body male is required and for female body female is required. After the body is bathed, thhen shroud is spread over the body. For male 3 things are there in the shroud which contains 1 lungi, 1 kurta and 1 lower which is long enough then we put kurta and lungi to the dead person, after putting all this, one sheet is spread to cover the entire body. Then the body is tied, so that it does not open. For female there are 5 clothes which include, 1 lungi, 1 kurta, 1 long sheet, 1 long scarf and 1 cloth to cover chest. Then the body is

covered. After putting 3 clothes over male and 5 clothes over female, the body is kept over bed like cot, then prayer is performed, namaz is read for that person. Prayer is performed for children, whether elder or younger, namaz is read for everyone. We pray that person should be forgiven for his deeds he/she should go in heaven. <children, neonates aged< 1 month, Stillbirth and abortion> the child who is small, innocent, had done no mistake. There is concept for small children. Small child will become recommendation for his parents to god will make way for his parents to heaven. If he wants to enter heaven, then the last prayer (janaze namaz) is performed in different way. We are sad and mourning for his death, make that child as recommendation for his parents. After that child is taken too cementary called as baadabta, in cementary child is laid over. After lying down, we turn the face of the child towards which are praying, namaz is read, then his face is turned, there is some space over it. There is so much space that person can sit up and down, after the wood and stone are placed on it. The stones are kept. Then it is packed so that no objects like soil or something fall on it. Then the soil is thrown over it and it is made equal. And then after putting the soil, we stand there and we pray for the person who dies, we pray that Allah forgives that person this is the way we bury someone who has died. <stillbirth> See the child/little child who is just born and then died the same process is followed baadabta, that the child is bathed and after that clothes are put over him. After putting clothes namaz is read and after reading namaz we bury the child. Same honour is given to child who is given to adult. But the child who is born dead, that child is not bathed, shroud is offered but no last namaz is read for that child. The one, who is born dead even if he has made a voice after death, has screamed, shouted even then, we will still believe him that yes he is born alive. If he is born dead. So shroud is given, after putting shroud we will take him to his cemetery and bury him in the same manner. But we do not bathe and do not offer prayers. After burial there is mourning period, here no one mourns for more than 3 days. If someones husband has passed away, then we understand that it is feeling that crown has been taken from her head. Support has been taken away from her. Then it is ordered to mourn for atleast 10 days to four months. In the midst of this mourning, it is said because her husband has died and the husband has passed away so the fun is lost in her life, love is taken away so she should not get ready and do makeup. The way married women usually get ready she should avoid getting ready in that way. We should not leave the house if there is no work, if there is some necessary work she could leave it is different issue. But if her needs are fulfilled then she should not go outside the house. 6 months period is long time, in which if she wish she can marry Is ... But if there are more people, its need can be fulfilled ... So it is said that you should not go out of the house and go out of the house ... and 6 months is a long period of time in which if she wish she can marry again. It is obvious thing that if that woman is now 25 years old or she was 25–30 years old and his husband has died. Then it is said that she can marry next, then after passing through the responsibilities. After 7 to 10 months she can marry. <If the child has come to an end, then they will mourn for only 3 days>

3. **Please describe the differences in rituals and practices according to the place of death and natural versus premature death like a child and at the time of delivery/birth.**
   (Probe: death at home versus death at hospital)
   No there is no difference. Rituals and practice are performed in the same way whether death happened at home or in hospital. All things are same then body is buried in the cementary.
4. **Please tell us about the requirements for burial or post death rituals in the religion.**
   (Probe: death certificate, timing, presence of relatives, cost of the procedure)
   Death certificate in us is a document which we get from the cemenatry after burial. It is government document it is a slip in which father name date of death is mentioned. This slip is made after regular procedure. This slip is reference for future in some work it can be used if someone needs for future work. This slip serves as a proof that the person is buried in this cemenatry on following date. This is made possible through management committee of cementary.
   <timing>document is issued later because people do not take at same time of death. Although you may get that document at same time hand to hand if some requires, otherwise one has to take from the office.

5. **In your view, what happens to the spirit, when a child/newborn dies? What is usually done by the family and/or community to help this happen? (Probe: what happens, what things are done, what are not done, what happens if they are not done)**

The face is same as it is, everything is there, and there is no change in it. Life has been taken out, now after extracting the soul, it has become a corpse and meaning the corpse to which we speak it is an Arabic word that we speak after having lost life has became a dead body. Now the corpse to which we speak Lashaiya means nothing. Whatever it was, it was spirit, it was life, and because of that spirit, it had a movement… it is moving hands and feet, the brain was doing its work. Heart was doing its job. Tongue was doing his work. But when the spirit is released. After dead, the movement from inside the body was over. The brain has stopped doing its work, which has come out from inside, it is spirit. The one who comes out of the body is spirit. When the spirit comes out after that many relatives are there, there are people who come and meet. This is a matter to note. That what we loved, was the body, we were in love with spirit or it was obvious but not from body. Body still exists. As before The child who is innocent in a way, has commited no crime, has done nothing wrong, so he is clear from all the bad deeds. The religious picture we have here ... Our prophet said that the souls of such children, angels are very well dressed in good clothes, which contain fragrances. They take it and go to the fifth roof is a sage on the sky called our sage Ibrahim Isra Tusri. The souls of such children go there, to them. Because all the prophets who have passed through Israel, they are called grandfathers of all the prophets and we are in the same way as the spirit of such children. The elders play in the same way they play the same spirit near them…. While he is an innocent child, he has not committed any crime he is clear from the crime.

<What happens when the things are not done> the child's soul will not have any problem but he will be told from social court that you are behaving indecently with him, we not doing well and society does not look at it with good eyesight ... the beauty of society is that your sorrow is our sorrow ... you are happy, it is our happiness. It is considered good by society and in this society and the world is a relief and a security for society. The child is innocent, there is no crime on them, so namaz is not read, so that they go to heaven, Namaz is read because as it recommends that child for us, we the people for those who pray. His prayer is different from the prayers of men or women…. That is the prayer that we study. , For the boy or for the girl. It means that our child has died. Make us change the child of the person who has died, and we are the parents of that child for the people; Make it a dream or make it a way for you. So tomorrow, that child will recommend to his parents and people that it is our parents that their mistakes will be committed, they will be guilty.Allah will recommend in the court of Rabb Izzat that our parents should be saved from astonishment and going. In this way, that child will recommend on the Day of Judgment, that child was a kind of Allah. , Allah has taken away from us. So that is why we do not offer namaz to go to heaven, but rather we are praying that it will recommend to us and in a way It has become a way for us.

6. People are usually sad when child dies. How do people in the community show their sadness? (Probe: what is done by the parents, immediate family members, how does the community and relatives support in this)

There are two things, first when someone dies, whether child or adult, relatives, neighbours should go to house where death took place and express their regret and console and support the family, they should get involve in family sadness. This is our beginning phase so that at least the person who was in possession has taken it, is comforted and our prophet has said that if we snatch someone's love and that on him. He agrees, he is happy, so will give him paradise and we will not agree on anything less than paradise. By doing such things, we give him peace. To be involved in his work, to carve the grave or to bring the shroud or other things like that, people help a lot in that. And it has also been said that if someone in our house or someone dies within our religion then food should be delivered to his house for at least three days. People of the neighborhood should give food in their house as those people are sad, they are worried right now and cannot cook and cannot do such a thing, at least the neighborhood people should cook food at their homes and send the food to the family so that they should not feel troubled or feel sad.

6. **People are usually sad when a pregnancy ends with birth of a dead child (still birth). How do people in the community show their sadness? (Probe: what is done by the parents, immediate family members, how does the community and relatives support in this)**
   <relatives support>in our religion it is said, the neighbours and relatives should provide food for atleast 3 days to the family where death took place as the family is mourning during that period. Food should be prepared by neighbors as family should not be troubled to make food for others.
   There is one blessing in our religion which we read when someone dies, "dua" it means that we are for Allah and Allah has created us, and we one have to return to Allah only. A person has been made from the soil and after dying in this soil, he will be buried and pressed and then one day Allah lock will come in apocalypse again. You will grow from the soil the way grass and vegetables grow. Allah will live all such people. Reaction is tremendous and trouble will be great for them. We people cannot feel that pain it is only felt by the parents of the child whose child has died.After a few days, when the child leaves, it it is very difficult for the parents who have brought the child in world, fed him, played with him then when the child dies it give so much sorrow and makes difficult for parents to understand that situation. This could not be explained in 2 or 4 words. When the child is born every parent thinks, our child will grow up and will take care of us in future. And when child dies in womb, then obviously the parents would feel sad. Sadness will happen in every situation whether the person who has come in the world has died or someone died in womb. The body is prepared accordingly and shroud is offered.
7. **How do the parents and families cope after death or loss of pregnancy/death of the child at birth?(Probe: behavior, spiritual practices, family and relatives role)**
   Sadness always remains somewhere, because man has to live his life ahead and within each person's life, sometimes there is happiness, sometimes there is sun or darkness, sometimes there is peace, sometimes there is trouble, it keeps causing troubles…. Thinking that it is sad today, but tomorrow there will be happiness. By thinking like this people will forget sadness.
8. **Is there any social stigma or taboo related to family or woman in relation to her child's death or stillbirth? (Probe: blame, spiritual factors, effect on family or children or subsequent pregnancy)**
   It is not necessary. Human do work on their own. But the purpose of delivering that work is not in our hands. We and you have done some work. There is a force behind that work. There is some kindness the name of that force is God. We try a lot sometimes for some work the result is visible in front. But again that cannot be accomplished, despite our efforts, there that power who is the evil that is not allowing it to reach its end… It is the power of God. When all the powers of human being are exhausted from there. The power of God begins. So, if someone's children are dying again and again, it is obvious. What is the option other than to have patience in it. We see that there is no mistake due to medical problem at the time of delivery or the mother got proper protein or diet during pregnancy or not. So, in the future for the birth of the next child, full treatment goes on taking the advice of the doctor, but if it is the case of recurrent abortion or stillbirth then there is no option but to have patience.

   Knowing cause death and implications of procedures
9. **In your view, how important is to know the cause of death? (Probe: how this can help the parents/family and others, how is seen in the community)**
   If the doctor, under whom the lady was getting treatment, says that you should know the reason, so that same condition does not happen again. Then it is possible in that situation.
10. **What are the beliefs about post mortem (or autopsy) in your religion? (Probe: acceptance, religious and spiritual concerns)**
    Post mortem take place in accidental cases, or in case of murder, in which it is important to know how and why the person died. So there is a legal requirement, there is also a legal process, so there is no problem in following it. Yes whether it is child or a grown up if there is a legal requirement then it can take place, our religion gives permission for this.

11. **After a post-mortem is done, are there any modifications in the rituals done in the funeral process? (Probe: body preparation, mode of burial/cremation, post burial/cremation practices, related cost implications)**
Even after the body is made to cut in post mortem, the last prayer took place in the same manner last namaz is read in the same way, no change occurs. Whether it is child or adult bathing take place in same manner. After bathing shroud is offered and then body it taken for burial in the same way. And we then bury the body.
12. **What are your views about collection of the tissue and body fluid samples for identifying the cause of death? (Probe: necessity, religious aspect, time needed, etc.)**
I think that there is no need for post mortem if any such technology has come it is obvious that in the era of medical science such technology has come. If the doctor considers it important and in future woman can get pregnant and have child then there is no harm in it. This would be much better, there would be no cutting or tearing of the body as in postmortem whole body of the dead person is opened, by this technique this could be avoided. We think the person whether child or adult deserves respect, even after death, we try that his body should not be disrespected, when postmortem take place of the body then whole body is opened up and is messed, but we avoid that no such thing should happen. Here if you are saying that by extracting some blood or fluid from the body through the syringe or needle etc. it can be detected ... If this is the way to know the cause of death, the way is very good. Also there will be no disfigurement of the body will happen. But nowadays the technique is going on, in that whole body is opened up, everything is checked that how death happened, but in that whole body is disfigured. Main point in technique should be that whether body disfigurement will happen or not.
13. **How parents and family members can be approached for the tissue sampling? (Probe: who, when and how should approach and whom)**
Need is like compulsion, if you think and doctors advice for this, then it can take place. It would be good if the doctor himself explain to patient relatives. Because if the doctor will explain then it would be better, they will not listen to me. This should be explained to parents of the child. It can be explained through social sites, television, and news in seminars etc. if we explain such things then it will be better. It is obvious if one person doesn’t know about some technique it should be explained to them that this technique has arrived, in which sample would be taken through syringe and no harm would happen.
14. **In your view, what factors are important for acceptance of the tissue sampling by the parents and family members? (Probe: desire to know the cause, trust, confidence on the health care provider)**
See if it is needed, then it can be done, if it is not needed then it is not necessary to check how the person died then it is not needed.
15. **In your view is there any modifications in the rituals done in the funeral process needed with the minimal tissue sampling procedure? (Probe: body preparation, mode of burial/cremation, post burial/cremation practices, related cost implications)**
No
16. **Any other opinion/ suggestions**
The thing is that the child who has died was someone son or daughter, someone heart, every parents has wish for their children, when the child dies there is sadness among parents, neighbors and friends, everyone should give comfort and console the parents and family. No matter whether you are Hindu or Muslim you should equally involve in that situation and spread humamity and spread peace. Once nabi sahib was sitting when someone last journey was coming, then he stood up and said we should respect the person who has died, and spread humanity. We should get involve in someone sadness as well as in someones happiness.

In-depth Interviews-Religious Leader-03

1. **Basic demography**
   1.1. Religion served/ followed- Christianity
   1.2. Designation- Priest
   Death and related practices
2. **Please describe the rituals and practices followed around and after death (Please describe these day-wise and duration). (Probe: for adults, children, neonates aged < 1 month, still birth and abortions)**

After the death depends on how the person dies by the law of our religion catholic, though the person dies naturally death or in the hospital, hospital means the hospital gives the certificate of the death.

For the child: - the same rituals unless its stillborn that is of the hospital. If the child who is baptize dies then we don't even ask when he died 1st inform us the person has to produce death certificate, there is no any other way.

For old age: - if the old age then we may consider it is ok, the person was sick, it's ok kind of thing because there are also problems they don't know when the person dies for eg. Midnight they have died and early morning may be around 8 o'clock or 9 o'clock they keep on waiting the person there is no response then finally they call up father till person is dead, the body is cold no doctor ready to give the certificate cosiderly the age factor we ask the MCD person to come and tell the person is dead then we go for…… but for children and adults we have to get the death certificate, there is no any other way neither at the cemetery we allow them to bury. The cemetery also needs for the person to conduct the rituals we need the certificates.

Stillbirth: - that is of the hospital, if the parents ask for the burial we usually do it. They directly bring from the hospital. We don't bring it to the home or to the church. It is directly to the cemetery where we bury.

Abortion: - church is totally against the abortion, they don't even come to the church. They know that we don't allow. There are no rituals or burial rituals according to catholic faith, because we are totally against even it happens naturally abortion they usually go to the hospital. What the hospital said they do it. They don't bring knowledge of the priest. Things are happening one or two incidence but they never tell us. Latter on we will ask something we come to know, when the ask they did tell it was a natural abortion father because we don't take it under consider because there are no burial rituals for the abortion.

3. **Please describe the differences in rituals and practices according to the place of death and natural versus premature death like a child and at the time of delivery/birth.**
   (Probe: death at home versus death at hospital)

If the person dies at home, we before the conduct any rituals we ask them to get the death certificate from the hospital that itself says that we are in the favor of autopsy and one has to go for the autopsy otherwise there is no any other way that we conduct the rituals.

They have to produce that to from either from govt. hospital or from the recognized doctor with his sheet, signature on his letterhead where he says the reason of cause of death without we don't go ahead. Once the person dies and they get the death certificate they usually wash the body, they get it ready.

4. **Please tell us about the requirements for burial or post death rituals in the religion.**
   (Probe: death certificate, timing, presence of relatives, cost of the procedure)

Timing: - before sunset burial has to take place after that we don't conduct the rituals. Death is at night then it is the following death or they take to the mortuary, they keep it there as long as it is convenient for them, at time what happen is that if their relations may in south or in abroad. They allow to come at times their relations say allow to come so we were keep the body in the mortuary in any of the hospitals mortuary then after 1 or 2 days or after a week even after 15 days but burial must be happening before the sunset.

Relatives: - everybody is allowed in the cemetery even the child is allowed.

Cost: - The church doesn't charge anything for it, the priest reaches to the place because we usually bless the body and there are small prayers, we do the prayers. Then we also bring the body to the church for the

pray, neither the church or the priest charges anything. There after that we take to the cemetery where it is burying even for the burial rituals. The priest or the church doesn't charge but there is the charge by the cemetery. They do the charge because cemetery is looked after by some association. for example, it is for all the religion it may be or for all the Christians, there are so many denominations in the Christians. So everybody united, they do it that should be somebody to dig the ground, somebody to make the coffin, somebody to look after, otherwise anybody or everybody enter into the cemetery. Those cost need to be born and the ambulance which takes the body to the cemetery all those things. It depends on the cemetery eg. There is cemetery land which is allotted by the govt. then the cost is less it comes around 5 to 6 thousands not including the coffin. If the land is of the church, the church is united they have got lands if that is the case they may be little higher because they have pay for the land, until now a day in Delhi there is no land. We are actually doubling or tripling the grave. Doubling the grave is allowed in Delhi by MCD, so that has to be intimated to person the MCD office after the burial, tripling is not allowed except few situations. Eg. When the tripling comes that should have been previous burial which should have been 10 plus years only then the tripling is allowed. Doubling is allowed usually after 6 years or 5 years but the tripling is always giving the prior permission. Doubling is in the cemetery told ok doubling allowed but they will see how many years are gone.

5. **In your view, what happens to the spirit, when a child/newborn dies? What is usually done by the family and/or community to help this happen?**
   (Probe: what happens, what things are done, what are not done, what happens if they are not done)

Spirit concept means it is a common belief and understanding, the human is made of body and soul, ones the body dies with the great reference we burry, why we do burry that's our belief our belief is that the death person will be raised life ones again it is not second life, it is not rebirth, who don't belief in reincarnation, rebirth in Christianity we call it resurrection, death after life the person continue to live the earlier the person was living in time stipulated time around earth may be 10 years, 1 year, 1 month, 100 years, 107 years after the death not with the physic but with his soul he is raised either he lives in heaven or hell according to his or her did but everyone will be raised so resurrection is for all but in the resurrection whether they are going to heaven or hell that is determent by work that one does so we belief in resurrection and life with god in his kingdom. It is same for adult and child everyone because the soon after the conception we belief that's why the abortion is not allowed in the holy catholic church ones the person is conceived, we consider whether it is one month or 2 month or 6 months grown child in the womb or 9 month grown child in the womb even from the moment of conception until the person is delivered it's a human being because it is only intension of god and the expression of male and female who are married they loved. So that's why it needs to be given respect honor, if you go to any Christian cemeteries you will find some graves small ones in some places there are when you ask the cemetery person, the person will say it's a stillborn baby's grave. We don't just throw it or something but still it is given our references.

6. **People are usually sad when child dies. How do people in the community show their sadness?**
   (Probe: what is done by the parents, immediate family members, how does the community and relatives support in this)

After the burials, it depends on the family and, In Delhi we are not just from Delhi, they are from UP, Bihar, Jammu & Kashmir, pahadi those people are less but there are people from Kerala, goa, Maharashtra area, Tamil Nadu, Andhra Pradesh, Karnataka from these places, each one has different rituals. Different rituals mean there are people we do visit after the burial. We visit the cemetery may be on 3rd or 5th day then after that we visit the cemetery on 30th and 40th day. These are the things people usually visit and they light the candle, put over the agarbati and malas, they make small prays. If it is possible they take the priest along them. It is just praying that are conducted. It depends on each person. There are people they may not able to go because of the other constrains. Eg. They may not even money to go to the place so they visit only one after the death may be 3rd or 5thday visit. They may not go on 30th or 40th day. But every year after the death people do remember that day and visit the cemetery. The 2nd November is all soul's day irrespective of the denomination of Christianity. We all visit the cemetery. The

people whole day spent in the cemetery, pray for, put mala and agarbati. The child is baptizing then after death we do visit on 30th & 40th day after the death.

The priest usually visits on the day of death, 3rd day, 5th day & 9th day and we have prayers in church where the lots of community comes together. On 7th day we have and on the 40th day we have mass prayers, where whole congregation come together for eg. The areas around we have got 500 families. They all come together, where one person in the family they come together. We look after the needful and we support. There are some poor family we also support from the church for the burial cost, there are some children or somebody because they go work for the certain time so in those days we financially support also.

7. **People are usually sad when a pregnancy ends with birth of a dead child (still birth). How do people in the community show their sadness?**
   (Probe: what is done by the parents, immediate family members, how does the community and relatives support in this)

That depends on the family expression, there are people who don't come to the church for few days because whatever to the others, others have not seen the child but for the mother it is whether it is in the womb or outside, for mother it was a child so they do feel bad about it so they do morn, they do after masses, prayer in the church, we also had one she was almost to the full maturity, she had come home for the delivery, the you must have seen people taking the girl child girl to the home for the delivery you know, in certain month or something like that she had gone but miscarriage are happened the child was stillborn so nothing could be done and she was very bad feeling very bad, sad, she did not even come and join for job those days she took 3 months chhutti (holiday) because of that, then she came back she was already out of job, yes still she is looking for job only.

8. **How do the parents and families cope after death or loss of pregnancy/death of the child at birth? (Probe: behavior, spiritual practices, family and relatives role)**

<coping mechanism>We not work, community in the locality for eg. There are areas like janta flats there are janta 1,2,3,4… there are around 400 families but catholic are just 10 or 12 family, after the death it depends on the family, they might have 7 pray or 9 days prays every day evening, these come together in the family that they go. There are families, they make food for that family, they take food along them, 9 days it happens. There are some people from Jharkhand they do it for 30 days. The tribal community from Jharkhand, Chhattisgarh, Orissa and north east. They come together for a month every day evening, it depends on the timing may be 6 o'clock, 7 o'clock, 8 o'clock come together in the family. They pray for that particular person and they spend little time. They also take food along them different family takes food different days not for the whole group only for that particular family so that they don't need to spend time on cooking. They feel wanted, loved and cared for the same community, which is in the locality that is for irrespective of any age. Prohibited the family is ready, there are sudden families, they have work after3 or 5 days. They say it's enough" ham ni hai ghar pe aane ki jarurat ni hai."

<pregnancy loss>It is usually the person has to get back the relations after help in some family the acceptance is faster, in some families they take time to do that, in some families like we are all in whether our faith is there or not we are all human being, the culture is different place to place, in some places in the in-law's houses they start scolding it is because of you this and that then the person feels so bad they doesn't even come out it happens in some palace so its depends on the people of mind sets.

9. **Is there any social stigma or taboo related to family or woman in relation to her child's death or stillbirth? (Probe: blame, spiritual factors, effect on family or children or subsequent pregnancy)**

From the religion side there is no taboo but there are things which are attached to their own customs from the area they come from. Eg. Tribal community from Jharkhand, Orissa and Chhattisgarh, those people who are here, if the male person dies the female or wife doesn't come out of the house for 40 days, doesn't come out except for the church prayers. That is also on the day of burial, after 3rd day, after 5th day, 30th and 40th day. After the 40th day they mingle with people, they go for work and other work. It depends on the separate areas and customs where they come or locality. They try to follow here otherwise from the religion. There is no restriction with the irrespective of the age.

Knowing cause death and implications of procedures

10. **In your view, how important is to know the cause of death?**
    (Probe: how this can help the parents/family and others, how is seen in the community)

We not only the Christianity even before Christianity came into existence, the bible itself says there was a person called 'Tobis'. He used to bury the death. What you have read 'Red cross' or 'nineteen girls'. Whom you call in the medical line all those people have follow the actually Christians values and then death fathers barring called the death. The work of mercy you have to do some charity which comes out of your own love, faith and the due honor needs to be given to the body which is dead, so accordingly it has to be bury, no ill treatment to the death with the great honor & highest respect you can give you must give.

11. **What are the beliefs about post mortem (or autopsy) in your religion? (Probe: acceptance, religious and spiritual concerns)**

If the death takes place in the home, they usually call up some neighbors who usually inform us because the priest will be the 1st person to know. What happen and every family where the death has taken place They wants priest to visit there and that is prayers which we say that's why they inform priest 1st. There is time if the death has taken place at home they don't know then they have to get the death certificate when we receive the family we tell them how to get the death certificate if they are ok, there are family of 2 persons one is dead and other one is alive he and she can't the run around the hospital to get the death certificate then we our contact we try and get do for the same.

12. **After a post-mortem is done, are there any modifications in the rituals done in the funeral process? (Probe: body preparation, mode of burial/cremation, post burial/cremation practices, related cost implications)**

We bless the holy water only this is not gangajal which we like to wash the body. If you ask for blessing outside we use same holy water, before somebody goes to the hospital for the delivery off the baby still we bless with the same water. We bless the church with the same water. So in that we this is not something else. We usually try to follow the science also so that it helps the faith is without reason is waste. That's why the science should also go with faith otherwise it become blind faith, vary superstitious to get that, so the science always recommends after the autopsy, if you wash the body there is lots of tissue or something which go up which can harm the locality, ground and the people.

13. **What are your views about collection of the tissue and body fluid samples for identifying the cause of death? (Probe: necessity, religious aspect, time needed, etc.)**

About MITS: - there are no barriers to autopsy. Yes, it allowed in our religion.

In Christianity there is nothing which prevents from doing it, there is nothing connected with the faith in this, it may be because of their own culture, cultural setup their own mindset otherwise faith is nothing to do with these things yes.

14. **How parents and family members can be approached for the tissue sampling?**
    (Probe: who, when and how should approach and whom)

The somebody is already requested in the hospital and requesting them they will surely allowed, see there are people with mindset everywhere you can see nothing to do with faith, this is nothing to do with faith, taking samples, plus if there is certain people who are ready to convince them and to do things they will surely accept, yaa there are people I know that recently the people who died they have even donated all the organs they have even they own son and daughter they told here in Delhi itself they don't whatever that can be useful you please take so that is always possible that this is nothing to do with faith actually according to me. It is something to do with humanness even after that ones the person is that mean nothing can be done by crying, by not allowing somebody to the useful all those things. I used to tell my own friends, when I die you don't burry me if it is possible give to some medical colleges for the they can use it. It depends on the person's mindset.

15. In your view, what factors are important for acceptance of the tissue sampling by the parents and family members?(Probe: desire to know the cause, trust, confidence on the health care provider)

According to me when the tissues are taken that child cannot be saved in any case it is dead but in future there are many who can be helped, if they are able to find out the cause whether it was because of the mother or anything which is the common factor like the pollution in Delhi or water factor or the medicine that with which they helped by any private hospital or govt hospital all those things can be found out and which can be a real help for the future in prevention of mortality ratio can be prevented so in that sense these days people are still little more open sudden things and to the science. It should be allowed.

16. In your view, is there any modifications in the rituals done in the funeral process needed with the minimal tissue sampling procedure? (Probe: body preparation, mode of burial/cremation, post burial/cremation practices, related cost implications)

Nothing

<most influencer person according to you for the promotion of MITS in the religious point of view> for us you must have seen every lawender, pope is nothing to do with India. Any newspaper that you see in the world news pops photo come at times it's a small place 105 acers no family live inside Vatican only exception is the religious who look after the pepasi under Vatican media, museum or the religious women religious who stay inside in one's community, community means may be of seven or 9 sisters who stay inside that's all, no one else stay inside everybody stays in Rome. The houses are in Rome in these 105 acers only buildings. Pope lives pope secretary, his assistant, even pope's chef he cooks and goes, somebody else serves he also goes, they don't stay inside Vatican. So also the powerful man for the whole nation with regard to our faith it is pope, if not pope like Delhi, Lucknow, Allahabad like this it is bishop of the dices who is influencer means a he is also priest but one step ahead it's an ordination by ordination we call it consecration bishop here in Delhi it is Archbishop, he is in the secrete heart church. In Delhi he is the highest person and who influence for certain things. In the Paris it is the priest in charge we call it 'parish' the local or locality for example it is k area, in k area for the Catholics it is the priest who is in charge.

17. Any other opinion/ suggestions.

In-depth Interviews-Religious Leader-04

1. **Basic demography**
   1.1. Religion served/ followed-Sikhism
   1.2. Designation-Head Granthi
   Death and related practices
2. **Please describe the rituals and practices followed around and after death (Please describe these day-wise and duration). (Probe: for adults, children, neonates aged < 1 month, still birth and abortions)**
   According to Sikh rituals, whether the dead person is small or big, cremation should be done, if somebody put body in flowing water or bury the body this practice is not allowed in Sikhism. For stillbirth also, same procedure should be followed, that is it should be cremated. When cremation is not available meaning giving fire then the body can be buried or immersed in flowing water, this can be done either for child or adult. If cremation facilities are not available then it is not necessary to cremate, then alternate things can be done. For cremating, there is no consideration for the process to be done during day or night. However, the deceased may be young the body should be cremated. Where arrangements for cremation cannot be made, there no doubt should be there about the body being immersed in flowing water or disposed of in any other manner.
   The dead body should be bathed and clothed, the Sikh symbols- kesh, comb, kirpan, kachha, karha should not be taken off. Then, the body is put on the plank and then Ardas is offered. On reaching the cremation ground, the pyre is laid. When the body is placed on the pyre then, the son or other family member or relative set fire to it. When the pyre is fully aflame, the Kirtan Sohilla (prescribed pre-retirement night scriptural prayer) should be recited and Ardas is offered, then family member and relatives return.
3. **Please describe the differences in rituals and practices according to the place of death and natural versus premature death like a child and at the time of delivery/birth.**
   (Probe: death at home versus death at hospital)
   There is no difference, all the procedures are same for both situations.
   <how death at home can be identified> The family of the deceased comes to gurudwara and tells to the Granthi or sewadar at gurudwara, then they get involved in the procedure/rituals. Other all things are followed in the same manner.
4. **Please tell us about the requirements for burial or post death rituals in the religion.**
   (Probe: death certificate, timing, presence of relatives, cost of the procedure)

When the death happens at home then no certificate is seen. But if the death take place at hospital then family brings the paper from hospital and then the cremation take place. For performing death ritual no money is taken, but the cost which cremation ground people take is the only cost, this cost is different at different places. There is no difference between cremating the body in day or night.

5. **In your view, what happens to the spirit, when a child/newborn dies? What is usually done by the family and/or community to help this happen? (Probe: what happens, what things are done, what are not done, what happens if they are not done)**
   According to Sikh Maryada all the procedures are same whether the dead person is young or adult, Akhand path (reading of Shri Guru Granth Sahib Ji) needs to be done. After cremation, family comes home, then goes to gurudwara for Akhand path, which continues or three days. During this path (procedure) all the family members and relatives take part collectively. After the path no, funeral ceremony remains to be performed. In Sikhism there is no hue and cry, lightning lamp, pind daan (ritual donating of lump of rice flour, milk, ghee) or shradh, all is prohibited. Some people believe in the concept of Panchka, i.e. they believe that if in some home more than one death has happen, more death can take place so they burn five dummies. But this practice is against Sikh rituals.
6. **People are usually sad when child dies. How do people in the community show their sadness? (Probe: what is done by the parents, immediate family members, how does the community and relatives support in this)**

Parents are usually sad after this event, all family members support each other, we also try that family should get out of this soon, and they should start taking care of their second child and also care for their other family member.

7. **People are usually sad when a pregnancy ends with birth of a dead child (still birth). How do people in the community show their sadness? (Probe: what is done by the parents, immediate family members, how does the community and relatives support in this)**
8. **How do the parents and families cope after death or loss of pregnancy/death of the child at birth?(Probe: behavior, spiritual practices, family and relative's role)**
   By keeping and reciting Akhand path of Shri Guru Granth Sahib ji, there is peace of mind and peace at home. For this purpose, whole family members and relatives take part equally. To induce a mood of resignation to God's will, Gurbani is recited or "Waheguru" is repeated.
9. **Is there any social stigma or taboo related to family or woman in relation to her child's death or stillbirth? (Probe: blame, spiritual factors, effect on family or children or subsequent pregnancy)**
   In Sikhism there is no such thing, the person who has come to the world alive and if he/she dies than this all is pre-written, in this no mother or any family member is blamed for this.
   Knowing cause death and implications of procedures
10. **In your view, how important is to know the cause of death?**
    (Probe: how this can help the parents/family and others, how is seen in the community)
    It is very important to know the cause of death, if we will know the cause of death, it will be good for future government should also contribute for this. If we will know the correct cause of death then it will be good for both children and adult, the disease will be known before.
11. **What are the beliefs about post mortem (or autopsy) in your religion?**
    (Probe: acceptance, religious and spiritual concerns)
    According to Sikhism or Sikh rituals, there is no restriction for post mortem. Sikhism norms/rituals are far ahead of science.
12. **After a post-mortem is done, are there any modifications in the rituals done in the funeral process?(Probe: body preparation, mode of burial/cremation, post burial/cremation practices, related cost implications)**
    No, there is no difference, the same procedures are followed which is followed for natural death. When in religion there is no restriction for post mortem than why the procedure will be different it is all same.
    In some instances, autopsy is not possible due to various reasons. To find out the exact cause of death and underlying disease, some tissue biopsy (collection of very small sample using needles) and fluids (blood, urine, etc.) from the body of the child after they die, as done for investigation. But these samples are to be collected as soon as possible after death. This does not cause any disfigurement of the body.
13. **What are your views about collection of the tissue and body fluid samples for identifying the cause of death?(Probe: necessity, religious aspect, time needed, etc.)**
    If MITS is to be take place then no Sikh leader will deny to the procedure because after death is hardly makes any difference, whether the body is cremated or immersed in water, or whether the post mortem take place, the deeds of the person in his life will decide his fate. And this is also not necessary that the ashes should be put in water, it can be put at the place where the body is cremated. In Sikhism, all the decisions are made by Akal Thakat Sahib. People goes to Kiratpur Sahib for ashes immersion, this is also not written anywhere.
14. **How parents and family members can be approached for the tissue sampling?**
    (Probe: who, when and how should approach and whom)
    This all could be told by doctor only, or the doctor who is seeing the patient in the hospital can explain this thing. At that time, whosoever family member is present in the hospital should be told about this procedure, or the elder most person of the family should be approached for this procedure.

15. **In your view, what factors are important for acceptance of the tissue sampling by the parents and family members?(Probe: desire to know the cause, trust, confidence on the health care provider)**
    Parents will agree when they will have full knowledge about this procedure, and their next child should not have the same problem as previous child. If the doctor would explain everything clearly to them then nobody will disagree.
16. **In your view is there any modifications in the rituals done in the funeral process needed with the minimal tissue sampling procedure? (Probe: body preparation, mode of burial/cremation, post burial/cremation practices, related cost implications)**
    The procedure will remain same, nothing would change.
17. **Any other opinion/ suggestions.**

List of Focus Group Discussions

S. No.	FGD number	Participant category	Total Number of participants
1	FGD 1	Father	10
2	FGD 2	Father	8
3	FGD 3	Mother	10
4	FGD 4	Mother	9
5	FGD 5	Grandfathers	9
6	FGD 6	Grandfathers	8
7	FGD 7	Grandmother	7
8	FGD 8	Grandmother	9

Focus Group Discussion- Fathers 1

Total Number of participants-10

1. **Where do people from your community/ locality usually go or whom do you consult during illness of children and newborn?**

R6-Near about is MN Hospital, and the rest depends on its standard.
R7- S Hospital also.
R6-Safdarjung is famous, that is the last option nearly there is only Malaviya nagar in our place, if we have any emergency cases then there is B Hospital and M Hospital.
F- Private etc.
R6- Very few people go private because they cannot afford anything by going there and even if they afford, there is over budget so even some people think.
F- ASHA/ANM do you know? ASHA clinic/dispensary
R- Ladies only go there
R- Yes, pregnant women only go
R- Mostly, MN Hospital. Vaccine, test or medicine and any other problem is coming or there is any pain or something like that.
R- For children's vaccines, the main is MN Hospital.

1.1 -for delivery

R- for delivery MN Hospital.
R- MN Hospital or S Hospital
R- By the way, MN Hospital is close
F- Your children were also delivered in S hospital
R- My in S Hospital
R- My MN Hospital
R- Sir what is the choice
R- Visit S Hospital or MN Hospital

1.2 For vaccination

R- Actually what is it that now is in Anganwadi
R- Anganwadi madam comes and gives time to get tomorrow
R- She takes (Aangawadi worker)
R6- It is like when the injections come to them, they guide that you are come. Otherwise if we cannot go then the work is done from MN Hospital. Because when the last time daughter had to be vaccinated, she kept many days in pending not a long time vaccine yet, vaccines did not come when we got it in MN Hospital. Then after that they came that vaccines have come now if you want, get it done, then we said, now get it vaccinated, it happens sometimes.
R- Anyway written in card.
R- Yes, then we said we're done

1.3-For illness

R6- That to a local doctor at a nearby clinic
R- They go to private
F- For this, do you go to MN Hospital?
R- that is too far
R6- What is in it, do not see the response doctor, look at this doctor, see 10-20 patients in a day that they are able to give more time this but in dispensary and the government ambulance medicine it comes but what they have is 150-200 patients. So we verbally told his mouth that he has a cough, he wrote it down but do not test him whether he is lying or telling the truth Is it really cough or is it coughing like this, Just said that cough is coming, give him medicines then let's go. But he was given any medicine by him, but what was his illness in reality. It is not the doctor himself, who is telling the right, whatever you are in front of you, you will tell what type of cough you have, and Cough is also TB, A cough is also normal if you eat something oily, Now he said he has a cough, just wrote again of cough, If you have a slight

headache, then take paracetamol that's it. Neither test a bit, but watching Doctor Saab is reasonable because it is no joke to see 150-200 patients daily because if you want to see 150-200 patients then you have to do it fast they are also fine as far as they are sitting on the seat So a little change in this thing is better.

1.4-for admission in case of critical or severe illness

R3- S Hospital or A Hospital

R3-direct S Hospital

R7- MN Hospital, it is an emergency case and gets referred from there.

R1- We will go to MN Hospital which will be nearby

2-Any experience of child in serious condition in hospital

R10- It is not like this because it looks at the child and not much problem is coming. So we go Private does it, it does not take risk on children will take a risk on himself but in case of child don't take risk.

R6- Mostly go to private

R4- I am going to private my niece was very sick so I was going to the local doctor, that I was no longer with me his eyes was right there also consulted the doctor, the doctor said, do one thing, take it to MN Hospital, But I didn't have much time. I thought it was time for traffic, it was 6:30 pm till Devli in the evening it took me an hour I could not go then I had to go to M Hospital. I admitted him to M hospital. He had a problem of dehydration too many his eyes were turned upside down, and then I showed him in private, showed him in M Hospital.

2. In your view, what are the common illnesses and causes of child illnesses in your locality?

R1-loose motion

R4- Normally this is illness, cough, cold, and pneumonia etc., loose motion.

R3- Cough in winter

R2- By the way, there is no such thing, nor have I seen it

R8- The reason can be any

R7- The child had pneumonia in my front house, One and a half - Death of two years is dead The first child was cold, the cold kept getting treated slowly, here and there, he should also stay in S Hospital he might not even be breathing I had not gone, I had heard, The oxygen that was given to him was also given by him day and night, so only then he did not survive.

3. In your view, what are the common illnesses and causes of child death in your locality?

R1- Failure of treatment at the right time

R3- Their care is not able to be maintained properly as it is not known what the disease is Even the doctors cannot find out whether he is illness or anything else. Or what is the matter, as illness is going on right now, in one week, illness pains are going on.

R5- It is known that if something else is coming out later, then it is late

R8- You don't have such cases

R2- Can't get actual information what is does

R4- Is so active that if you do not have the name, if you do not reach there, then get your private treatment done immediately don't risk children.

R9- What the brother said, it is the same thing that in case of children do not take risk.

R10- If you do not take the risk on the children, and then you will see it above yourself

R6- no guidelines we, the child we think is a normal fever, we see it and it is also because we do not have knowledge ourselves, what will we guide anyone At that time, we know that have fever, give it medicines twice a day.

R8- He showed comfort to the local doctor first, he did not even check him properly.

R2- And if a case worsens, then he goes to the hospital, that's what happens again. Even the family itself will not know that the actual child should guide this As if it is cold now, it should be fed hot water, or it has loose motion, then it should be fed ORS solution Because we see these things on TV, we have this thing in the knowledge that people give ORS to the child if there is loose motion So, if the mucus is then drink hot water or let it steam. What is it that we see many things in the media, we read in the news or

many doctors also give advice, so remember small things in the mind, but we do not have knowledge about the parts of which are bigger than that, so we can't get over that right now?

We are trying to identify the causes of death in children and stillbirths, so that something can be done to prevent and reduce the number of deaths in children and stillbirths.

4. In your view, would this information be valuable?

R2-Everyone wants to know what is happening
R4- Want to know all
R8- It is said that we will have knowledge that there is no further problem or no one else. 100% wish what was the actual reason then even further; it is not the only child the other child will also have it, then it has happened, then we will not know. So we should know that yes it is not ahead for us, do not come forward before us again.
R6- Everyone wants to know, but what man does not know that emerges from that thing, then he forgets the later things Now come on, now what can happen, even if it is known, one feels more If I had done so much, I might have saved myself, then I understand myself.
R1- Sometimes stays
R6- Sometimes thinks it is no more, what to do now
R7-Mostly fever
R5- Mostly doctors tell us, now what they tell, it remains in us
R6- Actual, the doctor will tell us that he can only believe
R9- We can't even find ourselves
R6- My uncle is the last 2 years old. He has his own medical store, and is a pharmacist himself. His son was slightly heavy in body, little lad in age was overweight eating and drinking in love he is admit in SH Hospital, then his treatment was going on there, so he died So at last, when you asked whether the problem happened, what was not the problem, now it is not known whether the doctor told him or he did, He was a pharmacist himself, he continued to give according to your calculations, when he felt that he was out of limit, he got him admitted to SH Hospital. After getting admitted to SH Hospital, what happened that he died? Then later found out, the doctors told or the doctors guided them or they themselves saw the reports or did not understand anything. But he was told that his heart was not able to pump properly due to overweight, so it was a death problem. That brother who was our heart was not able to pump properly due to overweight fat, so he died in this way.
R6- Last time he was told that according to age, at the age of 12 years, he had a weight of 55 kg and he had a lot of weight. He used to think that eating and drinking is healthy, but he was harming him in the body, then when he could not pump, the heart stopped working until he was gone And they did not get this much time Because of which obesity was not known whether it was from thyroid or was from someone else. He did not know what the disease was.

5. How autopsy/post-mortem is viewed by you and community members for knowing the cause of death?

R9- Sir, for this the test would have to be
R8- Only the doctor can tell
R9- The post mortem happens.
R6- I have heard the post mortem sir ji, I have heard it the man on one side of us was finished, he has a relative his family sued him. They said he poisoned and killed him All work done, he did not even accept the medical here He had such parts of his body that he was checked in Bangalore, so the reports which Bangalore people sent there, he said that his attack had come But the people at home were not ready to accept this, they did not even accept the reports here, they said that they had taken it and got it final, we do not believe it So he got him tested again through the court, then he did that test from Bangalore, then it was known to him that he was dead from the attack.
At first, he had sat down, when it came to pay the money his family members gave us so many lakh rupees. We killed our man, when he got the test done again then he said that we do not believe the reports

here, we believe in Bangalore and Banglore says number one in this thing. Yes brother, what Safdarjung people have given is a fact and it has come out of us.
R6- The people of the house wanted them, they were not satisfied, they used to say that they have killed, someone gave something. In case of accident, all the reasons are to find out what the main reason was.
R4- Mostly this is what the householders do not like post mortem
R5- This is what you say is done
R6- This is the reason, actually like us, old people say that by doing post mortem, the body parts, which are given to us by nature or which are destroyed by us, Means go here and there, when his body is taken for this, now I do not know according verbally tells things, I do not know this thing. Because when it is next time, it will be seen that it will not have eyes, yes, in the next birth. But medical does not believe in this thing, I also believe that medical does not believe in this thing, so many people do not think and get medical.
R6- But a lot of people also believe that medical treatment is right, people who have not received life donation should get many, some kidneys may work, some eyes may work. If someone's liver comes in handy, many people think the same way, that thing has become a matter of organ donation.
R2- This is too much, how will the pine be torn and tear it, your man is already dead and give him a tear
R3- Soil is bad
R5- Everyone has their own thinking, now our housemates were assuming that I donated my eyes in Safdarjung, now the family is upset, why did you do this without asking, so it is the thinking of the housemates, ours is different.
R6- You has done this job well, you have said this today, you have your own thoughts, this is a good thing.
R5-We have our own thinking, our thinking is now dead, now burning, in the fire, whatever will be left in it, what will you see in it, now the family thinks, oh this is that, you have done it wrong, in the next life Eyes are not met, then the same thing comes around again. Our thinking is that we have donated our eyes to someone.

6. What are the benefits of knowing the reason of cause of death?

R- Those people do not want to know, they think that what has happened, who had known it, it is gone, now after knowing it will be more regretful
R5- The result is death, whatever it is, accident, light current, whatever happens, now the people of the village say that it was time, it was written, it was like this
R- The person who dies can also die by stumbling and does not die due to the downing of many tires, yet someone remains.
R5- Scientific reason is hard
R6- Yes, science seeks the reason that no one else is there, science brings some way forward
R- Previously, the patient died of TB 100%, but now it has become stagnant, then it is the same thing.
R- See this discovery, now the treatment has started, that we finish something, it was written that it is about to die, then that thing is over.

7. How do you view minimal tissue sampling procedure as a method for knowing the cause of death?

R6- Yes, he will also have his second line in the medical line, to know that without actual fussing. If it is, then the householders will agree completely, then why not?
R2- Apart from medical, there is another place in India which many people believe is superstition, people believe it more.
R4- This is in India donate full body for post mortem to see whether or not
R5- Don't even try to tell
R1- If we know only then we will tell further, we do not know yet
R6- This is again the easy way, it is so right, what is it that the person in front will not even be sad that according to which time the body is fluttering, according to that the body is just fine, there is no visible,

you are telling a mild injection type or not, in inserting injection type or thin syringe type, yes, any family will be satisfied with it that we will actually find out what happened with it.
R8- If someone gives it by writing, then only you know, get this test done
R9- Only the doctor knows everything

8. How the parents/families of a child/newborn who has died, should be approached by the hospital doctors/staffs for the minimal tissue sampling procedure?
R2- This is the only hurry they have, as soon as possible and take it, and finish the story, it was done
R4-No
R6-I have listened to the tumor
R1- I have got the needle done, I had got a wife done, I have done a mother, they tell it to the mother, it was lumped, she had got it done, my wife had got a lump in her chest, she had got it done too. It was not difficult, very easy. The hose done was my wife.
R7- It is to be told, as if sitting in the house, it keeps on talking that I went to that meeting, it was told to me that post mortem should not be done (MITS), this is a better way.
R8-Our environment is very social nowadays, you will tell many things in your house, then they will take the matter forward, then according to this, the matter will continue to move forward, any good thing or bad thing, tell the people ahead.
R5- She definitely goes ahead
R10- Now we will go, ask what is happening, any householder or friend, I will tell, he will say it has been told, and then he will tell it in future, if ever, he will tell accordingly.
R2- You should tell the doctor, we will not listen to you a little.
R5- He will say whether you are a doctor or we are a doctor, he will speak directly
R7- If you try to say anything, they say this, we are doctors or you are doctors, you sit on this seat
R3- You go too many, you go to the local dispensary, go to the private, go to hye fye, if you go too many, they will give you the same answer, are you a doctor or are we a doctor
R6- We don't listen
R3- We are checking, don't you put time while checking, the band will come out, we will be beating the line, this is mostly what happens, because this is going on nowadays.
R10- Do not let go for an hour inside
R3-This is going on nowadays, it is not that the government dispensary will go there, it is like this, many of you go to the best and also private, there is also the same situation, because senior doctors are rarely available, many also go, you do not get time.
R6- What you are saying is that in the medical line it is something that we can find out, because of which reason we died, then there is no such trick to check it. Is there any timing of that after some death? 24 hours or two days?
R10-No soil is allowed to be kept for more than 24 hours
R5-After death, no one will speak to us in this way, it should be checked, because everyone is quick, because after the death, only the last post mortem is left, so no one can tell that brother has to check it. Because of its death, it will not die.
R7- If someone becomes an accident, then only Banda will do post mortem, no matter how crane is, police case is there, then you have to go for post mortem.
R6- No one can stop him from getting post mortem
R4-But if a normal death has taken place, then no one will get it done, whether it is post mortem or check that it has died due to this, yes a little bit is known in the house that there was starvation due to this or that. There has been a long illness before that, but after a long illness, no one will be able to know that it will be known that it will not prevail only, now it is safe, whatever it was, let it be alright, now do this story.
R2- Only you can tell
R6- Must have knowledge of that thing
(Whom should we approach?)

R4-But you will know that only then someone will tell, as if there is a solution too, then this is the solution, brother, if it is done, then it will be known, but there is no solution, only post mortem after death. If there is any solution, we will tell it again
R6-Is there a good way to find out after death? Okay, who should be told in the family? Yes, then whoever is responsible for the family, who is tolerant, who digests and feeds that thing. There is a responsible man in every family
R2- He looks already
R6-He is his father, he is a mother, he is his sister, he is his brother, in that environment, it is known that the man is responsible, then it should be told to him that this is the problem if you want to know, if you are To know what was the reason, everyone wants to find out, because that too happens in misunderstanding, did I not do it in misunderstanding I would have done it like this, would have brought it like this, would have brought it ahead of time or 6 months earlier, I would have done something, there is no way to return but to give it clarity in my mind that they should advise it further Be sure that happened to me but don't you, if you see something like this, that's the reason to answer that thing That responsible man should be told that this was the actual problem. It should not be misguide like what doctors say "Time is over, you can take it" In most of the cases, the answer that they get is only that the time is over, you are late and move more Only a party or a family pressures that they had just come, now because of which they came or did not come, they do not know What was the disease for how many years, he came to know that two months ago, the disease was from 20 years, so his arrest was not detected, it was only known that the disease was carrying it, it just happens.
R4-Doctors also do more in it, oh man, it will not even save, do you do this or do it, do it fast. Now my niece had a tumor, her growing doctor was seen to be of 2.5 kg, nothing happened in the last, then she is a friend of mine in AIIMS, her father is a doctor in the army. Go to AIIMS They had gone to Agra, they had asked for 9 lakhs, even in 9 lakhs, the child was not a private one, but when he came to AIIMS, he gave a little tooth to the doctor, or whatever the doctor requested and talked to the army men in 10 days. He treated the girl, today it has been three years, she had developed a tumor of 2.5 kg, had a tumor of 2.5 kg and was a child of 2.5 years, it had increased so much that it was only visible, no one agreed, Everyone was ready to do it, but it was not a matter of responsibility, it increased, just took it to Rajasthan Ever taken to Jaipur.
R6- They also got the country treatment done by Ved
R4- Everything was done but when this hoa, meaning when we went to our end, was not taken, when the pressure came from their side or when it was hardened, when we hardly took it then it was done. But when we went, we had not heard from her side before, she was not ready to do it at all, when her pressure came, she operated the operation in 10 days, today that girl is perfectly fine, it has been three years now, every year Her test is from Uni's reference and the girl is right now. We were told that she will get it done, she will not become a mother, now she was also afraid that the child is brother. No, it was also said that no one can do anything but you are not ready for the right operation, but you were making excuses for 70-80 ways, the doctor is also like this.
R6- Every family will agree, for this, there is no such big thing, it is just that in the family, it is agreed with the right time, at the right time, the right guide will be agreed, 100% will be agreed. Who is the responsible man of the family who digests and agrees and digest that thing
R- That thing should be told to him, when he died, you can give the news to the person who called him first.
R6-No, it happens that the doctor said, call it, but in front of the doctor stands the wrong band, all the members of that time family are there, who go on at that time which is suitable, they say yes brother tell doctor what It is visible, but what is the situation in which the party in front who could not understand that thing or could not understand it, must have seen a military film, what happens in that military film is that the same condition is that In the family, everyone knows that my brother, my husband, my son has died But any family member from outside who used to guide who did not speak before, do not tell anyone that such expire has happened, he has gone on duty, because he knows that if father finds out So Dad will get an attack, because Dad was Hart's patient, If the wife comes, then her daughter will be seen, the sister

used to think that she would be seen in the family, but in fact everyone knew, everyone knew the matter but they did not want to tell each other, no - no, okay Everything is fine. The front should know how much the front can digest.
R6- The report that is handed over to the right man is to tell you that it is the job of a doctor, but you will give the report, but what will or will not pass on this bond later, it may be that there is some minor normal disease and their family Tension increases and, the dying person has died, now there are so many members in the family, they are also worried, that these people have been negligent, if they had been admitted two months ago, it would have survived, given the meaning of bigar , Bigger means there will be tension, it will not happen, the men is gone, gone, The doctor said that it was a little bit like giving medicine pills, it would not have been recovered and this was the disease, its disease is known but then it will also remain in their mind that we have learned the disease but we have taken carelessness So we lost you man or lost your family member.
F- How to reduce it or not
R6- Then there will be questions in that, not that the doctor said to the family, he will say yes brother wants to know, everyone wants to know how to kill.
R- But even before we die, we can know
R- Oh, it will happen only after death. No, as I have been saying, if any child is ill, has been ill for a month and has not been killed, then we can get it done only if it is found out realistically that we should wait to die then we will know
R6- There will be a solution first
R8- It happens in Safdarjung also, we do not have these tests here, get it done from outside, sometimes the date is long.

Focus Group Discussion- Fathers 2

Number of participants: 8

Illnesses, death and care seeking practices

You are aware that several of the children in the area/ community fall ill/become sick and some of them die.

1. **Where do people from your community/ locality usually go or whom do you consult during illness of children and newborn?**

R6- There are some doctors here in T, I go to them. I visited many doctors, but there was no difference. As we got some good result with one doctor, I feel comfortable, so I go there and go nowhere
R4- Our doctor is there in nearby area.
R7- Sir, I am doing home remedies at home right now, its cure, cold, like medicine, it increases, so I do it myself at home.
R9- We would have got it from here too, as cough is a pain and so on.
R8- We treats you at home and do not go to the doctor
R5- Those who are behind Agarwal, the doctor of children
R3- Cold is hot or cold etc. It is treated at home

1.1. Which ones are usually accessed for non-critical illnesses and why?
1.2. Which ones are usually accessed for critical or severe illnesses and why?

R8- Heard no
R5- Our two boys are also ours
R6- No sir there is no such child
R4-Sir was the son of my sister-in-law, the sister-in-law also had jaundice, someone also told them not to give their mother's milk, someone started saying that cow's goats started feeding milk, so their body did not get stains like stains. There was such a shortage of water…. As the doctors refused… they had kept the admissions for two months, kept them in the machine…. So they scolded that even if mother No problem, feed the mother's milk only by asking the doctor ..... You should not feed outside

<Have you ever heard of a child being admitted in hospital> R6- Have heard
<what have you heard>
R5- Do not know, but have heard that his child had become serious, he took him to the hospital, but he was heard far and wide but he was not even close to his nearest....
<in which hospital were you admitted>
R8- Now as we belong to Aligarh, Aligarh has only one name, Bansal is a hospital in the name of Agarwal, most of the children are treated in it, so they take everyone there

<have you also ever heard that Child was taken to hospital and there only he died>
R9- No it's nothing
R10- Yes, I have heard that I have heard in Aligarh, Patan was not his daughter, he had fallen in the head, he had come here to Delhi to get treatment, to no avail, his child was killed.
R2- Our neighborhood is not far away. There was a little boy of our aunt, who brought him here, drove medicine from here in S Hospital, still did not recover, did not recover, he had blood cancer, he was dead for 4 or 5 years. Went his
R5- One I have just heard, heard in Tughlakabad, that the child had a common cancer.
he was a child for Rs 6-7 lakh but he was not live.

Pregnancy, outcome and care seeking practices

You are aware that several of the pregnancies in the area/community terminate with stillbirth (death during delivery) or intrauterine death (death inside womb).

2. **Where do people from your community/ locality usually go or whom do you consult during pregnancy and delivery?**

2.1. Which ones are usually accessed for routine pregnancy check-ups?
R5 - S Hospital

R3 – MN Hospital
R2 – M Hospital
R1 – S Hospital
R8 - M Hospital
R7 – B Hospital
R6 - MN Hospital
R4 - MN Hospital
<why did you choose that place>
R5- They says it right, that's why they chose checks are right there and right there
<at S Hospital>
R6- There, they do it very freely, Sir, MN Hospital is very good… it is close by… and it is not far away… as everyone says that the government does not take proper care. .
<How many times had you gone>
R7- MN Hospital was to be visited month to month ..

1.1. Which ones are usually accessed for delivery or pregnancy with complications?

<was delivery done at same place>
R3- No, in MN Hospital, Same is provided from place to place

Causes of death and knowing the cause

3. In your view, what are the common illnesses and causes of child death in your locality?

<India is no. 1 in Case of Child deaths>
R4- Yes, India is No 1, in all this, I have heard in T.V.
<have you heard Anything>
R2- Yes, as you are not able to go, death is done on the way, people have not been able to get treatment on time or the doctor has some relaxation.
<have you heard anything>
R7 - There is negligence of some common public, negligence of some hospitals.. Because of time of all people ... Doctors also write that complete this formality, then you will not have to admit before admitting us, sometimes even the amount in some parents. There is a lack of people who cannot afford it… There are many other reasons…
<so the amount you are talking about, is it in government hospital or private hospital>
R10- It happens in private and the government's fame is so much that people will get upset while fulfilling them, because sometimes the female has no problem, they will not let the mail be taken away. .... Now it happens that there is no chance of many children, so there is a problem in admitting them, so what will happen to them ..... Means the mother was not there and after that the child was not even admitted. What has to happen from time to time, whatever wrong thing has to happen to him….
<cause of death unknown>
Dr., if it becomes a death, it is also the cause of the child that explains the reason, it is like this, sometimes the symptoms are seen according to the symptoms that are seen…. Just like you have said that you had a child of cancer, whom you told… .But many children who are, have coughs or toast …… the reason for not knowing the inside
R4- Because they did not treatment
<yes treatment and of things whatever>
R5- Sir, I do not know what happened.

4. In your view, what are the common causes of stillbirth and intrauterine death in your locality?

<have you ever heard of things like people who are pregnant do not give birth to a normal child, either baby is born prematurely, or they are not able to breathe>
R5 - yes
<of which females have you heard>

R1- Sir, when I was an Admit in S Hospital, I had heard that there was a child of 4 months, but she lived like this, she was dead 4-5 days after that….

<so, the cause must be kown>

R1- No, just knew that it was kept in ICU

R5- Yes have heard This happened to our elder daughter, it was seven months old, what was the gas in her stomach, or what gas was made in her stomach ... How did the stomach end up in her stomach but it was heard ... So the child was chopped off… the child was probably taken out…

<so, did you got to know the reason of death>

R4- No not detected

<how can we know the cause of stillbirth>

R2- Don't know this

<How can we know the cause of death>

R5- According to me, you should tell the doctors… Doctors should tell you in advance that this problem, this is this

We are trying to identify the causes of death in children and stillbirths, so that something can be done to prevent and reduce the number of deaths in children and stillbirths.

5. In your view, would this information be valuable?

(Probe: If valuable/ useful, why? If not, why not?)

<information provided is valuable or not>

R5- Why don't we know the reason, then we can

R3- Because yes, you know, what was the reason, brother, there was a lack in us, because of which it happened… or what we did careless in what happened to our child…. It is important to know or not so that we can take care of it for further .....

<Are people willing to know the cause of death>

R3- Just like everyone, why does the child become like this…

R7- If we did not know about the disease, and then the doctor would have told us earlier that we would not be able to cure, and then we would have gone elsewhere.

R4- If something happens to our son, you will ask, right, what was the disease… If you could not fix it, then tell us, we would have gone somewhere else….

<how many people want to know the cause of death>

R3- Everyone thinks

R2- Everyone should know

R4-90% think like this, they think….

In some cases the children die before even the exact cause of illness and death is identified/ investigated. In such cases, doctors suggest autopsy/ post-mortem to identify the cause(s) of death. Similarly for the stillbirths and intrauterine deaths, *doctors suggest autopsy/post-mortem of the baby to identify the cause(s) of death.*

6. How autopsy/post-mortem is viewed by you and community members for knowing the cause of death?

(Probe: views, concerns and believes in the community regarding autopsy)

R1- Only doctors will be able to tell you this… like ultrasounds are like they should do .. CT scans are like they are of the whole body… they should do that too, there would be sickness coming out of it. is....

<everything has separate procedure to know the cause>

R2- Everything has its own way… it is

<how can we know the internal cause>

R7- As a matter of fact, a normal human being should have to do a check every six months .... something that should be done without an important check etc. You cannot get a sari done ... this way you know something It goes on ....

Suddenly, it is not a matter for him, just how it happened, it can be known only later that his test and so on…. ... if he has a problem in the waist, then it cannot be the gift of the stomach.
R1- Just like there is a reason in the teeth ... it hurts to feel a tooth pain
<How is autopsy viewed By you, for knowing the cause of death>
R3- It is heard after death, post mortem or not later, mostly accident, etc.
R5- Among the sudden deaths
<so, do we get to know the cause of death from it>
R7- Where there is a stove, there is a stove
Dr. - But suppose a woman or a man dies suddenly, does not have an accident….
R7- If you do not know the reason, you should do it .... They are also on top of who they are, whether they want to get it done or not .... It depends on them ... because there are many things, everyone Don't want .... because when there is a postmortem, I take away those things that are in me .... eyes etc ... and in accident there are postmortems which are those whose hearts, etc., they don't stop soon. Accident's people are used to someone else's work, that's why they keep it, people .... The rest Normally, when someone will not speak, no postmortem can be done before that.
<acceptance, views for post -mortem in the community>
R5- Are not
<what is the cause of not accepting>
R7-- They say, rip it apart and put it away
<why people refuse for postmortem>

R4- Because their whole, whatever is inside is taken out ... All the different cases are in which they think that something will be known or some charges will come, etc. They do not even allow it to be done. ...
In some instances, autopsy/ post-mortem is not possible due to various reasons. To find out the exact cause of death and underlying disease, some tissue biopsy (collection of very small sample using needles) and fluids (blood, urine, etc.) from the body of the child after they die, as done for investigation. But these samples are to be collected as soon as possible after death.

7. **How do you view minimal tissue sampling procedure as a method for knowing the cause of death?(Probe: views, concerns and believes, religious aspects, comparison with autopsy)**
<what is view of minimal tissue sampling>
R4- Yes, it is heard… this is fine… no tearing will not happen… stomach will not tear…

8. **How the parents/families of a child/newborn who has died, should be approached by the hospital doctors/staffs for the minimal tissue sampling procedure?**
(Probe: who, when and how and whom should approach, who can be key influencer)
<how parents should be approached for minimal tissue sampling>
R6- You will have to explain to the family, first they will understand.
<whom should be approached>
R7- It is also necessary to be proper beforehand. It is not that at which time we have a problem, think at the same time ... It is also very important for anything to be pre-mature .... How is pneumonia caused? To the child… As far as the treatment of pneumonia is concerned, there is nothing that the death of pneumonia will be done directly by the child… There is a treatment of pneumonia, you should treat it, nor can we show the child's gift anywhere else. Because there is no disease that is untreated This is said to be the place where there is no cure for sickness… we cannot treat a child like that as far as we are trying but there is no cure for it… like cancer. Is there no such treatment for cancer, yet the rest of America is inside Delhi, it has been treated successfully… Now this is the reason why these problems cannot be left which is pneumonia, fever, so There are big diseases .... there is no cure for them For this, the doctor said that we cannot save the child… treatment is to be continued or not….
<when it should be told>

R4- No, be told in advance that the problem can happen, you have to be ready for the future, meaning… this problem can also move forward… only then will you have to tell… how will you tell at the end time .. On end time, toh parents will also get upset, meaning what to do now… meaning the last stage has come, what can parents do at that time…. Tell me in advance that the doctors know that This means that where the position can reach the stove… where it cannot reach the stitch…
R6- What is more important to tell in advance. Along with this, prepare a little bit so that they understand so that together they do not get shocked, it means ...
R8- According to me, you will agree more ...
R5- If they have told the family members, but the mother of the child is… she can tell it only by looking at the situation… because some get it bad, some cannot do it… how to direct Will tell you that your child is born dead or has a problem, cannot tell it… because it depends on them how the mother's condition is….
R4- It is a matter of whether you have to tell it at some time… then you cannot tell it at all…
Only the doctor will tell you the reason, for what reason it happened….

9. **In your view, what factors are important for acceptance of the minimal tissue sampling by the parents and family members?**
   (Probe: desire to know the cause, trust, confidence on the health care provider, who can be key influencer)

Now people tell me what to do
R4- So if such a problem happens then one must also get it done Dr. - tell them, can you agree
R5- I had heard, this is the case in front of me .... Once the lady was pregnant, when the doctor got the ultrasound etc., which was the child's retard, she was not progressing, the doctor who was with me, my check up She was doing .... So the doctor said ... Do not speak to the lady, tell the husband that your child is retarded, he is not progressing, so I cannot tell you to get abortion done right now from us. Even the big doctors are sitting, writing for AIIMS, for the sake of S Hospital who are bigger doctors than us.

Focus Group Discussion-Mothers 1

Number of participants: 10

1. **Where do people from your community/ locality usually go or whom do you consult during illness of children and newborn?**

R1-Most of the time they go to ASHA dispensary, is government, yet it shows, yet in M, there is a dispensary open there too, nowadays everyone is well cared for, everyone knows, so see takes
R2- I felt right dispensary from M. Is for free.
R3- Go to M, ladies and sister all.
R4- They spend money there, they take more money for everything, there it is for free, what you are saying
R2- free
R1- In the ASHA dispensary, there is a lot everywhere.
R4-in M all free
R2-free at all
R10-little bit for everywhere
R4- There is benefit
R3-There is benefit on there, then there is benefit on there, they check there well, injection for a long time is better, they also understand to drink food, they understand everything well, their doctors come and understand at home. Doctors send from AIIMS, they also come home
R1- She also comes to the children for vaccination, she puts it as well.
R10- Private or S Hospital. Then, most of the time they get the delivery done, it is government, which is suitable,
R4- S Hospital is government Dr SG is in private
R3-government
R4- Shashi Goel is private, who has the facility, people go there
R1-depends on budget

1.1. Where people go during pregnancy/Delivery

R 1 &3- S Hospital
R2- S Hospital
R4- Yes same goes all
R4-someone goes to Dr SG
R2- in here EL is also private
R1-one or two private
R3- Just like there is a serious case, like a normal everyone has their own budget, what is there, who does not have it, poor people only go to S Hospital.

1.2. Where people go during child illness

R2- Go there for them
R5-go to M
R1-in M Dr. R
R5- Nobody sees a little child here, much
R5- Yes ASHA comes, he goes to M asha dispensary, and he goes to the same
R1- No, I take him to M where is a Doctor R.
R4- Children's medicines get good at the same place.
R2- Dr M is a ladies doctor, she also give good medicine
R1- There is a number one. In the same CPI
R2- in down
R6- Have gone to Doctor S
R3- Doctor S

2. **In your view, what are the common illnesses and causes of child death in your locality?**

R 1- (laughing) this is
R4- It is that they also look after themselves, what they do it happens
R2- It happens
R4- Suddenly, children do not even clean their hands, take them into the mouth, while the germs go into the mouth.
R1- Like when the time will be in winter or in the winter time it will definitely happen
R4- Now we care very well, yet it happens
R1- He puts his hand in his mouth for 24 hours, so it must be for this (laughing).
R5- There is not much cleaning
R3- I was gonna say the same thing
R5- This is mostly it, that's why middle children ill
R4-too dirty
R3- There is a lot of dirt, there is no park for children, and there is nothing to play around.
R4- Everywhere is dirty
R5- There is no sunlight anywhere. Small children mostly need sun in winter. He is not.
R2- So he is not
R4- Nothing is there
R5- And then the parent, the mother who works, feeds her milk, then the child ill. Because the baby will feel cold when the mother gets cold

1. What home remedy Parents give in child illness

R1- One has to take care of eating and drinking as the doctor says
R5- It is important to keep the child clean, with the help of cleanliness of the child, giving medicines, washing hands with dettol.
R2- I am massage, with hot oil, I do what the doctor tells me. I am massage at home.
R4- Vicks, just a little care
R3- In diarrhea, ORS solution
R2-ORS
R6- Most people do what doctors say
R3- gives boil water
R4- boil water
R3- In cough and cold will give cough syrup or keep warm water in bloom, whether the child is well or not, they do it.
R4- Doctors give drop in fever, they give it there
R6- tells us massage; keep a bandage of cold water.
R4- Yes, cold water if it is temperature high.
R6- My child had a fever, so the doctor said, strip it naked, leave it at all, and keep a cold water strip. Then massage very well. When checked, its fever subsided
R1- Don't covers much

2. When child is critically ill

R3- going to hospital
R5- going to S Hospital
R4- mostly going to S Hospital
R6- MB or S Hospital
R1- refer to S Hospital
R4- going to S Hospital
R1- I have also gone because once it got very bad at night, it was sent to S Hospital directly from MB.
R7- didn’t go to doctor
R5- It has just come to live. It came from outside

3. How is the treatment given to child

R8- Children get rest, we are being taken at night at every heap, injecting them, giving them some medicine, say they will eat morning, then maybe the child will get rest.

8. Child died due to illness

R5- Yes, it has happened yesterday, it has happened in S Hospital

R1- Whose

R5- pneumonia fever

R1- Disease is dangerous

R5- Would not has noticed beforehand, if the child is small, he cannot speak, if he sees any problem, and then should be shown immediately. Whether it is shown in government or whether it is shown in private. Cleanliness is more important than caring for a child.

R5- The condition worsens, it does not have the strength to withstand disease

R1- Most of the time this happens when the child is a little ill, then she is careless. Later, when the child's condition worsens, he pays attention. It will be fine if you pay attention in advance

R6- The fever subsides, if it starts playing, we think it is okay

R8- Taking medicines from a small doctor, pill was taken, healed. Did not show the little doctor that beforehand show it a little, it will be corrected?

R1- Well here most of the doctors are like this, they sit here, untrained doctor

R8- They do not know what medicines are being given, the doctors here are useless.

R1- A doctor had given an injection that one of the ladies had died, so no one should show up here quickly.

We are trying to identify the causes of death in children and stillbirths, so that something can be done to prevent and reduce the number of deaths in children and stillbirths.

3. In your view, would this information be valuable?

R8- No nothing no one knows

R5- If you blame the doctor, then you will blame the doctor, if treatment is going on with the doctor for 4-5 days, then you will blame the doctor

R4- Doctors tell you that this disease has happened, it has become dengue, it has happened, it has happened, it has taken the name of the disease, now it is not known what caused it to end, no one knows what That's why it's over

Desire to know the cause

R4- We all wish that yes, we do not know why it is over, we will also know

R6- Yes i want to know

R3- What kind of mistake did we make that we lost our child with our hands? Everyone belongs to the mother, no one wants to lose their child a little. Mother is there

R4-There is equality in everyone's heart, that if we know what has ended, what has happened. No matter who the child is, it ends up hurting everyone, that yes, his child ended up like that, he had that disease. But that disease is not known whether it was a disease or not.

If child was ill or not that caused death

R4-No

R1- No, because now brother will not know at any time whether he had any disease or not, because he is tired enough to see if someone else does a test and see, it happens in him.

R6- No sister, still there is doubt in the mind.

R6-See this, our child speaks something, show it to a doctor, he will say it has a lot of cold, some will say fever, some tell something, some kind of doctor change it, it starts talking about 3-4 ways. They are telling something, they are telling something. What has happened in our mind also that it was not there, it was not this thing, yet it starts coming out. Now my son had dengue, I showed him something in Moti Bagh, he told something else, then she was taken to Chhatarpur, where she told that it is typhoid. The doctors told a lot of things, yet ... got it done

R4- This is wherever you take the child, to different doctors, he will tell different things. Will never tell you one thing. Till today it has not been known that they are telling the truth, what is this and what is not, it has not been found till date.
R5- Yes, of course, we have it here.
R4- I hear so much
R1- No brother, God does not do this, it is heard that someone's child is already killed already
R6- eight in a half months when he was born.
R4- This is also not known, it is not known whether it will be done in the first month, see how much treatment will be done, yes all is right, everything is all right. After being born, the child ends up in the stomach, and even after coming out.
R6- Not going on in S Hospital, treatment is going on in A Hospital too, of thyroid
R4- They are being treated
R6- It was only for me that, after 6 months, she was fine when she was born, it was over. As soon as born She was in sing
R6- There was nothing like it but immediately, I do not know why it was bad, the whole time was also gone, it was over. The doctor did not tell anything because the girl was at home. Yes (desire to know)
R4- Everyone has heard who will tell
R1- In hearing it comes from somewhere, it has happened to the people, brother,

4. In your view, what are the common causes of stillbirth and intrauterine death in your locality?
R5- Most of all this happens due to delivery at home, mostly
R2- I don't like anything at home. This girl was in my stomach the whole time I went to the dispensary with Munirka, Madam said that the child has died in the stomach, wrote to me that I will read about the operation. And also gave me a date after 3 days, then after 3 days Ajna, we will take you to the hospital, my girl has become very well at home. And I went mad crying for 3 days that my baby has died in the stomach, she is roaming well, she is roaming there too
R4- What a wrong thing.
R2- That's why I feel right at home, at home. So I never go to that dispensary. Do not like anyone, I do not go. ...... I used to live there, there is such a good dispensary, people will not find medicines of the private here, but they are engaged there.
R1- It is a matter of putting it.
R6- Apart from my girl Arya Samaj does not seem to have any medicines, got so much medicine for cold, got pneumonia in the middle, did not take any medicine at all, took Arya Samaj.
R6- It was okay for the whole time, the girl was also there in the house all the time, but it ended suddenly.

5. What are the rituals and practices observed by the family and community when a child dies?
Parents reaction on child death/stillbirth
R1- It would feel very bad that brother is the first child, if this happens, be it first or second, it looks bad at first, and whose first one he will feel even more.
Rituals
R6- The child, we buried him, was very small
R6- The same child finishes, yet the rules are taught to follow the same child. To feed the person who is the mother, who feeds the mother, the child only, feeds them, the registrar reads the feeding.
R1- Seeing is reading.
Difference in rituals in stillbirth and child death
R4- No no that is
R5- No, as long as they are not married, they have children, they bury them
R9- And don't burn
R6- Bury them and then feed them a little bit
R9- We don't do anything like this (asked rituals are same like adult death ritual)
R5- Don't do anything like that, don't do anything like that.

R8- We do not do here R6- (asked if stillbirth or child, rituals are same)
Yes little baby

6. How does the parents and family members cope with the death of their child?

Coping of mother

R4- Some even take 4–5 months, some even in 1–1.5 months, some get it right only after one and a quarter of a month. Now it is a matter of the mind.
R4- It takes a month and a half.
R1- It seems to me that the person whose child will die is the only thing in his life, it is a matter of maintaining that there is a lot of work to be done and also food to be eaten, a person has to study, and the rest is shock It never ends with the heart, that too of God, do it to God or not to anyone.
R6- Mother is unable to emerge.
R3- Matter of life
R4- Never ending heart
R6-child or daughter, anyone
R6- You are sitting here, look at them, their child is young, this is their mother, they have not forgotten till date, no one can forget.
R1- It is shock, cannot be forgotten, nor does it ever come out of the mind. Just leave it, let's work, we also have food, everything to live.
R4- The shock is the same, it remains in memory, as many as the Teej festivals come on that day they remember it every time.
R3- His birthday comes misses everything.

Coping of Father

R3- Men are like
R1- Men get set on their own
R4- There must be pain, why it does not happen, everyone hurts, be it father or son, everyone hurts.
R8- Everyone hurts
R4- Now, whoever he tells, how should he tell, a little too much has told himself, otherwise he has gone a bit if he has gone on time duty If a little too much remains in it, then it is right after coming home
R4- The mother stops everyone, stops all the anger, removes all the anger, still remembers child, child or daughter.

Parents go to work after child death

R5- Someone leaves immediately, only after two months
R2- It takes a gap of two months
R8- Work will be missed in a couple of months.
R6- The body is not there at that time, it is not able to work, it is avoided (stillbirth)
R6- When they go away after one and a half weeks (in case of child death), they do not give leave for so many days or any work
R3- Who gives so many days off at work
R6- Does not work, does not understand madam
R6- All go away (papa), those people leave early
R5- It does not happen here. I have to study to work
R4- If there is work, everything is there, whenever people are eating and drinking so much food, there is nothing if there is no work. Everyone is eating here after earning
R5- Everyone does not understand, no problem, no problem

Who Support in coping up?

R4- All the street mohalla people would be Sir
R4- Everyone does it as if someone has passed away, anyone, drinking food, tea and water, all the people of the neighborhood do it together.

Support from family in coping

R6- Everyone comes

R5- Now here the house is also small, if you are staying away, then you do not stay long.
R4-4-4 for 5-5 days, like 4 days stay, 3 days stay, everyone has their work
R2- We stop, month week, 15 days we stop
R5- If someone is nearby, they can see, those who are nearby, they do care, no matter what the mother is.

We are trying to identify the causes of death in children and stillbirths, so that something can be done to prevent and reduce the number of deaths in children and stillbirths.
7. In your view, would this information be valuable?
(everyone) I don't know
R3- We ladies do not know anything, mostly our gents go to all

In some cases the children die before even the exact cause of illness and death is identified/ investigated. In such cases, doctors suggest autopsy/ post-mortem to identify the cause(s) of death. Similarly for the stillbirths and intrauterine deaths*, doctors suggest autopsy/post-mortem of the baby to identify the cause(s) of death.*
8. How autopsy/post-mortem is viewed by you and community members for knowing the cause of death?
R2, R-3, R-4, R-1 yes (3-4 ladies)
R1- yes heard
R3- yes heard
R1- Cut off humans
R4- Take out all the things
R3- Take out all the stuff and fill it
R2- Like took out a kidney, got a heart, took out the eyes
R3- Take out the lungs, take out the eyes, to test it
R4- Close it and empty it and send it
R2- That is, many times, take out the kidney, take out the eyes, take out the heart
R5- Let's try to find out what was the problem in the post mortem, it is mostly, as if someone has got an accident or someone has suffered some injury, etc., if he died, then what has happened, the vein has ruptured. Or someone was hit deep, or there was too much bleeding in the extremities
R1- Due to some illness
R5- Or were ill, that's why we do post mortem.
R5- Information or address someone has to
R3-No, still my husband's friend's daughter of Behen has been born in S Hospital, when she was kept there for 3 days and 4 days, she was told to check up the child, also check up blood, everything was normal. Now that girl's health deteriorated again, the doctors told her a hole in her heart. Then tell whom to trust

In some instances, autopsy/ post-mortem is not possible due to various reasons. To find out the exact cause of death and underlying disease, some tissue biopsy (collection of very small sample using needles) and fluids (blood, urine, etc.) from the body of the child after they die, as done for investigation. But these samples are to be collected as soon as possible after death.
9. How do you view minimal tissue sampling procedure as a method for knowing the cause of death?(
No (all respondants about biopsy)
R1- Yes heard it (Rasoli)
R2- Only doctors can know this
R4- Very good thinking, in this way some disease will also be known or not immediately, brother, it is a disease, then it will be treated quickly.
R3- Yes, no one would mind
R2- Like someone got TB, got cancer, about these things,

R1- This is why humans, now people do not want to get post mortem, now it is good for them, it will be known that our humans will be stabbed
R4- Too good too much too
R1- It will be good that even after we die, its body is not there, all of that happened.
R4-nothing happen
R3- And the disease was also detected
R1- It seems bad, brother removes everything, then what does Naahik send to him, then that is also not good.
R3- It is a good thing, which does not divide half of our human beings in four parts, it is good,
R1- Now mine, I tell you, one of my brothers was killed here, the boys were not even twenty years old.
R8- Have been with them
R1- He was married, one month was left, 14 to 14 years old, then his friends took him and killed him. So now one killed him, we are very sad that brother killed him. The post mortem from above. I can't even see my mother. But it was spoiled with him and bitten by it. Either we would have given such a thing. Even if it was okay, it does not mean unhappiness. This is just nothing else.
R1- That would be fine, however, that there was not a lot of biting with him, much better.
R4- It has told itself that it is very good

10. How the parents/families of a child/newborn who has died, should be approached by the hospital doctors/staffs for the minimal tissue sampling procedure?
R6- First of all, we tell a man, we cannot tell women
R1- no one tells gents
R6- Now there is trouble
R4-As we know it is more serious, its health is worse, if we tell them more, then it may be too soon, and it is too much that it is not new, it has not happened, it has happened Two, let it be. This is also a mind for everyone.
When should be told?
R4- It should be told in advance, as the health is a little worse, the check up is going on, it should be told that yes, the doctor said, do something to understand this
R1- Should be told in advance so that this does not happen to the other child.
R2- Should accompany him (during death declaration)
Who should be told?
R2- Those who are at home should tell
R1-Most of the time, what has increased in the house, tell one, tell him that yes, it is a little sensible, it will get better, let's take care of it, it should be told to the elder. Meaning in the house that is older than us, like anyone in the house, brother, father, mother or mother
R5- Mostly in the married house there is husband, or mother.
R1- New, there is definitely one or the other, if the health is bad, then there is definitely someone at home.
Who should tell about MITS?
R4- In this, only doctors, doctors can tell, yes it is not so, only doctors can tell it.

11. In your view, what factors are important for acceptance of the minimal tissue sampling by the parents and family members?
R4- Whatever is inside it, even in the child, even in the eldest, this disease is anything, so the doctor should tell that there is little to be left in it, to do this with it.
R1- First, by telling them, they will be able to handle a little further, then they will not be a problem.
R3- Will take care. This is it
R4- No one knows at home
R4- My sister is just a year old, Didi is over, she had a disease of sugar, in sugar, she got her doctor checked, she told me typhoid, she told dengue in typhoid. So he died in that. (Death) in the hospital.

R6- Someone talks, someone asks, then when they talk about what happened, how what, we did not know, they have slept well at night, they have eaten food, like this , tell like this is.

12. In your view, what would be the most suitable method(s) of educating the community about the minimal tissue sampling?

R4- This is a very good thing, then everyone will know or not, everyone will know that yes, it should not be done like this, some solution will have to be found out about it.

R2- I don't know

R2- You tell me that it is right, only the doctor can tell. Only doctors can tell information.

R2- What else can doctors tell

R4- Don't tell me like this again, just give the medicine to tell, just take this medicine at this time and take this medicine at this time. Do not move beyond that, take something even further. This date is for you to try this check up. Will give one tablet to another, medicine will remain the same, yes just try again. 3 times we call for a check up, just after that we say that it is done.

R8- If you ask more then let's say yes it's okay now

R4- You are fine now, you are fine

13. In your view, how the community leaders/influencers like you can contribute/ participate in such activity?

R6- All will do

R5- The one with ASHA clinic, yes they can tell

R4- Now as we too have become, we have also come to know a little bit, we can also tell that it is not like that, it has happened, and we can also take some convenience to do a little something good

R4-(Thinking) I say, whatever is good, it means for children as well, for growing, and also for the elderly, whatever is good, my thinking is the same. There is more than just a little bit for children. About caution, in cleanliness, means something like this, about it. If it is clean, diseases will also be reduced. And where there is no cleanliness, there will be diseases. No matter how much we keep the child clean, there will be diseases

R4- I do this to my child that when there is a lot, whatever happens, I will show him first.

Focus Group Discussion- Mothers 2

Number of participants: 9

Illnesses, death and care seeking practices

You are aware that several of the children in the area/ community fall ill/become sick and some of them die.

1. **Where do people from your community/ locality usually go or whom do you consult during illness of children and newborn?**

R6- I go to doctors here. I showed all the doctors there was no difference But there I feel comfortable, so I go there and go nowhere

R4- Our doctor is there in Khanpur

R7- Sir, I am doing home remedies at home right now, its cure, cold, like medicine, it increases, so I do it myself at home.

R9- We would have got it from here too, as cough is a pain and so on.

R8- We treats you at home and do not go to the doctor

R5- Those who are behind Agarwal, the doctor of children

R3- Cold is hot or cold etc. It is treated at home

1.1. Which ones are usually accessed for non-critical illnesses and why?

1.2. Which ones are usually accessed for critical or severe illnesses and why?

R8- Heard no

R5- Our two boys are also ours

R6- No sir there is no such child

R4-Sir was the son of my sister-in-law, the sister-in-law also had jaundice, someone also told them not to give their mother's milk, someone started saying that cow's goats started feeding milk, so their body did not get stains like stains. There was such a shortage of water…. As the doctors refused… they had kept the admissions for two months, kept them in the machine…. So they scolded that even if mother No problem, feed the mother's milk only by asking the doctor ..... You should not feed outside

<Have you ever heard of a child being admitted in hospital> R6- Have heard

<what have you heard>

R5- Do not know, but have heard that his child had become serious, he took him to the hospital, but he was heard far and wide but he was not even close to his nearest....

<in which hospital were you admitted>

R8- Now as we belong to Aligarh, Aligarh has only one name, Bansal is a hospital in the name of Agarwal, most of the children are treated in it, so they take everyone there

<have you also ever heard that Child was taken to hospital and there only he died>

R9- No it's nothing

R1- Yes, I have heard that I have heard in Aligarh, Patan was not his daughter, he had fallen in the head, he had come here to Delhi to get treatment, to no avail, his child was killed.

R2- Our neighborhood is not far away. There was a little boy of our aunt, who brought him here, drove medicine from here in S Hospital, still did not recover, did not recover, he had blood cancer, he was dead for 4 or 5 years. Went his

R5- One I have just heard, heard in Tughlakabad, that the child had a common cancer.

Pregnancy, outcome and care seeking practices

2. **Where do people from your community/ locality usually go or whom do you consult during pregnancy and delivery?**

2.1. Which ones are usually accessed for routine pregnancy check-ups?

R5 – S Hospital

R3 - MN Hospital

R2 - M Hospital

R1 – S Hospital

R8 - M Hospital
R7 – B Hospital
R6 – MN Hospital
R4 - MN Hospital
<why did you choose that place>
R5- They says it right, that's why they chose checks are right there and right there
<at S Hospital>
R6- There, they do it very freely, Sir, MN Hospital is very good… it is close by… and it is not far away… as everyone says that the government does not take proper care. .
<How many times had you gone>
R7- MN Hospital was visited month to month .. Every month every week.

2.2. Which ones are usually accessed for delivery or pregnancy with complications?
<was delivery done at same place>
R3- No, in MN Hospital, Same is provided from place to place

3. In your view, what are the common illnesses and causes of child death in your locality?
R4- Yes, India is No 1, in all this, I have heard in T.V.
R2- Yes, as you are not able to go, death is done on the way, people have not been able to get treatment on time or the doctor has some relaxation.
R7 - There is negligence of some common public, negligence of some hospitals. Because of time of all people ... Doctors also write that complete this formality, then you will not have to admit before admitting us, sometimes even the amount in some parents. There is a lack of people who cannot afford it… There are many other reasons…
<so the amount you are talking about, is it in government hospital or private hospital>
R4- It happens in private and the government's fame is so much that people will get upset while fulfilling them, because sometimes the female has no problem, they will not let the mail be taken away. .... Now it happens that there is no chance of many children, so there is a problem in admitting them, so what will happen to them ..... Means the mother was not there and after that the child was not even admitted. What has to happen from time to time, whatever wrong thing has to happen to him….
<cause of death unknown>
Dr., if it becomes a death, it is also the cause of the child that explains the reason, it is like this, sometimes the symptoms are seen according to the symptoms that are seen…. Just like you have said that you had a child of cancer, whom you told… .But many children who are, have coughs or toast …… the reason for not knowing the inside
R4- Because they did not treatment
<yes treatment and of things whatever>
R5- Sir, I do not know what happened.

4. In your view, what are the common causes of stillbirth and intrauterine death in your locality?
<have you ever heard of things like people who are pregnant do not give birth to a normal child, either baby is born prematurely, or they are not able to breathe>
R5 - yes
<of which females have you heard>
R1- Sir, when I was an Admit in S Hospital, I had heard that there was a child of 4 months, but she lived like this, she was dead 4-5 days after that….
<so, the cause must be kown>
R1- No, just knew that it was kept in ICU
R5- Yes have heard This happened to our elder daughter, it was seven months old, what was the gas in her stomach, or what gas was made in her stomach ... How did the stomach end up in her stomach but it was heard ... So the child was chopped off… the child was probably taken out…

<so, did you got to know the reason of death>
R4- No not detected
<how can we know the cause of stillbirth>
R2- Don't know this
<How can we know the cause of death>
R5- According to me, you should tell the doctors… Doctors should tell you in advance that this problem, this is this

We are trying to identify the causes of death in children and stillbirths, so that something can be done to prevent and reduce the number of deaths in children and stillbirths.

5. In your view, would this information be valuable?
(Probe: If valuable/ useful, why? If not, why not?)
<information provided is valuable or not>
R5- Why don't we know the reason, then we can
R3- Because yes, you know, what was the reason, brother, there was a lack in us, because of which it happened… or what we did careless in what happened to our child…. It is important to know or not so that we can take care of it for further .....
<Are people willing to know the cause of death>
R3- Just like everyone, why does the child become like this…
R7- If we did not know about the disease, and then the doctor would have told us earlier that we would not be able to cure, and then we would have gone elsewhere.
R4- If something happens to our son, you will ask, right, what was the disease… If you could not fix it, then tell us, we would have gone somewhere else….
<how many people want to know the cause of death>
R3- Everyone thinks
R2- Everyone should know
R4-90% think like this, they think….

In some cases the children die before even the exact cause of illness and death is identified/ investigated. In such cases, doctors suggest autopsy/ post-mortem to identify the cause(s) of death. Similarly for the stillbirths and intrauterine deaths, *doctors suggest autopsy/post-mortem of the baby to identify the cause(s) of death.*

6. How autopsy/post-mortem is viewed by you and community members for knowing the cause of death?
(Probe: views, concerns and believes in the community regarding autopsy)
R1- Only doctors will be able to tell you this… like ultrasounds are like they should do .. CT scans are like they are of the whole body… they should do that too, there would be sickness coming out of it. is....
<everything has separate procedure to know the cause>
R2- Everything has its own way… it is
<how can we know the internal cause>
R7- As a matter of fact, a normal human being should have to do a check every six months .... something that should be done without an important check etc. You cannot get a sari done ... this way you know something It goes on ....
Suddenly, it is not a matter for him, just how it happened, it can be known only later that his test and so on…. ... if he has a problem in the waist, then it cannot be the gift of the stomach.
R1- Just like there is a reason in the teeth ... it hurts to feel a tooth pain
<How is autopsy viewed By you, for knowing the cause of death>
R3- It is heard after death, post mortem or not later, mostly accident, etc.
R5- Among the sudden deaths
<so, do we get to know the cause of death from it>
R7- Where there is a stove, there is a stove

Dr. - But suppose a woman or a man dies suddenly, does not have an accident….
R7- If you do not know the reason, you should do it .... They are also on top of who they are, whether they want to get it done or not .... It depends on them ... because there are many things, everyone Don't want .... because when there is a postmortem, I take away those things that are in me .... eyes etc ... and in accident there are postmortems which are those whose hearts, etc., they don't stop soon. Accident's people are used to someone else's work, that's why they keep it, people .... The rest Normally, when someone will not speak, no postmortem can be done before that.

<acceptance, views for post -mortem in the community>

R5- Are not

<what is the cause of not accepting>

R7-- They say, rip it apart and put it away

<why people refuse for postmortem>

R4- Because their whole, whatever is inside is taken out ... All the different cases are in which they think that something will be known or some charges will come, etc. They do not even allow it to be done. ...

In some instances, autopsy/ post-mortem is not possible due to various reasons. To find out the exact cause of death and underlying disease, some tissue biopsy (collection of very small sample using needles) and fluids (blood, urine, etc.) from the body of the child after they die, as done for investigation. But these samples are to be collected as soon as possible after death.

7. **How do you view minimal tissue sampling procedure as a method for knowing the cause of death?(Probe: views, concerns and believes, religious aspects, comparison with autopsy)**
   <what is view of minimal tissue sampling>

R4- Yes, it is heard… this is fine… no tearing will not happen… stomach will not tear…

8. **How the parents/families of a child/newborn who has died, should be approached by the hospital doctors/staffs for the minimal tissue sampling procedure?**
   (Probe: who, when and how and whom should approach, who can be key influencer)
   <how parents should be approached for minimal tissue sampling>

R6- You will have to explain to the family, first they will understand.

<whom should be approached>

R7- It is also necessary to be proper beforehand. It is not that at which time we have a problem, think at the same time ... It is also very important for anything to be pre-mature .... How is pneumonia caused? To the child… As far as the treatment of pneumonia is concerned, there is nothing that the death of pneumonia will be done directly by the child… There is a treatment of pneumonia, you should treat it, nor can we show the child's gift anywhere else. Because there is no disease that is untreated This is said to be the place where there is no cure for sickness… we cannot treat a child like that as far as we are trying but there is no cure for it… like cancer. Is there no such treatment for cancer, yet the rest of America is inside Delhi, it has been treated successfully… Now this is the reason why these problems cannot be left which is pneumonia, fever, so There are big diseases .... there is no cure for them For this, the doctor said that we cannot save the child… treatment is to be continued or not….

<when it should be told>

R4- No, be told in advance that the problem can happen, you have to be ready for the future, meaning… this problem can also move forward… only then will you have to tell… how will you tell at the end time .. On end time, toh parents will also get upset, meaning what to do now… meaning the last stage has come, what can parents do at that time…. Tell me in advance that the doctors know that This means that where the position can reach the stove… where it cannot reach the stitch…
R6- What is more important to tell in advance. Along with this, prepare a little bit so that they understand so that together they do not get shocked, it means ...
R8- According to me, you will agree more ...
R5- If they have told the family members, but the mother of the child is… she can tell it only by looking at the situation… because some get it bad, some cannot do it… how to direct Will tell you that your child

is born dead or has a problem, cannot tell it… because it depends on them how the mother's condition is….
R4- It is a matter of whether you have to tell it at some time… then you cannot tell it at all…
Only the doctor will tell you the reason, for what reason it happened….

9. **In your view, what factors are important for acceptance of the minimal tissue sampling by the parents and family members?**
   (Probe: desire to know the cause, trust, confidence on the health care provider, who can be key influencer)

Now people tell me what to do
R4- So if such a problem happens then one must also get it done Dr. - tell them, can you agree
R5- I had heard, this is the case in front of me .... Once the lady was pregnant, when the doctor got the ultrasound etc., which was the child's retard, she was not progressing, the doctor who was with me, my check up She was doing .... So the doctor said ... Do not speak to the lady, tell the husband that your child is retarded, he is not progressing, so I cannot tell you to get abortion done right now from us. Even the big doctors are sitting, writing for A Hospital, for the sake of S Hospital who are bigger doctors than us.

Focus Group Discussion-Grandfathers 1

No. of participants- 9

Ques-1

R9- Nowadays most of the things are happening by eating and drinking

R9-As women are pregnant in eating and eating, they do not eat from time, nowadays children eat fast-food, this is most wrong. Eating fast-food causes a lot of disease, maida eats boiling, what is in it in momos, it is boiled hot cheese. Do you eat chowmein, it is fine flour, it eats, mother is also pregnant, eat pizza too, this is what girls are today, this is the reason for high level illness here.

R2- There has been a lot of difference between eating and drinking environment.

R1- Tension is also there

R9- Tension is nothing; there is a lot of difference in food. Pollution has become so much

R2-Cleanliness should be maintained in a house, which is to be made, we have to be hygienic, and try to eat at home, once in a while it goes on occasionally to eat outside.

R10-Especially for pregnant lady, this homemade food is perfect

R9- Homemade food is the best

R1- They make homemade

R9-We make it too, but the children who live alone, some job girls are boys, what they do is eat more from outside. Now a grandmother and grandmother who has four children will remain with one and cannot live with everyone. It is right, is there a child nowadays, he also wants to be different, he says mother, you will sit at home and we will keep servants

R5-Where do grandma and grandmother live

R9- This is what we say

R5- Grandma lives with her grandmother, yet what do today's children eat

R9- Grandma will scold, mother will try to scold

R5- Only mother can feed.

R9- Mother-in-law will also feed, if we want mother-in-law, we have also fed them. After so much time, we have fed children, the doctor had accepted the whole has been in bed for months, how has he been served, he has been fed only by scolding, not eating outside food. But the child who will be alone from where he will eat.

R10- Actually, whatever is in our food is not polished. Because it is not pure, lentils are not pure, vegetable is not pure so they are home to disease

R9- The house of disease is that and the reason for not making it is that they do not have time. Bring fast food, bring it to fruition, what do you do with it?

R10- Children also have a difference, there is a difference with tension there is tension. If a lady is going to office, then she also has tension

R9- Doing home also does office. Do all the work

R10- Home is also there is office

What you are saying, these people who talk in their food, in their behavior, in cleanliness, as far as people do where they are in their bus. Everyone tries as much as they can

R10- Yes they do

R9- Our government, which comes in vegetables, it is not pure either, it has got B chemical.

R8- It also contains fertilizers and chemicals.

F- Everyone should yield more; they only do so when it happens.

R10-Urea is so mixed. Will spend the night, in the morning bitter guard will be so much

R10-It will happen

R10- What else than medicines

R1- vaccination what else grows up

R9- That is the home of disease

F- How to take care during pregnancy

R9- Can't takes much care, those who come will eat the same from the market, now it is bad or good we cannot check that.

R2- We can try it at home, right. The purchase that has to happen will not have to be made.
R9- If you want to blossom well, you cannot feed a child
R1- Doctors speak, to feed fruit, to feed fruit
R9- The whole fruit goes out to be exported, what goes here
R1- don't know what goes
R9- The highest in India is good yields of fruits, but everything goes out. He is focused here. Now eat it, you are fed a thousand rupees a kilo or 500 rupees a kilo. That's the point
R5- for pregnancy check-ups
R5- In some government, some in private
R9- Some are seen in government, some in private, nowadays more gifts are ready to go in private.
R4 - will go to the government, there is also an amount
R7- Trust works in government, more in private.
R1 - More gifts are shown in private. Most of the gifts are shown in private only.
R9- But the one with the government says it right
R4-Government Hospital, I was going to speak the same word, go to the government, you will know everything right
R6-They are happier by giving money in Private, many people are happy by giving money nowadays.
R4 - Only by paying the money to get the brain test, he got the test done, it is ok
R2- A lot of people do not have time, by taking appointments in line, the line is long in the government, that is what they think. We showed up in private and the work was done.
R9-Rush is also in private, Rush is also in government, the government does not like to sit because there are no fans, there is no air, there is no AC, but in private the money is AC, it is, it is fridge.
R4-is satisfying, but if something is wrong, the private ones cannot do that much, go to the government end.
R7-private ones do a quick-witted operation, do not wait, the governors do a little bit of wetting for normal delivery, which is better.
R4- My granddaughter is now, now it is 5 years (years), now, for the whole 9 months, we have done all the tests which is an internal test or not, but it is done today but in the evening she told There is a hole in the heart of a lamp. We do not know first, in G Hospital, first in SG Hospital, then in G Hospital, then maybe in E Hospital, we did three tests, we did not know anything, there were 25-30 thousand tests, but as it is. It is telling in the evening itself that there is a hole in your baby's heart. Next day refer to escort.
F-So what are you doing now?
R4- Then when he was 9 months old, he operated it.
R9- Our children used to look like a thing, used to go to GK, used to show the whole thing, that so many fingers have been made, this is the heart.
R4- Used to tell us too.
R8-hole thing does not tell soon |
R4-hole not detected.
R9- From the first day to Nine Months, he showed us all in ultrasound, even today we have ultrasound. We used to take it from GK, this is what we do in GK.
R8- Not every man can go there.
R9- Not every person can get it done, but talking about private things, but the government people tell, but you would have taken it to the medical people. That is even 10 years ago
R4- Nowadays baby gets inside.
R9- Yes everything goes. After going to private, I am telling you that private people do not tell in the process of money, government people tell.
R6-private people do not tell about the money, my granddaughter had a HOA, she was three months old, where did she have an abnormal baby, then got tested after that, so intelligent child is born. The one who had said that it is abnormal, so people are doing all this work in the process of money.
R9 - Verma ji's granddaughter is not right, feet have been cured, give me the cloth.
R10- Look corruption is not it, corruption is it, it is a man thing.

R5 - Government hospital is fine, children do not go there.
R7 - Because you do not go there because it takes time, time is spent in the government, in private it is broken.
R9 - Cleanliness is in the hospital, now what is there in the outside countries, no one goes there in private, goes to the government, and they are good. And it is a good treatment.
R5- Hungry will also be seen, they will also go to private, which doctor charges more fees, doctor is good.
R4- Good hospitals have been done here too, but those who are working they are not ready to work properly.
R10- Money also rises in Government Hospital.
R4- No in their fun, doctors will work according to their own.
R1- You is speaking right.
F-work, everyone tries, to do, what is in the government that no one is worried about their salary in government.
R7 - He has to come.
R10- Well, to see that experience, which is more in the government.
R3- There is a lot in our house, it has been going on 8 months, and till now everyone was saying right, what is said to them that there is a 1 kg baby, consider even the eighth month, 15 days have gone, so far Tell me why not found it?
F-Whose? They must have done ultrasound right?
R3- Everything is happening, after four years, he is still confused. So far, nothing has been told, I have told that I have 1 kg, now I have gone back and then I speak.
R9 - Weight (weight) is decreasing inside.
F - does not decrease inside, will only increase but each time you do ultrasound how carefully they do it and how many ways they see it how much should it be and how much is it according to time.
R3- Now look 6 is running we are very tense.
Is F-sixth month or eighth month going on?
R3- No, the eighth is running, meaning the eighth is in six ways, so it is a matter of tension, see, it is less than 1 kg.
F-Not done, if it was more before then it will not be less.
R3- No, he spoke like this.
F-No, how does it never happen inside, if there is a living thing inside, it will not fall inside, either the previous one where the ultrasound was wrong, or the current one is wrong.
R3- Ever since he has spoken, his mind has been upset he is getting it done from one place.
Look at the R9-change cause and see someone else get it done.
F-You are getting impregnated from one place, now get it from someone else.
R3- The medicines that are being given right now, the alert ones are giving to drink, said that wait now, the color which is tested, it has to be done, now the medicines have been given, the brain has deteriorated completely. Is God has given one so after 5 years.
R9- Now gets someone else to leave it.
R3- Today he had to go, so I said that many sons only go, so I said that this time to take me, I ask him, just from the day I heard that the mind has become like this.
R9 - Which hospital is getting it done?
R3-Rohini Side is No in PV
8 - You are seeing what is inside quite often it is not known why this has happened, now why it has not increased its weight.
R3- One is speaking water, it is less.
F-Water is more?
R3-His water is less.
F-Water does not cause weight loss, it is different, there is a problem in the texture, for some reason it is troublesome due to the same reason, which is not known until you do the ultrasound.

R3- And then the doctor has also said that, as soon as it takes 9-months, may have to operate.
F-will is his or her blood pressure.
R3 - Blood pressure is too high to be broken up-down.
F-Not everyone, 10-15 people is in 100 which is pregnant. But there are other reasons too, which need to be found.
R3- The reason for that was already many, it was thyroid and later it was found that there is also stone.
R9-Nothing happens due to stones
R3-stone was not known before, it was found after conceiving
8-It is a different matter that no one had done the investigation, it was not known, and it was found when the investigation took place. Earlier it was also there, it was together, but there was no problem for him then it was not known, now when it was checked, it was found out.
R3- As it was just discovered, it was not known before that the weight (weight) was not increasing or something.
R4-ultrasound users may have found out.
F-address can run every time you will know when you check it.
R3- Whenever I used to speak to the majority, the son should keep in mind that the weight is increasing. We did not go together, the mosque is saying okay, it is right now, I have been able to find me since then we have been upset since then. God has given me so much time after that, be it a boy or a girl.
F- There you think, I am not getting satisfaction, show it somewhere, where it seems that both are talking about the same thing, if two people three people are saying the same thing then it means again There is nothing anywhere
R3- Doctor is saying that he will cover in one and a half months, he has given such medicines, now look.
F- You have to see the reason, if the reason will be such that it will cover, if the reason is such that it will not be covered, then thinking it will not cover it.
R3- Money seems to be so much, too much, let's say now, after so much time, it has been given to me, whatever it is, it should be fine, now there is money and nothing is visible in front of it, now we are just talking about the eighth. The month is over, so you have to do it before the operation time
F-She will know the condition when she comes. So suppose that there is such a pregnancy which does not cause any problem, their problem is known right now, there are many women who do not know, on the go, everything was fine, when they went later, they said that now they have trouble Or he said that we are not getting its heartbeat, this is not a problem, ever heard of yourself, what is the reason why you think this happens.
R3- The reason is not known but this happens.
R8- But they often speak like this, they say that its heartbeat is going to stop we will have to do the operation.
9 - To do the operation, what do you think is the reason. According to the medical doctor, there are some reasons according to you and there are some reasons according to them. What is your reason for thinking?
R4- domestic style
F-domestic, whatever you are, tell me which is the reason for the common sense, tell me what you think must have happened for this reason
R8- Due to the money, private people do the operation that brother Drakan who is in his possession, is closing, I have often seen this.
F-No, no one knows this, tomorrow you will go and ask me to show me what was happening, what will you show me, do not do this, at least, as long as those who do CTG till then in CTG Cannot see till then.
R9- Sometimes the ladies smoke also can happen. Drinks can be more than that.
R8- My girl Pizza from her place.
R9-Nothing happens with pizza.
R8- Listen Brother, you are right, the girl's heart was troubled, the girl's brain was overjoyed, all of her wounds were gone, she was admitted to Kailash Hospital, it was about 15 days visits also came. Yes agreed for two years that means neither you will eat anything nor will the child do it, and then it is not possible.

R9- He knows what that cabbage is.
R1-will is due to the cabbage meal.
F- It is not just the cabbage, what is it that when you eat a salad, which looks like a salad in a salad, you do not have it, which are small ones, if you do not clean it properly.
R8- Even after not heating, such sticks do not die. And because of that, this went to, and did not get well for a long time.
F- It is more in our North India because we mostly eat radish and carrot.
R10- No, roughage should be known.
F-roughage goes, but if it is not washed properly then it is not up, so what happens to live in it, in that many children can know at this time.
R7- Well, radish is also present in carrots.
R9- That means nothing is increasing.
R5 - Eat and eat, some people eat like this.
R1- We washes the baby only.
R2- We keeps all the vegetables by washing them.
R6- He has more illness.
9 - When anyone, ever heard of a pregnant woman's child is born and the dead is born, is it ever heard?
R4- Yes, heard many times.
9-F- Yes, why must be giving some reason for that, why?
R6-Malnutrition can be the reason and what will happen.
R9- First lady knows what she used to do, that someone did something, yes, earlier people used to say that.
R1-Must have broken tuna.
R9 - But nothing happens to him. But earlier they used to say like this.
F-Why don't you say that here?
R9- Some illnesses, some weaknesses
R4- It is said that the potty has gone in its mouth someone will say that its placenta has got stuck due to suffocation we have heard one or two reasons.
10-Some doctors give reasons, they tell, but quite often no reason is known, everything was fine, everything looks fine from above, they do not know. So the doctor gets sick there as well, what I can tell you.
R7- Yes is the right thing.
F-So far it was all right now it is. You will not know until you search inside. So what was next in the pregnancy, what happened, just like when you think about the disease of the child, you said something, like you said that it is for diarrhea, for pneumonia, what is the reason and what is the reason Think, it happens, what kind of disease do you see in more children?
R10-jaundice, typhoid
8-Children do not get sick?
In chorus is all said- why not.
R4- Cough, cold, hungry, children continue to have these gifts.
R1- Somebody gets cold.
R4 - There is a lot of stomach ache for the children, we say that there is a blossom, there is no pain in the children, and there is a lot of pain in the stomach.
R7- The doctor also says give this medicine, give the medicine at night and it will leave by morning. There are also two types of R9-sticks.
R7 - One says more food, one eats less food
F- He bites in the stomach and no one dies from the stomach ache, he moves with it, eats less food, appears to eat but does not die from it. Is any kind of disease the cause of death?
R9- Nowadays children get sugar inside. We have heard this many times in the hospital, it happens inside itself.

R2-Children do not have immunity, nowadays a child is caught anything happens if the children are not able to change the weather, the children become ill, they go out and the dust is gone folded children get infection.
F-And some reason you know?
R2-We see that we have two children in our house. Now we have grandson, he means eight years old, he has ectopic dermatitis, since when is HOA, we mean no doctor left, no homeopathic, no allopathic but he is not well. Stayed so what is the reason for this?
F-Whom will I talk to after whom it is not for everyone, is it for everyone? The other common cause, like we said, is that most of the cases are pneumonia, diarrhea, hunger or measles.
R9- It is mostly from dirty water. In the same way nowadays so much mud is coming in the water, so these diseases happen due to water. Or the water is not boiling like that, children take it nowadays.
R10- No, nowadays even RO says that water is not pure.
R2- If all the injections are given by the government, which is given by the government.
F- If time is sustained then it also makes a difference.
R9- Better water is said to be boiled water which is right, it is better than RO.
R1 - Freshly drunk they are also good, children are better than that.
8- Like we also try, you guys also try drinking water cleanly, according to yourself, whatever was stopped earlier, it has to be kept separate, or something has to be done or filtered. Nowadays, RO does a boil, they do it in different ways, yet there are diseases, they do not happen?
R1- Everyone tries but still happens.
F-Because all the reasons that are due to all the reasons, do not kill all the kits. It is not only here but everywhere in the world, it is okay, it is not known that it is known from above but there is diarrhea, but inside it there are 100 reasons, for what reason done it is not known as it is above. There is an investigation or research, according to what comes in it, the doctor makes his treatment, so if he does not know, then what the doctor will treat, who has come will do according to the report.
R2-Don't know, understand.
10- No, everyone looks like they are the same, everyone has diarrhea, there are two or four different ways, according to that they give medicines. In the same way as pneumonia, there is tuberculosis or pneumonia inside, or it is for allergy, or it is asthma, which makes you look like common man. It looks like it is coughing or hungry together well it can happen in anyone. The doctor also tells you, and he hears it, or he sees the X-Ray. It is revealed accordingly. But inside which many is due to so it cannot tell until the investigation is done. To do so, he gets many time to check, very often he does not get time, because he worsens so fast that he cannot do anything at that time. So if this reason is to be found out, how can we find out it? What do you think of people? Doctor people think of themselves
R7 - You tell me, how can I do it?
F-What do you guys think, how can we know more?
R7-Can I get test done, what else.
R10- Because nowadays physical work is not a physical thing, it is also a thing, and when there is no physical work, it is a diet, two things are men.
F-But that is the reason why I ate the diet wrong, either I will be thin or it will be fat, then it will be a wrong infection for me, no one dies immediately from this thing, no one dies instantly from thin.
R1- Yes, someone dies due to illness.
10- If there is an infection then that condition worsens. But the children who reach the hospital do some investigation of them, how can the children who do not reach the hospital know about them?
R4- Whose gift is not an idea.
R9- The disease is not known, the disease is different, we are getting some other treatment, even the doctor does not know many times.
R2- It is not known that the child is hungry, something is eating less, is getting loose, is getting sluggish, so parents do not know what is the reason that there is disease inside. It increases completely, go to the doctor timely to get the test done, then treatment can happen.
F- Timely, only if it goes away, but very often the disease does not give that much time.

R9 - There is a lot of turnaround that people take care of the house.
R2- Many time's people think that we cannot get this disease, that's why people become careless.
R9- This is also the fault of our people.
R1- Yes, it is our fault that we will not take it from time.
11- So many times when we sit in the emergency or ward, then we have children who say that it is very serious, very serious, so many times the person whom Sirius is saying is actually killed when he reaches. Whether it is on the way or whatever, then at that time we only know about two-three things that what was the HOA child, what was the HOA, whatever symptoms they tell, we think according to that Was But there we do not know that for those who practice in medical science many times, or many times, they should know that some investigation should be done only then and there are ways of checking that you have ever heard. By post-mortem? Have you heard about that?
R7-Post Mortem shows which person is dead.
R10- There is an accident.
F-You have heard that someone has an accident, it is there. Have you heard anything else?
R10- Did not hear the rest
R9- Some people like someone ate something wrong someone died of it what time did they eat it.
R1-eate poison also causes it.
R9- I have seen post mortem, done in tees hazari, my brother-in-law was his friend means he was 28-29 years old, he drank acid, he had an affair with someone from inside his body, he could not get married. He drank the entire two liter bottle of acid, his body turned black and blue, within a second, he was burnt so, as soon as we got the news, then went to the hospital with him, he said, "Death has taken place, take straight tees Hazari where the post mortem takes place". There was nothing inside him. We ourselves have seen it standing in such a mirror, saw all the irritated curry inside, it has drunk it, has drunk it at this time, it has been death at this time, at the same time it has told.
11-F-so, what you have heard, why most of the post mortem is done, if it becomes an accident.
R9- To get their parts also out, in that time they will donate their organs, they also do it for them.
R5- Will come forward.
R7-body part do for charity.
R9- There is some time period, if you are well then it happens.
R7- It is a kidney, it is a liver.
R4- It is a time limit that it should be done in such a time, eyes are useful.
F-eyes are the most, which gets used, according to the time, because they have a lot of needs in some, but the kidney liver has to match it all, without it.
R9- It takes a lot of time.
11- You people have heard about the post mortem, it is only where the reason is not known or the reason has to be made, otherwise, it is a police case where there is an investigation, because someone has to go in the police case Where no one knows anything, do not know the reason, they do it for the reason, what is the reason, it is said that it is an accident so someone says that it is an accident so but it is not an accident, it was left to her. Then he will say that the accident But when he said that he would drink liquor, the reason is changed, someone said that he had drunk poison and another said - no, it was ill, it is not known, the reason is not known properly. But we do this post mortem even in a police case where the reason is done to find out, But many times it happens that the family does not want to find out the reason, because like they saw that the procedure, everyone can be there or not. If you are asked to get someone done, will you get it done? If you are not forced, will you get someone done?
R9- If you want to know the reason, get it done.
F-How much will you want to know the reason? How much desire does people have to know the cause of death.
R9- People say that he died in the same way, you will beat him and chop it, you will get irritated, and so people do not want the child to cry. Those who are old think they think that one who has gone to a toddler is irritated from above and so he does not get it done, many do not want to get done
F-So but if no reason is known

R9- My sister's son was five years old, after coming down the bus, the doctor said that post mortem is a matter of time, but not done, my sister says no, her grandmother says I will not get my child I do not want to die and be irritated. He did not talk about Ambala. Two settles had passed, was crossing the road from above, one hit, he did not break, then hit the other, he finished the same thing, he cannot forget the scene, we can never forget in life.
F-So, even if you have a police case, you will not be able to do so unless the family grants permission. Ok, so this is the situation where there is a police case, where it is necessary to know the reason. But where there is a death without it, everything comes over the family only.
R3-The family decides.
R2-Actually, there is not so much awareness here on this thing; people need to know that it is important to know the reason.
10- This is what we want to know, what is the matter of awareness and what should be done which is necessary to make awareness big and something, it is important to know the reason.
R2- Now people here have so much emotional attachments, one of our people knows that his 17 year old boy got a stomach ache at night and took medicine from doctor, next day got up to take medicine in morning and he fell down, all The doctor says that he has had a heart attack, but later no one has tried to know what is the, he did not know.
F-so heart attack or brain problem, or what thing is not known cause, because it is quite a disease,
R3- Just Abdominal Pain that it?
R2- Yes, he took the medicine in the night to take the next day medicine, and he fell in the day and the same fell and he died and nothing was found.
F-symptoms are few but stomach pain can be for stomach and may be of heart attack or brain too, where HOA will not be known until some investigation is done.
R2- That is not there, there is no awareness here, no one has thought what would happen to someone.
F-So how to tell people that they agree.
R2- This is the only government, or doctors can only give awareness.
F-No-No, doctors think to tell you but how to talk? So suppose you were standing at that place?
R2- These things should be told in the school of the children that the children come quickly and convince the parents, what is taught to the children in the school, they come home and speak.
F-But children do not decide.
R2- doesn’t decide but parents listen to them.
F-But when you took someone in the hospital, assume that those who died were taken with them to the hospital, the children did not go there or the bond to decide there is the same which has been taken, decide the same. Whether to get it done or not.
R1-Only can do big.
R2-In India, people attach so emotionally, at that point of time nothing is understood, the mind does not move.
R1- At that time intelligence gets impaired.
F-But whatever has to happen, it has to be repeated.
R9- Then we leave it to the one above, God has written what he wants to do. Every tax is left on him, his will be the one who has died in such a way, his wish is done to him, he does not think of himself, he shields himself on everyone, he makes good, and his will makes him bad. That is only his wish this is what happens at the end. Your excuse will be sweet, this is what we think.
R7 - His life was like this.
R2- Here people try when they feel that we can get that thing back so people say that the person who has gone back, he cannot go back, he cannot come back after that what we have to try.
F-Whose relevance is only when it is understood that this disease may also be in our house, in our house, there is a lot of illness that goes on in the house that you call hereditary such as Bap, Sugar someone has a heart attack. In the second, the risk of heart attack increases by 3-4 times, which does not happen in the house, if we know. Like this, if the death of a child is due to some reason, the person who is inside, other children or the children around them also have the same infection.

R9- We will know only then we will be able to do something.
12-MITS- If only then, Mann has a child with measles and due to measles inside, it is not just for him, so many people must have gone through infection spread or whom to believe. They have gone from TB, they are not like TB they have to stay inside the same band they have to be spread, right? The So how will we know that as you told us that we have very few measures with the doctor, there is a measure to do post mortem of see what changes have taken place in which organ, and check it out. The second option that comes right now, you must have heard of biopsy, have heard it, have you ever seen it?
R4- Yes my husband, he had cancer.
F-What cancer did they have?
R4- Was here first on the tonsils and then on the food pipe.
F-Then was his biopsy curry? How was the biopsy curry?
R4- Had taken the liquid from here first, then took a sample 2-3 times with a needle, then had a broken skin, made an incision from inside, then reports came after that.
F-and has anyone ever heard of biopsy? So this is biopsy, was it very difficult?
R4- No, not after that, I have seen many difficulties after that, I do not remember that.
F-which was a biopsy procedure which was very difficult?
R4- no that wasn't so hard
F-Who is coming now, where we see that post mortem is not known because we do not want to attach too much emotionally, to cut those whose disfigurement speak, the body should not be different. So to speak for it as biopsy, do the procedure in them, which comes out so much, what comes out in biopsy, or pull it with a needle It comes out very liquid, a couple of drops comes in somewhere, or it comes like a tissue, where you suspect, you suspect a lung infection, then I cut the biopsy and it I have checked with it, no rip-off has taken place anywhere, so if I do it, and explain it to the family, what will they believe?
R9- Yes, maybe it will be accepted, if the cause is to be found out, we will have to agree.
R2- If you will be strong, you will agree, when the doctor talks to someone, such an accident, it should be immediate, all of the time it takes a lot.
13- No-no, it will be immediate, post mortem takes a lot of time because it is enough for it to be done, this procedure which is done in this, you should only check on the same, where it is, check it only in half an hour , Everything is done in an hour.
R4- Those who are strong will get them done from inside, which will be of a slightly soft heart, they will think again.
F-But those who are strong will also have to tell.
R2- No, we know 50 people, if one took action, then something can happen.
R4- Before the test was done by the doctor, the doctor told us that either he has a brain tumor, or there could be cancer, so you have to get this test done, then he had to get it done, then he had to get it done. After treatment, so had come, then it was gone, this so keeps going on.
F-As it grows, it gets compressed accordingly.
R4- It lasted 6 years.
14- So when is it to be told, when should people be told for biopsy that such biopsy can be done, you are thinking right now? No-no you don't, if you have a family or someone's death in a relationship, then the child is not aware of the reason why it is happening, so if they want to do it like a biopsy, then they When can I tell?
R7- Now tell me
R9- When someone has 7-8 births in their family, they will have something, then they will ask to know the reason for it, so that at least let's tell it to someone else, not to someone else. That doctor will tell you that you can have it tomorrow, you can have other children also, to do this, one has to first give a gift, only then we will know in the family, only then it will cure the other. Will work. One will be kept safe by the other.
16- Yes, how to tell them so, imagine that you would have been replaced by family members or people in your family. Are you going to do it?

R2- It can also be told through advertisement, in the newspaper, or more in TV, which people watch all day, it will be seen in it or there can be awareness as a society is formed, people should be put outside, Neither will you study nor will there be two out of ten people.
F-If you speak like this, then it should be started in the hospital itself, where people go more and more, and the place to tell everywhere.
R7- That too is correct.
F- Tell everywhere that at one place where all, most sick people go, tell there, it is good to apply here? No one went to the hospital, if they died at home, what should they do?
R9- Sugar was started in our family, first of all, they were grown, and no one was there, neither did the package come alive, first they were done, then they had to test that no one else could do it, then we got the test done, Dewar's Did not get it done, or it was done till then, but it happened after that, now I know it is going on in the family, my mother-in-law has also got sugar, now my brother-in-law has sugar, now what will be the reason? To eat or drink, what is the reason inside?
F-Like many people in your home have diabetes, the children you have right now
R9- Yes, we get them done.
Not to get F-done, they have to tell them that you are at risk.
R2-You're asking after death, aren't you?
F-No, it is also saying that you are talking about the disease, if I know the cause of death, then what to do first, do not know about the next ones.
R2-So the doctors say that the family is history, if we ever go to the doctor in such a way, for the sugar, they say that your elders have grown up, you may try to find out what is the reason for this in the children. No doctor ever spoke.
16- You can tell the cause of something not in diabetes, but someone in your home has diabetes or heart disease or something, which is your child, who is at risk, what they should take care of so that they have diabetes take it for some time after hitting the house or not. It is not as if what you have written will be the same.
R9- You can pay attention in eating and drinking, you can reduce sugar. Now in my family, my daughter gets in my family, firstly my sister's daughter came to know , it is no longer there, then second came to know, in the last I have done my daughter's breast, now Four girls had breast cancer in our house. Didn't know first Sister's daughter must have been of so, didn't know what to do before the medicine was done
F-If you don't know in a long time, then you don't know.
R9- After that, I got the check done, now last year my daughter also got her done and I also got her operated. Now the same doctor has said that you should get the daughter's daughter checked, you get them checked, then they have also given a hinga, that it should be installed. If you continue to investigate, then something will be known. There is nothing to be tested now the test is done in 6-8 months.
R2- This is a normal check sub, the reason is not broken.
R9- No, if it is known in the beginning then it can be treated as a treatment.
F- Cause it is genetic in quite some place.
R2- Now what happens with this disease, it is broken that take precaution.
16- This is what is needed, that is why, check for this, breast cancer is known, biopsy took it from him, but the reason for that is due to 3-4 things, so when I advance test I will know that if I go there and see that there is breast cancer, then it is over but it will not be known So if this kind of explanation is explained to those who are available in the hospital or whoever talks to them at that time, then they can speak to them or talk to them, then talk to them, how to be told and how to prepare?
R2- Nowadays tests are so expensive that poor people cannot afford, they think that we are doing well, then what is the need to get the test done, now medical treatment is not so expensive in the outside country, now in old age All tests are free.
There is nothing free on F-many.
R9- This is our wrong fame, there is a lot of cost outside.
F-Nothing in the world is free, it bites you from your salary for all people, or takes away many.
R2-People say that it is free there.

F-free is visible to them, it is free in only three countries, where the government puts its money, the government used to put it here too, but here our population is so much that the government will be full, half the budget will be put into it.
R2-Shouldn't be nominal
F-trying everywhere, the government on its behalf.
R2-Private doctors seek their consultation, on whom should the government be broken?
R2- Many times as children are read sick, they do not live, as if they do not have so much money, they cannot show a good place and if they do not give money to the doctor, they will not see it.
R10- Well in private, what does it mean to be sure that it is a shop?
R1-Private, we say that let's deposit the money first and then treat it
17- Have you heard about Aung-dan? So what have you heard from him? What is heard in TV, should I tell about it that way too? Ways to know the reason?
R10- No that too many times advertisement comes in TV.
R7- Yes, tell us that people should be vigilant.
R10- No actually in TV there is a panel which should be sitting.
R10- Yes, seeing the panel as we are discussing like this, the same way their panel sits, sometimes it comes on the TV, and doctors come.
R2- Comes as many of Homeopathic Ayurvedic, many people know that there is a problem, take it, it will be alright, people see it.
F-That's fine, your suggestion is ok that you know what the method is.
R10- TV or newspaper people read.
R7-No people believe newspaper, because nowadays fake doctors have sat, so people are scared.
16- So how should people be willing to know the reason? Get to the root and find out the reason, died and find out the reason?
R10- main is TV.
All people can see in R7-TV.
R2-Hospital is a big AD, who can tell the doctors who are sitting.
R5- TV news is watched by all.
R9- Once you have placed all the grandmothers and grandmothers on TV, they will tell you the Disadvantage, first of all everyone was on the Disadvantage.
R2-like people can go to the office, put it there.
R10- Now where do children listen to grandma's grandmother, nowadays, we do not even listen to many things, we say something, goggle it.
R5- Children are on the move, nowadays, children with jobs see the same thing nowadays, where do our children believe.
R2- Broke awareness in school or walk in office nowadays.
R7- TV will come to the hospital channel, so people can watch it on the go.
R4-TV is available everywhere.
R7- It should be that people become aware of the break.
F-Let's thank you all.

Focus Group Discussion- Grandfathers 2

No of participants: 8

1. **Where do people from your community/ locality usually go or whom do you consult during illness of children and newborn?**

Care during pregnancy

R5-Got a lot of trouble

R8- All were in the house, five were children, all five were in the house, all five are well and all grew up

R6- There is hope, people had got their names written, they have time to give themselves every month, then they come and give themselves, there is a hospital, the government has ESI hinges, needles are getting iron tablets to eat, its time. It will be finished so Asha herself speaks to her house where she wrote her name, this is the date that you go away, if you can't go, then I will go with you, in the same way every other third woman stays for 9 months. After this, if the doctor has written admit, then there is hope, otherwise he will take it directly to the hospital and after a couple of days the doctor gives leave, then we bring home.

R7- This is as it is speaking.

R8- Our child was all born here in the hospital, S Hospital, he had shown this and sent him to S Hospital, used to go to check for a month

R3- All the children were born in our house, small and big too, we did at home.

RE4- We end up at home

R10- There is a lot of difference between the former woman and now the woman. Listen, let me tell you what the difference is, what we are saying nowadays, we have done it at home and nowadays we are running in the hospital, injections are being done, then the children are there, I am not saying what is wrong So I tell you, I also check, or if I live on my side, I speak to go to the doctor, if it is my strength, then I make the child do it. Yes, I give birth to a baby.

How many people get it done (about delivery), how many people call?

R10- At least one or two people call.

R8- Our child was at home

R10- I can tell that I had cursed one of his granddaughters of eyeglass, I had born a boy, it must have been a month and a half, it has been a month for 10 days, I am upset all night so I will check once and check it. I am not able to take my bus, then take it to the hospital, then I said that check is not our bus, take it to the hospital and then go to the hospital. I have a child with them, that girl is alive in front of her. This old lady has three children, granddaughter, not the old lady but her daughter-in-law.

R8- Used to eat and eat the same morning time, made his moong dal khichdi for 6 days, started eating

R10-The change is this, you give the path 8 days, now you do not give 8 days at all, there is no path, even jaggery, egg milk, leaf is applied, milk is given daily by the people, in the past, we will not give such food. Here it is rivaas, bread fried ghee and give egg and milk.

R4- Only give bread

R2- There is no difference.

R4- Difference of food

R8- The former food was good, now the food is not good

R10-This daughter of today does not eat jaggery, she says we will eat sugar, there is an upset stomach, before eating jaggery, the stomach will remain clean, there will be no disease, now bread will ask for milk, will ask for egg, let the egg be hot Still it should be given early, but it should be rightly given, no one believes this tomorrow, we need milk directly, and we need jaggery and not sugar.

2. **In your view, what are the common causes of stillbirth and intrauterine death in your locality?**

R4- Yes in the hospital we saw no god

R5- Some children have no life left

R10-This old lady was grandson right now, O God, don't lie, give me ten, just God give me ten, His family called me, I said when the hospital is talking to you, why are you going to the hospital? , His boy

started saying, no aunt look once more, look once more, after talking, I came to my house and fell asleep, again the boy went and said, "Just watch once, very upset." See, I am sitting inside, I have not even seen it before and I have checked it, I am saying that I am feeling like I was trying to sit down, sit down, and sit down, I was trying hard His mother-in-law's mother-in-law, mother-in-law, come, but the child came out from the inside, I took a place with great difficulty, if the placenta does not stay, then the life of the woman ends and I too, because the midwife ends first. On the train, one lives in jail, so I told his daughter-in-law very well that you lie down

What are the reasons?

R6- There is no reason for yourselves, children do not allow treatment to be known in the beginning

R8- From the very beginning, he had timely, his, he has come on Wednesday and on Friday, he was born on Thursday only.

R6- It had been two days before, when he had explained everything to the daughter-in-law, yet he did not know what happened, he said that he had to do it at home.

R5- Do not know how it happened, what was the will of the above, what was it, since then the child has not been able to hold it till date, it has not happened

R7- It's been 8 years

We are trying to identify the causes of death in children and stillbirths, so that something can be done to prevent and reduce the number of deaths in children and stillbirths.

3. **In your view, would this information be valuable?** (Probe: If valuable/ useful, why? If not, why not?)

R6- Had shown it during pregnancy.

R7- Then he also took his child to the hospital.

R8- Well it is fine, but the baby has no hope of getting high

R7- Some treatment should be treated, if there is a cure, it is not known.

R5- I am getting it done, how much I got it done in ESI, and in what is it said, yes, I went to medical, got a lot of treatment. After that there was another one, there was such a big bloom in his stomach, then the stomach became weak then it meant that 4-5 years later it was finished.

Causes of death and knowing the cause

4. **In your view, what are the common illnesses and causes of child death in your locality?**

R9- Coughing occurs in the child due to cold, when the child eats something cold, or walks barefoot on the floor, it is due to him.

R7- No we are not at home

R6- Yes admitted, vomiting is going on

R5- Nowadays, the doctor is seen, most of them go to Lalkuna there is a Shimla doctor there too.

R9- Run more on the same

R6- So we go more and more, our child gets well from there.

R7- Yes we go the same

R5-These people go to S Hospital, these people made up their mind there, but they went to S Hospital, they said that this child is suffering too much, go to the hospital

R9- All of us are surgical children, by operation.

R7- Children go to Shimla only when they are sick.

R5- Yes, yes, he is very old, meaning that we are also very old that this will cure our child.

5. **Where do people from your community/ locality usually go or whom do you consult during illness of children and newborn?**

R9- Take to S Hospital in more serious

R10- My granddaughter was in trouble, she had a fever.

R9- Where will I get the medicine from anywhere.

R5- Many doctors have satchel impressions too, they have

R6- ESI should also go, S Hospital can also go most of the time going to S Hospital does not leave the girl and just gets hit in the head, throws hands and feet and then gets cold hands.
R5- Yes, the thing in the house is good, it will give the mobile to everyone, yes I am telling you the truth, it throws up its turn, it does so
R6- One is my son, 30 years old, he has never had such a disease, he suddenly got dizzy and he does not know anything what happened to him. Then two months later on 31 T also fell from there, everyone took him to ESI, he had to do a test, so he said that it will take a month or two months, so I said I will get it outside, There is a new hospital behind NN, from there I got an MRI of 10 thousand, then the test I took was said that there is a deficiency in some vein, not telling clearly, another test ECG has been done, they will give the report on January 21. Yes, the medicine is going on.
R5- Both are giving medicines, for the first time in life, it has never happened before.
R7- When R came to know, he said that you did not tell me, so I said I did not even know it happened for the first time, I had not even been to the ESI brother before walking away from my house, but all of that God was there to tell me otherwise. I used to move here and there, but it has happened inside him, he sits like this and gets chocked, I say why Ashok does this, he says mummy I know but I don't do it by myself, I know my Mundi is happening like this, but I don't do it by myself, what does it do with lack, Will do this, 2-4 minutes will not do, 2-4 minutes will do.
R8- Only tell when the child is being shown only then, according to the child, you cannot understand that the eldest was of my elder brother, he started having seizures, then later on my boy, then after that I had a girl, then later, and another girl, then I had two girls, there was a boy. And now knowing a girl is running, a boy has finished, now the son of a brother is running medicine, he has a granddaughter, he is also walking, when he is three years old, he gets a visit.
R10- In some way, we are showing all the children again in A Hospital, so now we have one girl in it, the other three girls have been cured, a boy has also been cured.
R7- This doctor will cure you, may not come out
R7- As time is outdated if it is early then time will take less.
R7- As this is happening, she will quit the medicine
R7- As if not from the beginning
R7- The doctor can tell him which one to start
R7- I was talking about them as they are happening, the treatment is going on, the reports are being made every single day, it can tell that it means the beginning.
R7- If the doctor does not tell you any disease, then you will have to ask them forcefully, tell me the reason, Khali says it is okay, this is the problem, what does this doctor sir mean, they brush it off.
R5- Now i will go neither i will ask
R6- We will tell you again and then we will go ahead.
R8- We will tell you then we will further
R5- I had gone before to show my girl, it's been years, I was saying, how to eat medicine and what to say, when you will be proud, you may have it, your child may not survive and you are not left well The medicine has to be eaten equally, now it is eating the medicine equally, now it has been given medicines which have been increased, it was telling of 500 mg, now it has been increased to 700.
R8- That's why I'm taking hospital
R6- I am going to the hospital
R9- What should i say that's the matter
R6- Our boy had dengue, then he got a lot of treatment, he got cured when he did medicine
R7-Telling the daughter-in-law of this boy, get the whole treatment done here, with a satchel, you came and started crying with me, the aunt has said such a thing, I have said that you are crazy, you can do 100 fifty daily medicines It is not possible to do a blood test for 200 rupees, there will be a report on the blood test, what is the reason, what is the deficiency, what is the disease, immediately the tumor was removed, the aunt said if you do not say, then I do not know what. Gets, most fever is first blood test
R5- The fever did not stop.
R7- There was a lack of plasma, it was saying that aunt if you do not speak

R6- Is a medicine.
R9- Goat's milk, papaya milk was crushed.
R7- There was dengue my one eye vein is still inflated
R7- When someone went to the bathroom and picked up the father, it used to go like this.
R7- At that time, doctor, when someone lifts my hand and shouts, only then leave someone else, he also speaks fever with a broken bone.
R5- Yes, he goes with goat's milk.
R6- We also drank goat milk
R10- Yes, drink papaya's boils, and the medicine you bring home.
R7- The one who dead my boy, lacked plasma, I used to bring 20 rupees, the doctors used to treat me, but I used to do what people used to say to my comfort, that no one can get anywhere, in my house. If anyone has a fever already, then I do not carelessly because I advise everyone, or do not give medicine here and there, if the fever does not go in two to four days, show it to the doctor. I do not think that we have to cry like this next, I will tell everyone immediately, brother, do your treatment or do a blood test which shows what is the disease, get your son done, in two to four hours the report came that aunt For 10 days, he has been losing money, he has given his guess medicine, he had not given the medicine for that disease, who gave the paper, which medicine was said, when his record will come out, only if the blood is checked, then the doctor told him.
R7- Doctors will give medicine in it, avail will not give allergies too, doctor sir, and I do not know that while visiting the doctor ... I know a lot.
R7- The mother-in-law took two months, the leg was broken, it took two months, we had gas, I had lost so much medicine since childhood and ESI lost everything, referred me to G Hospital, she did not test me.
R7-Oh doctor sir, I told you to come with a towel, I took an ultra sound with a towel, I will wipe it with this, then I said, "Mother, I should be like this, but doctor, such an ultra sound is not there, then everyone says this." Test, now what will be done in AC alone Dr. Saheb is giving a boiled pulse saying that there is tea ghoti (swallow), tears like sweat, when this test report came I got treatment in G for two years.
R7- One day, 6 x 7 x ray was done, my son is there, but there was nothing to do but nothing happened and the person who referred to the hospital got the report again, but from that he tested me with the telescope and it was found that water is also tea tea. Also, the PU feels as if the wound is full, the path is still moving and the stomach is moving like this, but the doctor is all right now, then someone happens, I advise everyone.
R5- You means the gas is gone
R7- If you get so much treatment, why won't you go
R5- So do our treatment too.
R7- Go to G Hospital, Cure That
R9- Did not go to the doctor
R5- We are walking on the medicine
R7-Doctor sir, I went to ESI, one day my pain took place, I was in my house, I came from ESI; Go, put 9 injections in a bottle, now tell me, I spoke to these unconscious people, my tongue means the voice is not breaking, they said to these people that I am speaking, you are listening to me, my voice is not Is it all inked People got up and took the ESI, the ESI person was not carrying it, he was saying, you have brought it to death, when the people who were taken from here yesterday said why not bring it yesterday, in an unconscious state, I got upside down, then I got the bathroom, When I went to the bathroom, the doctor came to my senses and took the same treatment in the ESI emergency, started the same treatment, if I did not go, it would have died, the ink was completely gone.
R9- Giving medicine on its own, mouth is drying up, mouth was completely dry.
R7- I didn't know anything
R7- I tell everyone in my neighborhood, and when something happens, I also come to you for advice.
R7- Doctor sir, I have not left any hospital, give an empty address, a woman came and was working, her hand is hurting, then the doctor injected her, now what was the injection, she got her full hand from here ..

R7-No, it did not swell, it started to dry completely, it did not turn like wood, it did not turn from such happiness, tell it was only pain the pain was injected with pain. Got chopped
R9- Got chopped
R7- Otherwise, what she would have done, she did not know what she had done, that it had happened, she had lost such a big hand.

6. In your view, what are the common causes of stillbirth and intrauterine death in your locality?
F- Then the same will come from your report, you will know from that what is the other thing, when the child is more serious then he goes to the hospital, then what does he do if the child has died in the hospital?
R7-Little children were safe for me, he started doing it all of a sudden, then I checked it and then people started saying what the upper air is, then what I did, then I checked him and I have a Reshma Hospital in it, I have a hospital for children. The doctors have gone there, here it is not, you should come during the day, I said there was no problem during the day, then from there I was picked up and moved to another hospital and there I was doing the same way, then backbone Took all the tests, what will the child know now
R7- There were three days
R7- The doctor did not tell me anything clear.
R7- My big boy finished all over the place, the doctor with ESI said that this is a test with a bone marrow, I also got him done for a month and a half from Mumbai and said that another test is in Chandigarh or aims, he was referred to A Hospital , Said there, it will cost thirteen lakh rupees, I said doctor sir, I will give me thirteen lakh rupees, my child should be fine, I will sell the house, if the child is fine, then he would have eaten a boy of twenty three years, he must have known what is the problem. , The first doctor called me and said that ladies, you should make your heart stone, when can it happen, yet I have comforted my heart, O God, please give me a little comfort, be a woman to make so much heart stone keep my boy would have become a BCA course, he was not in luck.

We are trying to identify the causes of death in children and stillbirths, so that something can be done to prevent and reduce the number of deaths in children and stillbirths.

7. In your view, would this information be valuable? (Probe: If valuable/ useful, why? If not, why not?)
R7- Doctor, you will tell me this

8. How autopsy/post-mortem is viewed by you and community members for knowing the cause of death? (Probe: views, concerns and believes in the community regarding autopsy)
R7- Yes heard, as someone hanged, suddenly died, his post-mortem is done for what reason, he is killed by strangulation or killed by giving poison. , It comes in, this man is killed for this reason, when I saw him in the same careful India it shows all.
R- Do not see the doctor.
R7-I see both those careful India and crime petrol, I see both of them a lot.
R6- Watches till one o'clock in the night
R5- He finds out the reason.
R6-What is disease, after death, we see what happened due to deficiency, a disease is killed.
R7- I have felt what is the benefit of being persuaded, I have not left my child for a second, see if everything is done, my child will not come back by doing that, then I will not get it done, I immediately took it down.
R7- Why does the person tear things in the accident, what disease did it have, what happened to the accident, why do it?
R7- What the public there says, it says give a good response, take out the kidney, remove the eye remove the good things.

R7- People say, as the doctor says that he will have a kidney, he will have it, then he puts it, one life is gone, another life will be found.
R7- If I had listened, I would have told you, doctor, I thought I had not heard it.
R5-Doctors say that life goes on with a kidney.
R8- Yes, when those people say that they will be home
R7-There is one whose both kidneys were defective, then his father gave him a kidney, he said, I am old, how much my life is, well he gave it to the son after four months, he said if my child had survived Stays true
R5-Now I have a granddaughter no matter what happens to me, I am disturbed by cutting the rounds of the hospital, visiting her from the beginning then I take her to A Hospital on tour, also take S Hospital, show both places I am equal

In some instances, autopsy/ post-mortem is not possible due to various reasons. To find out the exact cause of death and underlying disease, some tissue biopsy (collection of very small sample using needles) and fluids (blood, urine, etc.) from the body of the child after they die, as done for investigation. But these samples are to be collected as soon as possible after death.

9. **How do you view minimal tissue sampling procedure as a method for knowing the cause of death?** (Probe: views, concerns and believes, religious aspects, comparison with autopsy)

R7- Will know that due to this disease
R8- What do we do, why did we let their body be destroyed, this is what the public thinks.
R3- The test which was a bone marrow, he said, "Go out Mataji, you will not be able to see that test."
R4- You tells me, it is not just about the woman but to see.
R7-A weak heart cannot bear
R5- Some like this weakness increases, many others say I will be able to see

10. **How the parents/families of a child/newborn who has died, should be approached by the hospital doctors/staffs for the minimal tissue sampling procedure?**

R2- Sir, we do not know, If they can explain well to the parents and family members, it may be accepted.
R3- I also agree.
R5- They should tell and assure no cutting and removal.
R4-The time taken should be less.
R8- may be the elders of the family assist in deciding.

Focus Group Discussion-Grandmother 1

Number of participants: 9

1. **Where do people from your community/ locality usually go or whom do you consult during illness of children and newborn?**

R1-When there is sickness in children, such as fever has come, cough has happened and like this mother, this day… Mother comes out in the day, measles comes out… there is another… it also comes out. ... So we do a little bit of it at home and go to see the doctor

< Which doctor do you consult the most >

R3-Then you go to the doctor, benefit and then go to there

R6-- No, that is not good, the doctor says that the child is this measles

<what do you think while going to a facility for the first time>

R2- Most of the time we go to BS, we go to the most Bengali of all… get well… he also takes medicine…

(So, you go to a place where you have trust)

R4-Yes trust first

<what do you think>

R3- Thinking according to trust, you have to go first or think according to money… think of money, think of trust too… even if it takes 200 instead of 100 but the child gets well

<how we can know this>

R7- Do not know, now as we went to the government, now we have given medicines from there, the child is not comfortable, so I think, this thing is not comfortable with the medicine, now we are not getting any other place. Should be shown..... So we go to another place.....

1.1. Which ones are usually accessed for non-critical illnesses and why?

<Non critical illness>

R1- Then you go to the doctor, benefit from and then go to there.

<How do you find out?

R6- No, it is not good, the doctor says that the child is this measles

<what do you think before visiting the doctor for the first time>

R6- Most of the time we go to Babu Sharma, we go to the most Bengali of all… get well… he also takes medicine…

Dr. goes to a place that has faith first

R4- Yes trust first

<What do you think>

R3- Thinking according to trust, you have to go first or think according to money… think of money, think of trust too… even if it takes 200 instead of 100, but the child gets well…. .

Dr. - How will he find out?

R6- Do not know, now as we went to the government, now we have given medicines from there, the child is not comfortable, so I think, this thing is not comfortable with the brother medicine, now we are not getting any other place. Should be shown ..... So we go to another place .....

1.1. Which ones are usually accessed for critical or severe illnesses and why?

<critical illness or severe illness>

R1- Go to S Hospital then to, then you will have to run in government, S Hospital only runs .. Go to MB, go to S Hospital..

R3- Now I tell you, the child of my elder daughter has become ill…. She was given medicine twice by a doctor …… Now the child's health deteriorated, then we, we thought That we take S Hospital …. So nobody told us that Kapoor is a doctor in Munirka… Take him here, the health of the child is very bad…. Poor ... means that there are so many people in S Hospital, to go there, go here Get number 10, - Get number 20 - You can show it better here…. Then we got him admitted, then immediately got his check up, then immediately his action started…. Blood test Started doing it, put glucose in it… Like this, they said, collect so much money… Then we deposit the money… In three days the child is fine and comes to

the house… 15 thousand rupees a month. ... this thinking is done ... and nothing is there ... go to S Hospital, go to number 10, - all think this is to be fixed Then put the line, then you go out like a child, children grow up, they become young, they become old, they start putting their numbers… .. Many people are like, let's go brother and get well treatment. Go again… - Everywhere a human being goes on thinking… He thinks that the people should get it right, we think that even if we will not eat bread, one day we will not eat bread, we will not eat 10 days but this is what a child does Ho… you will eat with salt, your children are right…

Pregnancy, outcome and care seeking practices

You are aware that several of the pregnancies in the area/community terminate with stillbirth (death during delivery) or intrauterine death (death inside womb).

2. **Where do people from your community/ locality usually go or whom do you consult during pregnancy and delivery?**
   2.1. **Which ones are usually accessed for routine pregnancy check-ups?**

R3- See, I will tell you my own, my daughter-in-law too had children… So to what they mean, here, we have a dispensary here…… in that, getting checked time to time, Ok, ... and they used to give medicines, rather, to eat ... When they called whenever we reached there .... Then they said, when the time of the baby came close, they said go the desired market He has a…, go there and get an ultrasound done…. He also got us done… Just as doctors used to say, we used to get them done…. The doctors used to say, that's how we got them done ... but the daughter-in-law's children were in the house, normal
And my daughter who is my daughter, both of her children were in S Hospital..... Time to time all the tests were done, and the child was well looked after, all the work was done ...
R4- Ours was a daughter-in-law, she had two children, in Munirka… who was born in her house, she was weak….
R5- I too was born in the house ..... (Yours, grand children, grand daughters) who are grand children, grand daughters…. Daughter was born in S Hospital and took place in a house …… in Bahu’s house also…
R6-- Our gifts have been filled in S Hospital... then we have also been at home ...... now people have to go back to S Hospital..... whether it is in the house or in S Hospital.... ..Where the younger daughter was gone .. Now the health had worsened, yet medicine, went, show up….
Which ones are usually accessed for delivery or pregnancy with complications?
<So, you uusually go to munirka or S Hospital?>
Yes
R5- When writing, S Hospital gives it to someone. so what
R4- It does not happen, Sir, there is a little problem among the government, people who have money, they fill it in the private, who do not have it, they go to S Hospital.....
R5- It is not necessary that there is no care in S Hospital, it is very good… (It is a matter of luck)… We doctors do check time to time… There is nothing like that… because I went I have seen ... (yes we have too) ..... a lot of people say that they do it there .. they do it ... So they carry it here in privets ... Now they do the operation and then collect the money…
R4- Can't get it done in private, so you had to run in government again .....
R3- We had done it in private, there was not much money to go to government
R5- There is some SG in VV … his toh rate is high fee… If this child is admissible… such as the treatment of women who have children, then the treatment will continue… After that, the operation will tell you .... The operation takes you directly to the table .... Submit 50 thousand, deposit 60 thousand ... This is his .....
R4- His operation was done in S Hospital, then he was puss, went to SG .... 200, 300, 500, 1000 rupees is paid for a room ...... thousand rupees Life is better than ...
<How do you decide about which facility should be accessed>

See how many times I have been to S Hospital, I take my daughter so well all the checks are done… very well… I came to you… People say that our time is going bad .... Let us run away in the private ... Look at what the private person is doing ... because he is eating money ....
R5- sister became very angry in this, did not come once to get the cleaning done… .. Then admitted there… Then in four days… Then she went…

Causes of death and knowing the cause

3. **In your view, what are the common illnesses and causes of child death in your locality?**
   <common causes of illness, causes of child death>

R3- Sir, there is such a thing here, which is a dirty place for us, - There is also a lack of calcium in the children - a little bit - then the children do not even eat properly, the mother goes to work a lot, they also care It is not given, the child ate it with dirty hands, what has he done .... It is also not like that a mother is left and the child goes away ... She has not washed the troupe, she has not washed .....
R7- And what is the reason this is the reason .... It is not right in the government, to run in private, it is just this ..... that the children have got what they have got and now their grandson falls Gaya not from here .... In our home, such a huge injury took place, S Hospital took the emergency .... He took the emergency and stitched it there ... Then there was the doctor of the children, then there Shifted .... Now my grandson is fine ..... fell into the house from bed .. here from the bed .... it all exploded, this job too ..... so now okay

<causes of child death >

R7- Now toh toh about it… Look vaccination we all get it … Now look, yesterday there was a girl, healthy, went in a fever of two days… She did not speak at S's house .. ... and such a beautiful girl ....- This girl who is going to press her ... In a fever of two days .... A girl of three years .... She took him to MB ... in MB Admitted him….
Dr. - Now that his fever was two days…, yes, his mother telling him for two days… Now what has happened is not known, the reason is not known, why
R4- It is not known that the parents think that the child should be cured, it turns around and runs around so that the child is cured… .. until now. Child only, what parents can do…
<cause of death>
R 1– (while laughing) Now this, you are a doctor, don't you…
R2- Now we have heard your voice, Doctor sir, you listen…
R6- Where does the doctor tell you, what is the disease to your child, take away, did not get well here, to take the place to another place… Doctors also run and kill…
R5- Now the disease has become such that it is not known what is the disease, it is known that there is disease…
R6- Now you are seeing a lot of good things and what will happen tomorrow?
R5- So sir, please check what is in our stomach?
R7- Yes, got the investigation done, now look, there have been better investigations in the world, have not left the hospital .. nor have the operation done ....
R4- All the papers are that one day one has to stay here

4. **In your view, what are the common causes of stillbirth and intrauterine death in your locality?**

We are trying to identify the causes of death in children and stillbirths, so that something can be done to prevent and reduce the number of deaths in children and stillbirths.

5. **In your view, would this information be valuable?** (Probe: If valuable/ useful, why? If not, why not?)

R3- See, it is important to know, but the doctors only know this thing, what has happened brother .... They say that your children did not bring you on time. Now we understand that too .. Let the body do the work, it has fever, it is over… .. know what the parents think, what to do…. (everyone starts speaking together) Now the fever is 15-15 days ..... so nice girl ... Now, according to your liking, everyone tries not to do it, that everyone can be saved…. It is revealed that the child has passed away ……

R1- Now this is gone or passed away Sir, now what should happen next ....
R2- don't know , we are sitting here ... You have called, we have come .... (Laughing)
R3- Doctor sir, you can do so much, you can only tell …… we are not educated so much, we are illiterate people… we do not have much knowledge from you….

In some cases the children die before even the exact cause of illness and death is identified/ investigated. In such cases, doctors suggest autopsy/ post-mortem to identify the cause(s) of death. Similarly for the stillbirths and intrauterine deaths*, doctors suggest autopsy/post-mortem of the baby to identify the cause(s) of death.*

6. **How autopsy/post-mortem is viewed by you and community members for knowing the cause of death?(Probe: views, concerns and believes in the community regarding autopsy)**

<post mortem>
I am Dr. Ok, and one way is, call it postmortem .....
R2- I have heard that a person has died, he is in that ...
R3- Some people also want that we should find out how humans have been killed, some people do not even want….
<concerns and believes in the community regarding autopsy>
Dr - Why don't they want that?
R7- Now that is their understanding ....
Soil deteriorates
Rip it apart ....
Take everything out…
<views,concerns and believes in the community regarding autopsy>
R3- They want that the husband is not sick… need to be aware… It is a matter of our own thinking… Most people do not want what we see, because… They feel that brother should not be like this .... Do not be beaten, do not be beaten ... In that we have another way out .... What is there in it that some people want to get it done ..... They also follow him a little bit about why to do this thing .... So there is a way out to us that you have heard like a biopsy.
R6- Yes heard ...
R1- But people say that they tear such rips, they tell like this ... So if I say to choose between post mortem and this one who is a biopsy, then which one will I choose?
R- The things you say ...., if you choose that, why would you choose ....
<concerns and believes>
Dr. - So if you choose that why would you choose ....
R8- That means they will take it out of the needle… the rip will not tear… the body will be found right, safe and safe, it is possible to see, O God, this should never happen to anyone.

In some instances, autopsy/ post-mortem is not possible due to various reasons. To find out the exact cause of death and underlying disease, some tissue biopsy (collection of very small sample using needles) and fluids (blood, urine, etc.) from the body of the child after they die, as done for investigation. But these samples are to be collected as soon as possible after death.

7. **How do you view minimal tissue sampling procedure as a method for knowing the cause of death?(Probe: views, concerns and believes, religious aspects, comparison with autopsy)**

8. **How the parents/ families are likely to view the minimal tissue sampling procedure as a method for knowing the cause of death? (Probe: anticipated views, concerns and believes, religious aspects, rituals, comparison with autopsy)**

9. **How the parents/families of a child/newborn who has died, should be approached by the hospital doctors/staffs for the minimal tissue sampling procedure? (Probe: who, when and how and whom should approach, who can be key influencer)**

10. **In your view, what factors are important for acceptance of the minimal tissue sampling by the parents and family members?(Probe: desire to know the cause, trust, confidence on the health care provider, who can be key influencer)**

11. **In your view, what would be the most suitable method(s) of educating the community about the minimal tissue sampling?(Probe: medium, place and target audience)**
    <educating the community>

R1- Sir, we do not know, you should understand…. Are, they are just explaining the meaning…. So you understand this thing… Those who are speaking….

R2- Sir is running in reverse nowadays, these vegetables are running, cereals are running… medicines are being used in all…. Go .... become 100 years old ... Now you are 80 years old ... 30 years old brothers ...

Honesty, the truth used to go on .... now it doesn't work

R8- Now that bus goes meaningless ... hahaha

Snatch them, pounce on them, keeps going like this…

R6- What difference does it make, is it suffering….

Where is the trouble coming, everyone is getting similar ...

First hand walks, so the human being was right….

There is also adulteration in oil, there are medicines, there is also adulteration in them….

R4-yes right

R8- You have also come with some benefit, you have not come without any benefit…

12. **In your view, how the community leaders/influencers like you can contribute/ participate in such activity? (Probe: who or what is it, what did you do in this context)**

13. **Any other specific comments/ observations.**

Focus Group Discussion-Grandmother 2

No of participants: 9

1. **Where do people from your community/ locality usually go or whom do you consult during illness of children and newborn?**

Care during pregnancy

R5-Got a lot of trouble
R8- All were in the house, five were children, all five were in the house, all five are well and all grew up
R6- There is hope, people had got their names written, they have time to give themselves every month, then they come and give themselves, there is a hospital, the government has ESI hinges, needles are getting iron tablets to eat, its time. It will be finished so Asha herself speaks to her house where she wrote her name, this is the date that you go away, if you can't go, then I will go with you, in the same way every other third woman stays for 9 months. After this, if the doctor has written admit, then there is hope, otherwise he will take it directly to the hospital and after a couple of days the doctor gives leave, then we bring home.
R7- This is as it is speaking.
R8- Our child was all born here in the hospital, S Hospital, he had shown this and sent him to S Hospital, used to go to check for a month
R3- All the children were born in our house, small and big too, we did at home.
RE4- We end up at home
R10- There is a lot of difference between the former woman and now the woman. Listen, let me tell you what the difference is, what we are saying nowadays, we have done it at home and nowadays we are running in the hospital, injections are being done, then the children are there, I am not saying what is wrong So I tell you, I also check, or if I live on my side, I speak to go to the doctor, if it is my strength, then I make the child do it. Yes, I give birth to a baby.

How many people get it done (about delivery), how many people call?

R10- At least one or two people call.
R8- Our child was at home
R10- I can tell that I had cursed one of his granddaughters of eyeglass, I had born a boy, it must have been a month and a half, it has been a month for 10 days, I am upset all night so I will check once and check it. I am not able to take my bus, then take it to the hospital, then I said that check is not our bus, take it to the hospital and then go to the hospital. I have a child with them, that girl is alive in front of her. This old lady has three children, granddaughter, not the old lady but her daughter-in-law.
R8- Used to eat and eat the same morning time, made his moong dal khichdi for 6 days, started eating
R10-The change is this, you give the path 8 days, now you do not give 8 days at all, there is no path, even jaggery, egg milk, leaf is applied, milk is given daily by the people, in the past, we will not give such food. Here it is rivaas, bread fried ghee and give egg and milk.
R4- Only give bread
R2- There is no difference.
R4- Difference of food
R8- The former food was good, now the food is not good
R10-This daughter of today does not eat jaggery, she says we will eat sugar, there is an upset stomach, before eating jaggery, the stomach will remain clean, there will be no disease, now bread will ask for milk, will ask for egg, let the egg be hot Still it should be given early, but it should be rightly given, no one believes this tomorrow, we need milk directly, and we need jaggery and not sugar.

2. **In your view, what are the common causes of stillbirth and intrauterine death in your locality?**

R4- Yes in the hospital we saw no god
R5- Some children have no life left
R10-This old lady was grandson right now, O God, don't lie, give me ten, just God give me ten, His family called me, I said when the hospital is talking to you, why are you going to the hospital? , His boy started saying, no aunt look once more, look once more, after talking, I came to my house and fell asleep,

again the boy went and said, "Just watch once, very upset." See, I am sitting inside, I have not even seen it before and I have checked it, I am saying that I am feeling like I was trying to sit down, sit down, and sit down, I was trying hard His mother-in-law's mother-in-law, mother-in-law, come, but the child came out from the inside, I took a place with great difficulty, if the placenta does not stay, then the life of the woman ends and I too, because the midwife ends first. On the train, one lives in jail, so I told his daughter-in-law very well that you lie down

What are the reasons?

R6- There is no reason for yourselves, children do not allow treatment to be known in the beginning

R8- From the very beginning, he had timely, his, he has come on Wednesday and on Friday, he was born on Thursday only.

R6- It had been two days before, when he had explained everything to the daughter-in-law, yet he did not know what happened, he said that he had to do it at home.

R5- Do not know how it happened, what was the will of the above, what was it, since then the child has not been able to hold it till date, it has not happened

R7- It's been 8 years

We are trying to identify the causes of death in children and stillbirths, so that something can be done to prevent and reduce the number of deaths in children and stillbirths.

3. **In your view, would this information be valuable?** (Probe: If valuable/ useful, why? If not, why not?)

R6- Had shown it during pregnancy.

R7- Then he also took his child to the hospital.

R8- Well it is fine, but the baby has no hope of getting high

R7- Some treatment should be treated, if there is a cure, it is not known.

R5- I am getting it done, how much I got it done in ESI, and in what is it said, yes, I went to medical, got a lot of treatment. After that there was another one, there was such a big bloom in his stomach, then the stomach became weak then it meant that 4-5 years later it was finished.

Causes of death and knowing the cause

4. **In your view, what are the common illnesses and causes of child death in your locality?**

R9- Coughing occurs in the child due to cold, when the child eats something cold, or walks barefoot on the floor, it is due to him.

R7- No we are not at home

R6- Yes admitted, vomiting is going on

R5- Nowadays, the doctor is seen, most of them go to Lalkuna there is a Shimla doctor there too.

R9- Run more on the same

R6- So we go more and more, our child gets well from there.

R7- Yes we go the same

R5-These people go to S Hospital, these people made up their mind there, but they went to S Hospital, they said that this child is suffering too much, go to the hospital

R9- All of us are surgical children, by operation.

R7- Children go to Shimla only when they are sick.

R5- Yes, yes, he is very old, meaning that we are also very old that this will cure our child.

5. **Where do people from your community/ locality usually go or whom do you consult during illness of children and newborn?**

R9- Take to S Hospital in more serious

R10- My granddaughter was in trouble, she had a fever.

R9- Where will I get the medicine from anywhere.

R5- Many doctors have satchel impressions too, they have

R6- ESI should also go, S Hospital can also go most of the time going to S Hospital does not leave the girl and just gets hit in the head, throws hands and feet and then gets cold hands.
R5- Yes, the thing in the house is good, it will give the mobile to everyone, yes I am telling you the truth, it throws up its turn, it does so
R6- One is my son, 30 years old, he has never had such a disease, he suddenly got dizzy and he does not know anything what happened to him. Then two months later on 31 Tariq also fell from there, everyone took him to ESI, he had to do a test, so he said that it will take a month or two months, so I said I will get it outside, There is a new hospital behind Nehru Nagar, from there I got an MRI of 10 thousand, then the test I took was said that there is a deficiency in some vein, not telling clearly, another test ECG has been done, they will give the report on January 21. Yes, the medicine is going on.
R5- Both are giving medicines, for the first time in life, it has never happened before.
R7- When R came to know, he said that you did not tell me, so I said I did not even know it happened for the first time, I had not even been to the ESI brother before walking away from my house, but all of that God was there to tell me otherwise. I used to move here and there, but it has happened inside him, he sits like this and gets chocked, I say why Ashok does this, he says mummy I know but I don't do it by myself, I know my Mundi is happening like this, but I don't do it by myself, what does it do with lack, Will do this, 2-4 minutes will not do, 2-4 minutes will do.
R8- Only tell when the child is being shown only then, according to the child, you cannot understand that the eldest was of my elder brother, he started having seizures, then later on my boy, then after that I had a girl, then later, and another girl, then I had two girls, there was a boy. And now knowing a girl is running, a boy has finished, now the son of a brother is running medicine, he has a granddaughter, he is also walking, when he is three years old, he gets a visit.
R10- In some way, we are showing all the children again in A Hospital, so now we have one girl in it, the other three girls have been cured, a boy has also been cured.
R7- This doctor will cure you, may not come out
R7- As time is outdated if it is early then time will take less.
R7- As this is happening, she will quit the medicine
R7- As if not from the beginning
R7- The doctor can tell him which one to start
R7- I was talking about them as they are happening, the treatment is going on, the reports are being made every single day, it can tell that it means the beginning.
R7- If the doctor does not tell you any disease, then you will have to ask them forcefully, tell me the reason, Khali says it is okay, this is the problem, what does this doctor sir mean, they brush it off.
R5- Now i will go neither i will ask
R6- We will tell you again and then we will go ahead.
R8- We will tell you then we will further
R5- I had gone before to show my girl, it's been years, I was saying, how to eat medicine and what to say, when you will be proud, you may have it, your child may not survive and you are not left well
R8- That's why I'm taking hospital
R6- I am going to the hospital
R9- What should i say that's the matter
R6- Our boy had dengue, then he got a lot of treatment, he got cured when he did medicine
R7-Telling the daughter-in-law of this boy, get the whole treatment done here, with a satchel, you came and started crying with me, the aunt has said such a thing, I have said that you are crazy, you can do 100 fifty daily medicines It is not possible to do a blood test for 200 rupees, there will be a report on the blood test, what is the reason, what is the deficiency, what is the disease, immediately the tumor was removed, the aunt said if you do not say, then I do not know what. Gets, most fever is first blood test
R5- The fever did not stop.
R7- There was a lack of plasma, it was saying that aunt if you do not speak
R6- Is a medicine.
R9- Goat's milk, papaya milk was crushed.

R7- There was dengue my one eye vein is still inflated
R7- When someone went to the bathroom and picked up the father, it used to go like this.
R7- At that time, doctor, when someone lifts my hand and shouts, only then leave someone else, he also speaks fever with a broken bone.
R5- Yes, he goes with goat's milk.
R6- We also drank goat milk
R10- Yes, drink papaya's boils, and the medicine you bring home.
R7- The one who dead my boy, lacked plasma, I used to bring 20 rupees, the doctors used to treat me, but I used to do what people used to say to my comfort, that no one can get anywhere, in my house. If anyone has a fever already, then I do not carelessly because I advise everyone, or do not give medicine here and there, if the fever does not go in two to four days, show it to the doctor. I do not think that we have to cry like this next, I will tell everyone immediately, brother, do your treatment or do a blood test which shows what is the disease, get your son done, in two to four hours the report came that aunt For 10 days, he has been losing money, he has given his guess medicine, he had not given the medicine for that disease, who gave the paper, which medicine was said, when his record will come out, only if the blood is checked, then the doctor told him.
R7- Doctors will give medicine in it, avail will not give allergies too, doctor sir, and I do not know that while visiting the doctor ... I know a lot.
R7- The mother-in-law took two months, the leg was broken, it took two months, we had gas, I had lost so much medicine since childhood and ESI lost everything, referred me to G Hospital, she did not test me.
R7-Oh doctor sir, I told you to come with a towel, I took an ultra sound with a towel, I will wipe it with this, then I said, "Mother, I should be like this, but doctor, such an ultra sound is not there, then everyone says this." Test, now what will be done in AC alone doctor is giving a boiled pulse saying that there is tea ghoti (swallow), tears like sweat, when this test report came I got treatment in G Hospital for two years.
R7- One day, 6 x 7 x ray was done, my son is there, but there was nothing to do but nothing happened and the person who referred to the hospital got the report again, but from that he tested me with the telescope and it was found that water is also tea tea. Also, the PU feels as if the wound is full, the path is still moving and the stomach is moving like this, but the doctor is all right now, then someone happens, I advise everyone.
R5- You means the gas is gone
R7- If you get so much treatment, why won't you go
R5- So do our treatment too.
R7- Get GTB Cure That
R9- Did not go to the doctor
R5- We are walking on the medicine
R7-Doctor sir, I went to ESI, one day my pain took place, I was in my house, I came from ESI; Go, put 9 injections in a bottle, now tell me, I spoke to these unconscious people, my tongue means the voice is not breaking, they said to these people that I am speaking, you are listening to me, my voice is not Is it all inked People got up and took the ESI, the ESI person was not carrying it, he was saying, you have brought it to death, when the people who were taken from here yesterday said why not bring it yesterday, in an unconscious state, I got upside down, then I got the bathroom, When I went to the bathroom, the doctor came to my senses and took the same treatment in the ESI emergency, started the same treatment, if I did not go, it would have died, the ink was completely gone.
R9- Giving medicine on its own, mouth is drying up, mouth was completely dry.
R7- I didn't know anything
R7- I tell everyone in my neighborhood, and when something happens, I also come to you for advice.
R7- Doctor sir, I have not left any hospital, give an empty address, a woman came and was working, her hand is hurting, then the doctor injected her, now what was the injection, she got her full hand from here ..
R7-No, it did not swell, it started to dry completely, it did not turn like wood, it did not turn from such happiness, tell it was only pain the pain was injected with pain. Got chopped
R9- Got chopped

R7- Otherwise, what she would have done, she did not know what she had done, that it had happened, she had lost such a big hand.

6. In your view, what are the common causes of stillbirth and intrauterine death in your locality?
F- Then the same will come from your report, you will know from that what is the other thing, when the child is more serious then he goes to the hospital, then what does he do if the child has died in the hospital?
R7-Little children were safe for me, he started doing it all of a sudden, then I checked it and then people started saying what the upper air is, then what I did, then I checked him and I have a Reshma Hospital in it, I have a hospital for children. The doctors have gone there, here it is not, you should come during the day, I said there was no problem during the day, then from there I was picked up and moved to another hospital and there I was doing the same way, then backbone Took all the tests, what will the child know now
R7- There were three days
R7- The doctor did not tell me anything clear.
R7- My big boy finished all over the place, the doctor with ESI said that this is a test with a bone marrow, I also got him done for a month and a half from Mumbai and said that another test is in Chandigarh or aims, he was referred to AIMS , Said there, it will cost thirteen lakh rupees, I said doctor sir, I will give me thirteen lakh rupees, my child should be fine, I will sell the house, if the child is fine, then he would have eaten a boy of twenty three years, he must have known what is the problem. , The first doctor called me and said that ladies, you should make your heart stone, when can it happen, yet I have comforted my heart, O God, please give me a little comfort, be a woman to make so much heart stone keep my boy would have become a BCA course, he was not in luck.

We are trying to identify the causes of death in children and stillbirths, so that something can be done to prevent and reduce the number of deaths in children and stillbirths.

7. **In your view, would this information be valuable?** (Probe: If valuable/ useful, why? If not, why not?)

R7- Doctor, you will tell me this

8. **How autopsy/post-mortem is viewed by you and community members for knowing the cause of death?** (Probe: views, concerns and believes in the community regarding autopsy)

R7- Yes heard, as someone hanged, suddenly died, his post-mortem is done for what reason, he is killed by strangulation or killed by giving poison. , It comes in, this man is killed for this reason, when I saw him in the same careful India it shows all.
R- Do not see the doctor.
R7-I see both those careful India and crime petrol, I see both of them a lot.
R6- Watches till one o'clock in the night
R5- He finds out the reason.
R6-What is disease, after death, we see what happened due to deficiency, a disease is killed.
R7- I have felt what is the benefit of being persuaded, I have not left my child for a second, see if everything is done, my child will not come back by doing that, then I will not get it done, I immediately took it down.
R7- Why does the person tear things in the accident, what disease did it have, what happened to the accident, why do it?
R7- What the public there says, it says give a good response, take out the kidney, remove the eye remove the good things.
R7- People say, as the doctor says that he will have a kidney, he will have it, then he puts it, one life is gone, another life will be found.
R7- If I had listened, I would have told you, doctor, I thought I had not heard it.
R5-Doctors say that life goes on with a kidney.

R8- Yes, when those people say that they will be home
R7-There is one whose both kidneys were defective, then his father gave him a kidney, he said, I am old, how much my life is, well he gave it to the son after four months, he said if my child had survived Stays true
R5-Now I have a granddaughter no matter what happens to me, I am disturbed by cutting the rounds of the hospital, visiting her from the beginning then I take her to A Hospital on tour, also take S Hospital, show both places I am equal

In some instances, autopsy/ post-mortem is not possible due to various reasons. To find out the exact cause of death and underlying disease, some tissue biopsy (collection of very small sample using needles) and fluids (blood, urine, etc.) from the body of the child after they die, as done for investigation. But these samples are to be collected as soon as possible after death.

9. **How do you view minimal tissue sampling procedure as a method for knowing the cause of death?** (Probe: views, concerns and believes, religious aspects, comparison with autopsy)

R7- Will know that due to this disease
R8- What do we do, why did we let their body be destroyed, this is what the public thinks.
R3- The test which was a bone marrow, he said, "Go out, you will not be able to see that test."
R4- You tells me, it is not just about the woman but to see.
R7-A weak heart cannot bear
R5- Some like this weakness increases, many others say I will be able to see

10. **How the parents/families of a child/newborn who has died, should be approached by the hospital doctors/staffs for the minimal tissue sampling procedure?**

R2- Sir, we do not know, If they can explain well to the parents and family members, it may be accepted.
R3- I also agree.
R5- They should tell and assure no cutting and removal.
R4-The time taken should be less.
R8- may be the elders of the family assist in deciding.
