# Supplementary material for: Grief reaction and psychosocial impacts of child death and stillbirth on bereaved North Indian parents: A qualitative study
Source: PLoS One. 2021 Jan 27;16(1):e0240270. doi: 10.1371/journal.pone.0240270 (PMC7840017; doi:10.1371/journal.pone.0240270)

ID-12401

FGD-Fathers 1

Q.1

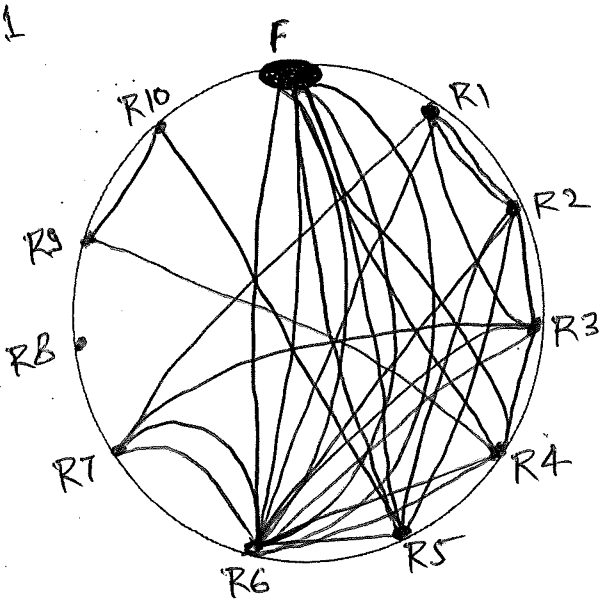

Q.2

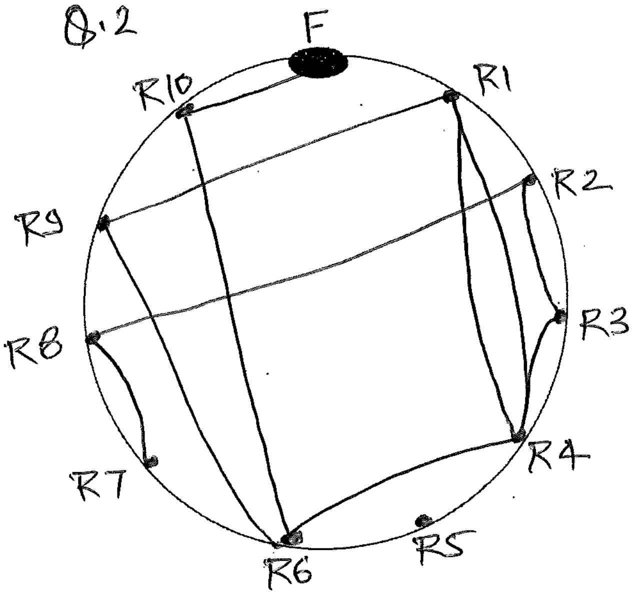

Q.3

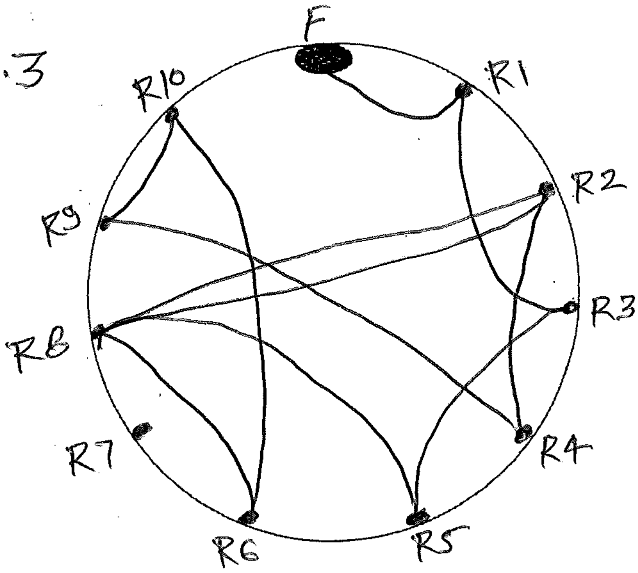

Q.4

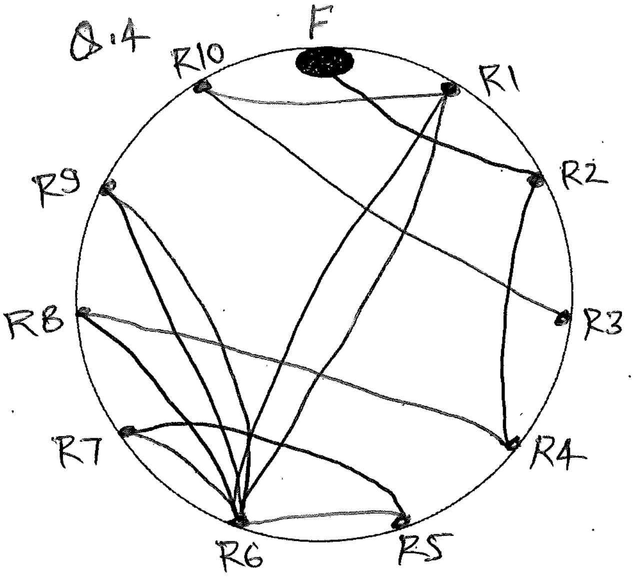

Q.5

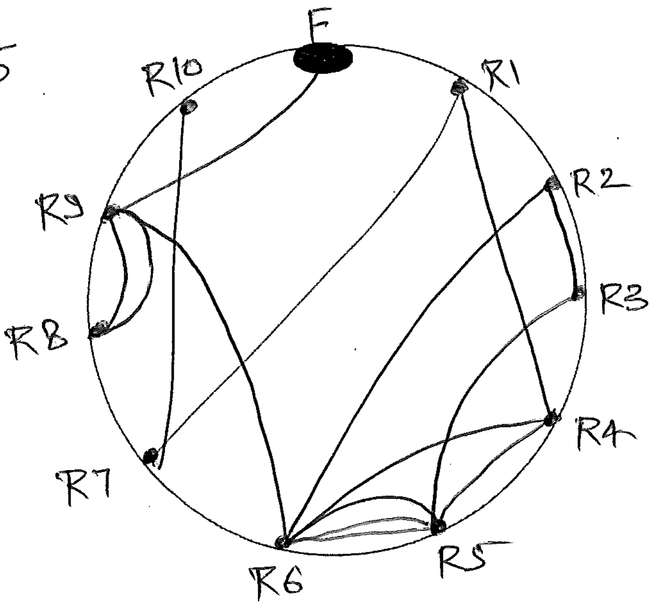

Q.6

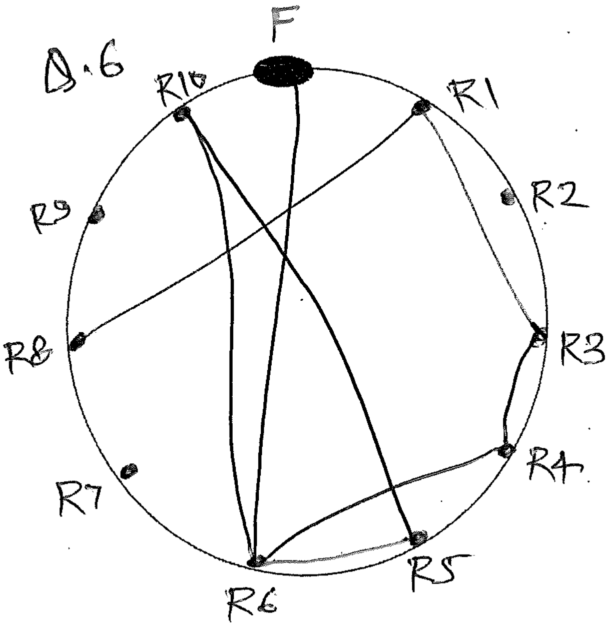

ID-12401
FGD-fathers L

Q.7

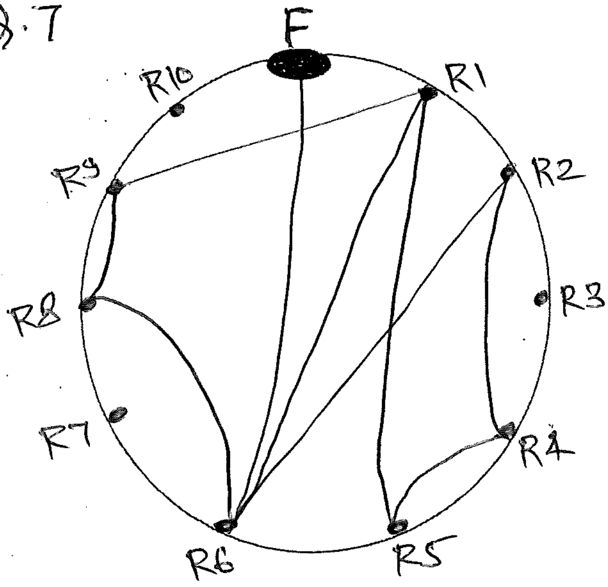

Q.8

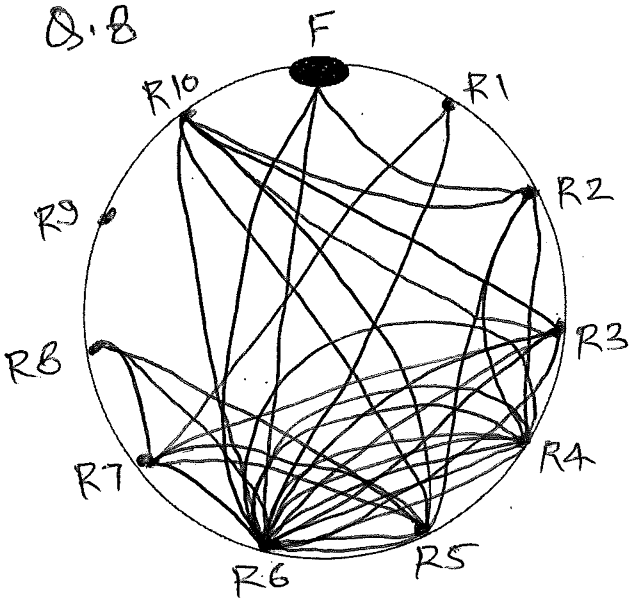

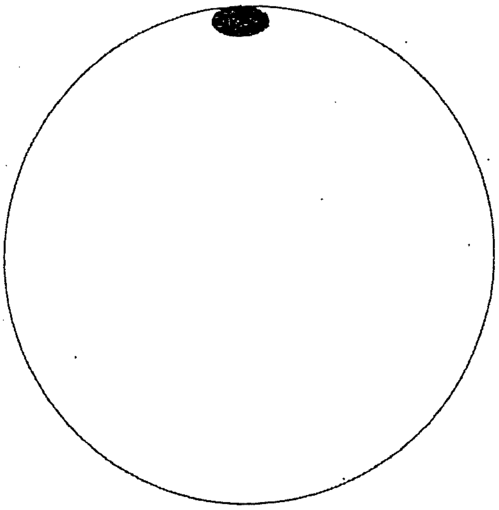

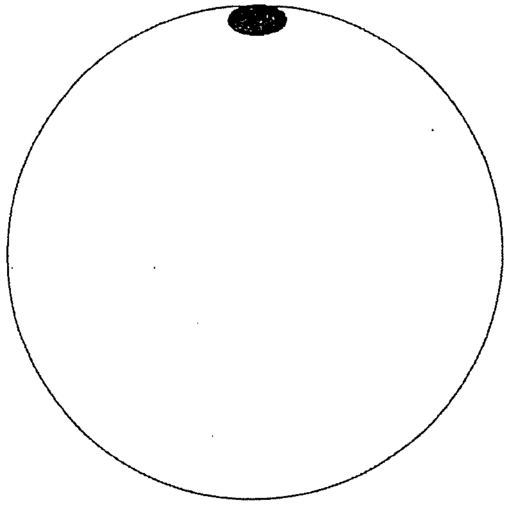

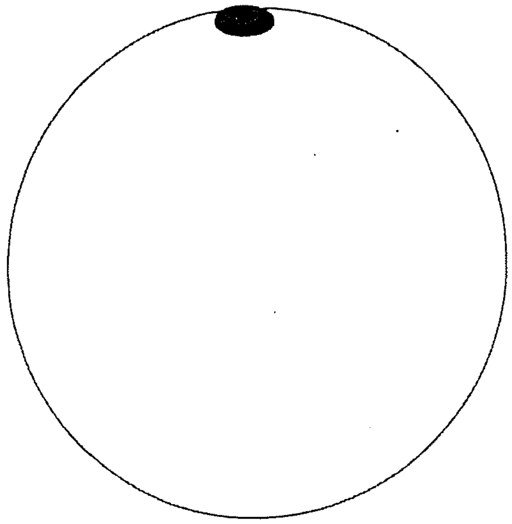

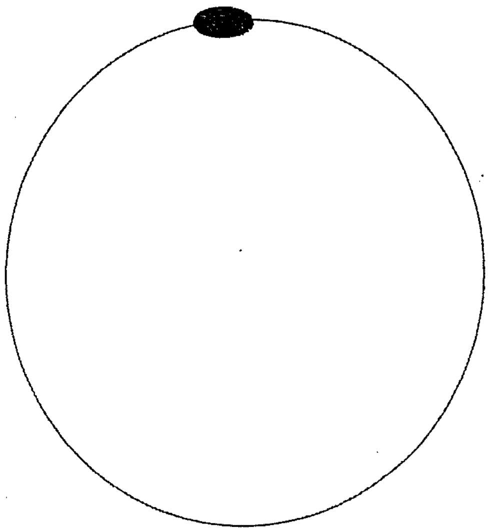

ID- 12402

FGD- Fathers 2

Q.1

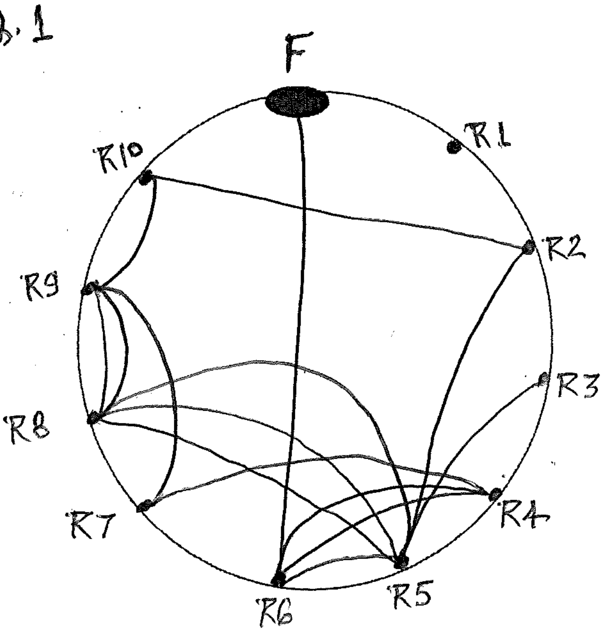

Q.2

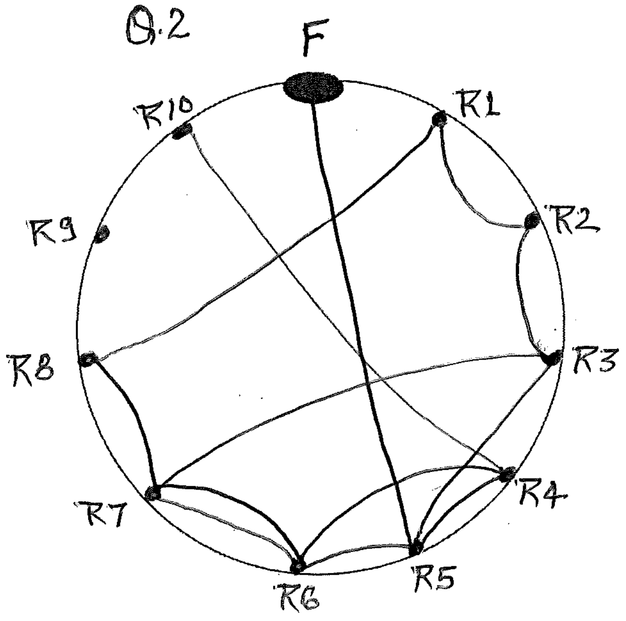

Q.3

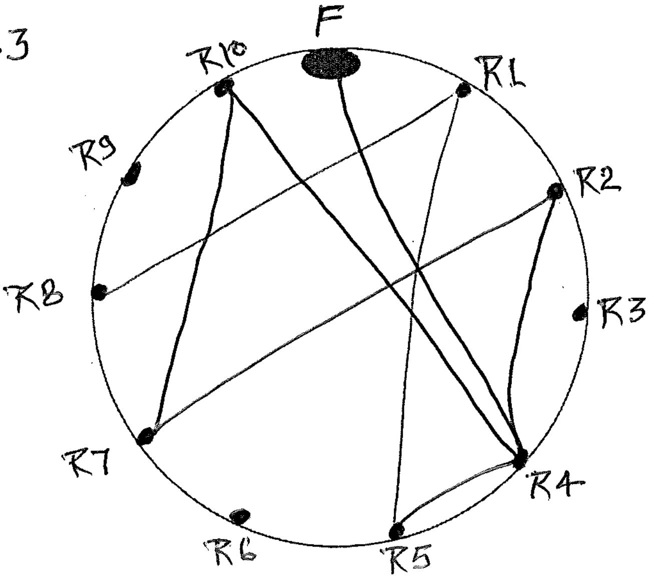

Q.4

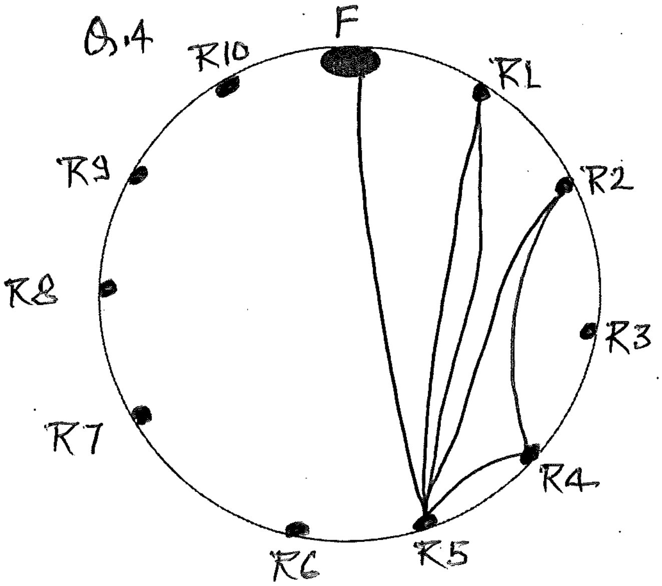

Q.5

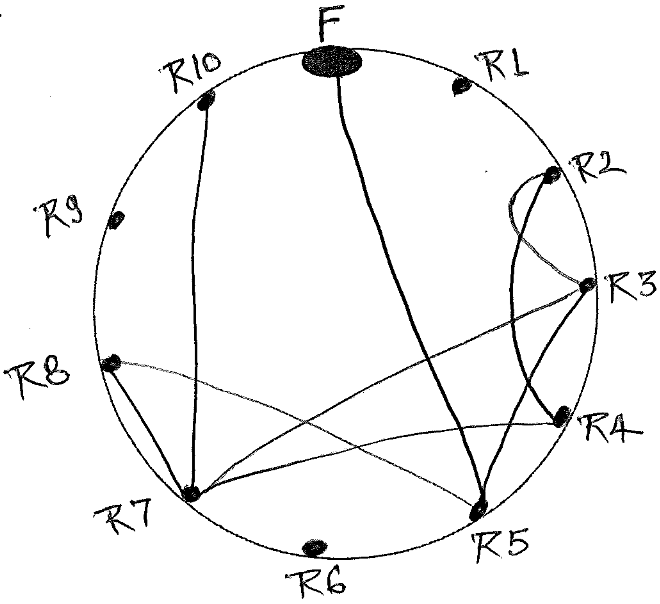

Q.6

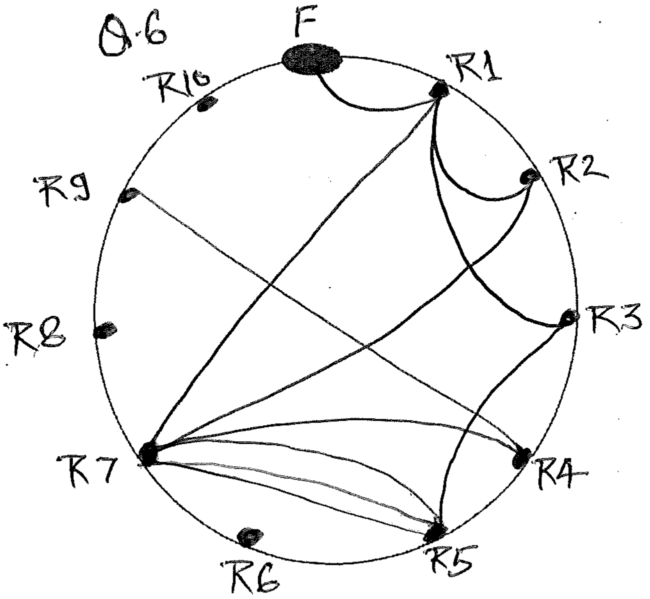

ID-12402

FGD-Fathers-2

Q.7

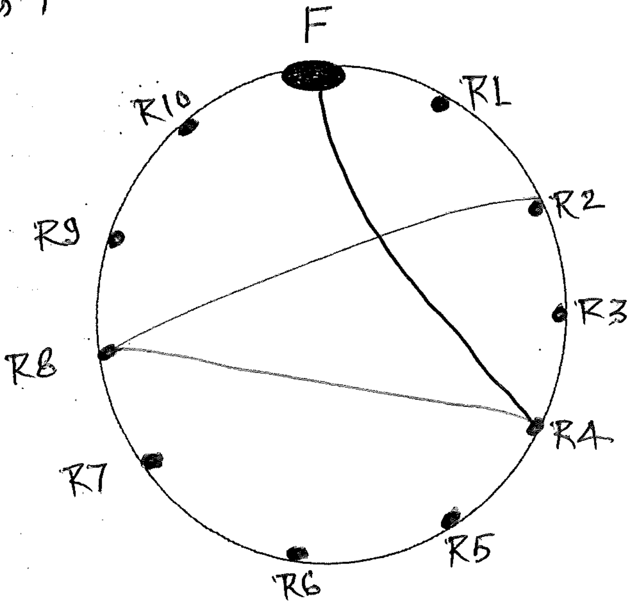

Q.8

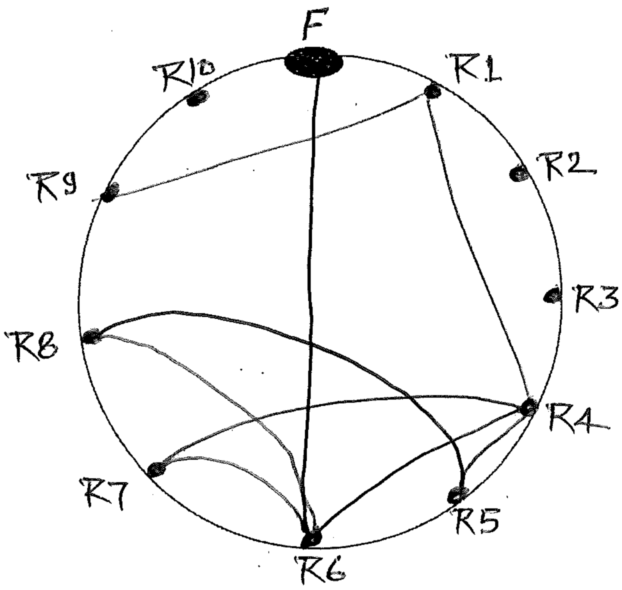

Q.9

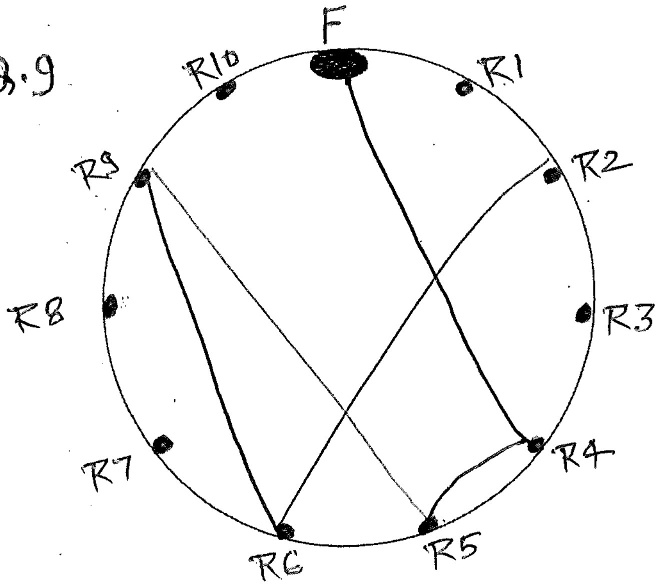

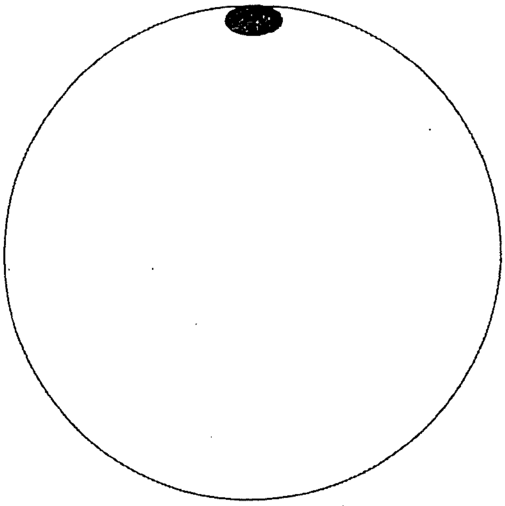

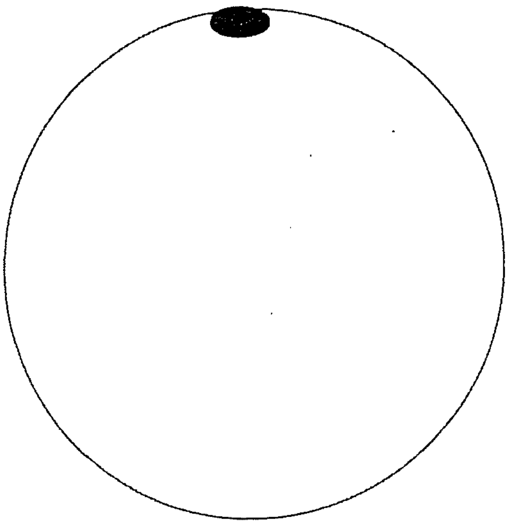

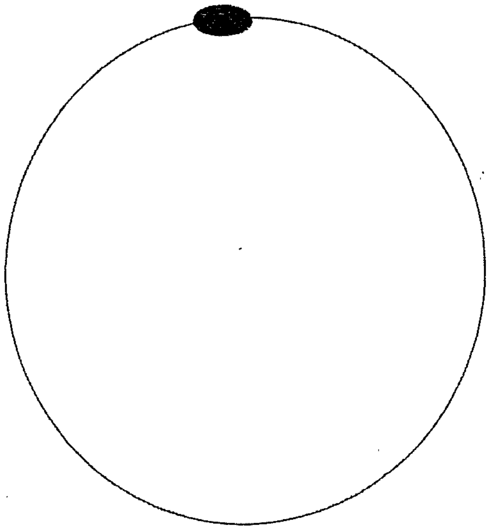

ID - 12403
FGD - Mothers 1

Q.1

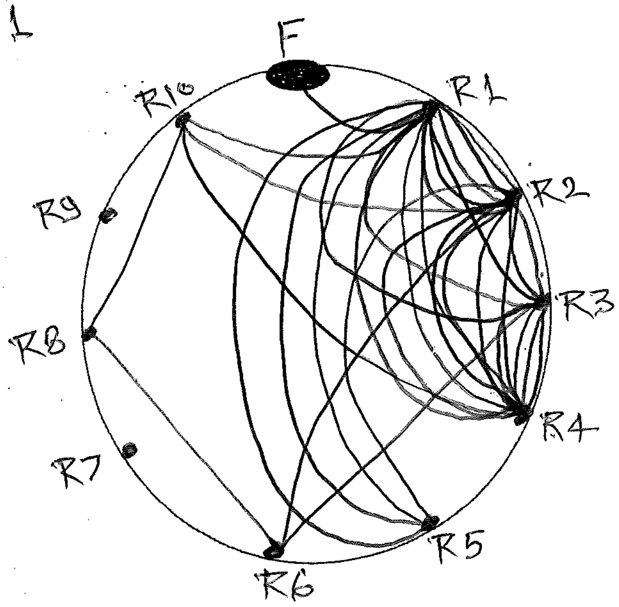

Q.2

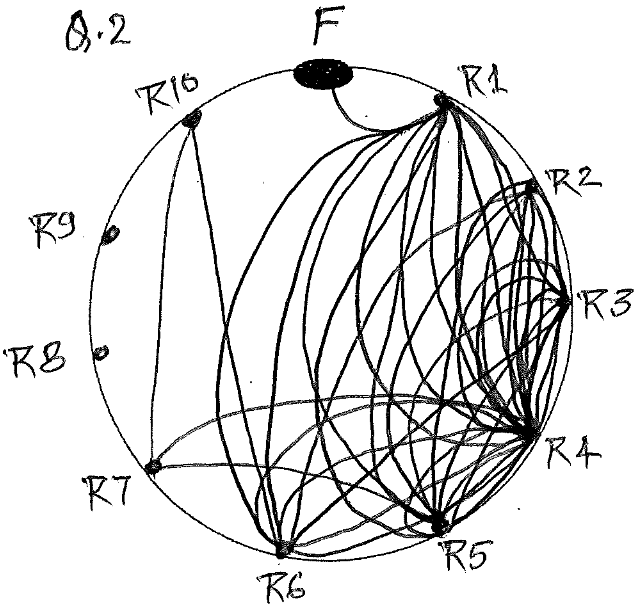

Q.3

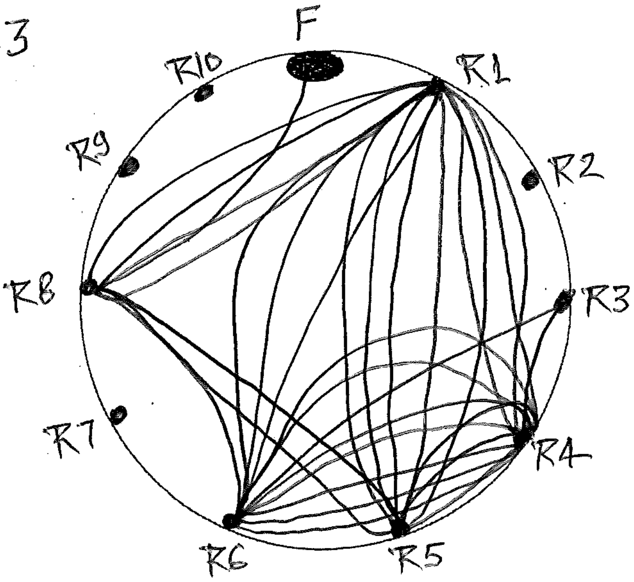

Q.4

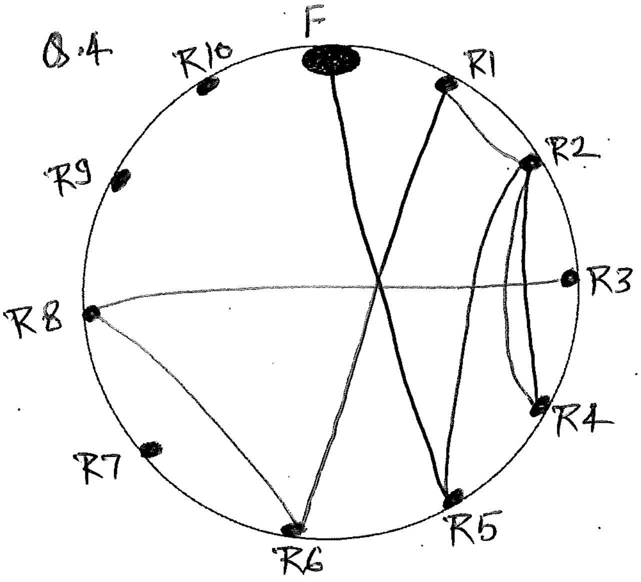

Q.5

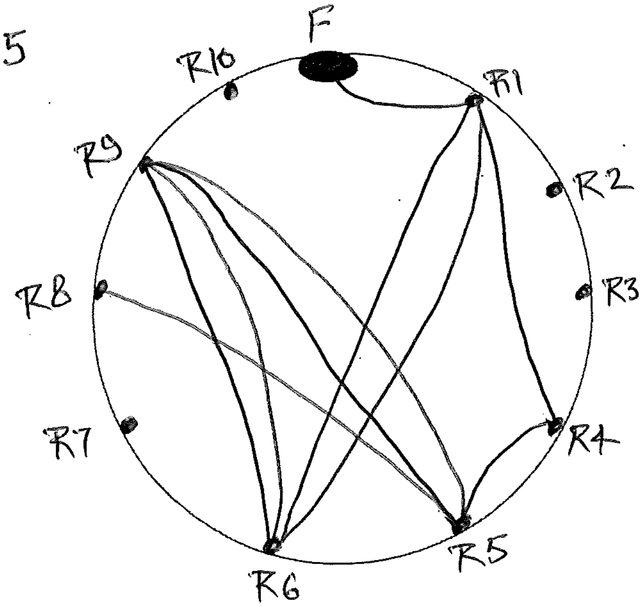

Q.6

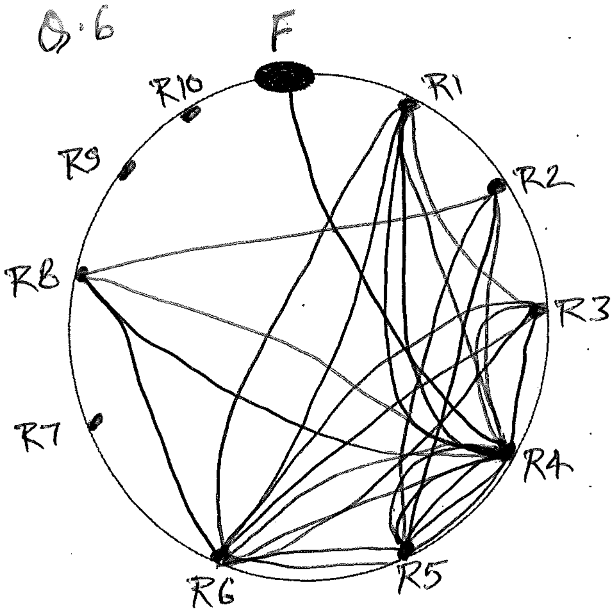

ID - 12403
FGD - Mothers - 1

Q.7

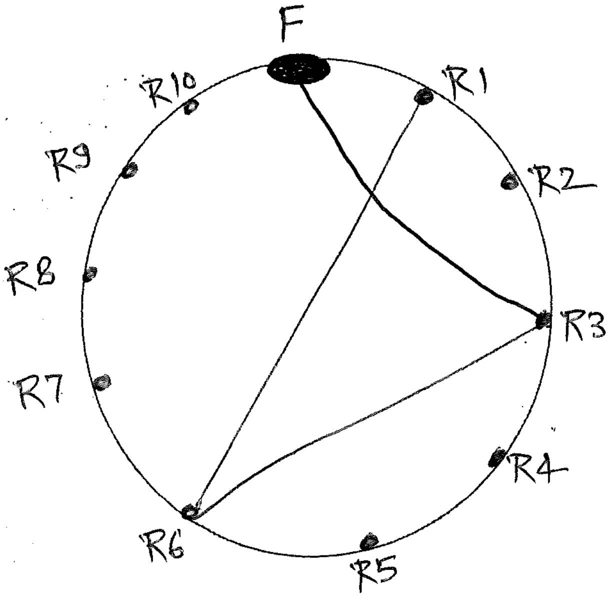

Q.8

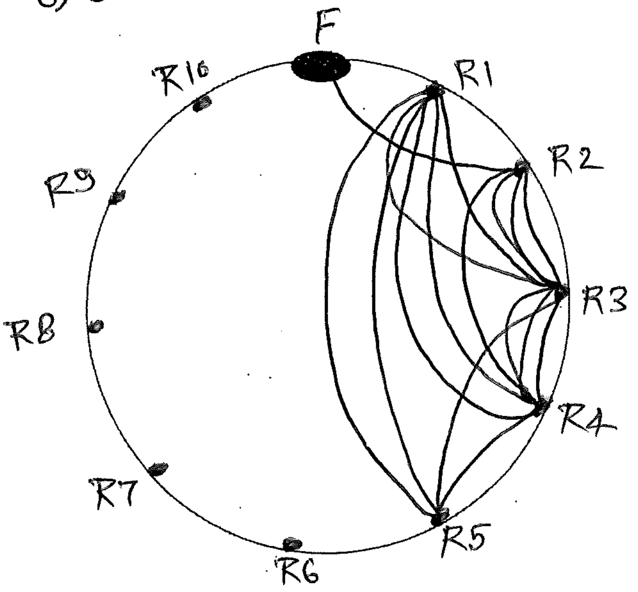

Q.9

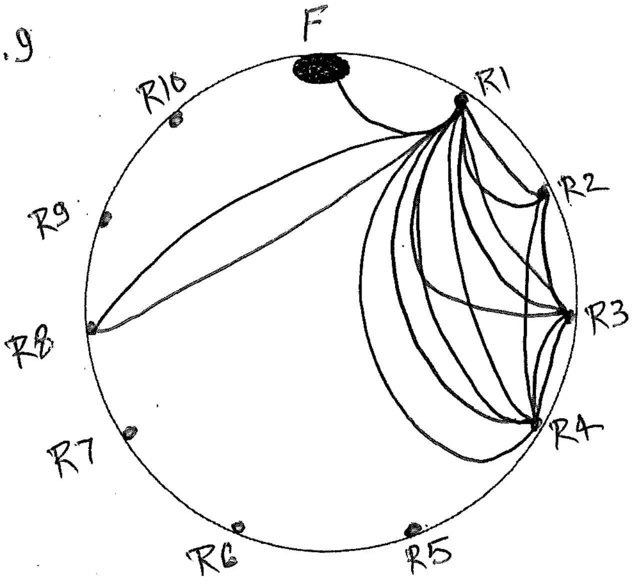

Q.10

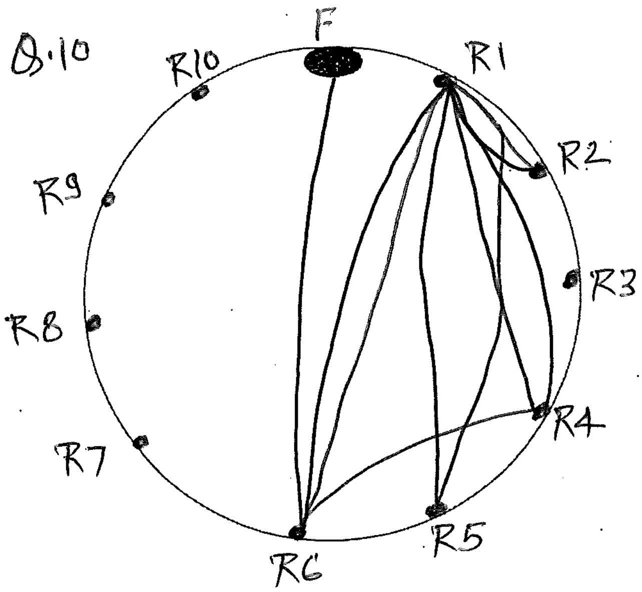

Q.11

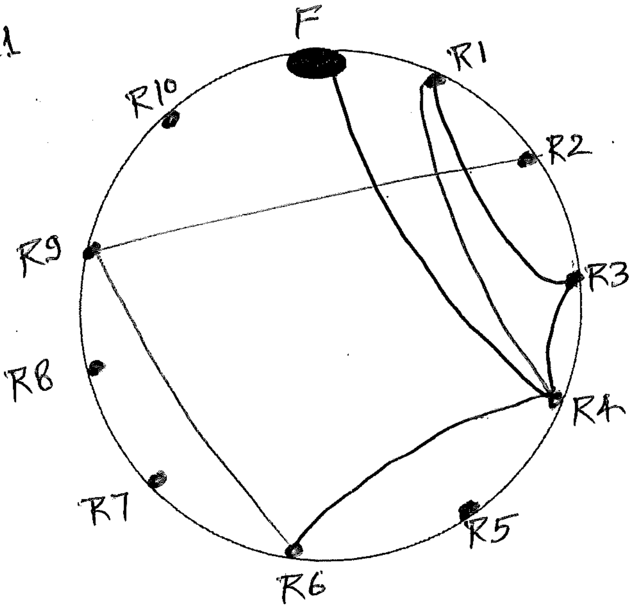

Q.12

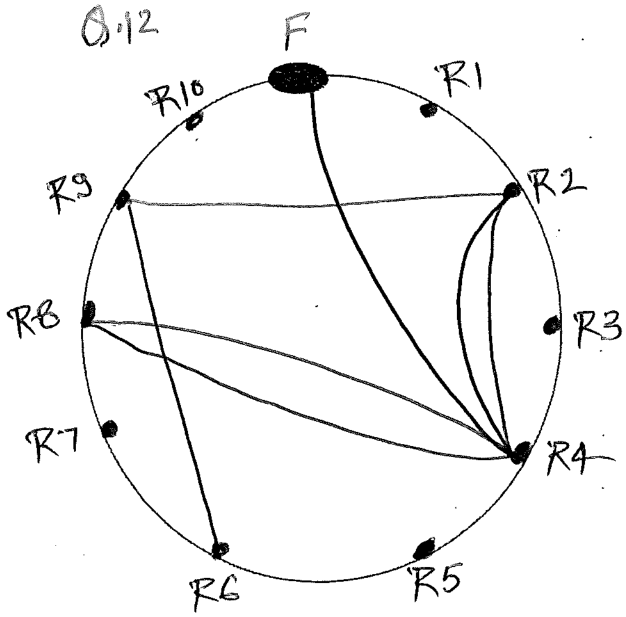

ID-12404
FGD-Mothers 2

Q.1

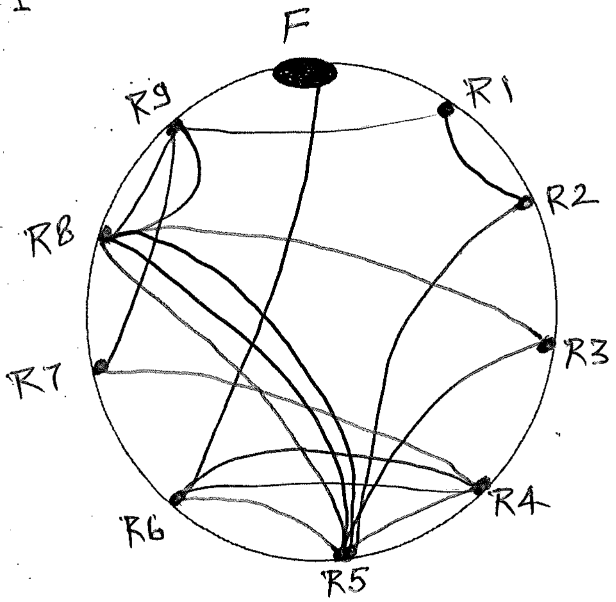

Q.2

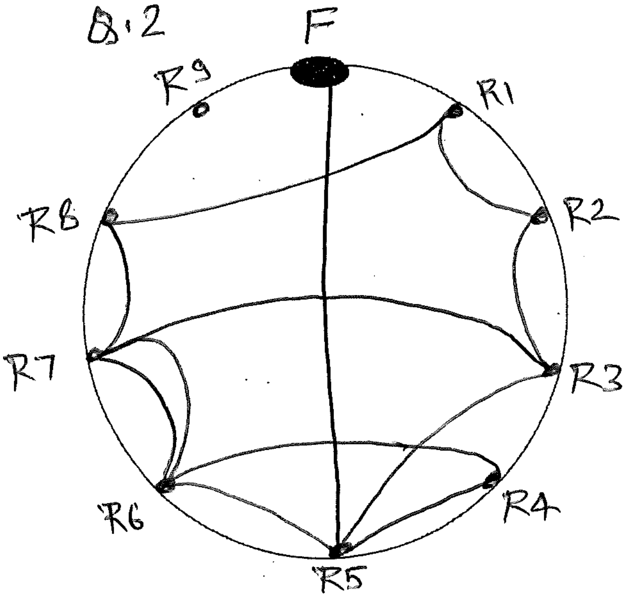

Q.3

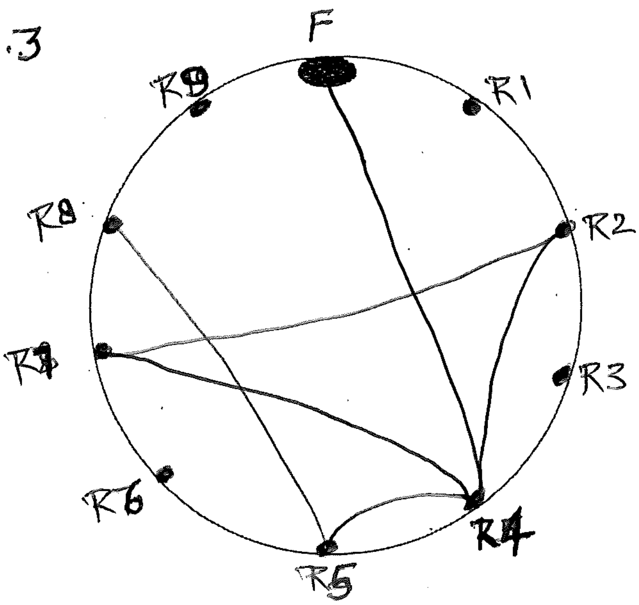

Q.4

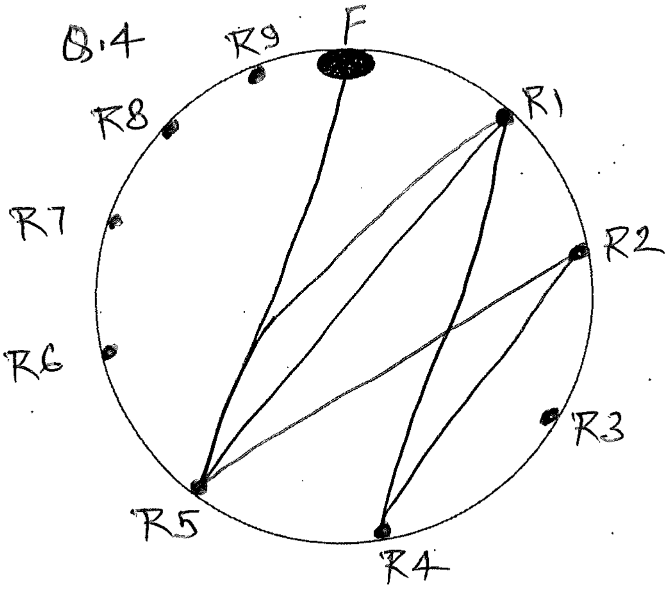

Q.5

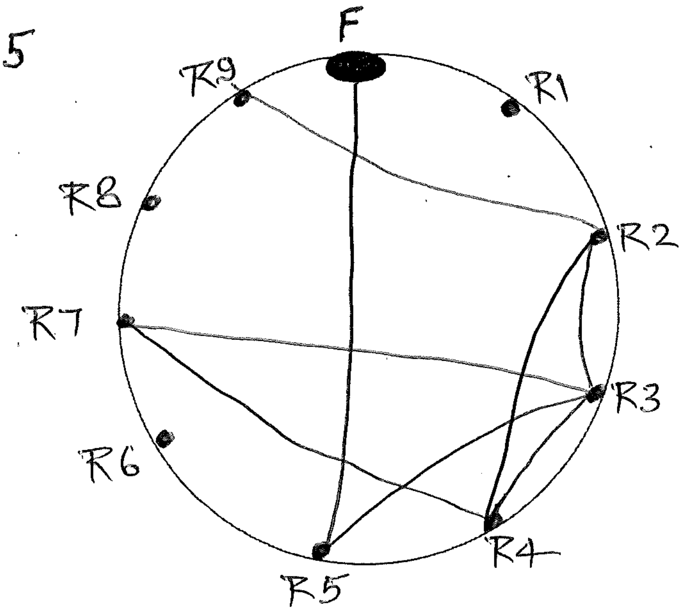

Q.6

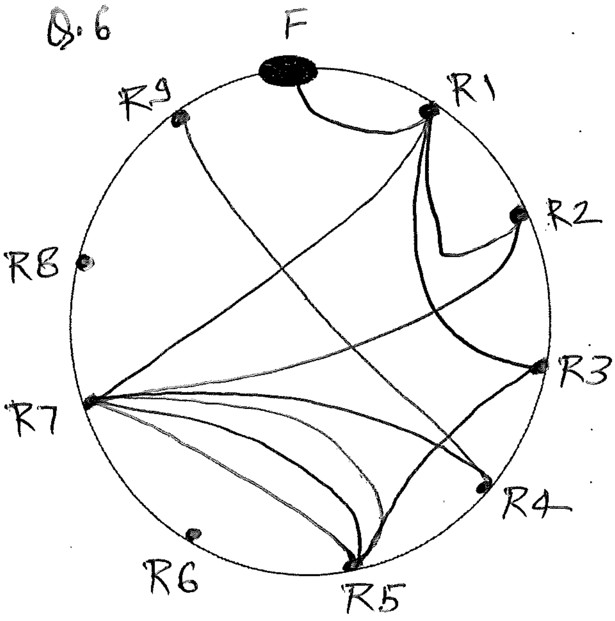

ID-12404
FGD-Mothers 2

Q.7

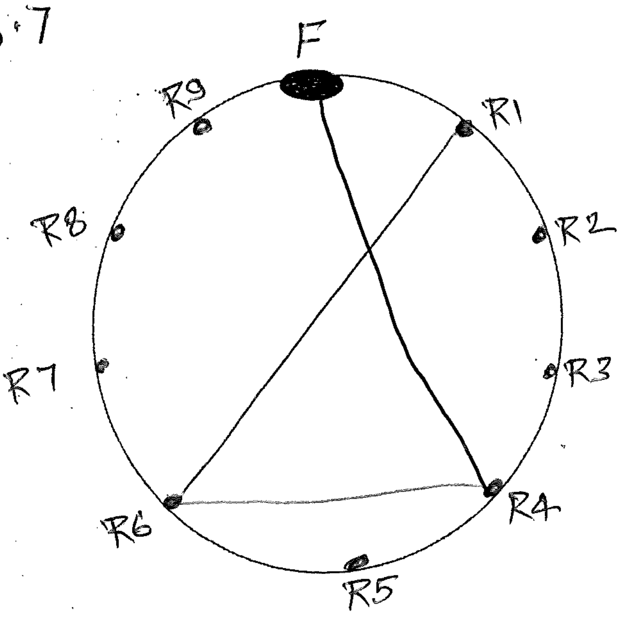

Q.8

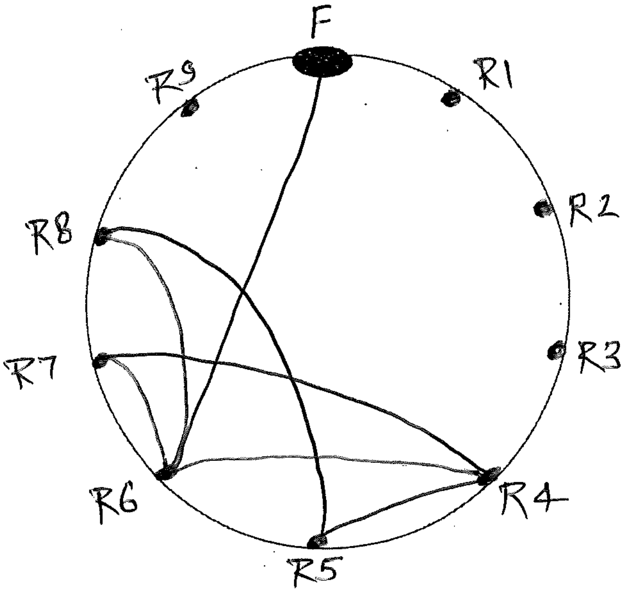

Q.9

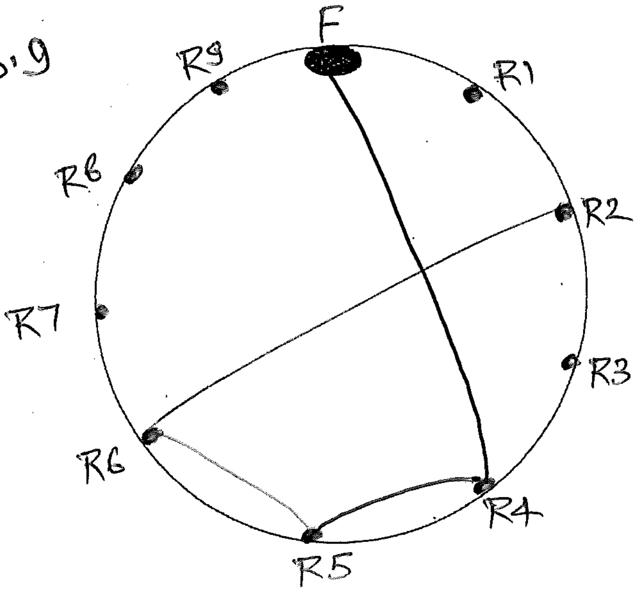

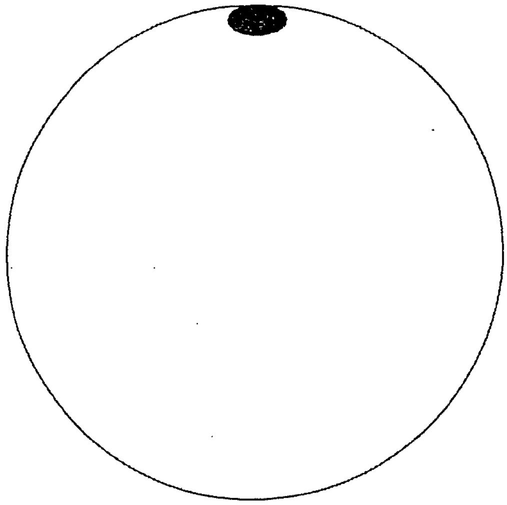

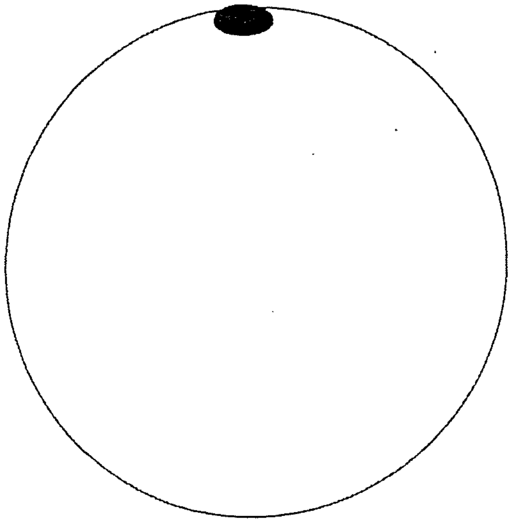

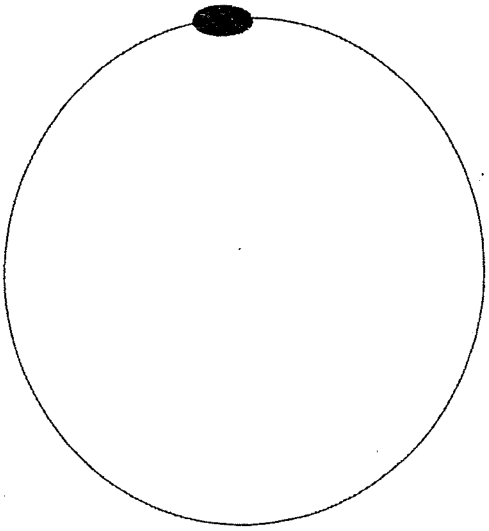

ID- 12405

FGD- Grandfathers 1

Q.1

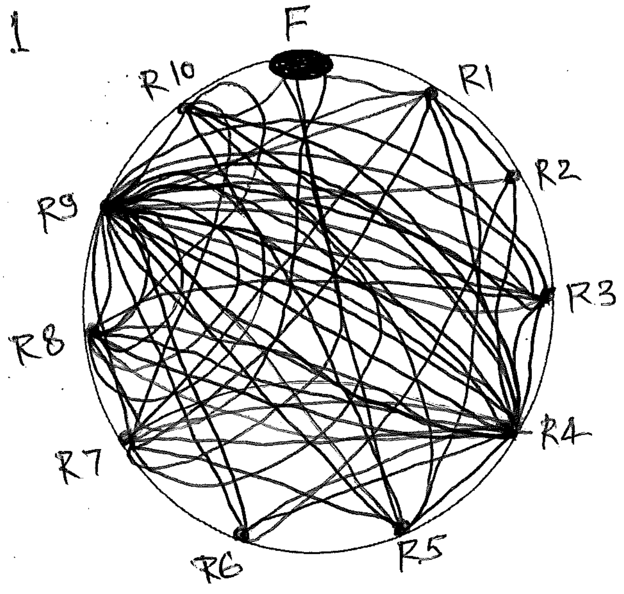

Q.2

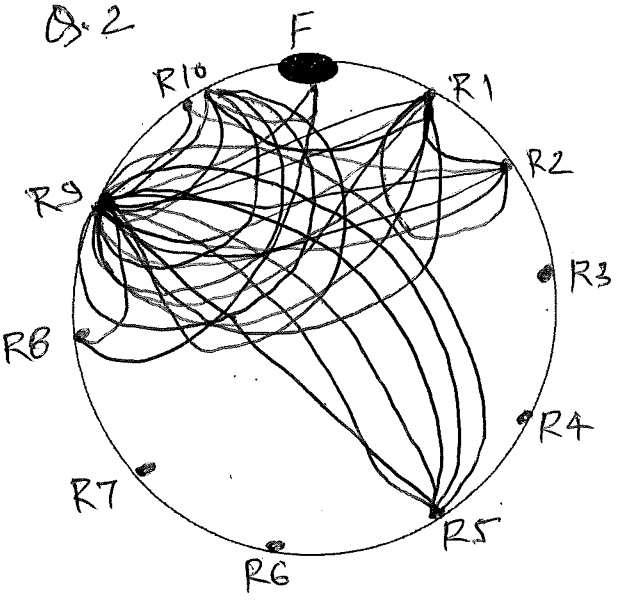

Q.3

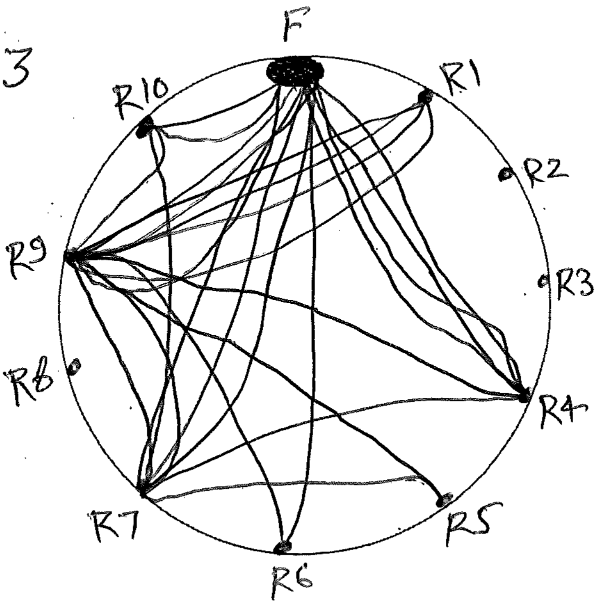

Q.4

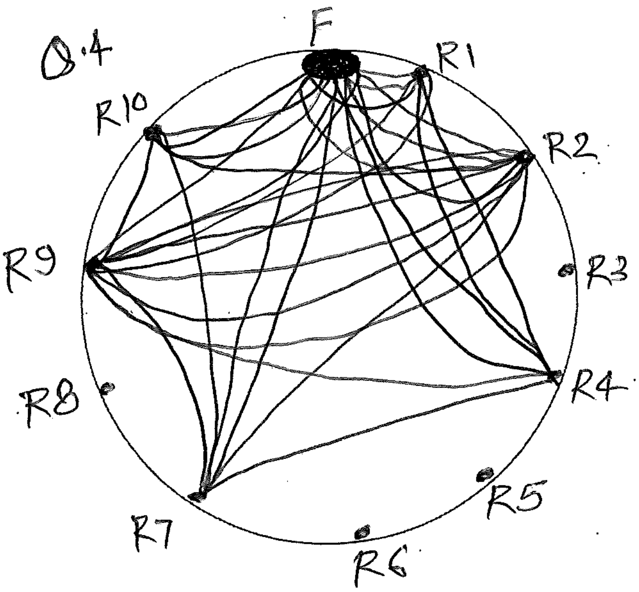

Q.5

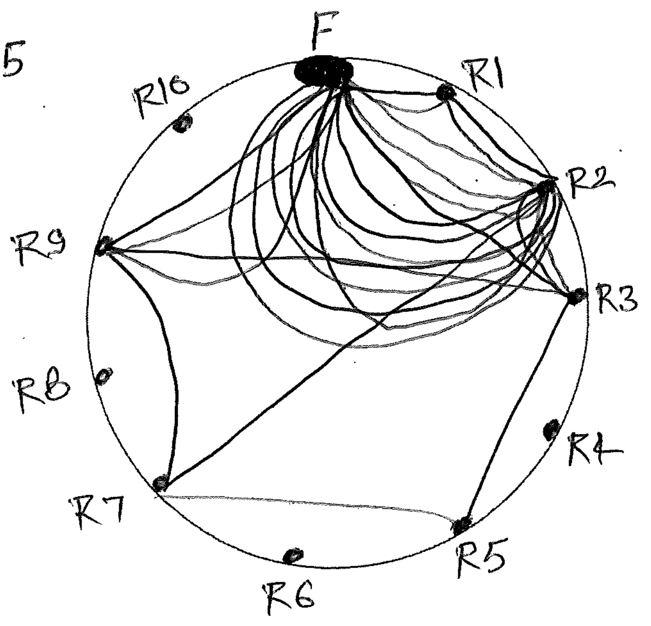

Q.7

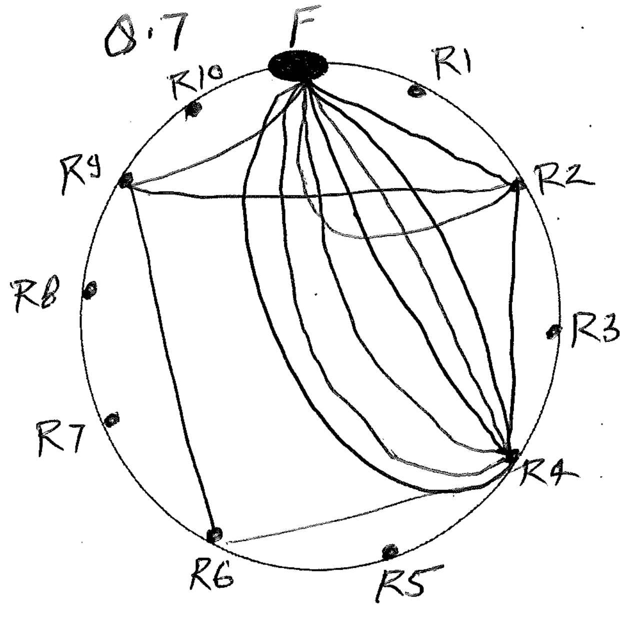

ID-12405
FGD-Grandfathers 1

Q.8

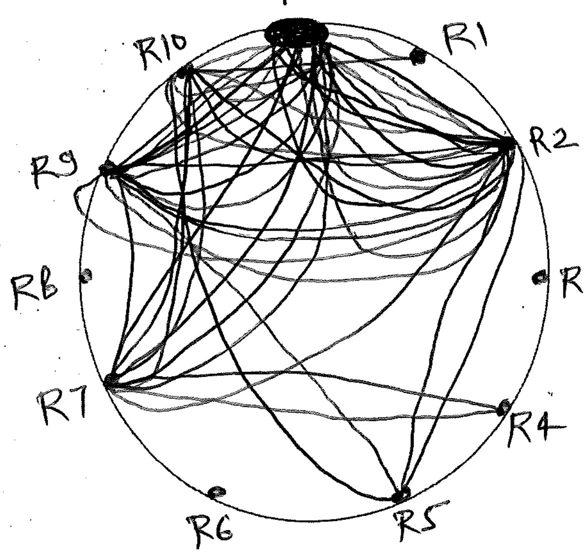

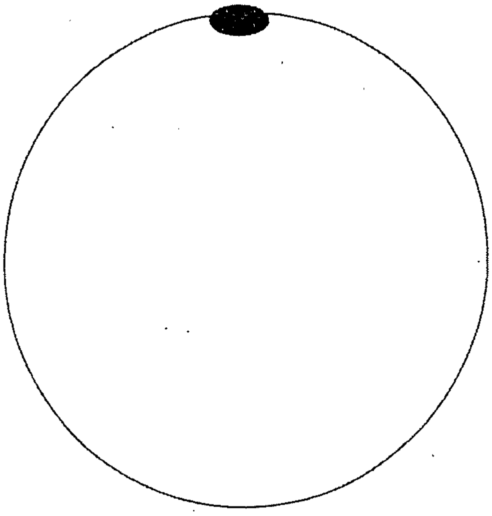

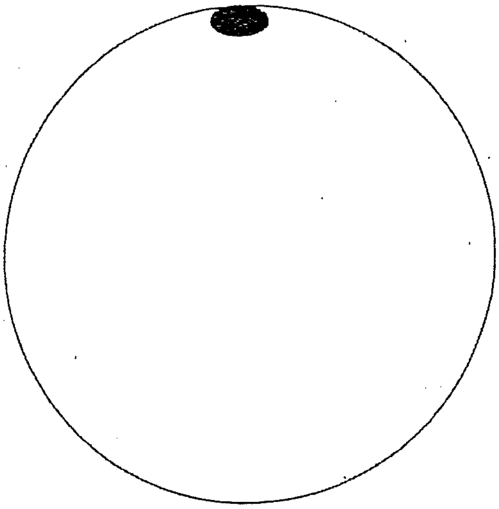

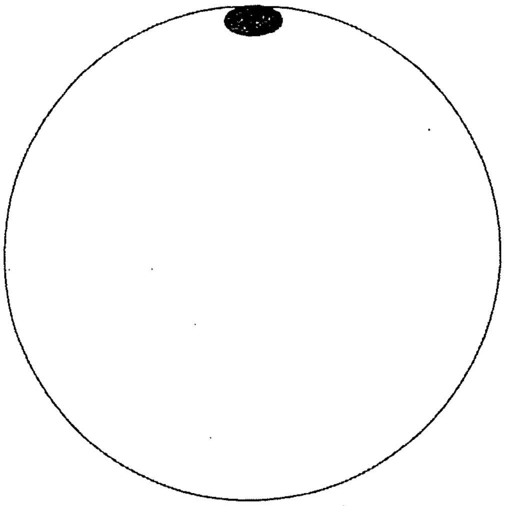

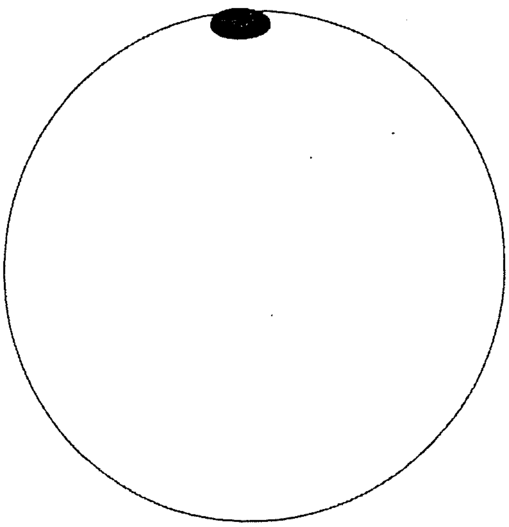

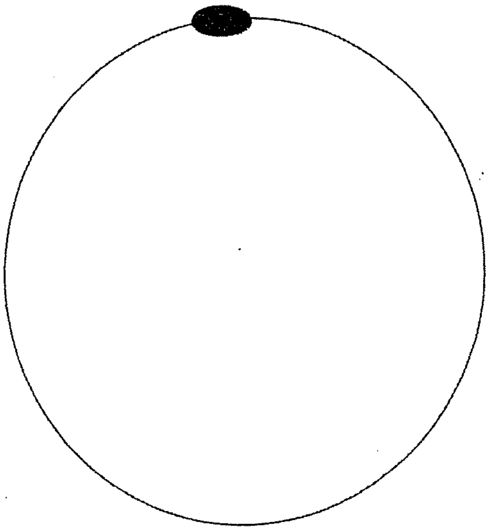

ID-12406

FGD-Grandfathers 2

Q.1

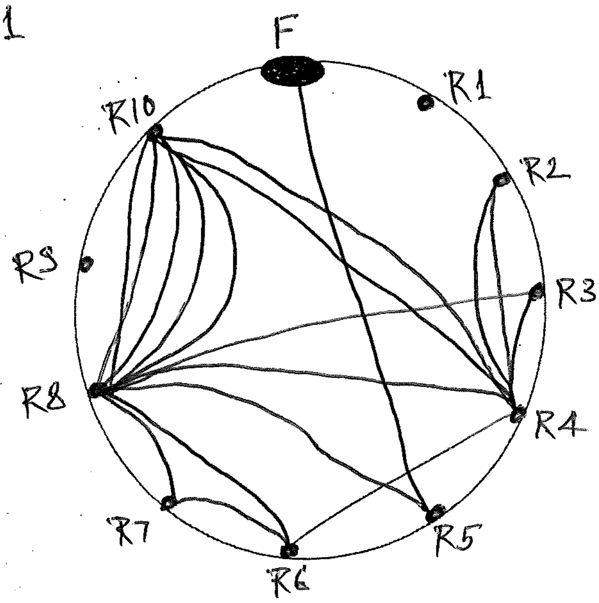

Q.2

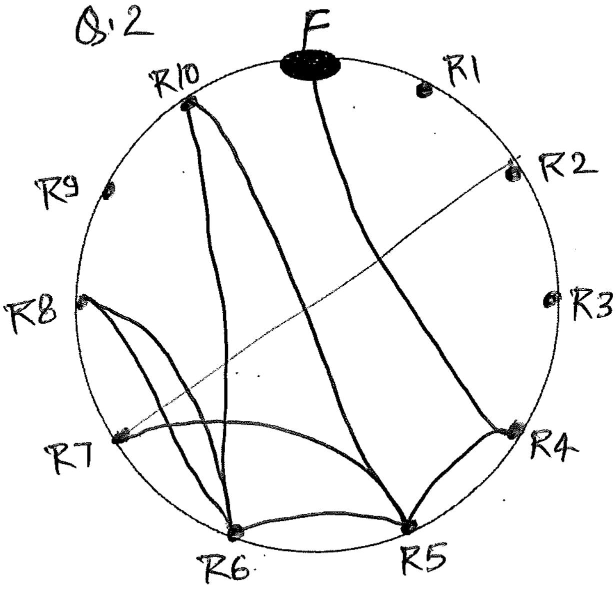

Q.3

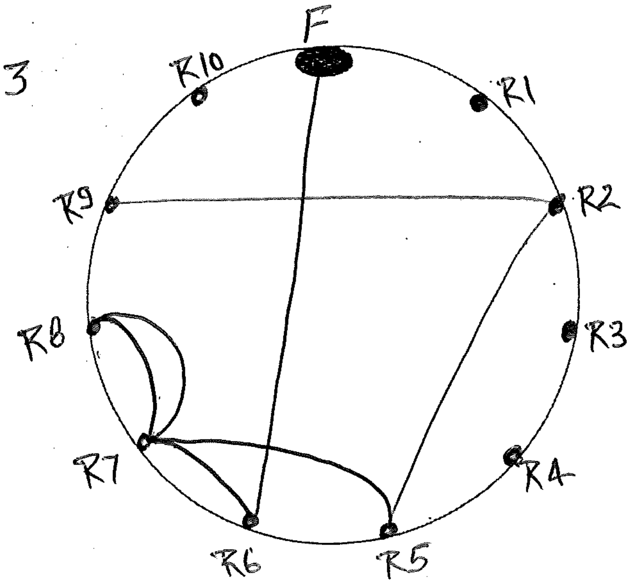

Q.4

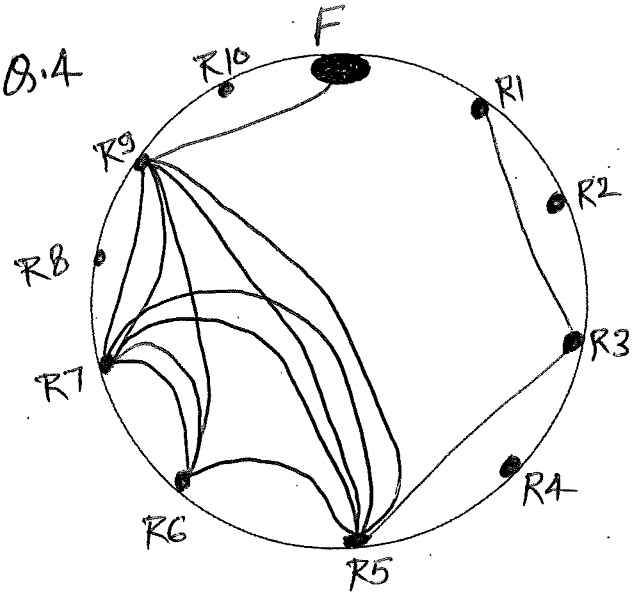

Q.5

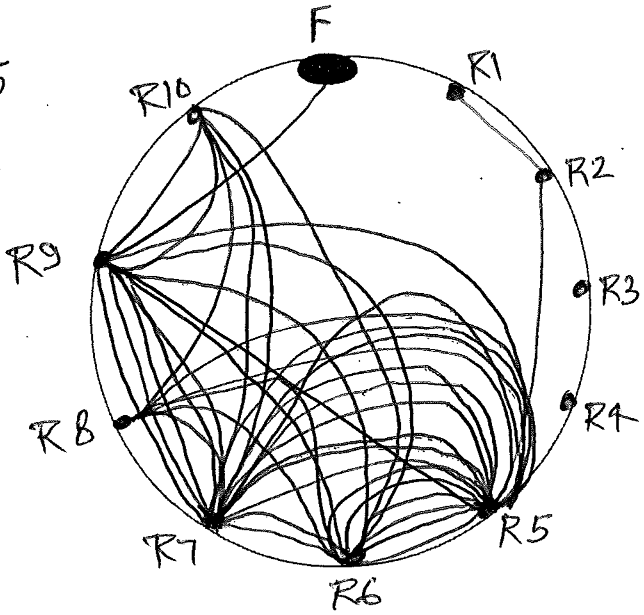

Q.6

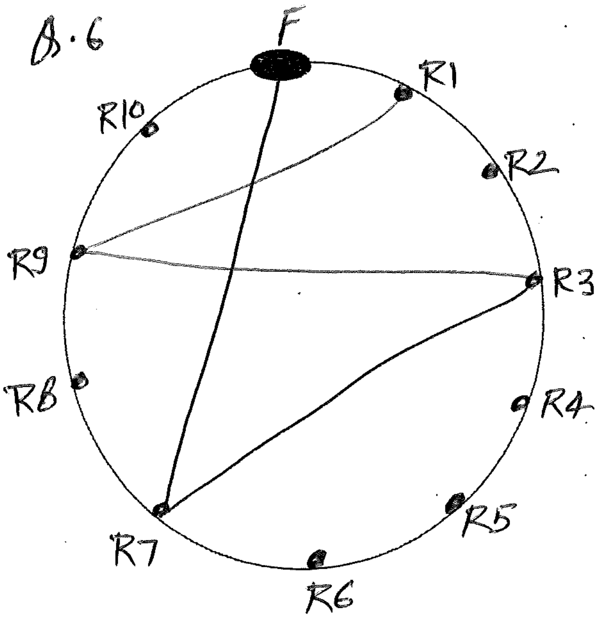

ID-12406

FGD - Grandfathers 2

Q.7

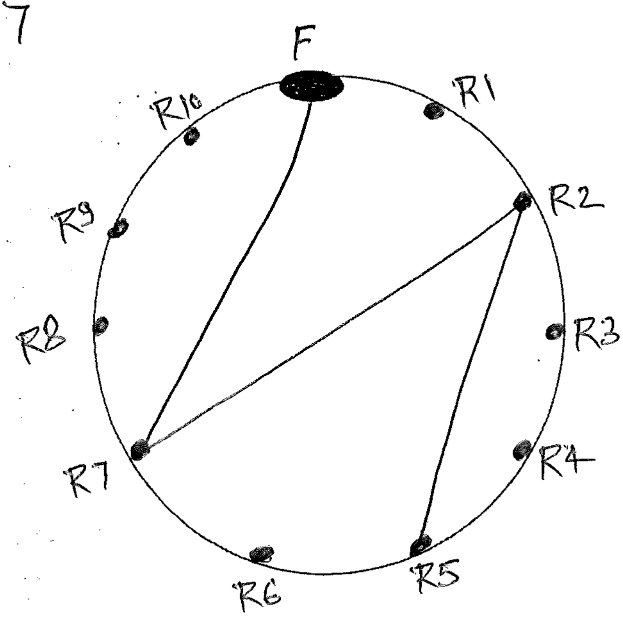

Q.8

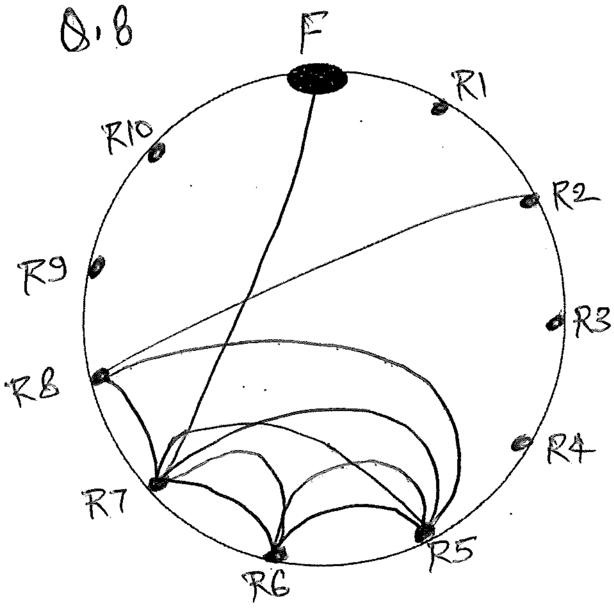

Q.9

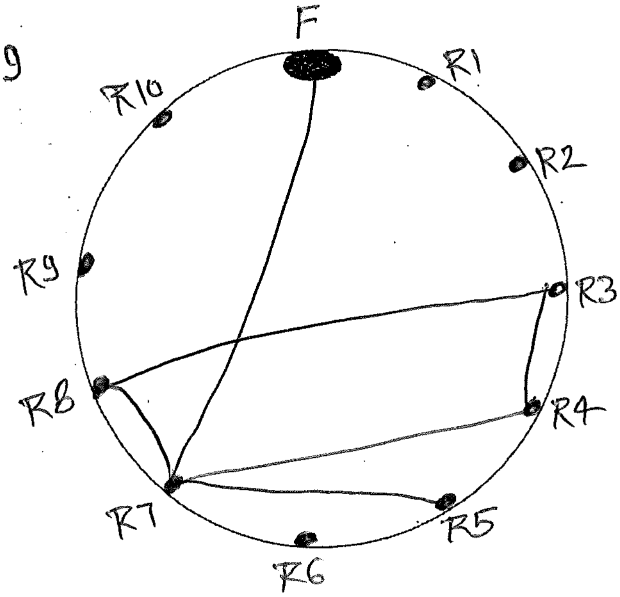

Q.10

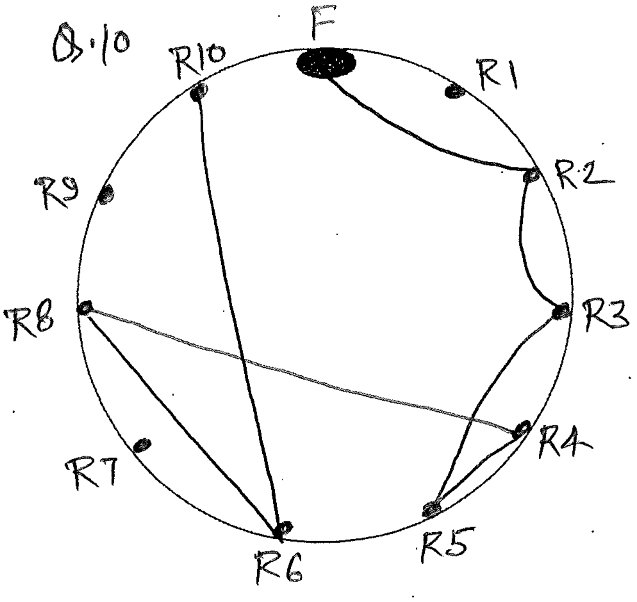

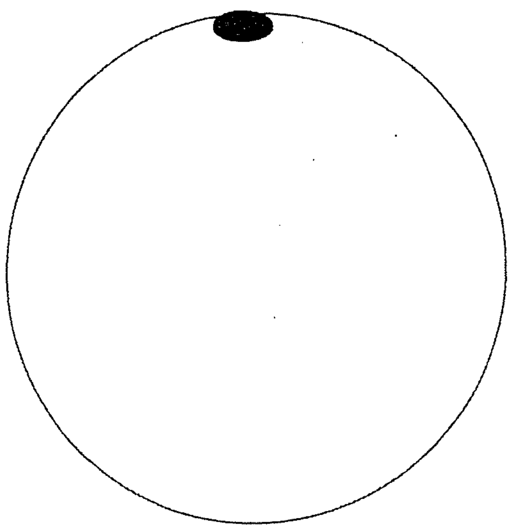

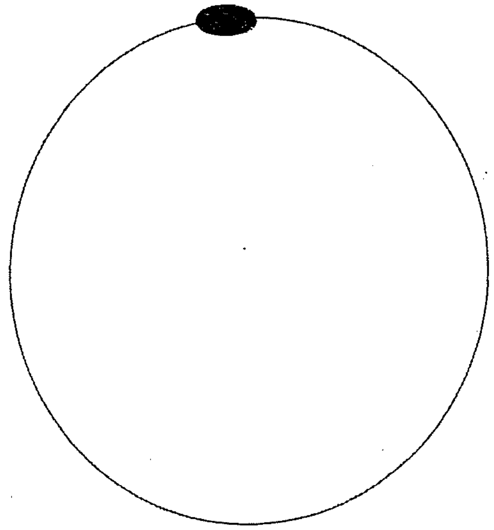

ID - 12407

FGD - Grandmothers 1

Q.1

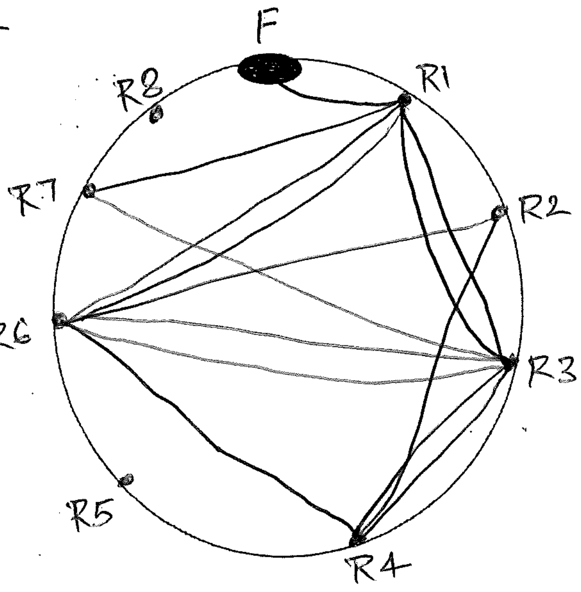

Q.2

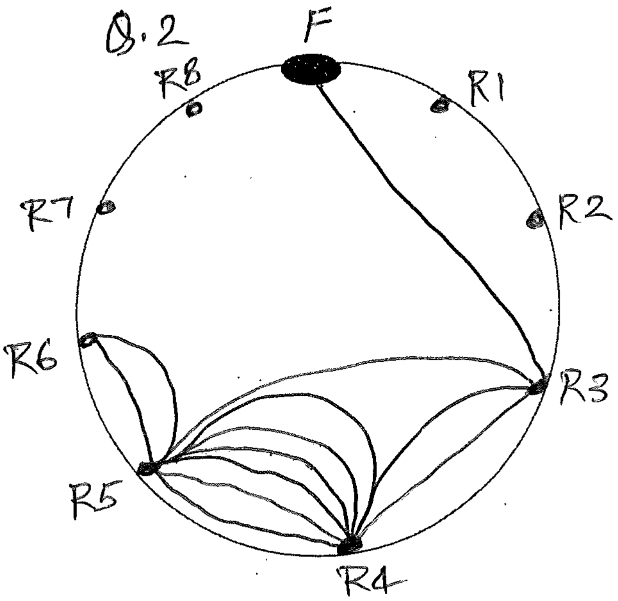

Q.3

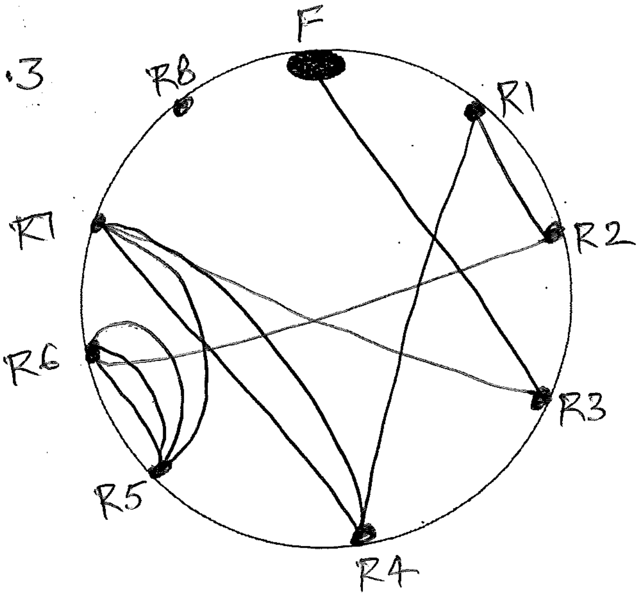

Q.5

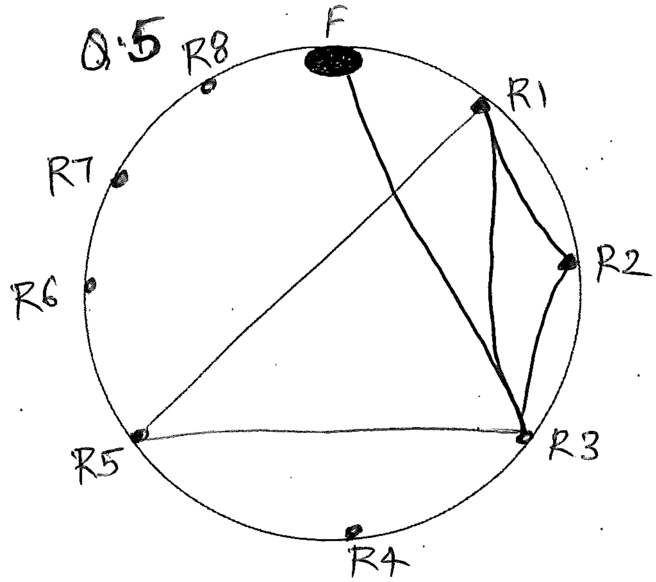

Q.6

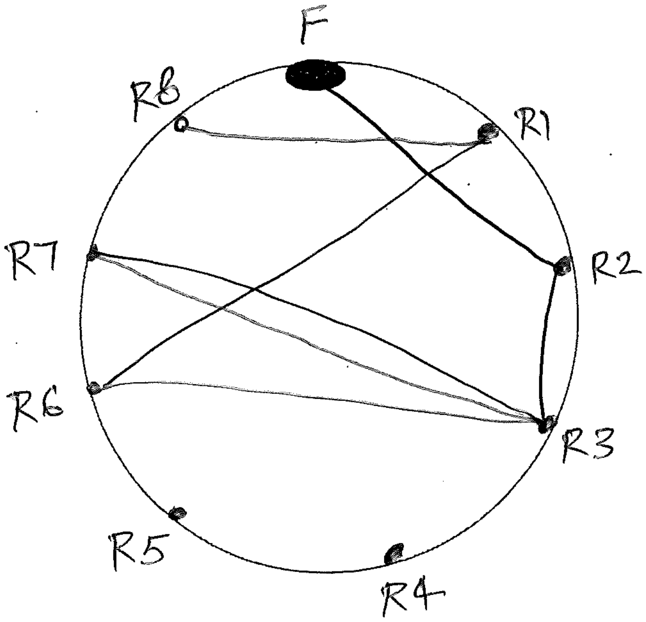

Q.11

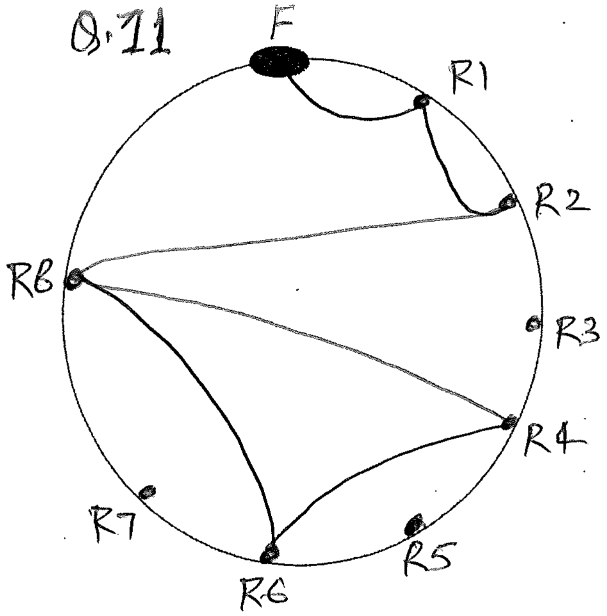

ID-12408

FGA- Grandmothers 2

Q.1

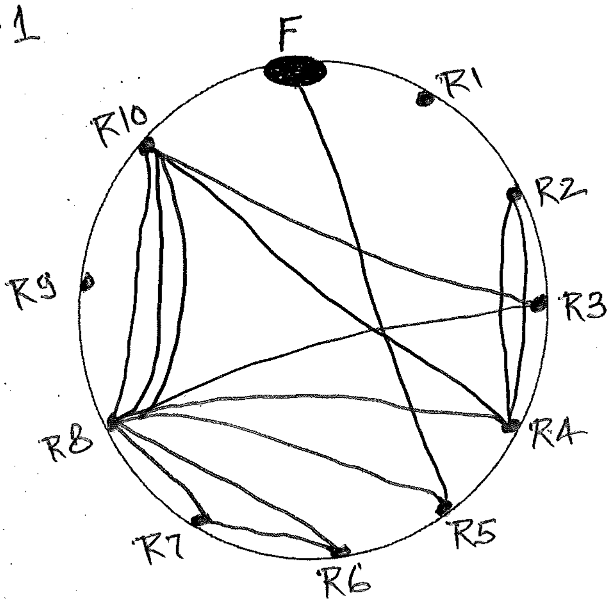

Q.2

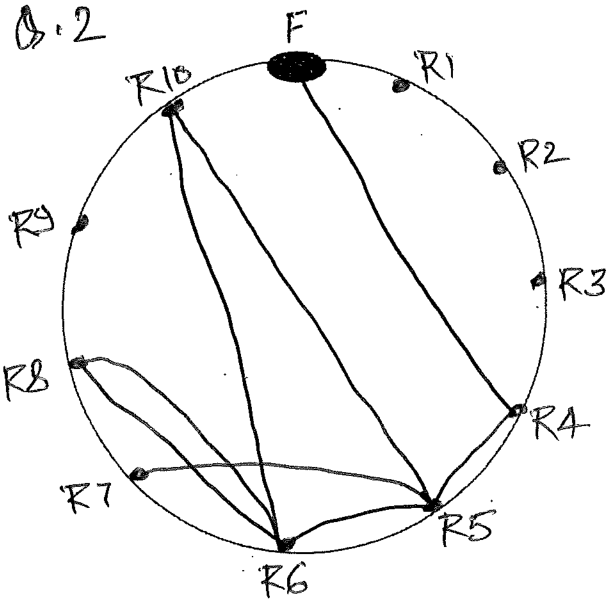

Q.3

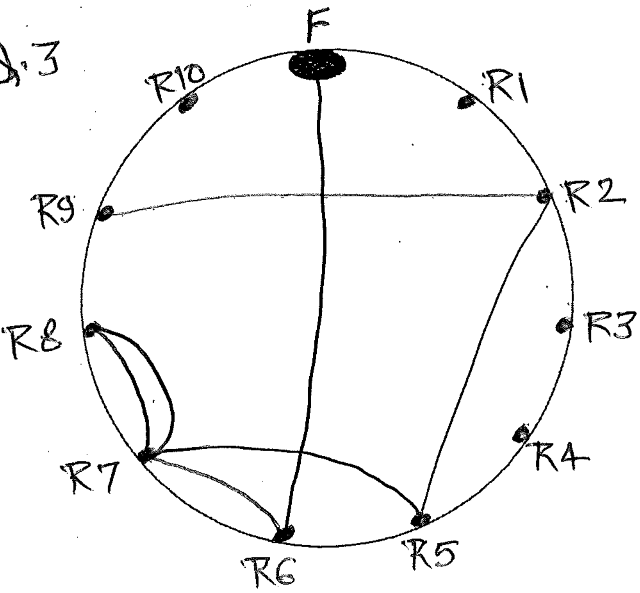

Q.4

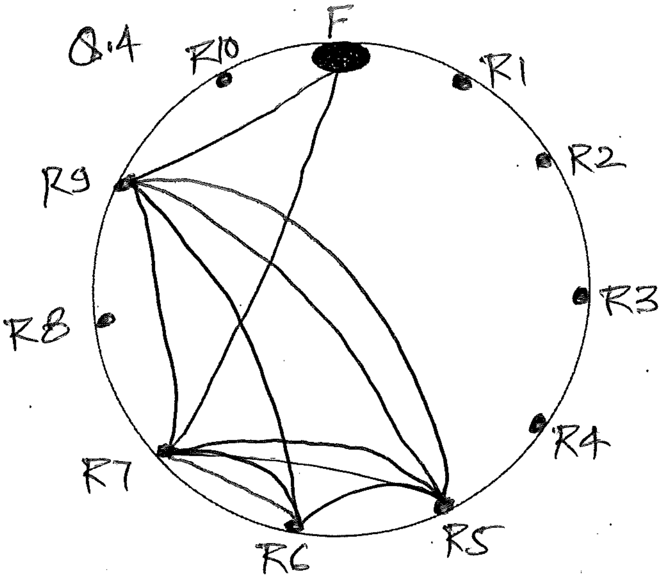

Q.5

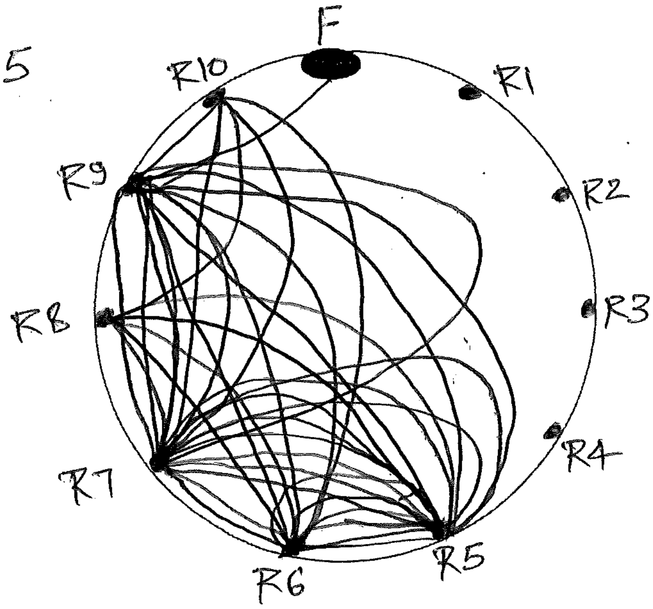

Q.6

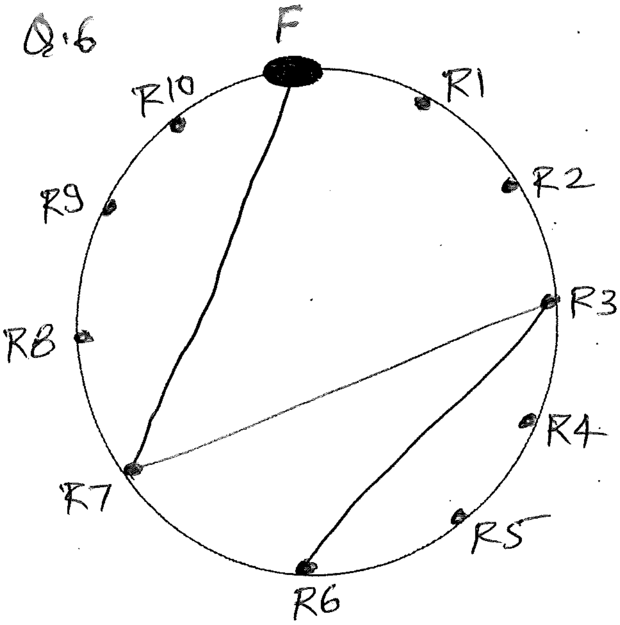

ID- 12408

FGD- Grand Mothers 2

Q.7

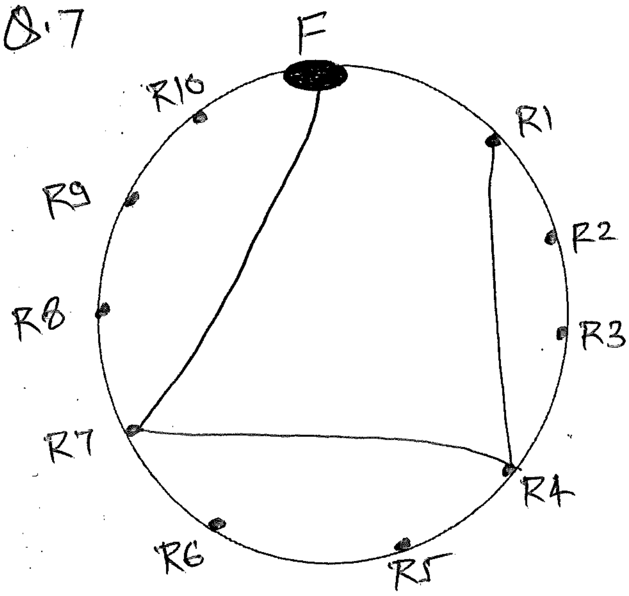

Q.8

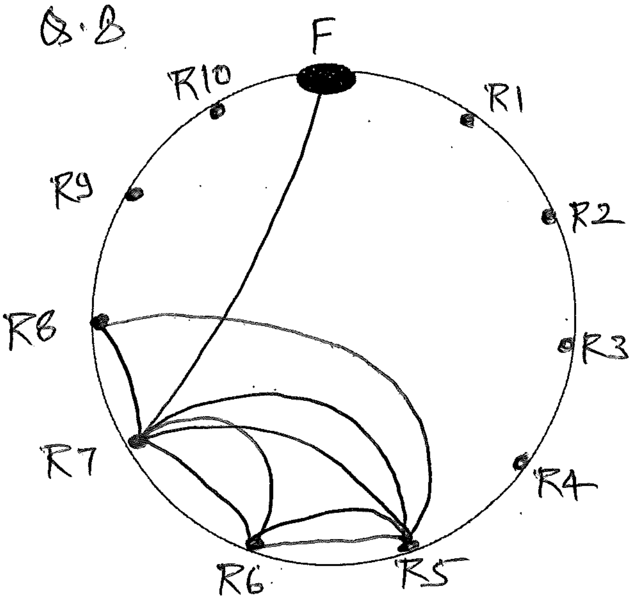

Q.9

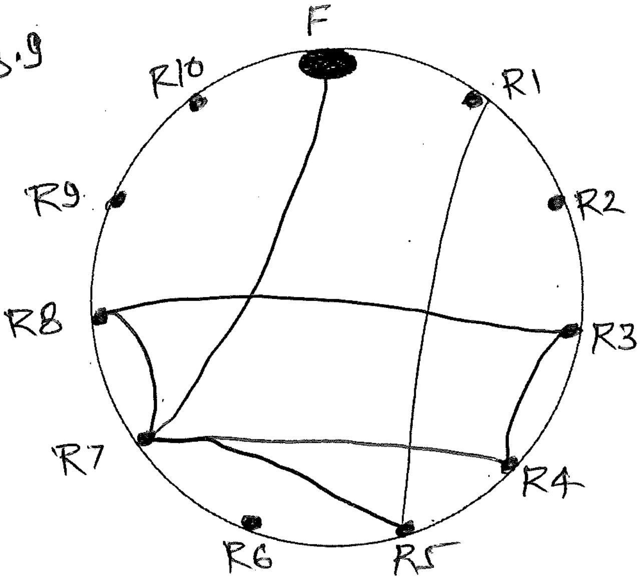

Q.10

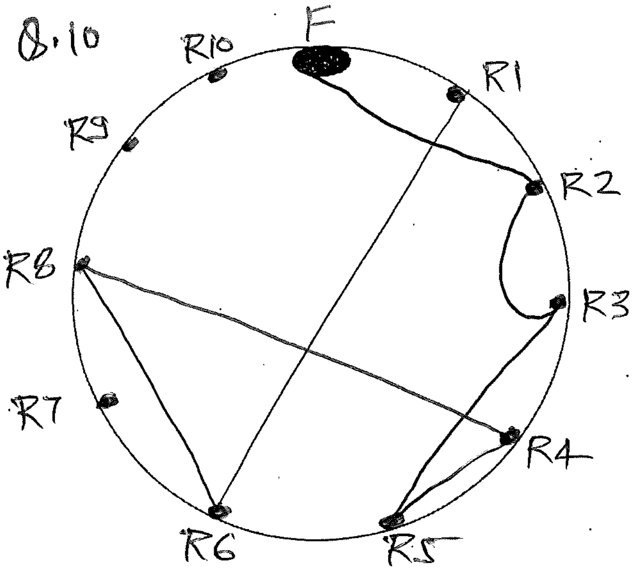

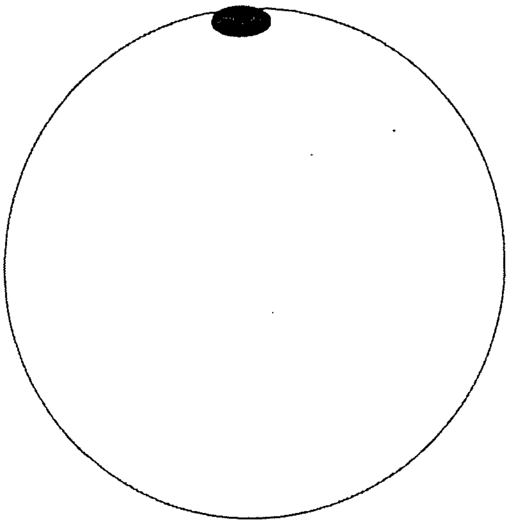

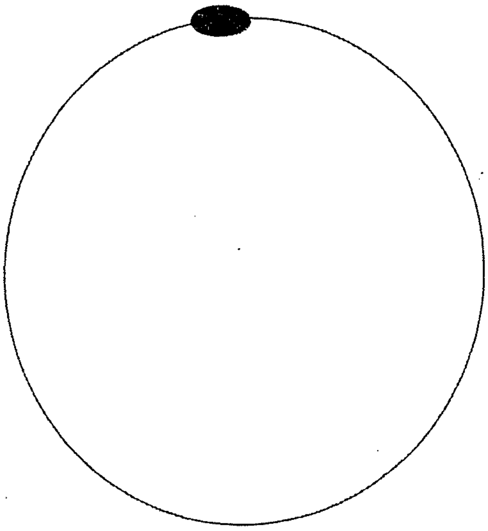

Supplement: S3 File — (PDF) [file pone.0240270.s003.pdf]
