# Supplementary material for: Grief reaction and psychosocial impacts of child death and stillbirth on bereaved North Indian parents: A qualitative study
Source: PLoS One. 2021 Jan 27;16(1):e0240270. doi: 10.1371/journal.pone.0240270 (PMC7840017; doi:10.1371/journal.pone.0240270)
Supplement: S5 File — (PDF) [file pone.0240270.s005.pdf]

Supplementary document 5
Causes of death in children and neonates

Sl. No.	Participant ID	Diagnosis (as per the hospital records)
**Children**		
1	Child 1	Meningitis
2	Child 2	Congenital heart disease (ventricular septal defect) with pneumonia
3	Child 3	Pneumonia
4	Child 4	Pneumonia with septic shock
5	Child 5	Pneumonia
6	Child 6	Congenital cyanotic heart disease with pneumonia
7	Child 7	Pneumonia
8	Child 8	Hepatic encephalopathy
9	Child 9	Malaria and Encephalopathy
10	Child 10	Acute gastroenteritis, severe dehydration and septic shock
11	Child 11	Recurrent pneumonia
12	Child 12	Renal failure
13	Child 13	Severe anaemia under investigation
**Neonates**		
14	Neonate 1	Term newborn with hypoxic ischemic encephalopathy-stage 3
15	Neonate 2	Pneumonia
16	Neonate 3	Septic shock
17	Neonate 4	Sepsis with meningitis
18	Neonate 5	Meningitis
19	Neonate 6	Septic shock
20	Neonate 7	Preterm, extremely low birthweight with sepsis
21	Neonate 8	Sepsis with meningitis
22	Neonate 9	Sepsis with pneumonia
23	Neonate 10	Septic shock
24	Neonate 11	Sepsis with pneumonia
25	Neonate 12	Septic shock
