# Supplementary material for: Grief reaction and psychosocial impacts of child death and stillbirth on bereaved North Indian parents: A qualitative study
Source: PLoS One. 2021 Jan 27;16(1):e0240270. doi: 10.1371/journal.pone.0240270 (PMC7840017; doi:10.1371/journal.pone.0240270)
Supplement: S6 File — (PDF) [file pone.0240270.s006.pdf]

Coding Tree

1. Grief anticipation and expression

- 1.a. Anticipation of death or stillbirth
  - Child/neonate
    - 1.a.1. No improvement in child’s condition
    - 1.a.2. Worsening in child’s condition or need for respiratory support
    - 1.a.3. Ongoing illness/past course
    - 1.a.4. Difficult labour or problem after delivery
  - Stillbirth
    - 1.a.5. High risk pregnancy
    - 1.a.6. Sudden loss of fetal activity
    - 1.a.7. Prolonged or difficult labour
- 1.b. Immediate emotional outpouring
  - Child/neonate
    - 1.b.1. Priming by the doctor/nurse
    - 1.b.2. Mother moved away from bedside or taken home
    - 1.b.3. Father or male member called (if not present)
  - Stillbirth
    - 1.b.4. Declared to father or other family member not mother
    - 1.b.5. Mother discharged and informed at home
    - 1.b.6. Mother didn’t appreciate non-disclosure
- 1.c. Physical reactions
  - 1.c.1. Parents experienced shock and extreme pain
  - 1.c.2. Mother fainted
  - 1.c.3. Mother cried loudly
  - 1.c.4. Some fathers cried openly or in isolation
  - 1.c.5. Fathers tried to support mothers
  - 1.c.6. Seen the baby before packing the body
- 1.d. Grief and reactions
  - 1.d.1. Disbelief
  - 1.d.2. Extreme pain
  - 1.d.3. Helplessness
  - 1.d.4. Feeling of vacuum
  - 1.d.5. Unsure how to react
  - 1.d.6. Unable to convince themselves

2. Impact of the bereavement

- 2.a. Guilt, remorse and blames
  - 2.a.1. Blaming self or family member for delay in care seeking
  - 2.a.2. Blaming self or family member for underestimating severity
  - 2.a.3. Regret of going to the hospital or doctor
  - 2.a.4. Blaming hospital or doctor for care
- 2.b. Changes in relationship
  - 2.b.1. Couples felt and understand each other
  - 2.b.2. Couples supported each other and increase in closeness
  - 2.b.3. Fathers/Husbands supported wives/mothers
  - 2.b.4. Neglected wife by husband and/or family members and marital disharmony
  - 2.b.5. Limited family support
- 2.c. Changes in lives and livelihood
  - 2.c.1. Extended illness or exacerbation of illness
  - 2.c.2. Experienced psychological/mental problem
  - 2.c.3. Sleeping disturbances
  - 2.c.4. Loss of appetite/eating problem
  - 2.c.5. Loss of concentration
  - 2.c.6. Absence from work or loss of work/wage
  - 2.c.7. Financial stress/ need for loan

3. Coping mechanism

Mother
- 3.a.1. Delay in resuming routine work
- 3.a.2. Keeping engaged in household chores and care of other children
- 3.a.3. Engaged in spiritual/religious activities
- 3.a.4. Weeping in isolation

Father
- 3.b.1. Resumed work to keep engaged
- 3.b.2. Avoiding discussion about the death with family and others
- 3.b.3. Remembering child in isolation

4. Sociocultural norms and practices

- 4.1. Burial and differences in ritual for children and stillbirths than adults
- 4.2. Women not allowed to the burial ground
- 4.3. Families coping through religious and spiritual engagement
- 4.4. Families doing donation/charity
- 4.5. Families blame witchcraft for the loss
