# Supplementary material for: Grief reaction and psychosocial impacts of child death and stillbirth on bereaved North Indian parents: A qualitative study
Source: PLoS One. 2021 Jan 27;16(1):e0240270. doi: 10.1371/journal.pone.0240270 (PMC7840017; doi:10.1371/journal.pone.0240270)
Supplement: Attachment — Submitted filename: Response to Reviewer and Editor comments-10122020.docx [file pone.0240270.s008.docx]

**Manuscript Ref no: PONE-D-20-30229**

**Title: Grief reaction and psychosocial impacts of child death and stillbirth on bereaved North Indian parents**

**Journal: PLOS ONE**

**Response to Editorial comments**

1. Please ensure that your manuscript meets PLOS ONE's style requirements, including those for file naming.

The PLOS ONE style templates can be found at

<https://journals.plos.org/plosone/s/file?id=wjVg/PLOSOne_formatting_sample_main_body.pdf> and

<https://journals.plos.org/plosone/s/file?id=ba62/PLOSOne_formatting_sample_title_authors_affiliations.pdf>

Response: We have made the necessary revisions in the manuscript style and file naming, as per the guideline.

2. Please ensure that you include a title page within your main document. We do appreciate that you have a title page document uploaded as a separate file, however, as per our author guidelines (<http://journals.plos.org/plosone/s/submission-guidelines#loc-title-page>) we do require this to be part of the manuscript file itself and not uploaded separately.
Could you therefore please include the title page into the beginning of your manuscript file itself, listing all authors and affiliations.

Response: We have included the title page in the main document as advised.

3. Thank you for stating the following in the Acknowledgments Section of your manuscript:

"This study is funded to The INCLEN Trust International by Bill and Melinda Gates
Foundation (OPP1184205) through Indian Council of Medical Research (no 5/7/1504/2016-
CH). The funders had no role in study planning, conduct, analysis and manuscript preparation."

We note that you have provided funding information that is not currently declared in your Funding Statement. However, funding information should not appear in the Acknowledgments section or other areas of your manuscript. We will only publish funding information present in the Funding Statement section of the online submission form.

Please remove any funding-related text from the manuscript and let us know how you would like to update your Funding Statement. Currently, your Funding Statement reads as follows:

"None"

Please include your amended statements within your cover letter; we will change the online submission form on your behalf.

Response: We have removed the funding statement from the main document. (Page 33, Lines 599-601)

The funding statement to be updated as follows. We request to update the funding statement online.

"This study is funded to The INCLEN Trust International by Bill and Melinda Gates
Foundation (OPP1184205) through Indian Council of Medical Research (no 5/7/1504/2016-
CH). The funders had no role in study planning, conduct, analysis and manuscript preparation."

4. Thank you for stating the following in your Competing Interests section:

"None"

Please complete your Competing Interests on the online submission form to state any Competing Interests. If you have no competing interests, please state "The authors have declared that no competing interests exist.", as detailed online in our guide for authors at [http://journals.plos.org/plosone/s/submit-now](about:blank)

This information should be included in your cover letter; we will change the online submission form on your behalf.

Please know it is PLOS ONE policy for corresponding authors to declare, on behalf of all authors, all potential competing interests for the purposes of transparency. PLOS defines a competing interest as anything that interferes with, or could reasonably be perceived as interfering with, the full and objective presentation, peer review, editorial decision-making, or publication of research or non-research articles submitted to one of the journals. Competing interests can be financial or non-financial, professional, or personal. Competing interests can arise in relationship to an organization or another person. Please follow this link to our website for more details on competing interests: [http://journals.plos.org/plosone/s/competing-interests](about:blank)
Response: Please update the authors Competing Interest statement to

"The authors have declared that no competing interests exist.”

5. We note that you have indicated that data from this study are available upon request. PLOS only allows data to be available upon request if there are legal or ethical restrictions on sharing data publicly. For information on unacceptable data access restrictions, please see <http://journals.plos.org/plosone/s/data-availability#loc-unacceptable-data-access-restrictions>.

In your revised cover letter, please address the following prompts:

a) If there are ethical or legal restrictions on sharing a de-identified data set, please explain them in detail (e.g., data contain potentially identifying or sensitive patient information) and who has imposed them (e.g., an ethics committee). Please also provide contact information for a data access committee, ethics committee, or other institutional body to which data requests may be sent.

b) If there are no restrictions, please upload the minimal anonymized data set necessary to replicate your study findings as either Supporting Information files or to a stable, public repository and provide us with the relevant URLs, DOIs, or accession numbers. Please see <http://www.bmj.com/content/340/bmj.c181.long> for guidelines on how to de-identify and prepare clinical data for publication. For a list of acceptable repositories, please see <http://journals.plos.org/plosone/s/data-availability#loc-recommended-repositories>.

We will update your Data Availability statement on your behalf to reflect the information you provide.

Response: We are submitting the transcripts of IDIs and FGDs as supplementary file (S2 File).

We are submitting the perinatal grief scale scores for the participants as supplementary file (S4 File).

6. Your ethics statement should only appear in the Methods section of your manuscript. If your ethics statement is written in any section besides the Methods, please delete it from any other section.

Response: We have updated the Ethical considerations section in the main text (page 12, lines 185-190) and deleted the ethics statement from Acknowledgement/Declaration section (Page 33, Lines 586-590).

7. Please include captions for your Supporting Information files at the end of your manuscript, and update any in-text citations to match accordingly. Please see our Supporting Information guidelines for more information: [http://journals.plos.org/plosone/s/supporting-information](about:blank).

Response: We have added the section on Supplementary files/documents with the titles (Page 41). We have also mentioned the Supplementary files/documents in the main text as appropriate.

Reviewers' comments:

Reviewer #1: This is a well-written qualitative paper which aims to study the social, emotional, and psychological impact of child death and stillbirths on parents and their families along with the coping strategies in North Indian context. I appreciate the authors for their commendable work as this is particularly important and less investigated topic of women and child health.

Response: Thank you very much for the encouraging words.

Comments
Title
I would suggest the authors to add “a qualitative study” at the end of the title as the readers will know the type of study was conducted for this research question.

Response: As suggested, we have revised the title and added “a qualitative study”.

The revised title now reads as “Grief reaction and psychosocial impacts of child death and stillbirth on bereaved North Indian parents: a qualitative study” (Title, Page 1 and Page 5)

Abstract
The abstract summarizes the rationale for the study and the methods used for this study, as well as the key findings.

Response: Thank you. No action needed.

Introduction
The introduction includes important information about the global burden of neonatal and infant mortality, the bereavement and grief caused to the mother and family after infant death and shows the paucity of information on the social and psychological impact after child death in North India.

Line 54- I would suggest the authors to write few more lines on the epidemiology of child death in India.

Response: As suggested, we have added few lines on the epidemiology of child death and stillbirths in India. (Introduction, page 7, lines 54-61)

At the end of line 54, authors should discuss about the consequences of child death in the family and then the statements on bereavement and grief should be discussed, as it would give the readers a sense of continuity. Right now, it looks separate because the burden was discussed in the initial few lines of Introduction followed by definition of bereavement and grief.
Response: We have added a sentence after the mortality epidemiology to link between the two segments. The bereavement and grief follows the sentence.

The sentence added is “Death of a child is one of the most severe, shattering and overwhelmingly painful event for parents.” (Introduction, Page 7, line 63-64)

Line 85- Authors had mentioned “causes of a large proportion of child, infant deaths and stillbirths remain unknown”. This statement should be removed as the leading causes of infant and neonatal deaths are well-documented. I would suggest the authors to write a few lines on the common causes of neonatal and infant deaths in India along with the description of epidemiology of child deaths in the 1st paragraph of Introduction.

Response: As suggested by reviewer, we have removed the statement and added the epidemiology in 1^st^ paragraph. (Introduction, page 7, lines 54-61)

Methods
The methods section provide details on how the participants were chosen, how the interview was conducted, and all the required information needed to understand the whole process of the study.
Line 114- I would suggest the authors to provide some information on how many refused to participate in the study among those who were contacted to find whether there is any systematic difference in the socio-demographic characteristics between the participants and non-participants.

Response: In total during the reference period, there were 45 child deaths, 52 neonatal deaths and 60 stillbirths. Out of these 69 parents were not traceable due to incomplete address or no contact number. We were able to contact 30 eligible parents with child death, 28 parents with neonatal death and 40 parents with stillbirth. Out of these approached, 13 parents with child death, 12 parents with neonatal death and 22 parents with stillbirth consented for IDI. We have added the same to text also. (Methods, page 11, lines 129-134)

Line 143- There is no information on the internal validity (Cronbach’s alpha) of the scale used in this study.

Response: The Cronbach’s alpha estimated was 0.91 for the perinatal grief scale used among the participants. This has been added to the text. (Methods, Page 13, lines 194)

I suggest the authors to comment on the fidelity of the transcription. Whether it was read out to participants after each session to check for correctness or any other method was adopted.

Response: The questions were readout to the participants and the responses were recorded. Any response needed clarification were clarified or needed further exploration were explored or probed. These have been added to the text. (Page 12, lines 169-172)

While transcription, the audio recordings were used. The transcripts were checked by another research team member for correctness. This has been mentioned in the text. (Page 11, lines 178-179)

There is no information regarding whether any reimbursement/ incentives were given to the participants (E.g. For transport, refreshments etc.) and also, I request the authors the mention a word on how data saturation was reached in the interviews and discussion.

Response: No payment or reimbursement/incentive was made to any participant. The statement has been added in the text. (Page 12, Lines 172)

Regarding data saturation, we have added the statement in Methods section. (Methods, Page 10, Lines 150-152).

Though it was mentioned in the study’s protocol, it is worth mentioning who were the study participants for 8 FGDs in the methods section (Men and women with under-five children and older family members fathers, mothers and their family members) for easy understanding of the readers.

Response: We have added the participants for FGDs in the methods as suggested. (Page 11, Lines 135-137)

The authors should describe the distribution of participants in the FGD, whether any group (mothers/ grandparents/fathers) dominated the discussion. It is important to mention this in the methods section as it is susceptible to bias. The group and individual opinions can be swayed by dominant participants. Also, it is good to mention how this was taken care during the FGDs.

Response: The facilitator tried to moderate the discussion among participants and encouraged all participants to contribute. The quality of discussion was documented by sociogram. We have added this to the text in Methods section. (Page 12, Lines 158-159). The sociograms are attached as supplementary document (S3 File).

Results
The data appear to be sound and the results section elaborately presents the output of the processes described in the methods section.
Line 176- There is a discrepancy in the number of participants for FGDs. In methods section line 131, says 8-11 participants. This should be corrected.

Response: The distribution of participants for FGDs were:

- Fathers: Total 18 (FGD-1, n=9, FGD-2, n=9)
- Mothers: Total 19 (FGD-1, n=11, FGD-2, n=8)
- Grandfathers: Total 17 (FGD-1, n=9, FGD-2, n=8)
- Grandmothers: Total 18 (FGD-1, n=9, FGD-2, n=98)

Thus we have written the participants as 8-11 in the methods section.

Table 1- Point 1.4, Literacy of father, the 3rd column does not add up to 46. The sub-heading age in years and median (IQR) and n (%) should be shifted to 3rd column for easy understanding.
Response: We apologise for the typo error. The number for category <5^th^ standard is 6. It has been corrected.

The correction in the table has been done as suggested. (Table 1, Page 14-16)

Line 172- I would suggest the authors to mention the cause of death as per the hospital record for all the 25 child deaths, to understand the leading cause of death among the children of recruited family.

Response: The causes of death for the 25 children (12 neonates and 13 post-neonatal children) have been added as supplementary document (S5 file). The same has been added to the text in Results section. (Page 14, Lines 212-213).

Line 189- It would be good to show the coding tree to describe how the codes that were generated from the data were translated into categories and then to themes.
Response: The coding tree of the themes and categories are given as supplementary document (S6 File). The same has been added to the text in Results section. (Page 16, Lines 230-231).

Line 284- “Mothers were supported by husbands and family members”. Does it include in-laws as well? I would suggest the authors to mention whether any support was given to the mothers from their in-laws during the grief period.

Response: The mothers were supported by in-laws and also their mothers and sisters. The same has been added to the text as suggested. (Page 20, Lines 323-324)

Table 2- It is better to use test of significance to find whether there is statistically significant difference exists among the groups.

Response: The mothers with stillbirth had higher PGS scores for total and subscales than the mothers with child/neonatal death. Statistical significant differences were observed in the total PGS score (p=0.02) and one sub-scale (despair, p=0.02) between the mothers with stillbirth and mothers with child/neonatal death. It has been added to the text in Results. (Page 23, Lines 403-405)

The discussion and conclusion are well balanced and adequately supported by the data.
Lines 510 and 527- should be specific and not generic. I suggest the authors to write specific recommendations at the family level, community level and health system level and what should be done to address various factors as mentioned in the results section associated with grief, by whom and at what level of healthcare system this should be implemented.

Response: We thank reviewer for the suggestion. We have added the recommendations for different levels, as advised. We hope that these are appropriate. (Page 30, Lines 553-561)
